# In the shadow of the “psychopharmacological revolution”: Malaria fever, insulin coma, cardiazol and electroconvulsive therapy at the Vienna Psychiatric University Clinic, 1951–1969

**DOI:** 10.1007/s00508-025-02592-w

**Published:** 2025-10-02

**Authors:** Gernot Heiss

**Affiliations:** Institute of History, University of Vienna, Universitätsring 1, 1010 Vienna, Austria

**Keywords:** History of psychiatry, Somatic therapies in psychiatry, History of psychopharmakology, Hans Hoff, Psychiatric ethics

## Abstract

The study presents the results of a 2-year research project on the therapeutic practices in psychiatry at the University Clinic of Vienna under the leadership of Hans Hoff from 1951 to 1969. Hoff took over as Head of the Department of Psychiatry and Neurology shortly after his return to Vienna from whence he had had to flee after Austria’s “Anschluss” (annexation) to Nazi Germany in 1938 because of his Jewish background. The focus of the investigation was on the extent, methods and justifications for the application of four highly invasive somatic treatments, which were developed during the interwar period and continued to be practiced at the Vienna Clinic during the years under study. These treatments included electroconvulsive therapy (ECT), cardiazol-induced convulsion therapy, insulin coma therapy and malaria fever therapy, the latter also used beyond its traditional application for neurosyphilitic conditions. The study analyzes the medical records of patients from the adult departments (aged 15 years and older) who were diagnosed with neurosyphilis, intellectual disabilities, schizophrenia, affective disorders, or psychopathy (a total of 14,919 patient records). Additionally, it examined how these therapies were discussed in professional publications from Austria, Switzerland, Germany, France, and Great Britain, and to what extent their application was considered state-of-the-art during the transition to new pharmacological treatments.

**Supplementary Information:**

The online version of this article (10.1007/s00508-025-02592-w) contains supplementary material, which is available to authorized users.

## Background: the research project

The four highly invasive therapies developed in the interwar period were used at the Vienna Psychiatric-Neurological University Clinic under the direction of Hans Hoff [1]^1^ until the end of the 1960s. At other clinics, such as in Zurich [2, pp. 93–5], their use had already been greatly reduced or abandoned in the 1950s. The following remarks present the results of a 2-year research project (2013–2014),^2^ in which the number of and indications for these therapies, the expectations of Viennese clinicians regarding their therapeutic effect and the scientific discussion of the four therapies in these two decades were examined as the basis for a critical historical assessment. The project was initiated by Rector Wolfgang Schütz and Vice-Rector Christiane Druml of the Medical University of Vienna after it was claimed in the media that young patients were infected with malaria at the psychiatric department in the 1960s solely for research purposes, without therapeutic indications.^3^ The aim was to elucidate the use of the four somatic therapies during two decades of fundamental therapeutic change in psychiatry, namely the transition to neuroleptics from 1952, to antidepressants from 1958 and also the transition from malaria fever therapy in the treatment of neurosyphilis, to penicillin, from the 1940s [3, pp. 281–5].^4^

Sources used in this study were the medical records of the two adult wards of the clinic (kept in the archives of the University Clinic for Psychiatry and Psychotherapy of the Vienna General Hospital) for the years 1951–1969 as well as contemporary medical literature, supplemented by individual interviews and written inquiry responses from doctors with first-hand knowledge of the clinic during the period of interest.

Around 90,000 medical records from the 2 adult wards during the years 1951–1969 have been preserved. As a rule, the patients were aged 15 years and over, with some exceptions as young as 13 and 14 years old. The loss of files is estimated at just over 20%. For the statistical analysis, a database was created in which data relevant to the project were entered from the medical records of patients who had stayed at the clinic for 5 days or more, and who had been treated with malaria fever therapy and/or had received one of the five following diagnoses: neurosyphilis, intellectual disabilities, or schizophrenic, affective and psychopathic disorders. These five diagnoses were the main indication fields in Vienna of the “‘large’ somatic treatment procedures carried out as cures” [4, p. 151] which were, including the malaria fever therapy as ‘non-specific’ therapy outside neurosyphilis, usually referred to as shock therapies at the time. Regarding the inclusion criterion length of stay, the Vienna clinic was a ‘transit ward,’ from which around two thirds of patients were transferred to a psychiatric hospital or discharged within the first few days. Of the patients who stayed longer at the clinic, around half had a diagnosis from these five diagnostic fields.

There are 11,720 patients who had 14,919 hospital stays of more than 4 days (each admission counted as a case). Of these, in 6915 cases (46.3%) the above mentioned 4 highly invasive therapeutic interventions were applied. A total of 7755 convulsive, coma and fever cures were administered, including sequences and combinations of several of these therapies during a single stay (10.8%). Patients with a diagnosis from the 5 diagnostic fields on which the study focused received electroconvulsive therapy with 1 or more application 5886 times, malaria fever therapy 772 times and insulin therapy 965 times (in the majority of cases this consisted of insulin coma therapy, for which the clinic was responsible and insulin sub-coma therapy in a proportion estimated at less than 10%). Cardiazol convulsant therapy was used in more than 114 cases.

## Malaria fever therapy

Malaria fever therapy was developed as a treatment for progressive paralysis at the end of the First World War at the Vienna clinic under Julius Wagner-Jauregg, following a long period of studying the effects of fever on psychoses and other mental disorders. The success of the therapy and the awarding of the Nobel Prize for Medicine to Wagner-Jauregg in 1927 greatly enhanced the international reputation of Viennese psychiatry. The international discussion and widespread recognition of this invasive somatic therapy led to an increased willingness within psychiatry, which until then had had few therapeutic options, to experiment with treatments that carried significant risks for patients, often caused them considerable pain and triggered fear. In the early 1920s, continuous sleep therapy using the barbiturate Somnifen (F. Hoffmann-La Roche, Basel, Switzerland) was introduced by Swiss psychiatrist Jakob Klaesi; malaria fever therapy was also applied to other psychiatric illnesses and their symptoms as a kind of “shock treatment” to bring about a change of state (“*Umstimmung*”). In the 1930s, the three new “heroic therapies” were developed [5, pp. 18–27]^5^, which alongside malaria fever therapy are the focus of this contribution.

Malaria fever therapy was the third most common of the four therapies in use on the adult wards during the period under investigation. In total, 869 cases of malaria fever therapy are recorded in the project’s database, including the previously mentioned 772 cases with 1 of the 5 main diagnoses, and a further 97 cases of patients with other diagnoses, such as alcoholism, neurotic illness, states of neglect or depravation (*Verwahrlosung*)^6^. As malaria fever therapy was only given a second time in rare instances, the number of cases, as with coma therapy and in contrast to convulsive therapies, roughly equals the number of patients. Its application reached a low in 1959 but rose again in the 1960s to between 35 and 45 applications per year. The last recorded use was in mid-December 1968, shortly before the end of the “Hoff era”: Hans Hoff died on 23 August 1969 while still on active duty. In what follows, we will consider first the application of malaria fever therapy for neurosyphilis (for which it was originally introduced) and then for other conditions.

The rationale for malaria fever therapy in neurosyphilis was that the fever was expected to kill or weaken the syphilis bacteria (*Treponema pallidum*) and to strengthen the immune system in patients with neurosyphilis through temperatures above 40 °C; the success of the malaria fever therapy alone was estimated by its proponents to lead to a cure in one third of cases, and to an improvement in another third. In Vienna malaria fever therapy was still used during the study period for neurosyphilis, above all for progressive paralysis but occasionally for taboparalysis and tabes dorsalis but almost exclusively in combination with penicillin. In the international contemporary discussion from the 1940s to the 1960s, the question was whether this combination would bring the same or better results than penicillin alone. Hans Hoff and other psychiatrists held that the combination brought significantly better clinical outcomes [6, 7]. Others saw the adherence to the combination as a reluctance on the part of older psychiatrists to completely abandon ‘tried and tested’ therapies in favor of new therapies: that it was a generational problem [8].

Eventually, the treatment of neurosyphilis with penicillin alone became generally accepted. The opinion had prevailed among many doctors that the results were just as good with the simpler and gentler therapy as with the combination. Moreover, it was also highly significant that the cure of syphilis with penicillin in earlier stages meant that there were few patients suffering from neurosyphilis. According to the database, the number of patients with neurosyphilis at the Vienna clinic fell in the 1950s from 65 in 1951, 35 of whom received malaria fever therapy, to 8 in 1959, 4 of whom received malaria fever therapy.

Maintaining a tertian malaria strain using only patients with neurosyphilis, as was still practiced at other clinics in the 1950s^7^, was no longer possible, as this required an unbroken chain of blood transfers from one person to the other and there were not enough patients with neurosyphilis at hand. Expressions of regret about the lack of suitable blood infected with malaria parasites for the treatment of progressive paralysis can be found several times in the specialist publications [10].

As with other somatic therapies, the application of malaria fever therapy was extended beyond its original indication and came to include intellectual deficiencies, schizophrenia and affective and psychopathic disorders. The frequent use in patients without neurosyphilis is the reason why in Vienna, a “syphilis-free” tertian malaria strain [11, p. 140]^8^ was maintained at least until 1968, with a brief interruption in 1959.

The abovementioned figures in the 1960s and their regularity suggest the preservation of the malaria strain in patients who were selected for malaria fever therapy but could have been treated with the new psychotropic drugs (neuroleptics, antidepressants) as an alternative. Whether this was indeed the case, the possibility of individual patients being infected without therapeutic indications only to preserve the malaria strain, was one of the questions we tried to answer in our project. One indicator was the fact that patients being sent back from the City of Vienna Psychiatric Hospital Baumgartner Höhe (*am Steinhof*) to the University Clinic specifically for malaria fever treatment and then back again were referred to in the records both from the clinic as from the “Steinhof” hospital as “hosts” (*Stammträger*).^9^ It is therefore evident that these patients served as regular “‘hosts” for the malaria strain; however, a therapeutic indication for malaria therapy cannot be ruled out as other patients with the same diagnosis and without similar notes in their medical records also received the therapy and furthermore with or without being used as “hosts” through the transfusion of malaria-infected blood to another patient. Therefore, the question of whether the patients were used solely as strain carriers cannot be answered conclusively.

There was no detailed discussion of the indication of malaria fever treatment for the four mentioned nonsyphilitic diagnoses (intellectual deficiencies, schizophrenia, affective and psychopathic disorders) in the contemporary literature; only brief references to its use for these diagnoses were made in the publications consulted or, in the case of “psychopathy”, no references were found at all.

There are isolated references to the use of malaria fever therapy in neurosyphilis and also in the follow-up treatment of polio after Kauders [12, p. 179]^10^ at other hospitals, using malaria-infected blood drawn from someone without a syphilis infection, provided by the University Clinic. Although its use in psychiatry in Vienna for these diagnoses was quite frequent (in 609, i.e. almost 79% of the 772 cases), the only detailed explanation for the use of malaria fever therapy found in this period was in the case of children with intellectual disabilities in a publication by the then head of the Vienna University Children’s Clinic [13]. From the children’s ward of the Vienna Psychiatric-Neurological University Clinic, whose surviving files enabled the creation of a separate database for the project, and to which a separate article is dedicated in the 2015 project report, early childhood cerebral impairments resulting from birth trauma, encephalitis, postnatal jaundice and conditions classified under the diagnostic category of intellectual disabilities were diagnosed in 22 out of 35 patients who were treated with malaria fever therapy.^11^ They were mostly private patients from abroad who, after unsuccessful treatment attempts elsewhere, were admitted to the clinic, often through Hans Hoff’s private practice. Their frequent origin from Arab and Turkish regions was likely due not only to the international reputation of Viennese psychiatry but also to Hoff’s connections stemming from his early exile and professional activity in Baghdad.

In the Federal Republic of Germany, the use of malaria fever therapy for non-syphilis-related diseases likely ended as early as the mid-1950s with the introduction of neuroleptics^12^, at a time when it was still being used alongside penicillin for the treatment of neurosyphilis. This practice also came to an end in the Federal Republic of Germany (FRG) by the late 1950s [14], whereas the broad application of malaria fever therapy (for both syphilis and non-syphilis-related diseases) can be traced in Vienna up until 1968. In 1948, before the development of the neuroleptic chlorpromazine (Largactil), the Zurich psychiatrist Manfred Bleuler defined “shock and fever therapies” as “concussion therapies” that were useful for treating “all psychopathic conditions that cannot yet be treated causally” [15, p. 147]. In the 1960 edition of his father Eugen Bleuler’s textbook on psychiatry (which he edited), he wrote of “rarely effective” applications of malaria fever therapy outside of progressive paralysis, but without denying its justification if other physical therapies had failed [4, p. 405].

The therapeutic goals of malaria fever treatment in cases of non-syphilitic illness were described in medical records, scientific literature, and eyewitness reports as aiming to induce a shift in psychological state (*Umstimmung*)^13^. This included patients at risk of suicide or those who often were severely agitated but also cases where the therapy sought to bring patients out of states of stupor or to stimulate psychological development in adolescents with delayed maturation. The therapy was believed to exert a kind of “stress” on the organism, which in turn would trigger a general adjustment process, providing new impulses for psychophysical development and overcoming developmental delays (*Reifung*) [11, p. 140, cf. 13, p. 425, 19, pp. 60–1 and 4, p. 159].^14^

Malaria fever therapy was used to treat motor-neurological symptoms in children with intellectual disabilities [16, p. 449–50], cases of so-called *Pfropfschizophrenie* [11, p. 140, 19, p. 60–1], and adolescents diagnosed with psychopathy and delayed intellectual development [13, p. 425]. Intensive contact between doctors and nursing staff on the one hand and the patient on the other during the treatment was expected to open the patient up to psychotherapeutic intervention.^15^

*Plasmodium vivax* (the causative agent of tertian malaria) was transmitted to mainly male patients with diagnoses and symptoms that were treated elsewhere with the new psychotropic drugs and/or psychotherapy or with other physical cures, electroconvulsive or insulin coma therapy, sleep or twilight cures. At the same time, in Vienna, in the vast majority of cases with diagnoses other than neurosyphilis that met the inclusion criteria of this project, no malaria fever therapy was given. Female patients with one of the four non-neurosyphilis diagnoses were treated with malaria fever therapy in only 13 cases (out of 596 patients); no explanation was found for this comparatively rare use in women. In neurosyphilis cases, where malaria fever treatment was assumed to be specifically and causally effective, the ratio was much more balanced, with 51 women to 112 men.

The indication decision for the non-specific somatic therapies, as for malaria fever therapy outside neurosyphilis, was made based on an assessment of symptoms and not on the ultimate diagnosis.

## Insulin coma therapy

Insulin coma therapy, which had been developed by Manfred Sakel at the Vienna Clinic in the mid-1930s, had been the subject of extremely controversial debate since its introduction but nevertheless quickly became established worldwide. In the “Hoff era,” it was the second most common of the “grand old cures” at the Vienna clinic. Hans Hoff wrote in 1953 and 1958 about his assessment of the therapy when he proposed to the Nobel Committee that Manfred Sakel and Ugo Cerletti be awarded the Nobel Prize for Medicine (without success): “through the knowledge of shock therapy […] the complete change in psychiatry from a science that was content with purely descriptive accounts of clinical pictures to a highly active therapeutic part of medicine […] was initiated”; it was Sakel’s “merit to have shown that there is a possibility of curing schizophrenia.”^16^

As with malaria fever therapy for progressive paralysis, at the Vienna Clinic insulin coma therapy was expected to cure schizophrenics if used within 2 years of the first onset of the disease. It was believed that insulin coma, repeated daily (except Sunday) and at least 50 times, would block the glucose uptake of the nerve cells and thus their maintenance metabolism, in such a way that those cells “in lesion” underlying the schizophrenic process, would not have enough time to recover [17, p. 263 and pp. 267–8].

After the introduction of neuroleptics (1952/1953) and due to the specification and restriction of the indication [18, p. 507, 19, p. 168–9] (possibly also due to the scepticism of individual clinicians about the effectiveness of the therapy) [21, p. 60]^17^, the use of insulin coma therapy gradually decreased: according to the project’s database, from 95 insulin treatments in 1951, to 58 in 1959, 43 in 1960 and 16 in 1968. Viennese clinicians, however, made a significant contribution to improving its use in reducing the number of undesirable side effects by using the hexamethonium derivative depressin as a ganglion blocker to significantly reduce insulin tolerance [22]. In the final years of the “Hoff era,” patients were no longer given a sugar solution by nasogastric tube to break the coma but were injected intramuscularly with glucagon [23]. Ottokar H. Arnold, who had contributed a great deal to the specification of diagnostics and indications as well as to the technique of application and who published mainly on schizophrenia, emphasized in his “overall treatment plan” [19, pp. 168–9]^18^ that, depending on the type of schizophrenia and the course of the process, other convulsive therapies (cardiazol and electroconvulsive therapy), psychosurgical methods (lobotomy), fever therapy (malaria therapy), treatment with psychotropic drugs and with psychotherapeutic methods (individual and group therapies), “complementary methods” (occupational and sports therapy, gymnastics, dance and music therapy) and sociotherapy would have to be integrated; however, it remained clear to him, as he wrote in 1960, that “even today […] the correctly indicated and administered full insulin shock cure is the basic treatment for schizophrenia” [17, p. 262].

In the 1950s and 1960s the effectiveness of the insulin cure was disputed [24, 25] or assumed to be very poor by some practitioners but was still considered indispensable by others [26, p. 936]. In Zurich, its use, like that of malaria fever therapy, came to an end at the end of the 1950s, in Vienna only around 1970, after the end of the “Hoff era,” and in other countries, such as the German Democratic Republic (GDR), Japan and Russia, much later [27, pp. 224–5, 28, p. 213]. The long use of insulin coma therapy in Vienna, as with malaria therapy, was probably due to the experience of clinic’s director Hans Hoff, who had been involved in its development in the 1930s [29, 30] and considered it a proven successful therapy.

## Cardiazol and electroconvulsive therapy

The cardiazol convulsive treatment developed by the Hungarian psychiatrist Ladislas Joseph Meduna in 1934 and the electroconvulsive therapy first presented by Ugo Cerletti in Rome in 1938 were both used in Vienna in the 1950s and 1960s. Cardiazol convulsive therapy was usually only administered in insulin coma during the period under investigation due to the very unpleasant “agonising sensations” [31, p. 123–25] that preceded it [32, p. 516]. One exception, for which no further examples were found in the files or in the literature consulted, was with two patients in 1956 who were awakened from a difficult to release coma after a suicide attempt; another exception in 1952 and 1957 was the use of a “cardiazol electroshock” in two patients with schizophrenia diagnoses [19, p. 45].

The last of the four great old cures of the interwar period was electroconvulsive therapy (ECT). Since its introduction by Ugo Cerletti in 1938, ECT has been the subject of controversy not only in specialist circles but also in the media. Nevertheless, its use spread rapidly worldwide and it was the most frequently used of the four therapies in Vienna during the period under investigation. At the beginning of the 1950s, Viennese psychiatrists were already convinced that ECT, using the muscle relaxant lysthenone, was a low-risk and highly effective method of treating certain schizophrenic and manic-depressive illnesses and their symptoms. In 1952, Hans Hoff wrote of the successes in shortening the phases of melancholia, in the treatment of acute fatal catatonia and in calming agitated patients, which considerably simplified care in psychiatric clinics and sanatoria; 19however, the number of applications, which affected schizophrenics and patients with affective disorders in almost equal proportions, fell significantly during the period under investigation: from 382 patients receiving ECT in 1951, to a peak of 469 in 1955 and 366 in 1959, a continuous decline can then be seen from 1960 with 357 applications, down to 123 in 1969; however, its use remained very high in Vienna in the 1960s compared to the Zurich University Hospital [2, pp. 93–5], for example. Although neuroleptics had already been incorporated into treatment in the 1950s, the opinion at the clinic was that most of the drug therapies would cause more undesirable side effects and complications than electroconvulsive therapy alone [33, p. 289].

One reason for this judgment was the severe side effects of early neuroleptics, combined with significant improvements in the administration of ECT. The Viennese clinician Ottokar H. Arnold had already contributed significantly to this progress in the early 1950s by using lysthenon as a muscle relaxant during ECT. This helped prevent fractures caused by convulsions during the procedure. Viennese clinicians also clarified the indications for ECT in published works, for example, Arnold himself in 1949, when he recommended its use for acute, life-threatening catatonia [34].

## Conclusion

Although the “psychopharmacological revolution” influenced Viennese psychiatry during the period under investigation and led to a decline in the use of the four major physical therapies introduced during the interwar years, these treatments nonetheless remained central. They continued to be used alongside the new medications and, in the case of malaria fever therapy for neurosyphilis, also in combination with penicillin.

In addition to the established “major” therapies, the new psychopharmaceuticals were rapidly adopted at the Viennese Clinic.^20^ For example, the groundbreaking neuroleptic chlorpromazine (Largactil) received highly positive evaluations in Vienna in the very year of its introduction (1952) [35, 36] and was soon incorporated into comprehensive treatment protocols, as demonstrated by Ottokar H. Arnold’s 1963 indication schemes for schizophrenia [19, pp. 168–9]. These schemes also underscore the central importance of socializing therapies in Viennese psychiatry, particularly occupational therapy, which appears frequently in patient records as standard practice but also gymnastics, sports, dance and music therapy. During the rehabilitation phase, Arnold’s schemes assign a prominent role to (outpatient) group and family therapies, especially bifocal group therapy, which had been initiated by Raoul Schindler in the late 1940s and further developed at the Viennese Clinic in the 1950s in collaboration with Ottokar H. Arnold [37]. Although these psychotherapeutic interventions were widely discussed in the professional psychiatric literature by Viennese psychiatrists, they were rarely mentioned in patient records, as they generally took place after discharge, in outpatient centers, medical practices, or rehabilitation centers under the supervision of psychiatrists from the “Hoff Clinic”.

The long use of malaria fever therapy and insulin coma therapy (until 1968/1969 and 1970, respectively) compared with therapeutic practices at other clinics, can probably also be understood from Hoff’s position in the tradition of Viennese psychiatry. It was here that the two therapies had been developed and with them Viennese psychiatry had gained a worldwide reputation. Like his predecessor Otto Kauders, Hans Hoff was part of this tradition. Hoff had been involved in the development of both therapies, malaria fever therapy under Julius Wagner-Jauregg [38] and under Otto Pötzl, and as a close collaborator of Manfred Sakel, in the development of insulin coma therapy [29].

He programmatically aligned himself with the Viennese tradition of Theodor H. Meynert, Constantin von Economo, Wagner-Jauregg and Pötzl, which was characterized by a dominant organic-biological perspective [39], for example, in his inaugural lecture (12 October 1950) [40, p. 5] and in an essay of 1954, where he sought “to reconcile” and develop the clinical tradition with the Viennese “schools of [Sigmund] Freud and [Alfred] Adler” [41].^21^ In Hoff’s “overall treatment plan,” both “organic-biological and psychotherapeutic, psychodynamic, and social-psychiatric treatment approaches were integrated” [42, p. 40–1]. While traditional and emerging organic-biological therapies dominated clinical practice, it was the psychohygienic and social-psychiatric approaches that defined his many initiatives to establish or expand institutions beyond the clinic. These included the convalescent home for alcoholics in Kalksburg, the special facility for the treatment of mentally abnormal offenders in Mittersteig, the school psychology service, marriage and family counseling centers, and the crisis intervention center, among others [21, pp. 61–2].

## Supplementary Information

The extensive German-language supplement presents a thoroughly revised and significantly expanded version of the unpublished 2015 project report. It includes bar charts and statistical tables based on the analysis of the database, detailed discussions of contemporary debates on ‘heroic therapies’ and the introduction and use of new psychotropic drugs, as well as numerous case studies drawn from patient records

## Footnotes

1 For more information about Hoff [1].

2 Heiss G. Die Anwendung somatischer Therapien in der Psychiatrie an der Wiener Klinik in der “Ära Hoff” (1951–1969). Malariafieber‑, Insulinkoma‑, Elektro- und Cardiazolkrampf-, sowie Psychopharmakatherapie in der Praxis und der wissenschaftlichen Diskussion. See Supplementary Material.

3 See, for example, Der Standard, Wien, 06.02.2012; Kurier, Wien, 07.02.2012; Die Presse, Wien, 10.02.2012.

4 For a periodization of the history of psychiatry from 1870 to 1970, [3, pp. 281–5].

5 As editors of the anthology, Hans-Walter Schmuhl and Volker Roelcke provide, based on key findings from the contributions on various European countries, a highly informative introductory overview of the development, whereby the psychiatric discourse on war neurotics is seen as a crucial turning point toward the new attitude.

6 In rare cases malaria fever therapy also was given to patients having the diagnoses of epilepsy, delirium tremens, arteriosclerotic confusion, organic dementia, but also psychological consequences of skull injuries or poisoning.

7 Hippius H. E‑mail to Gernot Heiss 2014 Aug 12. (Archive Josephinum Vienna) about this practice in the 1950s at the psychiatric department of the FU Berlin; according to his recollection, only patients with neurosyphilis were treated there with malaria fever. See also reference [9, p. 516].

8 Spiel W. mentioned in the same book that the availability of this strain in Vienna gave the possibility to apply malaria therapy for hebephrenic schizophrenia in children and adolescents.

9 For example: 26-year-old JH, diagnosed with schizophrenia, was “returned from Steinhof [to the clinic] as a malaria carrier [*Stammtäger*]” on April 30, 1952 (M1952.05.12, Archive of the University Clinic for Psychiatry and Psychotherapy at the Vienna General Hospital). According to JH’s medical records from Baumgartner Höhe (discharge 1953, WStLA, M. Abt. 209, Otto-Wagner-Spital, A 12/4), he was transferred “to the psychiatric clinic as a malaria carrier [*Stammtäger*]” and returned on May 12, 1952.

10 Viktor Klare reports on his own experiences with a material of 2140 cases over a period of 10 years.

11 Geiger K. Die Kinderstation. In: Heiss G. Die Malariatherapie und weitere diagnosekorrelierte Therapien. Ihre Anwendung an der Wiener Universitätsklinik für Psychiatrie und Neurologie in den 1950er und 1960er-Jahren und ihre Diskussion in der zeitgenössischen Forschung. Projektendbericht für den Jubiläumsfonds der Österreichischen Nationalbank 2015. p. 253–54. The children’s ward was part of the research project, but not of this publication or its supplement (see fn. 2).

12 Hippius H., in an email to Gernot Heiss dated August 12, 2014 (Josephinum Archive, Vienna; cited above in footnote 7), recalls that during his work in the 1950s at the psychiatric department of the Free University of Berlin, he was only aware of patients with neurosyphilis being treated with malaria-induced fever.

13 For the application of the term, refer to the case examples from patient files in Heiss, G. (cf. fn. 2), pp. 98 and 104.

14 With regard to these concepts, Kundratitz refers to the effects of this type of stress on the “autonomic nervous system and the endocrine system,” while Bleuler points to “adaptive reactions” such as “altered adrenal cortex functions.” For Bleuler, this generally applies to nonspecific “shock therapies,” including malaria fever therapy in non-syphilitic diseases.

15 Presumably, this was the objective of malaria fever therapy as a form of pre-treatment (“Vorbehandlung”) or preparatory measure (“Vorbereitung”) for alcohol withdrawal therapy at the Kalksburg facility. See the case studies in Heiss, G. (cf. fn. 2), pp. 147–148.

16 Hans Hoff to the Nobel Committee, Oct 19, 1957. Copy in the Josephinum Vienna, NL-Hoff, MUW-AS-006005-0011-068 Zl. 68/5.

17 Peter Berner, assistant at the ‘Hoff Clinic’ throughout the entire period of study and eventually Hoff’s successor in psychiatry, also expressed his doubts about the effectiveness of insulin coma therapy in a French survey in 1980 [20, p. 167].

18 See also Heiss G. (cf. fn. 2), pp. 204–05.

19 Hoff H. [Letter to the Nobel Committee]. 1952 Oct 11. Copy in: Josephinum Vienna, NL-Hoff MUW-AS-006005-0015.

20 On the use of psychotropic drugs at the Vienna clinic, see Heiss G. (cf. fn. 2), pp. 272–300.

21 Psychoanalysis and individual psychology were represented under Hoff through his collaborators Wilhelm Solms, Raoul Schindler, Walter Spiel, and Erwin Ringel.
