# Supplementary material for: In the shadow of the “psychopharmacological revolution”: Malaria fever, insulin coma, cardiazol and electroconvulsive therapy at the Vienna Psychiatric University Clinic, 1951–1969
Source: Wien Klin Wochenschr. 2025 Oct 2;138(3-4):63–70. doi: 10.1007/s00508-025-02592-w (PMC12904974; doi:10.1007/s00508-025-02592-w)
Supplement: Supplementary file 1 — The extensive German-language supplement presents a thoroughly revised and significantly expanded version of the unpublished 2015 project report. It includes bar charts and statistical tables based on the analysis of the database, detailed discussions of contemporary debates on ‘heroic therapies’ and the introduction and use of new psychotropic drugs, as well as numerous case studies drawn from patient records [file 508_2025_2592_MOESM1_ESM.pdf]

Gernot Heiss

Die Anwendung der Therapien mit Malariafieber, Insulinkoma, Elektro- und Cardiazolkrampf sowie mit den neuen Psychopharmaka an der Wiener Psychiatrisch-Neurologischen Universitätsklinik unter Hans Hoff (1951 – 1969) und ihre zeitgenössische Diskussion

Stark überarbeiteter und erweiterter Projektbericht

Die Arbeit basiert auf dem Forschungsprojekt „Die Malariatherapie und weitere diagnosekorrelierte Therapien: ihre Anwendung an der Wiener Universitätsklinik für Psychiatrie und Neurologie in den 1950er und 1960er Jahren und ihre Diskussion in der zeitgenössischen Forschung" (2013 – 2015), gefördert vom *Medizinisch-wissenschaftlichen Fonds des Bürgermeisters der Bundeshauptstadt Wien* (Bürgermeisterfonds Projekt 12062, Dr. Fliedl) und vom *Jubiläumsfonds der Österreichischen Nationalbank* (Jubiläumsfondsprojekt Nr. 15249).

Inhaltsverzeichnis

Vorwort ……………………………………………………………………………………… 5

1 Einleitung: Das Projekt und seine Quellen ………………………………………….….. 9
1.1 Abgrenzung des Forschungfeldes ……………………………………………………... 10
1.2 Die Quellen ……………………………………………………………………………. 11
1.2.1 Die PatientInnenakten ……………………………………………………………….. 12
1.2.2 Zeitgenössische Publikationen ………………………………………………………. 21
1.2.3 Zeitzeugen …………………………………………………………………………… 21
1.3 Hans Hoff und die Wiener Universitätsklinik für Psychiatrie und Neurologie unter seiner Leitung (1950 – 1969 ……………………………………………………………………… 22

2 Die „'großen' körperlichen Behandlungsverfahren" in der zeitgenössischen Literatur 27
Kreuztabellen zur Summe und Relation von Diagnosen und den ‚großen' körperlichen Therapien ………………………………………………………………………………… 34
Das Geschlecht – Einfluss auf Diagnose und Therapie? ……………………………… 41
Soziale Parameter ………………………………………………………………………. 43
A) Stichprobe aus 1955 – 1960 ……………………………………………………….. 43
B) Männerabteilung März bis September 1964 ……………………………………… 47

2.1 Die Malariafiebertherapie ……………………………………………………………. 50
2.1.1 Geschichte und Diskussion des Wirkmechanismus ……………………………… 50
2.1.2 Zur Technik der Anwendung der Malariafiebertherapie an der Klinik …………… 53
2.1.3 Zur vielfältigen Anwendung der Malariafiebertherapie an der Wiener Klinik: Vorgangsweise der Untersuchung und Auswertung ………………………………. 57
2.1.4 Die Malariafiebertherapie bei den einzelnen Diagnosen in ihrer Anwendung an der Klinik und in der wissenschaftlichen Diskussion der Zeit ………………………… 63
2.1.4.1 Die Diskussion über Malariafiebertherapie und / oder Penicillin bei neuroluetischen Erkrankungen ……………………………………………………………………. 64
2.1.4.2 Malariafiebertherapie bei nicht-luetischen Erkrankungen in der wissenschaftlichen Diskussion und Praxis ……………………………………………………………… 75
2.1.4.2.1 Die Anwendung der Malariafiebertherapie bei schizophrenen Erkrankungen 83

2.1.4.2.2 Über die Anwendung der Malariafiebertherapie in der Behandlung „zerebralgestörter Kinder“ 91
2.1.4.2.3 Die Malariafiebertherapie bei affektiven Störungen 100
2.1.4.2.4 Malariafiebertherapie bei der Diagnose Psychopathie 108
A) Die Praxis der Wiener Klinik zur Malariatherapie bei Psychopathie (statistische Daten und Fallbeispiele) 119
B) EEG als Nachweis von Reifungsstörungen bei der Diagnose Psychopathie 122
C) Das psychopathische Syndrom nach Encephalitiden 126
D) PatientInnen mit der Diagnose Psychopathie aus Erziehungsheimen 128
a) Weibliche Jugendliche aus Heimen mit der Diagonse Psychopathie 129
b) Jugendliche aus der Bundesanstalt für erziehungsbedürftige männliche Jugendliche in Kaiserebersdorf 131
c) Patienten aus dem Heim der Wiener Jugendfürsorge in Eggenburg 137
d) Zwischenresumee zu Malariafiebertherapie bei Patientinnen aus Heimen ... 141
2.1.4.2.5 Malariafiebertherapie bei den Diagnosen Alkoholismus Neurose, Verwahrlosung (insb. Alkoholismus) 145
2.1.4.3 Die Weitergabe des mit Malaria tertiana-Plasmodien infizierten luesfreien Blutes (zum Begriff der „StammträgerInnen“) und seiner Bedeutung in den PatientInnenakten 150
2.1.5 Zusammenfassung, Schlussbemerkungen und offene Fragen zur Anwendung der Malariafiebertherapie 161

2.2 Die Insulinkoma- und die Insulinsubkomatherapie 172
2.2.1 Geschichte und Diskussion der Insulinkomatherapie 172
2.2.2 Die Position der Wiener Kliniker in ihren Publikationen und ihre Praxis 188
2.2.2.1 Die Wirkweise der Insulinkomatherapie nach ihren Wiener Befürwortern 188
2.2.2.2 Die Durchführung der Insulinkomatherapie an der Wiener Klinik und die (allgemeine) Einschätzung der Risiken 193
2.2.2.3 Indikation und Erfolg lt. Wiener Publikationen 197
2.2.2.4 Integration der Insulinkomatherapie in den „Gesamtbehandlungsplan“ lt. Wiener Publiktationen 199
2.2.3 Die Insulinsubkomatherapie in Publikationen 206
2.2.4 Die Anwendung der Insulintherapie an der Wiener Psychiatrie nach den PatientInnenakten 210

2.2.4.1 Insulintherapie bei PatientInnen mit der Diagnose schizophrener Erkrankungen … 213
Exkurs: Fallbeispiele mit Lobotomie …………………………………………………… 222
2.2.4.2 Insulintherapie bei PatientInnen mit affektiven Störungen ………………………225
2.2.4.3 Insulintherapie bei Psychopathie ……………………………………………….. 232
2.2.5 Zusammenfassung zur Anwendung der beiden Insulintherapien …………………… 233
2.2.5.1 Die Insulinsubkoma- und die verkürzte Komatherapie (Zusammenfassung) ……... 233
2.2.5.2 Die Insulinkomatherapie (Zusammenfassung) …………………………………... 234

2.3 Die Elektrokrampftherapie und ihre Anwendung an der Klinik Hoff ………………… 238
2.3.1 Die Diskussion um die Elektrokrampfbehandlung in den 1950er Jahren …………… 239
2.3.2 Die Entwicklung von Methoden zur Verringerung der Risiken an der Wiener Klinik 242
2.3.2 Erklärung des Wirkmechanismus in den Publikationen der Zeit …………………… 248
2.3.3 Einsatzgebiete der Elektrokrampfbehandlung 1951-1969 nach den Publikationen der Zeit ……………………………………………………………………………… 250
2.3.4 Statistische Auswertung zur Elektrokrampftherapie (EKT) ………………………… 258
2.3.4.1 Häufigkeitsverteilung über die Jahre …………………………………………… 259
2.3.4.2 Alter und Aufenthaltsdauer ……………………………………………………….. 261
2.3.4.3 Diagnosen zur Elektrokrampftherapie ……………………………………………….. 262

2.4 Psychopharmaka an der Klinik Hoff ……………………………………………… 271
2.4.1 Neuroleptika ………………………………………………………………………….. 273
2.4.2 Antidepressiva ……………………………………………………………………….. 290
2.4.3 Tranquilizer / Sedativa / Anxiolytika ……………………………………………... 295
2.4.4 Schlussbemerkungen zu den Psychopharmaka …………………………………… 297

3 Zusammenfassung zum Projekt und seinen Ergebnissen ……………………………….. 299
3.1 Die Elektrokrampftherapie ……………………………………………………… 301
3.2 Die Insulinkomatherapie ………………………………………………………… 303
3.3 Die Malariafiebertherapie ……………………………………………………………... 305
3.4 Ergebnisse, offenen Fragen und Forschungsdesiderata ……………………………… 310

Quellenverzeichnis ……………………………………………………………………… 317
Verzeichnis der Medikamente (Wirkstoff und Handelsname) ………………………….. 342

Vorwort

2012 berichteten österreichische Medien, dass an der Wiener Psychiatrisch-Neurologischen Universitätsklinik unter der Leitung von Hans Hoff (1897–1969) in den 1960er Jahren PatientInnen missbräuchlich zu Forschungszwecken mit Malaria infiziert worden seien. Die Meldung zog ein breites öffentliches Interesse nach sich. Dazu trug bei, dass zum selben Zeitpunkt auch Gewalt und Mißbrauch in den Kinderheimen der Stadt Wien (2012) sowie im Kinderheim am Wilhelminenberg (2013) in eigenen Forschungsprojekten untersucht wurden. Darüber hinaus waren in den Jahren davor auch Ergebnisse zu den NS-Euthanasie-Verbrechen *Am Spiegelgrund* in Wien publiziert worden.[1]

Die medialen Berichte nahm das Rektorat der Medizinischen Universität zum Anlass, ein Projekt zur Wiener Psychiatrisch-Neurologischen Universitätsklinik unter der Leitung von Hans Hoff, der der Klinik von 1951 bis 1969 vorstand, als Grundlage für eine historisch kritische Einschätzung zu initiieren. Ich übernahm die Planung, die Einreichung zur Finanzierung und die Durchführung dieses Projekts;[2] das Rektorat der Medizinischen Universität richtete in Absprache mit mir und zu meiner Unterstützung eine *externe Histoirkerkommission* ein.[3] In Zusammenarbeit mit Dr. med. Rainer Fliedl reichte ich ein Pilotprojekt beim *Medizinisch-wissenschaftlichen Fonds des Bürgermeisters der Bundeshauptstadt Wien* (Bürgermeisterfonds) und als alleiniger Antragsteller ein Projekt beim *Jubiläumsfonds der Österreichischen Nationalbank* (Jubiläumsfonds) ein. Die Anträge sahen vor, im Rahmen von zwei Forschungsjahren zur Anwendung der Malariafiebertherapie und weiterer somatischer und medikamentöser Therapien die überlieferten PatientInnenakten der beiden psychiatrischen Erwachsenenabteilungen und der Kinderabteilung für die Jahre von

---

[1] Vgl. SIEDER/SMIOSKI/EICH/KIRSCHHOFER, Der Kindheit beraubt. Gewalt in den Erziehungsheimen der Stadt Wien (2012); HELIGE/JOHN/SCHMUCKER/WÖRGÖTTER/WISINGER, Endbericht der Kommission Wilhelminenberg (2013); GABRIEL/NEUGEBAUER (Hrsg.), Vorreiter der Vernichtung? Von der Zwangssterilisierung zur Ermordung. Zur Geschichte der NS-Euthanasie in Wien 2 (Wien 2002).

[2] Die Frage, ob für die Übernahme der Projektleitung darin ein Problem zu sehen sei, dass mein Bruder Wolf-Dieter noch in den letzten Jahren der *Ära Hoff* (ab 1966) seine Ausbildung an der neurologischen Abteilung der Klinik begann, wurde in den ersten Gesprächen mit dem Rektorat von mir zur Diskussion gestellt und nicht als hinderlich erachtet. Seine Bekanntheit in Fachkreisen dürfte mehrmals meinen Zugang zu ZeitzeugInnen erleichtert haben.

[3] Mitglieder der externen HistorikerInnenkommission in der Leitung von ao.Univ.Prof. i.R. Dr. Gernot Heiss: o.Univ.Prof. Dr. Mitchell Ash, ao.Univ.Prof. Dr. Margarete Grandner, Univ.Prof.Dr. Gabriella Hauch, Univ.Prof. DDr. Oliver Rathkolb; beigeordnete externe ExpertInnen: Dr. med. Elisabeth Brainin (bis 15.05.2015, vgl. unten S. 143 Anm. 532), Hon. Prof. Dr. Konrad Brustbauer, Univ.Prof. DDr. Christian Kopetzki; Beirat aus der Medizinischen Universität: Univ.Prof. Dr. Stephan Doering, o.Univ.Prof. Dr. Max Friedrich, Univ.Prof. Dr. Michael Hubenstorf, o.Univ.Prof. Dr. Siegfried Kasper, Univ.Prof. Dr. Johannes Wancata. Die Mitglieder, beigeordneten ExpertInnen und Beiräte haben in Kommissionssitzungen und auf Rückfragen das von Gernot Heiss in Eigenverantwortung geführte Projekt beraten.

1951 bis 1969 quantitativ und qualitativ auszuwerten sowie zur historischen Einordnung die Erwähnungen in Handbüchern und vor allem die Diskussion dieser Therapien in der Wiener und in der internationalen wissenschaftlichen Literatur zu untersuchen.

Ende 2012 wurden durch die beiden genannten Fonds die Mittel bewilligt, mit denen 2013 und 2014 insgesamt fünf MitarbeiterInnen angestellt werden konnten: MMag.Dr. Katharina Arnegger (Datenaufnahme und Diskussion der zeitgenössischen Literatur); Dr.med. Dave Bandke (medizinische Fachberatung, statistische Auswertung, Literaturrecherche, Diskussion der zeitgenössischen Literatur); Mag. Dr. Silke Fengler (Datenaufnahme und Diskussion der zeitgenössischen Literatur); Mag. Dr. Katja Geiger (Datenaufnahme und Auswertung der Akten und Literatur zur Kinderstation in einem eigenen Beitrag zum Endbericht des Projekts von 2015); Mag. Thomas Mayer (Literaturrecherche).

Anfang 2013 wurde die Datenbank für die Aufnahme der Informationen aus den PatientInnenakten entworfen, bei deren Erstellung wir von Dipl. Ing. Dr. Andreas Gleiß und von Univ.Prof. Mag. Dr. Michael Schemper (beide vom Institut für Klinische Biometrie der Medizinischen Universität Wien) beraten wurden.

Der Endbericht zum Projekt von 2015 zu den Erwachsenenabteilungen wurde von mir als Projektleiter erstellt, wobei vielfach Textentwürfe von ProjektmitarbeiterInnen sowie die direkt übernommenen statistischen Auswertungen von Dave Bandke als Grundlage dienten. Ein eigenes Kapitel zur von Walter Spiel geleiteten Kinderabteilung verfasste Katja Geiger. Eine gemeinsame Autorenschaft des Gesamtberichts war aufgrund von Auffassungsunterschieden bei der Interpretation der Quellenbefunde nicht möglich.

Aufgrund dieser Differenzen publizierte Katja Geiger den Abschnitt über die von ihr im Rahmen des Projekts bearbeitete Kinderstation (Krankenakten und Literatur) als von ihr gezeichneter Beitrag im nicht publizierten Projektendbericht von 2015. Dieser wird in der Bibliothek der Medizinischen Universität Wien und in der Fachbibliothek des Instituts für Zeitgeschichte nach der Publikation des vorliegenden Textes zur Benützung und Nachprüfbarkeit aufliegen.[4]

---

[4] HEISS, Malariatherapie und weitere diagnosekorrelierte Therapien. Ihre Anwendung an der Wiener Universitätsklinik für Psychiatrie und Neurologie in den 1950er und 1960er Jahren und ihre Diskussion in der zeitgenössischen Forschung (2015).

Der Projektendbericht an den Jubiläumsfonds der Österreichischen Nationalbank (2015) wurde in einer Pressekonferenz des Rektorats von mir präsentiert, wobei Univ. Prof. Dr. Johannes Wancata[5] es als Vertreter der Psychiatrie übernahm, sich im Namen der Klinik für die nicht auszuschließende nichttherapeutische Verwendung von PatientInnen als „Stammträger" des mit Malariaparasiten infizierten Blutes[6] öffentlich zu entschuldigen.

Für die vorliegende Fassung wurde der Projektendbericht umfassend überarbeitet, erweitert und in einigen Abschnitten auch korrigiert.

Bedanken möchte ich mich bei den beiden Fonds für ihre finanzielle Unterstützung des Projekts, beim *Medizinisch-wissenschaftlichen Fonds des Bürgermeisters der Bundeshauptstadt Wien* (Bürgermeisterfonds) für die Förderung des gemeinsam mit Rainer Fliedl eingereichten Pilotprojekts, sowie beim *Jubiläumsfonds der Österreichischen Nationalbank* (Jubiläumsfonds) für die Förderung des übergreifenden Gesamtprojekts. Das Projekt wurde durch das Rektorat der Medizinischen Universität, durch Rektor Wolfgang Schütz und Vizerektorin Christiane Druml angeregt, die die Arbeit auch begleitend vielfach unterstützt haben. Zur Einrichtung und Auswertung der Datenbank waren wir auf die Unterstützung durch Michael Schemper und Andreas Gleiß vom Institut für Klinische Biometrie der Medizinischen Universität Wien angewiesen. Bei historischen und bei medizinischen Fragen während der Projektarbeit waren Mitglieder, externe ExpertInnen und Beiräte der HistorikerInnen-Kommission in den Sitzungen der Kommission, aber auch bei direkten Anfragen, sehr hilfreich. Den Professoren der Universitätsklinik für Psychiatrie und Psychotherapie Siegfried Kasper und Johannes Wancata ist für ihr fachliche Beratung zu danken, aber auch für ihre Unterstützung der praktischen Arbeit des Projekts, wodurch die PatientInnenakten aus dem Archiv der Klinik mit Zustimmung der Leitung des Allgemeinen Krankenhauses im Medizinischen Dokumentationszentrum des AKH bearbeitet werden konnten. Dem Dokumentationszentrum, seinem Leiter Martin Jordan und seinem Team ist dafür zu danken, dass dem Projekt für die Bearbeitung der PatientInnenakten mit besten Rahmenbedingungen nicht nur ein Arbeitsraum zur Verfügung gestellt wurde, sondern die Akten der Jahrgänge in zwei jederzeit für die BearbeiterInnen zugänglichen Nebenräumen

[5] Leiter der Klinischen Abteilung für Sozialpsychiatrie der Universitätsklinik für Psychiatrie und Psychotherapie der Medizinischen Universität Wien.
[6] Vgl. dazu die Ausführungen auf S. 155-161.

gelagert blieben, sodass sie auch nach 2015 für die weitere Bearbeitung durch mich eingesehen werden konnten. Dem Institut für Zeitgeschichte der Universität Wien ist für die Aufnahme des Projekts am Institut zu danken. Besonders bedanken möchte ich mich bei Oliver Rathkolb, der mich von Anfang an (bereits bei der Einreichung des Projekts) beraten hat, beim Tropenmediziner Michael Ramharter für die Klärung meiner Fragen zur Malaria, und bei Eberhard Gabriel, der immer bereit war, mich bei der Klärung und Präzisierungen vieler der medizinischen Fragen zu beraten, aber auch mit historischen Informationen zu unterstützen, sowie bei Monika Ankele, die mir mit vielen Hinweisen zur Kürzung bzw. zur Ergänzung in der Redaktion dieses Textes half.

Wien, im Sommer 2025 Gernot Heiss

1 Einleitung: Das Projekt und seine Quellen

Im Untersuchungzeitraum 1950/51 bis 1969, den Jahren der Wiener Psychiatrisch-Neurologischen Universitätsklinik in der Leitung von Hans Hoff, fanden in der Psychiatrie allgemein die goßen Veränderungen in der Anwendung körperlicher Therapien statt, die in der Medizingeschichte als „pharmakologische Wende“ bezeichnet werden. Die allgemeine Entwicklung aufgrund der „therapeutischen Innovationen“ seit dem Ersten Weltkrieg wird von den AutorInnen der Geschichte der Züricher Psychiatrie 1870 – 1970[7] folgendermaßen charakterisiert: „Eine potenzielle Funktionsverlagerung der psychiatrischen Anstalt vom Mittel [als Ort der Verwahrung, der Hospitalisierung, GH] zum Ort therapeutischer Intervention begann sich erst abzuzeichnen, als in der Zwischenkriegszeit eine Reihe von Fieber- und Schockkuren hoffen ließen, dass organische und funktionale Psychosen kausal beeinflusst und die klassischen ‚Zwangsmassnahmen' abgelöst werden könnten. Diese somatischen Kuren […] leiteten eine Phase der therapeutischen Innovation ein, die – unterbrochen durch Phasen der Ernüchterung – bis zur ‚pharmakologischen Wende' in den 1950er und 60er Jahren dauerte.“ [8] Die Zeit vom Ersten Weltkrieg bis in die 1950er Jahre war therapeutisch von ‚Schocktherapien‘[9] und „Kuren“ geprägt, von „physische[n] Methoden, insbesondere Barbituratnarkosen und anderen Schlaf- und Dämmertherapien, Cardiazolschocks, Insulinkomata und Malariakuren“, sowie seit Ende der 1930er Jahre von der Elektrokrampftherapie und Ende der 1940er Jahre von gehirnchirurgischen Eingriffen.[10] In den beiden folgenden Jahrzehnten als Übergangsphase dominierte zunehmend „das pharmakologische Paradigma“:[11] Diese , großen alten‘ Kuren wurden sukzessive und weitgehend von den neuen medikamentösen Therapien abgelöst – von den Neuroleptika (ab 1952), Antidepressiva (ab 1958) und Tranquilizern (Benzodiazepine ab 1960). Epochenübergreifend bekamen und behielten seit Beginn des 20. Jahrhunderts die Psychotherapien und die Arbeitstherapien einen hohen Stellenwert, Letztere mit dem Ziel der

---

[7] MEIER/BERNET/DUBACH/GERMANN (Hrg), Zwang zur Ordnung. Psychiatrie im Kanton Zürich (2007). Diese Publikation basiert auf einem Forschungprojekt mit dem Forschungsbericht: TANNER/MEIER/HÜRLIMANN/BERNET, Zwangsmassnahmen in der Züricher Psychiatrie (Typoskript 2002).
[8] MEIER/BERNET/DUBACH/GERMANN (Hrsg.), Zwang zur Ordnung. Psychiatrie im Kanton Zürich (2007), S. 79f. Ausführlich wird in diesem Sammelband diese Periodisierung beschrieben durch TANNER, Ordnungsstörungen: Konjunkturen und Zäsuren in der Geschichte der Psychiatrie (2007), S. 281-285.
[9] Vgl. zur Bezeichnung als Schocktherapien unten S. 27 Anm. 76**.**
[10] Vgl. GERMANN, Ein Insulinzentrum auf dem Land (2013), S. 153f. Vgl. TANNER, Ordnungsstörungen: Konjunkturen und Zäsuren in der Geschichte der Psychiatrie (2007), S. 283.
[11] Ebd., S. 284.

„‘Sozialisierung‘“, einer „‘sozialen Heilung‘“ des „gestörten Selbst“ innerhalb der Anstalt,[12] in Vorbereitung einer sozialen und beruflichen Reintegration nach der Entlassung.

Die therapeutische Praxis der „Klinik Hoff“ und die Positionen der Wiener KlinikerInnen in der wissenschaftlichen Diskussion der körperlichen Therapien mit ihren Besonderheiten – dem sukzessiven Ersatz der Malariafiebertherapie als Therapie der progressiven Paralyse (PP) durch das Penicillin und dem teilweise oder völlige Ersatz der „Fieber- und Schocktherapien“ als „Erschütterungstherapien“ [13] (d. h. auch der Malariafiebertherapie in ihrer Anwendung ausserhalb der Neurolues) durch die neuen Psychopharmaka[14] – ist in den folgenden Ausführungen zu erörtern und in die Entwicklung der Psychiatrie in dieser Übergangszeit einzuordnen.

1.1 Abgrenzung des Forschungsfeldes

Ausgangspunkt des Forschungsprojekts war die Malariafiebertherapie, die in den 1910er Jahren in Wien als Therapie der progressiven Paralyse (PP) entwickelt[15] und 1927 mit dem Nobelpreis für Julius Wagner-Jauregg honoriert wurde.[16] Das Forschungsprojekt sollte (1) zum einen die Frage klären, ob es in der *Ära Hoff* eine missbräuchliche Anwendung dieser Therapie alleine zu Forschungszwecken gab, und (2) zum anderen, inwiefern die Anwendung der Malariafiebertherapie in den 1950er und 1960er Jahren – auch international – noch „state of the art“ war.

Diese Therapie wurde im Untersuchungszeitraum nicht nur in der Behandlung der progressiven Paralyse, sondern auch bei PatientInnen mit Diagnosen aus dem Bereich der vier Diagnosefelder „Intelligenzmängel“, schizophrene und affektive Erkrankungen sowie

---

[12] GERMANN, Arbeit als Medizin: Die „aktivere Krankenbehandlung“ 1930 – 1960 (2007), S. 217f. und passim zur Arbeitstherapie in der psychiatrischen Diskussion. In diesem Sammelband beschreibt TANNER, Ordnungsstörungen: Konjunkturen und Zäsuren in der Geschichte der Psychiatrie (2007), S. 277, den Zusammenhang von „Störungen im psychischen System einzelner Menschen mit den komplexen Funktionsanforderrungen der modernen Gesellschaft“, in der das „geordnete Selbst […] Resultat einer permanenten Selbstnormierung und Selbstbeherrschung“ ist.

[13] Nach Manfred Bleuler: Siehe unten S. 28.

[14] Die Handelsnamen und die entsprechenden Wirkstoffe sind in einem Verzeichnis nach dem Quellenverzeichnis angeführt.

[15] WAGNER-JAUREGG, Über die Einwirkung der Malaria auf die progressive Paralyse (1918/19), S. 117-138. In den „Bemerkungen“ zu diesem Wiederabdruck von 1936 schildert Wagner-Jauregg diese Publikation als zentral in der Geschichte der Therapie. Die Malariafiebertherapie basierte auf der Erfahrung, dass sich auch psychisch Krankheiten nach stark fiebrigen Erkrankungen besserten.

[16] Zur Einschätzung der Bedeutung der Malariatherapie als erste erfolgreiche somatische Therapie in der Psychiatrie, vgl. SHORTER, Geschichte der Psychiatrie (1999), S. 294.

„Psychopathie“ angewandt. Um die Behandlung durch die Malariafiebertherapie bei PatientInnen mit diesen Diagnosen in ihrem therapeutischen Umfeld beurteilen zu können, waren darüber hinaus die anderen bei diesen PatientInnen angewandten „großen“ somatischen Therapien einzubeziehen, die in der Zwischenkriegszeit in der Psychiatrie entwickelt worden waren (Insulinkoma-, Cardiazolkrampf- und Elektrokrampftherapie [EKT])[17], sowie die Anwendung der neuen medikamentösen Therapien. Daraus ergab sich das erweiterte Untersuchungsfeld mit der Frage nach der Position der Wiener Psychiatrie in der ‚pharmakologischen Wende‘.

Im Vordergrund standen dabei die Fragen, in welchem Ausmaß und mit welcher Zielsetzung die ‚großen‘ körperlichen Therapien von den ÄrztInnen angewandt wurden bzw. ob Behandlungen durchgeführt wurden, die methodisch und ethisch nicht dem damaligen Stand der Wissenschaft entsprochen haben. Welche Wirkung wurde den vier „großen“ körperlichen Therapien aus der Zwischenkriegszeit zugeschrieben. Wurden sie auch an anderen Kliniken mit gleichen oder ähnlichen Indikationen angewandt, wie wurden sie in Fachpublikationen diskutiert und wie die neuen Medikamente (Penicillin bei progressiver Paralyse, Neuroleptika und Antidepressiva als Ersatz bzw. Ergänzung der sog. „Schocktherapien“ – d.h. auch der Malariafiebertherapie bei Diagnosen außerhalb der Neurolues). Die Veränderungen in dieser Zeit des Übergangs sowohl in der Zahl der Anwendungen, als auch in ihren Zielsetzungen und in ihrer Anwendungstechnik waren zu ermitteln, ebenso die Einschätzung der neuen Psychopharmaka als Ersatz bzw. in Kombination.

1.2 Die Quellen

Aufgrund der Eingangsfragen zum therapeutischen Übergang wurde das Untersuchungsfeld in Bezug auf die Quellen, die Diagnosen und die Therapien präzisiert und eingeschränkt. Zur Ermittlung der therapeutischen Praxis und ihrer Begründung aus der Sicht der ÄrztInnen waren als Quellen des Forschungsprojekts die Krankenakten vorgesehen und, um diese Praxis in die regionale und internationale wissenschaftliche Diskussion zu stellen, die Auswertung

[17] Die Cardiazolkrampftherapie, sowie andere Fiebertherapien (z. B. Fieber, das mit Typhus-Vakzinen provoziert wurde) werden im Text nur in ihrer Anwendung im Zusammenhang mit den drei andern „großen“ Therapien erwähnt. Die Lobotomie, die ebenfalls in den 1930er Jahren parallel von António Egas Moniz und Mario Fiamberti entwickelt und bis Anfang der 1960er Jahre auch in Wien an der Chirurgie in Absprache mit der ‚Klinik Hoff‘-vorgenommen wurde, wurde im Projekt nicht systematisch, aber im Text ergänzend in ihrer Position im therapeutischen Konzept der Klinik (‚Gesamtbehandlungsplan‘) in einem Exkurs behandelt.

der einschlägigen Wiener medizinischen Zeitschriften sowie einiger der wichtigen deutsch-, englisch- und französischsprachigen Zeitschriften und Handbücher zur Psychiatrie. Weiters wurden einzelne Stellungnahmen von ÄrztInnen als ZeitzeugInnen in Gesprächen und Briefen als Quellen verwendet.[18]

1.2.1 Die PatientInnenakten

Ende des 20. Jahrhunderts konnte noch bedauert werden, dass von den PatientInnenakten in der Forschung zur Geschichte der Psychiatrie zu wenig Gebrauch gemacht wurde.[19] Seither hat sich das deutlich geändert, zuletzt besonders in den Forschungen zu den Psychopharmaka.[20]

In der Bewertung der PatientInnenakten als Quelle ist zu beachten, dass sie Eintragungen des ärztlichen und des Pflegepersonals enthalten, also deren Arbeit und Sicht dokumentieren, und nicht die Sicht der PatientInnen; auch die Eintragungen der Ärzte/innen zu den persönlichen Ausführungen und dem Verhalten des /der Patienten/in sind von der ärztlichen Suche nach dem Krankheitsbild geprägt und lassen deshalb allenfalls nur indirekte Schlüsse auf die Sicht der PatientInnen zu.

Ausgehend von der Frage nach der medizinischen Berechtigung der Anwendung der ‚großen, alten' Kuren aus damaliger Sicht geht es in der vorliegenden Untersuchung um die Anwendung der ‚großen, alten' Kuren an der Wiener Klinik, um ihre Häufigkeit, Kombination und Begründung, und um ihre Beurteilung in der zeitgenössischen wissenschaftlichen Diskussion im Verlauf dieser zwei Jahrzehnte des Übergangs zu den

---

[18] PflegerInnen, die im Untersuchungszeitraum an der Psychiatrie gearbeitet hatten, haben sich – trotz der medialen Präsenz des Projekts – nicht gemeldet. Ein Rechercheversuch blieb vergeblich. In der Kommission wurden Gespräche mit Betroffenen, die in den 1950er und 1960er Jahren als Jugendliche an der Klinik mit malariainfiziertem Blut geimpft worden waren und sich gemeldet hatten, diskutiert. Man entschied sich dagegen, da die Fragestellung des umfangreichen Projekts auf die Perspektive der Ärztinnen ausgerichtet war und für die Suche nach weiteren Betroffenen, die wissenschaftlich notwendig geworden wäre, und für diese Gespräche einer starke Erweiterung sowohl zeitlich als auch personell durch psychotherapeutisch geschulte MitarbeiterInnen notwendig geworden wäre.

[19] RADKAU, Zum historischen Quellenwert von Patientenakten. Erfahrungen aus Recherchen zur Geschichte der Nervosität (1997), S. 73f.

[20] Vgl. die Literaturangaben in BEYER et al., Wissenschaftliche Untersuchung der Praxis der Medikamentenversuche in schleswig-holsteinischen Einrichtungen der Behindertenhilfe sowie in den Erwachsenen-, Kinder- und Jugendpsychiatrien in den Jahren 1949 bis 1975 (Endbericht 2021), S. 20-26: https://www.bmas.de/SharedDocs/Downloads/DE/Soziales/SAH/Materialien-und-Unterlagen/abschlussbericht_medikamentenversuche_1949-1975_schleswig-holstein.pdf?__blob=publicationFile&v=1 (23.09.2023)

neuen medikamentösen Therapien. Mit dieser Ausrichtung auf die ärztliche Perspektive wurden die PatientInnenakten und die wissenschaftlichen Publikationen der Zeit als geeignete Quellen gewählt und ausgewertet. Freilich wurde damit die Einseitigkeit der Perpektive akzeptiert und die andere Seite, die Sicht „von unten", die in den letzten Jahrzehnten in einer großen Zahl von Publikationen fruchtbar wurde,[21] vernachlässigt.

Wie allgemein, so ist auch bei den Wiener Akten zu beachten, dass die Eintragungen institutionellen Bedingungen und Gepflogenheiten folgen. Die oft sehr kurzen und meistens oberflächlichen Anamnesen, aber auch die spärlichen folgenden Einträge der Wiener Ärzte/innen zeigen wohl vor allem ihre Überforderung aufgrund der Belastung der Klinik als Durchgangsstation für die meisten der aufgenommenen PatientInnen, die von hier nach einer kurzen Beobachtung an psychiatrische Krankenanstalten überwiesen wurden. Die verbleibenden PatientInnen waren Fälle, in denen die Therapien und Therapiekombinationen am besten oder alleine an der Klinik durchgeführt werden konnten und / oder – dem Forschungsauftrag der Universitätsklinik entsprechend – PatientInnen mit Diagnosen bzw. Therapien von aktuellem wissenschaftlichem Interesse. Die Therapien (mit Ausnahmen[22]) sind zwar in den fast immer eingelegten Fieberkurven dokumentiert, jedoch nur selten (und dann nur in kurzen Bemerkungen) findet sich in den Beilagen eine Begründung für ihre Anwendung; die dazu informativen, wenn auch in Wien sehr kurzen und lückenhaften Beschreibungen des Krankheitsverlaufs (*Decursus morbi et therapiae*) und die Kopien der Entlassungsbriefe sind nur in wenigen Fällen in der Beilage überliefert. Die Hinweise zu den Therapien in den Publikationen der Wiener Kliniker sind hier wichtige Ergänzungen.

a) Ihre Auswahl: Bestimmung des Untersuchungsfeldes

Zur Auswahl der PatientInnenakten aus den fünf Diagnosefeldern wurden die Angaben in der Diagnosezeile am Deckblatt der Akten gewählt. Das, obwohl klar war, dass abgesehen von der Diagnose progressive Paralyse mit ihrem Bezug zur Syphilis diese Angaben nicht immer einer systematischen Diagnostik folgten bzw. eilig gestellt[23] und häufig auch erst nach der

---

[21] Vgl. BRÜCKNER/RÖSKE/ROTZOLL/MÜLLER, Geschichte der Psychiatrie „von unten" (2019), S. 347-376.
[22] So fehlen – mit seltenen Ausnahmen in den 1950er Jahren – Einträge zur Psychotherapie: vgl. unten S. 17.
[23] REITER/GABRIEL, Diagnose „Psychopathie" und diagnostischer Prozeß bei Jugendlichen (1973), S. 121-124 und passim, gehen ausführlich auf das Problem ein und zeigen u.a., dass die Notwendigkeit, die sich aus der organisatorischen Position der Klinik als Clearingstelle ergab, eine rasche Diagnoseerstellung ohne längere Beobachtung notwendig machte und häufig das Interesse an einer raschen Weiterleitung der Patientinnen

Behandlung und kurz vor der Ablage des Krankenakts eingetragen wurden.[24] Außerdem richten sich die Diagnosen, wie die Anamnesen nach sich wandelnden Krankheitsbildern richten. Es ist auch zu unterstreichen, dass der Untersuchungszeitraum überwiegend vor der Einigung auf die Verwendung international vereinbarter Diagnoseschemata (ICD) liegt, was wohl häufig zu Ungenauigkeiten führte. Bei der Auswahl nach dem Diagnoseeintrag vorzugehen, erschien aber dennoch als zielführend, da sich bei einer ersten Sichtung der Akten zur Malariafiebertherapie gezeigt hatte, dass lt. Diagnosezeile PatientInnen mit einer Malariafiebertherapie fast ausschließlich Diagnosen aus den folgenden fünf Diagnosefeldern hatten: Neurolues, Intelligenzmängel, Psychopathie, schizophrene und affektive Erkrankungen. Und dass diese Diagnosen auch Hauptanwendungsgebiet der körperlichen Therapien waren, die in dieser Studie interessieren: Malariafieber-, Insulinkoma-, Cardiazol- und Elektrokrampftherapie und ihrer Kombination mit bzw. Ablöse durch die neuen medikamentösen Therapien, durch Penicillin bzw. die neuen Psychopharmaka.

Da die kurmäßige Anwendung von körperlichen Therapien, d. h. die Anwendung im Rahmen einer längerfristigen Behandlung an der Klinik Thema dieser Untersuchung ist, kamen nur PatientInnen mit einem längeren stationären Aufenthalt in Frage. Dieser wurde pragmatisch mit mindestens fünf Tagen festgelegt. Durch dieses Auswahlkriterium wurden jene PatientInnen nicht aufgenommen, die nach einer kurzen Beobachtung entlassen, nur auf eine nichtstationäre Therapie eingestellt oder (oft auch aus Platzmangel) an eine andere psychiatrische Anstalt überwiesen wurden. Bereits vor der ‚Ära Hoff' hatte die Klinik die Funktion einer Clearingstelle mit der Aufgabe, die aus Wien und Umgebung (polizeiärztlich) zuerst an die Klinik eingewiesenen psychiatrischen PatientInnen aufzunehmen und die Erstbeurteilung und Verteilung vorzunehmen. Nur ein kleiner Prozentsatz wurde an der Klinik weiterbehandelt. Die meisten PatientInnen mit Wohnsitz in Wien, dem östlichen Niederösterreich und dem nördlichen Burgenland wurden an die *Heil- und Pflegeanstalt Steinhof* (ab 1963 *Psychiatrisches Krankenhaus der Stadt Wien Baumgartner Höhe / PKB*, in den Akten und im folgenden Text meistens nur nach der gebräuchlichen Bezeichnung *Steinhof* mit der der Lage am Hügel entsprechenden Präposition ‚auf den' oder ‚am'), ein Teil dann von dort oder auch direkt in die *Heil- und Pflegeanstalt* (ab Mitte der 1960er Jahre:

---

entscheidend war (sie zeigen dies am Beispiel der Diagnose „Psychopathie" in den Krankenakten jugendlicher Frauen in den Jahren 1958 und 1959).

[24] Vgl. unten S. 71 Anm. 230 zum Patienten N82 mit dem Diagnoseeintrag „Paranoid-halluzinatorisches Bild bei P.P."

Psychiatrisches Krankenhaus) der Stadt Wien in Ybbs überwiesen. Ein kleinerer Teil der PatientInnen, jene die in Niederösterreich gemeldet waren, wurden nach Gugging oder Mauer-Öhling überwiesen.[25]

Die PatientInnenakten aus den Jahren 1951 bis 1969, die im Archiv der Universitätsklinik für Psychiatrie und Psychotherapie des Allgemeinen Krankenhauses (AKH) gelagert sind und im Medizinischen Dokumentationszentrum des AKH[26] im Rahmen des Projekts unter dankenswert guten Bedingungen ausgewertet werden konnten, sind sehr umfangreich erhalten: Für die beiden Erwachsenenabteilungen der Psychiatrie sind die in den Jahrgängen 1951 bis 1969 abgelegten Akten auf 90.000 bis 100.000 zu schätzen. Aus arbeitstechnischen Gründen beschränkte sich das auf zwei Jahre und mit vier bis fünf MitarbeiterInnen geplante Projekt auf diese Akten, obwohl dadurch die Akten von PatientInnen fehlen, die zwar in der ‚Ära Hoff' an der Klinik behandelt, aber nach 1969 neuerlich aufgenommen und unter dem Entlassungsdatum ihrer letzten Aufnahme archiviert wurden.[27] Aus diesen unberücksichtigten Akten und aus Aktenverlusten (wohl vor allem bei den Umsiedlungen 1974[28]) ergibt sich eine Fehlstelle, die etwas mehr als 20% der PatientInnenakte der Erwachsenenstationen betragen dürfte. Katja Geiger, die im Rahmen des Projekts die rund 2.400 übelieferten Akten der Kinderstation sichtete, verglich in Stichproben die auf den Archivschachteln vermerkten Aktenzahlen mit dem Inhalt und schätzt den Aktenverlust auf ungefähr ein Drittel.[29] Das wird auch aus der Differenz zur Angabe im 10-Jahresbericht der Station ersichtlich,[30] wonach in den Jahren 1951 bis 1960 1.811 Kinder stationär behandelt wurden, während für diesen Zeitraum nur rund 1.300 Krankenakten überliefert sind. Beide Angaben liegen im Rahmen dessen, was auch in anderen Publikationen zum „natürlicher Aktenschwund" bei Krankenakten angegeben wird.[31] Ergänzende Quellen wie Verwaltungsakten wurden nicht gefunden und dürften skartiert worden sein. Dennoch wird in dieser nicht personenbezogenen,

---

[25] Dazu und zur Geschichte dieser Zusammenarbeit, die bis ins Ende des 19. Jahrhunderts zurückreicht und erst 1974 endete, vgl. GABRIEL, 100 Jahre Gesundheitsstandort Baumgartner Höhe (2007), S. 23f. Zur Klinik als „Durchgangsstation" Vgl. unten S. 20.
[26] Dem Leiter Martin Jordan und seinem Team ist für die gastfreundliche Aufnahme herzlich zu danken.
[27] Vgl. dazu z. B. unten S. 57f. und S. 151 die Ausführung zu 127 ‚StammträgerInnen', die im Akt des Empfängers ihres malariainfizierten Blutes genannt, deren Krankenakten aber nicht im Untersuchungszeitraum abgelegt sind.
[28] Vgl. SPIEL/MUTSCHLECHNER/SCHAUFLER/STÜTZ, Die Entstehung des Fachgebietes Kinder- und Jugendneuropsychiatrie (1994), S. 37: Spiel schreibt, dass bei dem chaotischen Umzug Krankengeschichten auf Mülldeponien gefunden wurden.
[29] Vgl. GEIGER, Kinderstation (2015), S. 216.
[30] N. N., 10-Jahresbericht der Psychiatrisch-Neurologischen Kinderstation (1962), S. 216.
[31] Vgl. BALZ, Zwischen Wirkung und Erfahrung – eine Geschichte der Psychopharmaka (2010), S. 159.

historischen Untersuchung davon ausgegangen, dass die aus diesem umfangreichen Quellenmaterial quantitativ und qualitativ gezogenen Annahmen und Schlussfolgerungen durch diese Fehlstellen nicht wesentlich verfälscht wurden.

Die Akten bestehen in jedem Fall einer Patientenaufnahme aus einem gefalteten Din A3 Doppelbogen, der als Aktenumschlag verwendet wurde. Auf dem Deckblatt finden sich außen Name, Adresse, Beruf, Art der Aufnahme (polizeiliche Einweisung oder freiwillig), Datum der Aufnahme und der Entlassung bzw. der Überweisung, Ziel der Überweisung („Steinhof" bzw. „M" für „monte", „Gugging" etc.), Diagnose, manchmal Name des / der behandelnden Arztes / Ärztin an der Klinik – in dafür durch Aufdrucke zur Erhebung der Personalien vorgegebenen Stellen. Auch findet sich hier, falls der / die PatientIn mit Malariafieber-, Insulinkoma oder Elektrokrampftherapie behandelt werden sollte, meistens ein Stempel oder eine handschriftliche Notiz, dass der „Revers", d. h. die Zustimmung zur Therapie vorhanden sei. Nicht selten sind unten auf dieser ersten Seite des Umschlagblattes Bemerkungen des Begleiters / der Begleiterin („Deponenten") des Patienten oder des Arztes bei der Aufnahme notiert. – Auf der Innenseite des Deckblattes (Seite 2) ist die maschinschriftliche Anamnese[32] des Arztes / der Ärztin (namentlich genannt und datiert) aufgrund eines Gesprächs mit dem Patienten / der Patientin eingetragen, die auch häufig auf der zweiten Innenseite des Doppelblattes fortgesetzt wurde. Es sind Hinweise zur Biographie, zu früheren Krankheiten und zum Eindruck, den der Arzt / die Ärztin vom Patienten / von der Patientin bei diesem Gespräch hatte (auf Symptome bezogen). – Die Rückseite dieses gefalteten DinA3 Umschlag-Bogens (4. Seite) ist oft leer, mehrmals sind auf ihr auch sehr kurze Hinweise zur Entlassung u. ä. eingetragen.

Für die Auswertung haben die genannten Hinweise auf dem Umschlag-Bogen zentrale Bedeutung. Die zweite wichtige Information bieten die Hinweise auf den 7-Tage-Bögen der Fieberkurven, die dem Akt beiliegen. Hier sind die Medikamente vermerkt und vor allem auch die im Forschungsprojekt zentralen Therapien Malariafieber-, Elektrokrampf-, Insulinkoma-, Cardiazolkrampftherapie, oder andere Fiebertherapien (vor allem mit Typhus Vakzinen) in ihren Verläufen. Es wurde hier auch kurz (ATh, ohne Spezifizierung) vermerkt, falls der Patient/ die Patientin zur Arbeitstherapie ging, sehr selten und weniger konsequent,

[32] Zu den Krankengeschichten und ihrer Abhängigkeit vom Zeitdruck und anderen „'institutionelle[n]' Faktoren" vgl.: REITER/GABRIEL, Diagnose „Psychopathie" und diagnostischer Prozeß bei Jugendlichen (1973), S. 122f.

wenn er / sie zu einer Gruppentherapie und leider gar nicht, wenn er / sie zu einer Einzeltherapie ging. Psychotherapie ist in einigen Fällen durch andere Beilagen im Akt nachzuweisen – manchmal durch Einträge zum *decursus therapiae* und selten auch durch Erwähnungen in Entlassungsbriefen oder in der Anamnese der nächsten Aufnahme.

Weitere Beilagen sind sehr unterschiedlich in den Akten überliefert: EEG- und Blutbefunde, psychologische Befunde, die internistische Freigabe für Therapien u. ä., sowie leider nur selten die bereits genannten Aufzeichnungen über den Verlauf der Therapien und manchmal die Kopien der Entlassungsbriefe. Die Beilagen sind also – abgesehen von den Fieberkurven – in den Akten der beiden Erwachsenenstationen nur fallweise und unregelmäßig vorhanden und aus ihnen ergeben sich meistens nur indirekte Hinweise zur Indikationsentscheidung.[33] Grund für die lückenhafte Information, vor allem auch für die meistens wenig ausführlichen Anamnesen, dürfte die starke Überlastung der beiden Erwachsenenabteilungen durch die Funktion der Wiener Klinik als ‚Clearingstelle' zur Erstbegutachtung und Verteilung der psychiatrischen PatientInnen auf die anderen psychiatrischen Krankenhäuser gewesen sein. In den Besprechungen der Therapien werden Fallbeispiele zitiert, die freilich die Problematik dieser schlechten Dokumentation nicht ausgleichen, aber doch einige Aspekte der Behandlung verdeutlichen können. Der Fokus liegt auf den beiden ‚Wiener Therapien', der Malariafiebertherapie und der Insulinkomatherapie, deren Besprechung deshalb auch umfassender ausfiel, als jene der Elektrokrampftherapie und der neuen Psychopharmaka.

Die Krankenakten der Kinderstation scheinen sorgfältiger, jedenfalls geordneter geführt worden zu sein. Ihre Aufnahme war nicht als ‚Clearingstelle' überlastet, was wohl dazu beitrug. Bei den Akten der Kinderstation haben die Angaben der Begleitpersonen, wie überhaupt die Angaben zu den Eltern und Geschwistern bzw. zur Heimkarriere den Stellenwert der Anamnese aufgrund des Arzt-Patienten-Gesprächs und diese füllen die Innenseiten: Neben Alter und Beruf der Eltern und Großeltern werden Erkrankungen, besonders ihre psychiatrischen Erkrankungen angegeben, ebenso Name, Alter und Erkrankungen der Geschwister. Die Entwicklungsdaten des Kindes, d. h. Angaben zum Verlauf der Schwangerschaft und der Geburt, zu Regelabweichungen in der Entwicklung des Kindes, zu Kindergarten- und Schulbesuch, zu Vorerkrankungen und Impfungen wurden

[33] Anders als im Vergleich etwa zu den Krankenakten der Psychiatrischen Universitätsklinik Heidelberg, wie Viola Balz sie beschreibt: BALZ, Zwischen Wirkung und Erfahrung – eine Geschichte der Psychopharmaka (2010), S. 159f.

erfragt. Die körperliche Verfassung des Kindes und die aktuelle Krankheit und ihr Erscheinungsbild wurden beschrieben. Ihre Auswertung wird im Folgenden zur Ergänzung und zum Vergleich mit den Erwachsenenstationen (mit den PatientInnen ab dem Alter von 14 Jahren) übernommen und findet sich ausführlich im Projekt-Endbericht von 2015.[34]

b) Die Datenbank zu den PatientInnenakten

Zur Auswertung der PatientInnenakten der Jahre 1951 bis 1969 wurden zwei Datenbanken eingerichtet: für die Akten der beiden Erwachsenenstationen eine SPSS-Datenbank; für die Akten der Kinderstation eine Excel Datei.[35] Während in die Excel Datei alle überlieferten Akten der Kinderstation aufgenommen wurden, wurden von den PatientInnenakten der beiden Erwachsenenstationen nur alle Akten zu jenen Fällen erfasst, die am Deckblatt in der Diagnosezeile eine Diagnose aus den fünf Diagnosefeldern und einen stationären Aufenthalt von mindestens fünf Tagen hatten.[36] Von PatientInnen mit mehreren Aufenthalten wurden alle den beiden Auswahlkriterien entsprechenden Fälle aufgenommen; mit Bezug darauf wird in der statistischen Auswertung von Fällen gesprochen. Nur zur Malariafiebertherapie wurden auch alle Fälle aufgenommen, die den beiden Auswahlkriterien nicht entsprachen: Das sind die Fälle, bei denen nur eine Diagnose eingetragen war, die nicht den Einschlusskriterien entsprach, in denen der / die PatientIn aber eine Malariakur erhielt,[37] sowie die sehr seltenen Fälle, in denen der / die PatientIn an der Klinik mit malariainfiziertem Blut geimpft und vor dem fünften Tag des Aufenthalts an ein anderes psychiatrisches Krankenhaus überstellt wurde.[38] Während die Anwendung der „‘großen‘ alten Kuren“,[39] die aus den Fieberkurven im Krankenakt recht gut und deutlich ersichtlich ist, systematisch in der Datenbank für die statistische Auswertung aufgenommen wurden, wurde der Einsatz der neuen Psychopharmaka

[34] GEIGER, Kinderstation (2015), S. 218.
[35] Die beiden Datenbanken sind im Josephinum (Wien) für weitere wissenschaftliche Forschung archiviert, mit dem Hinweis, wie die zur Anonymisierung der PatientInnen verwendeten Kennzahlen aufgeschlüsselt werden können, um im Archiv den Krankenakt zu finden. Sie wurden bereits für die Malariaforschung von zwei DiplomandInnen der MedUni Wien in der Betreuung von Michael Ramharter verwendet und mit deren Ergebnissen weiter in einer Publikation, die noch im Sommer 2025 im American Journal of Ttropical Medicine and Hygiene (AJTMH) eingereicht wurde: siehe unten S. 316 Anm. 1209 und 1210.
[36] Zu diesen beiden Auswahlkriterien vgl. S. 13-15.
[37] Vgl. unten den Abschnitt zur Malariafiebertherapie bei Diagnosen, die nicht den Einschlusskriterien des Projekts entsprechen (insb. Alkoholismus) S. 145-150.
[38] In der Datenbank sind einige Fälle von PatientInnen – immer mit der Diagnose progressive Paralyse (PP) – aufgenommen, in denen die PatientInnen nach der Impfung und vor den Fieberschüben zur Weiterbehandlung auf den *Steinhof* oder nach Gugging überwiesen wurden, einige davon auch nach weniger als 5 Tagen Aufenthalt, weshalb sie ohne Malariatherapie nicht in die Datenbank aufgenommen worden wären.
[39] Die Cardiazolkrampftherapie wurde in Zusammenhang mit der Tab. 1 und mit der Insulinkomatherapie bei schizophrenen Erkrankungen beschrieben, da sie lt. überlieferten Akten in diesen beiden Jahrzehnten mit 2 Ausnahmen nur im Insulinkoma „aufgesetzt“ wurde; vgl. unten S. 38-41, S. 39 Anm. 118.

in Wien aus pragmatischen Gründen[40] nur in Fallbeispielen und aus den Publikationen der Wiener KlinikerInnen beschrieben.

Eingetragen wurden zur quantitativen, aber auch zur qualitativen Auswertung folgende Angaben aus den Akten: Eine Kennzahl, die sich aus W oder M für das Geschlecht des / der Patienten/in und dem letzten Entlassungsdatum zusammensetzt, und mit der der Akt leicht gefunden werden kann (diese Kennzahl wurde in den Publikation weiter verschlüsselt). Weiters wurden die Daten der Aufnahme und der Entlassung im betreffenden Fall und die Zahl der Aufenthaltstage, sowie Alter und Geburtsjahr des / der Patienten/in eingetragen. Die Diagnosen wurden ausgeschrieben und für die statistische Auswertung aufgegliedert – nicht nur einzeln, sondern nochmals für jene Fälle, in denen die Diagnose differenziert angegeben wurde. Gleichermaßen wurden die Therapieeinträge zu den vier „‘großen‘ alten Kuren“ und zusätzlich zur Fieberkur mit Typhusvaccinen und zur Penicillintherapie ausgeschrieben und für die statistische Auswertung aufgegliedert. Weitere Einträge betreffen die Einweisung bzw. Entlassung, den Beruf, den einweisenden Arzt, die Station wenn es sich um die Privatstation B22 handelte, die Herkunft aus einem Jugendheim, manchmal medizinische und biographische Einträge zum Patienten / zur Patientin, den Wohnbezirk, die Namensabkürzung (erster Buchstabe des Vor- und des Nachnamens[41]) sowie besondere Bemerkungen zum Therapieverlauf, wie etwa bei der Malariatherapie das Datum der Impfung und das Namenskürzel des Spenders / der Spenderin.

In der SPSS-Datenbank zu den beiden Erwachsenenstationen wurden nach den Einschlusskriterien für die gesamte Periode von 1951 bis 1969 14.919 Fälle aufgenommen, wobei es sich wegen der 3.199 Mehrfachaufnahmen um insgesamt 11.720 PatientInnen der beiden Erwachsenenabteilungen handelt. Um den Anteil der PatientInnen, die den beiden Einschlusskriterien entsprechen, an den gesamten Aufnahmen in etwa einschätzen zu können, wurden für den Oktober 1955 die Grunddaten mit Diagnose und Aufenthaltsdauer von allen erhoben und ausgewertet, die in einer der Erwachsenenabteilungen aufgenommen wurden. Es waren 389 PatientInnen[42], von denen 17 % das Aufenthalts- und das Diagnosekriterium erfüllt

[40] Vgl. unten im Kapitel zu den Psychopharmaka S. 271-299.
[41] Diese ist bei Patientinnen besonders wichtig um den Akt zu finden, da sie im Unterschied zu den Patienten im Monat ihrer letzten Entlassung nicht chronologisch, sondern alphabetisch eingeordnet sind.
[42] Es waren 207 Patienten und 182 Patientinnen.

haben[43]: In den ersten vier Tagen waren ca. 2/3 entlassen oder – je nach Wohnort – in ein psychiatrisches Krankenhaus (vor allem ‚auf den *Steinhof*', aber auch nach Gugging und Mauer-Öhling) überstellt worden, und vom restlichen, an der Klinik bleibenden Drittel hatten ca. die Hälfte eine der 5 Diagnosen. Diese Praxis und diese Zahlenverhältnisse werden auch in zeitgenössischen Publikationen bestätigt. So schrieb 1959 der Erste Assistent und Stellvertreter des Klinikleiters Ottokar H. Arnold: „die Wiener Klinik mit ihren 223 psychiatrischen Betten [ist] natürlich nicht in der Lage […], die große Anzahl der Aufnahmen auch hier zu behandeln. Vielmehr stellt sie für die Mehrzahl der Aufnahmen ausschließlich eine Durchgangsstation dar und verteilt diese Aufnahmen an 3 psychiatrische Anstalten in der näheren und weiteren Umgebung Wiens. Es können nur jene Fälle behandelt werden, die aus Forschungsgründen oder zum Zwecke des Unterrichtes für die Klinik wichtig sind, oder die einer nur an der Klinik durchführbaren Spezialuntersuchung oder -behandlung bedürfen."[44] Und noch 1968 wird in einer Studie berichtet, dass „nur ein relativ kleiner Teil, etwa ein Sechstel der Gesamtaufnahmen, […] einer Langzeitbehandlung an der Universitätsklinik selbst zugeführt [wird], während die übrigen Patienten nach etwa 1 – 3 tägigem Aufenthalt entsprechenden Anstalten zur weiteren Therapie übergeben werden."[45] Der Anteil der rasch weiter geleiteten PatientInnen hatte sich demnach sogar deutlich erhöht.

Bei Mehrfachaufnahmen, die in den beiden Erwachsenenstationen im Mustermonat Oktober 1955 19,6% der Fälle, also beinahe 20% der Fälle betrafen, wurden alle jene Aufnahmen der Patientin / des Patienten in der Datenbank eingetragen, die den beiden Einschlusskriterien (eine der 5 Diagnosen, Aufenthaltsdauer mindestens 5 Tage) entsprachen (die Kennzahl blieb gleich). Wie bereits angesprochen, fehlen aufgrund der Archivierung unter dem Entlassungsdatum der letzten Aufnahme des Patienten / der Patientin bei mehrfachen Aufnahmen Fälle aus dem Untersuchungszeitraum in der Datenbank, die mit einer Aufnahme nach 1969 archiviert wurden.[46]

Im Projekt war vorgesehen, in einem ersten Schritt als „Pilotstudie" die PatientInnenakten der Erwachsenenstation der Jahre 1955 bis 1960 aufzunehmen und in einer repräsentativen

---

[43] Von diesen ca. 17% ausgehend, die auch in der Literatur als Annäherungswert bestätigt werden, ergeben die in der Untersuchung für die Zeit vom 1. Jänner 1951 bis 31. Dezember 1969 nach den Einschlusskriterien (Diagnosen und Aufenthaltsdauer) aufgenommenen 14.919 Fälle hochgerechnet 87.758,8 Aufnahmen.
[44] ARNOLD, Klinische Erfahrungen mit dem Neuroleptikum Truxal (1959), S. 892-898.
[45] KÜFFERLE/SCHULTES, Übersicht über die an der Wiener Psychiatrischen Klinik im Jahr 1967 zur Sedierung verwendeten Medikamente (1969), S. 71-75.
[46] Vgl. oben S. 15.

Stichprobe auszuwerten. Dazu wurden aus den für diese sechs Jahre 5.140 aufgenommenen Fällen 525 Fälle gezogen. Bei der Erstellung der Stichprobe wurden wir – wie bei der Gliederung der Datenbank – von Dr. Gleiß und von Prof. Schemper vom Institut für Klinische Biometrie der Medizinischen Universität Wien beraten. Zu diesen 525 Fällen der Stichprobe wurden Informationen ergänzt und systematisiert, so der Beruf (Hilfsarbeiter und Angestellte) oder die Aufnahme in die Privatstation als soziale Indikatoren, sowie medikamentöse Therapien; Ergebnisse der Auswertung der Stichprobe aus diesen sechs Kernjahren sind in den vorliegenden Text integriert.[47]

Für den Endbericht des Projekt von 2015 wurden die gesamten Daten der Erwachsenenstation ohne Stichprobe ausgewertet und die Ergebnisse dieser Auswertung wurden in der vorliegenden Publikation auf der Basis dieses Projektendberichts übernommen. Die Entscheidung gegen eine Stichprobe für den gesamten Zeitraum erfolgte, da der Aufwand für wenig ertragreich eingeschätzt wurde. Für die wichtige Frage nach einer sozialen Indikation blieb die Auswertung der Stichprobe zu 1955/1960 durchaus aufschlussreich.

1.2.2 Zeitgenössische Publikationen

Zur Frage der Einordnung der Wiener Praxis in die zeitgenössische wissenschaftliche Diskussion wurde mit Unterstützung der Bibliothek der Medizinischen Universität nicht nur in den Wiener Zeitschriften (*Wiener Zeitschrift für Nervenheilkunde und deren Grenzgebiete*; *Wiener klinische Wochenschrift*; *Wiener medizinische Wochenschrift*; *Wiener Archiv für Psychologie, Psychiatrie und Neurologie*) sondern auch in wichtigen deutsch-, englisch- und französischsprachigen Zeitschriften (*Der Nervenarzt*; *Münchner medizinische Wochenschrift*; *Schweizerische medizinische Wochenschrift*; *The Lancet*; *Journal of the American Medical Association*) und Lehrbüchern recherchiert.

1.2.3 Zeitzeugen

Informationen wurden auch aus einzelnen Gesprächen, Telefonaten und e-mail-Kontakten mit ÄrztInnen bezogen, die in den 1950er und 1960er Jahren an der Wiener Psychiatrie bzw. an anderen psychiatrischen Kliniken tätig waren.

---

[47] Vgl. S. 43-47.

1.3 Hans Hoff und die Wiener Universitätsklinik für Psychiatrie und Neurologie unter seiner Leitung (1950 – 1969)

Die 19 Jahre der Wiener Psychiatrisch-Neurologischen Universitätsklinik von Herbst 1950 bis 1969 waren geprägt durch ihren Leiter Hans Hoff (1897-1969). Deshalb wird im Folgenden auf seine Karriere und wissenschaftliche Position, auf die Entwicklung der Klinik in seiner Leitung und die Einschätzung von Hoffs Tätigkeit durch einzelne seiner Mitarbeiter eingegangen.

Hoff hatte 1938 vor der rassistischen Verfolgung der Nazis fliehen müssen[48] und war einer der wenigen Universitätslehrer, die nach Österreich zurückkehrten.[49] Nach dem Ende seiner Verpflichtungen in New York übernahm er 1950 die Leitung des Neurologischen Instituts der Universität Wien („Obersteiner-Institut") sowie die Leitung einer für ihn geschaffenen zweiten Abteilung in der Nervenheilanstalt Rosenhügel. Im selben Jahr wurde er von der medizinischen Fakultät in der Nachfolge von Otto Kauders (1893-1949)[50] an der Klinik *primo et unico loco* vorgeschlagen und berufen, behielt aber bis 1959 weiterhin die Leitung des Neurologischen Instituts.[51]

Zur Entscheidung der Fakultät dürften die von Hoff seit 1946 aktivierten Netzwerke aus der Zeit vor 1938 und die Absicht geführt haben, die Tradition der Wiener Psychiatrie fortzusetzen, die in der Zwischenkriegszeit vor allem durch die Entwicklung der beiden „großen" körperlichen Therapien – der Malariatherapie gegen die progressive Paralyse sowie der Insulinkomatherapie bei Schizophrenie – Weltgeltung besaß. Wie sein Vorgänger Otto Kauders kam Hans Hoff aus der Wiener Psychiatrie der Zwischenkriegszeit und beide

---

[48] Vgl. ARIAS, Hans Hoff (1897-1969) – Remigrant und Reformer? (2016), passim**.** Zu den personellen Auswirkungen der NS-Politik an der Klinik, vgl. den Abschnitt zu den Dozenten bis 1945, vgl. GABRIEL, Zum Wiederaufbau des akademischen Lehrkörpers in der Psychiatrie in Wien nach 1945 (2016), S. 49-55.

[49] Vgl. zu den Problemen der Wiener Universität nach 1945 den Sammelband hgg. von GRANDNER/HEISS/RATHKOLB, Zukunft mit Altlasten. Die Universität Wien 1945 bis 1955 (2005), mit Beiträgen, die die Entnazifizierung, sowie die personellen und ökonomischen Probleme der Universität beschreiben. Zur medizinischen Fakultät vgl. in diesem Sammelband ARIAS, Die medizinische Fakultät von 1945 bis 1955 (2005), S. 68-88 und die Wiener phil. Diss. von ARIAS, Die Wiener Medizinische Fakultät von 1945 bis 1955. Entnazifizierung, Personalpolitik und Wissenschaftsentwicklung (2013). Vgl. auch GABRIEL, Die Orientierung(en) der österreichischen Psychiatrie 1945 bis Mitte der 50er Jahre (2015), passim, und GABRIEL, Zum Wiederaufbau des akademischen Lehrkörpers in der Psychiatrie in Wien nach 1945 (2016), passim.

[50] Er hatte 1938 die Professur in Graz verloren und diese 1945 wieder zurückbekommen, übernahm jedoch bereits 1945 supplierend und 1946 mit seiner Ernennung bis zu seinem frühen Tod die Professur in Wien. Zu ihm GABRIEL, „Wie dann der Hoff gekommen ist, hat man den Kauders geschwind vergessen" (2018), passim.

[51] GABRIEL, Hans Hoff (2019), 344-349.

verstanden sich als in dieser Tradition stehend.[52] Wie Kauders, so knüpfte Hoff einerseits an die organisch-biologische Tradition an.[53] Beide waren aber zugleich auch offen für andere Ansätze in der Psychiatrie. Die These von einer multifaktoriellen Genese psychischer Erkrankungen (Erbfaktoren, frühkindliche Traumata, soziale Faktoren), die Hoff vertrat, bedeutete für ihn die Einbeziehung sowohl organisch-biologischer, psychotherapeutischer, psychodynamischer und sozialpsychiatrischer Therapieansätze[54] in einem umfassenden, individuell nach dem Krankheitsbild des / der Patienten/in zu differenzierenden „Gesamtbehandlungsplan". [55] Kauders und Hoff griffen etwa die Psychohygiene[56] auf, die von ersterem schon in der Vorkriegszeit vertreten worden war. Hoffs Mitarbeiter und Nachfolger Peter Berner (1924-2012) nennt 1998 dazu folgende Institutionen, die auf Hoffs Initiative in Österreich eingeführt bzw. systematisch ausgebaut wurden: 1961 wurde ein Genesungsheim für Alkoholkranke (Kalksburg) und ein Rehabilitationszentrum für Schizophrene, getragen von der Caritas (Maria Lanzendorf), eingerichtet, 1963 auf Hoffs Anregung vom Justizministerium eine Sonderanstalt zur Betreuung psychisch schwieriger Rechtsbrecher (Mittersteig); auf Hoffs Initiative seien auch „die Lebensmüdenfürsorge [der Caritas], der schulpsychologische Dienst, die psychiatrische Betreuung straffälliger Jugendlicher [Kaiserebersdorf und Wiener Neudorf]" entstanden, wie auch Child-Guidance-

---

[52] Für Hoff ging es dabei – mehr als bei Kauders – nicht nur um die Ära Wagner-Jauregg, sondern auch um die Ära Pötzl: Vgl. Hoffs Antrittsvorlesung 1951 für das Ordinariat für Psychiatrie und Neurologie und die Leitung der Klinik, zitiert unten S. 172. GABRIEL, Zum Wiederaufbau des akademischen Lehrkörpers in der Psychiatrie in Wien nach 1945 (2016), S. 49, zitiert zur Verehrung und zur Instrumentalisierung Wagner-Jaureggs „*im Interesse des Wiederaufbaus des Ranges der sogenannten ‚Wiener Schule'*" die Feiern zum 90. Und zum 100. Geburtstag Julius Wagner-Jaureggs. Zu Pötzl (zu ihm vgl. unten S. 173 Anm. 645) schrieb HOFF, Professor Dr. Otto Pötzl – 75 Jahre (1952), S. 971: „Der Weg, den Pötzl beim Versuch das Geheimnis der Psychosen und Neurosen zu lüften, gewiesen hat, ist klar vorgezeichnet: er repräsentiert die Tendenzen der Wiener Schule, die wir als seine Schüler weiter zu verfolgen haben."

[53] In seiner Antrittsvorlesung als Professor am Neurologischen Institut, dem sog. „Obersteiner-Institut" – zitiert in GABRIEL, Hans Hoff (2019), S. 352 – beschrieb er Julius Wagner-Jauregg als Vorbild in dessen „Abneigung gegen jede Theorie [nach Eberhard Gabriel sind „vermutlich spekulative Konstruktionen" gemeint] und […] Ehrfurcht vor der objektiven Beobachtung" und dessen Grundsatz, „dass jede medizinische Forschung den Zweck haben muss, den leidenden Menschen zu helfen." Nach Gabriel betont dieses Zitat **„**den Primat der therapeutischen Situation und des Nutzens für den Kranken" gegenüber der Theorie.

[54] Vgl. GRÖGER, Zur Entwicklung der Psychiatrie in der Wiener Medizinischen Schule (1999), S. 40f.

[55] Für den maßgeblich dieses Konzept mitbestimmenden Wiener Kliniker Ottokar H. Arnold waren psychiatrische Krankheiten Veränderungen einer bestimmten Persönlichkeit, die sich aus der Besonderheit ihrer ontogenetischen Entwicklung und ihrer spezifischen Struktur zusammensetzten. Deshalb sei ein Gesamtbehandlungsplan nötig, in dem die unspezifische Schockbehandlung an geeigneter Stelle neben einer individuellen Psychotherapie und „Maßnahmen psychohygienischer Betreuung und Fürsorge" eingesetzt würden, und es seien „die Schocktherapie ausschließlich in der Hand eines Therapeuten zu belassen, der eine seelische Störung nur als einen Teilaspekt in der Dynamik der Gesamtpersönlichkeit zu betrachten und zu behandeln entschlossen ist": ARNOLD, Schockbehandlungen (1954), Teil II, S. 53.

[56] Hans Hoff wurde 1951 in der Nachfolge von Otto Kauders zum Präsidenten der Österreichischen Gesellschaft für psychische Hygiene gewählt, die 1948 gegründet worden war. 1953 und 1958 wurde der Kongress der World Federation for Mental Health (1948 in London gegründet) unter seiner Leitung in Wien abgehalten. Vgl. BERNER, Hans Hoff (1998), S. 62; GRÖGER, Die Entwicklung der psychischen Hygiene (2012), S. 221-232.

Kliniken, Ehe- und Familienberatungsstellen und das Kriseninterventionszentrum.[57] Vieles davon basierte auf Initiativen von Kauders nach 1945, die durch dessen frühen Tod im Jahr 1949 von ihm nicht zu Ende geführt werden konnten und von Hoff im Sinne seiner expansiven Klinikpolitik fortgeführt wurden.[58] Als Präsident der Österreichischen Gesellschaft für psychische Hygiene betonte Hoff 1952 die Vorreiterrolle seines Vorgängers auch in dieser Funktion und erweiterte das Komitee als guter Netzwerker mit führenden Ministerial- und städtischen Beamten, mit Psychologen und Medizinern, die in der Planung und Ausführung von Projekten aufgrund ihrer Position und ihres Fachwissens nützlich sein konnten – wie Hoff es ausdrückte: „um unserer Arbeit eine praktische Linie zu geben".[59]

Positiv beurteilt wurde seine rasche Zusammenarbeit mit dem Innenministerium, um psychohygienische Maßnahmen in der Betreuung der Ungarnflüchtlinge 1956 durchzusetzen.[60] Hoff betraute damit seinen Mitarbeiter Hans Strotzka (1917-1994), der in den folgenden Jahren (und nach ihm Peter Berner bis 1965) Berater des Flüchtlings-Hochkommissars in Genf in der Versorgung von Flüchtlingen als Menschen mit einem besonderen Risiko für psychische Störungen wurde.[61]

1967, in einer Ausgabe der *Wiener Medizinischen Wochenschrift* zum 70. Geburtstag von Hans Hoff, beschrieben seine Mitarbeiter – dem Anlassfall entsprechend überschwänglich – diese bereits erwähnten Initiativen des Jubilars und bemerkten zu seiner Öffentlichkeitsarbeit: seine „zahlreiche[n] Eingaben an Behörden des Sanitätswesens und des öffentlichen

---

[57] BERNER, Hans Hoff (1998), S. 61-62. Vgl. ARIAS, Hans Hoff (2016), S. S. 185-187; Ingrid Arias sieht Hoffs Initiativen zur Sozialpsychiatrie und Psychohygiene als seine wesentlichen neue Impulse in der Wiener Psychiatrie.
[58] Einschätzung von Eberhard Gabriel, mit der er in Gesprächen mit Peter Berner übereinstimmte.
[59] HOFF, Tätigkeitsbericht (1952), S. 47-55; er nennt: Frau Dr. Redlich im Sozial- und Sektionsrat Dr. Lang im Unterrichtsministerium, Viktor Frankl (als Verbindungsmann zur Presse), Anton Tesarek (Leiter des städtischen Jugendamtes), Hans Asperger (damals Dozent an der Kinderklinik und Heilpädagoge), Albert Niedermeyer (er publizierte über ärztliche Seelsorge, Pastoralmedizin), Alfons Huber (Leiter der Frauenheilanstalt am Steinhof bis 1950), Herbert Reisner (nun ärztlicher Direktor am Rosenhügel; er sollte sich der Psychopathie widmen); zum Problem des Alkoholismus nennt Hoff keinen Namen; Dr. R. K. Jech (publiziert 1951 über „Die psychische Hygiene in der Industrie" in der Zeitschrift für Nervenheilkunde), Dr. Tuchmann (Chefarzt der Wiener Gebietskrankenkasse), Hubert Rohracher (Univ. Prof. f. Psychologie).
[60] Vgl. zu dieser Maßnahme HOFF/ STROTZKA (Hrsg.), Die psychohygienische Betreuung ungarischer Neuflüchtlinge in Österreich 1956-1958 (1958), passim.
[61] Vgl. BERNER, Hans Hoff (1998), S. 62. Peter Berner war seit 1951 an der Klinik, Ende der 1950er Jahre bis ca. 1967 mit längeren Unterbrechungen durch seine Tätigkeit in Genf und Lausanne Oberarzt der Frauenabteilung der Psychiatrie, 1969 interimistischer Leiter der Klinik nach dem Tod Hans Hoffs und ab 1971 o. Prof. und Leiter der – nun von der Neurologie getrennten – Klinik für Psychiatrie: lt. Gespräch Peter Berner mit Gernot Heiss am 14.5.2012 in Paris.

Lebens“[62] hätten „letztlich immer nach der vernünftigen psychologisch fundierten Handhabung sozialer Regulative“ gezielt.[63] Ausführlich gehen sie auch auf die baulichen und organisatorischen Veränderungen an der Klinik ein:[64] Im Unterschied zum Eindruck, den das über 100 Jahre alte Gebäude mit „über 200 psychiatrische[n] und 100 neurologische[n] Betten“ Anfang der 1950er Jahre als den einer „alten klassischen Irrenanstalt hervor[brachte]“, sei „heute […] die Klinik offen, die Fenstergitter sind entfernt und es gibt keine Gitterbetten mehr“. „Beruhigungsräume für akute Aufnahmen“, „Ausbau der kinderpsychiatrischen Abteilung,[65] einer psychosomatischen Abteilung, der Multiple-Sklerose-Abteilung und der Rehabilitationsstation für Schädel- und Hirnverletzte […]. Neben der allgemeinen Ambulanz betreibt die Klinik Spezial-Ambulanzen für Epileptiker, Multiple-Sklerose-Kranke, eine kinderpsychiatrische Ambulanz, eine Eheberatung und eine Beratungsstelle für alte Leute sowie eine psychotherapeutische Ambulanz.“[66] Weiters werden hier als Einrichtungen der psychiatrischen Abteilung genannt: „Arbeitsräume neben der zentralen Arbeitstherapie und Räume, in denen kreative (z.B. Malen) Arbeitstherapie geleistet wird“, ein „moderner Sporthof, ein eigenes Theater (Psychodrama), eine musiktherapeutische Institution, eine Tanztherapiegruppe und […] Therapiegruppen der psychiatrischen, psychosomatischen und Rehabilitationsabteilungen.“ Die Einrichtung des psychologischen Laboratoriums, wie die des biochemischen Laboratoriums,[67] wird als Leistung Hoffs genannt.

---

[62] Hoff war Mitglied des Obersten- und des Wiener Sanitätsrates: GABRIEL, Hans Hoff (2019), S. 350.
[63] ARNOLD/TSCHABITSCHER, Die Wiener Neurologisch-Psychiatrische Schule unter H. Hoff (1967), S. 1131.
[64] Ebd., S. 1128-1131. Längere Zitate zur Neurologie werden hier nicht wiedergegeben.
[65] Unter Kauders wurde „eine Kinder- und Jugendneuropsychiatrische Ambulanz“ eingerichtet und in Weiterführung dieser Planung nach dem Tod von Kauders (1949) vom interimistischen Leiter Herbert Reisner zur stationären Betreuung ein Zimmer zur Verfügung gestellt. Unter Hoff wurde schließlich bereits im Oktober 1951 die „Kinderstation mit sieben Betten“ feierlich und öffentlichkeitswirksam eröffnet, mit dem bereits unter Kauders eingestellten Walter Spiel fortgeführt und mit amerikanischen Hilfsgeldern ausgebaut. Bisher hatte aufgrund einer alten Übereinkunft zwischen Clemens von Pirquet und Julius Wagner-Jauregg die Kinderklinik die Behandlung von psychisch erkrankten Kindern unter 14 Jahren übernommen. Vgl. die Schilderung in SPIEL/MUTSCHLECHNER/SCHAUFLER/STÜTZ, Die Entstehung des Fachgebietes Kinder- und Jugendneuropsychiatrie (1994), S. 10-11 und S. 14-15. Vgl. auch SPIEL, 25 Jahre Neuropsychiatrie des Kindes- und Jugendalters in Wien (1977), S. 3.
[66] Dazu wären nach einer mündlichen Mitteilung von Alfred Springer (seit Mitte der 1960er Jahre an der Klinik) die Sprechstunden der Ärzte/innen zur Beratung der Angehörigen zu ergänzen, eine Einrichtung, die im Sinne der Psychohygiene das soziale Umfeld des /der Patienten/in in die Therapie einbezog.
[67] Vgl. ARNOLD/TSCHABITSCHER, Die Wiener Neurologisch-Psychiatrische Schule unter H. Hoff (1967), S. 1130 zur Charakterisierung Hoffs als Förderer der Forschungen seiner Mitarbeiter wird hier auf die Forschungen des Mitautors Ottokar H. Arnold hingewiesen, dessen Interesse den körperlichen Ursachen der Schizophrenie und ihrer Symptome galt: „Das biochemische Laboratorium der Klinik wurde für die Bearbeitung von Problemen der Grundlagenforschung geschaffen. Die wichtigsten Probleme, die derzeit bearbeitet werden, sind der Versuch, die biochemische Störung hinter dem Genotypus der Schizophrenie zu erfassen und näher zu charakterisieren. Weiters wird der Problemkreis des Alkoholismus, insbesondere Fragen der Membranpermeabilität und des Lipoidstoffwechsels bearbeitet.“ Mit Förderung durch Hoff habe die „Arbeitsgruppe“ Arnold und Hofmann „in der Biochemie des Kohlehydrat-Phosphatstoffwechsels bei Psychosen in 15 Jahren systematisch

Es wurde darin hervorgehoben, dass Hoff „die seelischen Störungen des Menschen unter dem Blickwinkel der biologisch-biochemischen, hirnpathologisch-neurophysiologischen und tiefenpsychologisch-sozialen Faktorengruppen“ betrachtete. Hoff habe „demonstriert, daß es um die Integration der biologisch-medizinischen, der neurophysiologisch-hirnpathologischen und der tiefenpsychologisch-psychopathologischen Forschungsrichtungen in jedem einzelnen Krankheitsfall“ gehe.[68] Diese Beschreibung der Position Hoffs (und der Klinik) trifft die Selbstdefinition im Rahmen des – nach Hoffs Anspruch – ‚Wiener‘ Konzepts der multifaktoriellen Genese psychischer Erkrankungen und des ‚Gesamtbehandlungsplans‘.[69] Darin behielt seine starke Prägung durch die organisch-biologische Tradition der Wiener Psychiatrie ihren hohen Stellenwert für die Praxis bei.[70]

Zu allen diesen in der Festschrift 1967 genannten Aktivitäten und Wirkungsfeldern sind noch seine Positionen in den Wiener und internationalen psychiatrisch-neurologischen Gesellschaften und Publikationsorganen zu nennen,[71] sowie seine internationalen Kontakte: Durch die beruflichen und persönlichen Kontakte Hoffs, der in der Emigration in Bagdad und New York gelehrt hatte, und wohl auch noch aufgrund des Ansehens der Wiener Psychiatrie der Zwischenkriegszeit hatte die Wiener Klinik viele ausländische PatientInnen und konnte Hoff vielen seiner jungen MitarbeiterInnen Auslandsaufenthalte ermöglichen und ausländische Förderungen lukrieren.[72] Von MitarbeiterInnen wurde er als autoritär

---

Laboratoriumstechniken und Methoden ausbauen können, deren Ergebnisse eben jetzt international zur Diskussion stehen“. Vgl. dazu den Hinweis von Eberhard Gabriel unten S. 84 Anm. 287.

68 ARNOLD/TSCHABITSCHER, Die Wiener Neurologisch-Psychiatrische Schule unter H. Hoff (1967), S. 1130 bzw. S. 1128. Vgl. dazu auch das ausführliche Zitat zu den „biologische[n] Basistherapie[n]“ unten S. 206f.

69 Hoff und sein Mitarbeiter Ottokar Heinrich Arnold vertraten dieses Konzept (multifaktorielle Genese und Gesamtbehandlungsplan) als Konzept der Wiener Schule. Sehr ähnliche Konzepte vertraten freilich auch andere: Vgl. etwa Henri Claude, 1922 bis 1939 Direktor der psychiatrischen Klinik St. Anne in Paris, und sein Co-Autor Rubenovich, die betonten, dass alle drei Bereiche, der biologische, psychologische und soziale, in der Behandlung einbezogen werden müssten: COFFIN, French Biological Therapeutics in the European Context (2013), S. 194 zitiert dazu CLAUDE/RUBINOVITCH, Thérapeutiques (1940), S. 333-335. Vgl. GABRIEL, Hans Hoff (1897-1969) (2019), S. 353-355 zu gleichen Konzepten und zur Kritik des Wiener Konzepts, das „bei der autoritativen Äußerung einer Meinung ohne einen Ansatz zu deren vertiefter Begründung durch Forschung geblieben“ sei.

70 Zu Hoffs „Verwurzelung in organische[n] Krankheitskonzepte“ und seiner Ablehnung „rein psychogene[r] Entstehung“ schizophrener und manisch-depressiver Psychosen: BERNER, Hans Hoff (1998), S. 59, zitiert unten S. 171. Dazu auch Hoffs Aussage 1950 über die Position seines Lehrers und Vorbilds Wagner-Jauregg, vgl. unten S. 23 Anm. 53.

71 Detailliert GABRIEL, Hans Hoff (2019), S. 350.

72 Vgl. SPIEL, 25 Jahre Neuropsychiatrie des Kindes- und Jugendalters in Wien (1977), S. 3f. zu den Studienaufenthalten Walter Spiels und seiner MitarbeiterInnen und zur Förderung der Kinderstation durch die Rockefeller-Foundation 1954 bis 1958 und danach durch die Foundation for Research in Psychiatry der Universität New Haven und die Child-Stiftung, sowie zu den internationalen Tagungen in Wien; vgl. ARIAS, Hans Hoff (2016), S. 188.

beschrieben, der jedoch seine MitarbeiterInnen auch hat „machen lassen“[73], und der „mit großer organisatorischer Energie“ und „rastloser Aktivität“, „sein Ziel der Wiederherstellung des Ranges dieser Institution verfolgt“ habe.[74] Die lange Liste der Wirkungsfelder zeigt Hoffs erstaunlich umtriebige und erfolgreiche Tätigkeit als Organisator und Netzwerker, wobei er – obwohl NS-Verfolgter und anders als sein Vorgänger Otto Kauders – wenig Berührungsängste mit Kollegen hatte, die Mitglieder in nationalsozialistischen Organisationen gewesen waren.[75]

2 Die „’großen’ körperlichen Behandlungsverfahren“ in der zeitgenössischen Literatur[76]

Zentrales Thema dieser Untersuchung zur Wiener Psychiatrie zwischen 1951 und 1969 ist die Anwendung der in der Zwischenkriegszeit entwickelten somatischen Therapien, die aufgrund ihrer massiven körperlichen Intervention auch als „heroische Therapien“[77] bezeichnet wurden. Zu ihrer historischen Positionierung dienen in diesem einleitenden, allgemeinen Abschnitt vor allem Publikationen Manfred Bleulers,[78] der damals Direktor der Psychiatrischen Universitätsklinik Zürich „Burghölzli“ war und der die „umgearbeiteten“ Neuauflagen des Lehrbuchs der Psychiatrie seines Vaters seit der 7. Auflage von 1943 betreute.

Manfred Bleuler, der nach seinem Vater Eugen Bleuler der anerkannte Spezialist für schizophrene Erkrankungen war, hielt 1947 auf Einladung des Vorstands der Wiener Klinik

---

[73] GABRIEL, Hans Hoff (2019), S. 349f. zitiert Gespräche mit und autobiographische Quellen von Mitarbeitern – die zitierten Worte von Raoul Schindler.

[74] Ebd., S. 358-360.

[75] Hans Hoff publizierte etwa einen Beitrag in der Festschrift für seinen sehr verehrten Lehrer Otto Pötzl (Leiter der Klinik nach Wagner-Jauregg 1928 bis 1945) – vgl. URBAN (Hrsg.), Pötzl-Festschrift (1947) –, obwohl ihn Kauders wegen der NS-Vergangenheit einiger der Beitragenden davor gewarnt haben soll. Hoff förderte Franz Seitelberger, seinen Stellvertreter, dann Nachfolger in der Leitung des Neurologischen Instituts der Universität Wien, trotz dessen SS-Mitgliedschaft. Er lehnte es jedoch ab, Heinrich Gross, der an den Euthanasie-Verbrechen am Spiegelgrund, also direkt an NS-Verbrechen beteiligt gewesen war, zu habilitieren.

[76] In der zeitgenössischen Literatur und in den Krankenakten wird fast ausschließlich von Insulinschock (IS), Cardiazolschock und Elektroschock (ES) geschrieben. Diese Bezeichnungen werden in den wörtlichen Zitaten selbstverständlich beibehalten, im Text werden jedoch meistens die Therapiebezeichnungen Insulinkoma-, Cardiazolkrampf- und Elektrokrampftherapie (EKT) gewählt. Die Bezeichnung dieser körperlichen Kuren als Schocktherapien, wie sie auch in neueren Publikationen verwendet wird, geht davon aus, dass diese Therapien auf unterschiedliche Weise einen ‚Schock‘ auslösen, der therapeutische Wirkung hat. So nennen etwa BARATTA/MORALI, Les traitements biologiques en psychiatrie entre la seconde moitié du XIXe siècle et la première moitié du XXe siècle (2010), S. 539-547 (20.1.2020: https://www.cairn.info/revue-l-information-psychiatrique-2010-6-page-539.htm) die Malariafiebertherapie als erste der « traitements de choc » und schreiben : « La plupart de ces traitements visent à provoquer un choc : thermique pour la malariathérapie, convulsif pour les cures de Sakel [Insulinkomatherapie, GH] et au Cardiazol, cardiaque pour le choc acétylcholinique. »

[77] Vgl. den Titel des Sammelbandes von SCHMUHL/ROELCKE (Hrsg), „Heroische Therapien“ (2013).

[78] Zum „Burghölzli“ hatte die „Klinik Hoff“ gute Kontakte, was diese Schwerpunktsetzung motivierte: So waren die Wiener Ärzte Raoul Schindler und Eberhard Gabriel in diesen Jahren auch an der Züricher Klinik; Hans Hoff korrespondierte mit Manfred Bleuler; vgl. GABRIEL, Hans Hoff (2019).

Otto Kauders im soeben wiedergegründeten „Verein für Psychiatrie und Neurologie“ einen Vortrag über „Forschungen zur Schizophreniefrage“.[79] Er berichtete über die Ergebnisse seiner Untersuchung von Fällen von ‚schockgeheilten‘ und ‚schockresistenten‘ Schizophrenien mit deutlicher Kritik an den enthusiastischen Berichten über große Erfolge der ‚Schockkuren‘, d.h. der ‚großen‘ körperlichen Kuren bei Schizophrenen. In Bezug auf die Behandlung der Schizophrenie mit diesen Therapien („mit Fieber, Dauernarkose, Insulin, Cardiazol- und Elektroschock“) argumentierte er, diese seien „nur als moderne Methoden der uralten Erschütterungstherapien“[80] zu sehen. Wie „die geduldige Erziehungs- und Gewöhnungstherapie“[81] sei „die Erschütterungstherapie“ jedoch eine Methode „an die Krankheit heranzukommen“. Beide seien keine kausalen Behandlungsmethoden, „sie können aber die Heilung beschleunigen und bessernd und sozialisierend in allen Fällen [von schizophrenen Erkrankungen, GH] wirken. Ihr Anwendungsbereich geht weit über die Grenzen der Schizophrenie hinaus und umfasst alle psychopathischen Zustände, die noch nicht kausal angegangen werden können.“[82]

Das war vor der Einführung der neuen Psychopharmaka. In den neu bearbeiteten Auflagen des renommierten Lehrbuchs der Psychiatrie von 1955, 1960 und 1969 (den drei Auflagen, die in den Untersuchungszeitraum fallen) geht Bleuler im Abschnitt über die „‚großen‘, kurmäßig durchgeführten, somatischen Behandlungsverfahren“ – er nennt in der Reihenfolge ihrer Einführung „die Fieberbehandlung (WAGNER von JAUREGG 1917)“ an erster Stelle, dann „die Schlafkur oder Dauernarkose (KLAESI 1920), die Insulinkur (SAKEL 1935), die Cardiazol-Krampf-Behandlung (MEDUNA 1934) und die Elektroschock-Behandlung (BINI u. CERLETTI 1937)“ – auf die Neuerungen durch die Entwicklung der Neuroleptika ein, die er in „die ‚großen‘ kurmäßig durchgeführten Behandlunsverfahren“ einbezieht: „Seit 1952 hat die kurmäßige Anwendung von verschiedenen neuen Medikamenten (besonders Phenothiazinen [Chlorpromazin 1952] und Rauwolfia-Alkaloiden [Reserpin 1954]), die eine

---

[79] BLEULER, Forschungen zur Schizophreniefrage (1948), S. 145-147.
[80] „sog. ‚Erschütterungsbehandlungen‘“ bereits bei BLEULER, Lehrbuch der Psychiatrie ([7]1943), S. 332. BLEULER, Lehrbuch der Psychiatrie ([10]1960), S. 403 führt zu ihrer Anwendung aus: „In der richtigen Abwechslung zwischen dem ununterbrochenen Appell an die gesunden psychischen Kräfte der Schizophrenen und gelegentlichen plötzlichen schweren Eingriffen in ihre gewohnten körperlichen und psychischen Daseinsformen scheinen heute wichtige Beeinflussungsmöglichkeiten der Schizophrenie zu liegen.“
[81] „Zu ihr gehören in erster Linie die Arbeitstherapie, die richtige Freizeitgestaltung, die Angewöhnung an Ordnung und Gemeinschaftsleben in der Anstalt, das geduldige Bemühen darum, mit dem Kranken in einen geordneten Kontakt zu kommen – kurz, ein großer Teil der Milieutherapie einer modernen Anstalt und eines modernen Psychotherapeuten.“
[82] BLEULER, Forschungen zur Schizophreniefrage (1948), S. 147.

Beruhigung von bisher nicht gekannter Art setzen, eine rasch zunehmende Bedeutung gewonnen."[83] Das habe „den Anwendungsbereich" der älteren Therapien wesentlich eingeengt.[84]

Das bedeutete jedoch keineswegs, dass er ihre Anwendung ablehnte, deren Technik und auch „Hauptgefahren" in diesem Abschnitt des Lehrbuchs erläutert werden – mit Ausnahme der „Fieberkur", zu der er auf die Ausführungen im Abschnitt über die progressive Paralyse verwies, da sie bei ihr „in der Hauptsache von spezifischer Wirksamkeit" sei.

Auf die Frage „Wieso sind alle die beschriebenen ‚großen' körperlichen Kuren bei psychiatrischen Kranken oft wirksam?" heißt es in den drei Lehrbüchern: Zwar sei ihnen mit der Ausnahme der Fieberkur bei progressiver Paralyse „eine spezifische oder kausale Wirksamkeit für die eine oder andere psychisch Störung" nach heutigem Wissensstand „abzusprechen", sie würden jedoch „symptomatisch" wirken. Für ihre Wirksamkeit würden „bei allen von ihnen" folgende Gründe in Frage kommen: „Die kurmäßige Beruhigung" war ein zentrales Ziel ihrer Anwendung, (1) etwa beim Morphium-Entzug, bei vielen depressiven Zuständen, bei akuter schizophrener Erregung. Sie würde „über besonders quälende und gefährliche Krankheitsperioden hinweg[helfen]". (2) „Die vorübergehende Ruhigstellung, die alle Kuren mit sich bringen," würden „beim Unterbrechen eines […] *Circulus vitiosus* [der „vielen Verschlimmerungen von psychotischen Zuständen" zugrunde liege[85]] helfen, indem sie Zeit zur Besinnung, zum Abstandnehmen von den psychotischen Erlebnissen schafft." (3) Alle Kuren würden „zu einer intensiven Beschäftigung von Ärzten und Schwestern mit dem Kranken" führen, und „einen Kontakt mit ihm, und zwar in einer packenden, elementaren Art" ermöglichen. (4) Die Kuren würden „als eine Belastung des Organismus (‚stress') [wirken] und […] nach körperlichen und psychischen unspezifischen Anpassungsreaktionen [rufen]. Von denselben sind veränderte Nebennierenrinden-Funktionen am besten bekannt. Vielleicht kommt auch solchen Vorgängen therapeutische Bedeutung zu."

---

[83] BLEULER, Lehrbuch der Psychiatrie ([9]1955), S. 154; BLEULER, Lehrbuch der Psychiatrie ([10]1960), S. 151; BLEULER, Lehrbuch der Psychiatrie ([11]1969), S. 159.

[84] Ebd., in allen drei Auflagen. Lt. BLEULER, Lehrbuch der Psychiatrie ([10]1960), S. 160, hatte bereits nun an einzelnen Kliniken „die Kur mit Chlorpromazin und ähnlichen Körpern […] alle anderen Kuren verdrängt".

[85] Bleuler erklärt die Abwärtsspirale wie folgt: „wenn einmal eine psychotische Abwendung von der Umgebung erfolgt ist, so entstehen schon daraus neue Schwierigkeiten: der Kranke ist erzürnt über die sozialen Folgen seines Verhaltens oder er ist mit Schuld- und Schamgefühlen belastet; dadurch wird er tiefer in den psychotischen Rückzug von der Welt gestoßen."

Im folgenden Absatz kommt Bleuler noch einmal auf den Kontakt zum Patienten / zur Patientin aufgrund der intensiven „ärztlichen und pflegerischen Maßnahmen“ zurück. Dadurch würden sich „psychotherapeutischen Möglichkeiten“ ergeben, die vor allem am Ende der Kur aktiv genutzt werden müssten.[86]

Zu den „‚großen’, kurmäßig durchgeführten, somatischen Behandlungsverfahren“, die nach Bleuler zwar „bei der Schizophrenie am häufigsten, jedoch keineswegs ausschließlich“ angewandt würden,[87] werden Indikationen vor allem im Kapitel über „die ‚endogenen‘ Geistesstörungen“ genannt.[88] Die „Fieber-, Schlaf-, Cardiazol-, Elektroschock-, Insulin-, Chlorpromazin-, Rauwolfia-Kur“ hätten in „den letzten Jahrzehnten […] neben der erziehenden und kontaktschaffenden Behandlung und neben der psychischen Überraschungsbehandlung […] weiteste Verbreitung gefunden“. Zwar seien sie „weit überschätzt worden“, sicher sei „trotzdem, daß sich an die Behandlung in frischen Fällen oft unmittelbar Heilungen und in chronischen doch weitgehende Besserungen in eindrucksvoller Weise anschließen. Die Verfahren sind geeignet, akute Schübe abzukürzen und chronische Kranke zu sozialisieren, und sind deshalb eine wertvolle Bereicherung unserer Therapie.“[89]

Zur Indikation der genannten körperlichen Behandlungsverfahren bei schizophren Erkrankten heißt es in den Ausgaben des Lehrbuchs von 1955, 1960 und 1969: Man könne „bei akuten Schizophrenen mit gutem Verlauf und bei sozial gut angepassten chronischen Schizophrenen auf sie verzichten. Die Hauptindikation ist bei frischen Fällen gegeben, die nicht rasch unter psychotherapeutischer Führung zur Besserung neigen. Wichtig ist die körperliche Behandlung auch, wenn ein Schub zum Abklingen neigt und der Patient trotzdem von Wahnvorstellungen und triebhaften Handlungen oder von der Furcht vor der Arbeit und dem Leben nicht loskommt. Ferner können besondere Schwierigkeiten (Stupor, Erregung, Gewalttätigkeit, Unsauberkeit, Nahrungsverweigerung usw.) in akuten und chronischen Zuständen erfolgreich mit einem der körperlichen Verfahren angegangen werden.“[90] „Schlaf-, Insulin- oder Krampf-

---

[86] BLEULER, Lehrbuch der Psychiatrie (^{9}1955), S. 159f; BLEULER, Lehrbuch der Psychiatrie (^{10}1960), S. 159; BLEULER, Lehrbuch der Psychiatrie (^{11}1969), S. 164.
[87] BLEULER, Lehrbuch der Psychiatrie (^{10}1960), S. 152; BLEULER, Lehrbuch der Psychiatrie (^{11}1969), S. 159. Vgl. auch oben S. 28 das Zitat aus BLEULER, Forschungen zur Schizophreniefrage (1948), S. 147.
[88] BLEULER, Lehrbuch der Psychiatrie (^{10}1960), S. 401-406, 425-430; BLEULER, Lehrbuch der Psychiatrie (^{11}1969), S.416-423, 445-453.
[89] BLEULER, Lehrbuch der Psychiatrie (^{9}1955), S. 375f; BLEULER, Lehrbuch der Psychiatrie (^{10}1960), S. 403f.; BLEULER, Lehrbuch der Psychiatrie (^{11}1969), S. 420f.
[90] Ebd.

Kur oder […] medikamentöse Beruhigung mit Chlorpromazin oder ähnlichen Präparaten" (also ohne die Fieberkuren zu nennen) kämen in Frage, wenn die „bestehende Geistesstörung […] nicht kausal oder psychotherapeutisch mit genügender Aussicht auf Erfolg angegangen werden" könne und sie „einen Schweregrad erreicht" hätte, „der höhere Gefahren, Leiden und Unannehmlichkeiten in sich" trüge „als die Kur selbst"; es kämen „fast nur Geistesstörungen in Frage, bei denen Erregungen, Verstimmungen, Unrastzustände, Autismus, Verwirrungen oder andere Ausnahmezustände im Vordergrund stehen, oft mit gefährlichen Verhaltensweisen, wie Nahrungsverweigerung, Aggression, Selbstbeschädigungs- oder Selbstmordtendenzen u. ä."[91] „Die großen körperlichen Kuren (Dämmer-, [Dauer-]Schlaf-, Insulinschock- und Elektroschock-Kuren) dienen in verschiedener Art und in verschiedenem Ausmaß allen drei genannten therapeutischen Erfordernissen: der Bildung eines engeren Verhältnisses mit Schwestern, Pflegern und Ärzten, der Überraschung und Erschütterung und der Beruhigung. […] Bei der Auswahl des körperlichen Verfahrens kommt es auf die Dringlichkeit des Eingriffes, den körperlichen Zustand, das vorliegende Syndrom (Stupor oder Erregung, depressive oder maniforme Verstimmung u.a.) und individuelle günstige oder ungünstige Erfahrung mit der einen oder anderen Behandlung an." [92]

Der „Fieber[kur]" wird zwar in die nicht „spezifisch oder kausal" wirkenden körperlichen Kuren eingereiht, in ihrer unspezifischen Anwendung als „Erschütterungs-" bzw. „Schocktherapie" wird sie jedoch kaum erwähnt bzw. (1969) als überholt bezeichnet.[93] Möglich, dass im Fall der Malariakur die Praxis Bleulers an der Züricher Klinik, wo die Anwendung der Malariakur auch bei progressiver Paralyse bereits in den 1950er Jahren zu Ende ging, Einfluss auf die Ausführungen im Lehrbuch hatte. Anders bei der Insulinkomatherapie, sowie bei der Cardiazol- und der Elektrokrampftherapie, bei denen sich das Lehrbuch – seiner Funktion als allgemeine Informationsquelle entsprechend – nicht einschränkend auf die eigene Praxis bezog, sondern auf akzeptierte Vorgehensweisen, auch wenn in Zürich nicht mehr damit gearbeitet wurde.

---

[91] BLEULER, Lehrbuch der Psychiatrie ([9]1955), S. 160; BLEULER, Lehrbuch der Psychiatrie ([10]1960), S. 160f; BLEULER, Lehrbuch der Psychiatrie ([11]1969), S. 165.
[92] BLEULER, Lehrbuch der Psychiatrie ([11]1969), S. 425.
[93] BLEULER, Lehrbuch der Psychiatrie ([10]1960), S. 405: die Fieberkur sei „selten wirksam" und würde „man heute nur noch anwenden, wenn die anderen körperlichen Methoden versagt haben"; BLEULER, Lehrbuch der Psychiatrie ([11]1969), S. 422: „Fieberkuren sind mit Recht beinahe außer Gebrauch gekommen". Bereits BLEULER, Lehrbuch der Psychiatrie ([7]1943), S. 330: „Die Fieberkur Schizophrener geht auf Wagner-Jauregg zurück […]. Die Erfolge scheinen hinter den anderen Methoden deutlich zurückzustehen, immerhin wird doch nicht allzu selten von der Fieberbehandlung ein umstimmender Einfluß auf die Psychose gesehen."

Aus dem Projektbericht von 2002 der Untersuchung der Züricher Praxis zwischen 1870 und 1970 mit einer Auswertung der PatientInnenakten in Stichjahren mit einem Abstand von zehn Jahren[94] wird ersichtlich, dass an der Züricher Psychiatrie die Malariakur im Stichjahr 1929 ihren Höhepunkt hatte und ihre Anwendung in den 1950er Jahren zu Ende ging. Die 1930er Jahre „können als eigentlicher Beginn einer Kur-Periode im Burghölzli bezeichnet werden. In rund zwanzig von hundert Eintritten dieses Jahrzehnts wurde eine Kur durchgeführt. So etwa auch die auf diesen Zeitraum beschränkten Cardiazolkuren.“ Die Elektrokrampftherapie setzte sich hier sehr rasch durch: „Rund ein Sechstel der im Stichjahr 1949 eingetretenen PatientInnen wurden mit Elektroschock behandelt. Ebenso erreichten Insulinkuren und Schlafkuren[95] in diesem Jahr Höchstwerte.“ Alle drei Kuren nehmen bereits in den 1950er und dann stark in den 1960er Jahren ab. Am ‚Burghölzli' dürften Ende der 1950er Jahre die Malariakur[96] und Mitte der 1960er die Insulinkur nicht mehr und die Elektrokrampftherapie kaum noch angewandt worden sein.[97] Der Abschluss der Kurperiode fällt in den 1960er Jahren „mit dem Beginn einer neuen Phase zusammen: 1959 ist das erste Stichjahr, das den Einsatz von Neuroleptika verzeichnet.[98] Ihr Einsatz ist fulminant und veranschaulicht den so genannten ‚pharmakologischen Paradigmenwechsel' der Psychiatrie […]. Über die Hälfte der 1959 aufgenommenen PatientInnen wurden mit Neuroleptika behandelt. Dieser Wert steigt im letzten Stichjahr [1969] noch weiter an und wird nun zusätzlich durch den schnell ansteigenden Wert der Antidepressiva ergänzt.“[99]

[94] TANNER/MEIER/HÜRLIMANN/BERNET, Zwangsmassnahmen in der Züricher Psychiatrie (2002), S. 93-95 im Unterkapitel „Therapie statt Zwang?“. Eugen und Manfred Bleuler, die beiden Autoren des Lehrbuchs waren Direktoren der Züricher psychiatrischen Universitätsklinik Burghölzli: Eugen Bleuler 1898-1927, Manfred Bleuler 1942-1969.
[95] In den 1920er Jahren vom Schweizer Psychiater Jakob Klaesi entwickelte Kur, damals mit dem Medikament Somnifen, im Untersuchungszeitraum mit Neuroleptika. In unserer Datenbank findet sich nur eine Anwendung von Somnifen 1950 in Wien: vgl. zum Patienten S4636 unten S. 224.
[96] Dazu, dass an der Züricher Klinik die Malariakur nicht mehr angewandt wurde, könnte auch die Schwierigkeit, malariainfiziertes Blut zu bekommen, ausschlaggebend gewesen sein. Zu diesen Schwierigkeiten findet sich in der persönlichen Korrespondenz Manfred Bleulers in Staatsarchiv des Kantons Zürich (Signatur Z 99.1047 und Z99.1048) ein Briefwechsel aus 1948/49 (zwischen Zürich, Lausanne und München) zu einer nicht erfolgreichen Suche nach malariainfiziertem Blut (ich danke Eberhard Gabriel für diesen Hinweis).
[97] So die Erinnerungen von Eberhard Gabriel zur IKT und zur EKT, der 1966 am Burghölzli gearbeitet hat, bevor er an die Wiener Klinik kam, und so die Kurven in der Grafik zu 1969 im Forschungsbericht von 2002 – TANNER/MEIER/HÜRLIMANN/BERNET, Zwangsmassnahmen in der Züricher Psychiatrie (2002), S. 93-95.
[98] „1954 wurde im Burghölzli das Chlorpromezin-Präparat ‚Largactil' eingeführt.“
[99] „Antidepressiva […] im Burghölzli ab 1958.“ Vgl. das Zitat aus dem Forschungsbericht der Klinik von 1953 zum Einsatz der neuen Medikamente in TANNER/MEIER/HÜRLIMANN/BERNET, Zwangsmassnahmen in der Züricher Psychiatrie (2002), S. 94f. unten S. 298f. Anm. 1135.

Die Unterschiede zwischen Lehrbuch und Züricher Praxis in den 1950er und 1960er zeigen, dass die Wahl aus der Vielfalt der therapeutischen Möglichkeiten, die das Bleuler'sche Lehrbuch nennt, von der individuellen Entscheidung des Psychiaters abhing. Dazu schreibt Manfred Bleuler, die unterschiedlichen Präferenzen in der Entscheidung für die einen oder anderen dieser Kuren, „der Wirrwarr der Meinungen" sei „durchaus verständlich, wenn man weiß, daß bei ein und derselben Krankheit bald die eine, bald die andere Kur wirksam oder unwirksam ist." Ihm selbst scheine wichtig – so formuliert Bleuler vorsichtig –, immer die ungefährlichste Kur zu wählen, „die wirksam ist" und das sei „soweit wir bis heute wissen, [...] diejenige mit Phenothiazinen und ähnlich wirkenden Körpern".[100] Es ging um einen Behandlungsplan je nach Beurteilung des / der einzelnen Patienten/in, wobei die Therapiewahl des Arztes / der Ärztin aufgrund seiner / ihrer Einschätzung des /der einzelnen Patienten/in akzeptierte wurde.[101]

Diese Offenheit und – wie es sich auch in Zitaten zur Insulinkomatherapie in einer französischen Umfrage Ende der 1970er Jahre zeigt[102] – auch positive Beurteilung der Indikationsentscheidung, wenn der / die betreffende Arzt / Ärztin „günstige" Erfahrungen damit gemacht hatte, entsprach der Meinung, die als Argument gegen eine Standardisierung der Therapien geäußert wurde, dass die Therapie in jedem Einzelfall nach den Besonderheiten der Symptome und des Krankheitsverlaufs zu entscheiden sei. Das dürfte besonders bei den Therapien zutreffen, denen eine nicht spezifische Wirkung auf die Krankheit, sondern eine symptombezogene Wirkung zur Verbesserung des therapeutischen Umfelds zugeschrieben wurde. Daraus ergab sich in dieser Zeit, als es zwar Bemühungen um eine Standardisierung der Diagnosen und ihrer Behandlung, aber noch keine allgemeine Übereinkunft darüber gab,[103] eine sehr unterschiedliche Praxis auf der Basis der verschiedenen Schulen und Traditionen, sowie unterschiedlicher individueller Erfahrungen und Einschätzungen. Nicht eine international geprüfte und anerkannte Therapie für eine ebenso in der internationalen Fachgemeinschaft fixierte Diagnose gab den Ausschlag, sondern die persönliche Einschätzung des Besten für den Patienten / die Patientin. Es war freilich ein System, das ein

[100] BLEULER, Lehrbuch der Psychiatrie ([9]1955), S. 161; BLEULER, Lehrbuch der Psychiatrie ([10]1960), S. 160; BLEULER, Lehrbuch der Psychiatrie ([11]1969), S. 165.
[101] Vgl. dazu oben S. 31. das Zitat aus BLEULER, Lehrbuch der Psychiatrie ([11]1969), S. 425.
[102] Siehe unten S. 188 und 236f.
[103] Es ist die Zeit vor einer breiteren Anerkennung des ICD-8 in den Jahren nach 1965 und noch lange vor den Therapie-Guidelines der 1980er und 1990er Jahre. Zu früheren Bemühungen in Deutschland, vgl. DÖRRIES, Der „Würzburger Schlüssel" von 1933 (1999), S. 188-205.

Abgehen von eingeführten Therapien aufgrund neuer Erkenntnisse erschweren bzw. verhindern konnte.

Kreuztabelle zur Summe und Relation der Diagnosen und der alten ‚großen' körperlichen Therapien 1951-1969:

	keine „Schock-therapie"	EKT	Insulin-koma- & Insulin-subkoma-therapie	Malaria-Kur	Typhus-Kur	Cardiazol krampf-therapie	Gesamt (Akten)
Intelligenzmängel	601	70	5	255	1	2	919
S-%|Z-%	7,0% | 64,3%	1,1%|7,5%	0,5%|0,5%	27,6%|27,3 %	5,6%|0,1%	1,7%|0,2 %	5,6%|100 %
Neurolues	194	18	2	163	12	1	371
S-%|Z-%	2,2% |49,7%	0,3%|4,6%	0,2%|0,5%	17,7%|41,8 %	66,7%|3,1 %	0,8%|0,3 %	2,3%|100 %
Schizophrene Erkr.	1896	3313	927	193	4	111	5640
S-%|Z-%	22,0%| 29,4%	50,3%|51,4 %	90,2%|14,4 %	20,9%|3,0 %	22,2%|0,1 %	93,3% | 1,7%	34,4%|100 %
Psychopathie	1188	36	10	205	1	0	1438
S-%|Z-%	13,8%|82,5%	0,5%|2,5%	1,0%|0,7%	22,2%|14,2 %	5,6%|0,1%	0,0%|0,0 %	8,8%|100 %
Affektive Erkr.	4756	3155	84	107	0	5	8036
S-%|Z-%	55,1%| 58,7%	47,9%|38,9 %	8,2%|1,0%	11,6%|1,3 %	0,0%|0,0%	4,2% |0,1%	49,0%|100 %
Gesamt<br>Gesamt (S-%|Z%)	8004<br>100% | 50,8%	5886<br>100% | 37,4%	965<br>100% | 6,1%	772<br>100% | 4,9%	18<br>100% | 0,1%	114<br>100% | 0,7%	14919

Tab. 1 Kreuztabelle Diagnosen und Koma-, Krampf- bzw. Fiebertherapien (1951-1969), Aufenthalt mehr als 4 Tage, n = 14.919 Aufnahmen, Mehrfachnennungen bei Diagnosen und Therapien sind möglich.

S-% gibt die Spaltenprozente und Z-% die Zeilenprozente an. Es wurde durch die Gesamtzahl der Diagnosen oder Therapien gerechnet und nicht der Akten oder PatientInnen, da es aufgrund der Mehrfachnennungen, sonst zu Werten von über 100% gekommen wäre. Auch Gesamt-S-% und Gesamt-Z-% beziehen sich auf alle Diagnosen und Therapien.

In der Kreuztabelle werden die Diagnosen und Koma-, Krampf- und Fiebertherapien[104] in ihrem Ausmaß und in ihrer Relation zueinander dargestellt. In den Gesamtspalten und -zeilen werden immer die Anzahl der Aufnahmeakten angegeben und nicht die PatientInnen, da es PatientInnen gibt, die im entsprechenden Zeitraum auch mehrmals aufgenommen wurden. Da jedoch nicht selten ein/e Patient/in bei einer Aufnahme mit mehr als einer Therapie behandelt oder mehr als eine Diagnose erhalten hat, gibt es mehr Therapien als die Zeilen-Gesamtzahl angibt, beziehungsweise mehr Diagnosen als die Spalten-Gesamtzahl angibt. So wurden mit den Einschluss-Kriterien (Diagnose und Aufenthaltsdauer) insgesamt 14.919 Fälle zwischen 1951 und 1969 aufgenommen. Das sind insgesamt 11.720 PatientInnen (3.199 Mehrfachaufnahmen, 21,4% aller aufgenommenen Fälle) mit 16.404 vergebenen Diagnosen und 7.755 durchgeführten Krampf-, Koma- oder Fieberkuren.

Die Datenbank enthält 6.915 Fälle[105] mit Krampf-, Koma- oder Fieberkuren, wobei aufgrund von Abfolgen mehrerer Krampf-, Koma- und Fieberkuren während eines Aufenthaltes und von Doppel- und Dreifachkombinationen insgesamt 7.755 Krampf-, Koma- und Fieberkuren gegeben wurden. Somit sind 46,3%, d. h. 6.915 von 14.919 der in der Datenbank aufgenommenen Akten, Fälle, in denen eine Krampf-, Koma- und / oder Fieberkur angewandt wurde; in 10,8% der Fälle wurden zwei oder mehrere Krampf-, Koma- und Fieberkuren – teils als Kombinations-Therapien[106] – gegeben.

Die Datenbank enthält 8.004 Fälle, in denen keine Krampf-, Koma- oder Fieberkur gegeben wurde: Geht man nur von den Diagnosen aus, zeigt sich, dass vor allem Schizophrene mit einer Krampf-, Koma- oder Fieberkur behandelt wurden; der Anteil der Schizophrenen, die keine Krampf-, Koma- oder Fieberkur erhalten haben, liegt bei 29,4% (Minimum unter den aufgenommenen Diagnosen). Umgekehrt haben 82,5% aller Psychopathie-PatientInnen keine Krampf-, Koma- oder Fieberkur erhalten (Maximum unter den aufgenommenen Diagnosen). Das ist freilich in Zusammenhang mit den unterschiedlichen Indikationen zu sehen, so wurden die Patienten mit junger Schizophrenie, wenn sie an der Klinik behandelt wurde, standardmäßig mit der in Wien als „spezifische“ Therapie eingeschätzten Insulinkomatherapie (und sehr häufig kombiniert mit EKT oder Cardiazolkrampf) behandelt, während bei

[104] Die wenigen Fälle einer Kur mit Typhus Vakzinen wurden als mildere Fiebertherapie aufgenommen.
[105] Hier sind alle Aufnahmen gezählt, also auch bei Mehrfachaufnahmen jede Aufnahme einer Patientin / eines Patienten mit einer Diagnose aus den fünf Diagnosefeldern und mit einem Aufenthalt von mehr als vier Tagen.
[106] Etwa die dem Insulinkoma ‚aufgesetzten‘ Elektro- oder Cardiazolkrampftherapien, die unten im Kapitel zur Insulinkomatherapie genannt werden.

Psychopathie nur manchmal die Malariafiebertherapie und EKT symptombezogen angewandt wurden.

Die Elektrokrampftherapie (EKT) ist die am häufigsten angewandte ‚alte' somatische Kur, vor allem bei PatientInnen mit schizophrenen Erkrankungen (3.313 PatientInnen) oder mit affektiven Erkrankungen (3.155 PatientInnen). Bei diesen Zahlen ist jedoch Vorsicht geboten und zu bedenken, dass 690 PatientInnen mit einer Legierungspsychose[107] sowohl in die Klasse der schizophrenen als auch in die Klasse der affektiven Erkrankungen eingeordnet und gezählt wurden.

Die Insulinkoma- und -subkomatherapie[108] ist unter den in der Datenbank aufgenommenen PatientInnen die zweithäufigste der ‚alten' körperlichen Kuren. Üblicherweise bekamen PatientInnen mit einer schizophrenen Erkrankung eine Insulinkomatherapie: 90,2% der Insulinkuren haben PatientInnen mit schizophrenen Erkrankungen bekommen, bzw. 14,4% aller an der Klinik mit diesem Krankheitsbild Behandelten (bzw. 6,1% aller in der Datenbank aufgenommenen PatientInnen). Von den 84 PatientInnen mit affektiven Erkrankungen hatten 51 PatientInnen eine Mischdiagnose (vor allem „Legierungspsychose"); sie bekamen die Komatherapie aufgrund ihrer schizophrenen Symptomatik. Von den restlichen 33 PatientInnen bekamen nachweislich mehr als die Hälfte eine Insulinsubkomatherapie.[109]

Die Malariafieberkur war bei den in der Datenbank aufgenommenen Diagnosen die dritthäufigste der an der Klinik angewandten ‚alten' Kuren. Knapp 4,9% aller in der Datenbank eingetragenen Aufnahmen (einschließlich der Mehraufnahmen von PatientInnen) bekamen eine Malariakur. Von den in der Datenbank eingetragenen Neurolues-PatientInnen bekam elf zweimal eine Malariatherapie.[110] Wenn man nur die 11.720 PatientInnen und nicht die Mehrfachaufnahmen einbezieht, steigt der Anteil der Malariakuren auf 6,9% aller aufgenommenen PatientInnen.

Auf den ersten Blick scheinen (anders als bei der EKT oder der Insulinkomatherapie) alle Diagnosen häufig vertreten zu sein.[111] Bei einer Kreuztabellen-Auswertung in vier

[107] Vgl. zur Diagnose unten S. 202 und S. 225-228 im Kapitel zur Insulinkoma- und -subkomatherapie.
[108] Sie wurden in der Datenbank nicht getrennt; vgl. unten das Kapitel zur Insulinkur.
[109] Siehe dazu im Insulinkapitel S. 225-229.
[110] Vgl. unten S. 72 (11 von 175); bei einigen von ihnen war die Malariakur unterbrochen worden.
[111] Die Farbmarkierungen nach dem Ampelprinzip in der Spalte Malariatherapie heben die Reihung der Häufigkeit der Diagnosen, die mit einer Malariatherapie behandelt wurden, hervor.

Jahresblöcken (3mal 5, 1mal 4 Jahre) im Projektendbericht[112] zeigt sich, dass die Malariafiebertherapien bei den Diagnosen nicht gleichmäßig verteilt waren, sondern sich im Verlauf des Untersuchungszeitraumes in ihrer Häufigkeitsverteilung deutlich veränderten. Während im ersten Block (1951-1955) den schizophrenen Erkrankungen 32,3% (127), der Neurolues 30,0% (118), den Intelligenzmängeln 22,6% (89), der Psychopathie 9,7% (38) und den affektiven Erkrankungen 5,3% (21 Anwendungen) der 329 Malariakuren zuzurechnen waren, kamen im letzten Block (1966-1969) auf schizophrene Erkrankungen 3,6% (3), Neurolues 6,0% (5), Intelligenzmängel 27,4% (23), Psychopathie 34,5% (29) und affektive Erkrankungen 28,6% (24) der nur mehr 69[113] durchgeführten Malariakuren. Wie hier im letzten Jahresblock, so wurde auch in der ersten Hälfte der 1960er Jahre die Malariafiebertherapie bei Psychopathie am häufigsten (in 83 von 185 Fällen, das sind 39,0%) angewandt (mit einer Steigerung in den 1950er Jahresblöcken von 9,7% / 38 von 329, auf 23,6% / 55 von 189 Anwendungen), während ihre Anwendung in den Jahresblöcken bei der Neurolues – ihrem ursprünglichen Hauptindikationsgebiet – in den vier Jahresblöcken von 30,0% (118), über 9,4 (22) und 8, 5 (18) auf 6,0% (5 Fälle) gefallen war.[114] Dazu wie auch insgesamt zur Abnahme der Anwendungen der Malariakur hatte freilich vor allem die radikale Abnahme der Neuroluesfälle durch die Frühbehandlung der Syphilis mit Penicillin beigetragen.

Bei den Zahlen zur Malariatherapie ist zu beachten, dass in dieser statistischen Auswertung nur 772 PatientInnen aufgelistet sind, in der Datenbank jedoch 869 PatientInnen mit einer Malariakur aufscheinen, da alle PatientInnen mit einer Malariakur unabhängig von Aufenthaltsdauer und Diagnose aufgenommen wurden. 97 PatientInnen wurden hier aus den Vergleichen herausgenommen; es sind PatientInnen die in den 1960er Jahren mit anderen als den fünf Auswahldiagnosen die Malariafiebertherapie bekamen (die also nicht den Einschlusskriterien entsprechen) und von denen es deshalb keine Vergleichszahl über die PatientInnen mit denselben Diagnosen, aber ohne Malariakur gibt. Nur in die gesonderte Auswertung aller Fälle mit Malariatherapie werden diese 97 PatientInnen einbezogen.[115]

Die Fieberkur mit Typhus Vakzinen alleine ist sehr selten: in der Datenbank sind 18 PatientInnen aufgenommen, die Typhus-Vakzine bekommen haben. Davon haben elf

---

[112] HEISS, Projektendbericht (2015), S. 131-142
[113] Wegen Mehrfachzählungen aufgrund von Doppeldiagnosen stimmt diese Zahl nicht mit der Summe der Zahlen in der Klammer überein.
[114] HEISS, Projektendbericht (2015), S. 141.
[115] Siehe unten S. 57f., S. 61f.

PatientInnen die Typhus-Impfung während einer Malariatherapie erhalten – vor allem zur Fieberprovokation, aber auch nach Problemen mit der Malariakur. In einem Fall gibt es eine EKT-Malaria-Typhus-Behandlung.[116] Lediglich sieben PatientInnen haben eine reine Typhus-Fieberkur durchgemacht, davon sechs männliche Patienten mit einer Neurolues (von vornherein statt einer Malariakur oder nach deren Abbruch wegen zu geringer Fieberschübe oder schlechter Verträglichkeit) und eine Patientin mit der Diagnose „Hebephrenie“: [117] Die 21-jährige Privatpatientin aus dem Ausland bekam 1964 zehn Tage nach einer EKT mit vier vollmitigierten Anwendungen die mildere Fiebertherapie mit acht Typhus Vakzine-Injektionen in Abständen von zwei bis drei Tagen. Es könnte dazu die unterschiedliche Indikation auf der Frauenabteilung beigetragen haben, wo nach der Datenbank nach 1964 keine und auch vorher kaum eine Patientin außerhalb der Neurolues eine Malariafiebertherapie bekam – ganz anders als die jungen männlichen Patienten bei diesen Diagnosen. In einer Kur mit Typhus-Vakzinen – das zeigen die Einträge in den Fieberkurven dieser sieben Fälle – wurden mit sehr unterschiedlichen Dosen von Typhus-Keimen (mit 100 Mill. bis 250 Mill., aber auch mit 150 Mill. bis 2.000 Mill. Keimen) am Tag der Injektion Fieberschübe hervorgerufen (insgesamt 4 bis 10mal), die unter 40° C blieben. Fieberkuren mit Typhus-Vakzinen wurden an der Wiener Klinik in den 1950er und 1960er Jahren auch bei anderen – teils neurologischen – Diagnosen gegeben, die nicht in die Datenbank aufgenommen wurden.

Die Cardiazolkrampftherapie war bei den in die Datenbank aufgenommenen PatientInnen die vierthäufigste der Krampf-, Koma- und Fieberkuren. Der Cardiazolkrampf wurde in den in

[116] Der 18jährige Patient S399 („Hebephrenie mit katatonen Exacerbationen“) bekam am 3., 6., 10. und 11. Tag nach der Aufnahme je eine EKT-Anwendung. Das überschnitt sich mit den ersten Tagen der Malariakur, da er schon am 8. Tag mit Malariablut geimpft wurde. Er bekam jedoch keine Fieberschübe, wurde am 10. Tag nach der Impfung zur Provokation mit Typhus Vakzinen geimpft und als auch das erfolglos blieb, wurde noch am gleichen Tag mit der Gabe von Chinin+Atebrin begonnen. Der Patient wurde nach Gugging überstellt.

[117] S18002. Im Befundbericht heißt es 1964: „Es handelt sich um eine Retardation unbekannter Genese im Verein mit einem M[orbus] Bleuler [Schizophrenie]. Die Pat. wurde einer E-Schockbehandlung sowie einer Truxal-Valium und Melleriltherapie und einer Fieberkur unterzogen, sowie mit anabolen Substanzen behandelt. Unter dieser Behandlung kam es zu einer recht guten Besserung. Die Patientin wurde nach Hause entlasssen und angewiesen weiterhin täglich 3 x 5 mg Valium und 3 x 1 Tabl. Encephabol weiterzunehmen und sich nach Ablauf von 3 Monaten zu einer neuerlichen stationären Behandlung wieder für ca. 2 Monate an unserer Klinik aufnehmen zu lassen.“ Beim 2., letzten und nur kurzen Aufenthalt zur „Kontrolluntersuchung“ 1965 heißt es im Befundbericht: „Diagnostisch handelt es sich bei der Patientin um einen schizophrenen Defekt mit Exacerbationen bei einer Retardation unbekannter Genese. […] Die Patientin erhielt therapeutisch täglich 3 x 1 Tablette Luvatren, 2 x 1 Tablette Kemadrin [gegen die Nebenwirkung des Neuroleptikums?] und abends 30 mg Valium. Diese Medikation soll die nächsten 6 Monate unverändert beibehalten werden“. Zu den Erfahrungen mit Luvatren: WEISER, Erfahrungen mit dem Butyrophenonpräparat Luvatren in der Behandlung der Schizophrenie (1968), S. 444-446.

der Datenbank aufgenommen Fällen fast nur in Kombination mit einer Insulinkomakur bei Schizophrenen, (ähnlich wie häufig auch die EKT) zur Verstärkung der Insulinkur „im Coma aufgesetzt“.[118] Da diese Kombination so deutlich überwiegt, wird die Cardiazolkrampftherapie nicht in einem eigenen Kapitel, sondern im Insulinkapitel behandelt.[119] Selten wurden auch die beiden Krampftherapien als „Cardiazol-Elektro-Schock“ gemeinsam gegeben.[120] Cardiazol in Dosen, um während einer Malariakur den Blutdruck zu stabilisieren, also nicht als Krampftherapie, wurde mehrmals gegeben, diese Fälle sind jedoch hier nicht einbezogen.[121] Die Anwendung ohne Insulinkoma oder Elektrokrampf von Cardiazol in hohen Dosen, wie sie der Krampftherapie entsprechen, gab es in der ‚Ära Hoff‘ vermutlich nur in einzelnen Fällen, so bei zwei Patientinnen nach einem Selbstmordversuch, die durch die Schmerzen der Krampfbehandlung aus dem Koma geweckt werden sollten.[122] Zu diesen beiden Patientinnen ist zu bemerken, dass die Cardiazolkrampftherapie nicht bei einer schizophrenen Erkrankung, ihrem ursprünglichen und Hauptanwendungsgebiet angewandt wurde;[123] ähnliche Ausweitungen des Anwendungsgebietes auf andere Diagnosefelder sind auch bei den anderen ‚großen‘ alten Kuren zu sehen und verweisen auf ihre Indikation bei bestimmten Symptomen und Symptomgruppen.

---

[118] Zu begründen ist diese Einschränkung der Anwendung damit, dass der / die PatientIn durch das Koma nicht unter den dem Schock vorangehenden quälenden Sensationen und Angstzustände leidet, deretwegen Max Müller bereits 1949 von der Cardiazolkur abrät (s. unten S. 251) und Sargant/Slater noch 1972 nur diese Kombination empfehlen (s. unten S. 216 Anm. 841). Vgl. dazu 1963 im deutschsprachigen Handbuch zur „Psychiatrie der Gegenwart“ Hugo SOLMS, Die Krampfbehandlung (1963), S. 463, wonach sich die Kombination der Insulinkomatherapie mit der Cardiazol- oder Elektrokrampftherapie „weitgehend eingebürgert“ habe. Zu den Anfängen der „Summation“ der Therapien im Insulinkoma vor 1950 vgl. GAWLICH, Eine Maschine, die wirkt (2018), S. 271f.

[119] Vgl. u. a. S. 215f.

[120] Vgl. unten S. 90 und im Insulinkapitel S. 215 Anm. 838.

[121] Drei (Fehl-)Einträge zur Kombination von Malariafiebertherapie und Caridazol dürften auf Cardiazol zur Stabilisierung des Blutdrucks zurückzuführen sein. Vgl. unten Anm. 166 das Zitat zu den Mischspritzen mit Cardiazol und Strychnin.

[122] Die 21jährige Patientin X12355 bekam 1956 mit der Diagnose „Neurose, SMV“ (Diagnose nicht in der statistischen Auswertung): Magenspülung (nach SMV mit Schlafmitteln) 13:30 10cc Cardiazol i. v., 300.000E Hypropen, 14h+15h+16.30 je 5cc Cardiazol i. m.; 3stdl. 100.000E Penicillin, 18.30+20.30+22.30 5cc Cardiazol, “ansprechbar”; am folgenden Tag 9:30 5cc Cardiazol; dann sind keine Medikamente mehr auf der Fieberkurve eingetragen; erst in den letzten 6 Tagen Arbeitstherapie. Die 35jährige, bei ihrer Aufnahme nicht ansprechbare Patientin A12351 mit der Diagnose „Depression, SMV“ bekam im selben Jahr (1956) nach einem SMV mit unbekannten Medikamenten (und nach der Magenspülung) bei der Aufnahme zweimal in einem Abstand von ca. 15 Minuten 10ccm Cardiazol i. v. (also eine Schockdosis); zur 2. Gabe heißt es: „noch einmal 10cc Cardiazol i.v hierauf Reaktion auf Schmerz. Pat. auch weiterhin schwer somnolent“. Außerdem bekam sie bei ihrem 7tägigen Aufenthalt (vor ihrer Überstellung auf den *Steinhof*): tgl. 1-2x 300.000E Hypropen i. m., 1 A Destrydon i. m. und an den letzten 3 Tage je 2A Largactil. Vgl. unten S. 90 zur Cardiazolkrampftherapie 1949 bei der Patientin S895 – also vor der ‚Ära Hoff‘.

[123] MICHAUX, Psychiatrie (1965), S. 1002, erwähnte, dass einige Psychiater den Cardiazol-‘Schock’ noch « dans les états psychopathiques aves déséquilibre important et conduites antisociales [was in Wien wohl als Psychopathie diagnostiziert wurde, GH], ainsi que dans certains états hypocondriaques en désespoir de cause » anwenden würden.

Häufig wurden bei einer Aufnahme auch zwei oder mehrere der ‚großen‘ körperlichen Therapien in einer Abfolge oder kombiniert (gleichzeitig) gegeben. Die folgende Tabelle zeigt die Anwendung mehrere Krampf-, Koma- und Fiebertherapien bei einer Aufnahme – gleichzeitig oder in einer Abfolge:

	EKT	Insulinkoma- & -subkoma-therapie	Malaria-Kur	Typhus-Kur	Cardiazol-krampf-therapie
EKT.	5887	657	54	2	80
Insulintherapien	657	965	10	0	101
Malaria-Kur	54	10	869	11	3
Typhus-Kur	2	0	11	18	0
Cardiazoltherapie	80	101	3	0	114

Tab. 2 Mehrere Fieber-, Koma- oder Krampftherapien in Abfolge oder Kombination (1951-1969), es sind auch Dreifachnennungen möglich.

Kombinationen der EKT waren jene (seltenen) als „Cardiazol-Elektro-Schock“ [124] und jene, in denen Elektrokrämpfe – gleich wie die Cardiazolkrampfanwendungen – dem Insulinkoma „aufgesetzt“ wurden. Häufig waren Anwendungen vor oder nach einer der anderen Kuren (meistens der Insulinkomatherapie[125]). In der überwiegenden Zahl der Fälle war die EKT aber die einzige der ‚großen‘ alten körperlichen Therapien.

In 68% aller Insulinkomatherapien (657 von 965 Fällen) wurde zusätzlich eine EKT gegeben (vor der Insulinkomatherapie oder während dieser dem Koma „aufgesetzt“). In 10,5% der Insulinkuren wurden während dieser einige Cardiazolkrämpfe dem Koma „aufgesetzt“.[126]

Die Malariakur wurde meist als „Einzelkur“ gegeben; in 6,2% der Malariakuren (54 von 869 PatientInnen) wurde jedoch vor oder nach der Fiebertherapie (für gewöhnlich vorher) eine EKT durchgeführt. In elf der Fälle wurden Typhus-Vakzine während der Malariafieberkur geimpft, um das Fieber zu steigern.

[124] Vgl. unten S. 90 und S. 215 Anm. 838.
[125] Die 80 Fälle mit Cardiazolkrämpfen sind Anwendungen der EKT vor einer Insulinkur, in der dann dem Koma ein Cardiazolkrampf „aufgesetzt“ wurde, oder die seltenen Fälle von „Cardiazol-Elektro-Schocks“.
[126] Vgl. oben S. 39 Anm. 118.

Typhus-Vakzine wurden in elf von 18 Fällen (über 60%) mit einer Malariakur kombiniert (meistens zur Fieberprovokation), in sieben von 18 Fällen alleine als Fieberkur gegeben.[127]

Die Cardiazolkrampftherapie wurde in den in der Datenbank aufgenommen Fällen fast ausschließlich in Kombination mit einer Insulinkomakur bei Schizophrenen zur Verstärkung des Komas eingesetzt („im Coma aufgesetzt"). In 80 von 114 Fällen wird der Cardiazolkrampf mit der Insulinkomakur nach einer Elektrokrampftherapie angewandt.

Das Geschlecht – Einfluss auf Diagnose und Therapie?

In der Zuordnung aller in die Datenbank aufgenommenen Fälle (A) bzw. der von diesen mit einer der Koma-, Krampf- und Fieberkuren behandelten Fälle (B) nach ihrem Geschlecht zu einer der fünf Diagnosen soll dargelegt werden, wie sich Diagnose und Therapie auf die Geschlechter (Mann/Frau) verteilen.

A) Alle in der Datenbank eingetragenen PatientInnen-Aufnahmen (Fälle) verteilt auf Diagnose und Geschlecht:

	Männlich	Weiblich	Gesamt
	Anzahl (Fälle)	Anzahl (Fälle)	Anzahl (Fälle)
Intelligenzmängel	596	323	919
S-%|Z-%[128]	8,4% | 64,9%	3,5% | 35,1%	5,6% | 100%
Neurolues	231	140	371
S-%|Z-%	3,3% | 62,3%	1,5% | 37,7%	2,3% | 100%
Schizophrene Erkr.	2572	3068	5640
S-%|Z-%	36,3% | 45,6%	33,0% | 54,4%	34,4% | 100%
Psychopathie	868	570	1438
S-%|Z-%	12,3% | 60,4%	6,1% | 39,6%	8,8% | 100%
Affektive Erkr.	2814	5222	8036
S-%|Z-%	39,7% | 35,0%	56,0% | 65,0%	49,0% | 100%
Gesamt (Akten)	6336<br>100% | 43,2%	8583<br>100% | 56,8%	14919

Tab. 3 Alle Fälle nach Diagnosen und Geschlecht (1951-1969), n = 14.919 Aufnahmen, Mehrfachnennungen bei Diagnosen sind möglich.

[127] Vgl. oben S. 37f.
[128] Zu diesen Kürzeln vgl. oben S. 34 zu Tabelle 1.

Unter den in der Datenbank nach den Einschlusskriterien eingetragenen PatientInnen-Aufnahmen (Fällen) gibt es insgesamt mehr Frauen als Männer (ca. 57% : 43%). Bei der Zuordnung der Diagnosen zeigt sich, dass Frauen weitaus häufiger mit affektiven Störungen diagnostiziert wurden (65% : 35%). Demgegenüber weisen mehr als 60% der männlichen Patienten die Diagnosen „Intelligenzmängel", „Neurolues" oder „Psychopathie" auf.

B) Alle in der Datenbank eingetragenen PatientInnen-Aufnahmen (Fälle) mit einer Koma- Krampf- und / oder Fiebertherapie verteilt auf Diagnosen und Geschlecht:

	Männlich	Weiblich	Gesamt
	Anzahl (Fälle)	Anzahl (Fälle)	Anzahl (Fälle)
Intelligenzmängel	283	35	318
S-%|Z-%[129]	8,3% | 89,0%	0,8% | 11,0%	4,1% | 100%
Neurolues	118	59	177
S-%|Z-%	3,5% | 66,5%	1,3% | 33,5%	2,3% | 100%
Schizophrene Erkr.	1605	2139	3744
S-%|Z-%	47,3% | 42,9%	48,9% |57,1%	48,2% | 100%
Psychopathie	232	18	250
S-%|Z-%	6,8% |92,8%	0,4% | 7,2%	3,2% | 100%
Affektive Erkr.	1157	2123	3280
S-%|Z-%	34,1% | 35,2%	48,5% |64,8%	42,2% | 100%
Gesamt (Akten)	2976<br>100% | 43,7%	3939<br>100% | 56,3%	6915

Tab. 4 Nur Fälle mit Koma-, Krampf- oder Fiebertherapien: Geschlecht und Diagnosen, n = 6.915 Aufnahmen, Mehrfachnennungen bei Diagnosen sind möglich.

Der Tabelle 4 ist die Häufigkeit der mit (einer oder mehreren) Koma-, Krampf- und Fiebertherapien behandelten PatientInnen nach Diagnose und Geschlecht zu entnehmen; dabei geht es wiederum um die Zahl der PatientInnen-Aufnahmen, d. h. es wird auch jede Mehrfachaufnahme gezählt, wenn sie den Aufnahmekriterien in die Datenbank entspricht. Im Vergleich zu den Zahlen aller Eintragungen von Aufnahmen im betreffenden Diagnosefeld, zeigen sich hier starke Unterschiede zur Zahl der Fälle, die jeweils mit ‚großen' alten Kuren behandelt wurden: bei Patientinnen mit Diagnosen aus dem Feld der Intelligenzmängel wurden von 919 Aufnahmen in 318 Fällen (34,6%) eine oder mehrere Kuren angewandt, bei PatienInnen mit Neurolues erfolgte diese Behandlung in 177 (47,978%) von 371 Fällen (vor

[129] Zu diesen Kürzeln vgl. oben S. 34 zu Tabelle 1.

allem PP), bei PatienInnen mit schizophrenen Erkrankungen in 3.744 (66,38%) von 5.640 Fällen, bei der Diagnose Psychopathie in 250 (17,38%) von 1.438 Fällen und bei affektiven Störungen in 3.280 (40,81%) von 8.036 Fällen. Die Verhältniszahlen der aufgenommenen männlichen bzw. weiblichen Patienten zu jenen der mit einer der ‚großen' alten Kuren behandelten sind mit einer Abweichung von 2% bis 4% bei den NeuroluespatientInnen mit oder ohne Kur ähnlich, bei den männlichen bzw. weiblichen Patienten mit Diagnosen aus dem Feld der Intelligenzmängel und besonders mit jenen mit der Diagnose Psychopathie sehr unterschiedlich: während die Aufnahmen mit ‚Intelligenzmängeln' auf männliche bzw. weibliche PatientInnen im Verhältnis von 64,9% : 35,1% war, war die Verteilung der mit dieser Diagnose mit ‚großen' alten Kuren Behandelten 89,0% zu 11,0%. Bei der Psychopathie waren die Aufnahmen mit dieser Diagnose auf männliche bzw. weibliche PatientInnen mit 60,4% : 39,6%, die Fälle mit einer oder mehreren Kuren mit 92,8% : 7,2% verteilt. Die Frage nach dem Grund der unausgeglichenen Verteilung der Kur-Behandlung auf die Geschlechter wird noch im besonders deutlichen Fall der Psychopathie gestellt, kann aber nach unseren Quellen nicht beantwortet werden.[130]

Soziale Parameter

A) Stichprobe aus 1955 - 1960

Als soziale Indikatoren zur Frage nach der Bedeutung der sozialen Herkunft für die Wahl einer Therapie wurden der Beruf des Patienten / der Patientin und die Behandlung in der Privatstation gewählt. Beides wurde nur in der Stichprobe zur Pilotstudie (1955-1960) und bei der vollständigen Aufnahme aller Akten der männlichen Patienten von März bis September 1964 aufgenommen. Als zwei Berufe, die als Indikator für eine unterschiedliche soziale Schicht des Patienten / der Patientin dienen konnten, eigneten sich die Kategorien „Angestellte/r" und „Hilfsarbeiter/in"; diese Berufe eigneten sich auch deshalb, da sie in den PatientInnenakten häufig vorkommen. Zur Frage wird auf die repräsentative Stichprobe der Pilotstudie (Akten 1955-1960) zurückgegriffen, da nur in ihrem Rahmen die beiden Kriterien, mit denen eine Annäherung versucht wird, vollständig erhoben wurden.

a) PatientInnen auf der Privatstation B22 (Stichprobe 1955-1960)

[130] Vgl. unten S. 62f. und S. 164 Anm. 606.

Im Rahmen der Datenauswertung für die Pilotstudie zeigte sich die Bedeutsamkeit der sozialen Herkunft der mit Malariatherapie behandelten PatientInnen. Von den 45 PatientInnen mit ‚großen alten Kuren' in der Stichprobe, die auf der Privatstation B22 untergebracht waren, bekam im Untersuchungszeitraum 1955-1960 keine/r eine Malariatherapie; von den 480 PatientInnen der Stichprobe, untergebracht auf den allgemeinen Stationen, bekamen jedoch 25 PatientInnen eine Malariatherapie. Hingegen erhielten PatientInnen, die auf der Privatstation B22 lagen, viel häufiger eine Elektrokrampf- oder eine Insulinkomatherapie (39 von 45 PatientInnen, ≈ 86,7 % der PrivatpatientInnen, aber nur ≈ 47,1% der PatientInnen auf den allgemeinen Stationen). PatientInnen der Privatstation hatten zwar Diagnosen, die bei PatientInnen der anderen Stationen zur Malariatherapie führen konnten – so wurden bei den PrivatpatientInnen der Stichprobe 22-mal eine Schizophrenie, 1-mal eine „Debilität", 1-mal eine „Psychopathie" und 1-mal eine Pfropfhebephrenie diagnostiziert –, dennoch wurde bei keinem/er einzigen dieser PatientInnen eine Malariatherapie angewandt.[131]

		Station		
		Nicht B22 (allgemeine Station)	B22	Gesamt
		Anzahl	Anzahl	Anzahl
Therapien	EKT (Fälle)	193	36	229
	Insulinkur (Fälle)	28	13	41
	Malariakur (Fälle)	25	0	25
	Cardiazolkrampfth. (Fälle)	11	1	12
	Keine „Schocktherapien" (Fälle)	254	6	260
	Gesamt „Schocktherapien" (PatientInnen)	226	39	265
	Alle PatientInnen	480	45	525

Tab. 5 Anzahl der Koma-, Krampf- und Fiebertherapien, aufgeteilt nach Station in der Stichprobe

[131] In der Gesamtauswertung gibt es zwar einzelne Malariafiebertherapien bei PrivatpatientInnen mit diesen Diagnosen, die Zahl ist jedoch so gering, dass sich der Gegensatz nicht ändert. Die PatientInnenakten der Kinderstation ergeben ein anderes Bild: Hier waren es vor allem PrivatpatientInnen aus dem Ausland, die wegen verschiedener Entwicklungsrückstände oder Cerebralschädigungen mit Malariatherapie behandelt wurden. Vgl. unten S. 92.

In der Stichprobe bekamen etwa 28,9% der PatientInnen auf B22, aber nur ≈ 5,8% der PatientInnen auf den allgemeinen Stationen eine Insulinkur. Damit lag die Wahrscheinlichkeit in den Jahren 1955-1960 mit einer Insulinkur behandelt zu werden fast fünfmal höher, wenn der/die PatientIn auf der Privatstation lag.

Werden nur die schizophrenen PatientInnen der Stichprobe betrachtet, wird die obige Aussage zwar etwas abgemildert, bleibt jedoch signifikant. So haben ≈ 54,5% der schizophrenen PatientInnen auf B22 eine Insulinkur erhalten, hingegen nur ≈ 17,0% der schizophrenen PatientInnen der allgemeinen Erwachsenenabteilungen; das heißt, die Wahrscheinlichkeit war mehr als dreimal so hoch, dass man mit einer Insulinkoma-Kur behandelt wurde, wenn man als schizophrene/r Patient/in auf der Privatstation und nicht auf den beiden allgemeinen Erwachsenenabteilungen lag. Dafür lassen sich einige Hypothesen formulieren:

- Da die Insulinkomatherapie eine längere Aufenthaltsdauer benötigt, dürfte die Wahl der Therapie auch von wirtschaftlichen Überlegungen abhängig gewesen sein. Arnold bestätigt in einem Artikel 1954 diese Vermutung: „Die praktische Indikation allerdings wird immer den ökonomischen Aufwand der IS [Insulinschocktherapie] in Betracht ziehen müssen, so daß die engere Auswahl der Fälle auch von den verfügbaren Möglichkeiten (Bettenanzahl) abhängig gemacht werden muß.“[132]
- Für die Häufigkeit der Insulinkomatherapie bei PatientInnen der Privatstation – im Unterschied zu jener in den allgemeinen Stationen – dürfte auch die Wahl der Wiener Klinik als Insulinkomatherapie-Spezialistin[133] eine Rolle gespielt haben. Nur 19 der 45 PrivatpatientInnen der Stichprobe hatten ihren Wohnsitz in Österreich, von den verbleibenden 26 PatientInnen kamen zehn aus dem arabischen Raum (Ägypten, Irak, Kuwait, Saudi-Arabien) und sechs aus Griechenland. Ausländische PatientInnen, vor allem aus arabischen Ländern, haben sich einer Insulinkur unterzogen, und zwar höchstwahrscheinlich auf eigenes Verlangen, auf das der Eltern bzw. auf Empfehlung ihrer Ärzte. Bei PrivatpatientInnen an der Kinderstation zeigt sich ein ähnliches Bild,

[132] ARNOLD, Schockbehandlungen (1954), Teil II, S. 71. Die Einschränkungen in der Verfügbarkeit des Insulins, wie in der Kriegs- und Nachkriegszeit, dürften in der ‚Ära Hoff‘ keine Rolle gespielt haben.
[133] ARNOLD, Die körperlichen Behandlungsmethoden der Schizophrenie (1960), S. 265 betont die herausragende Qualität der Insulinkomatherapie an der Wiener Klinik, vgl. unten im Insulinkapitel S. 190 und S. 193-195. Vgl. bereits ausführlich zum Problem: ZEITLHOFER/TSCHABITSCHER/WANKO, Zur Pathologie des protrahierten Insulinschocks (1954), S. 445-458.

indem sie auch dort eher als andere PatientInnen eine der aufwendigen ‚großen' körperlichen Therapien bekamen, ganz anders als bei den Jugendlichen und Erwachsenen war jedoch dort die Malariafiebertherapie bei PrivatpatientInnen mit „Cerebralschäden" aus den oben genannten Ländern besonders häufig.[134]

b) Berufe (Stichprobe 1955-1960)

In der Stichprobe zeigt sich ein ähnliches Ergebnis, wenn der Beruf für eine Annäherung an die Beantwortung der Frage herangezogen wird, ob die soziale Herkunft bei der Wahl der Therapie eine Rolle spielte. Die einzigen beiden größeren Gruppen, die auf einen Unterschied in der sozialen Schichtzugehörigkeit hinweisen können, waren die Gruppe der Angestellten und die der HilfsarbeiterInnen. Vergleicht man die beiden Gruppen bezüglich der Schock- und Fiebertherapien, so lässt sich die Hypothese formulieren, dass es zwar keinen Unterschied in der Häufigkeit der Anwendung von ‚alten Kuren' gibt, dass jedoch ein Unterschied in der Art der Therapie bestand. So haben Angestellte eher eine Insulin- oder eine Elektroschocktherapie erhalten, wohingegen HilfsarbeiterInnen eher mit einer Malariakur behandelt wurden. Genauer gesagt haben in der Stichprobe sieben Hilfsarbeiter (alle männlich) eine Malariakur erhalten, aber kein/e einzige/r Angestellte/r. Zu erwähnen ist hier, dass 35 Angestellte und 43 HilfsarbeiterInnen in der Stichprobe sind, davon erhielten 18 HilfsarbeiterInnen sowie 19 Angestellte eine Schock- oder Fiebertherapie. Die Diagnosen waren zwischen den HilfsarbeiterInnen und den Angestellten in etwa gleich verteilt, mit Ausnahme von den Erkrankungen mit Intelligenzmängeln (sieben HilfsarbeiterInnen, aber kein Angestellter). Bei einer weiteren Analyse zeigt sich, dass nur zwei der sieben HilfsarbeiterInnen, die eine Malariakur erhalten haben, auch mit einer „Debilität" diagnostiziert wurden. Die anderen fünf HilfsarbeiterInnen weisen demnach ähnliche Diagnosen auf, die auch etwa in gleicher Häufigkeit bei Angestellten auftraten. Die Frage stellt sich also, warum diese fünf Patienten mit einer Malariakur behandelt wurden, aber kein/e Angestellte/r mit ähnlichen Diagnosen.[135]

---

[134] Vgl. unten S. 92.

[135] Das Geschlecht spielt hierbei eine untergeordnete Rolle, so gibt es zwar unter den Hilfsarbeitern etwas mehr Männer als unter den Angestellten, jedoch hat von den 13 männlichen Angestellten (sieben davon mit Schizophrenie) keiner eine Malariakur erhalten. Überdies ist der Insulinschock in der gesamten Stichprobe über die Geschlechter hinweg durchschnittlich verteilt, jedoch haben Angestellte vergleichsweise häufiger Insulinschocktherapien erhalten. Überdies stellt die Schizophrenie für den Insulinschock die Hauptindikation dar. Diese Erkrankung ist jedoch auch durchschnittlich verteilt: Von den schizophrenen Hilfsarbeitern sind sechs

		Therapien				
		Therapie 1: Elektrokrampf	Therapie 2: Insulinkoma	Therapie 3: Malariafieber	Therapie 4: Cardiazolkrampf	Gesamt
Beruf-Code	Angestellte*r	17	5	0	0	19
	Hilfsarbeiter*in	12	1	7	1	18
	Rest	200	35	18	11	228
	Gesamt	229	41	25	12	265

Tab. 6 Verhältnis zwischen den Koma-, Krampf- und Fiebertherapien und dem Beruf der PatientInnen

Die Zahl der Fälle ist statistisch signifikant, wenn auch nicht sehr groß.

B) Männerabteilung, März bis September 1964

a) Berufe

In die Auswertung wurden die Fälle von Soldaten (Rekruten) hereingenommen, da in den 1960er Jahren die Rekruten, die eine Malariatherapie erhielten, auffielen. Sie sind nur in der statistischen Auswertung enthalten und nicht in den Bemerkungen dazu, da sie keiner sozialen Gruppe zuzurechnen sind und keine Schlussfolgerung in Bezug auf eine soziale Komponente in der Therapievergabe zulassen.

	**Hilfsarbeiter**	**Angestellte**	**Soldaten**	**Andere**	**Gesamt**
	**Anzahl (Fälle)**	**Anzahl (Fälle)**	**Anzahl (Fälle)**	**Anzahl (Fälle)**	**Anzahl (Fälle)**
**keine „Schock-therapie“**	83	29	17	445	574
**EKT**	3	11	3	37	54
**Insulinkoma-therapie**	1	3	0	6	10
**Malariakur**	4	0	4	12	20
**Gesamt**	90	40	24	495	649

Tab. 7 Berufe und Koma-, Krampf- und Fiebertherapien, Männer März-September 1964, n = 649.

männliche und acht weibliche Patienten, von den schizophrenen Angestellten sind sieben männliche und sieben weibliche Patienten (also sehr ausgeglichene Verhältniszahlen) in der Stichprobe.

Auch hier fällt im Vergleich der Hilfsarbeiter mit den Angestellten auf, dass es einen Unterschied in der Anwendung der Insulinkomatherapie und der Malariatherapie zwischen den beiden Gruppen gibt. So hat kein Angestellter eine Malariakur erhalten, hingegen 4 Hilfsarbeiter, während umgekehrt nur 1 Hilfsarbeiter eine Insulinkomatherapie erhalten hat, jedoch 3 Angestellte. Aufgrund der geringen Zahl der Fälle kann diese Verteilung freilich nicht als statistische Bestätigung der Ergebnisse aus der repräsentativen Stichprobe gelten.

Wesentlich für die Beurteilung dieser Werte ist, ob die Diagnosen unter den betrachteten Gruppen annähernd gleich verteilt sind.

	**Hilfsarbeite r**	**Angestellte**	**Soldaten**	**Andere**	**Gesamt**
	**Anzahl (Fälle)**	**Anzahl (Fälle)**	**Anzahl (Fälle)**	**Anzahl (Fälle)**	**Anzahl (Fälle)**
**Intelligenz-mängel**	18	0	1	23	42
**Neurolues**	1	0	0	4	5
**Schzophr. Erkr.**	10	16	5	77	108
**Psycho-pathie**	25	2	12	49	88
**Affekt. Erkr.**	3	12	4	79	98
**Alkoholis-mus**	37	6	4	183	230
**Neurose**	0	3	1	33	37
**Gesamt**	78	33	22	388	521[136]

Tab. 8 Berufe und Diagnosen, Männer März-September 1964, n = 521.

Die Unterschiede in der Therapie lassen sich nicht nur durch die soziale Schicht, sondern auch durch die Diagnosen erklären. So haben 25 Hilfsarbeiter eine Psychopathie-Diagnose, hingegen nur zwei Angestellte. Die Psychopathie war in den 1960er Jahren eine der hauptsächlichen Indikationen für eine Malariatherapie.

[136] Hier sind nur 521 der 649 Fälle von Männern zwischen März und September 1964 aufgelistet, da in den verbleibenden 128 Fällen die Diagnosen zu keinen der angeführten 7 Diagnosen zugeordnet werden konnte.

b) PatientInnen der Privatstation B22

	B22	Nicht-B22	Gesamt
**keine „Schocktherapie“**	26	548	574
**EKT**	6	48	54
**Insulinkomatherapie**	1	9	10
**Malariatherapie**	0	20	20
**Typhus-Therapie**	0	1	1
**Cardiazolkrampftherapie**	0	0	0
**Gesamt (Akten)**	32	618	650

Tab. 9 B22 und Therapien, Männer März-September 1964, Mehrfachtherapien sind möglich, n = 650.

	B22	Nicht-B22	Gesamt
	Anzahl (Fälle)	Anzahl (Fälle)	Anzahl (Fälle)
**Intelligenzmängel**	1	41	42
**Neurolues**	0	5	5
**Schizophrene Erkrankungen**	7	101	108
**Psychopathie**	0	88	88
**Affektive Erkrankungen**	4	94	98
**Neurotische Erkrankungen**	14	23	37
**Alkoholismus**	4	226	230
**Diagnose nicht klassifizierbar**	6	305	311
**Gesamt (Akten)**	32	618	650

Tab. 10 B22 und Diagnosen, Männer März-September 1964, Mehrfachtherapien sind möglich, n = 650.

Bei diesem Vergleich der Therapien und Diagnosen der Patienten der Privatstation B22 mit jenen der allgemeinen Männerstation wurden die Akten der Männerstation von März bis September 1964 vollständig aufgenommen, d.h. nicht nur Patienten mit den fünf Diagnosen. Anders als in der Stichprobe aus den Jahren 1955 bis 1960scheinen viel mehr Patienten der allgemeinen Station eine Insulinkomatherapie bekommen zu haben; wie in der Stichprobe gab

es bei Patienten der Privatstation[137] wieder keine Malariatherapie, sie wurde in diesen Monaten nur Patienten auf der allgemeinen Abteilung gegeben.

2.1 Die Malariafiebertherapie

2.1.1 Geschichte und Diskussion des Wirkmechanismus

Die Malariafiebertherapie ist die erste der in der Zwischenkriegszeit an den psychiatrischen Kliniken weltweit eingeführten ‚großen' körperlichen Kuren. In ihrer ursprünglichen Indikation bei Neurolues unterscheidet sie sich von den anderen ‚großen' körperlichen Kuren in ihrer als spezifisch geltenden Wirkung gegen den Krankheitserreger. Sie wurde jedoch bereits in den 1920er Jahren auch als unspezifisch wirkende „Schock-" – bzw. „Erschütterungstherapie" angewandt.

Julius Wagner-Jauregg hat seit den 1880er Jahren psychiatrische Erkrankungen mit verschiedenen Fiebertherapien behandelt. Er griff dabei die alte Meinung von der heilenden Wirkung des Fiebers auf.[138] In seinen Bemerkungen zu den 1936 edierten älteren Aufsätzen schildert er den Weg über die Behandlung von Psychosen allgemein mit künstlichem Fieber zur Behandlung der progressiven Paralyse mit Tuberkulin und Quecksilber, dann mit Typhus Vakzinen und Quecksilber bzw. Salvarsan.[139] Ende des Ersten Weltkrieges entwickelte er schließlich die Behandlung der progressiven Paralyse mit der Malariafiebertherapie (weiterhin kombiniert mit einer Vor- oder Nachbehandlung mit Salvarsan).[140] 1927 bekam er für die Malariafiebertherapie der progressiven Paralyse den Nobelpreis für Medizin.

Der Heilmechanismus, die Wirkweise der Malariafiebertherapie bei der progressiven Paralyse wurde von Anfang an kontrovers diskutiert. Joel T. Braslow nennt 1999 im historischen Rückblick einige Vertreter – alle aus der Zwischenkriegszeit – für zwei Positionen: Die einen vermuteten, dass das Fieber den Syphiliserreger direkt zerstöre, da das Treponema pallidum

---

[137] Ihr Status als Privatpatienten ist aus dem Akt meistens auch dann ersichtlich, auch wenn Sie für die Malariatherapie in das entsprechende Zimmer auf der allgemeinen Station B2 verlegt wurden.
[138] Zur heilenden Wirkung von Fieber wird Parmenides (520/515-460/455 v.Chr.) zitiert: „Gib mir die Macht, Fieber zu erzeugen, und ich heile jede Krankheit!".
[139] Bemerkungen Wagner-Jaureggs im Sammelband von 1936 zum Aufsatz von 1918/19 WAGNER-JAUREGG, Über die Einwirkung der Malaria auf die Progressive Paralyse (1918/19), S. 133-138.
[140] Einen detailreichen Überblick bietet KAUDERS, Zur Klinik, Theorie und Geschichte der Malariabehandlung (1948), S. 47-71. Zu neueren Literaturhinweisen vgl. KRAGH, Malaria, Sulfosin and Metallosal in the Treatment of Mental Disorders in Denmark (2013), S. 100.

(Bakterium aus der Familie der Spirochäten) sehr hitzeempfindlich ist – das Fieber also antibakteriell wirke. Die anderen waren der Meinung, dass durch die Malariakur eine unspezifische Immunantwort ausgelöst würde, die den Körper nun selbst in der Lage versetzt, die Krankheitserreger effektiv zu bekämpfen.[141] Die zweite Theorie, die einen immunologischen Mechanismus annahm, gewann im Verlauf immer mehr Zuspruch, auch in Kombination mit der Theorie, die dem antibakteriellen Mechanismus weiterhin eine Bedeutung beimaß. Auch Wagner-Jauregg war in den 1930er Jahren der Meinung, dass die Malariafiebertherapie durch die erhöhte Temperatur zwar die Spirochäten schwächen würde, dass jedoch die Aktivierung der Abwehr- und Immunkräfte die Hauptursache des therapeutischen Effekts sei.[142] Das blieb die Meinung der Wiener Klinik auch nach 1945.[143]

Die Annahme einer nicht direkt auf die Krankheitserreger, sondern zur Aktivierung der Abwehrkräfte im Patienten ausgerichteten Wirkung führte dazu, dass auch andere Methoden zur Anregung und Stärkung der Immunkräfte versucht und diskutiert wurden. Fiebertherapien wurden allgemein bei verschiedenen Krankheiten in den 1930er und 1940er Jahren häufig angewandt[144] und hatten im deutschen Sprachraum im Internisten Ferdinand Hoff damals und auch noch in den 1950er Jahren einen prominenten Befürworter.[145] Die Anwendung von

---

[141] BRASLOW, History and Evidence-Based Medicine: Lessons from the History of Somatic Treatments from the 1900s to the 1950s (1999), S. 231-240.

[142] WAGNER-JAUREGG, Der Mechanismus der Wirkung der Infektions- und Fiebertherapie (1935), S. 282f. Vgl. KAUDERS (Zur Klinik, Theorie und Geschichte der Malariabehandlung (1948), S. 58), der die verschiedenen experimentellen Forschungen zur Wirkung der Malaria auf die Syphilis beschrieb.

[143] Vgl. HOFF, Lehrbuch der Psychiatrie (1956), S. 102: Nachdem „nach vielen Jahren beide Abwehrmechanismen sowohl des Retiko-Endothels als auch des ektodermalen Gewebes erschöpft sind“ und „nun erst […] die Möglichkeit gegeben [ist], daß die Spirochäte in das Gehirn eindringt, um dort die spezifischen paralytischen Veränderungen zu machen“, seien sowohl Salvarsan als auch Schwermetalle „wirkungslos und nur die Malariatherapie, welche durch Fieber eine Reizung der Retikuloendothels und eine neue Entfachung seiner Abwehrmechanismen bezweckt, hat Erfolg.“ Das Lehrbuch ist in „Vorlesungen“ gegliedert, und dieses Zitat stammt aus der Vorlesung „Progressive Paralyse“.

[144] Viele verschiedene Fieberkuren wurden angewandt: Vgl. unten S. 78, die Zitate im Text zu den französischen Autoren Claude/Rubinovitch und Michaux. WEINGARTEN, Die derzeitige Therapie der Neurolues an der Wiener Nervenklinik (1952), S. 955, nennt: Rekurrens, Sodoku, Rattenbissfieber, pyrogene Lipopolysaccharide (Pyrexal), Schwefel, Terpentinöl und – 1952 noch häufig in den USA – Fiebererzeugung durch Kurzwellendurchstrahlung.

[145] Ferdinand HOFF, Fieber – unspezifische Abwehrvorgänge – unspezifische Therapie (Stuttgart 1957), S. 132: „Wie wir gesehen haben, vermag die Fiebertherapie so unterschiedliche Krankheiten wie Progressive Paralyse, Encephalitis, postdiphtherische Polyneuritis, Asthma bronchiale zu heilen oder den Typhuskranken zur Entfieberung zu bringen. Ein besonders eindrucksvolles Beispiel eines Stoßes ins vegetative System ist auch die Schocktherapie durch Elektroschock, Cardiazolschock und Insulinschock […]. Bekanntlich kann hierdurch eine Umstimmung auch bei schweren psychischen Störungen zustande kommen.“ Das hier zitierte Buch von Ferdinand Hoff erschien 1957 als eine Zusammenfassung seiner These, dass es bei Fieber und Fieberbehandlungen zu einer ‚vegetativen Gesamtumschaltung‘ käme, die das eigentliche Wirkprinzip dieser „unspezifischen Therapie“ sei. Der Großteil seiner Studien zu unterschiedlichen Veränderungen während des Fiebers gehen auf die 1930er und 1940er Jahre zurück. Ferdinand Hoff (1896-1988) war u. a. 1941-45 Professor in Graz und ab 1951 (gegen den Einspruch von Max Horkheimer wegen F. Hoffs Aktivitäten im

Pyrifer[146] (inaktivierte E. coli-Stämme) war allgemein verbreitet, ebenso die Injektion von Typhus Vakzinen zur Fieberprovokation.[147] Wenn die Malariafiebertherapie kontraindiziert oder malariainfiziertes Blut nicht vorhanden war, wurden auch nach der Einführung der Malariakur bei der progressiven Paralyse weiterhin andere Formen künstlichen Fiebers mit einer Salvarsan Nachbehandlung angewandt. Noch in der ‚Ära Hoff' wurden an der Wiener Klinik nach den vorliegenden PatientInnenakten Typhus Vakzine in sieben Fällen als Fieberkur alternativ statt Malaria geimpft, häufiger freilich während einer Malariafieberkur, wenn das Fieber nicht genug anstieg.[148]

Als Alternativen zur Malariakur wurden Anfang der 1950er Jahre – also bereits in der Zeit der Penicillinbehandlung – in Kopenhagen die Hypertherm-Behandlung[149] oder in Wien durch den Psychiater Jaromir Lhotský eine hormonelle Behandlung der progressiven Paralyse mit Thyroxin (Schilddrüsenhormon) vorgeschlagen. Für Lhotský, der sich auf Ferdinand Hoff bezog, wirkte auch jede „anderen Infektions- und Reiztherapie", auch die Elektropyrexie nach dem gleichen Grundprinzip der „Umstimmung des Organismus" und der „Mobilisierung der Abwehrkräfte im reticuloendothelialen System", der „vegetative[n] Gesamtumschaltung also".[150]

Nationalsozialismus) Professor in Frankfurt a. M. http://de.wikipedia.org/wiki/Ferdinand_Hoff_%28Mediziner%29 (18.3.2015).

146 Die Fiebertherapie mit Pyrifer wurde Ende der 1920er Jahre an der *Heil- und Pflegeanstalt Steinhof* sehr breit angewandt (vgl. unten S. 77 Anm. 251) und nach den vorliegenden PatientInnenakten in einigen Fällen auch noch in den 1950er Jahren an der Klinik. Otto Kauders publizierte bereits 1929 eine Studie, nach der die Malariafiebertherapie bei PP bessere Ergebnisse habe, als die Pyrifer-Kur: Otto KAUDERS, Erfahrungen mit Pyrifer bei der Behandlung der progressiven Paralyse (1929), S. 1262-1264.

147 WAGNER-JAUREGG, Über die Einwirkung der fieberhaften Erkrankungen auf Psychosen, 1887, S. 13-20, hatte schon 1887 von psychiatrischen Verbesserungen bei Typhus-Infektion berichtet und ab 1911 mit Typhus-Vakzinen experimentiert, also sechs Jahre vor der Verwendung der Malaria. Vgl. KOHL, Wagner von Jauregg (1993), S. 158. Vgl. HOFF, Lehrbuch der Psychiatrie (1956), S. 125 (Vorlesung: Progressive Paralyse): „Bei schlechtem Allgemeinbefinden des Patienten muß manchmal die Malaria nach 4 Fieberstößen unterbrochen, um nach einigen Wochen wieder aufgenommen zu werden. Geht die Malaria das zweite Mal nicht mehr an, so muß man sich mit künstlichem Fieber, das durch Typhusvakzine oder Pyrifer u. ä. Mittel erzeugt wird, begnügen. Die Impfung mit Rekurrens, Sodoku oder Rattenbißfieber wird heute kaum mehr angewandt."

148 Vgl. oben S. 34. Typhus Vakzine wurden wie Pyrifer häufig außerhalb der Psychiatrie angewandt: Zur Dermatologie vgl. die Wiener Dissertation von PAHNKE, Ueber den Einfluss der Fiebertherapie auf die Behandlung der Psoriasis vulgaris (1941).

149 ARENTSEN/WELNER, Hypertherm Treatment of Neurosyphilis (1955), S. 529-552; BOAS, On the Treatment of Neurosyphilis with Artificial Fever and Penicillin (1950), S. 24-34.

150 LHOTSKÝ, Kritische Betrachtung der modernen Neuroluesbehandlung (1951), S. 228f. Er führt in den Fußnoten zudem noch zwei Belege an, weshalb die Hitze nicht entscheidend sein könne: Erstens habe Truffi bei fiebernden Vögeln (> 42° C) und zweitens Wagner-Jauregg bei fiebernden Menschen (40°, manchmal auch über 41°) noch lebende Syphillis-Spirochäten entdeckt.

In den 1950er Jahren war die Steigerung der Durchlässigkeit der Blut-Liquor-Schranke alleine durch die hohen Temperaturen des Malariafiebers ein Argument für die Kombination der Penicillin- mit Malariafiebertherapie bei Neurolues.[151]

2.1.2 Zur Technik der Anwendung der Malariafiebertherapie an der Klinik

An der Wiener Klinik wurde im Untersuchungszeitraum (wie seit 1921) nur das *Plasmodium vivax* (Erreger der *Malaria tertiana*) durch Blutübertragung verwendet, einmal (1960) auch das ähnlich chininempfindliche *Plasmodium malariae* der *Malaria quartana* mit Blut aus dem Tropeninstitut Amsterdam; das *Plasmodium malariae* wurde aber nicht weitergegeben. Wesentlich für die positive Beurteilung der Verträglichkeit und Ungefährlichkeit der Therapie war, dass bei Blutübertragung die *Malaria tertiana* durch die Behandlung mit Chinin ohne die Gefahr von Malariarezidiven zu beenden ist,[152] da das Blut keine Sporozoiten enthält. Die Sporozoiten, wie sie von der Mücke übertragen werden, wandern in die Leber, nisten sich zunächst in den Leberzellen des Menschen ein, um hier das erste Mal ihre Form zu verändern: Die einzelligen Sichelkeime, die Sporozoiten, wachsen zu Hypnozoiten und zu vielkernigen Gebilden (Leberschizonten) heran. Erstere bleiben in der Leber, letztere spalten sich nach nur wenigen Tagen in viele einzellige Teilsprösslinge, Merozoiten; diese werden aus den Leberzellen freigesetzt und wandern ins Blut und weiter in die roten Blutkörperchen. Hier entwickeln sich die Merozoiten zu Blutschizonten, die jetzt nicht in den Leberzellen, sondern in den roten Blutkörperchen zu vielzelligen Gebilden heranwachsen, die wiederum in viele Merozoiten zerfallen. Dabei platzen die roten Blutkörperchen. Die neuen Merozoiten gelangen wieder in die Blutbahn, von dort wieder in die roten Blutkörperchen und so weiter. Jetzt erst zeigen sich auch die ersten Krankheitssymptome. In dieser Phase wird das Malariablut abgenommen und zur Fieberkur auf den nächsten Patienten / die nächste Patientin übertragen; es enthält keine Sporozoiten, die in die Leber wandern würden, dort als Hypnozoiten zurückbleiben und weitere Fieberanfälle auslösen könnten; Sporozoiten werden nur von der Mücke übertragen. Wenn auch nicht in dieser detaillierten Begründung, so war das bereits in der Entwicklung der Therapie unter Wagner-Jauregg festgestellt worden. Es war

[151] Vgl. die positiven Ergebnisse anderer Studien, zitiert in BAUMANN/KARDOS, Über die Schrankenprobleme bei der Penicillintherapie der Neurolues (1957), S. 26.
[152] Dazu ein Zitat aus dem Untersuchungszeitraum: PAMPANA, A Textbook of Malaria Eradication (1963), S. 53: "blood-induced malaria does not give rise to exoerythrocytic cycles in the receiver, so that it can be radically and rapidly cured with any schizontocide – even with quinine."-Pampana war Professor in Rom und "Director, Division of Malaria Eradication, WHO, Genova".

eine Voraussetzung für ihre Anwendung,[153] dass – so Julius Wagner-Jauregg 1936 – „die Impfmalaria in ganz anderem Masse als die natürliche Malaria chininempfindlich ist“, sodass es nach der Kupierung zu keinen Malariarezidiven kommt.[154]

Zur Praxis der Malariabehandlung an der Wiener Klinik werden aus den PatientInnenakten folgende Punkte ersichtlich:

- Voraussetzung war die Zustimmung des Patienten /der Patientin bzw. seines /ihres gesetzlichen Vertreters[155] zur Malariafiebertherapie, ein „Revers“[156], dessen Vorhandensein am oder im Patientenakt vermerkt wurde, der aber nur selten beiliegt; mehrmals wurde ein Revers eingeholt – auch für mehrere Therapien –, die Therapie aber nicht durchgeführt, obwohl auch die Freigabe des Internisten vorlag.
- Es wurden 4-6 (in Einzelfällen 10)[157] ccm Malariablut intravenös, seltener auch intramuskulär appliziert.[158] Üblicherweise wurde das Malariablut an der Klinik direkt vom / von der Spender/in auf den / die Empfänger/in übertragen, wodurch der ‚Malariastamm‘ erhalten blieb; malariainfiziertes Blut konnte sich auch „drei Tage und darüber […] infektionsfähig erhalten“,[159] sodass der Wiener ‚Stamm‘ auch transportiert und anderswo verwendet werden konnte.[160]
- Nach der Injektion des Malariablutes begann der erste Anstieg des Fiebers meist nach 5, max. 10[161] Tagen. In einigen wenigen Fällen schlug die Malariafiebertherapie

[153] KAUDERS, Zur Klinik, Theorie und Geschichte der Malariabehandlung (1948), S. 51.
[154] Bemerkungen Wagner-Jaureggs im Sammelband von 1936 zum Aufsatz von 1918/19 WAGNER-JAUREGG, Über die Einwirkung der Malaria auf die Progressive Paralyse (1918/19), S. 137.
[155] Mehrmals das Bezirksgericht als Vormundschaftsgericht.
[156] Ein ‚Revers‘ wurde auch bei der EKT, der Insulinkommatherapie und anderen Therapien (wie Cardiazol- und Majeptiltherapie) eingeholt. Eine Verweigerung der Zustimmung kam vor, wenn auch äußerst selten; vgl unten S. 145 Anm. 538. Vgl. auch unten S. 86 zur Geschichte der „*rechtlichen Regulierung von Zwangsmaßnahmen*“ in der Psychiatrie.
[157] HOFF, Lehrbuch der Psychiatrie (1956), S. 124: „5 bis 10ccm Blut i. m. oder i. v.“
[158] Entspricht dem, was WEINGARTEN, Therapie der Neurolues (1960), S. 242 ausführlicher beschreibt. So auch HOFF, Lehrbuch der Psychiatrie (1956), S. 124. Die Einleitung der Malariafiebertherapie mit Bluttransfusion war international üblich – mit wenigen Ausnahmen, in denen Mücken und sogar Malaria tropica verwendet wurden.
[159] Bemerkungen Wagner-Jaureggs im Sammelband von 1936 zum Aufsatz von 1918/19 WAGNER-JAUREGG, Über die Einwirkung der Malaria auf die Progressive Paralyse (1918/19), S. 137. Zu den Konservierungsmethoden für einen bis zu 4tägigen Transport in den 1920er Jahren: KAUDERS, Über Malariablutkonservierung (1926), S. 372.
[160] Lt. PAKESCH, Spezielle Therapie der Nervenkrankheiten (1951), S. 175, wurde an der Grazer Klinik das malariainfiziert Blut von der Wiener Klinik bezogen. Heinz Häfner erinnert sich, vor 1950 in München am Bahnhof Malariablut aus Wien für die dortige Klinik abgeholt zu haben (e-mail vom 19.05.2014 an Gernot Heiss).
[161] Diese Beobachtungen decken sich mit der Literatur, etwa: WEINGARTEN, Therapie der Neurolues (1960), S. 242.

jedoch nicht an. In diesen Fällen wurden manchmal Typhus Vakzine, selten auch Pyrifer zur Verstärkung bzw. zur Provokation des Fiebers injiziert.

- Es wurde in Wien ausschließlich *Plasmodium* verwendet, dass eine *Malaria tertiana* auslöst.[162] Diese ist nach der ihr spezifischen Fieberkurve benannt (ein Tag Fieber, ein Tag ohne Fieber, hiernach wieder Fieberanstieg), bei der Impfmalaria war jedoch dieser Rhythmus, „der Fiebertypus recht unregelmäßig."[163]
- Am Fiebertag wurde angestrebt, dass der Peak (höchster Anstieg der Fieberkurve) über 40° C kam.[164]
- Analeptika (z.B. Cardiazol, Coffein, Strychnin, Sympatol)[165] wurden vorwiegend gegeben, um Kollapszustände zu vermeiden, d.h. um den Blutdruck nicht zu stark absinken zu lassen. Zu diesem Zweck wurden häufig am Tag des Peaks, seltener auch einen Tag vor diesem, sogenannte Mischspritzen verabreicht, dabei kam es auch vor, dass diese mehrmals am Tag appliziert wurden. Diese enthielten eine Mischung aus Cardiazol und Strychnin.[166] In den 1960er Jahren wurde stattdessen Effortil (Etilefrin hydrochlorid) und Doca (Desoxycorticosterone Acetate), in den letzten Jahren des Untersuchungszeitraums und / oder „1A Akrinor [Cafedrin und Theodrenalin]+10mg Percorten [Desoxycorticosteron] i.m." gegeben.[167]
- Nach 4 bis 8,[168] in Einzelfällen bis zu 12 Fieberschüben wurde die Malariafiebertherapie durch Anti-Malariamittel immer über 7 Tage beendet und zwar immer nach einem der beiden Schemata:
    - Chinin (an den ersten 3 Tage 2x 0,5g/d, dann 4 Tage á 0,5g/d) + Atebrin (2x 0,1g /d)
    - Chinin (an den ersten 3 Tage 2x 0,5g/d, dann 4 Tage á 0,5g/d) + Resochin (1x 0,25g/d)[169]

---

[162] So auch WEINGARTEN, Therapie der Neurolues (1960), S. 242. Die eine Ausnahme der Impfung mit *Malaria quartana* aus Amsterdam, die nicht weitergegeben wurde, wurde bereits erwähnt.
[163] HOFF, Lehrbuch der Psychiatrie (1956), S. 124.
[164] Ebd.: „Als Anfall wird nur das Fieber über 39 bis 39,5° gerechnet. In den meisten Fällen ist der Fieberanstieg sogar höher, über 40°, ja sogar bis fast 42°." In den PatientInnenakten wurden nur einzelne Fälle mit Fieber über 41° gesehen.
[165] Vgl. BLEULER, Lehrbuch der Psychiatrie ([10]1960), S. 163 und S. 168.
[166] Vgl. WEINGARTEN, Therapie der Neurolues (1960), S. 242.
[167] 1961/62 bekam der Patient P574, der besonders hoch fieberte (2mal auf knapp über 41°), zusätzlich zu Effortil und Doca untertags auch „2cc Coffein+Strychnin i. m."
[168] Zur Behandlung der progressiven Paralyse wurden 8 Fieberschübe empfohlen. Die bei anderen Diagnosen recht häufig niedrigere Zahl dürfte auf den Einsatz der Malariafiebertherapie als ‚Erschütterungstherapie' und nicht als spezifische Behandlung wie bei der Neurosyphilis (Abtötung bzw. Schwächung der Spirochäten und Anregung der Immunkräfte) zurückzuführen sein.
[169] Lt. WEINGARTEN, Therapie der Neurolues (1960), S. 242: Es „wird besonders in Deutschland Santochin und Resochin, und zwar 10 Tabletten à 0,25 in 24 Stunden statt Chinin, verwendet." Lt. KAINZ, Retrospektive

Wesentlich für die Therapie war, mit *Plasmodium vivax* infiziertes Blut zur Übertragung zur Verfügung zu haben. Während etwa in Berlin das malariainfizierte Blut von einem / einer Patienten/in mit progressiver Paralyse auf die / den nächste/n übertragen wurde,[170] wurde in Wien standardmäßig luesfreies Malariablut verwendet, d.h. Blut, das nicht von NeuroluespatientInnen stammte.[171] Das war freilich die Voraussetzung für ihre Anwendung bei nicht-luetischen Krankheiten. Auf der Fieberkurve wurde nicht nur eingetragen, wann und in welcher Quantität ‚luesfreies *Malaria tertiana* Blut' injiziert wurde, sondern auch der Name des Spenders / der Spenderin.[172]

Dass die Malariafieberkur in den 1950er und 1960er Jahren in Wien und etwa auch in französischen Handbüchern[173] als ‚gut verträglich' galt, dürfte auf den positiven Erfahrungen beruhen, die mit der Blut-Übertragung von Mensch zu Mensch und der Verwendung eines durch Erfahrung bereits gut einzuschätzenden Stamms[174] der *Malaria tertiana* (in Wien bereits vor dem Krieg[175]) zusammenhängen, sowie mit der sorgfältigen Beobachtung des Blutdrucks durch ein kompetentes Personal, das rasch etwa bei Kreislaufkrisen oder bei Erregungszuständen des / der Patienten/in im hohen Fieber eingreifen konnte, und mit der Berücksichtigung der (wenigen) Kontraindikationen[176] – also mit der (nach Meinung der Wiener Kliniker) guten Beherrschbarkeit der Malariafiebertherapie und ihres Abbruchs mit

---

Datenanalyse iatrogener P. vivax Infektionen (2019), S. 31, die ausgehend vom Projektendbericht und der Datenbank des Projekts die Akten der NeuroluespatientInnen an der Klinik 1951-1969 untersuchte, wurde in Wien „ab 1960 Resochin statt Atebrin eingesetzt."

170 Vgl. unten S. 66 Anm. 204 zum Hinweis von Hanns Hippius über die Anwendung an der Berliner Klinik.

171 Von den in der Datenbank verzeichneten 869 Malariafiebertherapiefällen wurde nur bei 3 NeuroluespatientInnen das malariainfizierte Blut von 2 Neuroluespatienten übertragen; s. unten S. 66 Anm. 204.

172 Dieser Name fehlt nur bei 19 dieser 869 Einträge. Ebenfalls nur ganz selten wurde auch in der Fieberkurve des Spenders die Weitergabe vermerkt – hier aber ohne Mengenangaben und ohne Empfängernamen.

173 Es geht hier um die Verträglichkeit für ParalytikerInnen, also für PatientInnen, die häufig alt, schwach und körperlich krank waren. Dazu schrieb F. Ramée in POROT, Manuel ([1]1952), S. 303-305, nach der Nennung der Kontraindikationen: « Le plupart des parlytiques généraux […] sont susceptible de suivre ce traitement. »

174 Dass es wichtig sei, einen Malariastamm zu erhalten, mit dessen Anwendung man gute Erfahrungen hatte, wurde mehrfach betont. KAUDERS, Zur Klinik, Theorie und Geschichte der Malariabehandlung (1948), S. 51f. schreibt von „über 600 Passagen" des leider im Krieg verlorenen Malariastamms und betont, dass ein „in seinen klinischen und biologischen Eigenschaften genau bekannter" *Malaria tertiana* Stamm zu verwenden sei. Der rumänische Malariologe Lupascu verwendet 1974 (zitiert unten S. 82) dieses Argument, um die Impfung von PatientInnen ohne Syphilis zu rechtfertigen.

175 Nachdem es in Wien zu einigen Todesfällen gekommen war, auch durch die versehentliche Impfung mit *Plasmodium falciparum* (es löst *Malaria tropica* aus), konnte ab Juni 1917 in enger Zusammenarbeit mit der Dermatologie ein weniger virulenter Stamm hergestellt werden, der die *Malaria tertiana* auslöst, mit welchem mit großen Erfolgen bei Paralytikern weitergearbeitet wurde: KOHL, Wagner von Jauregg (1993), S. 158f.

176 WEINGARTEN, Therapie der Neurolues (1960), S. 242: „Kontraindikation zur Malariabehandlung sind nur schwere chronische Erkrankungen, wie z.B. chronische Nephritis, Koronarsklerose, Leberzirrhose oder schwer dekompensierte Herzleiden. Kompensierte Klappenfehler oder Mesaortitis gelten nicht als solche. Fettleibige und hochgradig unterernährte Patienten müssen vor und während der Kur sorgsam überwacht werden.

Chinin. Nach den zwischen 1951 und 1969 archivierten Akten starben an der Wiener Klinik eine Patientin und zwei Patienten während einer Malariafiebertherapie (3 von 869 in der Datenbank verzeichneten PatientInnen, die eine Malariafiebertherapie erhielten). [177]

2.1.3 Zur vielfältigen Anwendung der Malariafiebertherapie an der Wiener Klinik: Vorgangsweise der Untersuchung und Auswertung

Wie in der Einleitung beschrieben, wurden mit der Frage nach der Häufigkeit und Zielsetzung der Anwendung der ‚großen' körperlichen Kuren an der Wiener Psychiatrie fünf Diagnosen gewählt (Neurolues, Intelligenzmängel, Schizophrenie, affektive Störungen, Psychopathie), mit denen die Fälle – bei einer Aufenthaltsdauer der Patientin / des Patienten an der Klinik von mehr als 4 Tagen – in die Datenbank aufgenommen wurden. Zwar waren es die fünf Diagnosen, bei denen nach einer ersten Sichtung häufig die ‚alten' Kuren angewandt wurden, es war jedoch nicht auszuschließen, dass auch Patienten mit anderen Diagnosen eine dieser Therapien bekamen. Um bei der Malariafiebertherapie eine bestmögliche Vollständigkeit zu erreichen, wurden alle PatientInnen aufgenommen, die diese Therapie erhielten. Deshalb sind in diesem Kapitel nicht nur die 772 Fälle mit einer der fünf als Aufnahmekriterium gewählten Diagnosen enthalten, es werden auch die 97 Patienten berücksichtigt, deren Diagnosen am Deckblatt des Krankenakts sich keiner der fünf Diagnosen zuordnen lassen.[178] Von den Fällen, die in der Datenbank verzeichnet sind und in denen die in der Zwischenkriegszeit entwickelten Krampf-, Koma- und Fiebertherapien angewandt wurden (6.915), hatte die Malariafiebertherapie einen Anteil von 11,164%.[179]

Zur Quellenproblematik ist darauf hinzuweisen, dass von 127 SpenderInnen, die auf der Fieberkurve des Empfängers / der Empfängerin genannt werden, kein Krankenakt im

---

[177] 1951 die 23jährige Patientin I/S896 („Pfropfhebephrenie"; im Obduktionsbericht werden Hirnblutungen als Todesursache angegeben). 1952 der 27jährige Patient S236 („Schizophrenie, stat. thymolymphaticus, [darunter nachgetragen] Kollaps im Erregungszustand"; zu ihm unten im Text S. 88f. Der 60jährige Patient N107 („p.P., Malariakur, Lebercir[r]hose, Hirnödeme"; letztere Diagnosen aufgrund des Obduktionsberichts) kam 1958 nach einer Penicillinkur und nachdem er bereits „vergangene Woche mit Malariablut geimpft" worden war, vom Rosenhügel an die Klinik; lt. Rosenhügel-Parere „Anfalls-P.P.", mit linksseitigen Krämpfen - „ist renitent, bedroht Schwestern und Ärzte. Bettflüchtig". Er verstarb nach dem 2. Fieberschub „unter den Zeichen der Herz- und Kreislaufschwäche"; vgl. auch S. 58 Anm. 180.

[178] Da zu diesen Fällen mit Diagnosen außerhalb der Einschlusskriterien keine Vergleichsfälle ohne Malariakur aufgenommen wurden, scheinen sie nur in jenen statistischen Auswertungen auf, in denen es nicht um einen Vergleich der Anwendung und Nichtanwendung geht: vgl. unten S. 61f.

[179] Für den Vergleich wurden nur die 772 Fälle mit Malariakur genommen, deren Diagnosen sowie Aufenthaltsdauer den Einschlusskriterien entsprachen, wodurch auch die vergleichbaren Fälle ohne Malariafiebertherapie in die Datenbank aufgenommen wurden.

Untersuchungszeitraum archiviert ist. Wahrscheinlich ist, dass seine / ihre Krankengeschichte nach 1969 archiviert wurde oder verloren ging bzw. in einigen Fällen der / die Patient/in mit malariainfiziertem Blut aus der Klinik anderswo behandelt wurde und nun wieder als SpenderIn diente.[180] Trotz der umfangreichen Überlieferung ist davon auszugehen, dass nicht alle PatientInnenakten überliefert sind.[181]

Im folgenden Balkendiagramm sind die in den Jahren 1951 bis 1969 archivierten und in die Datenbank aufgenommenen insgesamt 869 Malariakuren abgebildet:

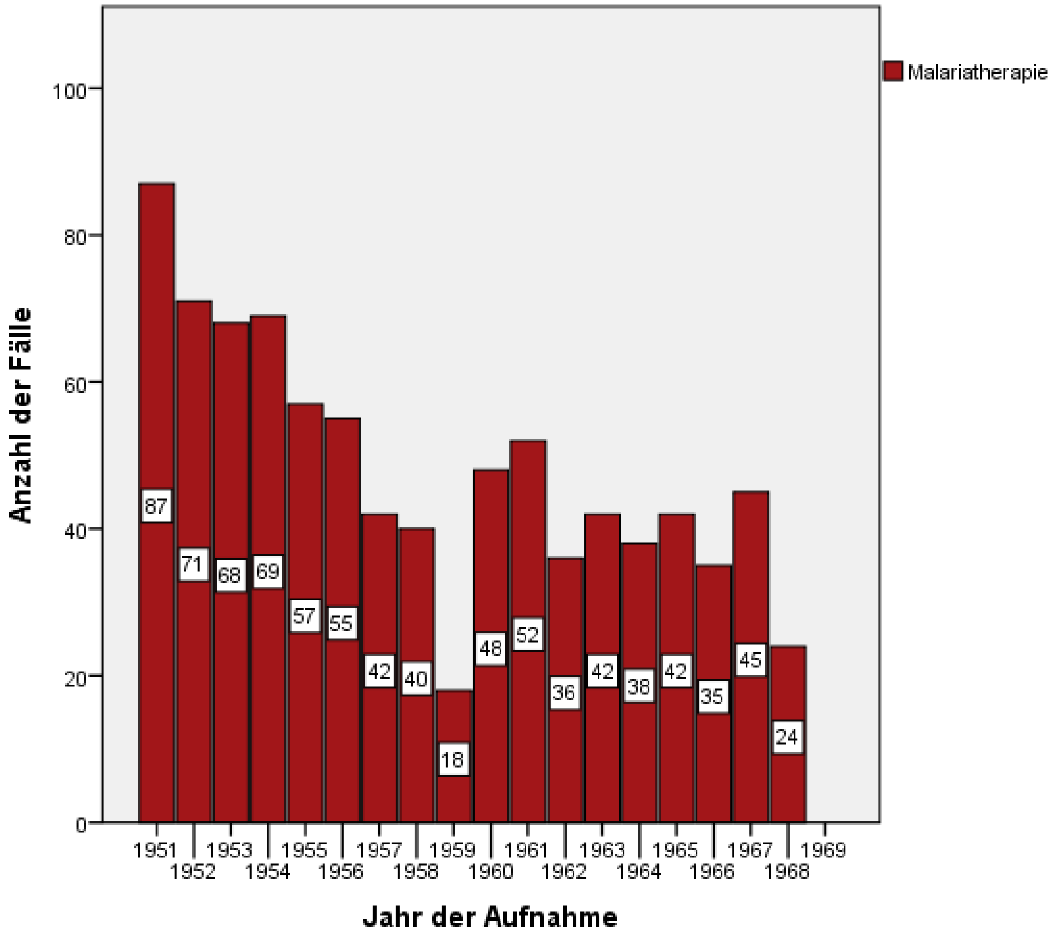

Abb. 1 Balkendiagramm, absolute Anzahl aller Malariakuren in den vorliegenden Akten (1951-1969), n = 869.

[180] Die Erinnerung eines Patienten, dass er 1955 in der Nachbehandlung der Kinderlähmung zur Impfung mit malariainfiziertem Blut vom Wilhelminenspital auf den *Steinhof* gebracht wurde (s. unten S. 79 Anm. 264), legt diese Interpretation nahe, ist freilich als Erinnerungen fast 60 Jahre später nicht als sicherer Beleg zu werten. Der oben Anm. 177 genannte 60jährige Patient N107 mit „Anfall-P.P.", wurde 1958 5 Tage vor seiner Verlegung an die „Klinik Hoff" am Rosenhügel mit malariainfiziertem Blut geimpft. Sein malariainfiziertes Blut wurde jedoch nicht weitergegeben. Er dürfte mit malariainfiziertem Blut aus der Klinik geimpft worden sein, denn es ist unwahrscheinlich, dass es am Rosenhügel einen *Malaria tertiana* Stamm gab.

[181] Vgl. zu Aktenverlusten bei den Übersiedlungen 1974, oben S. 15.

Die ursprüngliche Hypothese im Projekt ging vor allem aufgrund der Abnahme der PatientInnen mit Neurolues-Diagnosen davon aus, dass die Malariafiebertherapie sukzessive abnehmen würde. Bis 1959 schien sich diese Annahme zu bestätigen, 1960 stieg jedoch die Häufigkeit der Malariafiebertherapien wieder deutlich an. Die Malariafiebertherapie wurde in den 1960er Jahren zwar nicht mehr so häufig wie noch in den frühen 1950er Jahren angewandt, dennoch wurden zwischen 1960 und 1968[182] im Durchschnitt mehr als 40 Malariafiebertherapien pro Jahr durchgeführt (lt. vorliegenden PatientInnenakten: $\bar{x}$ = 40,22). Dazu ist zu bemerken, dass bei der Annahme einer durchschnittlichen Dauer von der Impfung bis zur Weitergabe von ca. 15 Tagen ca. 24 „StammträgerInnen" notwendig waren, um die Kette aufrecht zu erhalten – was einen Teil der Steigerung von 1959 auf die 1960er Jahre und der folgenden Regelmäßigkeit erklären dürfte.

Nach der Boxplot-Auswertung liegt das Alter der männlichen Patienten, die eine Malariakur bekommen haben bei 21 Jahren, weit unter dem Median aller in der Datenbank aufgenommenen Fälle (35 Jahre); hingegen liegt der Median der Frauen, die eine Malariakur bekommen haben über dem Median des Alters aller in der Datenbank aufgenommenen PatientInnen: bei 41 Jahren. Dies ist darauf zurückzuführen, dass die meisten Frauen, die eine Malariakur bekommen haben, eine neuroluetische Erkrankung hatten und das Durchschnittsalter dieser Gruppe älter war als jenes der anderen, und dass die überwiegende Zahl der männlichen Patienten mit nicht-neuroluetischen Diagnosen in jugendlichem Alter die Malariakur bekamen. Auffällig länger war die Aufenthaltsdauer der männlichen im Vergleich zu den weiblichen MalariatherapiepatientInnen (ca. 30 : 16). Das dürfte daran liegen, dass mehrmals Neurolues-PatientInnen – und häufiger Frauen als Männer – schon während der Malariakur auf den *Steinhof* überwiesen wurden, während die nicht-luetischen, fast nur männlichen Malariafieberpatienten bis zum Ende der Therapie an der Klinik behalten wurden – was bei vielen von ihnen (als Stammträger) auch zur Übertragung des „luesfreien" Malariablutes kurz vor den Chinintagen notwendig war.

Die folgende Tabelle zeigt nach Jahren die Häufigkeit der Malariafiebertherapie bei den 5 Diagnosen, die als Aufnahmekriterien gewählt wurden. Farbig markiert wurde die Fallzahl in jener Diagnose, die im betreffenden Jahr die höchste war:

[182] 1969 gab es in den vorliegenden PatientInneakten keine Malariakur mehr. Die letzte Impfung erfolgte im Dezember 1968; es war der 21jährige Patient X872 mit der (außerhalb der Einschlusskriterien stehenden) Diagnose „Medikamentenüberdosierung, neurotische Reaktion".

+

Diagnose	Intelligenzmängel	Neurolues	Schizophrene Erkrankungen	Psychopathie	Manisch-depressive Erkrankungen	Gesamt (alle Patienten)
	Anzahl Fälle	Anzahl Fälle	Anzahl Fälle	Anzahl Fälle	Anzahl Fälle	Anzahl Fälle
1951	21	35	30	11	1	84
1952	24	27	21	4	5	68
1953	11	24	20	7	6	59
1954	18	22	34	3	5	65
1955	15	10	22	13	4	53
1956	12	6	23	16	3	51
1957	21	7	13	9	6	39
1958	17	5	7	10	5	38
1959	10	0	8	2	3	17
1960	18	4	3	18	7	44
1961	15	9	5	12	9	43
1962	15	0	1	15	7	34
1963	15	2	3	18	8	40
1964	8	4	0	11	8	28
1965	12	3	0	27	6	40
1966	7	2	2	11	3	22
1967	6	3	1	11	16	32
1968	10	0	0	7	5	15
1969	0	0	0	0	0	0
Gesamt	255	163	193	205	107	772

Tab. 11 Diagnosen und Malariafiebertherapie (1951-1969), bei den vorliegenden Akten, aufgenommen aufgrund der Einschlusskriterien (Diagnose, Aufenthaltsdauer), n = 772; Mehrfachnennungen aufgrund von Doppeldiagnosen sind möglich!

Über die Jahre verändern sich schwerpunktmäßig die Diagnosefelder, die zur Malariafiebertherapie geführt haben. Dass sich auch die Häufigkeit im Bereich der Unterdiagnosen, die in einigen Fällen aus der Datenbank erkennbar sind, veränderten, zeigen die gestapelten Balkendiagramme in den Unterkapiteln zu den Diagnosen.

In den 1960er Jahren mehren sich Malariakuren bei Patienten[183], welche mit Diagnosen aufgenommen wurden, die rein von diesen aus betrachtet in den 1950er Jahren kaum eine

[183] Von den Patientinnen, deren Akten in die Datenbank aufgenommen wurden, wurden nach Jänner 1960 mit der Ausnahme der 19jährigen Privatpatientin I904 („Imbezillität“) aus dem Ausland, die 1960/61 eine Malariafiebertherapie bekam, und der 17jährigen Patientin P194 („Psychopathisches Syndrom“, vgl. unten S.

Indikation zur Malariafiebertherapie darstellten.[184] Nun finden sich Patienten, die an der Klinik mit Malariafiebertherapie behandelt wurden, mit den Diagnosen „Alkohol chr[onisch]" (8mal ohne zusätzliche Diagnose), „neurotische Verwahrlosung" (in der Diagnosezeile 3mal ohne zusätzliche Bemerkung, 5mal mit SMV-Hinweis und 1mal mit dem Zusatz „Erregungszustände"), „Aggressionsneurose" (1mal ohne zusätzliche Diagnose) oder Kombinationen wie „neurotische Verwahrlosung, Alk. Mißbrauch" oder „Verwahrlosung, Alkoholmissbrauch" (je 1mal). Auch die Patienten mit der Diagnose „Neurose" in der Diagnosezeile (ohne oder mit Zusätzen wie SMV, Zwangsmechanismen, Alkoholismus) und mit einer Malariafiebertherapie finden sich in der Datenbank fast nur in den 1960er Jahren.[185]

Aus diesem Grund wurden für die Auswertung der gesamten 869 Malariakuren drei weitere Diagnose-Klassen eingeführt: Alkoholismus, Neurosen und „Verwahrlosung" (in der folgenden Tabelle mit * gekennzeichnet). Da jedoch diese Diagnosen außerhalb der den Einschlusskriterien entsprechenden fünf Diagnosen nicht vollständig, sondern nur vollständig im Zusammenhang mit der Malariafiebertherapie aufgenommen wurden, können keine statistischen Vergleiche der unterschiedlichen Behandlungsschemata bei diesen Diagnosen erfolgen. Diese 97 Fälle sind deshalb nur in jener Darstellung enthalten, in der es nur um die Malariafiebertherapie geht, wie in der folgenden Tabelle. Sie zeigt alle 869 Fälle von Malariafiebertherapie gegliedert nach den Diagnosen einschließlich der Diagnosen, die nicht den Einschlusskriterien entsprechen. Außerdem wurde nach dem Geschlecht gegliedert:

120f.), die 1963 eine Malariafiebertherapie bekam, nur mehr Patientinnen mit Neurolues mit Malariafiebertherapie behandelt.

[184] Eine Ausnahme bilden 2 Fälle 1951 mit „Neurotische Verwahrlosung" (X586, 16jährig) bzw. „Erregungszustände, innere Verwahrlosung" (X200, 20jährig).

[185] Vgl. unten S. 145 – 150, den Abschnitt zur Malariafiebertherapie bei Diagnosen, die nicht den Einschlusskriterien der Datenaufnahme entsprachen.

Gesamt (Akten)	799<br>100% | 92,0%	70<br>100% | 8,0%	869
	Männlich	Weiblich	Gesamt
	Anzahl (Fälle)	Anzahl (Fälle)	Anzahl (Fälle)
Intelligenzmängel	249	7	256
S-%|Z-%[186]	23,8% | 97,3%	8,6% | 2,7%	22,7% | 100%
Neurolues	112	51	163
S-%|Z-%	11,5% | 68,2%	69,1% | 31,8%	15,6% | 100%
Schizophrene Erkr.	187	8	195
S-%|Z-%	17,9% | 95,9%	9,9% | 4,1%	17,3% | 100%
Psychopathie	201	4[187]	205
S-%|Z-%	19,2% | 98,0%	4,9% | 2,0%	18,2% | 100%
Affektive Erkr.	104	4	108
S-%|Z-%	9,9% | 96,3%	4,9% | 3,7%	9,6% | 100%
Alkoholismus*	79	0	79
S-%|Z-%	7,6% | 100%	0,0% | 0,0%	7,0% | 100%
Neurotische Erkr.*	54	0	54
S-%|Z-%	5,2% | 100%	0,0% | 0,0%	4,8% | 100%
"Verwahrlosung"*	25	1	26
S-%|Z-%	2,4% | 96,2%	1,2% | 3,8%	2,3% | 100%
Diagnose nicht klassifizierbar*	27	1	28
S-%|Z-%	2,6% | 96,4%	1,2% | 3,6%	2,5% | 100%
Gesamt (Akten)	799<br>100% | 92,0%	70<br>100% | 8,0%	869

Tab. 12 Malariatherapie: Geschlecht und Diagnosen (1951-1969), n = 869 Aufnahmen, Mehrfachzuordnungen bei Diagnosen wie Neurolues und „Debilität“, Pfropfhebephrenie, Depression und „intellektuelle Unterbegabung“ u. a. sind recht häufig.

Die Fiebertherapien bei PatientInnen mit Neurolues sind anteilsmäßig an den Aufnahmezahlen (Gesamtzahl der Aufnahmen von NeuroluespatientInnen[188] 371, männlich 231, weiblich 140 : Gesamtzahl der Fiebertherapien bei NeuroluespatientInnen 163, männlich 112, weiblich 51) in etwa gleich häufig unter den Geschlechtern verteilt, d.h. die Wahrscheinlichkeit, dass man als männlicher bzw. weiblicher Patient eine Fiebertherapie bei

[186] Zu diesen Kürzeln vgl. oben S. 34 bei Tabelle 1.
[187] In dieser Tabelle sind mehrere Fälle mehrfach zugeordnet. So ist z. B. die 19jährige Patientin P/N157, die 1953 mit der Diagnose „Psychopathie, Paralysis iminens“ eine Malariafiebertherapie bekam, doppelt zugeordneten und auch hier eingerechnet, sie wird in den Ausführungen im nächsten Absatz („3 [Frauen hatten] die Diagnose Psychopathie“) jedoch nicht doppelt, sondern nur zur Neurolues gezählt.
[188] Vgl. oben S. 41-43.

Neurolues bekam, war etwa gleich groß.[189] Aber generell wurden mit einer Malariakur mehr Männern als Frauen behandelt. Rechnet man hier noch die PatientInnen mit Neurolues heraus, so haben ohne diese Diagnose nur in 13 Fällen Frauen eine Malariatherapie bekommen, hingegen 693 Männer; demnach waren 98,16% aller Patienten, die eine Malariakur erhielten und keine Neurolues hatten, männlich. Von den 13 Frauen, die ohne Neurolues eine Malariatherapie bekamen, hatten 4 Patientinnen die Diagnose Intelligenzmängel, 4 die Diagnose Schizophrenie, 2 affektive Störungen (einmal als zweite Diagnose „Unterbegabung"), 3 die Diagnose Psychopathie.

Bemerkenswert ist, dass die beiden letzten der seltenen Patientinnen, die ohne Neurolues eine Malariatherapie erhielten, Patientinnen von Hoff waren: 1960/61 die 19jährige Privatpatientin I904 „Imbezillität") aus dem Ausland und 1963 die 17jährige Patientin P194 („psychopathisches Syndrom"), die aus der Privatordination Hoff zur(!) Malariatherapie an die Klinik kam.[190] Die 11 Patientinnen, die in den 1950er Jahren ohne Neurolues mit Malariakur behandelt wurden, sind unregelmäßig verteilt: 4 Patientinnen bekamen sie 1951, 3 1953, eine 1955 und 3 1959. Warum im Vergleich mit den männlichen Patienten so wenige Frauen mit den Diagnosen außerhalb der Neurolues eine Malariatherapie erhielten, dürfte nicht zu klären sein – weder in den Krankenakten noch in der zeitgenössischen Literatur konnten dazu Hinweise gefunden werden.

2.1.4 Die Malariafiebertherapie bei den einzelnen Diagnosen in ihrer Anwendung an der Klinik und in der wissenschaftlichen Diskussion der Zeit

In den folgenden Abschnitten wird die breite Anwendung der Malariafiebertherapie an der Wiener Klinik aufgrund der Auswertung der im Projekt erstellten Datenbanken in die zeitgenössische Diskussion gestellt: zuerst in die Diskussion, ob die ‚klassische' Anwendung bei der progressiven Paralyse durch die Einführung von Penicillin überholt sei; dann in die (seltenen) Erwähnungen der Anwendungen bei anderen psychischen Erkrankungen bzw. in die Diskussion über diese Krankheiten und ihre Behandlung.

---

[189] Die Malariafibertherapie ist in Wien bei progressiver Paralyse (gemeinsam mit Penicillin) Standardtherapie geblieben; vgl. dazu die Ausführungen unten im Abschnitt zur Malariakur bei der Diagnose Neurolues.
[190] Zu ihr vgl. unten S. 120f.

2.1.4.1 Die Diskussion über Malariafiebertherapie und / oder Penicillin bei neuroluetischen Erkrankungen: die Position und Praxis der Wiener Klinik

Bereits Ende der 1940er Jahre wurde diskutiert, ob das seit 1943 eingesetzte und nach 1945 sukzessive allgemein verwendete Penicillin bei der progressiven Paralyse[191] die Malariafieberkur ersetzen soll. Eine Zusammenfassung dieser frühen Debatte präsentierten die beiden Pariser Psychiater R. Bessiere und J. Alizon beim 1. Weltkongress der Psychiatrie 1950 in Paris:[192] Zwar hätten Stovarsol (das 1921 am Institut Pasteur durch Ernest Fourneau entwickelte, antibakteriell wirkende Acetarsol, aus der Gruppe der org. Arsenverbindungen) und die Malariafiebertherapie Erfolge in der Behandlung der bis dahin nicht wirkungsvoll therapierbaren Neurolues gebracht, die Erfolge – von Optimisten mit 30%, von Pessimisten mit 10% angegeben – seien aber doch rasch als zu gering eingeschätzt worden. Das seit 1943 vor allem in angelsächsischen Ländern häufig verwendete Penicillin stelle eine verheißungsvolle Alternative zur Malariafiebertherapie dar. In Nordamerika werde Penicillin als erste Therapie bei progressiver Paralyse eingesetzt und in günstigen Fällen würde eine einmalige Kur mit 6 Millionen Einheiten als genügend erachtet. Die Frage, ob es notwendig sei, Penicillin gemeinsam mit der Malariafiebertherapie anzuwenden, stehe allerdings noch im Raum. Die Ergebnisse der amerikanischen Untersuchungen seien nicht eindeutig. Sie zitieren schließlich Jean Delay (1907-1987)[193], der in seinem Buch über die biologischen Methoden in der Psychiatrie[194] weiterhin Penicillin-Serien mit bis zu 25 – 30 Millionen Einheiten befürwortete, aber auch meint, dass ‹ die Verbindung von Penicillin mit anderen Behandlungen die Regel › sein müsse (andere Behandlungen waren nicht nur die Malariafiebertherapie[195]). Diese Position sei jene vieler älterer französischer Psychiater, die zögern würden, die alten Therapien aufzugeben und diesen deshalb die neuen hinzufügen.

---

[191] Die Malariafiebertherapie wurde bei Neurolues-PatientInnen fast ausschließlich bei progressiver Paralyse und in einzelnen Fällen bei Tabes dorsalis gegeben; vgl. unten S. **69** Anm. 220 und S. 75 Anm. 244.

[192] BESSIERE/ALIZON, Premiers résultats de l'introduction de la pénicilline dans le traitement de la paralysie générale (1950), S. 1 (folgende Übersetzungen in einfachen Guillemets von GH).

[193] Er war ab 1946 Professor an der Pariser Universitätsklinik für Psychiatrie und Chefarzt am psychiatrischen Krankenhaus St. Anne und mit Henry Ey (1900-1977), seinem älteren Kollegen, der ebenfalls beide Funktionen innehatte, Vorsitzender dieses ersten Weltkongress der Psychiatrie. Zu Delays Bedeutung für die Entwicklung des ersten Neuroleptikums Chlorpromazin vgl. unten S. 274 und S. 278.

[194] DELAY, Méthodes biologiques en clinique psychiatrique (1950).

[195] Vgl. unten S. 69 Anm. 220.

In England wurde zum Vergleich von Penicillintherapie und Malariafiebertherapie bereits seit 1946 kontrovers publiziert, nachdem hier Penicillin bereits in diesen Jahren verfügbar war.[196] In der Diskussion dürften die Lebenserfahrungen der Diskutierenden eine Rolle gespielt haben: James Purdon Martin erwähnt 1948 in seinem Aufsatz, in dem er in allen Fällen von Neurolues die Therapie mit Penicillin alleine befürwortet, dass er immer schon die Malariafiebertherapie abgelehnt habe.[197] W. D. Nicol (Psychiater am Londoner Royal Free Hospital) nahm hingegen bereits in der Diskussion zu diesem Artikel von Martin 1948 gegen ein völliges Aufgeben der Malariafiebertherapie Stellung; er, der Wagner-Jauregg 1936 besucht hatte und mit ihm in diesen Jahren in Briefkontakt geblieben war,[198] argumentierte auch noch in den 1950er Jahren für eine Beibehaltung in bestimmten Fällen.[199]

W. D. Nicol erwähnte in einem Aufsatz 1956, dass nach einer Befragung von Ärzten, die seit 1954 unter der Leitung des namhaften Tropenmediziners Sir Gordon Covell[200] durchgeführt würde, in Belgien, Dänemark, Deutschland, Frankreich, Holland, Italien, Norwegen, Österreich, Schweden, Schweiz und Tschechoslowakei noch Malariafiebertherapie in Kombination mit Penicillin Standard sei. In Kanada sei zwar Penicillin plus Malaria die Methode der Wahl, „although penicillin alone is largely used owing to the lack of available malaria“.[201]

Der Mangel an Malaria infiziertem Blut war ein Hauptproblem mehrerer Kliniker/innen, die eine kombinierte Therapie bevorzugten: Jørgen Madsen von der Klinik in Roskilde

---

[196] Vgl. W. D. NICOL, Treatment of neurosyphilis: a comparison between malaria plus tryparsamide and malaria therapy (1946), S. 112-121. Vgl. MARTIN, The treatment of neurosyphilis with penicillin (1948), S. 89-100; die Diskussion dazu, in der Penicillin fast von allen Diskutierenden nur positiv beurteilt wurde: MARTIN et al., Discussion on the treatment of neurosyphilis with penicillin (1948), S. 100-103.
[197] MARTIN, The treatment of neurosyphilis with penicillin (1948), S. 90: „I confess that I have always had a strong dislike of malarial therapy and have used it with great reluctance.”
[198] W. D. NICOL, Wagner von Jauregg (1957), S. 126.
[199] MARTIN et al., Discussion on the treatment of neurosyphilis with penicillin (1948), S. 100f.; W. D. NICOL, General paralysis of the insane (1956), S. 9-16.
[200] PatientInnen mit Malariafiebertherapie wurden auch noch in den 1950er Jahren im Rahmen des Malaria Eradication Programms der WHO herangezogen, um am Ende der Therapie neue Malariamedikamente zu testen. Vgl. HULVERSCHEIDT, Forschungslenkung international – Malariaforschung im Rahmen des Malaria Eradication Programme der WHO 1955-1972 (2010), S. 133-146. In Wien fand sich kein Hinweis auf eine Mitarbeit an diesen Forschungsprogrammen (vgl. unten S. 169 Anm. 629). Wie in Deutschland, so könnte das Wissen um die menschenverachtenden Versuche mit geimpftem Malariafieber in der Zeit des Nationalsozialismus aufgrund der Prozesse nach 1945 eine Mitarbeit an diesen Forschungsprogrammen verhindert haben; vgl. HULVERSCHEIDT, Germann Malariology experiments with humans, supported by the DFG (2006), S. 221-236, und HULVERSCHEIDT, Die Beteiligung von Mitarbeitern des Robert Koch-Instituts an Verbrechen gegen die Menschlichkeit (2009), S. 147-168.
[201] W. D. NICOL, General paralysis of the insane (1956), S. 13f.

(Dänemark), der 1950 in der Kombination mit Penicillin für die Malariafiebertherapie im Vergleich zur besonders in Kopenhagen erprobten Hypertherm-Behandlung argumentierte (letztere hätte nicht den für die Malariakur so charakteristischen „roborating effect“), schreibt nach seinen Behandlungsvorschlägen: „One difficulty attached to this scheme of treatment: in Denmark the number of patients given malariotherapy is scarcely sufficient to maintain the malaria strain [die Kette der Übertragungen – hier nur mit Patienten/innen mit PP, GH], but it may be possible to solve the difficulty by cooperation with Swedish hospitals. [202]

Die hier angesprochene Methode, den „Malariastamm“ durch Übertragung des malariainfizierten Blutes von PatientInnen mit progressiver Paralyse auf PatientInnen mit progressiver Paralyse zu erhalten, war an vielen Kliniken, die die Malariakur nur bei PatientInnen mit progressiver Paralyse anwandten, Anfang der 1950er Jahren die übliche Methode. Während es in Wien üblich und durch die Anwendung bei nichtluetischen Erkrankungen auch möglich war, einen „luesfreien“ Stamm aufrecht zu erhalten,[203] wurde etwa an der Psychiatrie der FU-Berlin in den 1950er Jahren nach einer Information von Hanns Hippius gestaffelt nur unter Neurolues-PatientInnen weitergegeben.[204] Diese alleinige Anwendung bei progressiver Paralyse führt zum Problem, das Madsen angesprochen hat: Durch die erfolgreiche Behandlung der Syphilis in frühen Stadien mit Penicillin nahmen in den 1950er Jahren die Fälle von progressiver Paralyse radikal ab, sodass damit die Planung einer Stammträger/innen-Kette nicht mehr möglich war.[205] Hier mussten die Lücken durch Malariablut aus den Tropeninstituten oder aus einer anderen Klinik, an der mit

---

[202] MADSEN, Treatment of neurosyphilis (1950), S. 13-23. Vgl. zur Anwendung der Malariathrapie in Dänemark Ende der 1940er Jahre KRAGH, Malaria, Sulfosin and Metallosal in the Treatment of Mental Disorders in Denmark (2013), S. 112; der Artikel fokussiert auf Malaria- und andere, dänische Therapien in der Zwischenkriegszeit (Fiebertherapie mit Sulfosin, einem Sulfat-Öl-Präparat; Therapie mit Mangan-Salz) nicht nur bei progressiver Paralyse, sondern auch bei anderen psychiatrischen Krankheiten, vor allem bei Schizophrenie. Madsen erwähnte diese Anwendungen nicht mehr.

[203] In Wien wurde nur in Einzelfällen das malariainfizierte Blut von NeuroluespatientInnen an NeuroluespatientInnen weitergegeben. In der Datenbank finden sich lediglich 1950/51 und 1954 2 Patienten (N32, N80) mit PP, von denen Malaria an 3 PP-PatientInnen (N37, N79 und N172) übertragen wurde**.** In Wien wurde nur in Einzelfällen das malariainfizierte Blut von NeuroluespatientInnen an NeuroluespatientInnen weitergegeben.

[204] Hanns Hippius an Gernot Heiss, e-mail vom 12.8.2014: „Der Malaria-Stamm wurde an der Berliner Klinik so ‚gepflegt’, dass immer mindestens ein Patient eine Malaria-Kur begann, so dass der Stamm erhalten blieb.“ An eine Übertragung von luesfreiem Blut bzw. an ihre Anwendung bei anderen Diagnosen hatte Hippius keine Erinnerung. Auch MICHAUX, Psychiatrie (1965), S. 516 schrieb nur von einer Übertragung von einem Patienten mit PP « impaludé et en cours d’accès“; der Plasmodiumstamm werde „durch aufeinanderfolgende Übertragungen aufrechterhalten“.

[205] Zur gleichen Methode in Dänemark den Stamm zu erhalten und den Problemen, die sich daraus durch die starke Abnahme der Neurolues bereits Ende der 1940er Jahre ergab, vgl. KRAGH, Malaria, Sulfosin and Metallosal in the Treatment of Mental Disorders in Denmark (2013), S. 112.

NeuroluespatientInnen oder aber – wie in Wien – mit PatientInnen mit anderen Erkrankungen die Kette aufrecht erhalten wurde, geschlossen werden: So bezog nach den Erinnerungen von Miloš Vojtěchovský[206] die Prager psychiatrische Heilanstalt Bohnice zwischen 1953 und 1956 zur kombinierten Therapie bei ParalytikerInnen malariainfiziertes Blut von der Psychiatrischen Heilanstalt Pezinok bei Bratislava. [207]

Das Hauptargument für die Kombination findet sich in wissenschaftlichen Publikationen der Zeit: In einer Diskussion in *Der Nervenarzt* 1953/55 verweist Helmut Grage (Chemnitz) auf die Ergebnisse der Studie von Ehrig Lange in Jena von 1952,[208] wonach Penicillin alleine die entzündlichen Erscheinungen beseitigen, „die kombinierte Malaria-Penicillinkur aber in stärkerem Maße die seelischen Auffälligkeiten" beeinflussen würde. In einer seit 1952 laufende Studie in Chemnitz würde sich bestätigen, „daß die Erfolge der kombinierten Therapie im Fall von psychischen Auffälligkeiten besser sind".[209] Auch im Bericht von Rudolf Degkwitz in *Der Nervenarzt* 1955 über eine Studie an der Klinik Frankfurt a. M. wurden deutlich bessere klinische Erfolge mit der Kombination vor allem in der Behandlung der progressiven Paralyse beschrieben. Degkwitz beklagt, dass man zur Zeit geneigt sei, „wegen der großen Schwierigkeit, Malariablut zu bekommen, sich auf die alleinige Penicillinbehandlung zu verlassen."[210] Noch 1961 kam eine Studie aus der Neurologisch-Psychiatrischen Klinik der Universität Leipzig zur Frage Penicillin alleine oder in Kombination mit der Malariafieberkur zu ähnlichen Ergebnissen und Schlüssen. Der Erfolg der Kombination sei bei der progressiven Paralyse deutlich: Während in der Liquorsanierung die Penicillin-Malaria-Kombination (27 PatientInnen) gegenüber der Behandlung mit Penicillin alleine (24 PatientInnen) keinen Vorteil brachte, war die Kombination deutlich – in einem Verhältnis 1 : 3 – erfolgreicher „bei der Besserung im psychischen Befund und bei den guten sozialen Remissionen".[211] Zur eigenen Praxis aufgrund dieser Ergebnisse schreibt der

---

206 Telefongespräch von Gernot Heiss mit Dr. Miloš Vojtěchovský am 28.10.2014. Die Malariafiebertherapie sei in „akuten Phasen der Neurolues" gemacht worden und er könne sich noch an 3 bis 4 Fälle erinnern.
207 Der Leiter der Heilanstalt Pezinok, Karol Matulay, der als „Nestor der slowakischen Psychiatrie" gilt, empfahl 1957 Penicillin- und Malariafiebertherapie, aber auch Malariafiebertherapie alleine, je nach Art der Neurolues: MATULAY, Die Behandlung der Syphilis des Nervensystems mit Penicillin (1957), S. 180-182. Es konnte nicht festgestellt werden, ob damals in Pezinok nur PatientInnen mit Neurolues mit Malariafieber behandelt wurden.
208 Vgl. LANGE, Kritische Darstellung der Behandlungserfolge bei syphilitischen Erkrankungen des Zentralnervensystems (1954).
209 Kommentar von GRAGE, Penicillin allein oder in Kombination mit Fieber bei der Behandlung luischer Erkrankungen (1954), S. 301, zu GLAUBITZ, Penicillin allein oder in Kombination mit Fieber bei der Behandlung luischer Erkrankungen des Zentralnervensystems (1953), S. 505-507;
210 DEGKWITZ, Reicht bei der Neurolues in jedem Falle alleinige Penicillinbehandlung aus? (1955), S. 120.
211 ABEL, Katamnestische Untersuchungen zur modernen Therapie der Neurolues (1961), S. 425.

Autor: „Was die Kombination des Penicillins mit [Malaria]Fieber betrifft, so haben wir diese auf Grund eindrucksvoller Beobachtungen und entsprechender Literaturhinweise etwa von 1954 an in der Regel bei allen Patienten mit deutlicher psychischer Alteration angewandt, ausgenommen natürlich die Fälle, bei denen eine internistische Gegenindikation bestand.“[212]

In den eingesehenen Lehr- und Handbüchern der Psychiatrie von Anfang der 1960er Jahre wird die Penicillintherapie alleine, aber auch ihre Kombination mit der Malariafiebertherapie besprochen. Das lässt darauf schließen, dass ihre Anwendung akzeptiert und praktiziert wurde. Im Lehrbuch von Bleuler von 1960 heißt es im Abschnitt zur progressiven Paralyse, es sei „so frühzeitig als möglich entweder die Fiebertherapie [mit „Impfmalaria“ der *Malaria tertiana*] mit nachfolgender Salvarsan-Bismuth oder eine Penicillinbehandlung (oder Kombination beider) durchzuführen“.[213] Das renommierte französische Handbuch von Henry Ey, Paul Bernard und Charles Brisset beschrieb in seinen beiden Auflagen (1960, ²1963) die Malariafiebertherapie bei progressiver Paralyse an zweiter Stelle nach dem Penicillin ohne für eine Ausschließlichkeit von Penicillin oder für eine Kombination zu plädieren.[214] Das schmälere französische Handbuch zur Psychiatrie von Antoine Porot beurteilt in seinen drei Auflagen[215] Penicillin unterschiedlich: während 1952 die Skepsis gegenüber Penicillin überwog, galt 1960 Penicillin bereits als Therapie der ersten Wahl und es wurde die Kombination empfohlen. 1965 wurde die Ablehnung der Malariafiebertherapie zugunsten der ausschließlichen Therapie der progressiven Paralyse mit Penicillin als zu voreiliges und zu absolutes Urteil zurückgewiesen.[216]

In Wiener Publikationen wurde für die Kombination mit den in der Diskussion in *Der Nervenarzt* 1953 / 1955 und in der Leipziger Studie von 1961 beschriebenen Erfolgen „bei der Besserung im psychischen Befund und bei den guten sozialen Remissionen“ argumentiert. 1958, in einem Artikel über die psychiatrischen Therapien an der Wiener Klinik, schrieb Stefan Hift: „Die Malariakur der progressiven Paralyse in Kombination mit Penicillin scheint

---

212 Ebd., S. 426f.

213 BLEULER, Lehrbuch der Psychiatrie (^{10}1960), S. 231. Vgl. BLEULER, Lehrbuch der Psychiatrie (^{11}1969), wo er nur die Penicillintherapie ausführlich beschreibt, aber auf S. 231f. noch die Kombination mit einer Fieber- und nachfolgenden Bismuth-Salvarsan-Kuren in besonders resistenten Fällen empfiehlt. In der Züricher Klinik dürfte die Malariakur jedoch nur bis Ende der 1950er Jahre angewandt worden sein – vgl. TANNER/MEIER/HÜRLIMANN/BERNET, Zwangsmassnahmen in der Züricher Psychiatrie (2002), S. 93-95

214 EY/BERNARD/BRISSET, Manuel de Psychiatrie (^{2}1963), S. 719-721.

215 POROT, Manuel (1952, ^{2}1960, ^{3}1965).

216 Auch lt. MICHAUX, Psychiatrie (1965), S. 518, war die Kombination von Penicillin und Malariafiebertherapie 1965 für einige (französische) Psychiater noch die Regel.

uns der reinen antibiotischen Therapie überlegen. Wir wenden sie weiterhin in jedem Fall der Erkrankung an.“ [217] Hoff bestärkte im selben Jahr diese Auffassung: „Schließlich sei noch erwähnt, daß die neue Penicillinära die Malariafiebertherapie bei weitem nicht entwertet. Wie genauere Untersuchungen, besonders an meiner Klinik von Jech und Weingarten durchgeführt, zeigen konnten, sind die klinischen Heilungserfolge durch die Malariafiebertherapie denen der Penicillinbehandlung weit überlegen. Eine Kombination beider Methoden scheint die besten Resultate zu ergeben.“[218]

1960, in dem von Hoff herausgegebenen Sammelband „Therapeutische Fortschritte in der Neurologie und Psychiatrie“ schrieb Klara Weingarten (1909-1973)[219] ausführlich über die verschiedenen Formen der Neurolues und ihre Therapien, so auch zur Position der Wiener Klinik in der Diskussion Malariafieber- bzw. Penicillinbehandlung.[220] Sie berichtet über die Abwägung der Vorteile der Malariafiebertherapie gegenüber dem Penicillin, die in Wien zu einer anderen Meinung geführt habe, als bei anderen, insbesondere US-amerikanischen Autoren. Letztere, die sich für die alleinige Behandlung der Neurolues mit Penicillin aussprechen, würden sich allein auf die Liquoruntersuchungen stützen. In Wien würde jedoch „der Therapieerfolg in erster Linie nach der sozialen Anpassung und der Abnahme der klinischen Symptomatik beurteilt […] nicht allein nach der Liquorsanierung.“ So sei bei der alleinigen Penicillintherapie an der Klinik „selten eine Besserung des klinischen Bildes, besonders der Demenz, ganz im Gegenteil zu der Malariafiebertherapie festgestellt“ worden. Penicillin aber habe hervorragende Ergebnisse bei der Liquorsanierung.[221] Die „klinischen

---

[217] HIFT, Die klinische Lenkung der psychiatrischen Therapie (1958), S. 127-134.
[218] HOFF, Zum 100. Geburtstag Wagner-Jaureggs (1958), S. 4. Vgl. HOFF, Lehrbuch der Psychiatrie (1956), S. 124-126: „Auch heute noch ist die Malariafiebertherapie die beste und sicherste Behandlungsmethode. […] In den letzten Jahren wurde besonders in Amerika die Paralysebehandlung mit hohen Penicillindosen entwickelt. Es ist noch nicht ganz sicher, ob das Penicillin allein dieselbe Wirkung hat wie die Malariabehandlung und es bestehen in dieser Hinsicht Divergenzen zwischen amerikanischen und europäischen Autoren. Da die Malaria und das Penicillin verschiedene Angriffspunkte hat[!], glauben wir, daß die kombinierte Malaria-Penicillin-Behandlung die Methode der Wahl darstellt. Die Penicillininjektionen (täglich 500.000 E. i. m.) können während oder nach der Malariabehandlung gemacht werden. Die Gesamtdosis beträgt 15,000.000 bis 20,000.000 E. […] Wir machen die Penicillinbehandlung allein nur dann, wenn die Malariabehandlung kontraindiziert ist.“
[219] Vgl. https://de.wikipedia.org/wiki/Klara_Weingarten (30.10.2022). Lt. Eberhard Gabriel wurde sie Hoff sehr geschätzt.
[220] WEINGARTEN, Therapie der Neurolues (1960), S. 242-246. Lt. ebenda, S. 238-241 wurden in Wien je nach der Art der Neurolues verschiedene Therapien und Therapiekombinationen – auch ohne oder mit bzw. nur in hartnäckigen Fällen mit Malariafiebertherapie – angewandt: so bei „Gehirngumma […] Jodbehandlung […] in manchen Fällen ist es zweckmäßig, eine mildere Fiebertherapie anzuschließen, jedoch keine Malaria“; „Tabes dorsalis […] das Spirozid als fünfwertiges Arsenpräparat […]. Wenn es auf diese Weise nicht gelingt, den Liquor zu sanieren, wird auch hier zur Fiebertherapie gegriffen“. Bei der progressiven Paralyse sei die Malaria-Penicillin-Kombination jedoch die Standardtherapie. Sie übernahm vieles aus ihrem in einzelnen Details etwas ausführlicheren Artikel von 1957: WEINGARTEN, Zur Therapie der Neurolues (1957), S. 722-725.
[221] WEINGARTEN, Therapie der Neurolues (1960), S. 243.

Zeichen“ würden „dann am besten durch Penicillin beeinflußt […], wenn die degenerativen Veränderungen im Zentralnervensystem gegenüber den entzündlichen in den Hintergrund treten; deswegen werden wir bei bestimmten benignen Formen der Neurosyphilis mit Penicillin allein befriedigende Resultate erreichen, während bei den parenchymatösen Formen die Kombination von Malaria und Penicillin, gleichzeitig oder hintereinander verabreicht, die Methode der Wahl darstellt.“ Der Erfolg sei freilich davon abhängig, wie viel vom Nervengewebe bereits zerstört ist.[222]

In Wien hatte die Malariafiebertherapie nicht nur an der ‚Klinik Hoff' weiterhin Fürsprecher.[223] Hans Asperger, ab 1962 Leiter der Universitätskinderklinik in Wien, schrieb in seinem Standardwerk zur „Heilpädagogik“ von der 1. Auflage 1952 bis zur 5. Auflage 1968[224]: „die Therapie der Wahl […] bei jedem ersten Zeichen kindlicher Neurolues“ – erfolgversprechend besonders „bei der Encephalomeningitis luetica“, wenig „bei der juvenilen PP“ – sei „eine Malariakur mit anschließender energischer antiluetischer Behandlung“. Erst ab der 3. Auflage von 1961 fügte er zur ‚anschließenden antiluetischen Behandlung' die Präzisierung bei: „heute ja vornehmlich mit Penicillin“.[225] An der Kinderstation der ‚Klinik Hoff' wurden lt. Datenbank nur zwei Patientinnen mit progressiver Paralyse mit Malariafiebertherapie behandelt: 1955 die 13jährige Patientin NK487 mit dem Diagnoseeintrag „P. P.? (neg)“ und 1960 die 11jährige Patientin NK1236 mit „juvenile p. P.“[226] 1963 wurde der 13jährige Patient NK1736 mit der Diagnose „Epi, Lu cerebri“ mit Typhusvakzinen zur Fieberprovokation und Penicillin behandelt; andere Kinder mit Lues cerebri bzw. kongenitaler Lues bekamen keine Fiebertherapie. Im Gegensatz dazu waren an den Erwachsenenstationen in den 1950er Jahren die PatientInnen mit progressiver Paralyse und Malariakur noch häufig. Möglich, dass damals Kinder mit luetischen Erkrankungen an der bis 1961 von Karl Kundratitz (1889 – 1975) geleiteten Kinderklinik (mit

---

222 Ebd., S. 245.

223 So sei lt. Peter Berner (Bemerkung gegenüber Eberhard Gabriel) nicht nur Hoff, sondern auch Herbert Reisner (Ende der 1950er Jahre, GH) – damals Leiter der Neurologie am Rosenhügel – für eine Kombinationsbehandlung der PP mit Malariafiebertherapie und Penicillin eingetreten. (Gespräch Eberhard Gabriel mit Gernot Heiss am 13.5.2013.) Darauf verweist auch der Fall des 60jährige Patienten N107 mit „Anfall-PP“, dessen Malariakur am Rosenhügel begonnen wurde: zu ihm siehe oben S. 57 Anm. 177 und S. 58 Anm. 180.

224 ASPERGER, Heilpädagogik ([1]1952, [2]1956, [3]1961, [4]1965, [5]1968).

225 Wobei er wegen der ‚Herxheimer-Reaktionen' einen vorsichtigen Beginn empfahl; ASPERGER, Heilpädagogik ([3]1961), S. 100.

226 Bei ihrem ersten Aufenthalt an der Station 5 Monate früher bekam sie eine Fieberkur mit Typhus-Vakzinen und Vitaminen.

malariainfiziertem Blut aus der ‚Klinik Hoff‘[227]) behandelt wurden; Kundratitz hatte 1924 die Malariakur als erster bei Kindern mit kongenitaler Lues eingeführt und dazu seit 1925 publiziert.[228]

Aus den PatientInnenakten der Erwachsenenstationen ist zu ersehen, dass Malaria- gemeinsam mit Penicillinkur auch an der Wiener Klinik bereits 1950 angewandt wurde, wenn auch nicht standardmäßig:

> Der 48jährige Privatpatient N8 mit in der Diagnosezeile „Lues Cerebri, Malariakur" bekam Ende 1950 nach seiner Aufnahme zuerst innerhalb von 9 Tagen 10.000.000 E „Pen. Depot" i. m., dann feierte er Silvester zu Hause und wurde am Tag seiner Rückkehr mit malariainfiziertem Blut geimpft; gleichzeitig erhielt er an 4 Tagen insgesamt 4.800.000 E „Pen. Depot."[229]

Anfang der 1950er Jahre wurden in der Kombination 10 – 15 Mill. Einheiten Penicillin gegeben, gegen Mitte der 1950er Jahre wurde die Dosis auf 25 Mill. Einheiten gesteigert.

Diese Steigerung von 1952 bis 1954 ist in zwei Fällen zu erkenne, deren Behandlungsverlauf außerdem zeigt, wie Diagnose und Therapie an neue Krankheitsbilder angepasst wurden. So führte das Auftreten von paranoiden Zustandsbildern zum Wechsel von der Malariafieber- zur Elektrokrampftherapie:

> Der 42jährige Patient N37 („P.P., paranoid-halluzinatorisches Bild"; im Akt werden auch „*schizophrene Symptome*" erwähnt) bekam 1952 nach einer Penicillintherapie mit 16 Mil. Einheiten eine Malariafiebertherapie; beim zweiten Fieberschub trat „ein paranoid-halluzinatorisches Bild" auf, die Fiebertherapie wurde abgebrochen und nach der Chininbehandlung folgte eine EKT mit 6 Anwendungen.
>
> Der 30jährige Patient N82 bekam 1954 eine Penicillintherapie mit 25 Mill. Einheiten Penicillin, dann ein Malariakur, die – so der Arztbrief – „nach dem zweiten Fieberanfall abgebrochen" wurde, da es „zum Auftreten eines schweren paranoid-halluzinatorischen Zustandsbildes" gekommen war; dem folgte eine EKT mit 9 Anwendungen.[230]

[227] Vgl. S. 151 Anm. 565.
[228] GRÖGER, Röntgen- und Malariatherapie. Zur Therapie des kindlichen Schwachsinns (2019), S. 166f. Zum Beginn 1924 und zu Kundratitz und der Anwendung der Malariakur bei „zerebralgestörten Kindern", vgl. unten S. 91.
[229] Als Beispiele zum frühen Einsatz und zur Dosierung vgl. 1952 den Fall des 51jährigen Patienten N45 mit der Diagnose „incipiente P.P." und jenen des 68jährige Privatpatient N43 mit der Diagnose „Tabo-paralyse": beide erhielten parallel zu einer Malariakur eine Penicillinkur mit insgesamt 15,000.000 Einheiten. „Wegen des cardialen Zustandes" als Kontraindikation wurde 1950 im Fall des 61jährigen Patienten N975 mit der Diagnose „Lues cerebri" nur eine „Penicillinkur mit insgesamt 10 Mill. E" und danach „eine Neo S[alvarsan]-Vismut Kur" durchgeführt. KOLLE, Psychiatrie (1955), S. 227-229, führte die Wiener Klinik mit 8 bis 15 Millionen Einheiten, wenn notwendig mit einer Wiederholung nach 8 Wochen, als Beispiel an und schrieb: „Besonders vorsichtige Ärzte verwenden übrigens zur Zeit noch die kombinierte Behandlung Malaria + Penicillin." Kurt Kolle war 1952 bis 1966 Ordinarius und Leiter der Universitätsklinik für Psychiatrie an die Universität München.
[230] Der Eintrag in der Diagnosezeile auf seinem Patientenakt „Paranoid-halluzinatorisches Bild bei P.P." die Praxis, dass die Diagnose-Eintragungen oft erst kurz vor der Ablage des Aktes erfolgten.

Parnoid-halluzinatorische Bilder konnten lt. Klara Weingarten bei ParalytikerInnen mit einer schizophrenen Veranlagung durch das Malariafieber ausgelöst werden und – so wie bei der „sogenannten Tabespsychose“ – wurde hier „gelegentlich“ die Elektrokrampftherapie angewandt.[231]

Von 175 Fällen, in denen Neurolues-PatientInnen der Datenbank eine Malariatherapie bekamen, wurden in 68 Fällen die PatientInnen bereits nach der Injektion von malariainfiziertem Blut an die *Heil- und Pflegeanstalt Steinhof* verlegt,[232] wodurch aus den Klinikakten eine Parallelbehandlung mit Penicillin weder auszuschließen, noch zu belegen ist. Neun der 107 PatientInnen, die während der Malariakur an der Klinik weiterbehandelt wurden, bekamen lt. Fieberkurve bei ihrem Aufenhalt keine Penicillintherapie. In zwei weiteren (späten) Fällen ist nachzuweisen, dass vor der Malaria- anderswo eine Penicillinkur gegeben worden war.[233] Anders als bei PatientInnen mit nicht-luetischen Diagnosen wurden einige der an Syphilis erkrankten PatientInnen zweimal mit Malariafiebertherapie behandelt (11 von 175).[234]

---

[231] WEINGARTEN, Therapie der Neurolues (1960), S. 247f. schrieb zur Anwendung der EKT bei Neurolues-Patienten in „den seltenen Fällen von paranoid-halluzinatorischen Bildern, die während der Malariafiebertherapie auftreten, wobei wir annehmen, dass eine schizophrene Veranlagung der Patienten als Grundlage für diese Erscheinung dient. Auch bei der sogenannten Tabespsychose werden wir gelegentlich die [E-]Schocktherapie anwenden.“

[232] KAINZ, Retrospektive Datenanalyse iatrogener P. vivax Infektionen (2019), S. 36. Diese raschen Überweisungen gab es in den ausgewerteten Akten nur bei Neurolues-PatientInnen. Ich verdanke Frau Rosa Kainz im Gespräch und ihrer medizinischen Diplomarbeit über die Malariafiebertherapie (Verlauf) in der ‚Ära Hoff‘ aus tropenmedizinischer Sicht wichtige Detailinformationen zur Fiebertherapie bei Neurolues-PatientInnen.

[233] Die Patientin N190 1965 und der Patient N127 1967 (beide mit der Diagnose „P.P.“) hatten vor der Malariatherapie am *Steinhof* bzw. an der Hautklinik eine Penicillintherapie erhalten. Erstere, die 1964 am *Steinhof* und 1965 eine „ambulante Pen.Kur (21 Mill.)“ bekam und auf Empfehlung von „Frau Prof. Weingarten“ nun zur Malariaimpfung an die Klinik kam, wurde am 2. Tag nach der Malariaimpfung in die *Heil- und Pflegeanstalt Steinhof* transferiert; letzterer wurde nach der Malariatherapie noch zwei Monate an der Klinik weiterbehandelt.

[234] KAINZ, Retrospektive Datenanalyse iatrogener P. vivax Infektionen (2019), S. 49. Vgl. die 1904 geborene Patientin N182 „Progressive Paralyse“: sie bekam in einem Abstand von 33 Monaten (Februar 1953; November 1955) 2mal eine Malariafieberkur. Beim 1. Mal hatte sie 8 Fieberschübe, wobei jene vom 3. bis zum 7. über 40° erreichten. Beim 2. Mal stieg das Fieber bei den ersten 3 Schüben nur auf um 39°, beim 4. Schub auf ca. 39,7°, beim 5. Schub auf ca. 40,5°; um den 6. und 7. (letzten) Fieberschub zu verstärken, wurden Typhus Vakzine injiziert, das Fieber blieb dennoch auf unter 40°. Daraufhin wurde Chinin+Atebrin zum Abbruch der Malariakur gegeben. Mit Penicillin (15,000.000 E) wurde die Patientin bereits bei ihrem 1. Aufenthalt 1950 behandelt, ebenso (wieder mit 15,000.000 E) während ihres 2. Aufenthalts 1953 parallel zu ihrer 1. Malariakur, sowie 3 Monate später 1953 in einem anderen Krankenhaus oder zu Hause – so eine Beilage – mit 10,000.000 E). Vor der Einführung von Penicillin dürfte eine zweite Malariakur häufiger gewesen sein, bei der Wiederholung blieb die Infektion oft „nach einer gewissen Anzahl von Fieberanfällen stecken“, so KAUDERS, Zur Klinik, Theorie und Geschichte der Malariabehandlung (1948), S. 55.

Anfang der 1950er Jahren wurde die Malariafiebertherapie auch noch ohne Penicillin – weder parallel noch nach- oder vorher – gegeben. Nach der Entlassung wurde in diesen Fällen „eine kombinierte Salvarsan-Wismuthkur“[235], eine „Neosalvarsan“-Behandlung,[236] eine „antiluetische Behandlung mit kombinierten Kuren“[237] angeschlossen.[238]

> Zur antiluetischen Nachbehandlung wurde 1951 dem 55jährigen Patientin N137 mit der Diagnose „p.P.“ im Entlassungsbrief „eine kombinierte Neosalvarsan-Bismogenol-Kur“ empfohlen.[239]
>
> Auch 1952 im Fall der 23jährigen Patientin N146 mit der Diagnose „P.P. incipiens“ wurde diese Behandlung im Arztbrief am Tag der Entlassung angesprochen: „Es handelt sich somit um Paralysis incipiens nach Malariakur, es wäre nach interner Kontrolle, sobald es der Zustand der Leber erlaubt,[240] kombinierte Salvarsan-Wismuth-Kur erforderlich“.
>
> Bei der 1954 28jährigen Patientin N172 mit der Diagnose „Conjugale progr. Paralyse“ hieß es im Arztbrief: „Nach der Malariakur war der Liquor, was die Zellzahl betrifft, weitgehend saniert, der WaR natürlich weiterhin positiv. […] Wir empfahlen bei Pat. wegen des positiven WaR noch eine komplette antiluetische Behandlung durchzuführen und sie in 3 Monaten zur Kontrollpunktion zu uns zu schicken.“ Hier ist vermutlich eine Salvarsan-Wismuth-Kur und nicht – außerhalb der Klinik – eine Penicillinkur gemeint.

In seinem Lehbuch der Psychiatrie schrieb Hoff 1956: „Jeder Malariabehandlung muß eine komplette spezifische Behandlung in Form einer Salvarsan-Wismut-Kur folgen. Bei der kombinierten Malaria-Penicillin-Kur wird[!] das Salvarsan und Wismut entbehrlich.“[241]

Nach der Mitte der 1950er Jahre dürfte sich bei progressiver Paralyse und bei Taboparalyse zwar die Malariafiebertherapie in Kombination mit Penicillin an der Klinik weitgehend

---

[235] 1951 bei der 55jährigen Hilfsarbeiterin N145 mit der Diagnose „p.P.“ „mit deutlicher Demenz“.
[236] 1951 wurde beim 37jährigen Studenten N22 mit der Diagnose „P.P. (hypochondrisches Bild)“ lt. Patientenakt nachweislich kein Penicillin gegeben, sondern nach der Malariakur „mit Neo-Salvasan begonnen“.
[237] 1952 beim 23jährigen Patienten mit N39 mit der Diagnose „p.p. imminens“.
[238] In einigen der Patientenakten finden sich dazu Informationen, in anderen weder positive noch negative Hinweise. Wie auch zu anderen Fragen müssen auch hier aus den wenigen Hinweisen Schlüsse gezogen werden.
[239] Zur Therapie hieß es hier: „sie machte eine Malariafieberkur mit neun Fieberstössen durch. […]. Nach der Malariakur Besserung des neurologischen Status und der Vergesslichkeit, auch im Liquor ist bereits eine geringe Besserung in den Eiweißwerten und leichter Zellenvermehrung eingetreten. […]. Ferner wäre eine kombinierte Neosalvarsan-Bismogenol-Kur, wenn intern nicht gegenindiziert, möglichst gleich anschließend an die Fieberkur erforderlich. Wir würden mit 0,15 Neosalvarsan einmal wöchentlich 3mal hintereinander und dann mit 0,3 Neosalvarsan Behandlung empfehlen, ferner 2mal wöchentlich 1 ½ ccm Bismogenol. Nach Beendigung der Kur oder auch schon nach 2 Monaten wäre eine neuerliche Lumbalpunktion, die bei uns durchgeführt werden könnte, zu empfehlen.“
[240] Davor gab es Hinweise auf Leber- und Milzprobleme der Patientin.
[241] HOFF, Lehrbuch der Psychiatrie (1956), S. 126.

durchgesetzt haben, die Neurolues-Spezialistin der Klinik Klara Weingarten lässt jedoch in ihrem Artikel 1960 die Möglichkeit einer Therapie ohne Penicillin weiterhin offen.[242]

Nach den vorliegenden Akten gab es freilich Ende der 1950er Jahre auch an der Wiener Klinik de facto kaum noch PatientInnen mit einer Neurolues-Diagnose und dementsprechend kaum noch Fälle von Malariafiebertherapie bei diesen Diagnosen. Lt. Datenbank verteilen sich die 371 PatientInnen mit einer Neurolues-Diagnose und die 163 PatientInnen, die mit einer Neurolues-Diagnose eine Malariafiebertherapie bekamen (44,0 %), über die Jahre folgendermaßen:

Jahr	Neurolues-PatientInnen insgesamt	Malariafiebertherapie erhielten von diesen Neurolues-PatientInnen (s. oben Tab. 11)
1951	65	35
1952	57	27
1953	41	24
1954	44	22
1955	30	10
1956	21	6
1957	22	7
1958	16	5
1959	5	0
1960	8	4
1961	13	9
1962	1	0
1963	11	2
1964	13	4
1965	5	3
1966	6	2
1967	9	3
1968	3	0
1969	1	0
Summe	371	163

Abb. 2 Anzahl der Fälle mit Neurolues und der mit Malariafiebertherapie behandelten Neurolues-PatientInnen.

[242] WEINGARTEN, Therapie der Neurolues (1960), S. 237, S. 247 und passim. Erstaunlich ist die Argumentation des rumänischen Malariologen LUPASCU, Applications actuelles de la malariathérapie (1974), S. 166, aus dessen Vortrag zur Aufrechterhaltung der Malariafiebertherapie im Interesse der Malariaforschung noch mehrmals zitiert werden wird, mit der Befürchtungen einer Resistenzentwicklung von T. pallidum gegenüber dem Penicillin, denn Beobachtungen dazu sind ausgeblieben. Anders ist es mit Penicillin-Unverträglichkeit, deretwegen 1967 der 42jährige Patient N126 mit der Diagnose „Progressive Paralyse“ vom Wilhelminenspital zur Malariatherapie an die Klinik kam.

Insgesamt wurden 371 Neurolues-PatientInnen länger als 4 Tage an der Klinik aufgenommen. In den Jahren 1951-1953 war diese Diagnose die häufigste, bei der eine Malariafiebertherapie angewandt wurde.[243] Die 163 Neurolues-PatientInnen mit Malariafiebertherapie waren fast ausschließlich Fälle von progressiver Paralyse (21mal „Tabo-P.P.“, 2mal „Tabes dorsalis“[244]).

Die Gliederung des Untersuchungszeitraumes in zwei Teile zu je 9 Jahren zeigt deutlich die Abnahme der Neurolues-Fälle:[245] In den Akten finden sich von 1951 bis 1959 noch 301 Fälle ($\bar{x}$ = 33,44 Fälle pro Jahr), davon bekamen 136 PatientInnen eine Malariafiebertherapie (in 45,16 % der Fälle, $\bar{x}$ = 15,11 Fälle pro Jahr). In diese erste Periode von 9 Jahren fällt 1959, in dem nur 5 PatientInnen mit Neurolues aufgenommen wurden und keine/r eine Malariakur bekam: es war das Jahr, in dem der Malariastamm erlosch und erst wieder mit malariainfiziertem Blut aus den Tropeninstituten Hamburg und Amsterdam – nach der Wiener Praxis bei nicht-luetischen Patienten – erneuert werden musste. In den folgenden 9 Jahren von 1960-1968 hatten 70 PatientInnen eine Diagnose aus dem Diagnosefeld Neurolues ($\bar{x}$ = 7,77 Fälle pro Jahr, davon jedoch 1962 und 1968 kein/e Einzige/r), 27 davon bekamen eine Malariakur (in 38,57 % der Fälle; $\bar{x}$ = 3 Fälle pro Jahr). Das 19. und letzte Jahr des Untersuchungszeitraums (1969) wurde hier nicht einbezogen, da es in diesem Jahr nach den vorliegenden Akten weder eine Malariafiebertherapie bei einer anderen Diagnose, noch bei einer/em Patientin/en mit Neurolues gab. Die Prozentanteile der Malariafiebertherapien bei PatientInnen mit neuroluetischen Erkrankungen zeigen, dass nicht nur die Anzahl der Neurolues-PatientInnen in den 1960er Jahren rückläufig war, sondern auch die Häufigkeit, mit der eine Malariafiebertherapie bei neuroluetischen Erkrankungen angewandt wurde. Zu diesem Rückgang dürfte auch die Anwendung von Penicillin alleine bei Fällen mit geringen klinischen Symptomen beigetragen haben.

2.1.4.2 Malariafiebertherapie bei nicht-luetischen Erkrankungen in der wissenschaftlichen Diskussion und Praxis

Die Malariafiebertherapie der progressiven Paralyse hatte als somatische Therapie einer psychischen Erkrankung weitreichende Folgen für die Entwicklung der Psychiatrie: Während es bisher in den ‚Pflege-Anstalten‘ vor allem um die langfristige Versorgung unheilbar-

[243] Vgl. die Tab. 11, oben S. 60.
[244] Die beiden Patienten mit „Tabes dorsalis“ (1950 und 1957) wurden mit Malariakur+Penicillin behandelt, während Taboparalyse lt. Datenbank meistens nur mit Malariafiebertherapie behandelt wurde.
[245] Vgl. zum Folgenden die etwas andere Aufschlüsselung der Häufigkeitsverteilung oben S. 36f.

Erkrankter ging, veränderte sich durch die Aussicht auf Heilung nun das Selbstbild der PsychiaterInnen als aktiv behandelnde Ärzte/innen und damit ihre Einstellung gegenüber den PatientInnen.[246] Aufgrund des Erfolgs der Malariafiebertherapie bei progressiver Paralyse wurde in der Psychiatrie intensiv nach weiteren körperlichen Therapien gesucht. Das führte zur Entwicklung in den 1930er Jahren der Koma- und Krampftherapien, im nächsten Jahrzehnt der neurochirurgischen Lobotomie und in den 1950er Jahren der neuen medikamentösen Therapien. In der Wendung zu körperlichen Therapien wurde die Malariafiebertherapie seit den 1920er Jahren auch bei anderen psychiatrischen Erkrankungen versucht.[247] Diese Anwendungen außerhalb der Neurolues sind Thema der folgenden Zeilen als Einleitung für die speziellen Abschnitte zur Malariakur und ihren therapeutischen Zielen bei den nicht-luetischen Diagnosen. Als Quellen dienen die Datenbank für den Wiener Gesamtüberblick, sowie Hinweise in zeitgenössischen und auch in rückblickenden Fachpublikationen, und vereinzelt Erinnerungen von Zeitzeugen.

In Wien wurde die Malariafiebertherapie im Untersuchungszeitraum (1951-1969) vorwiegend bei anderen Diagnosen als der progressiven Paralyse angewandt. Es waren jugendliche Patienten und nur selten jugendliche Patientinnen.[248] Auch an der Kinderstation[249] hatten von 35 überlieferten Fällen, in denen PatientInnen eine Malariafiebertherapie bekamen, 33 PatientInnen nicht-luetische Erkrankungen. Das Geschlechterverhältnis war mit 25 Knaben und acht Mädchen ungleich aber doch deutlich ausgeglichener als bei den ab 14-jährigen der Erwachsenenstationen (693:13[250]). Im Folgenden geht es um die zeitgenössischen Erwähnungen der Malariafiebertherapie als Indikation und die Befundung für ihre Anwendung bei nicht-luetischen psychischen Erkrankungen; im Fall der Anwendung bei der

[246] Vgl. die Ergebnisse einer textkritischen Analyse von PatientInnenakten der Zeit, BRASLOW, Effect of Therapeutic Innovation on Preception of Disease and the Doctor-Patient Relationship (1995), S. 660: “Before the introduction of malaria fever therapy, physicians saw their neurosyphilitic patients as ‘hopeless’, ‘immoral’, and ‘stupid’ paretic-objects to be acted upon, a view consistent with the cultural belief that syphilitic patients were sinful and depraved. After the introduction of malaria fever therapy, doctors wrote more positively and empathically about their neurosyphilitic patients, allowing patients to become active participants in their therapeutic regiments. Patients with neurosyphilis voluntarily sought admission specifically for fever therapy, seeing the asylum as a place of cure rather than as an institution of confinement.” Ähnlich in der Zusammenfassung des ausführlicheren Artikels von BRASLOW, The Influence of a Biological Therapy on Physicians’ Narratives and Interrogations: The Case of General Paralysis of the Insane and Malaria Fever Therapy, 1910-1950 (1996), S. 607f.
[247] Vgl. WAGNER-JAUREGG, Über spezifische und unspezifische Behandlung von Geisteskrankheiten (1931), S. 291f., mit der Forderung nach Untersuchungen der Wirksamkeit bei diesen Diagnosen, zitiert unten S. 78.
[248] Der Altersdurchschnitt aller Malariafiebertherapie-PatientInnen der Erwachsenenstationen ohne jene mit Progressiver Paralyse war 21,7 Jahre.
[249] Vgl. Tabelle in GEIGER, Kinderstation (2015), S. 253f.
[250] Vgl. oben S. 62f.

Diagnose Psychopathie geht es auch um die Diskussion zu dieser Diagnose. Publikationen der Zeit und einzelne Erinnerungen von Ärzten, die in diesen Jahren an psychiatrischen Anstalten tätig waren, gaben dazu die spärlichen Informationen. Vor diesem Hintergrund ist die Anwendung an der Wiener Klinik in der ‚Ära Hoff' nach den Ergebnissen der Auswertung der PatientInnenakten zu diskutieren.

Für diese Diagnosen, die bzw. deren Symptome in Wien zur Indikation einer Malariafiebertherapie führten, fanden sich nach 1950 in Fachpublikationen nur wenige Hinweise auf ihre Anwendung und kaum auf Begründungen für die Anwendung sowie auf die erwartete Wirkung. Trotz der Häufigkeit ihrer Anwendung in der ‚Ära Hoff' und der sonst üblichen Publikationstätigkeit der KlinikerInnen, fehlen hier die klärenden wissenschaftlichen Publikationen aus der Zeit. Begründungen finden sich auch in den PatientInnenakten der Erwachsenenabteilungen nur selten und nur andeutungsweise. Waren es Anwendungen, die aus den 1920er Jahren stammten[251] und die fortgesetzt angewandt, aber kaum oder gar nicht mehr begründet wurden? Oder waren es Anwendungen, um den Malariastamm zu erhalten? Und wenn ja, stellt sich die Frage wozu, da die progressive Paralyse in den 1960er Jahren kaum noch vorkam und nach Meinung vieler Ärzte/innen mit Penicillin gut zu behandeln war?[252]

Rückblickend auf die Zeit vor 1945 stellen die Medizinhistoriker Hans-Walter Schmuhl und Volker Roelcke 2013 zur vielfältigen Anwendung der Malariafiebertherapie in der Psychiatrie fest, dass „die Fieber- und [die] Dauerschlafbehandlung, die für Patienten mit Progressiver Paralyse gedacht waren, schon bald auf Patienten mit Schizophrenie oder bipolarer Störung, ja selbst auf ‚Psychopathen' übertragen" wurden. Ähnlich habe es sich mit den „Koma- und Krampftherapien" verhalten, die als Mittel gegen Schizophrenie entwickelt, bald aber auch

[251] In *Der Nervenarzt* in der Ausgabe von 1930 finden sich beispielsweise mehrere Besprechungen von Publikationen der letzten Jahre zur Anwendung der Malariafiebertherapie bei „nicht-luetischen Geisteskrankheiten" mit oder ohne Erfolg, an italienischen, deutschen und polnischen Kliniken: so bei Epilepsie, manisch-depressivem Irresein, Involutionsmelancholie, „Dementia praecox", bei „Akinetisch-Hypertonischen [Patienten] (zum Teil mit Charakterveränderungen)", „postencephalitschen Zuständen", multipler Sklerose, und vor allem bei Schizophrenie. Weiters wird eine Studie aus der Wiener Klinik zum erfolgreichen Einsatz von Pyrifer besprochen, deren Autoren (A. MANDL/O. SPERLING) „das Pyrifer für ein wertvolles, in dem therapeutischen Effekt ‚nur hinter der Impfmalaria' zurückstehendes und unschädliches Mittel zur künstlichen Fiebererzeugung bei allen Nervenkranheiten [halten], bei denen eine Fieberbehandlung in Frage kommt und eine Malariakur kontraindiziert erscheint oder erfolglos geblieben ist." Sie berichten über die Behandlung von insgesamt 70 Patienten mit PP (14), Lues cerebrospinalis (11), Tabes dorsalis (13), mit multipler Sklerosis (15), postenzephatischem Parkinsonismus (13), Schizophrenie (3) und Malariaenzephalitis (1): MANDL/SPERLING, Ergebnisse und Indikationen der Fieberbehandlung mit Pyrifer bei Nervenkrankheiten (1929), S. 169-172.
[252] Vgl. die Diskussion im Exkurs zu den „Stammträgern" und in den Schlussbemerkungen.

bei Manien, Depressionen, Neurosen u. a. eingesetzt wurden. Diese Praxis des „off-label-use" führen die Autoren auf eine „neue Aufgeschlossenheit zum therapeutischen Experiment" zurück, die sich seit dem 1. Weltkrieg in der Psychiatrie als „ärztliche Grundhaltung" verfestigt habe.[253] Wagner-Jauregg reagierte 1931 mit der Aufforderung zur wissenschaftlichen Untersuchung der Wirkung der Malaria- und anderer Fiebertherapien, wie sie nun „bei anderen Psychosen, besonders bei Fällen von manisch-depressivem Irresein und Schizophrenie" angewandt würden, mit der Methode des Simultanvergleichs.[254] Zur breiten Anwendung schrieben Henri Claude (Leiter der Pariser Universitätsklinik Hôpital St. Anne) und Pierre Rubinovitch 1940: ‹ Die Fiebertherapie, die zuerst fast ausschließlich für die Behandlung der P.P. reserviert war, wurde zunehmend bei fast allen psychischen Störungen versucht. Es scheint, dass die große Gruppe der Verwirrungssyndrome, der schizophrenen Erkrankungen am meisten profitierten.›[255] Die Malariafiebertherapie war für Claude jedoch weniger erfolgversprechend als andere Fiebertherapien.[256]

In österreichischen Publikationen finden sich Hinweise auf die Anwendung der Malariafiebertherapie bei nicht-luetischen neurologischen und auch bei dermatologischen und anderen Krankheiten in der Zwischenkriegszeit und in einigen wenigen Fällen bis Ende der 1950er Jahre: Von Dermatologen wurde in den 1930er und 1940er Jahren von Erfolgen der Malariafiebertherapie in der Behandlung der Gonorrhoe berichtet. [257] Otto Kauders berichtet 1948, dass in den 1920er Jahren „die verschiedenartigsten Erkrankungen mit Malaria behandelt wurden. So wurde Epilepsie, Schwachsinnsformen, die große Gruppe der Schizophrenien, die meisten chronischen Erkrankungen des Zentralnervensystems, aber auch luetische Gefäßleiden, wie die Mesaortitis, Diabetes und sogar Karzinom mit Malaria behandelt."[258] Auf drei Anwendungen bei nicht-luetischen neurologischen Erkrankungen geht

[253] SCHMUHL/ROELCKE, Einleitung (2013), S. 23-24.
[254] WAGNER-JAUREGG, Über spezifische und unspezifische Behandlung von Geisteskrankheiten (1931), S. 291f. Zu dieser Methode vgl. unten im Kapitel über die Insulinkomatherapie S. **188f.** Anm. 722 und 723.
[255] Übersetzungen aus dem Französischen (von GH) werden mit einfachen Guillemets gekennzeichnet.
[256] CLAUDE/RUBINOVITCH, Thérapeutiques biologiques des affections mentales (1940), S. 66. In einem Artikel gemeinsam mit Jean Dublineau 1935 ging es Claude bereits um andere Fiebertherapien, vor allem um "l'association sulfochrysothérapeutique": CLAUDE/DUBLINEAU, Résultats de la pyrétothérapie dans 34 états dit de démence précoce (1935), S. 553-583. Claude dürfte in Frankreich Schule bildend gewesen sein, vgl. MICHAUX, Psychiatrie (1965), S. 1006.
[257] Vgl. über die Anwendung an der Innsbrucker Klinik, SCHWARTZ, Zur Frage der Malariatherapie der Gonorrhoe [1941]. Vgl. mehrere Kurznachrichten in der *Wiener Klinischen Wochenschrift* und der *Wiener Medizinischen Wochenschrift* der 1930er Jahre. Vgl. die Studie des Königsberger Klinikers RAU, Behandlung der Gonorrhöe[!] im Kindesalter mit Impfmalaria (1930), 121–124, mit Literaturzitaten aus deutschsprachigen Publikationen von 1927 und 1929.
[258] KAUDERS, Zur Klinik, Theorie und Geschichte der Malariabehandlung (1948), S. 66.

er ausführlicher ein:[259] bei Multipler Sklerose,[260] progressiver Muskeldystrophie und bei spinaler Kinderlähmung (Poliomyelitis). Unter den 35 PatientInnen, die nach der Datenbank zur Kinderstation eine Malariafiebertherapie erhielten, fanden sich vier Fälle mit progressiver Muskeldystrophie (morbus Erb)[261] und drei Fälle mit „Zust[and] nach Poliomyelitis".[262] Mit der Diagnose „status post Poliomyelitis" bekam auch auf der Erwachsenenstation 1951 der 33-jährige Patient X226 eine Malariafiebertherapie.

In der Nachbehandlung der Polio hat Kauders selbst die Malariakur als Behandlungsmethode eingeführt und er nennt hier Berichte mehrerer Autoren über Erfolge. Kauders skizziert seine Überlegungen, die ihn zur Erweiterung des Anwendungsspektrums der Malariakur auf Lähmungserscheinungen führten, die mit der Polio einhergehen: Diese Anwendung zeige, wie sehr eine Therapie, wenn sie nicht empirisch, sondern klinisch und theoretisch „gründlich […] durchgearbeitet ist, neue therapeutische Möglichkeiten zu erschließen vermag" – auch wenn die Krankheit „sowohl klinisch wie ätiologisch fernab von der Paralyse liegt".[263] Die Anwendung in der genannten Nachbehandlung von Schädigungen durch die Kinderlähmung, wie sie Kauders empfahl, wurde in den 1950er Jahren in Wien nicht nur an der Klinik praktiziert.[264] Diese Anwendungen erforderten „luesfreies" Malariablut und verweisen auf einen luesfreien Malariastamm bereits in der Zwischenkriegszeit.[265]

---

[259] 1926 veröffentlichte er mit gemeinsam mit HOFF/KAUDERS, Über die Malariabehandlung der Tabes dorsalis (1926), S. 306-322.
[260] Vgl. die Wiener KlinikerInnen SCHINKO/SLUGA-GASSER/TSCHABITSCHER, Therapie der Multiplen Sklerose (1960), S. 57: die Malariafiebertherapie und auch andere Fieberbehandlungen würden bei Multipler Sklerose „heute allgemein abgelehnt".
[261] 1950 ein 8jähriger (XK573), 1954 eine 15jährige (XK313), 1955 eine 11jährige (XK541) und 1956 ein 8jähriger (XK1942). Danach wurden mehrere Patientinnen mit dieser Diagnose nur mehr ohne Malariafieber behandelt.
[262] 1953 eine 13jährige (XK106), 1954 ein 10jähriger (XK310) und 1956 ein 4jähriger (XK582).
[263] KAUDERS, Zur Klinik, Theorie und Geschichte der Malariabehandlung (1948), S. 68. Vgl. KAUDERS, Über die Anwendung der Malariabehandlung im Anschluß an das akute Lähmungsstadium bei Poliomyelitis epidemica (1936), S. 1729-1732 und S. 1766-1769, und KAUDERS, Weitere Mitteilung über die Malariabehandlung bei Poliomyelitis (1937), S. 1464-1468 und S. 1502-1506.
[264] Vgl. KLARE, Rehabilitation und Poliomyelitis (1960), S. 179. KLARE/FURTENBACH, Erfahrungen und Erfolge in der Behandlung der Poliomyelitis (1948), S. 380. Viktor Klare war am Wilhelminenspital 1946 bis 1974 Vorstand der Physikalischen Therapie. Herr P. S., der damals 22 Jahre alt war, erzählte 2014 dem Autor über seine Nachbehandlung der Kinderlähmung 1955 im Wilhelminenspital mit Malariafiebertherapie; die Übertragung des Malariablutes sei seiner Erinnerung nach am *Steinhof* direkt von einem Malariafiebertherapiepatienten erfolgt.
[265] Im Archiv finden sich die nach 1950 abgelegten Akten von zwei Patienten, die bei einer Aufnahme vor 1951 (1939 bzw. 1948) mit dem Hinweis auf einen ‚luesfreien Malariastamm' eine Malariafiebertherapie bekamen. Beide hatten die Diagnose „Debilität". Die Vermerke auf den Fieberblättern am Tag der Impfung waren: „Lu[es] frei, zum Stamm" bzw. „Luesfr. M. Stammhalter", d.h. dass beide auch für eine Weitergabe des malariainfizierten Blutes vorgesehen waren.

Um 1950 waren die positiven, wenn auch nur kurzen Hinweise zur Anwendung der Malariafiebertherapie in der Psychiatrie außerhalb der Neurolues in den eingesehenen internationalen Handbüchern noch häufiger als dann in den 1960er Jahren. So schrieb der Schweizer Psychiater Max Müller, Leiter der Heilanstalt Münsingen bei Bern 1949 (2. Auflage), dass bei epileptischen Störungen durch Fieber „mit luesfreien Malariastämmen oder auch mit chemischen Fiebermitteln […] zweifellos vorübergehende Erfolge erzielt“ worden seien, „ohne aber ein dauerhaftes Sistieren der Anfälle bewirken zu können.“[266] Georges Heuyer[267], der als Begründer der Kinderpsychiatrie in Frankreich nach 1945 gilt, nannte 1952 die Fiebertherapie – wobei nicht deutlich wird, ob es sich um die Malariafiebertherapie handelt – als hilfreich in der Kinderpsychiatrie in der Behandlung von „retards de croissance“, bei Folgeschäden einer Enzephalitis und bei Schizophrenie.[268] Und das französische Handbuch zur Psychiatrie von Porot erwähnt in den drei gleichbleibenden Auflagen von 1952, 1960 und 1965 die Anwendung der Malariafiebertherapie abgesehen von der PP „mit interessanten Ergebnissen“ nicht nur bei der Tabes, sondern auch bei der Multiplen Sklerose, bei Entzündungen des Sehnervs und bei der Chorea nach Thomas Sydenham.[269]

In *The Lancet* finden sich 1952 noch zwei kurze Fallberichte von britischen Kinderärzten über Kinder im Alter von zweieinhalb und fünf Jahren, die eine Malariafiebertherapie bei einer Lipoidnephrose (heute besser bekannt unter Minimal-Change Glumerulonephritis) bekommen hatten.[270] In der Leipziger Zeitschrift *Psychiatrie, Neurologie und medizinische Psychologie* erwähnt 1965 ein Artikel Erfolge in der Behandlung der genuinen Epilepsie mit Malaria- und anderen Fiebertherapien.[271]

---

[266] MÜLLER, Prognose und Therapie der Geisteskrankheiten (1949), S. 161.
[267] Zum 10jährigen Jubiläum der Wiener Kinderstation schrieb er einen Beitrag: HEUYER, Evolution de la Pédiatrie et de la Neuropsychiatrie infantile (1962), S. 135-145. Ebenso der Schweizer Pionier der Kinderpsychiatrie Moritz TRAMER, Zur Frage der Somatopsychik (1962), S. 166-171.
[268] Georges HEUYER, Introduction à la psychiatrie infantile (Paris 1952), S. 293. Zur Anwendung verschiedener Mittel zur Fieberprovokation bei Kindern vgl. KUNDRATITZ, Die therapeutische Beeinflußbarkeit zerebralgestörter Kinder (1957), S. 425 („Pyrifer, Typhusvakzine, Vakzineurin oder […] Frauenmilchinjektionen“); KLARE, Rehabilitation und Poliomyelitis (1960), S. 179, zitiert oben S. 79 Anm. 264.
[269] POROT, Manuel (1952, ^{2}1960, ^{3}1965), S. 303 (Übersetzung in einfachen Guillemets von GH).
[270] BYRNE, Malarial Therapy in Lipoid Nephrosis (1952), S. 844-845; GAIRDNER, Nephrosis treated by Malaria (1952), S. 842-844.
[271] WAND, Die Fieberbehandlung bei der genuinen Epilepsie (1965), S. 17-19. A. Wand, Chefarzt der Nervenklinik des St. Joseph-Krankenhauses in Berlin-Weißensee (Berlin-Ost) zitiert einleitend Berichte über die Anwendung von Fiebertherapien bei Epilepsie 1928 bzw. in den 1930er Jahren. Über seine eigenen Erfahrungen schreibt er hier von ca. 30 der ca. 40 Epilepsie-PatientInnen pro Jahr, die in den letzten Jahren mit Fiebertherapie behandelt wurden.

Eduardo Balduzzi[272] schrieb in seinem Buch über die ‚Schocktherapien', in dem er 1962 diese verteidigte – vor allem die Insulinkoma-, die Elektrokrampftherapie sowie den Acetylcholin-Schock nach Mario Famberti –, im Kapitel „Metodi desueti e metodi minori" über „Gli shock febbrili", dass die Fiebertherapie – abgesehen in der Behandlung der progressiven Paralyse und anderer Endstadien der Lues („manifestazioni metaluetiche") – sich als sehr wenig erfolgreich vor allem bei der Behandlung endogener Psychosen, insbesondere der Schizophrenie erwiesen habe; nur bei psychomotorischer Erregung[273] (besonders in manischen Stadien) sei die Fiebertherapie noch angewandt worden, bis sich zeigte, dass EKT auch bei manischen Syndromen sehr gut und schnell wirken würde und schließlich die Neuroleptika entdeckt wurden. Die Fiebertherapie hätte heute nur noch Aufgaben von zweitrangiger Bedeutung.[274] Rückblickend – vor allem auf die 1950er Jahre – nannte Balduzzi Kombinationen von Fiebertherapien mit EKT und die ‚totale Methode' des spanischen Psychiaters A. De Sales Pessoa, der mit der Kombination von Cardiazol-Insulin-Fiebertherapien Erfolge gehabt habe.[275] Nach Balduzzi scheint die Malariafiebertherapie Anfang der 1960er Jahre in (Nord-)Italien als ‚Schocktherapie' außerhalb der Neurolues nicht mehr aktuell gewesen zu sein, wohl aber noch in den 1950er Jahren in marginaler Bedeutung.

In Frankreich war die Anwendung noch Anfang der 1960er Jahre aktuell – wohl vor allem durch ältere Psychiater wie im folgenden Fall: In einem kurzen Protokoll über seine Erinnerungen an ein Praktikum als Medizinstudent im dritten Jahr an der Pariser *Salpêtrière* Anfang der 1960er Jahre schrieb Professor François Lemaire (Internist),[276] dass er zwar keine Malariafiebertherapie selbst gesehen habe (wohl aber Elektrokrampf- und Insulinkomatherapien), dass der Leiter der Abteilung Prof. N. P. hingegen mehrmals betont habe, dass eine Malariakur als *ultima ratio*-Therapie[277] in Erwägung zu ziehen sei, wenn die anderen Therapien keine Besserung brächten. Das habe nicht Paralytiker betroffen, von denen

---

[272] Er leitete in diesen Jahren das Ospedale neuropsichiatrico provinciale di Varese (als Nachfolger von Mario Fiamberti).
[273] In Wien dürfte das keine Standardindikation gewesen sein. Hier wurde bei Diagnosen in Kombination mit ‚psychomotorischen Anfällen' nur in 2 von 12 Fällen eine Malariakur gegeben: So beim 1967 16jährigen Patienten X827 mit der Diagnose „Psychomot. Anfälle, Verhaltensstörung" und bei der 1959 15jährigen Patientin I903 mit der Diagnose „psychomotor. Anfälle, Debilität". SPIEL/SPIEL, Zur Therapie des Schwachsinns (1960), S. 449f. nennen Erfolge in Fällen „von Kombinationen des Schwachsinns mit motorischen neurologischen Erscheinungen" (zitiert unten S. 92f.).
[274] BALDUZZI, Le terapie di shock (1962), S. 174f.
[275] Ebd., S. 176f.
[276] Gedächtnisprotokoll von François Lemaire zu einem Gespräch, Beilage zu seiner e-mail an Gernot Heiss vom 16.5.2012. Übersetzung aus dem Französischen von GH in einfachen guillemets.
[277] Vgl. oben S. 31 Anm. 93 das Zitat aus BLEULER, Lehrbuch der Psychiatrie ([10]1960), S. 405.

er in diesem Praktikum keinen einzigen Fall gesehen habe. Der Stamm des Plasmodiums, so der Leiter der Abteilung, würde ‹ im bakteriologischen Labor des Spitals sorgfältig aufbewahrt und am Leben erhalten ›. François Lemaire berichtete schließlich von dem Fall eines extrem verwirrten Patienten, bei dem die Malariafiebertherapie für den nächsten Tag angeordnet worden sei; in der Nacht sei der Patient jedoch plötzlich ins Koma gefallen und sei an einem posttraumatischen, intra-zerebralen Hämatom operiert worden. Das hämische Schmunzeln, mit dem der Leiter der Abteilung am nächsten Morgen empfangen worden sei, habe der falschen Diagnose gegolten und nicht dem Umstand, dass die Therapie bereits überholt gewesen wäre.

Im Rückblick erwähnt 1974 G. Lupascu, Professor am Institut für Mikrobiologie, Parasitologie und Epidemiologie „Dr. I. Cantacuzino“ in Bukarest, mehrere Anwendungsbereiche der Malariafiebertherapie in der Psychiatrie, die auch in den PatientInnenakten der „Ära Hoff“ vorkommen: ‹ bei Schizophrenie, bei Oligophrenie mit Pfropf-Schizophrenie und großer Erregung, bei affektiven Psychosen, Chorea etc. › Die Entwicklung von ‹ beruhigenden Substanzen › habe, so Lupascu 1974, die Behandlung mit Malariafiebertherapie bei diesen Krankheiten jedoch ‹ mit voller Berechtigung › ersetzt.[278] Dieser Aufzählung fügt er noch den Hinweis bei, dass auch versucht worden sei, mit Malariafiebertherapie die Thrombangiitis obliterans, eine chronisch-entzündliche Gefäßerkrankung, zu behandeln und sogar Krebs; es seien jedoch Einzelfälle gewesen, die keine Schlüsse zuließen.

Es sind eine Vielfalt von Anwendungen außerhalb der Neurolues, die häufig im Rückblick, aber auch noch zu den 1950er und 1960er Jahren genannt werden. Im Folgenden geht es um die Praxis an der Wiener Klinik und ihre Motive bei den einzelnen Diagnosen, wozu nicht nur die Krankenakten, sondern auch die Hinweise zur Anwendung bei der spezifischen Diagnose in der Wiener und in der internationalen Literatur als Quelle dienen.

---

[278] LUPASCU, Applications actuelles de la malariathérapie (1974), S. 166. Übersetzungen aus dem Französischen mit einfachen Guillemets im Text von GH. Für die Erprobung von Malariamedikamenten sollte seiner Meinung nach dennoch die therapeutische Überimpfung bei nicht luetischen PatientInnen mit deren Konsens weiter aufrechterhalten werden: vgl. unten S. 169 Anm. 627 und 629.

2.1.4.2.1 Die Anwendung der Malariafiebertherapie bei schizophrenen Erkrankungen

Die Anwendung der Malariafiebertherapie bei schizophrenen Erkrankungen wurde in der zitierten Literatur mehrmals erwähnt, wenn auch immer nur sehr kurz und ohne die Indikation etwas ausführlicher zu begründen und die Wirkweise zu erklären. Angewandt wurde sie bei diesen Diagnosen bereits seit den 1920er Jahren. Forschungen über die Wirksamkeit scheinen gefehlt zu haben und so schrieb Max Müller noch 1949, dass die „Fieberbehandlung (mit luesfreiem Malariablut)“ und andere Therapien[279] „in allen refraktären Fällen“ „selbstverständlich […] immer wieder versucht werden müssen“, auch wenn dies „bisher noch weniger gesicherte Maßnahmen“ seien.[280] In seiner Zusammenfassung zur Anwendung somatischer Therapien in der Psychotherapie erwähnte H. Walther-Brüel in *Der Nervenarzt* 1954, „daß unspezifische Reize, z. B. Fieberkuren, bei endogenen Psychosen mitunter günstig wirken können, ist seit alters bekannt.“[281] 1963 schrieb der Wiener Kliniker Ottokar H. Arnold[282] in seinem Buch zur *Therapie der Schizophrenie*: „schon bald nach der Einführung der Malariafiebertherapie durch Wagner-Jauregg in der Behandlung der progressiven Paralyse wurden einzelne Versuche unternommen, den Effekt, auch bei anderen Erkrankungsgruppen, also z.B. Schizophrenie, zu prüfen. Im Allgemeinen können wir heute sagen, daß bei bestimmten Formen der prozeßhaften Verläufe die Fiebertherapien innerhalb des Gesamtbehandlungsplanes in Frage kommen.“ „Methodik der Wahl“ sei die „Kombination einer solchen Fieberkur mit anschließenden Majeptil[krampf]kuren[283] und dann der Versuch der Eingliederung in eine Arbeits- bzw. Gruppentherapie“ vor allem bei Jugendlichen mit Diagnosen, „die man früher unter dem Begriff der ‚Pfropfschizophrenie’ oder ‚Pfropfhebephrenie’ zusammengefaßt hat“[284], d.h. bei Fällen von Schizophrenie, „bei denen auf der Basis einer Reifungsstörung der Persönlichkeit – im besonderen sichtbar im Spektrum

[279] Er nennt „die antitoxische und antiinfektiöse Therapie, bei frühzeitigen hebephrenen Krankheitsbildern auch die Hormondarreichung nach Wagner-Jauregg“, weiters die Thyreoidinbehandlung bei Gjessingschen Fällen. Es würden „namentlich von französischen Autoren […] Kombinationen zwischen Fieber- und Krampfbehandlung, Dauerschlaf- und Krampfbehandlung empfohlen, ohne daß über die Resultate bereits etwas Abschließendes zu sagen wäre.“

[280] MÜLLER, Prognose und Therapie der Geisteskrankheiten (1949), S. 101.

[281] WALTHER-BÜEL, Zur klinischen Therapie der endogenen Psychosen (1954), S. 194.

[282] Er war nach seinen ersten Arbeiten über Schizophrenie und zum EKT-Block bei der akuten tödlichen Katatonie bereits von Kauders an die Klinik geholt worden, hatte sich hier 1954/55 habilitierte und war seit Ende der 1950er Jahre Erster Assistent und damit Vertreter des Klinikleiters: vgl. GABRIEL, Zum Wiederaufbau akademischer Lehrkörper in der Psychiatrie in Wien nach 1945 (2016), S. 66f.

[283] Zum Majeptil (Thioproperazin) s. unten im Abschnitt zu den Neuroleptika, S. 285-288. Vgl. ARNOLD/HOFF, Neuroleptika, Tranquilizer und Antidepressiva (1962), S. 42.

[284] Zur zeitgenössischen Diskussion der Frage, ob der in der Diagnosebezeichnung gestellte enge Zusammenhang von frühkindlichem Intelligenzmangel und Schizophrenie besteht, vgl. ATSCHKOVA, Die Pfropfschizophrenie im Kindes- und Jugendalter (1966), S. 292-295.

der intellektuellen Reifung – und bei gegebener spezifischer Disposition in der Pubertät oder beginnenden Reifezeit eine primäre schizophrene Prozeßpsychose ausgeklinkt wird“. [285] Dabei schildert er auch den Behandlungsverlauf der Fiebertherapie und die „selten[en]“ Komplikationen.[286]

> Ein Fall aus dem Publikationsjahr von Arnolds Buch, der diese Ausführungen – die Anwendung der Malaria- mit anschließender Majeptil[krampf]kur – bestätigt (auch die standardmäßige Arbeitstherapie) und mit einer nachfolgenden Neuroleptikakur ergänzt, war jener des 19jährigen Mittelschülers S655. Er kam mit der Diagnose „Schizophrenie (pri[märer][287] Prozess)“ und erhielt bei seinem ersten, 136tägigen Aufenthalt Anfang 1963 zuerst zur Sedierung Truxal, wurde 14 Tage nach der Aufnahme mit *Malaria tertiana* inokuliert,[288] hatte 5 Fieberschübe und ging nach den 7 Chinintagen fast täglich zur Arbeitstherapie. 23 Tage nach dem letzten Chinintag wurde ihm 3 Tage lang Majeptil gegeben (10, 20 bzw. 30 mg); am 2. und am 3. Tag bekam er Krämpfe im Nacken. Nach 8 Tagen wurde die Majeptilkur 4 Tage lang mit 10, 20, 30 bzw. 40 mg wiederholt; am 3. Tag „krampft[e]“ er wieder (der Krampf während der Majeptilkur war nach Arnold das „Ziel“ der Therapie [289]). 10 Tage später wurde eine 84tägige Therapie mit dem Neuroleptikum „Fr33“[290] begonnen; während dieser Therapie ging der Patient regelmäßig zur Arbeitstherapie.

In der ‚Ära Hoff‘ hatte bei frühen schizophrenen Erkrankungen die Insulinkomatherapie den Vorrang, die häufig mit Cardiazol- oder Elektrokrampf im Koma kombiniert wurde. Eine EKT wurde mehrmals vor der Insulinkur gegeben, manchmal auch eine Malariakur: Vor einer Komatherapie bekamen etwa 1954/55 der 14jährige Patient S319 mit der Diagnose Hebephrenie bei seinem 6monatigen Aufenthalt eine Malariafiebertherapie.[291] Ähnlich bekam

[285] Von einer Pfropfschizophrenie wurde gesprochen, wenn sich sozusagen auf eine Oligophrenie eine Schizophrenie „aufpfropft“, das heißt, dass zeitgleich eine Oligophrenie und eine Schizophrenie festzustellen ist. „Oligophrenie“ und synonym „Schwachsinn“ sind damals gebräuchliche Ausdrücke für Intelligenzmängel gewesen. Bei diesen Diagnosen fehlte die Angabe zum Intelligenzquotienten.

[286] ARNOLD, Die Therapie der Schizophrenie (1963), S. 59-61. Zur Anwendung der Malariafiebertherapie bei Pfropfschizophrenie vgl. auch unten S. 93 das Zitat aus SPIEL, Die Therapie in der Kinder- und Jugendpsychiatrie (1967), S. 140.

[287] Information von Eberhard Gabriel (ab 1966 an der ‚Klinik Hoff‘): „'primärer Prozess' hat im damaligen, wohl auf Arnold zurückgehenden Sprachgebrauch geheißen: primär = von Anfang an chronisch (unter Umständen fortschreitend), nicht in Episoden verlaufend. Das Wort Prozess hat sich dabei auf einen hypothetisch angenommenen somatischen Krankheitsprozess bezogen, zu dessen Aufklärung die (schließlich diesbezüglich ergebnislosen) Stoffwechseluntersuchungen (von Arnold und [Gustav] Hofmann) dienen sollten.“ Vgl. auch oben S. 25 Anm. 67.

[288] Lt. Eintrag am Deckblatt waren nicht nur der „Fieber-Revers“ eingeholt worden, sondern – ohne Anwendung – auch die Reverse für „E“ und „I“ „Schockbehandlung“. Wie in diesem Fall, so wurden mehrmals bei PatientInnen Reverse für mehrere dieser Therapien eingeholt, aber nicht alle ausgeführt.

[289] Vgl. das Zitat aus ARNOLD, Die Therapie der Schizophrenie (1963), S. 79-81 im Kapitel zu den Medikamenten S. 285f.

[290] Wie Haloperidol / Haldol und Luvatren ein Neuroleptikum aus der Stoffgruppe Butyrophenone, das im Versuchsstadium blieb: MEIER/ KÖNIG/TORNAY, Testfall Münsterlingen (2019), S. 146 (Versuche ab 1962 mit negativen Testergebnissen).

[291] Zu ihm unten S. 218f.

die 13jährige Patientin S902 bei ihren beiden ersten, nur durch eine Woche getrennten Aufenthalten 1953/54 mit der Diagnose „Schizophrene Reaktion bei antriebsgestörter postencephalit[ischer] Persönl[ichkeit]“[292] („Grund der Aufnahme: Mutismus, Depression, Geräusche-Hören, SM-Gefahr, Fluchttendenzen“) zuerst eine Malariafiebertherapie und nach einer EKT mit sechs Anwendungen eine Insulinkomatherapie mit 50 Komata.[293] Bei einer nächsten Aufnahme 1 ½ Jahre nach einer Insulinkomatherapie erhielt 1955 der nun 20jährige Patient S348 mit der Diagnose „Schizophrenie“ eine Malariatherapie.[294]

Für diese letztgenannte Reihenfolge – zuerst die Insulinkomatherapie und erst bei einem späteren Aufenthalt die Malariafiebertherapie – dürfte ausschlaggebend gewesen sein, dass die Insulinkomatherapie nur früh, während oder nach dem ersten schizophrenen Schub und üblich auch nur einmal gegeben wurde. So hatte der Patient S790 1946, als 17jähriger, bei seinem zweiten Aufenthalt mit der Diagnose „Incip. Schizo“ eine Insulinkomatherapie mit 37 Komata bekommen.[295] Vier Jahre später (1950), bei seinem sechsten Aufenthalt an der Klinik bekam der nun 20jährige Maturaschüler eine Malariafiebertherapie. Nachdem er erst 14 Tage zuvor vom *Steinhof* positiv entlassen worden war, war er nun mit „Parere“ eingewiesen worden – nach der Sicht des gerade noch minderjährigen Patienten grundlos auf Initiative des Vaters. Der Grund für die immer wiederkehrenden Auseinandersetzungen mit den sehr religiösen Eltern, vor allem der Mutter, dürfte bereits 1946 seine Homosexualität gewesen sein.[296]

Dieser Fall gibt einen kurzen Einblick in die Sicht des Patienten, der die Einweisung auf Initiative des Vaters ablehnte. Darauf wird in den Anamnesen selten eingegangen, obwohl die Zwangseinweisung bei psychisch Kranken aus ärztlicher Perspektive als Problem für den Genesungsprozess gesehen wurde.[297] Diese Einsicht war aus therapeutischer Sicht ein Grund dafür, dass viele der polizeilich mit „Parere“ eingewiesenen PatientInnen an der „Klinik Hoff“

---

[292]Zur Anwendung der Malariatherapie bei postencephalitischer Persönlichkeitsstörung vgl. unten S. 95 und S. 127.
[293] Zu ihr unten S. 220. Sie war vermutlich wegen ihrer Jugend, wie nach den überlieferten Akten alle MalariatherapiepatientInnen der Kinderstation, nicht als Stammträgerin vorgesehen und hatte (nur) 4 Fieberschübe.
[294] Zu ihm unten S. 220.
[295] Er bekam (nach der Zick-Zack Methode) innerhalb von 2 ½ Monaten sehr unterschiedliche Dosen Insulin: die Dosis wurde anfangs von 16 E[inheiten] auf 144E beim 5. Koma gesteigert; dann gesenkt auf 120E, 80E und 72E, die zum Koma führten, und dazwischen mehrere Tage auf 20-40E mit ‚Subschock‘ ohne Koma; daraufhin führten bereits Insulingaben von zwischen 48 und 20E zum Koma (14.-37. Koma).
[296] Hinweise in den Anamnesen des 7mal aufgenommenen Patienten.
[297] Vgl. SCHARFETTER, Die “freiwillige Aufnahme” in eine geschlossene Anstalt (1958), S. 661. Vgl. BLEULER, Lehrbuch der Psychiatrie ([10]1960), S. 160f. und BLEULER, Lehrbuch der Psychiatrie ([11]1969), S. 165.

dazu bewogen wurden, der Aufnahme als „freiwillig“ zuzustimmen.[298] Die Freiwilligkeit der Aufnahme sollte allerdings nicht nur die Einstellung der PatientInnen gegenüber der Behandlung positiv verändern, sondern für die Klinik entfiel dadurch auch die Notwendigkeit, für die (weitere) zwangsweise Anhaltung die richterliche Zustimmung einzuholen, die bereits seit 1916 nach den anhalterechtlichen Bestimmungen der Entmündigungsordnung erforderlich war. 1956 wurden die Regelungen im Krankenanstaltengesetz erweitert, wodurch „nun die Selbs- und/oder Fremdgefährdung zum entscheidenden Kriterium eines zwangsweisen Eingriffs [wurde]. Damit war eine Anhaltung zu rein therapeutisch-fürsorglichen Zwecken unrechtmäßig und konnte nur über den Weg des Entmündigungsrechts erfolgen.“[299] Die neuen Einschränkungen der ärztlichen Behandlungsentscheidungen führte zu heftiger Ablehnung durch leitende Psychiater. Hoff etwa empfahl seinen Kollegen Anstaltsleitern „eine sehr ‚geräumige‘ Interpretation der Selbstgefährdung“.[300] Die Einforderung von mehreren Reversen bei der Aufnahme, also vor jeder Therapieentscheidung, dürfte eine Maßnahme gewesen sein, um diese Einschränkungen zu umgehen.

Laut Datenbank wurden in Wien in den beiden Jahrzehnten folgende schizophrene Erkrankungen (bzw. ihre Symptome) mit Malariafiebertherapie behandelt:

[298] Vgl. SCHARFETTER, Die “freiwillige Aufnahme” in eine geschlossene Anstalt (1958), S. 662: Die Freiwilligkeitserklärung sei „nicht an den Augenblick der Aufnahme gebunden“, der / die Kranke solle „sogar erst zu einer Zeit die Erklärung vorgelegt bekommen, ‚wo er sich nicht mehr in der Umgebung jener Personen befinde, die ihn in die Anstalt gebracht haben‘ […]. Auch soll er […] zuerst sehen, wie er untergebracht ist, und Fragen stellen können. Selbstverständlich“ sei, dass „der Gesundende jederzeit die Anhaltung in eine Freiwilligkeit verwandeln“ könne. Die Änderung am Umschlagblatt der Akten von polizeilicher Einweisung auf „freiwillig“ – meistens mit Stempel – ist sehr häufig. Vgl. auch SCHARFETTER, Die Bewährung des österreichischen Anhaltungsverfahrens (1959), S. 21-29 (ausführlich Diskussion im Vergleich zu Schweizer und deutschen Regelungen).

[299] Zur öffentlichen Kritik an der Psychiatrie und an den psychiatrischen Anstalten Anfang der 1950er Jahre in Deutschland, angestoßen durch den Film *Die Schlangengrube* (*The Snake Pit*, US 1948), und (in deren Folge) zu den rechtlichen Änderungen in Deutschland: NOACK, Über Kaninchen und Giftschlangen: Psychiatrie und Öffentlichkeit in der frühen Bundesrepublik Deutschland (2006), S. 311-340.

[300] FORSTER, Staat, Politik und Psychiatrie in Österreich – am Beispiel der rechtlichen Regulierung von Zwangsmaßnahmen von 1916 bis 1990 (1999), S. 173f. Zur Geschichte der „rechtlichen Regulierung von Zwangsmaßnahmen“ in der Psychiatrie in Österreich im 20. Jahrhundert mit der Tendenz zur „Einschränkung ärztlichen Ermessens“, „vom Schutz des ‚Geistesgesunden‘ vor einer ungerechtfertigten Internierung [in der Entmündigungsverordnung 1916] hin zum Schutz des ‚Geisteskranken‘ vor unverhältnismäßigen Eingriffen in persönliche Rechte [im Unterbringungsgesetzt 1990]“ – mit Kritik des Autors an der geringen Wirksamkeit der rechtlichen Einschränkungen in der klinischen Praxis –, vgl. ebenda, S. 166-189.

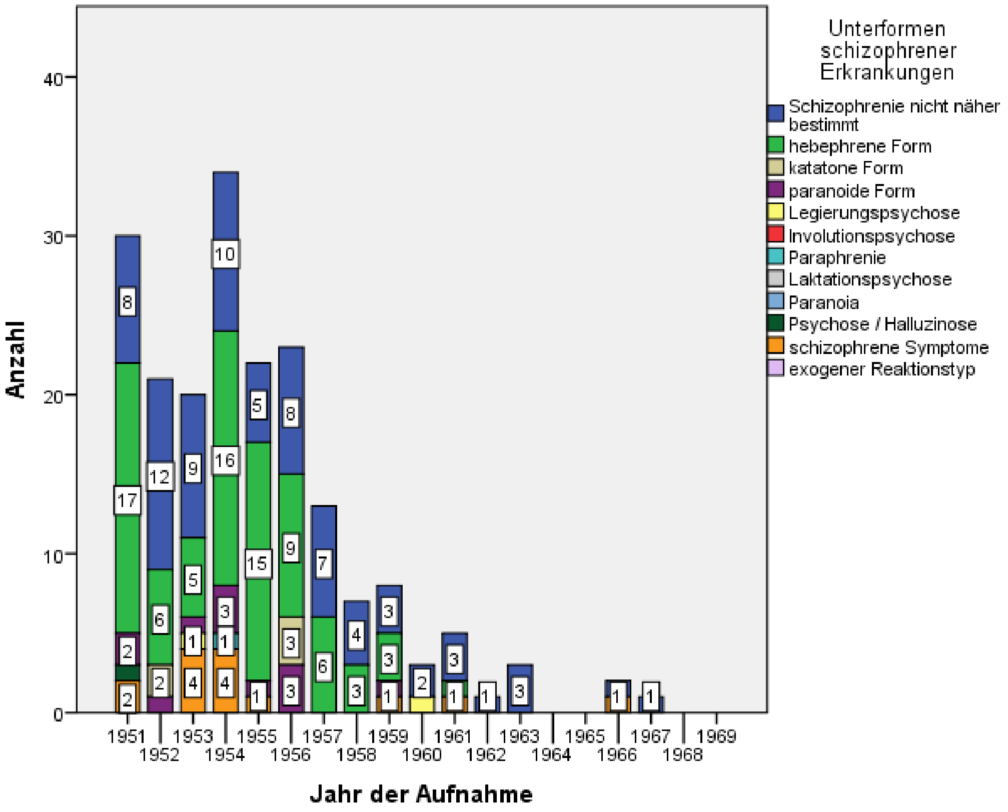

Abb. 3 Gestapeltes Balkendiagramm, Malariafiebertherapie + Unterformen der schizophrenen Erkrankungen (1951-1969), Pat. länger als 4 Tage stationär, n = 193.

PatientInnen mit einer schizophrenen Erkrankung machten einen großen Teil derjenigen aus, die in den 1950er Jahren in der Wiener Klinik mit einer Malariakur therapiert wurden (häufigste Diagnose von 1954-1956). In den 1960er Jahren hat die Malariafiebertherapie in der Behandlung der schizophrenen Erkrankungen hingegen fast keine Rolle mehr gespielt. In der Wiener Literatur der 1950er Jahre – wie oben zitiert – finden sich jedoch kaum Argumente für die Anwendung der Malariafiebertherapie bei Schizophrenie und keine für die Einschränkung dieser Indikation in den 1960er Jahren. PatientInnen mit einer hebephrenen Schizophrenie sind in den 1950er Jahren überproportional häufig mit einer Malariakur behandelt worden (Abb. 3, grüne Balken); diese Diagnose geht in den 1960er Jahren stark zurück[301] und ihre Therapie mit einer Malariakur verschwindet.

An der Wiener Klinik wurden bei einem Aufenthalt oder bei nachfolgenden Aufenthalten verschiedene der alten somatischen Therapien angewandt,[302] von denen die

[301] Die Diagnose Hebephrenie geht nach der Statistik völlig zurück – von 211 Fällen 1951-1959 auf 14 Fälle 1960-1969. Vgl. oben S. 83 das Zitat aus ARNOLD, Die Therapie der Schizophrenie (1963), S. 59-61, woraus man hier auf eine Änderung der Diagnosebezeichnung schließen könnte.
[302] Vgl. die Beispiele im Abschnitt über die Insulinkomatherapie unten S. 215-225.

Malariafiebertherapie – gleich wie die Insulinkomatherapie und anders als die Krampftherapien – bei PatientInnen ohne Neurolues nur einmal gegeben wurde.[303] Nach Arnold war, wie auch aus dem obigen Zitat von 1963 hervorgeht, die Fiebertherapie bei PatientInnen mit schizophrenen Erkrankungen nur eine Therapie kombiniert oder in Abfolge mit anderen Therapien – auch mit Neuroleptika. Zu letzterem schrieb Arnold: „Wenn eine Exazerbation bei Prozeßverläufen abzufangen ist, wird an die Fieberkur eine längerdauernde Neuroleptikabehandlung angeschlossen werden müssen."[304] Ein Beispiel, in dem die Neuroleptikatherapie einer unvollständigen Malariakur folgte, war der 17jährige kaufmännische Angestellte S867 aus dem Wiener Lehrlingsheim „im Werd". Er bekam bei seinem ersten Aufenthalt (von sechs, innerhalb von zwölf Monaten aufeinander folgenden Aufenthalten 1967/68) an der Klinik mit der Diagnose „Schizo-defekt, hypomanisches Bild" eine Malariafiebertherapie:

> Zuerst erhielt er fast täglich 10 – 30 mg Valium, bis er am zwölften Tag nach seiner Aufnahme mit malariainfiziertem Blut inokuliert wurde. Beim ersten Fieberschub (39,3°) bekam er Wahnvorstellungen, musste fixiert werden und erhielt an mehreren Tagen, aber nicht regelmäßig, Truxal. Der Patient wurde bereits beim zweiten Fieberschub „abgeimpft"[305] und kupiert. Noch während der Chinintage wurde eine 25tägige Therapie mit täglich 2x 200 mg Melleril „ret" (Neuroleptikum Thioridazinhydrochlorid)[306] begonnen. Bei den folgenden vier Aufenthalten blieb man mit Truxal und Melleril-retard bei der medikamentösen Behandlung.[307] Der Patient wurde jeweils nach dem 1. bis 5. Aufenthalt in das der Klinik angeschlossene Rehabilitationszentrum in Maria Lanzendorf entlassen. Beim letzten (6.) Aufenthalt mit der Diagnose „Schizophrenie Defekt, Exacerbation" wurden dem Patienten vor seiner Überstellung auf den *Steinhof* eine EKT mit fünf Anwendungen gegeben.

Bei der Diagnose einer schizophrenen Erkrankung wurde die Malariafiebertherapie – wie auch bei anderen Diagnosen – sehr unruhigen Patienten gegeben.

> Beim 1952 27jährigen Patienten S236 mit der Diagnose „Schizophrenie, stat. thymolymphaticus, [darunter nachgetragen] Kollaps im Erregungszustand" scheint die Fiebertherapie sogar eine Steigerung der Therapien gewesen zu sein, „da dann [nach einer EKT mit 6 Anwendungen] ein Rückfall mit Körpersensationen und Angst auftrat, wurde eine Malariabehandlung begonnen", heißt es in einem Protokoll der Klinik im Patientenakt. Laut Eintragungen zum ‚decursus therapiae' kam es „zwei Tage nach der Beendigung der [E-]Schockbehandlung […] zu einem Rückfall. Pat. ist unruhig, erregt,

---

[303] Anders – wenn auch selten – bekamen lt. Datenbank 11 PatientInnen mit einer Neurolues-Diagnose zweimal eine Malariafiebertherapie: Vgl. oben S. 72.
[304] ARNOLD, Die Therapie der Schizophrenie (1963), S. 60, und weiter: „Die gleichzeitige Gabe von Neuroleptika während der Fieberkur ist dagegen zu vermeiden."
[305] Seltener Eintrag auf der Fieberkurve.
[306] Melleril retard wurde häufig gegeben. Zur Indikation und erwarteten Wirkung vgl. die Studie der beiden Kliniker CZERWENKA-WENKSTETTEN/HOFMANN, Klinische Erfolge mit Melleril-retard (1966), S. 845-847, zitiert unten S. 283 Anm. 1061.
[307] Beim 4., 15tägigen Aufenthalt bekam der Patient ebenfalls in den ersten 5 Tagen Truxal und anschließend Melleril ret., beim 5., 18tägigen Aufenthalt durchgehend Melleril.

> in hypomanischer Stimmungslage […] wurde heute mit 4 ccm Malariablut geimpft." Der Patient war einer der drei PatientInnen, die während einer Malariafiebertherapie an der Klinik verstarben.[308]

Eine Steigerung der Therapien scheint die Malariafiebertherapie auch beim Patienten S636 mit der Diagnose „Schizophrenie" gewesen zu sein.

> Er bekam erst 1963, bei seinem dritten und letzten 19tägigen Aufenthalt als 21Jähriger eine Malariafiebertherapie. Für diese Therapie wurde er – so in der Anamnese – „*als Stammträger*" vom *Steinhof* an die Klinik transferiert; „[B]ei der Exploration ist Pat deutlich manisch". Beim ersten 160tägigen Aufenthalt 1958/59 hatte der 16jährige Tischlerlehrling zuerst eine EKT mit vier Anwendungen, dann eine Insulintherapie mit 50 Komata bekommen, dann Arbeitstherapie und – dazu parallel – eine EKT mit sechs Anwendungen; dann war er weiter bis zur Entlassung durch mehrere Wochen zur Arbeitstherapie gegangen. Beim zweiten, 56tägigen Aufenthalt 1960 bekam er vom 3. bis zum 13. Tag eine EKT mit sechs Anwendungen und ab dem 5. Tag bis zum 14. Tag tgl. 3x 1 Tbl. à 10 mg des Neuroleptikums 7843 (Studienname für Majeptil oder ein „verwandtes" Neuroleptikum[309]). Ab dem 15. Tag bis zur Entlassung ins Lehrlingsheim (Leopoldstadt) wurde ihm mehrmals jeden 2. Tag nur 1 Tbl. des Medikaments 7843 à 10 mg gegeben; aus dieser Medikation sind keine Krämpfe verzeichnet; auch ging er fast täglich zur Arbeitstherapie. Nach den Therapievorschlägen der Wiener Kliniker in ihren Publikationen dürfte er bei beiden längeren Aufenthalten in Gruppentherapie gewesen sein, die – wie auch bei anderen Fällen, in denen sie aber im *Decursus* oder im Entlassungsbrief genannt wurde – nicht auf der Fieberkurve eingetragen war.[310] Ob er 1963 mit der *Malaria tertiana* infiziert wurde, um seine akute manische Unruhe oder den schizophrenen Schub zu behandeln und dabei die Malariakur als weiteren Versuch nach dem Scheitern der anderen Therapien einzusetzen, oder nur als „Stammträger", um den Stamm der *Malaria tertiana* zu erhalten,[311] was die Formulierung in der Anamnese nahelegt, ist aus den Akten nicht zu klären.

Aufgrund des Mangels an Aussagen in den Wiener Krankenakten ist leider nicht eindeutig zu klären, welche Gründe für die Indikation ausschlaggebend waren: War es Unruhe bzw. Mutismus, waren es andere Symptome? Erhoffte man die Ansprechbarkeit verschlossener PatientInnen zu verbessern oder – wie bei Kindern mit Intelligenzmängeln nach Karl Kundratitz – erkrankte Neuronen auszuschalten und gesunde anzuregen?[312] Oder ging es darum, körpereigene Abwehrkräfte zu aktivieren? Und wurde aufgrund eines dieser

---

[308] Lt. Protokoll nach dem Tod: Diagnose „paranoide Schizophrenie"; nach 2. Fieberzacke „aus voller Ruhe einen Erregungszustand mit heftigen Halluzinationen […] hochgradig motorisch unruhig, erregt, aggressiv […]." Nach einer Beruhigung mit Medikamenten (1 Amp. Scopolamin), „aus der Ruhe plötzlicher Gefässkollaps mit Herzstillstand". Zu ihm und den beiden anderen Todesfällen vgl. oben S. 57 Anm. 177.

[309] Lt. Patientenakt des 1959 23jährige Patient S4634: „Sulfonamid-Phenothiacin-Therapie".

[310] Vgl. die Ausführungen zur Psychotherapie an der Klinik S. 104f.

[311] Siehe dazu unten S. 155-157 den Exkurs zum Begriff „Stammträger". Er gab die Malaria weiter; es gab parallel eine 2. Stammträger-Kette, was jedoch auch zur doppelten Sicherung des Stamms gedient haben könnte und deshalb nicht eindeutig gegen die durch die Bemerkung in der Anamnese angeregte Hypothese einer Anwendung nur (!) zur Erhaltung des Stamms spricht.

[312] Vgl. die Hypothesen von Karl Kundratitz unten S. 91.

Symptome oder Ziele hier statt einer medikamentösen, einer Koma- oder einer Krampftherapie die Fiebertherapie gewählt? Auch hier kann nicht ausgeschlossen werden, dass in einigen Fällen mit der Anwendung vorrangig[313] oder allein beabsichtigt war, den Plasmodien-Stamm zu erhalten.

Die Anwendung der Malariafiebertherapie bei nicht-luetischen Erkrankungen waren kürzer als bei der progressiven Paralyse. Sie wurden üblich nach vier bis maximal sechs (manchmal auch früher) und nicht wie bei der progressiven Paralyse nach acht Fieberschüben kupiert. Ein Beispiel für die kurze Fieberkur, wie auch für die sukzessive Anwendung mehrerer der ‚großen' körperlichen Kuren aus der Zwischenkriegszeit gibt die Krankengeschichte der folgenden Patientin:

> Die Patientin S895 mit der Diagnose „Schizophrenie" war laut Anamnese ein lebhaftes Kind, bis sie nach 1938 aufgrund der nationalsozialistischen Rassengesetze sehr isoliert gewesen sei und Schul- und Platzangst sowie 1944 schweres Bronchialasthma bekommen habe**.** 1949, als 18-Jährige, wurde sie zum ersten Mal an der Klinik mit der Diagnose „Hemmungszustand Schizo" aufgenommen. Sie fühlte sich von unbekannten Männern verfolgt, lag im Bett und war nicht ansprechbar. Sie erhielt eine EKT mit insgesamt 8 Anwendungen, nach den letzten drei – so der *Decursus* – sei sie „aus ihrem stuporösen Zustandsbild herausgekommen"; trotz der Besserung wurde schließlich noch „ein Cardiazolschock mit 0,4g 10% Lösung durchgeführt".[314] Auch beim 2. Aufenthalt im Frühjahr 1951, als sie 20 Jahre alt war, erhielt sie eine EKT, aber nach 15 Anwendungen, wovon die letzten 6 „E[-Schock] m[it] 4[-5]ccm Cardiazol [Embro i.v.]" (lt. Decursus: „6 Cardiazol-Elektroschocks"[315]) waren, „erwies sich auch diese Behandlung ohne weiteren Effekt". Bereits Ende 1951 wurde sie zum 3. und letzten Mal „kaum kontaktfähig" aufgenommen. Den Aussagen ihres Vaters zufolge nahm sie „keine Nahrung zu sich, spreche gar nichts und bewege sich nicht aus dem Bett heraus. Zeitweise gehe sie gegen die Eltern tätlich vor." Aufgrund dieses Bildes wurde die Malariafiebertherapie mit 2 Fieberschüben gegeben, für deren Kürze in der Krankengeschichte kein Grund erwähnt wurde. Am 2. Chinintag wurde die Patientin wohl aufgrund des üblichen Platzmangels an der Klinik zur Weiterbehandlung auf den *Steinhof* überstellt.

---

[313] Wofür sich etwa LUPASCU, Applications actuelles de la malariathérapie (1974), S. 166, noch 1974 ausspricht: vgl. das Zitat oben S. 82**.**

[314] In den ausgewerteten Akten ist das einer von 3 Fällen einer Cardiazolkrampftherapie ohne dem Insulinkoma ‚aufgesetzt' oder als ‚Cardiazol-Elektro-Schock' kombiniert zu sein – und dieser Fall liegt außerhalb des Untersuchungszeitraumes; sein sehr schmerzhafter Verlauf wird im Akt ausführlich beschrieben. Vgl. dazu Hugo SOLMS, Die Krampfbehandlung (1963), S. 468, der von „Todeserlebnisse[n], Angst- und Vernichtungsgefühle[n]" in der Latenzzeit schreibt). Die beiden Anwendungen im Untersuchungszeitraum (1951 und 1954) wurden gegeben, um die Patientinnen nach einem Suizidversuch (durch den Schmerz) aus dem Koma zu wecken (vgl. oben S. 39 Anm. 122).

[315] Es ist auch einer von drei Krankenakten, in dem „Cardiazol-Elektro-Schocks" ohne Insulinkoma erwähnt wurden; vgl. unten S. 215 Anm. 838.

2.1.4.2.2 Über die Anwendung der Malariafiebertherapie in der Behandlung „zerebralgestörter Kinder“

Über die Anwendung der Malariafiebertherapie in der Behandlung „zerebralgestörter Kinder“ referierte 1956 Karl Kundratitz, der von 1952 bis 1961 die Universitätskinderklinik in Wien leitete. Diese sei 1924 zunächst an kongenital-luetischen (mit Syphilis infiziert geborenen) und später an entwicklungsgeschädigten Kindern angewandt worden. Gestützt auf die Erfahrungen, die er an seiner Klinik gesammelt habe, sah Kundratitz in der Malariafiebertherapie neben der Hormontherapie, der Behandlung mit Glutaminsäure, der Röntgenbestrahlung des Zwischenhirns, heilgymnastischen und orthopädischen sowie medikamentösen Behandlungen eine aussichtsreiche Möglichkeit einer positiven Beeinflussung von prä- und postnatal geschädigten Kindern. In seinem Referat von 1956 gab er an, die Malariakur bei „bisher weit über 100“ schweren Fällen, bei Kindern, die über drei Jahre alt waren, durchgeführt zu haben.[316] Bereits in einer Publikation von 1946 hatte Kundratitz die Anwendung „der Fiebertherapie auch bei nichtluetischen Schwachsinnigen“, bei „bisher […] gegen 50 Kinder[n]“ erwähnt.[317]

Kundratitz ist in seinem Vortrag von 1956 (publiziert 1957) ausführlich und aufschlussreich hinsichtlich seiner Hypothesen zum Wirkmechanismus der Fieberbehandlung, für den sonst bei nicht-luetischen Erkrankungen keine Überlegungen in der eingesehenen Literatur der Zeit zu finden waren: „Der günstige Erfolg durch die Malariakuren ist […] einerseits im Sinne einer Umstimmung, Reiz- und Stresswirkung auf das Vegetativum und Endocrinium aufzufassen, andererseits wohl auch als eine direkte Einwirkung auf die pathologisch-anatomisch veränderten Gehirnregionen im Sinne einer Herdreaktion“; „derselbe Stress, der die kranken oder zugrunde gegangenen Zellen zur vollsten Ausschaltung bringt, regt andererseits andere Zellen zur Regeneration und Funktionssteigerung an.“[318] Nach Kundratitz bewirkte die Malariafiebertherapie eine Stoffwechselsteigerung im Gehirn, womit sie einen regenerativen Vorgang einleiten würde: „Die Eigenart der Impfmalaria stellt also eine organspezifisch gerichtete Komponente dar, die zellulär an dem Hauptort der Hirnerkrankung angreift; die Malaria wirkt in gewissem Sinne encephalotrop. So müssen wir annehmen, daß

[316] KUNDRATITZ, Die therapeutische Beeinflußbarkeit zerebralgestörter Kinder (1957), S. 424.
[317] GRÖGER, Röntgen- und Malariatherapie. Zur Therapie des kindlichen Schwachsinns (2019), S. 168 zitiert KUNDRATITZ, Die Bedeutung der Encephalographie bei Krampfkrankheiten und geistigen Defekten im Kindesalter und deren therapeutische Beeinflussbarkeit (1946), S. 36.
[318] KUNDRATITZ, Die therapeutische Beeinflußbarkeit zerebralgestörter Kinder (1957), S. 425.

es bei den Malariakuren bei zerebralgeschädigten Kindern zu einer Einwirkung auf die krankhaft veränderten Gehirnpartien kommt. [...] Das Malariafieber wirkt durch Ausschaltung erkrankter Neurone (Pötzls Mauserungs- oder Reinigungstheorie[319]): Erliegen der geschädigten Zellen, um bei der Riesenreserve an Ganglienzellen des Gehirns noch stummen, nicht unmittelbar eingeschalteten, aber gesunden Zellen ihre Funktion zu überantworten sowie durch Leistungssteigerung vorhandener noch inaktiver Neurone desselben oder des benachbarten bzw. korrespondierenden Bereiches."[320] Diesem Verständnis des therapeutischen Nutzens des Fiebers nach hatte die Malariakur den doppelten Nutzen einerseits pathologische Veränderungen im Gehirn zu beseitigen und andererseits gesunde, aber noch nicht oder nur wenig genutzte Bereiche zu aktivieren.

An der ‚Klinik Hoff' leitete Walter Spiel die 1948 von Kauders angeregte und vorerst als Ambulanz für Kinder- und Jugendpsychiatrie eröffnete und schließlich unter Hoff 1951 eingerichtete „Psychiatrisch-Neurologische Kinderstation".[321] Mit der Malariafiebertherapie wurden hier überwiegend Kinder mit Gehirnschädigungen aufgrund von Geburtstraumen, Encephalitis, postnatalem Icterus und Diagnosen aus dem Diagnosefeld der ‚Intelligenzmängel' behandelt. Mehr als die Hälfte der Kinder mit Malariafiebertherapie waren PrivatpatientInnen und die meisten davon kamen aus dem Ausland.[322]

Lona und Walter Spiel schrieben 1960 mit einem Verweis auf die Untersuchungen von Kundratitz und auf dessen „optimistisch[e]" Beurteilung „der Verbesserung der Intelligenzfunktion" bei „Schwachsinnszustände[n]"[323]: „Zweifelsohne ist durch die Einführung der Fiebertherapie ein beachtenswerter Schritt in der Therapie gemacht worden, besonders geeignet wahrscheinlich für Fälle von Kombinationen des Schwachsinns mit

---

319 Das ist bei Pötzl freilich nur ein Aspekt in der Erklärung der Wirkung der Insulinkomatherapie; vgl. zum Bezug auf Pölzls ‚Mauserungstheorie' unten S. 189f. die Zitate aus den Publikationen von ARNOLD, Zur Theorie der Insulinschocktherapie der Schizophrenie (1952), S. 976, und HIFT, Zur weiteren Entwicklung der Insulintherapie (1952), S. 976, sowie von ARNOLD, Die Therapie der Schizophrenie (1963), S. 23f.
320 KUNDRATITZ, Die therapeutische Beeinflußbarkeit zerebralgestörter Kinder (1957), S. 425.
321 Vgl. oben S. 25 Anm. 65.
322 Zu den Ländern vgl oben S. 45. Vgl. GEIGER, Kinderstation (2015), S. 249-264 (mit Fallbeispielen), bes. die Tabelle S. 253f. Bei den 35 Kindern, die lt. Datenbank zwischen 1953 bis 1965 mit Malariafieber behandelt wurden, wurde 4x morbus Erb (1950, 1954/55/56), 3x nach Kinderlähmung (1953/54, 1956), 2x fragliche bzw. juvenile PP (1955, 1960), 2x kindliche bzw. Hellersche Psychose (1964/65), 1x Listeriose mit epileptischen Anfällen (1954), 1x Autismus (1965) diagnostiziert; bei den anderen 22 Kindern waren es frühkindliche Cerebralschäden.
323 In den wissenschaftlichen Publikationen, die im Projekt gesichtet wurden, wurde nur in diesem Artikel die Arbeit von Kundratitz zitiert, aber auch hier dessen Theorie nicht weiterführend diskutiert.

motorischen neurologischen Erscheinungen."[324] Und noch 1967 schrieb Walter Spiel: „An der vom Verfasser geleiteten kinderpsychiatrischen Abteilung der Psychiatrisch-Neurologischen Universitätsklinik Wien besteht noch immer die Möglichkeit, die von Wagner-Jauregg eingeführte Malaria-Fieberbehandlung durchzuführen, da an dieser Klinik der Malariastamm gepflegt wird und jederzeit eine Überimpfung möglich ist." Sie sei, wie andere Fieberkuren, „niemals eine spezifische Therapie", würde „aber immer wieder durchgeführt, vorwiegend bei zerebralgeschädigten retardierten Individuen, und zwar unter der Vorstellung, daß im Rahmen der Fiebertherapie es zu einer allgemeinen Umstellung im Organismus kommt, daß die Fiebertherapie eine Art Stress darstellt und die retardierte Entwicklung und die damit zurückgebliebene psychophysische Reifung mit neuen Impulsen versieht." [325] „Zwischenfälle wurden eigentlich nie beobachtet, da die Malaria-Fieberkur sehr gut kontrollierbar" sei.

Zehn Jahre nach der Publikation von Kundratitz scheint man weniger optimistisch, aber am gleichen wissenschaftlichen Stand gewesen zu sein. 1967 erklärte Spiel, dass die Malariafiebertherapie trotz mangelnder wissenschaftlicher Fundierung aufgrund von klinischer Erfahrung so lange in Verwendung sei: „Auch bei dem Zustandsbild der Pfropfschizophrenie[326] wurde öfters Malaria-Fieberbehandlung angewendet. Natürlich sind wir uns klar darüber, dass die Wirkung der Fieberkur wissenschaftlich in ausreichender Weise nie genau überprüft wurde, es gibt aber doch Fälle, wo eben der ‚Eindruck' einer Besserung besteht"**.**[327] Worauf dieser Eindruck beruhte, nennt Spiel nicht; nach den Hinweisen zur Indikation sollten die PatientInnen intellektuell aktiver und ruhiger werden.[328]

Die Anwendung bei Kindern mit Down-Syndrom erwähnt Hans Asperger – strikt ablehnend – 1952.[329] Erwähnenswert ist seine Vermutung, diese Therapie sei versucht worden, da

---

[324] SPIEL/SPIEL, Zur Therapie des Schwachsinns (1960), S. 449f.
[325] SPIEL, Die Therapie in der Kinder- und Jugendpsychiatrie (1967), S. 140. Ähnlich der Heidelberger Kinder und Jugendpsychiater MÜLLER-KÜPPERS, Die Therapie im Kindes- und Jugendalter (1972), S. 998: Bei der „Anwendung von Fieberkuren, insbesondere mit speziellen Malariastämmen oder Vaccinen," handle es „sich um eine unspezifische Reiztherapie, die z. B. bei hirngeschädigten Kindern in der theoretischen Vorstellung durchgeführt wird, daß es durch die Fieberattacken zu einer Umstellung des Organismus und zu einer Reaktivierung retardierter Persönlichkeitsmerkmale kommt."
[326] Die Pfropfschizophrenie ist beiden Diagnosefeldern zugeordnet: den schizophrenen Erkrankungen und den Intelligenzmängeln.
[327] SPIEL, Die Therapie in der Kinder- und Jugendpsychiatrie (1967), S. 140.
[328] Vgl. die beiden Patienten I408 1950 mit 19 Jahren und der Diagnose „Zustand nach frühkindlicher Meningitis, Debilität, Athetose double" und 1960 den 14jährigen Patienten X619 mit der Diagnose „Erregungszustände, neurotische Verwahrlosung, Zust. nach Meningitis" unten S. 127.
[329] Die Anwendung ist nicht lokalisiert und nicht datiert; der Hinweis 1952 dürfte sich auf Fälle vor 1951 bezogen haben, wurde aber (als aktuelle Warnung?) auch in den späteren Auflagen wiederholt.

„Mongolismus“ häufig in gutbürgerlichen Familien auftrete und man hier – auf Drängen der Familie – alles versuchen wollte, um den Zustand des Kindes zu bessern.[330]

Bei Diagnosen aus dem Feld der ‚Intelligenzmängel‘ wurde auch bei Jugendlichen, die – üblich ab dem Alter von 14 Jahren – stationär an den Erwachsenenabteilungen aufgenommen wurden, und bei jungen Erwachsenen die Malariafiebertherapie angewandt. Von 919 mit Diagnosen aus diesem diagnostischen Feld (freilich auch häufig in Kombination mit anderen Diagnosen, die dann zu beiden Diagnosen gezählt wurden) in der Datenbank aufgenommenen Fällen aus den PatientInnenakten der Erwachsenenabteilungen wurde die Malariafiebertherapie in 255 Fällen angewandt (s. oben Tab. 1).

Die Auswertung der PatientInnenakten brachte zum Diagnosefeld der ‚Intelligenzmängel‘ folgende Ergebnisse:

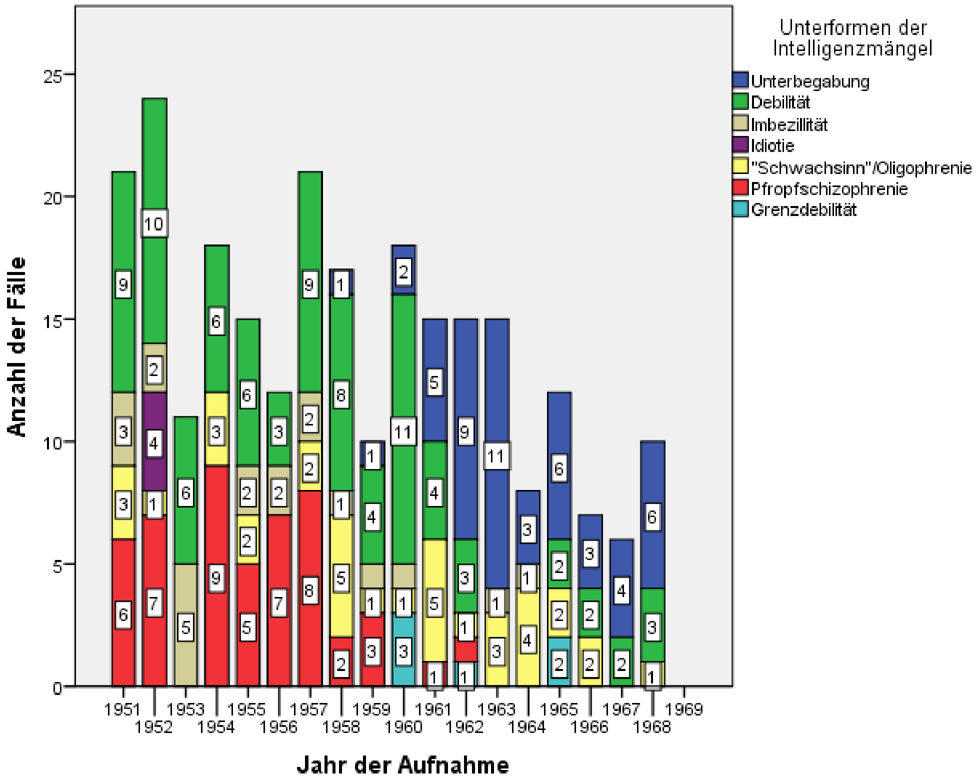

Abb. 4 Gestapeltes Balkendiagramm, Malariafiebertherapie + Unterformen der Intelligenzmängel (1951-1969), Pat. länger als 4 Tage stationär, n = 255.

[330] ASPERGER, Heilpädagogik ([1]1952), S. 100. Er war damals in Wien Dozent an der Kinderklinik.

Bei den Diagnosen aus dem diagnostischen Feld der ‚Intelligenzmängel' wird die Malariafiebertherapie über den gesamten Zeitraum angewandt; 1957 bis 1962 ist diese Diagnose die häufigste für eine Malariafiebertherapie. In den 1950er Jahren werden noch viele PatientInnen mit der Diagnose „Pfropfschizophrenie" (als Unterbegabung mit psychotischen Symptomen)[331] mit Malariafiebertherapie behandelt, nicht mehr in den 1960er Jahren. Die Diagnose „Unterbegabung"[332] tritt erst in den 1960er Jahren auf, sowohl bei den PatientInnen, die mit Malariafiebertherapie behandelt werden, als auch generell bei den an der Klinik aufgenommenen PatientInnen; diese Diagnose wurde in den 1950er Jahren entweder an der Klinik kaum vergeben oder kaum an der Klinik stationär behandelt.

Hinweise auf eine Anwendung zur „Verbesserung der Intelligenzfunktion" bzw. auf die Stimulation einer Nachreifung bei prä- oder postnatal geschädigten PatientInnen als Zielsetzung für die Indikation der Malariafiebertherapie bei Kindern, wie sie von Kundratitz und Spiel angesprochen und in der von ihm geleiteten Kinderabteilung vor allem bei PrivatpatientInnen durchgeführt wurde, finden sich auch manchmal in den PatientInnenakten junger PatientInnen der Erwachsenenabteilungen, vor allem bei PatientInnen mit den Diagnosen Psychopathie und aus dem Bereich der Intelligenzmängel: bei postnatalen Schädigungen (‚postencephalitischen Störungen') und Reiferückstände. So schrieb der behandelnde Kliniker 1965 in einem Brief an die Mutter des 14-jährigen Patienten I745 (Diagnose: „Psychopathie, Debilität"[333]): „Bevor wir die Durchuntersuchung abschliessen und er wiederum in die Heil- und Pflegeanstalt […] zurückkehrt, haben wir eine Fieberkur eingeleitet, welche dazu dienen soll, ihn ein bisschen nachreifen zu lassen."[334] Der 16jährige Patient X687, der bei seinem dritten und letzten Aufenthalt – alle 1964 – eine Malariafiebertherapie bekam, hatte immer die außerhalb der Einschlusskriterien stehende Diagnose „Postencephalitisches Zustandsbild, Kleinwuchs, Reifungsstörung, Erregungszustände";[335] Reifestörung und (wiederkehrende heftige) Erregungszustände

---

[331] Zur Diagnose vgl. oben S. 83f. Anm. 284 und 285.
[332] Die „Unterbegabung" wurde im Hoff-Skriptum, Allgemeine Psychiatrie [um 1961], S. 29 mit einem IQ von 85-95 definiert. Die Unterbegabung fällt in der ICD-8 in den Bereich der „Grenzfälle von Intelligenzmängeln", für die ein IQ von 68-85 angegeben wurde.
[333] Die „Debilität" fällt in der ICD-8 in den Bereich des „leichten Schwachsinns", für die ein IQ von 52-67 angegeben wurde.
[334] Möglich ist auch, dass die Therapie ‚Reiferückständen' im Syndrom der zweiten Diagnose „Psychopathie" gegolten habe. Vgl. zu ‚im EEG feststellbaren Reiferückständen' unten S. 122-126. Dieser Patient hatte ein: „Gering abnormes dysrhytmisches EEG mit diffuser Theta und flacher Delta-Einstreuung über den vorderen Schädelpartien."
[335] Er bekam während und nach der Fieberkur tgl. 3x 1Tbl. Tergetol (Carbamazepin; zählt chemisch zur Klasse der Dibenzazepine und ist ein Antikonvulsivum, das vorwiegend gegen fokale Epilepsien eingesetzt wird.

begegnen häufig in den Akten von PatientInnen mit Diagnosen außerhalb der Neurolues, die eine Malariafiebertherapie bekamen.

Was wurde bei ähnlichen Fällen gemacht, wenn an der Wiener Klinik keine Malariafiebertherapie gegeben wurde? Der Fall des 13jährige Privatpatienten[336] I9324 von 1969 mit der Diagnose „Debilitas, Geburtstrauma“, ist aus mehreren Gründen interessant: er fällt in das letzte Jahr der ‚Ära Hoff‘, in dem in Wien kein Malariastamm mehr zur Verfügung stand und eine Malariakur mit Malariablut aus dem Amsterdamer oder Hamburger Tropeninstitut wohl nicht mehr erwogen wurde; er war in einem Alter, in dem PrivatpatientInnen aus dem Ausland mit Diagnosen aus dem Feld ‚Intelligenzmängel‘ häufig an die Kinderstation zur(!) Malariafiebertherapie gesandt wurden; ein von Hans Hoff gezeichneter Befundbericht informiert über die Therapien und die Ergebnisse des 90tägigen Aufenthalts:

> „Der Patient wurde einem regelmässigen Schulunterricht zugeführt. Medikamentös erhielt er […] 3x 1 Dragee Encephabol [zur Verbesserung des Stoffwechsels im Gehirn, GH]. Weiters wurde Gymnastik und Massage durchgeführt. Der Patient wird nun in deutlich gebessertem Zustand entlassen. […] geistige Entwicklungszustand deutlich aufgeholt[337] […] etwas rascher im Tempo […] beobachtet gezielter […] Merkfähigkeit ist besser geworden. […]. Würde man den Patienten entsprechend weiter schulen, könnte noch eine deutliche Besserung der Leistungen erzielt werden […]. Wir empfehlen daher, die angegebene medikamentöse Therapie sowie die physikalische Behandlung weiter[zu]führen“. Eine „weitere Rehabilitation“ an der Klinik nach zwei Monaten wurde empfohlen, aber nicht wahrgenommen.

Am Anfang der ‚Ära Hoff‘ dürfte die vom rumänischen Tropenmediziner Lupascu im Rückblick genannte Indikation der Malariafiebertherapie zur Beruhigung von wiederkehrenden Erregungszuständen noch häufiger gewesen sein als nach der Entwicklung der neuen Psychopharmaka. Aber auch in der Zeit der Neuroleptika, neuen Tranquilizer und Antidepressiva dürften ‚Beruhigung‘ und ‚Umstimmung‘ noch Gründe für die Indikation gewesen sein[338] – mit einer Arbeitstherapie im Anschluss an die Fieberkur, gedacht als Vorbereitung der Eingliederung der PatientInnen in ihr soziales Umfeld.

> So bei der 17jährigen Patientin I899, die bei ihrem 34tägigen einzigen Aufenthalt 1959 mit der Diagnose „Pseudologia phantastica; Debilität“ eine Malariafiebertherapie

---

Darüber hinaus wird es auch als Phasenprophylaktikum bei verschiedenen psychiatrischen Erkrankungen eingesetzt. Strukturchemisch ist es dem Antidepressivum Imipramin ähnlich).

336 Diese lange und aufwändige stationäre Behandlung des Privatpatienten war freilich nicht Standard allgemein.

337 Es wird hier eine Zunahme des IQ im Test von der Aufnahme zur Entlassung von 50 auf 72 genannt.

338 So wurden Wutanfälle bzw. Aggressivität und / oder innere Spannungen, aber auch passive Ängstlichkeit, ‚Mutismus‘ und Verzweiflung bei FiebertherapiepatientInnen mit unterschiedlichen Diagnosen im gesamten Untersuchungszeitraum häufig genannt.

bekam.[339] Im Arztbrief bei der Entlassung hieß es: „Die Einweisung der Pat. erfolgte durch den Amtsarzt, da die Pat. sehr unruhig und erregt geworden war, und eine Reihe von phantastischen Erzählungen produziert hatte, welche der Wahrheit entbehrten. Derselbe Zustand war dann in den ersten Tagen ihres Aufenthaltes in der Klinik weiterhin gegeben, die Behandlung erfolgte anfangs mit Largactil, später wurde die Pat. einer Malariabehandlung mit insges. 6 Fieberstössen unterzogen […]. Pat. war unter dieser Behandlung wieder ruhig geworden, konnte dann der Arbeitstherapie zugezogen und […] wieder nach Hause entlassen werden. […] auch hysteriforme Mechanismen nachweisbar. Eine weitere Behandlung ist im Augenblick nicht nötig, doch ersuchen wir, uns die Pat. fallweise zur Kontrolle zuzuweisen."

Der 27jährige Patient I751 war 1965 nach einer Behandlung an der Linzer Psychiatrie mit dem Neuroleptikum Nozinan und mit „E-Schock[s]" „wegen seiner Verhaltensschwierigkeiten zur Beobachtung" an die Klinik überwiesen worden; seit einem Monat habe er „unter Konzentrationsstörungen, Zerfahrenheit, reizbarer Verstimmung […] und paranoide[n] Ideen" gelitten. Zu seiner Behandlung an der Klinik mit der Diagnose „Debilität, Überforderung" hieß es nach seinem 55tägigen Aufenthalt im Arztbrief: „Diagnostisch handelt es sich um eine Überforderungsreaktion bei einem debilen Pat. Für das Vorliegen einer Psychose, war während des klinischen Aufenthaltes kein Anhaltspunkt gegeben.[340] Therapeutisch machten wir bei dem Pat. nach einer anfänglichen versuchsweisen Einstellung auf 3x 10 NPL 82 (ein neues Neuroleptikum) eine Umstimmungstherapie mit einer Fieberkur. Bei der Entlassung war der Pat. beschwerdefrei und afebril. Es wird reichlicher Erholungsurlaub empfohlen."

Die Anwendung der Malariafiebertherapie als „Umstimmungs"- bzw. „Fieberumstimmungstherapie", um den Patienten bei ‚Überforderung', depressiver Stimmung und / oder nach einem Selbstmordversuch zu beruhigen,[341] dürfte neben der Zielsetzung einer ‚Nachreifung' ein Hauptmotiv für die Malariafiebertherapie bei PatientInnen mit ‚Intelligenzmängeln' gewesen sein.

So hatte sich der 19jährige Tischlerlehrling I202, der Anfang 1951 mit dem Diagnoseeintrag „Debilität, Suicidversuch" aufgenommen wurde, vor der Schande gefürchtet, da er in der Berufsschule vor dem Durchfallen stand. Zur Malariafiebertherapie, die bereits vier Tag nach der Aufnahme begonnen worden war, hieß es in *Decursus*-Einträgen: Der Patient sei nach zwei Fieberanfällen[342] mit Erbrechen „ziemlich heruntergekommen und muss coupiert werden. Psychisch fühlt er sich aber wohl, er werde jetzt seine Nervosität verlieren. Er wünscht schon im Fieber

---

[339] Ihre Diagnose „Pseudologia phantastica" wurde von BLEULER, Lehrbuch der Psychiatrie ([11]1969), S. 543, unter den „Psychopathieformen" genannt; die Therapie könnte also auch dem symptomatischen Prozess der psychopathischen Störung gegolten haben.

[340] Der Linzer Psychiater hatte kurz vor der Überweisung nach Wien in einem Schreiben an die oberösterreichische Gebietskrankenkasse geschrieben, der Patient leide an Pfropfhebephrenie und bedürfe einer „Insulin-Schockbehandlung"; in Wien hatte man nach einer Neuroleptikatherapie von der Diagnose und von einer Insulinkur Abstand genommen, da „kein Anhaltspunkt" für eine Psychose gegeben war, und – erst einen Monat nach der Aufnahme – mit einer Malariafiebertherapie zur „Umstimmung" begonnen.

[341] Der Begriff ‚Fieberumstimmungstherapie' findet sich manchmal auch in Akten von Patienten mit anderen Diagnosen nach einem Selbstmordversuch, so mehrmals bei depressiven PatientInnen; vgl. unten S. 103 zu drei Patienten 1968.

[342] Mit 40,5 bzw. 40,1°; sein malariainfiziertes Blut wurde weitergegeben.

mit der Fürsorgerin zu sprechen, damit man etwas für seine Arbeit tun kann. Es wird ihm bedeutet, dass dies noch ein paar Tage Zeit hätte.“ Sechs Tage später hieß es: „Pat. hat sich nach Koupieren rasch erholt. Er arbeitet auf der Station mit. Psychisch ist er völlig frei, keine Spur einer depressiven Verstimmung mehr, keine Suicidgedanken. Er will aufs Land gehen, nicht mehr die Schule besuchen. Die Eltern sind mit der Lösung einverstanden.“

PatientInnen mit unterschiedlichen Diagnosen kamen nach Suizidversuchen oder Suizidandrohungen mit einer Zwangseinweisung an die Klinik und wurden dort in den beiden Abteilungen für akute Fälle an den Erwachsenenstationen (B12 und A14) versorgt. Nach drei Tagen wurden sie meistens bereits auf den *Steinhof* überwiesen. Mehrere der mehr als vier Tage aufgenommenen PatientInnen bekamen eine Malariafiebertherapie. Bei PatientInnen mit Diagnosen aus dem Feld der ‚Intelligenzmängel‘ wurden Suizidversuche als „*Überforderungsreaktion*“ beschrieben, in mehreren Fällen auch als „*Kurzschlussreaktion*“ bzw. „*Kurzschlusshandlung*“; als solche wurden Suizidversuche als charakteristisch nicht nur für Patienten mit ‚Intelligenzmängeln‘,[343] sondern auch mit ‚Psychopathie‘[344] bezeichnet.

Der 20jährige Rekrut I556 mit dem Vermerk in der Diagnosezeile „SMV, Kurzschlussreaktion bei Unterbegabung“ erhielt bei seinem einzigen, 30tägigen Aufenthalt 1961 eine Malariafiebertherapie. Er gab finanzielle und familiäre Probleme aufgrund seiner Einberufung an – befürchtete, „dass man ihn [in seinem Beruf] nicht mehr arbeiten werde lassen, da er durch seinen Dienst beim Bundesheer zu lange in der Arbeit ausgesetzt hätte.“ Im Entlassungsbrief – zum Bundesheer – hieß es: es handle „sich bei dem Pat. um einen Unterbegabten, mit zahlreichen neurotischen Mechanismen, der in einer Kurzschlussreaktion diesen SMV begangen hat. Wir behandelten den Pat. mit einer Malariafieberkur, worauf sich sein psychischer Zustand bedeutend besserte.“

Rekruten nannten häufig Probleme mit dem Bundesheer als Grund für ihren Suizidversuch. Trotzdem wurden sie üblicherweise „zur Truppe“ entlassen.

Anders im Fall des Patienten I/P 847, der mit der Diagnose „SMV im Rausch, Unterbegabung, Psychopathie“ 1968 als 21jähriger eine Malariakur bekam. Im Patientenakt findet sich keine Klage über das Bundesheer, vielmehr hatte er sich selbst

---

343 Zum 17jährigen Patienten I2212 mit der Diagnose “Debilität, SMV” (ohne Malariafiebertherapie) hieß es 1954: „Es handelt sich bei dem M[inder]j[ährigen] um einen schwer debilen Psychopathen. Die Selbstmorde sind als Kurzschlusshandlungen, wie sie bei Debilen oft aufzutreten pflegen, aufzufassen“; auch in Zukunft sei mit Selbstmordversuchen zu rechnen. Der Patient wurde nicht zurück in die Erziehungsanstalt Kaiserebersdorf gebracht, sondern in die Landes Heil- und Pflegeanstalt Gugging „abgegeben“.

344 Zu „Kurzschlusshandlungen“ als charakteristisch für „Psychopathen“: HOFF, Lehrbuch der Psychiatrie (1956), S. 691 (Vorlesung: Psychopathie I): Diese Angst (Hoff schreibt von „raptusartige[n] Ausbrüche[n] einer primitiven Angst“ – anders als die „neurotische Angst“, die Aggressionen hemmt) führe „dann häufig zu Kurzschlußhandlungen.“ Hoffs Ausführungen, dass die psychopathische Persönlichkeit ihre „mächtigen“ Aggressionen vorwiegend nach außen richtet, widersprechen die häufigen Suizidversuche als ‚Kurzschlusshandlungen‘ in dieser Patientengruppe. HOFF/SLUGA, Das psychopathische Syndrom (1962), S. 253 erläutern jedoch zu den Suizidversuchen in dieser PatientInnengruppe: sie entstünden „nicht aus einer Depression heraus, sondern aus einem Haßgefühl gegen die ganze Welt“.

verpflichtet,[345] aber im Entlassungsbrief der Klinik hieß es: „Da Patient zu Kurzschlusshandlungen neigt und leicht überfordert und beeinflussbar ist, ist unseres Erachtens nach, eine Fortsetzung des Einsatzdienstes nicht zu vertreten."[346]

Im Übergang zu den anderen nicht-luetischen Patientengruppen ist der 19jährige Patient I/A853 zu nennen, der eine ‚Mischdiagnose' mit „Unterbegabung, depressives Bild, Alk. Mißbrauch**"** hatte und bei dem die Fürsorge zur ‚freiwilligen Aufnahme' mit Depression und der Gefahr eines Suizids argumentierte.[347] Er kam 1968 aus einem Kinder- und Lehrlingsheim in Niederösterreich, wo er eine Tischlerlehre absolvierte. Der Patient hatte eine ‚Heimkarriere' hinter sich. In den Heimen war er isoliert, wurde von den Mitzöglingen geschlagen und ausgenützt. 1966 lief er deshalb einmal davon. Danach bot die „Persönlichkeitsuntersuchung (Exploration AT)" „das Bild einer passiven, stimmungslabilen, willensschwachen Persönlichkeit, der es an Ichident[ität] ebenso gebricht (nicht sehr gescheit), wie an realisierbaren Zielvorstellungen". 1967 wurde zwar eine Besserung beobachtet, dennoch kam er nun (1968) zur Beobachtung an die Klinik und bekam bei seinem 51tägigen Aufenthalt eine Malariafiebertherapie. Zur Entlassung berichtete der „klinische Befundbericht": Die Aufnahme sei „wegen eines depressiven Zustandsbildes mit symptomatischem Alkoholmissbrauch bei Unterbegabung und psychopathischer Entwicklung" erfolgt.[348] „Wir unterzogen den Jugendlichen einer Fieberkur, ausserdem konnte er in die Arbeitstherapie eingegliedert werden[349]. Der Jugendliche verhielt sich an der Klinik angepasst und unauffällig, er ist jedoch nach wie vor leicht beeinflussbar, psychisch etwas labil, etwas ängstlich und infolge seines Intelligenzmangels leicht überfordert. Daraus resultierende Kurzschlusshandlungen sind möglich. Als wesentlichen Stabilisierungsfaktor würden wir eine Fortsetzung und Beendigung der Berufsausbildung ansehen. […] Eine weitere medikamentöse Therapie ist derzeit nicht angezeigt.[350] Eine intensive Bindung an einen Erzieher wesentlich." Nach Beendigung der Therapie wurde der Patient wieder ins Lehrlingsheim entlassen.

Die in den Krankenakten dieser PatientInnen-Gruppe angesprochenen Erwartungen, die Therapie würde eine ‚Nachreifung' anregen, nach einem Suizidversuch in erregter oder depressiver und stuporöser Stimmungslage ‚umstimmen' – beruhigen oder aktivieren –

---

[345] Es war sein 2. Aufenthalt an der Klinik: 1963 wurde er als 16jähriger mit der Diagnose „Psychopathie, Debilität" aufgenommen und nach 6 Tagen nach *Steinhof* überwiesen.
[346] Vgl. unten S. 145 Anm. 541 zur Empfehlung, den zeitverpflichteten Soldaten X849 aus dem Dienst zu entlassen.
[347] Der beiliegende Auszug aus den Eintragungen ins Personalblatt der Fürsorge berichtet von einem Gutachten und vom Grund für die Aufnahme: „Augenblicklich hätten endogene und exogene Ursachen (letzter wären in der Angst vor der Zukunft, im Zusammenhang mit Lehrende und bevorstehender Entlassung zu erblicken) zum Zustandekommen eines ausgesprochenen depressiven Zustandsbildes beizutragen[!]. Wie schon früher ist der Jugendliche auf Grund der bestehenden Persönlichkeitsinsuffizienz nicht im Stand sich ausreichend zu stabilisieren oder zu kompensieren. Augenblicklich ist die Gefahr, daß er sich mit Alkohol zu stimulieren sucht größer als die Gefahr eines Suicidversuches, doch geht die Entwicklung zweifelsohne in kritische Richtung. Wir empfehlen eheste Klinikvorstellung und medikamentöse Stimulierung."
[348] Die Kombination der Diagnose „psychopathische Entwicklung" bei Unterbegabung wird aus den sonstigen Angaben im Patientenakt nicht ersichtlich.
[349] Lt. Fieberkurve ging er auch zur Gymnastik.
[350] Auf der Fieberkurve sind – abgesehen von den während der Malariatheapie üblichen „1A Akrinor+10mg Percorten i. m.", den während der Fieberanfälle üblichen „20gtt Effortil" und den „2x0,5 Chinin+1x025 Resochin" zur Beendigung der Malaria – keine Medikamente eingetragen.

werden auch in den folgenden Ausführungen zu anderen Diagnosen genannt. Diese gleichen Erwartungen geben Hinweise auf Motive in der Indikationsstellung. Sie scheinen der Grund für die Anwendung der Malariafiebertherapie bei verschiedenen nicht-luetischen Erkrankungen gewesen zu sein. Warum jedoch der Mehrheit der PatientInnen mit gleichen Symptomen keine Malariakur gegeben wurde, erschließt sich daraus nicht.

2.1.4.2.3 Die Malariafiebertherapie bei affektiven Störungen

In den oben einführend zur Anwendung der Malariafiebertherapie bei Diagnosen außerhalb der Neurolues erwähnten Hinweisen aus der eingesehenen Literatur wurden auch affektive Störungen[351] genannt.

Nach der Auswertung der PatientInnenakten der Wiener Klinik zwischen 1951 und 1969 wurden 107 von insgesamt 8.037 Aufnahmen von PatientInnen mit Diagnosen aus dem Diagnosefeld der manisch-depressiven Erkrankungen mit der Malariafiebertherapie behandelt:[352]

---

[351] Etwa LUPASCU, Applications actuelles de la malariathérapie (1974), S. 166. In der Zwischenkriegszeit wurde von Erfolgen und über die Einschätzung der Wirkweise berichtet: Herrmann (Prag), Rezension (1930), S. 58, der Studie von M. L. BIANCHINI (Teramo), Malariatherapia delle psicosi maniacodepressiva. Vgl. auch WAGNER-JAUREGG, Über spezifische und unspezifische Behandlung von Geisteskrankheiten (1931), S. 291f.

[352] Von den 8036 Aufnahmen von PatientInnen mit diesen Diagnosen bekamen 4756 keine der ‚großen' alten Kuren, 3155 eine Elektrokrampftherapie, 84 eine Insulinkomatherapie, 2 eine Cardiazolkrampftherapie ohne die häufige Anwendung im Insulinkoma oder manchmal im E-Schock.

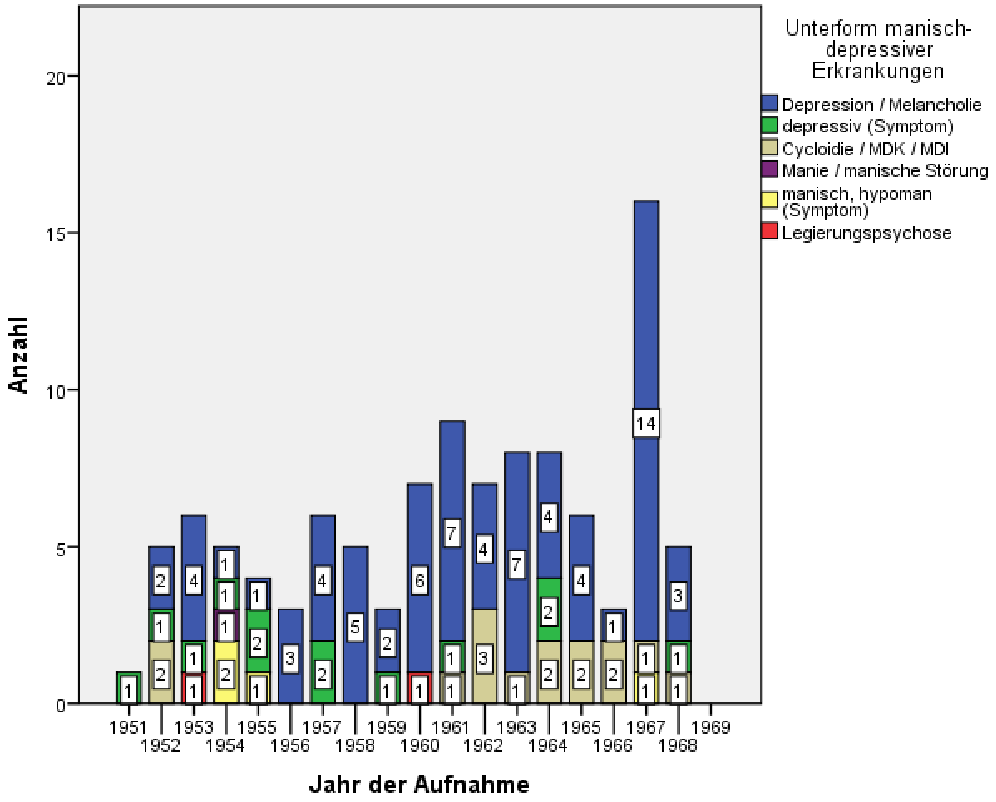

Abb. 5 Gestapeltes Balkendiagramm, Malariafiebertherapie und Unterformen der manisch-depressiven Erkrankungen (1951-1969), Pat. länger als 4 Tage stationär, n = 107.

Der Großteil der 107 PatientInnen, die eine Malariakur mit einer manisch-depressiven Erkrankung erhalten haben, hatte diese als Zweitdiagnose. Dies ändert sich in den 1960er Jahren: Nun hatten plötzlich über 30 Malariakur-Patienten (keine Patientin) als einzigen Hinweis in der Diagnosezeile einen Suizidversuch („SMV“) und eine psychogene Depression vermerkt (meist eine als ‚reaktiv‘, seltener als ‚exogen‘ bezeichnete Depression); die Fälle finden sich vor allem in den Jahren zwischen 1962 und 1967. Diese Veränderung in der Indikation ist auch einer der Gründe, warum die affektiven Störungen 1967 die häufigste Diagnose unter den Patienten mit einer Malariakur war (in diesem Jahr gab es keine Patientin mit einer Malariafiebertherapie). In der eingesehenen Wiener Literatur der Zeit wurde keine Erklärung für eine Malariafiebertherapie bei affektiven Störungen gefunden und so auch nicht für diese plötzliche Relevanz in den 1960er Jahren.

Die Diagnose Depression ohne zweite Diagnose erhielt der folgende Patient mit dem bei dieser Diagnose häufigen Zusatz „SMV“:

> Der 1964 20jährige Schlosser (z. Zt. Rekrut mit Aussicht auf eine Ausbildnerstelle) A674 mit der Diagnose „SMV, reaktive Depression“ erhielt bei diesem einzigen, 39tägigen Aufenthalt eine Malariakur. Laut Vater hatte er immer gut gelernt, es hätte

nie Erziehungsschwierigkeiten gegeben und er sei gerne beim Militär. Laut Anamnese hatte er in einem Kaffeehaus aus „Renom[m]iersucht“[353] einen Revolver hergezeigt; daraufhin habe ihn die von der Kellnerin gerufene Polizei angezeigt. Aus Angst vor Bestrafung habe er zu Hause das Gas aufgedreht; er schäme sich fürchterlich vor seinen Eltern und habe Angst vor seinem Vater. Im Entlassungsbrief an den Leiter des Heeresfachambulatoriums wird die Malariafiebertherapie wie üblich ohne einen Hinweis auf die Zielsetzung erwähnt. Angst vor der Justiz und vor dem Vater sowie Scham vor den Eltern wurden als bedeutsam für die reaktive Depression und den Suizidversuch angegeben – und einzig eine Malariafiebertherapie angewandt.[354]

In diesen Jahren war die Behandlung manisch-depressiver Erkrankungen mit Neuroleptika und Antidepressiva auch in der Wiener Klinik bereits durchaus üblich – beim folgenden Patienten erst bei der zweiten Aufnahme innerhalb eines Jahres gefolgt von einer Malariakur:

> Der Patient A761 wurde 1964 als 15jähriger bei seiner ersten, 29tägigen Aufnahme mit der Diagnose „Endogene Depression, Substupor“ mit Antidepressiva (3x 75 mg Truxal i. m. bzw. 3x 1Drg SD 709[355]) behandelt und ging zur Arbeitstherapie. 5 Wochen nach seiner Entlassung wurde er mit der Rettung wieder an die Klinik gebracht: Er war vom Meister beschimpft worden und wollte nun nicht mehr zur Arbeit gehen; „nachdem die Mutter [den] Pat[ienten] nötigen wollte aufs Jugendamt zu gehen, drohte er mit Suicid und richtete sich eine Eisenstange, falls man ihn gewaltsam wegführen wollte.“ Bei diesem zweiten, 61tägigen stationären Aufenthalt wurde er mit der Diagnose „Depressiver Hemmungszustand, Cycloidie“ in den ersten Tagen mit 2x 50 mg Truxal Drg, ab dem 5. Tag mit 2x 2 Amp. SD 709 i. m., ab dem 11. Tag mit 3x 2Drg SD 709 behandelt.[356] Er ging auch wieder zur Arbeitstherapie. Am 29. Tag seines Aufenthalts wurden die Medikamente abgesetzt und er wurde mit malariainfiziertem Blut inokuliert. Erst am 43. Tag bekam er den ersten Fieberanfall, nach 6 Fieberzacken und den 7 Chinintagen wurde er entlassen. Laut Arztbrief war die depressive Verstimmung durch die Antidepressiva abgeklungen, erst dann wurde die Malariafieberkur durchgeführt. Ein Grund, warum nach der positiven Wirkung des Antidepressivums die Malariafiebertherapie angewandt wurde, wurde auch hier nicht genannt. Die EEG-Befunde könnten zur Indikation beigetragen haben.[357]

---

[353] Vgl. „Geltungssucht“, die von BLEULER, Lehrbuch der Psychiatrie ([11]1969), S. 543, als Symptom der Psychopathie zugeordnet ist.

[354] Am Tag vor der Inokulation ergab der Befund ein „normales EEG mit regelrechtem Alpha-Rhythmus.“

[355] Vgl. dazu die Studie von Guth/Hoffmann, Erfahrungen bei der Anwendung von SD 709 beim depressiven Syndrom (1966), S. 14 und S. 16: SD 709 „saures Tatrat“ Handelsname Istonil; „neuartiges Antidepressivum mit vorwiegend antriebssteigernder Wirkung, das sich durch das Zusammentreffen von besonders raschem Wirkungseintritt mit sehr hohen Remissionsquoten und sowohl parental wie auch oral guter Verträglichkeit mit minimalen Nebenerscheinungen auszeichnet.“

[356] Am Abend bekam er – zur Sedierung – 2 Tbl. Miltaun (Meprobamat)+2 Tbl. Doriden (Glutethimid).

[357] EEG-Befund eine Woche nach 2. Entlassung (Nov. 1964) mit Bezug auf EEGs am Ende des 1. Aufenthalts (Juli 64): „Zusammenfassung: Gering abnormes EEG mit rechts Theta gestörtem Alpha-Rhythmus, der in Hyperventilation neben links temporalen niedrigen scharfen Wellen und niedrigen Theta Gruppen aktiviert wird. – Die abnormen Zeichen haben gegenüber den Vorbefunden weitgehend abgenommen.“ Am Ende der ersten Aufnahme hieß es: „Zusammenfassung: Abnormes EEG mit rechts parieto-occcipital umschrieben gestreuten Theta- und Delta-Wellenzügen, sowie steilen Wellen und davon abhängigen links frontal-zentralen Theta-Delta-Gruppen mit Spitzen und steilen Wellen. – N.B. Das Maximum der starken EEG-Veränderungen liegt über dem rechten [occi]pitalen Quadranten.“ Zur Frage des Einflusses von ‚leicht abnormen‘ EEG-Befunden auf die Indikation vgl. unten S. 122-126.

Manchmal finden sich in den Akten von Patienten mit affektiven Störungen kurze Hinweise auf die Zielsetzung der Fiebertherapie. So hieß es bei drei Rekruten, die 1968 nach einem Suizidversuch im Rahmen einer affektiven Störung aufgenommen worden waren, im Arztbrief bei ihrer Entlassung zur Truppe, sie hätten eine „Fieberumstimmungstherapie“ bekommen.

> Beim 21jährigen Buchdruckergesellen (z. Zt. Rekrut) A846 habe es sich „diagnostisch […] um einen Suicidversuch bei einem depressiven Zustandsbild im Rahmen einer Cycloidie“ gehandelt. „Der Pat. wurde bei uns einer Fieberumstimmungs-Therapie bestehend aus 6 Fieberstössen unterzogen, ausserdem wurde er arbeitstherapeutisch und psychotherapeutisch behandelt. Unter unserer Therapie kam es zu einem weitgehenden Abklingen der Symptomatik.“
>
> Beim 21jährige Rekruten A841 habe es sich „diagnostisch […] um einen Suicidversuch im Rahmen eines reaktiv depressiven Zustandsbildes“ gehandelt. „Der Pat. wurde bei uns [1967/68] einer Fieberumstimmungs- und Psychotherapie unterzogen, ausserdem wurde er zusätzlich medikamentös behandelt.[358] Unter dieser Therapie kam es zu einem Abklingen der depressiven Symptomatik.“
>
> Beim 20jährigen Präsenzdiener X/A866 mit der Diagnose „SMV im Rausch“ hieß es 1968: „Diagnostisch handelte es sich um einen Suicidversuch […] im Rahmen einer psychogenen Depression. Neben Psychotherapie und Arbeitstherapie wurde eine Fieberumstimmungstherapie durchgeführt. Bei der Entlassung erschien die Stimmungslage stabilisiert, die exogene Konfliktsituation (Bundesheer) gelöst“[359].

In den drei stereotyp formulierten Entlassungsschreiben aus 1968 (sie waren nicht vom gleichen Arzt unterzeichnet) sind zu den Fiebertherapien als Umstimmungstherapien zwei weitere Hinweise bemerkenswert – die positiven Hinweise auf die Arbeitstherapie, die als Vorbereitung für eine soziale Eingliederung galt und den Erfolg der Therapie kennzeichnete, und die Hinweise auf eine psychotherapeutische Behandlung: Arbeitstherapie ist fast immer auch in der Fieberkurve eingetragen, Psychotherapie nur in sehr wenigen Fällen in den 1950er Jahren; Hinweise auf Psychotherapie sind in den Akten überhaupt selten, manchmal wurde sie in den *Decursus*-Einträgen erwähnt. In den Jahren 1967/68 scheint sich das geändert zu haben.

> So hieß es 1967/68 zum 29jährige Fliesenleger A/I837, der mit der Diagnose „SMV (Leuchtgas), reaktive Depression, Unterbegabung“ aufgenommen worden war im Arztbrief: Der Patient wurde „einer Fieberkur unterzogen und psychotherapeutisch betreut. Eine echte Psychotherapie ist in Folge der Unterbegabung des Pat. sicher nicht erfolgversprechend.“[360]

---

[358] In der Fieberkurve sind jedoch keine Psychopharmaka verzeichnet.
[359] Letzteres, da er den Suizid am Ende seines Präsenzdienstes bei der Abrüstungsfeier versucht hatte.
[360] Auf der Fieberkurve ist nur Arbeitstherapie eingetragen. Im Entlassungsbrief wird auf den EEG-Befund eingegangen, der sich nach der Malariakur verbessert habe.

> Auch der um die Jahreswende 1966/67 aufgenommene 14jährige Patient P795 mit dem Vermerk in der Diagnosezeile „Psychopath[isches] Syndrom (Reifungsstörung im EEG) […]“ bekam nicht nur eine Malariafiebertherapie, sondern – wie im Befundbericht an das Bezirksjugendamt vermerkt wurde – auch „psychotherapeutische Betreuung“.[361]

Aus den Erwähnungen in den Krankenakten wird jedoch nicht ersichtlich, in welchem Umfang und mit welchen Methoden in den Erwachsenenabteilungen Psychotherapie angewandt wurde bzw. wo sie im Anschluss an den Klinikaufenthalt stattfand. Dazu dieser und der folgende Absatz: Psychotherapie wird in Publikationen der Wiener Kliniker häufig und bei verschiedenen psychischen Erkrankungen genannt; so verwies Hoff 1956 in seinem Lehrbuch auf die Notwendigkeit von Psychotherapie – vor allem der Gruppentherapie[362] – in der Behandlung der Schizophrenie. In den 1950er Jahren kann auch aufgrund des ärztlichen Personals eine psychotherapeutische Betreuung der PatientInnen angenommen werden, obwohl sie in den Krankengeschichten so spärlich erwähnt wurde. Einen Einschnitt in der psychotherapeutischen Behandlung an den Erwachsenenstationen dürfte um 1960 jedoch der Weggang von Wilhelm Solms (1959), Raoul Schindler (1960) und Heimo Gastager (1962) bedeutet haben, die an den beiden Erwachsenenstationen arbeiteten und dort jene Kliniker waren, die sich mit speziellen psychotherapeutischen Methoden beschäftigten.[363] Hoff veranlasst 1961 (bis 1965) die Einrichtung eines Psychotherapeutischen Lehrinstituts unter der Leitung von Hans Strotzka und unter Mitarbeit von Alois M. Becker, Wilhelm Solms, Knut Baumgärtel und Walter Spiel, von denen „die Definition von Psychotherapie als Voraussetzung für Lehre, Forschung und Behandlung erarbeitet wurde“.[364] Welche Auswirkungen dieses Lehrinstitut für die Praxis an der Klinik in den 1960er Jahren hatte, konnte im Rahmen des Projekts nicht ermittelt werden. Zeitzeugen, die an den beiden Erwachsenenstationen ab Mitte der 1960er Jahre arbeiteten, können sich jedoch an keine spezielle Form der Psychotherapie – auch an keine Gruppentherapie – in den Erwachsenenstationen erinnern.[365] In der zweiten Hälfte der 1960er Jahre dürfte es sich in den

---

[361] Ausführlicher zu ihm unten S. 123f.
[362] Vgl. z.B. HOFF, Lehrbuch der Psychiatrie (1956), S. 494 (Vorlesung: Therapie der Schizophrenie), zu den Erfolgen der Gruppentherapie geleitet von Schindler und Hift im Anschluss an die Insulinkomatherapie.
[363] Zu ihnen und zu drei weiteren Dozenten, die an der Klinik auch mit speziellen psychotherapeutischen Methoden arbeiteten – zu Erwin Ringel (Leiter der Psychosomatischen Station mit einem Schwerpunkt in der Individualpsychologie), zu Walter Spiel (Leiter der Kinderabteilung und ebenfalls Individualpsychologe) und zu Hans Strotzka (Leiter der von ihm auf Vermittlung von Hoff gegründeten psychotherapeutischen Ambulanz der Wiener Gebietskrankenkasse etc.) vgl. GABRIEL, Zum Wiederaufbau akademischer Lehrkörper in der Psychiatrie in Wien nach 1945 (2016), S. 67-70.
[364] Springer-Kremser, Die Neukonstituierung der Psychotherapeutischen Schulen (2016), S. 198.
[365] Interview Gernot Heiss mit Bernd Küfferle am 06.05.2013 und mit Eberhard Gabriel am 13.05.2013.

Erwachsenenstationen nur um führende Gespräche[366] gehandelt haben – anders an der Kinderstation der Klinik, wo Psychotherapie zum Standard gehörte.[367]

Gruppentherapie ist jedoch im Rehabilitationszentrum der Psychiatrie in Maria Lanzendorf (in Niederösterreich südöstlich, nahe bei Wien) anzunehmen, wohin in den 1960er Jahren die mit Schizophrenie diagnostizierten PatientInnen der Erwachsenenstationen, an denen PatientInnen ab 14, in Einzelfällen auch mit 13 Jahren behandelt wurden, zur Nachbehandlung überstellt wurden;[368] ebenso für Alkoholkranke im *Genesungsheim Kalksburg* und für die Jugendlichen aus den Erziehungsanstalten Kaiserebersdorf und Wiener Neudorf nach ihrer Rückkehr im Heim.[369]

Ob die Empfehlung im Entlassungsbrief des folgenden Patienten, ihn „bei neuerlich auftretenden Schwierigkeiten […] einem Psychotherapeuten oder unserer Klinikambulanz zuzuweisen“, als Hinweis auf eine ambulante psychotherapeutische Betreuung 1965 zu werten ist, war nicht zu klären. Sein Fall ist jedoch für die Beratung der Kliniker für die Änderung der Lebensführung nach der Entlassung aufschlussreich:

> Der Patient A742 wurde als 15jähriger 1965 mit der Diagnose „SMV, psychogene Depression“ aufgenommen wurde. Fünf Jahre zuvor (1960) war er an der Kinderambulanz begutachtet worden und damals hatte es geheißen: der Patient „leidet sehr unter dem Spott seiner Mitschüler. […] Im wissensmaessigen Bereich hoch trainiert. Gedaechtnis – neurotisch herabgesetzt. Persönlichkeitsmaessig handelt es sich um ein neurot. eingeengtes Kind, das sehr unter erzieh[erischem] Stress steht. – Zahlreiche Bremsfaktoren hat Pt. aufgebaut und versucht seine Affektivitaet zu hemmen. Psychotherapie angezeigt.“ Die psychischen Reaktionen des „neurotisch eingeengten“ 10jährigen Kindes aufgrund von „erzieherischem Stress“ sind auch noch 5 Jahre später dem Entlassungsbrief von 1965 zu entnehmen: „Anlaß zu diesem Suicidversuch war, daß der Patient nicht länger als Lehrling bei einer Bank tätig sein wollte, sich aber wegen eines Berufswechsels mit seinen Eltern nicht zu besprechen wagte. Diagnostisch handelt es sich bei dem Patienten um eine Überforderung auf Grund einer neurotischen Vorentwicklung. Sicherlich besteht bei dem Patienten eine

---

[366] Vgl. BLEULER, Lehrbuch der Psychiatrie ([10]1960), S. 498 zu „Psychopathien“: **„eine psychotherapeutische Führung und beständige Beratung über die Gestaltung des Alltags“.** Vgl. das ausführlichere Zitat unten S. **110** Anm. 388.

[367] Psychotherapie hatte an der Kinderstation der Klinik (bei Kindern unter 14 Jahren) auch in den 60er Jahren durch ihren Leiter Walter Spiel und seine Mitarbeiterinnen einen hohen Stellenwert. Vgl. SPIEL/HIFT/KOS/SCHISCHITZA, Die Psychotherapie im Kindes- und Jugendalter (1960), passim. Zu Fallbeispielen aus den überlieferten Krankenakten der Station, vgl. GEIGER, Kinderstation (2015), S. 245-249. Vgl. auch HOFF, War die Errichtung einer kinderpsychiatrisch-neurologischen Abteilung nötig? (1962), S. 103: Für ihn sei „die Möglichkeit […] verschiedenste [psychotherapeutische] Therapiemethoden anwenden und die Reaktion objektiv studieren zu könne“ einer der Hauptgründe für die Einrichtung der Kinderstation gewesen.

[368] Vgl. unten S. 191.

[369] Vgl. das Zitat dazu von Ringel, Solms und Spiel unten S. 115f.

> Reifungsdissoziation derart, daß die psychische Entwicklung hinter der körperlichen weitaus zurückbleibt. So ist der Patient psychisch infantil, unsicher und ängstlich. Wir haben therapeutisch die Situation mit den Eltern besprochen und veranlaßt, daß der Patient seinen Berufswunsch durchsetzen kann. Ansonsten wurde eine Fieberkur durchgeführt […]. Wir empfehlen bei neuerlich auftretenden Schwierigkeiten, den Patienten einem Psychotherapeuten oder unserer Klinikambulanz zuzuweisen.“ Wie meistens, wurde auch hier nicht angegeben, was von der Malariafiebertherapie erwartet wurde und in welchem Bezug die Malariafiebertherapie zur Diagnose stand; Umstimmung und Beruhigung gegen die Unsicherheit und Ängstlichkeit, vielleicht auch ‚Nachreifung‘ des psychisch infantilen Patienten sind hier mögliche Ansatzpunkte für die Indikation.

Selbstgefährdende Erregungs- und Angstzustände bei psychogener, meist reaktiver Depression wurden offenbar noch in den 1960er Jahren mit Malariafiebertherapie behandelt. Wie bei den anderen Diagnosen außerhalb der progressiven Paralyse bekam die (große) Mehrheit der PatientInnen mit affektiven Störungen jedoch keine Malariafiebertherapie.[370] Sie wurden mit EKT und / oder medikamentös behandelt und auch die Malariafiebertherapie-PatientInnen bekamen häufig die neuen Psychopharmaka.

> So der 28jährige Patient A836 (Cykloidie, sympt. Alc. Mißbrauch), der bei seiner 1. Aufnahme 1966 eine Malariafiebertherapie bekam. Im Entlassungsbrief hieß es, er leide „an Angst, Appetitlosigkeit, Einschlafstörungen, Lustlosigkeit und Antriebsschwäche, sowie Kopfschmerzen. […]. Diagnostisch handelt es sich um einen symptomatischen Alkoholmissbrauch bei hypochondrisch gefärbter Zykloidie.[371] Therapeutisch haben wir […] zunächst eine Fieberkur durchgeführt und anschließend eine medikamentöse Therapie mit 2x 5 mg Valium und 20 mg Distraneurin (Clomethiazol)[372] abends eingeleitet.[373] Unter dieser Therapie ist das Zustandsbild des Patienten leicht gebessert worden. Wir empfehlen jedoch weiterhin diese Medikation fortzuführen.“
>
> Eine Malariafiebertherapie sollte Anfang 1968 bei seinem einzigen 19tägigen Aufenthalt der 23jährigen Hilfsarbeiter A8648 mit der Diagnose „Cykloidie, depressive Phase“ bekommen. Laut Anamnese war er wegen „Todesangst“[374] freiwillig zur Aufnahme gekommen**.** Der Malariafiebertherapierevers war zwar eingeholt worden,

---

[370] Siehe oben Tab. 1 auf S. **34** und S. 100 Anm. 352.
[371] Bei anderen Aufnahmen wurde „Unterbegabung“ – lt. Hoff-Skriptum, Allgemeine Psychiatrie [um 1961], S. 29 mit einem IQ von 85-95 definiert – genannt.
[372] Vgl. die Studie an der Psychiatrischen Spitalsabteilung der Landesnervenklinik Salzburg mit 261 Patienten von GASTAGER/HAAS/WEINKAMER, Erfahrungsbericht über die Anwendung von Distraneurin in der Psychiatrie (1964), passim: Indikation bei Delirium tremens, „chron. Alkoholismus – zusätzlich zur üblichen Standardbehandlung zur Beseitigung von Abstinenzerscheinungen“, Medikamentensucht, Schlafstörungen, akute Psychosen, Unruhezustände bei chronischen epileptischen sowie bei organischen Psychosyndromen.
[373] Lt. Fieberkurve wurde ihm am 1. und 2. Tag 1x bzw. 2x 3 Kps. Distraneurin und vom 3. bis 7. Tag (dem Tag der Inokulation) tgl. Truxal (2x 80 mg+ am Abend meistens noch einmal 8 mg. Truxal p.o.) gegeben; er hatte fünf Fieberschübe; vom Tag vor dem 1. Fieberschub am 18. Tag tgl. bis zur Entlassung am 39. Tag 2x 5 mg Valium Tbl. (meistens am Abend noch einmal 5 mg Valium Tbl., in den letzten Tagen abends durch 1x 2 Kps. Distraneurin ersetzt).
[374] Seit er während eines Spitalaufenthalts seiner Frau 4 Monate allein zuhause war, begannen, Herzklopfen, Herzstechen, Atembeschwerden, Schluckbeschwerden, Todesangst mit Schweißausbrüchen.

> wegen einer Schilddrüsenüberfunktion war die Fieberkur jedoch kontraindiziert.[375] Welche Symptome zur ursprünglichen Indikation der Malariatherapie geführt hat, wird aus dem Akt nicht ersichtlich. Er bekam laut Fieberkurve durchgehend Valium[376] (tgl. 3x 10 mg, dann 3x 5 mg) und Tryptizol (Amitriptylin, Antidepressivum; anfangs 50+50+100 mg, dann 2x 50 mg).

„SMV“ in der Diagnosezeile sind bei PatientInnen mit einer Diagnose aus dem Spektrum der manisch-depressiven Erkrankungen sehr häufig, ist aber auch bei den Diagnosen aus dem Bereich der „Intelligenzmängel“, der schizophrenen Erkrankungen und des ‚psychopathischen Syndroms‘ nicht selten. Meistens waren entsprechende Diagnosen bei PatientInnen, die eine Malariakur bekamen, mit anderen Diagnosen und Symptomhinweisen in der Diagnosezeile kombiniert. So ist nicht deutlich zu erkennen, welcher Diagnose bzw. welchem Syndrom die Malariakur galt.[377]

Zu klären, warum die Fiebertherapie angewandt wurde, wird vor allem durch die Seltenheit und die durchwegs mangelnde Ausführlichkeit der Hinweise auf die Ziele und die Vorstellungen zur Wirkweise in den Wiener Krankenakten und Publikationen erschwert bzw. verunmöglicht. Bei den vielen PatientInnen mit mehreren Diagnose- oder Symptomhinweisen und mit der Anwendung von mehreren körperlichen Therapien erschweren bzw. verunmöglichen diese Fehlstellen die hypothetische Bestimmung des Ortes der Fieberkur im „Gesamtbehandlungsplan“.

> So hieß es 1964 in der Anamnese zum 20jährigen gelernten Zimmermann und nun zeitverpflichteten Gefreiten A700 mit der Diagnose „SMV, Hy Mechanismen im Rausch, Cycloidie“, der bei seinem einzigen Aufenthalt am Abend sedierende Medikamente[378] bekam, bis er am 15. Tag mit malariainfiziertem Blut inokuliert wurde: „Pat. gibt an, dass er schon als Bub nach geringen Differenzen enorm verärgert war, mit depressiver Verstimmung, und immer nachher einfach auf und davon lief, und zwar auf mehrere Stunden. Schulbesuch mit gutem Erfolg, Gesellenprüfung […]. Bei der Arbeit und nach geringen Differenzen mit dem Chef oder seinen Kollegen war er leicht verärgert. Anschließend geht Pat. trinken und läuft immer weg, meistens kann er sich dann an nichts mehr erinnern.“ Er reiht sich ein in die Malariakur-Patienten mit verschiedenen Diagnosen, die mit Stimmungsschwankungen als (immer wieder) leicht erregbar, verärgert und aggressiv und mit Fluchttendenzen („Pseudoporiomanie“) beschrieben wurden – mit Symptomen, die zur Indikation geführt oder beigetragen

---

[375] Lt. Antwort des Internisten auf Anfrage zur Freigabe des Patienten war die Malariakur „bei Vorliegen einer Hyper Thyreose […] K.I.“ „PST kontra“.

[376] Valium wird besonders als Medikament bei Angstzuständen geschätzt.

[377] So bekam 1959 die 16jährige Patientin A/I900 mit der Diagnose „Psychog[ene] Depression; SMV; intellektuelle Unterbegabung“ eine Malariafiebertherapie. Aus dem Krankenakt ist nicht ersichtlich, ob sie zur ‚Beruhigung‘ bzw. ‚Umstimmung‘ oder etwa zur ‚Antriebssteigerung‘ oder ‚Nachreifung‘ gegeben wurde oder eine andere Zielsetzung hatte.

[378] In den ersten Tagen am Abend 100 mg Truxal (3x), dann 2Tbl. Miltaun+2 Tbl. Doriden (10x).

haben könnten. Auch in seinem Fall sagte der Arztbrief an den Truppenarzt nichts über die Zielsetzung der Malariafiebertherapie aus.[379]

In den 1960er Jahren wird bei Patienten mit affektiven Störungen, mit häufigen Stimmungsschwankungen (Wutausbrüchen und Fluchttendenzen) und oft nach Suizidversuchen, mehrmals von der Notwendigkeit einer Umstimmung gesprochen und eine Malariafiebertherapie gegeben. Das therapeutische Ziel der „Umstimmung" durch die Malariakur scheint in diesen Jahren zu ihrer häufigeren Anwendung geführt zu haben. So auch für ihre Zunahme bei Alkoholismus und (neben dem Hauptmotiv einer „Reifung") bei Psychopathie, die noch in eigenen Unterkapiteln besprochen wird. Die ‚Schocktherapie' zur Umstimmung bei PatientInnen mit affektiven Störungen scheint zwar plausibel – unklar bleibt jedoch auch hier (wie bei den anderen nichtluetischen Diagnosen) wie unter den Patienten mit dieser Diagnose eine Minderheit für eine Therapie mit Malariafieber ausgewählt wurde.

2.1.4.2.4 Malariafiebertherapie bei der Diagnose ‚Psychopathie'

Psychopathie wurde in den eingesehenen Publikationen von KlinikerInnen, die in Wien tätig waren, nicht als Indikation für die Malariafiebertherapie genannt, obwohl an der Wiener Klinik die Fiebertherapie bei jugendlichen Patienten mit dieser Diagnose häufig angewandt wurde. In 205 der in der Datenbank aufgenommenen 1.438 Fälle von PatientInnen mit einer Psychopathie-Diagnose (alleine oder kombiniert mit anderen Diagnoseeinträgen und mit einem Aufenthalt an der Klinik von mehr als vier Tagen) wurde eine Malariafiebertherapie gegeben (s. Tab. 1). Es waren jugendliche PatientInnen, da eine Therapie der Psychopathie bei älteren PatientInnen allgemein als nicht erfolgversprechend galt.[380]

Das Fehlen von Hinweisen auf die Malariakur nicht nur in den eingesehenen internationalen Publikationen des Untersuchungszeitraums, sondern vor allem auch in den Publikationen der Wiener Kliniker verwundert, da diese zur international heftig geführten Debatte um die Diagnose „Psychopathie"[381] und ihrer Behandlung mehrmals und ausführlich publizierten. Die folgenden Ausführungen gelten diesem Widerspruch zwischen Theorie und Praxis in der Therapiewahl und damit der Frage, warum und mit welchen Argumenten in den Publikationen

[379] „Es handelt sich um hysterische Mechanismen im Alkoholrausch bei cykloider Persönlichkeitsstruktur. Der Patient wurde einer Malariafieberkur unterzogen und wird mit heutigem Tage zur Truppe entlassen."
[380] Vgl. unten S. 114.
[381] Im von German E. BERRIOS/Roy PORTER herausgebenen Sammelband „A History of clinical psychiatry" (1995, ^{2}1999) wird im Kapitel „Personality Disorders. Clinical Section" (633-644) rückblickend über die verschiedenen Meinungen zur Psychopathie berichtet.

nur Psycho- und Gruppentherapie sowie „therapeutisch orientierte Nacherziehung“ der „unausgereiften Persönlichkeit“ von PsychopathInnen empfohlen wurde,[382] wohingegen in der klinischen Praxis viele, wenn auch eine Minderheit der PatientInnen mit einer Psychopathie-Diagnose, eine Malariafiebertherapie bekamen. Die Position der Wiener Kliniker in der Diskussion zur Diagnose Psychopathie soll beschrieben werden, bevor auf die Auswertung der PatientInnenakten der Wiener Klinik zur Malariafiebertherapie bei dieser Diagnose statistisch und mit Fallbeispielen eingegangen wird.

Hans Hoff und Willibald Sluga stellen 1962 zur Diskussion um die Psychopathie-Diagnose fest, dass der „Psychopathiebegriff“ „noch immer zu den schwierigsten Problemen der Psychiatrie“ gehöre. In den deutschsprachigen Ländern werde damit „ein Persönlichkeitszustand beschrieben […], der ungefähr zwischen Krankheit und Gesundheit“ stehe, in den englischsprachigen Ländern hingegen werde „die Existenz einer Psychopathie überhaupt geleugnet“[383] und dafür „der Ausdruck Chrakterneurose verwendet. Dadurch erhält der Zustand den Stempel des Krankhaften“. Hingegen werde in den deutschsprachigen Ländern „keineswegs als sicher angenommen, daß es sich hier um eine Krankheit handelt.“[384] Viele seien der Meinung, man könne „lediglich von einer Störung des Gleichgewichtes sprechen, wodurch diese psychopathischen Persönlichkeiten untauglich werden, sich den sozialen Forderungen des Lebens anzupassen.“[385] Mit dieser Charakterisierung wird deutlich, dass die Stabilisierung des psychischen Gleichgewichts im Sinn einer Anpassung an das

---

[382] Vgl. unten S. 116 das Zitat aus RINGEL/SOLMS/SPIEL, Die Therapie der Psychopathie (1960), S. 456.
[383] Das dürfte sich auf die Gegenwart (1962) beziehen. In den 1950er Jahren wurde der Begriff in *The Lancet* noch verwendet: vgl. den Artikel von JONES/STALLARD/HUNTER/BROOKS, The Psychopath and the Mental Health Bill (1959), zitiert unten S. 114 Anm. 415. Die Einschränkung bzw. Aufgabe der Diagnose wurden freilich schon damals diskutiert. SHORTER, Geschichte der Psychiatrie (1999), S. 445f. schreibt zur Situation in den USA, die zu den Bemühungen um eine Klassifikation und Ausdifferenzierung der Diagnosen beitrug: „Wenig gravierende Schwierigkeiten mit der eigenen Persönlichkeitsstruktur, die im Zivilleben praktisch nicht ins Gewicht fielen, waren im militärischen Umfeld [des 2. Weltkriegs] plötzlich von großer Bedeutung. Doch für sie standen nur Begriffe wie ‚psychopathische Persönlichkeit' zur Verfügung.“
[384] Vgl. BLEULER, Lehrbuch der Psychiatrie ([11]1969), S. 543: „die Übergänge zwischen Norm und Psychopathie sind fließend“. Ähnlich in BLEULER, Lehrbuch der Psychiatrie ([10]1960), S. 496f. Vgl. KRANZ, Psychopathie in ihrer Problematik (1953), S. 763: „Die Beziehung zum Krankheitsbegriff ergab [in seiner Untersuchung] die Notwendigkeit einer klaren Scheidung zwischen Psychopathie als Persönlichkeitsvariante und Psychose als Krankheit.“
[385] HOFF/SLUGA, Das psychopathische Syndrom (1962), S. 241.

soziale Umfeld Ziel der Behandlung war.[386] Dazu wurde mehrmals ‚strenge Erziehung' und Psychotherapie, sowie Arbeitstherapie[387] genannt, aber keine der ‚großen' Kuren.[388]

Die psychopathische Persönlichkeit charakterisiert Hoff bereits 1956 in seinem Lehrbuch der Psychiatrie als „ein Individuum ohne hemmende Angst, asozial, mit mächtigen Aggressionen und ungehemmten, unkoordinierten Triebregungen, […] ohne Streben nach sozialer Einordnung" und „nicht übertragungsfähig".[389] In dem von Hoff 1960 herausgegebenen Sammelband „Therapeutische Fortschritte in der Neurologie und Psychiatrie" schreiben dazu die drei Wiener Kliniker Erwin Ringel, Wilhelm Solms und Walter Spiel: die „ganze Persönlichkeit" des „Psychopathen" sei „erkrankt, unreif, in der Entwicklung zurückgeblieben" und „unfähig, innere Spannungen zu ertragen"; die ‚PsychopathInnen' seien „zu libidinösen Bindungen nur mangelhaft befähigt" und ihre „Gewissensfunktionen" seien „nur mangelhaft entwickelt"; es bestehe „keine Tendenz, sozial vorwärtszukommen"; „an Stelle der Anpassung an das Realitätsprinzip" herrsche „das Lustprinzip"; „das neurotische Angstgefühl" fehle. Die Psychopathie unterscheide sich von der Neurose durch eine „unreife Persönlichkeit" mit „mangelhaft entwickeltem Über-Ich".[390]

In der Therapie jugendlicher Psychopathie in der Gruppe – eventuell mit einer anschließenden Einzeltherapie – sieht Hans Hoff bereits 1951 in einem Vortrag in der *Aichhorn-Gesellschaft* die einzige Möglichkeit für einen beschränkten Erfolg, d. h. „gewiß" für „keine Heilung, aber doch [für] ein gewisses Maß einer guten sozialen Anpassung und eines Wegfalles krimineller Handlungen". „Zu jeder Behandlung" dieser PatientInnen sei „eine Übertragung

---

[386] Nach Birgitta BERNET, „Der bürgerliche Tod": Entmündigungsangst, Psychiatriekritik und die Krise des liberalen Subjektentwurfs um 1900 (2007), S. 128f., zeigt Psychopathie neben „Querulantenwahnsinn" als neue Krankheitsbilder der Psychiatrie nach 1900 eine „Ausweitung des psychiatrischen Blicks", der sich von „der Wahrnehmung und Beschreibung psychischer Krankheiten von der anatomischen und symptomatologischen Betrachtung gelöst hatte und sich an sozialen Erwartungen, normativen Vorgaben […] oder am juristischen Schuldbegriff zu orientieren begannen".

[387] Zur Arbeitstherapie als Therapie zur Integration im sozialen Umfeld, vgl. GERMANN, Arbeit als Medizin: Die „aktive Krankenbehandlung" 1930-1960 (2007), S. 195-233.

[388] Vgl. BLEULER, Lehrbuch der Psychiatrie ([10]1960), S. 496-498: er unterstreicht die „besondere Bedeutung" einer „langandauernde Erziehung" in „von Pädagogen geleitete Erziehungsanstalten oder psychiatrische Krankenhäuser", er erwähnt aber auch in einzelnen Fällen „psychoanalytische Behandlungen […], öfters eher eine psychotherapeutische Führung und beständige Beratung über die Gestaltung des Alltags." Grundsätzlich könne „die aktive Gestaltung der Lebenserfahrung in der ärztlichen Psychotherapie bei Psychopathen [mit gegebenen Erbanlagen] dasselbe leisten wie bei gesund Veranlagten, die das Leben in eine neurotische Entwicklung gedrängt hat."

[389] HOFF, Lehrbuch der Psychiatrie (1956), S. 691f. (Vorlesung: Psychopathie I).

[390] RINGEL/SOLMS/SPIEL, Die Therapie der Psychopathie (1960), S. 453f.: Die Neurose kennzeichne im Unterschied dazu eine „teilweise ausgereifte Persönlichkeit", ein „starre[s] Über-Ich", „starke neurotische Schuldgefühle", die Übertragungsfähigkeit und eine Aggression, die sich vorwiegend nach innen richtet.

notwendig“[391]. Diese Patienten mit ihren libidinösen Beziehungen, die „auf den eigenen Körper narzistisch beschränkt“ seien, seien jedoch „kaum zu solchen Übertragungen im positiven Sinn bereit“. [392]

Zur Kombination der vielen Symptome[393] im Krankheitsbild der Psychopathie[394] schlagen Hoff und Sluga 1962 vor, vom „psychopathischen Syndrom“ zu sprechen.[395] Sie gliedern „die große Zahl psychopathischer Persönlichkeiten“ anhand der unterschiedlichen Genese und Prognose in fünf Typen mit Fallbeispielen, denen unterschiedliche Therapien zugeordnet werden:

- Die „Kerngruppe der Psychopathie“ würden demnach kriminelle Jugendliche aus einem asozialen Elternhaus bilden, bei denen „Vererbung und Milieuverwahrlosung[396] zu gleichen Teilen eine Rolle spielen“. Ihnen wird keine positive Prognose gegeben.
- Bei der zweiten Gruppe[397] manifestiere sich „der Beginn als neurotische Entwicklung“, von der aber „die Funktion des Ich und damit das Gefüge der Gesamtpersönlichkeit betroffen werden, so daß schließlich […] auch die Über-Ich-Struktur gestört erscheint.“[398] Diese Fälle hätten eine sehr schlechte Prognose; hier würden therapeutische Gruppen versucht, die „zunächst einmal den Gruppentypus des ‚gang‘ imitieren“ und „schließlich einem Familienmuster angepaßt werden.“ In den

[391] SOLMS, Zum Psychopathieproblem (1951), S. 27 sieht ebenfalls „die Schwierigkeit in dem Kardinalpunkt jeder Psychotherapie, in der Übertragung“ und zitiert August Aichhorn, „daß es zum Überfließen von narzistischer Libido kommen kann, wenn der Patient den Eindruck hat, daß der Behandler ihm ähnlich sei und dieser ihm imponiert“.

[392] HOFF, Therapie der jugendlichen Psychopathie (1951), S. 209f. Vgl. unten S. 115-117 zum Problem, die verschlossenen PatientInnen ohne Krankheitseinsicht für das Gespräch mit dem Psychotherapeuten zu öffnen.

[393] Es sind Symptome, wie heftige Erregung, Verschlossenheit, Aggressivität, Gemütsschwankungen, keine Krankheitseinsicht, die sich mit jenen anderer hier zur Diskussion stehenden Krankheitsbilder überschneiden und die wohl aufgrund dieser Symptome zu ähnlichen Therapieentscheidungen bei den verschiedenen Diagnosen führten.

[394] HOFF/SLUGA, Das psychopathische Syndrom (1962), S. 241 tendieren in der Frage, ob Psychopathie überhaupt eine Krankheit oder nur eine Störung des Gleichgewichts ist, zu letzterem.

[395] HOFF/SLUGA, Das psychopathische Syndrom (1962), S. 241. Vgl. SOLMS, Zum Psychopathieproblem (1951), S. 27, der Psychopathie bereits damals „nicht für eine Einheit, sondern für ein Syndrom“ hielt, und deshalb „für eine dynamische und genetische Untersuchung jedes Falles“ plädierte.

[396] HOFF/SLUGA, Das psychopathische Syndrom (1962), S. 268: Die Verwahrlosung könne, „den jeweiligen Umständen entsprechend, eine Notstands- oder Wohlstandsverwahrlosung sein.“ Vgl. zu diesem Thema auch SLUGA, Jugendpsychiatrische Tätigkeit bei Verwahrlosten (1962), S. 212, der auf den starken Anstieg der Jugendkriminalität Ende der 1950er Jahre hinweist; zu diesem Phänomen vgl. Sepp SCHINDLER, Jugendkriminalität (1968), S. 19 (der Autor war in den 1950er Jahren in der Bundesanstalt Kaiserebersdorf tätig, später Professor für Psychologie an der Universität Salzburg).

[397] Es ist hier die mit drei Fallbeispielen von „psychopathischen[n] Zustandsbilder[n]“, deren „Beginn sich als neurotische Entwicklung manifestierte“, am ausführlichsten beschriebene Gruppe. HOFF/SLUGA, Das psychopathische Syndrom (1962), S. 248-256 beziehen sich auf BERNER/SOLMS, Die Entwicklung einer Neurose (1954), S. 242-252 und auch auf den dort beschriebenen Fall einer 27jährigen Patientin.

[398] HOFF/SLUGA, Das psychopathische Syndrom (1962), S. 248.

Fällen mit neurotischer Vorgeschichte sollte auch versucht werden eine Einzelpsychotherapie einzuleiten.[399]

- Die dritte Gruppe zeige „zunächst wieder alle Symptome der Psychopathie mit asozialen und aggressiven Tendenzen". „Die Besonderheit dieser Gruppe liegt jedoch in einer bestimmten Form von elektroenzephalographisch nachweisbaren Störung. Daneben ist eine zweite Besonderheit, daß diese Fälle sich im Laufe der Jahre bessern. […] auch ihr EEG normalisiert sich. Wir müssen annehmen, daß es sich bei diesen Fällen um Reifungsstörungen des Gehirns […] handelt."[400] Die Prognose für diese Gruppe sei sehr positiv: „Durch intensive erzieherische Maßnahmen, die durch psychotherapeutische Kurzbetreuungen unterstützt wurde", sei im Beispielsfall „eine Stabilisierung im Verhalten" erreicht worden und im Gegensatz zum EEG-Befund mit 17 sei jener mit 21 Jahren normal gewesen.[401] Bei PatientInnen dieser Gruppe müsse verhindert werden, dass sie sich „in jener Periode ihrer Entwicklungsstörung […] kriminelle und asoziale Lebensmuster zurechtlegen, oder daß ihr sozialer Abstieg ein solcher ist, daß der Weg zurück unmöglich wird."[402]
- Die vierte Gruppe würden jene PatientInnen bilden, „bei denen es nach der Encephalitis zu einer Änderung des Charakters gekommen ist."[403] Bei ihnen könne „ein Versuch auf der Basis des Neuerlernens bedingter Reflexe […] erfolgreich sein."[404]

---

[399] HOFF/SLUGA, Das psychopathische Syndrom (1962), S. 269f.

[400] HOFF/SLUGA, Das psychopathische Syndrom (1962), S. 256f.: „und zwar [Reifungsstörungen] ganz bestimmter Anteile […] Wir glauben, […] daß offenbar Störungen in den Verbindungen tiefer Stammganglienabschnitte und zentrozephaler Bereiche mit den Temporallappenregionen vorliegen, die ihrerseits das Resultat eines gestörten Reifungsprozesses darstellen."

[401] Lt. WISSFELD/KAINDL, Wert abnormer EEG-Befunde bei psychopathischen Persönlichkeiten (1961), S. 58 stellten Mitarbeiter von D. Hill bei ihren Kontrolluntersuchungen „ein parallel mit einer Besserung des psychopathologischen Bildes verlaufendes Rückgehen der dysrhythmischen Kurvenverläufe und fokalen EEG-Störungen" fest. Sie zitieren dazu REY/POND/EVANS, Clinical and Electroencephalographic Studies of Temporal Lobe Function (1949), S. 891 und S. 903.

[402] HOFF/SLUGA, Das psychopathische Syndrom (1962), S. 268f.

[403] HOFF/SLUGA, Das psychopathische Syndrom (1962), S. 261 (vgl. S. 268). Ähnlich bereits HOFF, Lehrbuch der Psychiatrie (1956), S. 271f. (Vorlesung: Epilepsie I) und S. 181 (Vorlesung: Exogener Reaktionstyp).

[404] KAUDERS, Psychopathie und Neurose als Grenzgebiete der Nervenheilkunde (1936), S. 4, empfahl bei sehr frühen Fällen von Persönlichkeitsveränderungen nach einer Encephalitis eine rasche Indikation der Psychotherapie. „In den Anfangsstadien dieser psychopathischen Charakterveränderungen [„als Folge einer durchgemachten Encephalitis epidemica Economo"] und nur in diesen läßt sich nämlich beobachten, daß die Kranken ihr eigenes psychopathisches Handeln und Affekt ablehnen, es als etwas ihnen Aufgezwungenes und wesensmäßig Fremdes empfinden, worin ein grundlegender Unterschied gegenüber den echten Psychopathieformen, dagegen eine Analogie zu dem Verhalten vieler Neurotiker liegt." Vgl. in Fällen ohne diese Vorgeschichte KAUDERS, Psychopathie und Neurose als Grenzgebiete der Nervenheilkunde (1936), S. 5: „Der Weg der Behandlung der Psychopathie heißt Erziehung […] im Sinne einer aktiven Charakterformung und seiner Gestaltung, unter dem Ziele der Bewältigung der sozialen und sittlichen Pflichten des Lebens."

- Die PatientInnen der 5. Gruppe, die „oft als schizoide Psychopathie bezeichnet“ werde, fallen durch „die Stärke der Introversion und Abkehr von der Außenwelt sowie der Gefühlskälte“ auf. „Ein beträchtlicher Prozentsatz von ihnen“ habe „oft kurzdauernde schizophrene Psychosen durchgemacht.“[405] Sie seien „ebenfalls behandlungsfähig. „Durch die Kombination von biologischen Behandlungsmethoden, z.B. durch die Verwendung von neueren Neuroleptica[406] und gruppenpsychotherapeutischer Behandlung“ könne „eine Besserung des Zustandsbildes“ herbeigeführt und „so neue Lebenseinstellungen“ angestrebt werden, „die sozial akzeptabel sind“.[407]

Zu den Beobachtungen im EEG bei der Diagnose Psychopathie, mit der die Gruppe 3 charakterisiert und auf die noch in der Auswertung der Krankenakten eingegangen wird, gab es im Untersuchungszeitraum eine rege Diskussion.[408] Nach Cornelius Borcks „Kulturgeschichte der Elektroenzephalographie“ (2005) war, nach dem Erfolg des EEG bei der Epilepsie-Diagnose, vielfach „nach pathologischen Kurvenmustern bei Menschen mit Persönlichkeitsstörungen sowie bei verhaltensgestörten Kindern und Jugendlichen“ gesucht worden.[409] 1963 schreibt der britische Psychiater Heaton-Ward, die „wahren Psychopathen“ seien sehr selten und ihre Erkrankung hätte „probably a genetically determined organic basis“: Obwohl es keine eindeutigen EEG-Veränderungen bei der „Psychopathie“ gäbe, habe die „Royal Commission on the Law Relating to Mental Illness and Mental Deficiency“ von 1957 erwähnt, dass in 60% der Fälle anormal langsame Hirnwellen zu sehen wären, wie sie bei Kindern, nicht jedoch bei Erwachsenen im wachen Zustand auftreten würden.[410] Reifungsstörungen zu beheben, wie sie allgemein bei jugendlichen ‚PsychopathInnen‘ angenommen und zur Gruppe 4 auf organische Schädigungen des Gehirns durch

[405] HOFF/SLUGA, Das psychopathische Syndrom (1962), S. 264f.: „Die Symptome, die uns als Defekt imponieren“, seien „in Wirklichkeit Gleichgewichtszustände in der Persönlichkeitsabwandlung aktiver schizophrener Symptome“, die „sich zu asozialen Bildern vereinigen“ können. Bezug genommen wird auf GASTAGER/SCHINDLER, Rehabilitationstherapie bei Schizophrenen (1961), passim (vgl. unten S. 190f.).
[406] Die Neuroleptika dürften bei der „schizoide[n] Psychopathie“ aufgrund der Schizophrenie-ähnlichen Symptome (vgl. oben „aktiver schizophrener Symptome“) gegeben worden sein und nicht wegen Psychopathie-Symptomen.
[407] HOFF/SLUGA, Das psychopathische Syndrom (1962), S. 269.
[408] Die Diskussion seit den 1930er Jahren fassen die Frankfurter Kliniker E. Wissfeld und E. Kaindl 1961 zusammen: WISSFELD/KAINDL, Wert abnormer EEG-Befunde bei psychopathischen Persönlichkeiten (1961), S. 62. In diesem Literaturbericht werden keine Therapien erwähnt. Nur einmal, zur „epileptoiden Psychopathie“, wird davon gesprochen, „daß man gelegentlich die endogen bedingten morosen Verstimmungszustände dieser Psychopathen mit gutem Erfolg mit einem Elektrokrampf therapieren kann, ähnlich dieser Therapie bei epileptischen Dämmerzuständen.“
[409] BORCK, Hirnströme (2005), S. 18.
[410] HEATON-WARD, Psychopatic Disorder (1963), S. 123.

Encephalitiden[411] zurückgeführt werden, waren der Ansatzpunkt der Therapie. Ihr erstes Problem war, einen Zugang zum / zur übertragungsunfähigen Patienten/in zu finden.

Zur Genese und zum Ansatz für eine Therapie schreiben Ringel, Solms und Spiel 1960: Man sehe „sehr häufig bei den Betreffenden das Wandern von Heim zu Heim", frühkindliche „Hospitalisierung" und „Kind-Mutter-Separation", was manchmal zu mangelhafter Entwicklung der Liebesfähigkeit, des Über-Ichs, der Persönlichkeit führe.[412] Und sie meinen: „Erst die Einbeziehung psychodynamischer Gesichtspunkte […], ermöglichte die Entwicklung wirklicher therapeutischer Maßnahmen gegen die ‚Erkrankung' Psychopathie."[413] Vor allem die lebensgeschichtlichen Ursachen der ‚psychopathischen' Entwicklung wurden als Ansatz für eine individuell abgestimmte Therapie gesehen.

Hoff und Ringel nannten in einem Artikel von 1956 als „einzig richtige Therapie": „Zuerst muß eine Nachreifung der Persönlichkeit einsetzen, die nur durch Aufbau einer allmählich sich verstärkenden, persönlichen Bindung erreicht werden kann."[414] Erst dann sei eine psychotherapeutische Behandlung möglich. Die Therapie sei bei dieser Diagnose „an und für sich schon äußerst schwierig" und würde ab dem 17. und 18. Lebensjahr „immer problematischer" und bei Erwachsenen dürften Erfolge „wohl ausgesprochene Einzelfälle sein".[415] Als besonders wichtig für einen Erfolg betonen sie die frühe Diagnose (die „erstmalig etwa in der Pubertätszeit möglich ist") und eine frühe Behandlung der Psychopathie.[416]

Zur Früherkennung veranlasste Hoff 1952 den Leiter der Kinderstation, Walter Spiel, zu einer Langzeitstudie über die Anfangssymptome und Verläufe von kindlichem Fehlverhalten, um durch deren Vergleich „jene Momente herauszuarbeiten, die eine prognostische Aussage

---

[411] Vgl. oben S. 112.
[412] RINGEL/SOLMS/SPIEL, Die Therapie der Psychopathie (1960), S. 454f.
[413] Ebd., S. 455f. Sie nennen fünf „genetische Faktoren einer solchen psychopathischen Entwicklung": „1. Das Fehlen von libidinösen Beziehungen (oder ihre schwere Störung) in der frühesten oder frühen Kindheit. [...] 2. Ungünstige Identifikationsobjekte. [...] 3. Erb- und konstitutionelle Faktoren. [...] 4. Möglicherweise sind auch organische Faktoren beim Zustandekommen solcher unreifen Persönlichkeiten beteiligt. [...] 5. [...] die konstellierenden Faktoren [...] gleich bleibend monoton über Jahre wirksam [...], was zum Großteil mit der von vornherein festgelegten abnormen Charakterstruktur der Eltern zusammenhängt."
[414] Dazu verweisen Hoff und Ringel auf die (pädagogischen und psychotherapeutischen) Möglichkeiten, die „uns als erster Aichhorn gezeigt [hat]".
[415] So auch RINGEL/SOLMS/SPIEL, Die Therapie der Psychopathie (1960), S. 460. In England sollten laut einer Gesetzesvorlage zur „Mental Health Bill" eine Therapie nur PatientInnen unter 21 Jahren bekommen: JONES/STALLARD/HUNTER/BROOKS, The Psychopath and the Mental Health Bill (1959), S. 566.
[416] HOFF/RINGEL, Anfänge der Psychopathie (1956), S. 424f.

gestatten".[417] In den drei Publikationen dazu, findet sich einleitend zwar ein allgemeiner Hinweis auf „moderne tiefenpsychologisch orientierte Therapie[n]", um „kindliche Fehleinstellungen zu verändern",[418] und weitere kurze Hinweise auf Psychotherapie bei den PatientInnen im kindlichen und dann jugendlichen Alter, längerfristige Beratung und administrative Maßnahmen (Heimeinweisung oder Umschulung)[419] – nicht jedoch auf die Anwendung einer körperlichen Therapie, weder einer der ‚großen Kuren', noch der neuen Medikamente.[420]

1960 schreiben Ringel, Solms und Spiel zur Therapie der Psychopathie: „Die einzige Möglichkeit, eine solche mangelhaft entwickelte Persönlichkeit zum Nachreifen zu bringen, scheint eine therapeutisch orientierte Nacherziehung zu sein", um „eine Nachentwicklung der unausgereiften Persönlichkeit herbeizuführen."[421] Aufgrund der fehlenden Krankheitseinsicht sei es jedoch sehr schwierig, „den Patienten zu einem Nachholen der früher versäumten emotionalen Beziehungen zu bringen." In einer Kombination von Einzelbehandlung (wozu sie auf die Erfahrungen von August Aichhorn verweisen) und Gruppentherapie[422] könne mit einer langsamen „Anpassung an das Bessere" zwar keine Heilung, aber „ein gewisses Maß an sozialer Anpassung" erreich werden.[423] Es waren psychodynamische und psychotherapeutische Modelle „therapeutisch orientierte[r] Nacherziehung" der „unausgereiften Persönlichkeit" von ‚PsychopathInnen'.[424] Sie berichteten, dass an der

---

[417] SPIEL, Beitrag zur Frage der psychopathischen und neurotischen Entwicklung im Kindesalter (1958), S. 291f. Es ging um die Frage, welche „Umstände, Bedingungen und Ereignisse" es sind, „die in einem Fall zur Entwicklung von psychopathischen Eigenschaften, im anderen zu neurotischen Mechanismen führen": SPIEL, Nachuntersuchungsergebnisse psychopathischer und neurotischer Entwicklungen (1967), S. 1166.
[418] SPIEL, Über den Beginn neurotischer und psychopathischer Entwicklungen im Kindesalter (1954), S. 21.
[419] SPIEL, Nachuntersuchungsergebnisse psychopathischer und neurotischer Entwicklungen (1967), S. 1167; SPIEL, Beitrag zur Frage der psychopathischen und neurotischen Entwicklung im Kindesalter (1958), S. 297.
[420] In den Überlieferten Akten der Kinderstation mit der Diagnose Psychopathie, psychopathische Syndrom u. ä. (51 Fälle in der Datenbank zur Kinderstation) wurden jedoch auch medikamentöse Behandlungen und Kinder im Alter deutlich vor der Pubertät mit dieser Diagnose genannt; die Erweiterung könnte darauf zurückzuführen sein, dass hier auch Kinder mit organischen Erkrankungen bzw. Schädigungen und dieser Diagnose einbezogen sind, die in der Untersuchung Spiels, in der es um frühe Anzeichen einer Fehlentwicklung ging, explizit ausgeschlossen waren.
[421] RINGEL/SOLMS/SPIEL, Die Therapie der Psychopathie (1960), S. 456.
[422] Vgl. HOFF/SPIEL, Die Dynamik der kriminellen Psychopathie Jugendlicher (1952), S. 33: bereits hier wird die Gruppentherapie für kriminelle ‚PsychopathInnen' in einer sorgfältig zusammengestellten Gruppe, die eine Anpassung an die / den Bessere/n in der Gruppe ermöglicht, als einzige Therapie erwähnt.
[423] RINGEL/SOLMS/SPIEL, Die Therapie der Psychopathie (1960), S. 459. Vgl. übereinstimmend HOFF, Therapie der jugendlichen Psychopathie (1951), S. 209f., zitiert oben S. 111.
[424] Solms, Ringel und Spiel waren freilich mit Gastager und Schindler jene Mitarbeitern Hoffs, die mit speziellen psychotherapeutischen Methoden arbeiteten. Aber auch HOFF, Lehrbuch der Psychiatrie (1956), S. 710 (Vorlesung: Psychopathie II, Perversion, Simulation und Monomanie) schrieb von einer Notwendigkeit der Behandlung jugendlicher PsychopathInnen „in eigenen Anstalten" mit Gruppentherapie und von Erfolgen, die die Wiener Psychiatrie mit ihrer Arbeit „in letzter Zeit in den Heimen für jugendliche, schwer erziehbare

praktischen Umsetzung dieser Ansätze sowie an der Ausarbeitung entsprechender Methoden in den Bundeserziehungsanstalten Kaiserebersdorf (für männliche Jugendliche) und Wiener Neudorf (für weibliche Jugendliche) gemeinsam mit den ErzieherInnen gearbeitet werde.[425]

Bei PatientInnen mit Psychopathiediagnose und ohne Malariafiebertherapie ist dieser Standpunkt manchmal auch zu erkennen, so die Gruppentherapie bei der Patientin P20342. Sie wurde 1951/52 als 16jährige mit der Diagnose „Psychopathie" bei ihrem langen 243tägigen Aufenthalt laut Fieberkurve mit keiner körperlichen Kur (und zwar, ebenso wie andere PatientInnen mit dieser Diagnose, auch mit keiner medikamentösen Therapie) behandelt. Die Gruppentherapie wurde wie üblich nicht in der Fieberkurve eingetragen, aber bei ihr in den *Decursus*-Aufzeichnungen mehrmals erwähnt.

Es sind erzieherische und psychotherapeutische Maßnahmen. Körperlich Therapien werden in der eingesehenen Literatur bei ‚Psychopathie' kaum genannt: Hoff und Sluga 1962 erwähnen eine körperliche Therapie mit Neuroleptica nur bei der Gruppe „des psychopathischen Syndroms auf der Basis einer durchgemachten schizophrenen Geistesstörung" und dies in Kombination mit „gruppenpsychotherapeutischer Behandlung". Wissfeld und Kaindl erwähnen in bestimmten Fällen der „epileptoiden Psychopathie" die Elektrokrampftherapie.[426] Nur in einer kleineren Arbeit von Spiel, die er 1957 über seine Erfahrungen in der Bundeserziehungsanstalt Kaiserebersdorf für Ärzte in der Justizverwaltung publizierte, werden noch weitere medikamentöse Therapien genannt: bei „Cerebralschaden" und besonders bei „Antriebsstörungen" würden sie „Präparate aus der Rauwolfia-Reihe und der Biobamate geben".[427] Das könnte sich mit Antriebsstörungen auf schizoide Psychopathie bezogen haben (nach Hoff und Sluga die oben genannten Patientinnen der 5. Gruppe der Psychopathie).[428]

Mädchen und Knaben [in Wiener Neudorf bzw. in Kaiserebersdorf, GH] nach diesen Prinzipien [Hoff bezog sich vor allem auf Aichhorn]" hatte.

425 RINGEL/SOLMS/SPIEL, Die Therapie der Psychopathie (1960), S. 460. Zu den beiden Anstalten, in deren Betreuung die Klinik einbezogen war, vgl. den Exkurs unten S. 131 – 141.

426 Siehe oben S. 113 Anm. 408.

427 SPIEL, Über jugendpsychiatrische Erfahrungen im Jugendstrafvollzug (1956), S. 32; vgl. zu diesem Artikel auch unten S. 133.

428 Der Konnex zur Psychopathie scheint hier deutlich durch das Thema gegeben zu sein: es handelte sich um schwererziehbare, straffällige Jugendliche.

In den zitierten Publikationen zur Psychopathie wurde der „Aufbau einer allmählich sich verstärkenden, persönlichen Bindung“[429] zwischen Patientin/en und Therapeutin/en als Voraussetzung für einen Erfolg der „Nacherziehung“ genannt. Nicht erwähnt wird jedoch, wie diese Beziehung mit dem / der zu sozialen Kontakten unfähigen ‚Psychopathen/in‘ ermöglicht werden sollte. Die Schlussfolgerung und Hypothese liegt jedoch nahe, dass die Öffnung durch die Malariafiebertherapie in ihrer Wirkung als eine der ‚Erschütterungstherapien‘, wie Manfred Bleuler sie 1969 beschreibt, erreicht werden sollte[430] - vergleichbar der Wirkung der Insulinkomatherapie bei Schizophrenen, deren Ende Hoff 1956 im Lehrbuchabschnitt zur „Schocktherapie“ als den idealen Zeitpunkt für den Beginn einer Psychotherapie bezeichnete.[431]

1999, im historischen Rückblick bezog sich der Medizinhistoriker Daniel Helmchen in seiner Einschätzung, dass bei der Malariafiebertherapie „zumindest bei nichtparalytischen Psychosen“ die „‚Erschöpfung’ des Patienten“ und die damit „einhergehende, intensive Interaktion der Patienten mit den Helfenden“, den PflegerInnen und ÄrztInnen, als alleinige Ursache für die Therapieerfolge anzusehen sei,[432] auf die beiden Psychiater des frühen 20. Jahrhunderts Emil Kraepelin und Jacob Klaesi. Kraepelin habe 1909 „zum Remissionsmechanismus bei Fieberkuren“ gemeint, dass „auch das Gefühl der Hilfsbedürftigkeit […] die Kranken vielleicht zugänglicher“ mache.[433] Klaesi habe „die gelegentlichen Besserungen bei Schizophrenen nach Fieber in der Hauptsache wenigstens als Wirkung des während der Bettlägrigkeit und Pflegebedürftigkeit wiedergewonnenen besseren Rapports des Kranken mit dem Arzt“ bezeichnet und die gleiche Formulierung in der Erläuterung des Heilungsmechanismus der von ihm eingeführten Schlafkuren mit Somnifen gebraucht.[434] Demnach machte die Therapie die PatientInnen hilfsbedürftiger und dadurch – aus der Perspektive der Ärzte/innen und PflegerInnen – zugänglicher. Die Therapie zeigte

---

429 HOFF/RINGEL, Anfänge der Psychopathie (1956), S. 424f.
430 Vgl. oben S. 29-31, (nach Bleuler) S. 165f. und nach Hoff im Insulinkapitel S. 200f.
431 HOFF, Lehrbuch der Psychiatrie (1956), S. 519f. (Vorlesung Hoff, Schocktherapie) zitiert im Insulinkapitel unten S. 200f.
432 HELMCHEN, Malariaimpftherapie der Progressiven Paralyse in den Wittenauer Heilstätten (1999), S. 446. Zur intensiven Behandlung während der Malariakur und ihrer möglichen Wirkung, vgl. EY/BERNARD/BRISSET, Manuel de Psychiatrie ([1]1960), S. 720. Es ist das gleiche Argument für eine mögliche Wirkung der Insulinkomatherapie, die auch in der Insulinkomatherapie-kritischen Untersuchung von ACKNER/HARRIS/OLDHAM, Insulin Treatment of Schizophrenia (1957), S. 611 genannt wird – vgl. im Insulinkapitel unten S. 177.
433 HELMCHEN, Malariaimpftherapie der Progressiven Paralyse in den Wittenauer Heilstätten (1999), S. 446.
434 Ebenda, S. 445. Kraepelin und Klaesi sehen darin freilich nur einen Teil des Wirkmechanismus der Therapie. Vgl. zur Wirkung der Schlafkur auch MEIER/BERNET/ DUBACH/GERMANN (Hrsg.), Zwang zur Ordnung. Psychiatrie im Kanton Zürich (2007), S. 104.

also soziale Effekte, die für den therapeutischen Prozess im Sinne einer sozialen Anpassung genutzt werden konnten.

In den Wiener PatientInnenakten wurde kein direkter ärztlicher Hinweis auf diese für Gespräche öffnende Wirkung der Malariafiebertherapie gefunden. Ein Hinweis darauf findet sich im Brief einer Mutter an den behandelnden Kliniker im Akt des 1965 16jährigen Patienten P197 mit der Diagnose „Psychopathie“. Sie schrieb nach ihrem Besuch, bei dem ihr Sohn wieder ihr gegenüber ablehnend reagiert hatte: „Ich stehe der Situation ziemlich fassungslos gegenüber, denn unmittelbar nach der Fiebertherapie schien die Tür offenzustehen. Anscheinend fiel sie zu, als ich dem Buben die Bitte abschlagen musste, wieder ganz zu Hause wohnen zu dürfen und sein Studium nicht vom Internat aus fortsetzen zu müssen.“ Aus einzelnen Hinweisen von KlinikerInnen in den PatientInnenakten dürfte jedoch ebenfalls, wenn auch nur indirekt und hypothetisch zu erschließen sein, dass von der Fiebertherapie eine ‚Öffnung‘ der PatientInnen erwartet wurde: so beispielsweise aus nicht seltenen Hinweisen auf eine psychische „Umstimmung“. Naheliegend scheint die Schlussfolgerung bei den Erwähnungen im nächsten Kapitel 2.1.4.2.5 der Malariafiebertherapie in der ‚Vorbereitung‘ einer Alkohol-Entzugskur zu sein.[435]

Ein therapeutischer Ansatz ergab sich bei ‚Psychopathie‘ vor allem aus den sozialen Komponenten der Genese, weshalb zur Behandlung fast ausschließlich Milieuwechsel, Erziehung und Psychotherapie genannt wurden. In den Fieberkurven wurde häufig regelmäßige Arbeitstherapie eingetragen, jene Therapie, die sozial angepasstes Verhalten bewirken sollte. Es wurden jedoch auch körperliche Ursachen der ‚Psychopathie‘ diskutiert – Störungen als Folge von Encephalitiden, zu denen im EEG feststellbare Auffälligkeiten als Hinweise auf Reifungsstörungen interpretierte wurden. Dazu finden sich auch Hinweise in den eingesehenen Krankenakten; in Fallbeispielen wird darauf noch eingegangen werden.

[435] Zur Malariakur bei Alkoholikern vgl. unten S. 145 – 150.

A) Die Praxis an der Wiener Klinik zur Malariatherapie bei Psychopathie (statistische Daten und Fallbeispiele)

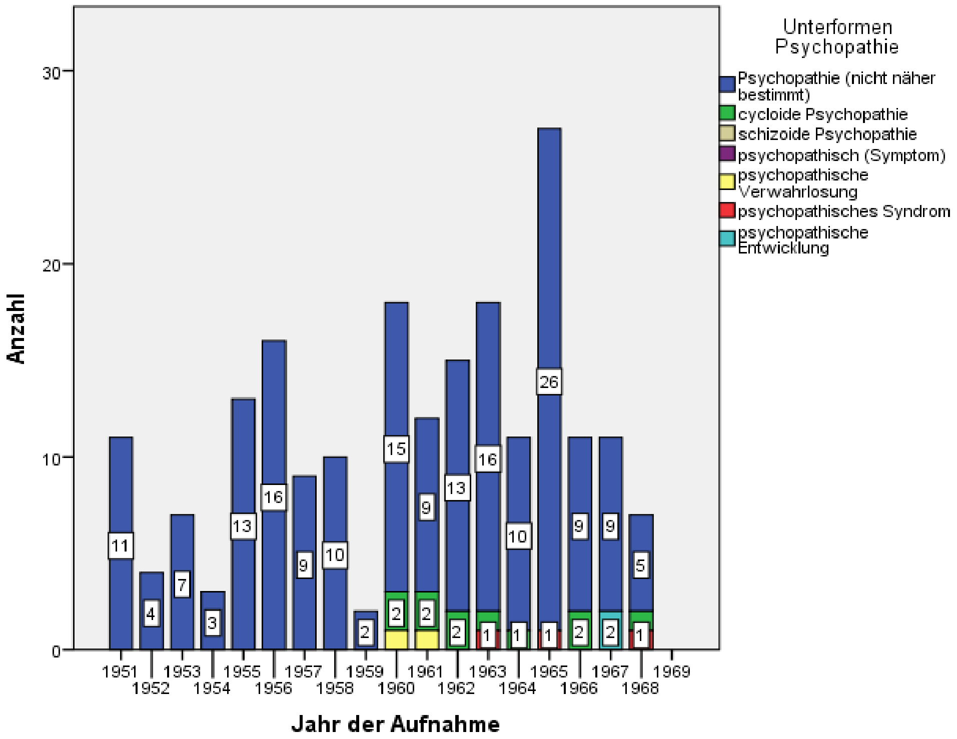

[redacted]

Abb. 6 Gestapeltes Balkendiagramm, Malariafiebertherapie + Unterformen der Psychopathie (1951-1969), Pat. länger als 4 Tage stationär, n = 205

In 205 Fällen der in der Datenbank eingetragenen 1.438 Aufenthalte von PatientInnen mit einer Psychopathie-Diagnose wurde im Zeitraum von 1951 bis 1969 eine Malariafiebertherapie gegeben (s. Tab. 1), d.h. dass die Malariafiebertherapie in 14,2% der stationär länger als vier Tage mit einer Psychopathie-Diagnose Aufgenommen angewandt wurde. Die Anwendung der Malariafiebertherapie bei diesen 205 PatientInnen betraf fast ausschließlich männliche Jugendliche[436] und war sehr unregelmäßig über die Jahre verteilt. Die Diagnose Psychopathie wurde zwar in den 1960er Jahren bei PatientInnen mit einem Klinikaufenthalt von mehr als vier Tagen seltener, blieb jedoch bei jungen Männern als Indikation für die Malariafiebertherapie in diesem Jahrzehnt besonders häufig, häufiger als in den 1950er Jahren. Eine Erklärung dafür findet sich weder in den eingesehenen

[436] Lt. Datenbank bekamen von insgesamt 13 Frauen, die mit einer anderen Diagnose als Neurolues die Malariafiebertherapie bekamen, drei Patientinnen mit einer Psychopathie-Diagnose eine Malariatherapie: Vgl. unten S. 127f.

Publikationen, noch in den Krankenakten.[437] Ebenso wenig für die auffällige Spitze, welche die Anwendung bei dieser Patientengruppe 1965 hatte, als von 53 stationär länger als vier Tage aufgenommenen PatientInnen mit einer Psychopathie-Diagnose 27 Patienten eine Malariafiebertherapie bekamen (im Jahr davor waren es von 53 elf PatientInnen, im Jahr danach von 36 ebenfalls elf).

Das jugendliche Alter der PatientInnen, die mit der Diagnose Psychopathie mit einer Malariafiebertherapie behandelt wurden, ist aus der Wiener und internationalen Literatur nachvollziehbar[438] – nicht die fast ausschließlich Anwendung bei männlichen Jugendlichen.

Der Fall der 17jährige Patientin P194 lässt darauf schließen, dass bei Patientinnen in erster Linie die in der Literatur genannten Therapien angewandt wurden:[439]

> Sie wurde bei ihrem zusammengerechnet 111 Tage langen (nur durch eine 3tägige Flucht unterbrochenen) 1. und 2. Aufenthalt[440] 1963 mit der Diagnose „Psychopathie" „medikamentös[441] und psychotherapeutisch" behandelt.[442] Dazu wird in fünf *Decursus*-Aufzeichnungen zum sehr positiv beurteilten Verlauf der Arbeitstherapie berichtet – „im Insulin", d.h. im Zimmer, in das die PatientInnen täglich früh am Morgen zur Insulinkomatherapie[443] gebracht wurden: Sie freue sich, dass ihr diese Aufgabe übertragen wurde, und sie scheine ihr gewachsen zu sein. Die erste Anamnese ist deutlich nach dem Bild der psychischen Störung des psychopathischen Syndroms geschrieben: intelligent,[444] nicht bindungsfähig,[445] ohne Angst; sie war ohne Erlaubnis der von ihr selbst als sehr nachgiebig geschilderten Eltern oft über das Wochenende von zu Hause weggeblieben und diese Vorfälle führten zu ihrer ersten Aufnahme. Im September des gleichen Jahres kam sie, nachdem sie im Sommer wieder „zu Hause nur

---

[437] Auf eine geänderte Indikation bei ‚Reifungsrückständen' könnte der Fall des Patienten P513 hinweisen, der 1956 als 15jähriger mit der Diagnose Psychopathie, dem Symptom Pseudoporiomanie und einem „mässig abnorme[n]" EEG, das auf „Immaturität" schließen ließ, keine Malariafiebertherapie bekam, wohl aber mit Psychopathie und Pseudoporiomanie bei seiner Aufnahme 1960.
[438] Vgl. oben S. 114 Anm. 415 und 416.
[439] Sie ist eine von drei Patientinnen mit der Diagnose Psychopathie, die mit einer Malariakur behandelt wurden.
[440] Bei der Wiederaufnahme nach der Flucht aus der Klinik heißt es in der Anamnese: „Angst vor eventueller Bestrafung bestand nur anfangs. Nun glaubt Patientin zu erkennen, dass es die Ärzte nur gut mit ihr meinen, lediglich helfen und nicht sie böswillig inhaftieren wollen."
[441] Lt. Fieberkurve und *Decursus*-Eintragungen erhielt sie fortlaufend 3x 10mg Librium (Tranquilizer mit dem Wirkstoff Chlordiazepoxid), aber keine weiteren Medikamente.
[442] Zu diesen früheren Aufenthalten sind in den Fieberkurven Arbeitstherapien und in den *Decursus*-Eintragungen auch Psychotherapien genannt. Letzteres wird auch in der „Zwischenanamnese" am Anfang der 3. Aufnahme genannt.
[443] In der Insulintherapie waren die PatientInnen teils im Koma sehr unruhig und schrien. Die Insulinkur wurde als die intensivste der „heroischen" Therapien beschrieben, deren Anwendung Otto Pötzl als Leiter der Wiener Klinik in den 1930er Jahren mit der Schwere der Krankheit Schizophrenie rechtfertigte.
[444] Überdurchschnittliche Intelligenz wird oft bei PatientInnen mit der Diagnose Psychopathie erwähnt – aber auch ihre Kombination mit Schwachsinnsformen, die ein psychopathisches Syndrom lt. HOFF/SLUGA, Das psychopathische Syndrom (1962), S. 267f. ‚verschärfen' könne.
[445] Es tue „ihr leid, dass sie zu den Eltern keine Liebe aufbringen kann und will, Patientin versteht sich auch nicht recht mit Freunden bzw. Freundinnen, sie versucht vergeblich sich an einen Menschen anzuklammern, der sie verstünde, das gelang bisher nie."

schwer haltbar“ gewesen war, mit der Diagnose „psychopathisches Syndrom“ „über die Privatordination von Herrn Prof. Hoff zur neuerlichen Aufnahme und soll jetzt eine Fieberkur durchmachen“.[446] Das dürfte auf eine persönliche Anweisung Hoffs hinweisen und sehr rasch[447] wurde die Fieberkur mit 8 Fieberzacken[448] durchgeführt. In ihrem 5monatigen 3. Aufenthalt sind keine Medikamente eingetragen, erst im letzten Monat Arbeitstherapie. Psychotherapie (Gruppentherapie, Individualtherapie) dürften wie beim ersten Aufenthalt gemacht worden sein – aber, wie fast durchwegs auch bei anderen PatientInnen, ohne Vermerk in der Fieberkurve.

Wie bei den anderen nicht-luetischen Diagnosen, wurden die meisten der PatientInnen, die mit der Diagnose ‚Psychopathie‘ in der Wiener Klinik aufgenommen worden waren, innerhalb der ersten vier Tage entlassen oder auf eine andere Psychiatrie (vor allem *auf den Steinhof*) überwiesen. So wurden im Mustermonat April 1964, zu dem alle Akten[449] der Männerabteilung (d. h. auch jene, die nicht den Einschlusskriterien – eine der fünf Diagnosen, länger als 4 Tage – entsprachen) in die Datenbank eingegeben wurden, von insgesamt 190 Patienten mit verschiedenen Diagnosen und unterschiedlicher Aufenthaltsdauer, 20 mit einer Psychopathie-Diagnose (oft kombiniert mit Hinweisen auf Suizidversuch, Alkohol etc.) aufgenommen. Alle bis auf zwei Patienten wurden innerhalb von vier Tagen überwiesen oder entlassen.

Einer der beiden, der 23jährige Patient P6569 (Hilfsarbeiter) mit der Diagnose „*SMV, Psychopathie, Alkoholmissbrauch*“, wurde ebenfalls bereits am 5. Tag. ohne Malariaimpfung auf den *Steinhof* gebracht, obwohl ihn der Internist für eine „Fieberkur“ freigegeben hatte.[450]

Der zweite, der 19jährige Patient P673 (Hilfsarbeiter) mit der Diagnose „SMV Psychopathie“, der nach einem Diebstahl[451] einen Suizid mit Gas versucht hatte, unzufrieden mit jeder Arbeit, nun arbeitslos war und keine Arbeit suchen wollte, der desinteressiert an seiner Umgebung war, bekam eine Malariafiebertherapie, blieb 37 Tage und wurde nach Hause entlassen. Bei der Anfrage an den Internisten wurde als Diagnose „SMV-Depr.“ angegeben; zuerst war von Kornelius Kryspin-Exner (Handschrift und Unterschrift) ein Befund erbeten worden, ob „gegen Antabus“ zur

[446] Daraus kann geschlossen werden, dass Hoff therapeutische Erwartungen in die Malariafiebertherapie bei Psychopathie hatte. Die Patientin wurde auch nicht als Stammträgerin verwendet (vgl. unten den Exkurs S. 150-160 zur fraglich nicht-therapeutischen Zielsetzung und ihrer Interpretation). Konkrete Hinweise auf Hoffs Vorstellungen zur Wirkweise und zu den Zielen der Therapie fehlen freilich.
[447] Es wurde nicht auf den EEG-Befund gewartet, obwohl das EEG-Labor darum gebeten hatte.
[448] Zweimal mit besonders hohem Fieber: 41° und 41,3°.
[449] Sie sind wie immer nach dem Entlassungsdatum eingeordnet.
[450] Auf der Anfrage an den Internisten wurde als Diagnose nur „SMV“ angegeben. War das vorerst (am 3. Tag) die einzige Diagnose? Oder wurde hier der SMV als hervorragender Grund für die Fiebertherapie angeführt? In der Anamnese heißt es: „Er habe einen grossen Zorn auf die Schwiegermutter [gegen die er auch handgreiflich geworden war] da sie […] ihre Tochter an einen ‚angeseheneren und wohlhabenderen‘ Bräutigam anbringen“ wolle. „Darüber war er so verzweifelt, dass er sterben wollte.“
[451] Dieser wird nur im polizeiärztlichen Befund bei der Einweisung genannt.

Unterstützung der Abstinenz bei Alkoholabhängigen[452] eine Kontraindikation vorliege; das wurde gestrichen und in einer anderen Handschrift auf „Malaria PST?" korrigiert.

Beide Patienten hatten – bezieht man das Alkoholproblem, das im durchgestrichenen Eintrag auf dem Ansuchen an den Internisten um Freigabe erwähnt wird, ein – vergleichbare Diagnosen, aber nur einer der beiden, der Jüngere, bekam die Malariafiebertherapie.
Zwar wurde im April 1964 auch ein 15jähriger Jugendlicher mit ähnlicher Diagnose (SMV, Psychopathie) bereits am zweiten Tag auf den *Steinhof* überstellt, diese rasche Überweisung erfolgte jedoch bei allen Patienten mit der Diagnose Psychopathie, die über 21 Jahre alt waren. Es fällt auch bei der Durchsicht anderer Monate der 1960er Jahre auf, dass die älteren Patienten – sie bildeten durchwegs die Mehrheit – in den ersten Tagen überwiesen wurden.[453] Für diese Entscheidung dürfte das oben aus der Literatur zitierte Argument den Ausschlag gegeben haben, dass eine Therapie nur bei jugendlicher Psychopathie erfolgversprechend sei.[454]

B) EEG als Nachweis von Reifungsstörungen bei der Diagnose Psychopathie

Zur – nach Hoff / Sluga 1962 – dritten Gruppe der PatientInnen mit dem „psychopathischen Syndrom", welches nach der Meinung der Autoren oft im EEG erkennbar „durch eine Störung der Reife des Gehirns bedingt" war, finden sich Beispiele in den Krankenakten mit und ohne Malariafiebertherapie. Hoff und Sluga erwähnen in ihrer Publikation freilich auch hier keine Malaria- oder medikamentöse Therapie, sondern „intensive erzieherische Maßnahmen[455], die durch psychotherapeutische Kurzbetreuung unterstützt" würden, und sie geben diesen PatientInnen eine gute Prognose.[456] Die Malariafiebertherapie bei PatientInnen mit im EEG erkennbaren und als Reifungsstörungen interpretierten Auffälligkeiten wird nicht nur manchmal aus den Krankenakten, sondern auch aus der Erinnerung eines Zeitzeugen ersichtlich. Der Kliniker Heribert Czerwenka-Wenkstetten äußerte 1962 gegenüber Bernd

[452] In der Anamnese und auch sonst wird jedoch nirgends Alkoholismus angesprochen.
[453] Länger aufgenommen wurde 1956 ein 37jähriger Patient mit der Diagnose „Psychopathie" (lt. psychologischem Test: mit „hysteriforme[n] Mechanismen"). Er erhielt bei seinem 28tägigen Aufenthalt „psychotherapeutische Führung und Arbeitstherapie" und an Medikamenten lt. Fieberkurve fast tgl. 1A Bromcalcium i. v. und 3x 2 Tbl. Largactil.
[454] Vgl. oben S. 114, die Zitate 1959 zur „Mental Health Bill" und aus RINGEL/SOLMS/SPIEL, Die Therapie der Psychopathie (1960), S. 460.
[455] Im dort zitierten Fall in einer „Bundeserziehungsanstalt" – d.h. in Kaiserebersdorf.
[456] HOFF/SLUGA, Das psychopathische Syndrom (1962), S. 259 und S. 268. Ausführlich oben S. 111-114.

Lötsch,[457] dass bei den Jugendlichen mit ‚psychopathischen' Verhaltensstörungen die Fieberschübe der Malariafiebertherapie eine Nachreifung von im EEG feststellbaren, nicht ausgereiften cerebralen Systemen stimulieren würden. Elektroenzephalographische Hinweise würden die Unreife im Schläfenlappen vermuten lassen und die Malariakur würde in einigen Fällen eine positive Veränderung zeigen (Auffälligkeit vor der Kur, Verminderung der Auffälligkeit danach).[458] Gegenüber Erinnerungen nach mehreren Jahrzehnten als historische Quelle ist zwar Vorsicht geboten, jedoch auf diesen Bezug von Diagnose und Therapie auf Ergebnisse im EEG kann auch aus einzelnen Hinweisen in den Krankenakten geschlossen werden: ein – meist als leicht bezeichnetes – abnormes EEG wurde kräftig unterstrichen, in seltenen Fällen auch ausdrücklich als Hinweis auf eine Reifungsstörung beschrieben und mit der Malariafiebertherapie in Zusammenhang gebracht.[459] Dazu folgende Fallbeispiele:

> Der 1960 15jährige kaufmännische Lehrling P516 mit der Diagnose „Psychopathie; Pseudoporiomanie" war laut psychologischer Untersuchung „intellektuell gut begabt (IQ. 122). Im Rorschachversuch Hinweise auf stimmungslabile, zykloide jugendliche Persönlichkeit mit psychopathischen Zügen. In sämtlichen Projektionstests […] werden aggressive Tendenzen projiziert […]. Affektiv enthemmt und triebhaft. Infantilismen." In der Zusammenfassung des EEG-Befundes, 10 Tage vor Beginn der Malariafiebertherapie, hieß es: „Leicht abnormes EEG mit temporalen links überwiegenden langsamen regelmässigen Theta-Gruppen, die in H[yper]V[entilation] nebst scharfen Wellen aktiviert werden. N[eben]B[emerkung] Am ehesten im Sinne eines Entwicklungsrückstandes." Der Patient hatte am 14. Tag seines 53tägigen Aufenthalts Malariablut geimpft bekommen, und war nach 7 Fieberzacken und den 7 Chinintagen noch weitere 8 Tage geblieben, an denen er meistens zur Arbeitstherapie ging. Der Patient wurde nach Hause entlassen; für die strenge (Heim)Erziehung, die Hoff / Sluga 1962 für die PatientInnen dieser Gruppe vorsahen, dürften die als sehr streng geschilderten Eltern als ausreichend angesehen worden sein.
>
> Beim 14jährigen Patienten P795, der 1966/67 58 Tage aufgenommen worden war, wurde die Diagnose schon in der Diagnosezeile mit einem Bezug auf einen EEG-Befund ergänzt: „Psychopath[isches] Syndrom (Reifungsstörung im EEG) […]". Nach Jähzorn-Ausbrüchen war er mit Polizeiparere eingeliefert worden, bekam zwei Wochen lang Medikamente zur Beruhigung (Valium, Truxal, Melleril), in der 3. Woche ist

[457] Bernd Lötsch erinnerte sich in einem Diskussionsbeitrag (Krems, 19.6.2014) und in Gesprächen mit Eberhard Gabriel an Äußerung Czerwenka-Wenkstettens ihm gegenüber, als Lötsch 1962 im Rahmen der Produktion des Films über Wagner-Jauregg an der Klinik war.

[458] Im EEG direkt nach der Fieberkur wird nur manchmal eine Veränderung zum „leicht abnormen" Vorbefund festgestellt: vgl. 1964 zum 15jährigen Kellnerlehrling P676 mit der Diagnose „Psychopathie, Alkoholabusus". Er ging in den ersten 5 Wochen fast täglich zur Arbeitstherapie und bekam erst am 38. Tag seines 81tägigen Aufenthaltes (also nach langer Beobachtungszeit) Malariablut geimpft. Im EEG-Befund vor der Malariafiebertherapie hieß es: „Gering abnormes EEG mit unregelmäßigem Alpha-Rhythmus bitemporal niedriger Theta-Tätigkeit ohne HV-Aktivierung [Unterstreichungen im Original]"; nach der Fiebertherapie: „EEG im Rahmen der Norm mit rechts überwiegendem Alpha-Rhythmus bei sonst flacher Kurve. – Gegenüber dem Vorbefund vor der Malariakur ist diesmal die bitemporale Theta-Tätigkeit geringer ausgeprägt, nach Flackerlicht kommt es jedoch zur Aktivierung generalisierter unregelmässiger Theta-Wellen."

[459] Die EEG-Befunde wurden bei der Datenaufnahme nicht systematisch erhoben, sondern nur in einer unsystematischen Nachrecherche, in der die meisten EEG-Befunde ohne derartige Hinweise waren.

> nichts in der Fieberkurve eingetragen; an ihrem Ende wurde ihm malariainfiziertes Blut geimpft. Im Befundbericht an das Bezirksjugendamt noch vor der Entlassung hieß es: „Es handelt sich um eine Psychopathie mit Erregungszuständen. Hinzuweisen ist auf ein abnormes EEG[460], das an eine Reifungsstörung denken lässt. Der Patient wird bei uns einer Fieberkur unterzogen und erhält psychotherapeutische Betreuung[461].“ Da das „psychisch sehr auffälliges Verhalten“ der Mutter als ein wesentlicher „Grund für die Fehlhaltung des Patienten“ angesehen wurde, wurde „dringend eine Absonderung des Minderjährigen aus dem häuslichen Milieu“ empfohlen. Diese Empfehlung wurde befolgt: der Patient wurde der Fürsorge übergeben.

Hier zeigt sich also die zur dritten PatientInnengruppe nach Hoff / Slugar 1962 genannten therapeutischen Vorgehensweisen (Psychotherapie und Änderung des erzieherischen Milieus) nun aber gekoppelt mit der Malariafiebertherapie. Da auch hier klärende Aussagen über die Zielsetzung fehlen, kann nur hypothetisch auf die Absicht geschlossen werden, mit der Malariakur einen Impuls zur ‚Nachreifung’ sowie zur Beruhigung des häufig erregten Jugendlichen, und / oder zur Öffnung des Patienten für eine Psychotherapie zu geben.

Ein ‚normale EEG‘ hinderte jedoch nicht, dass in sonst ähnlichen Fällen eine Malariafiebertherapie gegeben wurde:

> Der 18jährige Maurergehilfe P574 mit der Diagnose „SMV im Rausch, Psychopathie“ bekam 1961/62 bei seinem 33tägigen (einzigen) Aufenthalt eine Malariafiebertherapie. Im Arztbrief bei der Entlassung heißt es: „Psychiatrisch fand sich nach Abklingen des Alk. Rausches ein depressives Zustandsbild und bei einem etwas unterbegabten Pat. mit Reifestörung und Neigung zu Kurzschlussreaktionen[462]. Wegen triebhafter poriomaner Zustände wurde auch eine organische Untersuchung (EEG) angefordert, die jedoch einen normalen Befund ergab. Anschließend wurde eine Malariafieberkur und Neurolepticabehandlung[463] durchgeführt und der Pat. in gebessertem Zustand [nach Hause] entlassen.“[464] Dem Wortlaut nach wurde erwartet, im EEG (bestätigende) Hinweise auf die mehrmals mit Psychopathie verknüpften Diagnose „(Pseudo)Poriomanie“[465] als Reifungsrückstand zu finden.

Das ist nur eines von mehreren Beispielen, aus denen deutlich wird, dass aus den EEG-Befunden kein eindeutiger Hinweis zur Begründung einer Behandlung mit einer

---

[460] Dazu liegen zwei ausführliche EEG-Befunde bei.

[461] In der Fieberkurve ist – wie üblich – weder eine Gruppen- noch eine Einzeltherapie, nur Arbeitstherapie eingetragen.

[462] Zu „Kurzschlusshandlungen“ bei Patienten mit der Diagnose Psychopathie vgl. oben S. 98. (auch Anm. 344); zu ihrer Häufigkeit bei Patienten mit Diagnosen aus dem Bereich der ‚Intelligenzmängel‘ vgl. oben S. 98 Anm. 343.

[463] Neuroleptika wurden üblich in der Fieberkurve eingetragen, in diesem Fall sind jedoch keine eingetragen.

[464] Die Diagnose „Psychopathie“ dürfte darauf zurückzuführen sein, dass er mit 15 Jahren wegen Waffenbesitz und Mopeddiebstahl 22 Monate in einem Jugendgefängnis und dann in Kaiserebersdorf war; die Flucht aus dem Erziehungsheim dürfte den langen Aufenthalt im Erziehungsheim (2 Jahre) und den Zusatz „triebhafte poriomane Zustände“ bewirkt oder bestärkt haben.

[465] Lt. HOFF, Lehrbuch der Psychiatrie (1956), S. 733 gehört die Poriomanie zu den Monomanien, die er im Rahmen der „Vorlesung: Psychopathie II, Perversion, Simulation und Monomanie“ bespricht.

Malariafiebertherapie ersichtlich wird: Auch ‚Psychopathie-Patienten' mit ‚normalem' EEG bekamen manchmal eine Malariafiebertherapie und ein ‚leicht abnormes' EEG in der beschriebenen Weise führte oft zu keiner Malariafiebertherapie. In mehreren Fällen wurde das EEG auch erst Tage nach der Impfung mit malariainfiziertem Blut gemacht,[466] konnte also auf die Indikation keinen Einfluss genommen haben. Wenn auch die oben angeführte Argumentation des Klinikers Heribert Czerwenka-Wenkstetten darauf schließen lässt, dass EEG-Befunde, die als Hinweise auf Reifungsstörungen interpretiert wurden,[467] die Anwendung der Malariafiebertherapie rechtfertigen würden, so dürfte nach den Fallbeispielen dieser Befund, wenn überhaupt, so nur in Kombination mit anderen Symptomen oder Überlegungen eine Rolle gespielt haben. Mit der Meinung, dass bei „Psychopathen" im EEG häufig Hinweise auf Reifungsrückstände zu erkennen seien, konnte man sich freilich auf die internationale Diskussion beziehen, und dazu hatte man den Aussagen von Czerwenka-Wenkstetten zufolge in Wien die ‚Erfahrung' gemacht, dass diese manchmal durch eine Fiebertherapie abgemildert oder verschwinden würden. Rasche Erfolge scheint es – nach dieser unsystematische Nachrecherche – kaum gegeben zu haben. Offenbar wurde auch nicht unbedingt von einer sofortigen Wirkung ausgegangen. Auch der oben nach Hoff / Sluga 1962 zitierte Fall verweist auf ein durch strenge Erziehung (ohne Malariafiebertherapie) nur langsames Verschwinden des psychopathischen Bildes – eine Entwicklung, die schließlich auch im EEG nachvollziehbar zu sein schien.[468]

Hoffs Ausführungen im Lehrbuch von 1956 zeigen, dass diese Unregelmäßigkeit in der Anwendung der Fiebertherapie auch ihre Begründung haben dürfte. Im Abschnitt zur Elektroenzephalographie (für den er persönlich zeichnet) heißt es im Unterabschnitt „Das pathologische EEG der Psychiatrie": „Wenn wir auch bei den meisten psychiatrischen Erkrankungen nur in einem gewissen Prozentsatz abnorme EEGs finden, so liegt doch fast immer ein wesentlich höherer Prozentsatz vor, als es dem normalen Durchschnitt entspräche. Vor allem ist in den meisten Fällen mit pathologischem EEG die Fimilienanamnese nicht völlig o. B., während dies bei den Fällen mit negativem EEG meist zutrifft. Das läßt das Problem der Dysrhythmie wieder in den Vordergrund treten. Trotzdem halte ich es für falsch,

---

466 Bei der oben S. 120f. ausführlich genannten Patientin P194 etwa wurde trotz der Bitte des EEG-Labors nicht auf den EEG-Befund gewartet, sondern 5 Tage vor diesem das mit Malaria-infizierte Blut geimpft.

467 Vgl. zu dieser EEG-Interpretation die Zitate aus BORCK, Hirnströme (2005), S. 18, oben S. 113.

468 Vgl. oben S. 111-113. In den EEG-Befunden von PatientInnen mit leicht abnormem EEG vor der Malariakur wurden nach der Malariakur nur in Einzelfällen Veränderungen vermerkt; in einem Fall, in dem keine Veränderungen zu erkennen waren, wurde der Patient zur Wiederholung eines EEGs nach einigen Monaten aufgefordert.

zu behaupten: Ein abnormes EEG bei einer normalen Versuchsperson ist der Beweis für eine Grundlage, auf der sich Psychopathien, Psychoneurosen, Psychosen oder Epilepsien besonders leicht entwickeln können. – Sicher wirken neben den genetischen Faktoren auch Reifehemmung und frühkindliches Trauma mit.“[469]

Hoff bringt zwar zur Veränderung im EEG aufgrund einer (kombinierten) Malariakur im Lehrbuch von 1956 nur ein Beispiel zur Neurolues, setzt dieses jedoch in den allgemeineren Kontext mit „verschiedenen Formen diffuser organischer Erkrankungen, die mit Störungen des Bewußtseins einhergehen“: Reihenunersuchungen von Behandlungen der Neurolues mit Malariakur und Penicillin hätten gezeigt, dass in der Therapie „zu einer deutlichen Besserung der abnormen EEG-Zeichen“ komme, „die oft den Grad der klinischen Besserung bei weitem“ übertreffe.[470] Das war ein Argument für die Therapie-Kombination, da nach Wiener Meinung die Malariakur den klinischen Befund bei PatientInnen mit progresiver Paralyse bessert. Im Bezug auf die Verbesserungen im EEG könnte es jedoch auch ein – in den vorliegenden Quellen allerdings nicht ausgeführtes – Argument für die Hypothese von der Malariakur als ‚Erschütterungstherapie‘ bei bestimmten Symptomen nicht-luetischer psychischer Erkrankungen sein.

C) Das psychopathische Syndrom nach Encephalitiden

In der Datenbank finden sich 14 PatientInnen (6 weibliche und 8 männliche), in deren Krankenakt, meistens in der Diagnosezeile, ein Hinweis auf Encephalitis eingetragen war. In 6 Fällen ist dieser Eintrag mit Psychopathie kombiniert. In dieser Kombination gehören sie nach dem oben genannten Artikel von Hoff und Sluga (1962) zur vierten Gruppe der PatientInnen mit „psychopathischem Syndrom“.[471] Keine der drei weiblichen und keiner der drei männlichen Patienten bekam eine Malariafiebertherapie. Sie bekamen bei ihren mehr oder weniger langen Aufenthalten sedative Medikamente.

Während die sechs PatientInnen mit der Kombination von Encephalitis und Psychopathie – zur Beruhigung[472] – medikamentös behandelt wurden, bekamen drei der acht PatientInnen mit

---

[469] HOFF, Lehrbuch der Psychiatrie (1956), S. 222 (Vorlesung: Elektroencephalographie). Hoff nennt hier Hinweise in der Familienanamnese mit möglicher Wirkung auf eine krankhafte Entwicklung.
[470] HOFF, Lehrbuch der Psychiatrie (1956), S. 233 (Vorlesung: Elektroenzephalographie, Unterabschnitt „Störung des Bewußtseins“).
[471] Vgl. oben S. 112.
[472] Auch das Neuroleptikum wurde nur kurzfristig zur Beruhigung gegeben.

anderen Diagnosekombinationen zur Encephalitis eine Malariafiebertherapie: die 13jährige Patientin S902 1953 mit der Diagnose „schizophrene Reaktion bei antriebsgestörter postenzephalitischer Persönlichkeit", der 21jährige Patient X833 1967 mit der Diagnose „Status post Encephalitis, Epi[lepsie]", und der 16jährige Patient X687 1964 mit der Diagnose „Postencephalitisches Zustandsbild, Kleinwuchs, Reifungsstörung, Erregungszustände".[473]

Nicht eindeutig auf Psychopathie bezogen nennt der Grazer Psychiater Erich F. Pakesch 1951 die Malariafiebertherapie unter mehreren Fieberkuren sowohl bei chronischer Encephalitis, als auch bei chronischer Meningitis und schreibt: „Auch die Malariakur hat sich (nach den Erfahrungen unserer Klinik) bei diesen frühen parkinsonistischen Symptomen [den „geringfügigen Zeichen eines beginnenden postencephalitischen Zustandes wie leichter Tremor, Hypomimie und vegetative Störungen"] gut bewährt."[474] In der Datenbank sind bei drei Patientinnen und acht Patienten mit nicht-luetischen Diagnosen Hinweise auf eine Meningitis eingetragenen, in zwei Fällen wurde eine Malariafiebertherapie gegeben: 1960 beim 14jährige Patient X619, der bei diesem ersten Aufenthalt mit der Diagnose „Erregungszustände, neurotische Verwahrlosung, Zust[and] nach Meningitis" eine Malariafiebertherapie bekam. 1950 beim 19jährigen Patienten I408 bei seinem dritten von sieben Aufenthalten mit der Diagnose „Zustand nach frühkindlicher Meningitis, Debilität, Athetose double" (im Patientenakt sind noch weitere Hinweise auf Unruhe-Erregungszustände[475]). Beide Patienten hatten bei kurz nachfolgenden Aufnahmen auch die Diagnose Psychopathie in Kombination und bei beiden dürften „Erregungszustände" zur syptombezogenen Behandlung mit der Fieberkur geführt haben – beim Patienten von 1950 möglicherwies die athetotische Bewegungsstörungen[476].

Zusammenfassend lässt sich festhalten, dass in der ‚Ära Hoff' junge und fast nur junge Männer mit einer Psychopathiediagnose mit einer Malariafiebertherapie behandelt wurden. Es konnte keine Erklärung dafür gefunden werden, warum jugendliche Patientinnen mit der

---

[473] Vgl. oben S. 95 bzw. unten S. 219 und oben S. 85.
[474] PAKESCH, Spezielle Therapie der Nervenkrankheiten (1951), S. 162. Hoff beschrieb bereits 1923 die Anwendung der Malariafiebertherapie in der Behandlung von „chronischen Folgezuständen der Enzephalitis […], darunter war auch eine Zahl von Fällen mit Parkinsonismus. Die Besserungen waren aber keine sehr weitgehenden": HOFF, Uebersicht der therapeutischen Versuche bei der Encephalitis lethargica auf der Klinik vom 1. Jänner 1916 bis 30. Mai 1923 (1923), S. 901.
[475] „extrapyramidale Störungen im Sinne von Unruhebewegungen (Athetose double)"; in der Anfrage an den Internisten um Freigabe zur Malariatherapie: „Erregungszust[ände] bei Debilität".
[476] Vgl. das SPIEL/SPIEL-Zitat oben S. 92f. zur Malariafiebertherapie bei „Kombinationen des Schwachsinns mit motorischen neurologischen Erscheinungen".

gleichen Diagnose und den gleichen Symptomen nur in Einzelfällen eine Malariafiebertherapie erhielten.

D) Exkurs zu PatientInnen mit der Diagnose Psychopathie aus Erziehungsheimen

Die ‚Klinik Hoff' arbeitete eng mit den Wiener Fürsorgeämtern, dem schulpsychologischen Beratungsdienst[477] sowie mit der Administration der Kinder- und Jugendheime zusammen. Psychohygienische und psychotherapeutische Ansätze standen in der Begutachtung, Beratung und Therapie im Vordergrund. Diese Zusammenarbeit war zentral in der psychiatrische-neurologischen Kinderstation organisiert, die 1951 mit Walter Spiel als Leiter eingerichtet worden war.[478] Aber auch in den Erwachsenenstationen spielte die Klinik eine Rolle in der psychiatrischen Begutachtung und Behandlung unmündiger Jugendlichen im Alter von 13 und 14 bis 21 Jahren.

Im Fokus der folgenden Ausführungen stehen sowohl Patienten aus der Bundesanstalt für erziehungsbedürftige männliche Jugendliche in Kaiserebersdorf, die dem Justizministerium unterstand und schon mehrmals in den Zitaten aus den Wiener Publikationen zur Psychopathie angesprochen wurde, sowie aus der Erziehungsanstalt der Stadt Wien in Eggenburg – ebenfalls für männliche Jugendliche. Besprochen werden auch die nur zwei weiblichen Jugendlichen in der Datenbank, die mit der Diagnose Psychopathie eine Malariatherapie bekamen und aus dem Erziehungsheim Wiener Neudorf (das ebenfalls dem Justizministerium unterstand) bzw. aus den Lehrmädchenheimen der Stadt Wien in der Juchgasse und in der Rochusgasse kamen. Sie werden mit den anderen Patientinnen mit der Diagnose Psychopathie des selben Jahres (1951) verglichen. In die Erziehungsanstalten in Kaiserebersdorf und Wiener Neudorf kamen Jugendliche üblich nach einer Haftstrafe und in jene in Eggenburg und Wien-Rochusgasse nach Erziehungsschwierigkeiten in offenen Heimen und manchmal auch nach kleineren Straftaten. Nach den zeitgenössischen Publikationen entspricht ihnen und ihrem Alter die häufige Diagnose „Psychopathie" und auch in Zusammenhang damit die Diagnose „neurotische Verwahrlosung"[479].

Im Folgenden soll der Frage nachgegangen werden, ob bei diesen PatientInnengruppen ein System in der Indikation erkennbar wird, d.h. deutlich wird, welche Symptome bzw. Symptomgruppen hier und in welcher Regelmäßigkeit zur Anwendung der Malariafiebertherapie führten.

---

[477] N. N., 10-Jahresbericht der Psychiatrisch-Neurologischen Kinderstation (1962), S. 220.
[478] Vgl. oben S. 25 Anm. 65.
[479] Vgl. HOFF/SLUGA, Das psychopathische Syndrom (1962), S. 248: Der Beginn „psychopathische[r] Zustandsbilder" könne sich „als neurotische Entwicklung" manifestieren. Die Autoren beziehen sich auf BERNER/SOLMS, Die Entwicklung einer Neurose (1954), S. 242-252 – auf einen Artikel, in dem eine Patientin beschrieben wird, bei der die „Entwicklung von einer Neurose zum Bild der Psychopathie" (ebd., S. 252) zu beobachten war.

a) Weibliche Jugendliche aus Heimen mit der Diagnose Psychopathie

Weibliche Jugendliche mit Diagnosen außerhalb der Neurolues wurden in der Klinik Hoff sehr selten mit Malariafiebertherapie behandelt. Nach der Datenbank waren es insgesamt 13 Patientinnen. Davon hatten nur 3 die Diagnose Psychopathie, wovon zwei 1951[480] aus der Bundesanstalt für erziehungsbedürftige weibliche Jugendliche in Wiener Neudorf[481] bzw. aus dem Heim der Stadt Wien in der Rochusgasse[482] kamen. Nach Schwierigkeiten im familiären und beruflichen Umfeld waren sie dort untergebracht worden.

> Die 17jährige Patientin P892 mit der Diagnose „jugendl. Psychopathie" hatte sich nach ihrer Aussage nach einer Auseinandersetzung mit der Mutter im letzten Monat „freiwillig in das Lehrlingsheim der Stadt Wien, Wien, III., Juchgasse" begeben,[483] von wo sie „auf die hiesige Klinik überstellt" wurde. Nach der Beschreibung ihrer Mutter war sie „seit Schulbeginn „schwer erziehbar, im Benehmen dauernd anstössig, verlogen, stahl verschiedentlich kleine Dinge, auch Geldbeträge."[484] Vor 2 Jahren hatte sie eine Berufsschule abgebrochen und war seither an zwei Arbeitsstellen als Bürokraft tätig gewesen. Die Patientin, die vom Lehrlingsheim zur „Beobachtung" eingewiesen worden war, bekam bei ihrem einzigen, 29tägigen Aufenthalt eine Malariafiebertherapie mit 2 Fieberschüben.[485]
>
> Die 17jährig Patientin P893 wurde 1951 4mal aufgenommen und bekam bei ihrem zweiten Aufenthalt mit der Diagnose: „Psychopathie, Verwahrlosung" eine Malariafiebertherapie. Sie stammte aus schwierigen Verhältnissen mit neun älteren Geschwistern, war in mehreren Heimen gewesen und war wiederholt von zu Hause und vor dieser zweiten Aufnahme aus der Erziehungsanstalt Wiener Neudorf[486] weggelaufen. Nun sei sie „fest entschlossen, in kein Heim mehr zu gehen, sie wolle sich lieber umbringen. Sie werde, sofern man sie doch in ein Heim schicke, entweder

[480] Sie waren beide auch ‚Stammträgerinnen'. Die dritte Patientin mit der Diagnose Psychopathie, die eine Malariafiebertherapie bekam, wurde erst 1963 behandelt (vgl. oben S. 120f.). Sie kam von zu Hause über die Privatordination von Hoff und war nie in einem Heim und auch nicht Stammträgerin.

[481] Lt. MAYRHOFER, Besuch in der Bundesanstalt für Erziehungsbedürftige in Wiener-Neudorf (1958/59), S. 73 wurde – wie vor dem Krieg – das Heim ab 1951 wieder durch die Schwestern der Kongregation vom Guten Hirten betreut (bis 1955 parallel zum Heim in Maria Theresienfeld, vgl. unten S. 131 Anm. 490).

[482] „1946 wurde ein Lehrmädchenheim in 3, Rochusgasse (später Durchzugsheim für Mädchen mit Sondergruppe für milieugeschädigte Mädchen) eröffnet." (https://www.geschichtewiki.wien.gv.at/Lehrlingsheime, 24.11.2018).

[483] Auf dem Deckblatt ist als letzte Adresse das Heim „3, Rochusgasse 8" eingetragen.

[484] Lt. Rorschachtest „intellektuell unterbegabt"; „Zusammenfassung: […] das Bild einer neurotisch verwahrlosten Persönlichkeit".

[485] Aus den Unterlagen ist kein Grund für den Abbruch nach nur zwei Fieberschüben ersichtlich.

[486] Die Patientin lt. Anamnese: „Nun sei sie in einem Erziehungsheim, wo Klosterschwestern seien, sie arbeite im Garten, die Arbeit würde ihr ganz gut gefallen, das Essen sei aber schrecklich schlecht, ausserdem würfen die Schwestern immer den Zöglingen vor, dass sie vor dem Eintritt in das Heim einen schlechten Lebenswandel geführt hätten und das vertrage sie nicht. Sie habe immer Streit mit den Schwestern". In der Datenbank ist auch eine weitere Patientin eingetragen – P11360 mit den Diagnosen „Psychopathie, Selbstbeschädigung" –, die zur ersten Aufnahme an der Klinik (von 3 innerhalb von 18 Monaten) im Jänner 1953 aus der Erziehungsanstalt Wiener Neudorf kam, aber keine Malariafiebertherapie bekam; vgl. zu ihrer Behandlung in Wien 1953/54 mit Insulin[sub?]koma- und Elektrokramp- bzw. zuletzt mit einer 8-tägigen intensiven Largactiltherapie alleine, unten S. 277 Anm. 1040.

> sofort durchgehen, oder wenn ihr dies nicht gelinge, S[elbst]M[ord] machen." Zu ihrer Beurteilung hieß es: „Auf der Station benimmt Pat. sich ruhig und unauffällig, hilft mit, ist ruhig." Sie beendete ihren 60tägigen Aufenthalt an der Klinik, indem sie davonlief und wurde im selben Jahr noch 2mal aus dem Heim in der Rochusgasse zu einem 45- bzw. 6tägigen Aufenthalt an die Klinik gebracht.

Die Diagnose „Psychopathie" (mehrmals in der Diagnosezeile oder im Patientinnenakt mit „neurotischer Verwahrlosung" verbunden), wurde häufig weiblichen Minderjährigen gegeben, die unter dem Verdacht der Prostitution[487] von der Polizei aufgegriffen, ins Heim in der Rochusgasse im 3. Wiener Bezirk und von dort an die Klinik gebracht wurden. Die Prostitution minderjähriger Psychopathinnen war in den zitierten Publikationen von Hoff und seinen Mitarbeitern ein Thema, bei dem man sich meistens auf die einschlägigen Arbeiten August Aichhorns aus den 1920er und 1930er Jahren bezog.[488]

Im Jahr 1951 sind in der Datenbank 12 weitere Patientinnen mit der Diagnose Psychopathie verzeichnet, die keine Malariafiebertherapie bekamen. Nur 2 dieser Patientinnen waren jugendlich (beide nun erst 15 Jahre alt); die anderen 10 Patientinnen waren 26 bis 51 Jahre alt – kamen also wohl schon deshalb nicht für eine Therapie der Psychopathie in Frage. Eine der beiden kam aus einem Heim und ihre Beschreibung gleicht jener der beiden Patientinnen mit Malariafiebertherapie im selben Jahr:

> Die 15jährige Patientin P9841 mit der Diagnose „jugendliche Psychopathie" war von der Polizei in der Nacht aufgegriffen und ins Heim in der Rochusgasse gebracht worden. Sie kam nun an die Klinik, nachdem sie seit 4 Tagen aus Protest nichts gegessen und mit Suizid gedroht hatte. Das psychologische Gutachten sprach von einer „neurotisch verwahrloste[n] jugendliche[n] Persönlichkeit" mit „gegen sich selbst gewandte[r] Gewalttätigkeit, Masochismus". Gegen ihren Willen[489] wurde sie nach 9 Tagen ohne körperliche Therapie zu den Klosterschwestern „ins Heim Maria Theresienfeld gebracht".[490]

---

[487] In HOFF, Lehrbuch der Psychiatrie (1956), S. 703 (Vorlesung: Psychopathie I) heißt es: es handle sich „bei der Mehrzahl der sogenannten Soldatenbräute um psychopathische Persönlichkeiten".

[488] Vgl. auch HOFF/RINGEL, Die sogenannte Soldatenbraut (1952), S. 154, wo die Autoren gegen den Versuch, „sie durch Bestrafung zu erziehen oder sie durch Veränderung des Milieus zu bessern", „die tiefenpsychologische Behandlung der Einzel-Person und der Gruppe" als „das geeignete Mittel" nennen, „jene Mädchen wieder ins Leben zurückzuführen, sie an die Realität anzupassen, so daß aus ihnen und ihren Kindern nützliche Menschen werden." Sie berufen sich auch hier immer wieder auf Aichhorn.

[489] Sie „wollte lieber am Steinhof oder durchbrennen oder sich umbringen […] möchte keine Klosterschwestern, möchte in Lehrlingsheim."

[490] Ab 1912 gehört das Gebäude der „Kongregation der Schwestern vom Guten Hirten", womit das Gebäude zu einem Kloster umgebaut und aufgestockt wurde. 1955 gaben die Schwestern das Gebäude auf, es wurde in der Folge als Landesberufsschule genutzt; https://de.wikipedia.org/wiki/Landesberufsschule_Theresienfeld (15.06.2019).

b) Jugendliche aus der Bundesanstalt für erziehungsbedürftige männliche Jugendliche in Kaiserebersdorf

Die „Klinik Hoff" hatte in der dem Justizministerium unterstellten „Bundesanstalt für erziehungsbedürftige männliche Jugendliche" in Kaiserebersdorf in der Neuordnung nach der „Revolte" der Zöglinge von 1952[491] die psychologisch-psychiatrische Betreuung übernommen.[492] Ebenso in der „Bundesanstalt für erziehungsbedürftige weibliche Jugendliche" in Wiener Neudorf, die soeben im Fallbeispiel zu Patientinnen mit der Diagnose Psychopathie aus dem Jahre 1951 genannt wurde. In beiden Bundesanstalten wurden Jugendliche aufgrund eines richterlichen Entscheides nach einer Haftstrafe eingewiesen.

Zur Arbeit der Kliniker in der Erziehungsanstalt Kaiserebersdorf heißt es 1967 in der Festschrift für Hoff zum 70. Geburtstag, dass das Forschungsinteresse der Klinik der Frage gelte, „wie aus dem durch die Methodik der Tiefenpsychologie erhellten familiären Gefüge, der Eltern-Kind-Beziehung, Werden und Verhalten der menschlichen Persönlichkeit in Gesundheit und Krankheit verständlich" würde. „So wurde die Bundeserziehungsanstalt Kaiserebersdorf zum notwendigen Forschungsfeld für Hoff ebenso wie er und seine Mitarbeiter zur notwendigen Ergänzung des Erziehungs- und Behandlungsstabes der jugendlichen Kriminellen."[493] Die Zusammenarbeit funktionierte über „Konsiliarii", die von der Klinik entsandt wurden.[494]

---

[491] Vgl. HASELBACHER, Die „Revolte" in der Bundesanstalt für Erziehungsbedürftige Kaiser-Ebersdorf im Jahre 1952 (1991), passim.

[492] Zur Zusammenarbeit der Kliniker mit den ErzieherInnen der beiden Bundesanstalten, jener für männliche Jugendliche in Kaiserebersdorf und jener für weibliche Jugendliche in Wiener Neudorf, vgl. RINGEL/SOLMS/SPIEL, Die Therapie der Psychopathie (1960), S. 460 – zitiert oben S. 115f. Vgl. auch SLUGA, Jugendpsychiatrische Tätigkeit bei Verwahrlosten (1962), S. 211. In der „Bundesanstalt für erziehungsbedürftige Mädchen" in Wiener Neudorf stand „dauernd ein Psychiater der Klinik Hoff, Assistent Dr. Ring[e]l, zur Verfügung": MAYRHOFER, Besuch in der Bundesanstalt für Erziehungsbedürftige in Wiener-Neudorf (1958/59), S. 73. Zur Leitung (wie vor dem Krieg) durch die Schwestern der Kongregation vom Orden zum Guten Hirten vgl. oben S. 129 Anm. 481.

[493] ARNOLD/TSCHABITSCHER, Die Wiener Neurologisch-Psychiatrische Schule unter H. Hoff (1967), S. 1129.

[494] N. N., 10-Jahresbericht der Psychiatrisch-Neurologischen Kinderstation (1962), S. 220. Walter Spiel erhielt nach der 1994 erschienenen „Dokumentation" zur Geschichte der Kinder und Jugendpsychiatrie an der Wiener Universitätsklinik am 16.4.1953 vertraglich „den Auftrag, Konsiliararzt und Berater des Bundesministeriums für Justiz an den Strafanstalten für Jugendliche zu werden." SPIEL/MUTSCHLECHNER/SCHAUFLER/STÜTZ, Die Entstehung des Fachgebietes Kinder- und Jugendneuropsychiatrie (1994), S. 21f. Erwin Ringel übernahm die Bundesanstalt in Wiener Neudorf. 1961 übergab Spiel die Betreuung an Willibald Sluga und Rudolf Mader.

Walter Spiel, dem Leiter der Kinderstation an der ‚Klinik Hoff', kam in Kaiserebersdorf eine zentrale Rolle zu.[495] Bereits 1956 publizierte er gemeinsam mit dem Psychologen Manfred Haider über Erfahrungen auf Basis „einer großen Zahl von psychiatrisch und psychologisch, durch Tests und Aussprachen, genau erfaßten jugendlichen Kriminellen".[496] Sie besprachen die Probleme der Gefängnisstrafen und traten für „beurteilen und erziehen" anstatt „verurteilen und bestrafen" ein.[497] Nach einem Hinweis auf die Notwendigkeit von prophylaktischen „sozial-hygienische[n] Maßnahmen, welche die Familienstabilität fördern" (Erziehungshilfe, Schul- und Erziehungsberatungsstellen), traten sie für eine Aufwertung und Entlastung der Erzieher im Heim ein, die „als Grundlage einer Nacherziehung" die persönliche Eignung haben müssten, „Beziehungen zu Jugendlichen" aufzubauen. Die Bedeutung der Gruppe für die Jugendlichen mit der Gefahr der „Plattenbildung" (Bandenbildung) und der positiven Möglichkeit des Erziehers in die Gruppendynamik einzugreifen wurde erwähnt, ebenso die negative Wirkung der „Doppelbestrafung" durch Gefängnis und Einweisung in eine Erziehungsanstalt, wobei möglichst nur letzteres in einer reformierten (unter anderem entbürokratisierten, die auszubildenden Erzieher entlastenden) Form vom Richter gewählt werden sollte. Die Jugendrechtspflege werde „den Weg von den rein statischen Prinzipien der Verurteilung und des Bestrafens zu den dynamischen Prinzipien der Beurteilung, Differenzierung, Nacherziehung und Heilung gehen müssen."[498]

Die beiden Autoren unterschieden sechs „verschieden strukturierte Persönlichkeiten, beurteilt vor allem nach der Ausbildung ihrer Gewissensinstanzen: relativ normales, soziales Über-Ich; präsoziales Über-Ich; sozial labiles Über-Ich; dissoziales Über-Ich; antisoziales Über-Ich; asoziales Über-Ich." Der „Aufbau einer einigermaßen entsprechenden Gewissensinstanz" sei

---

[495] Auf der Kinderstation wurden Kinder bis ca. 14 Jahren behandelt, während in den Bundeserziehungsheimen Kaiserebersdorf und Wiener Neudorf Jugendliche untergebracht waren. Die Ausrichtung von Walter Spiel war bereits in den 1950er Jahren Kinder- und Jugendpsychiatrie.

[496] HAIDER/SPIEL, Einige psychologische und psychiatrische Betrachtungen zum Jugendstrafrecht und Jugendstrafvollzug (1956), S. 146.

[497] HAIDER/SPIEL, Einige psychologische und psychiatrische Betrachtungen zum Jugendstrafrecht und Jugendstrafvollzug (1956), S. 152.

[498] HAIDER/SPIEL, Einige psychologische und psychiatrische Betrachtungen zum Jugendstrafrecht und Jugendstrafvollzug (1956), S. 146 und S. 153. Erwähnenswert die Position der in Budapest geboren und ab 1932 in London lebenden Psychoanalytikerin Melitta Schmideberg (1904-1983), der einzigen Tochter der österreichisch-britischen Psychoanalytikerin Melanie Klein (1882-1960). Sie sprach sich für psychotherapeutische Behandlungen und gegen Gefängnisstrafen aus, da die Patienten am effektivsten behandelt werden könnten, wenn sie Teil der Gesellschaft seien und die Beziehungen, die sich in der Arbeit und im Sexualleben ergeben, in der Therapie analysiert würden. Bewährungsstrafen seien daher von unschätzbarem Wert, da diese die eigentliche Strafe nur aufschieben und dadurch wie eine Drohung über den Patienten schweben würden, die sie unter Strafe zur Therapie „zwinge". Ihr Vortrag wird zitiert in „Annotations" zu THOMAS/CLAY, Treatment of the Psychopath (1958), S. 249.

bei allen diesen Persönlichkeiten „nachzuholen, beziehungsweise zu fördern“, mit Ausnahme bei jenen der ersten Gruppe, deren soziale Einordnung mit Belehrung und Erziehungsmaßnahmen leicht herzustellen sei, und jenen der letzten Gruppe, die eine „völlige Unfähigkeit zur Gewissensbildung, hauptsächlich auf Grund von Anlagefaktoren, beziehungsweise schweren hirnorganischen Schädigungen“ hätten. Besonders wichtig sei eine psychotherapeutische Betreuung der vierten Gruppe der „neurotisch Verwahrlosten“, da Erziehungsmaßnahmen alleine „oft eine ‚paradoxe' Wirkung ausüben, indem das […] bestehende ‚Strafbedürfnis'“ – aufgrund „starke[r], oft unbewußte[r] Schuldgefühle“ – „dadurch abgesättigt“ würde. Die fünfte Gruppe mit „antisozialem Überich“, mit einer „pathologisch gebildete[n] Gewissensinstanz, bei welcher auf Grund von Identifizierung mit selbst verbrecherischen Eltern eine gegen die Gemeinschaft gerichtete Einstellung sozusagen zur sozialen Forderung erhoben wurde“, müsse man auch „einen verstärkten ‚Erziehungsdruck' anwenden […], um den Betreffenden die Forderungen und Wertmaßstäbe der menschlichen Gemeinschaft immer aktuell und real vor Augen zu führen.“

Im Publikationsorgan des Vereins der Ärzte in zeil Wichtigkeit, für das therapeutische Vorgehen die Diagnose Psychopathie individuell zu differenzieren, um „aus dem breiten Spektrum, den dieser Begriff in direkter Beschäftigung mit den Anstaltsinsassen bietet, jeweils ein bestimmtes Segment zu erfassen“.[499] Walter Spiel berichtet über Beobachtungen an „500 jugendliche[n] Kriminelle[n] der Erziehungsanstalt“ „im Hinblick auf Konstitutionstypen […] auf Reifungsprobleme […] auf die cerebralen Schädigungen […] als ‚Ursache‘ einer kriminellen Entwicklung […], [auf] die Beziehung der Intelligenz zur Kriminalität“ und ihre statistische Auswertung. [500] Als Grundlage für eine „Umgestaltung der Erziehungstendenz und des Erziehungsmilieus“ hätten sie „diagnostisch ein kompliziertes System einer Persönlichkeitsbeurteilung aufgebaut“, um dem Erzieher zu ermöglichen „das einmalige in jeder Persönlichkeit aufzufinden“ und „die Beeinflussung des Zöglings so vielgestaltig als nur möglich zu machen“.[501] – Aus dieser Untersuchung entwickelte Richtlinien werden jedoch nicht genannt.

Die in diesen Publikationen aus der Arbeit in Kaiserebersdorf angesprochenen Differenzierungen „aus dem breiten Spektrum“ der Diagnose Psychopathie sind nicht

---

[499] HAIDER/SLUGA, Probleme der psychotherapeutischen Betreuung in der Erziehungsanstalt (1956/57), S. 20f.
[500] SPIEL, Über jugendpsychiatrische Erfahrungen im Jugendstrafvollzug (1956), S. 29-32.
[501] Ebd., S. 36f.

vertiefend ausgeführt und auch aus den Angaben in den PatientInneakten nicht zu ersehen. Deshalb ist auch nicht zu klären, welche spezifischen Persönlichkeitsmerkmale der PatientInnen und welche Symptome der Erkrankung zur unterschiedlichen Behandlung mit oder ohne Malariakur führten. Deutlich wird jedoch aus den genannten Publikationen die Bedeutung der „Persönlichkeitsbeurteilung“ und der Symptome und Symptomkombinationen im Syndrom Psychopathie für die Indikation. Es sind Symptome, die auch bei PatientInnen mit anderen nicht-luetischen Diagnosen und Malariatherapie genannt wurden – bei Diagnosen, bei denen die Indikation in der medizinischen Literatur der Zeit erwähnt, wenn auch nicht diskutiert wurde. Daraus dürfte zu schließen sein, dass die Malariakur als ‚Erschütterungstherapie‘ allgemein aufgrund von gleicher Symptomatik und ihrer Ausprägung gegeben wurde.

In der Datenbank sind 25 männliche Jugendliche verzeichnet, die aus der Erziehungsanstalt Kaiserebersdorf an die Klinik oder von der Klinik nach Kaiserebersdorf kamen. Vier davon bekamen eine Malariafiebertherapie. Diese vier Malariatherapiepatienten hatten unterschiedliche Diagnosen: 1955 der 16jährige Patient P359 und 1956 der 18jährige Patient P394, hatten die Diagnose „Psychopathie“. Der Patient X312 hatte bei seiner dritten Aufnahme 1953, bei der er als 19jähriger eine Malariafiebertherapie bekam, die Diagnose „hysterische Anfälle“, bei den beiden vorhergehenden Aufnahmen jedoch diese Diagnose in Kombination mit Psychopathie. Der vierte Patient X691 hatte bei seinem Aufenthalt 1961 mit der Fieberkur als 17jähriger die als Vorstufe zur Psychopathie erwähnte Diagnose „neurotische Verwahrlosung“, bei seiner zweiten und letzten Aufnahme 1964 die Diagnose „SMV, Haftreaktion, Psychopathie“.

Von den 21 Jugendlichen, die aus der Erziehungsanstalt Kaiserebersdorf kamen und keine Malariakur bekamen[502], hatten 16 die Diagnose Psychopathie, 12 davon in Kombination mit

[502] Der 1951 16jährige Patient P7516 wird hier zugezählt: er kam mit der Diagnose „jugendliche Psychopathie“ aus dem Heim der Wiener Fürsorge in Eggenburg und wurde nach seinem 70tägigen Klinikaufenthalt in die „Bundesanstalt für Erziehungsbedürftige in Kaiserebersdorf“ überwiesen, da sich bei ihm – lt. Bescheid des Jugendamtes der Stadt Wien – „die Erziehungsversuche im Rahmen der Fürsorgeerziehung als aussichtslos erwiesen“ hätten.

einem Selbstmordversuch,[503] fünf hatten keine Psychopathie-Diagnose.[504] Elf der 21 Jugendlichen hatten einen Aufenthalt von fünf Tagen oder mehr. Von den zehn Jugendlichen, die weniger als fünf Tage an der Klinik waren und nur wegen ihrer Herkunft aus dem Erziehungsheim in die Datenbank aufgenommen wurden, hatten alle eine Psychopathie-Diagnose, davon neun mit dem zusätzlichen Eintrag Selbstmordversuch; alle wurden innerhalb der ersten vier Tage an die *Heil- und Pflegeanstalt Steinhof* überstellt – wie auch die Mehrzahl der anderen Patienten nach ihrem Aufenthalt an der Klinik: die Kliniker dürften eine psychiatrische Weiterbehandlung oder Beobachtung vor einer Rücküberstellung nach Kaiserebersdorf für notwendig erachtet haben.

Während die verzeichneten Aufnahmen ohne Malariafiebertherapie über den ganzen Untersuchungszeitraum verteilt waren, fallen die Aufenthalte der vier Patienten mit Malariafiebertherapie, obwohl mit dieser Therapie alle Patienten unabhängig von der Diagnose aufgenommen wurden, nur in die Jahre bis 1961 (1953, 1955, 1956 bzw. 1961). Dazu ist auf die allgemeine Quellenproblematik hinzuweisen, dass ein Teil dieser Akten erst nach 1969 mit einem späteren Aufenthalt archiviert ist und deshalb in der Datenbank fehlt, und Akten wohl auch verloren gingen.[505] Der Kliniker Bernd Küfferle erinnert sich jedenfalls an Malariafiebertherapien nach 1965 bei Patienten aus Kaiserebersdorf.[506] Aussagewert für einen Vergleich besitzt demnach nur, dass aus Kaiserebersdorf im gleichen Zeitabschnitt, d.h. bis Ende 1961 zwölf Patienten und davon sieben mit der Diagnose Psychopathie, keine Malariafiebertherapie bekamen. Aus der Diagnose ist kein System in der Anwendung der Malariafiebertherapie bei den „erziehungsbedürftigen" Zöglingen der Bundesanstalt zu erkennen. Ähnlich ist es mit den in den Krankenakten genannten Symptome oder Symptomkombinationen, denn auch sie führen nur in einer Minderheit der Fälle zu ihrer Anwendung.

---

[503] Drei von ihnen (P6519 18jährig; P6520 16jährig; P6521 17jährig) hatten im April 1964 ihr gemeinsames Zimmer angezündet, um aus Kaiserebersdorf weg zu kommen. Sie wurden mit der Diagnose „SMV, Psychopathie (Rauchgasvergiftung)" am 2. Tag auf den *Steinhof* überstellt.

[504] Sie hatten unterschiedliche Einträge in der Diagnosezeile: „Hebephrenie", keinen Eintrag, „Debilität SMV", „Verwahrlosung, Schwachsinn, Epilepsie (derzeit[?] keine Anfälle)" bzw. „Fakultätsgutachten: Eifersuchts- und Sexualdelikte".

[505] Vgl. auch oben S. 15f.

[506] Bernd Küfferle arbeitete ab Sommer 1965 an der Psychiatrie (Interview mit Gernot Heiss am 6. Mai 2013).

Üblich wurden die Zöglinge nach ihrer ärztlichen Beobachtung und Behandlung wieder zurück nach Kaiserebersdorf gebracht.[507] So auch die Zöglinge, die nach Suizidversuch oder Suizid-Androhungen aufgenommen wurden, und die als Motiv angaben, sich verletzt zu haben, um aus dem verhassten Heim wegzukommen:[508]

> Der Patient mit einer Malariafiebertherapie aus Kaiserebersdorf X691, der als 17jähriger 1961 mit der Diagnose „Demonstrativer SMV, neurotische Verwahrlosung" an die Klinik aufgenommen wurde, nachdem er „sich geschnitten [hatte] um endlich [nach 13 Monaten] aus der Erziehungsanstalt Kaiser Ebersdorf herauszukommen", wurde nach dem Klinikaufenthalt zurückgebracht. Ebenso sollte der 18jährige Hilfsarbeiter P394, der 1956 mit der Diagnose „Psychopathie, wiederholte SMV" aus Kaiserebersdorf an die Klinik aufgenommen wurde und eine Malariafiebertherapie bekam, wieder ins Erziehungsheim zurückgebracht werden. Er hatte schon viermal mit Suizid gedroht bzw. diesen versucht und er war bereits dreimal aus dem verhassten Heim geflohen. Als man ihn wieder nach Kaiserebersdorf überstellen wollte, sprang er in eine Glasscheibe und wollte sich mit dem Glas am Hals verletzen; daraufhin brachte man ihn auf den *Steinhof*. Ob er von dort wieder ins Erziehungsheim gebracht wurde, ist zwar aus dem Patientenakt der Klinik nicht zu ersehen, ist jedoch wahrscheinlich.[509]

Dass die Jugendlichen nach Suizidversuchen zur psychiatrischen Begutachtung an die Klinik gebracht wurden, entsprach der organisatorischen Regelung, der zufolge die Klinik für akute psychische Krankheitsfälle Aufnahme- und Verteilereinrichtung für Wien, Niederösterreich und das nördliche Burgenland war. Mehrere hatten sich, um aus der Erziehungsanstalt herauszukommen, so schwer verletzt, dass sie wohl auch deshalb nicht für eine stark intervenierende Therapie in Frage kamen. Aber auch wenn sie aus dem Vergleich der Patienten aus Kaiserebersdorf mit bzw. ohne Fiebertherapie herausgenommen werden, ergibt sich kein klares Bild über die Indikation der Fiebertherapie. Wesentlich bedingt war diese Unklarheit durch die mangelnde Ausführlichkeit der Akten, in denen nicht für die Indikation argumentiert und auch zu wenig individuell differenzierend auf die Symptome eingegangen wurde – zu wenig, um durch eine eindeutige Vergleichsmöglichkeit gesicherte

---

[507] In den häufigen Fällen, in denen die Patienten nach kurzer Beobachtung oder längerer Behandlung auf den *Steinhof* gebracht wurden, ist das aus den Klinikakten nur bei nachfolgenden Aufnahmen nachzuweisen, aber auch sonst zu vermuten.

[508] Ein drastischer Fall war jener des 1952 18jährigen Patient P1582 aus Kaiserebersdorf mit der Diagnose „Psychopathie", der keine Fieber- oder andere ‚Erschütterungstherapie' bekam. Er kam nach einer Operation an die Klinik, da er, um aus „der Hölle" Kaiserebersdorf herauszukommen, Essbesteck verschluckt hatte (weshalb er vermutlich nicht für eine stark intervenierende Kur in Frage kam). Er benahm sich an der Klinik bei seinem 68tägigen Aufenthalt sehr zur Zufriedenheit der Pfleger, fügte sich als positives Zeichen sozialer Anpassung also gut in die Ordnung der Klinik ein. Als er bei der Entlassung bemerkte, dass er wieder nach Kaiserebersdorf zurückgebracht werden sollte, wehrte er sich und wurde gefesselt abgeführt. Zöglinge des Fürsorgeheims Eggenburg, die noch besprochen werden, gaben ähnliche Motive für ihre Suizidversuche an.

[509] Aufgrund der richterlichen Einweisung dürfte eine Entlassung auf Empfehlung der Klinik schwieriger zu beeinflussen gewesen sein, als aus dem Heim in Eggenburg, das der Jugenfürsorgebehörde unterstand; vgl. dazu die Beispiele im Abschnitt zu den Patienten aus dem Heim in Eggenburg.

Schlussfolgerungen ziehen zu können. Möglich ist auch, dass fallweise die Fiebertherapie gewählt wurde, um den Malariastamm zu erhalten.[510] Gleiches gilt auch für die Patienten aus dem Heim der Stadt Wien in Eggenburg, die im Folgenden besprochen werden.

c) Patienten aus dem Heim der Wiener Jugendfürsorge in Eggenburg

Das Heim in Eggenburg unterschied sich institutionell von jenem in Kaiserebersdorf : Während die Erziehungsanstalt in Kaiserebersdorf dem Justizministerium unterstellt war, Jugendliche nach einer Gefängnisstrafe aufnahm und die Klinik direkt in die psychiatrisch-psychologische Betreuung vor Ort einbezogen war, war das Heim in Eggenburg dem Magistrat der Gemeinde Wien unterstellt; es nahm von der Fürsorge betreute Jugendliche auf und die Klinik war nicht im Heim verankert, sondern – wie allgemein – bei akuten Krankheitsfällen und in der Begutachtung einbezogen.[511]

Von 38 Patienten, die aus dem Heim der Wiener Jugendfürsorge in Eggenburg an die Klinik kamen und in die Datenbank aufgenommen wurden,[512] bekamen 31 Patienten, die einmal oder mehrmals, mehr oder weniger als vier Tage lang an der Klinik aufgenommen wurden und nur in 24 Fällen[513] eine der fünf Diagnosen hatten, keine Malariafiebertherapie. Eine Malariafiebertherapie bekamen sieben von 38 Patienten bzw. fünf von 29 Patienten, die den Einschlusskriterien entsprachen. [514]

---

[510] Vgl. unten S. 142 und 159f.

[511] GEIGER, Kinderstation (2015), S. 267f. zitiert den Brief von Walter Spiel an das Bezirksjugendamt zum 12jährigen Patienten SK754 mit IQ 123, der den Unterschied der beiden Institutionen verdeutlicht: Der Knabe hatte 1957 mit der Diagnose Hebephrenie an der Kinderstation eine Insulinkoma- und Elektrokrampftherapien bekommen. Nach einer Verschlechterung seines Zustands 1958 empfahl Walter Spiel eine Unterbringung in einem Heim und schrieb: „Es muss allerdings betont werden, dass bei dem fraglichen psychotischen Geschehen, das sich ja abspielt, eine gewisse Gefährlichkeit des Minderjährigen gegeben ist. Eine ständige Beaufsichtigung ist unbedingt nötig. Nach der Insulinschockbehandlung war er kurze Zeit hindurch recht angepasst, zeigt aber jetzt wieder sein hypomanisches Verhalten, seine Bosheitsakte und seine Aggressionen. Sollte er in einem halb offenen Heim wie Eggenburg nicht zu führen sein, wird man, da ja kriminelle Delikte vorliegen, schliesslich doch ihn versuchsweise nach Kaiser Ebersdorf geben müssen."

[512] Auch hier ist auf die genannten Ungenauigkeiten in der Datenaufnahme hinzuweisen: Aktenverluste, Aufnahme aller Malariatherapiepatienten, aber nur jener Patienten ohne Fiebertherapie, die den Aufnahmekriterien entsprachen bzw. deren Herkunft aus dem Heim als Wohnadresse angegeben ist.

[513] Es wurden hier auch 7 Patienten aus dem Heim in Eggenburg aufgenommen, die keine Malariafiebertherapie bekamen und auch keine der 5 Diagnosen hatten; ihre Diagnosen: 1954 1mal „neurotische Verwahrlosung" und in den 1960er Jahren 2mal „Alkoholrausch", 1mal o.B., 1mal „SMV im betrunkenen Zustand", 1mal „SM-Drohung, neurotisch" und 1mal „Verhaltensstörung".

[514] Zwei Eggenburger Zöglinge bekamen die Malariakur mit Diagnosen außerhalb der Einschlusskriterien: Ein 17jähriger 1957 mit der Diagnose „Enuresis, Enkopresis" und ein 16jähriger 1961 mit der Diagnose „SMV, neurotische Verwahrlosung".

Ein aussagekräftigerer Vergleich ergibt sich, wenn nur jene in der Datenbank verzeichneten Eggenburger Patienten zwischen 1951 und 1961 mit und ohne Malariafiebertherapie einbezogen werden. Denn die 31 Patienten aus Eggenburg ohne Malariafiebertherapie sind über den ganzen Untersuchungszeitraum (1951-1969) verteilt, die sieben Patienten mit Malariafiebertherapie bekamen diese jedoch – wie die in den Akten überlieferten vier Malariafiebertherapie-Patienten aus Kaiserebersdorf – nur in den Jahren zwischen 1951 und 1961. Zu vergleichen wären nun nur sechs Patienten ohne mit sieben Patienten mit Malariakur, was für diese elf Jahre einen extrem hohen Anteil von Patienten mit Malariafiebertherapie ergibt.

Im Vergleich der Diagnosen fällt auf, dass von den sieben Patienten mit Malariafiebertherapie fünf einen Suizidversuch gemacht oder mit Suizid gedroht hatten,[515] keiner hingegen von den sechs Patienten, die keine Fiebertherapie bekamen.[516] Das ändert sich in den Jahren 1962 bis 1969: Von den in diesen acht Jahren aufgenommenen 25 Patienten aus dem Heim in Eggenburg – alle ohne Fiebertherapie – hatten fünfzehn die Diagnose Psychopathie und davon zehn Patienten den Vermerk Suizidversuch oder Suizid-Androhung in der Diagnosezeile. Ist daraus zu schließen, dass 1951 bis 1961 die jugendlichen Patienten mit Suizidtendenzen mit Malariafiebertherapie behandelt wurden und nicht mehr in den Jahren danach? Dagegen spricht, dass bei den Patienten, die bis 1961 aus der Erziehungsanstalt Kaiserebersdorf kamen, dieser Vergleich kein ähnliches Bild zeigt: Von den vier Patienten, die eine Fiebertherapie bekamen, hatten zwei einen Suizid versucht, von den 9 Patienten, die zwischen 1951 und 1961 aus Kaiserebersdorf eingewiesen wurden und keine Fieberkur erhielten, hatten 3 Patienten einen Suizid versucht bzw. angedroht und ein vierter ohne Suizidabsicht einen Metallgegenstand verschluckt, um aus der Bundesanstalt herauszukommen.

Die Häufigkeit der Patienten aus dem Heim in Eggenburg, die lt. Datenbank mit einer Malariafiebertherapie behandelt wurden, im Vergleich zu jenen, die von dort in die Klinik Hoff kamen und keine Fiebertherapie erhielten, ist nicht eindeutig zu interpretieren. Zum

[515] Von diesen fünf Patient hatten vier die Diagnose Psychopathie (mit oder ohne Kombination). Der fünfte Patient X802, der als 16jähriger 1961 mit der Diagnose „SMV, neurotische Verwahrlosung“ eine Malariafieberkur bekam, hatte bei seiner zweiten Aufnahme 6 Jahre später ebenfalls die Diagnose „Psychopathie“.
[516] Vier hatten die Diagnose Psychopathie (2mal kombiniert mit „Debilität“), einer „Schizo“ (aber bei einem späteren Aufenthalt „Psychopathie“) und einer „neurotische Verwahrlosung“.

einen ist festzuhalten, dass die Zahl der Beispielsfälle sehr gering ist. Zum anderen ist – wie bereits erwähnt – die größere Ungenauigkeit bei der Erhebung der Patienten ohne Fiebertherapie zu berücksichtigen. Eine relativ hohe Zahl von PatientInnen mit einer Fiebertherapie ist aber wohl aufgrund des Alters der Zöglinge und ihrer Diagnosen zu erwarten gewesen: aufgrund der Meinung, Psychopathie sei nur im Jugendalter therapierbar.

Wie bei einzelnen der Patienten aus der Erziehungsanstalt Kaiserebersdorf,[517] so sprach sich die Klinik auch bei Patienten aus Eggenburg manchmal gegen eine Rückkehr ins verhasste Erziehungsheim und für eine Unterbringung in einem Lehrlingsheim aus.

> So hieß es im Entlassungsbrief an das Jugendamt der Stadt Wien zum 1952 17jährigen Patienten P825¸ der wiederholt aus dem Heim zur Mutter geflohen war und zuletzt einen Suizid versucht hatte: „Es handelt sich bei dem Patienten um eine Psychopathie mit cyclischen Gemütsschwankungen. Der Patient wurde nach einem SMV eingeliefert. Es wurde eine Malariabehandlung durchgeführt und er ist derzeit angepasst und symptomfrei. Der Patient ist als gesund zu betrachten. Da im häuslichen Milieu Schwierigkeiten bestehen und außerdem eine große Abneigung gegen die Anstalt Eggenburg besteht und da wir eine Wiederholung des SMV befürchten müssen, falls der Pat. nach Eggenburg zurückkehren würde, empfehlen wir den Pat. in das Jugendarbeiterdorf von Herrn Dr. Buchwieser einzuweisen. Es besteht der Vorteil, dass der Pat. seine in einem halben Jahr ablaufende Lehrzeit bei Herrn Dr. Buchwieser beendigen könnte.“[518]

Gut möglich, dass der Erfolg der Behandlung – symptomfrei und vor allem (sozial) „angepasst“ – neben der Suizidgefahr (bei diesem Patienten mit „cyclischen Gemütsschwankungen“) zu diesen Verbesserungsvorschlägen für den Zögling beigetragen hat. Die Lehre galt dem therapeutischen Ziel der sozialen Integration in eine geregelte Arbeitswelt. Ähnlich war es im Fall des 1957 17jährige Patient P/I438 aus Eggenburg mit der Diagnose „Psychopathie, Debilität, SM-Drohung“[519] und heftigen Erregungen, der „nach einer Malariakur,[520] Psycho- und Arbeitstherapie […] gut angepasst“ in das Lehrlingsheim in Wien 2 entlassen wurde. Das therapeutische Ziel einer sozialen Anpassung – nicht nur durch Arbeitstherapie (und Psychotherapie), sondern vermutlich auch durch ihre Kombination mit

[517] 1951 beim 17jährigen Patienten S2433 mit der Diagnose „Hebephrenie“, da für ihn „eine Bundesanstalt für Erziehungsbedürftige nicht geeignet“ sei und er „weiterer Anhaltung und Behandlung in einer Heilanstalt“ bedürfe, und 1956 beim 17jähriger Patienten P5391 mit der Diagnose „Psychopathie, Homosexualität, SM-Tendenz“, bei dem die Klinik von einer Rückkehr nach Kaiserebersdorf abgeraten hat, da er als besonders schwer suizidgefährdet eingeschätzt wurde – obwohl er nicht über die Erziehungsanstalt klagte. Beide Patienten hatten keine Malariafiebertherapie bekommen.

[518] 1967 kam er nach einem SMV mit einer Medikamentenüberdosis im Rausch wieder an die Klinik.

[519] Er „wanderte von Heim zu Heim“ und habe sich ohne „kontinuierliche Erziehung“ zu einer „bildungsarme[n] und affektkalte[n] Persönlichkeit“ entwickelt.

[520] Auf der Anfrage an den Internisten ist am 3. Tag der Aufnahme als Diagnose „Debilitas“ angegeben. Für die Indikation könnte das Symptom ‚heftige Erregung‘ bei dieser Diagnose den Ausschlag gegeben haben.

der „Erschütterungstherapie“ – ist als zentrales Motiv in der Behandlung der ‚asozialen‘ Jugendlichen mit dem „psychopathischen Syndrom“ anzunehmen.

Im folgenden Fall dürfte ebenfalls das sozial integrierte Verhalten des Patienten, wie auch seine Offenheit gegenüber psychotherapeutischen Führungsgesprächen zur Empfehlung beigetragen haben, den Jugendlichen zu seiner Mutter zu geben. Im Befundbericht an das Jugendamt zeigt sich außerdem die Hierarchie der beiden Erziehungsheime indem angedroht wurde, den Patienten ins Erziehungsheim Kaiserebersdorf einzuweisen, sollte der Versuch der Rückführung aus der Fürsorgeerziehung in die „Familienerziehung“ scheitern:

> Im Befundbericht an die Magistratsabteilung XI (Jugendamt) zum 15jährigen Patient P445, der 1957/58 mit der Diagnose „Psychopathie, demonstrativer SMV“ eine Malariafiebertherapie bekommen hatte, hieß es: die Vorgeschichte und die klinische Beobachtung würden zeigen, „dass es sich bei dem Genannten um eine schwer erziehungsgeschädigte Persönlichkeit handelt, so dass die bisherigen Versuche, eine soziale Anpassung bzw. Eingliederung zu erreichen, völlig nutzlos waren.“ Nach Rücksprache mit den Eltern, „erscheint es uns jedoch richtig, noch einmal den Versuch einer Familienerziehung […] zu machen und den Jugendlichen zu seiner Mutter zu geben.“ Es sei „allerdings unumgänglich, dass eine intensive, nachgehende Fürsorge mit entsprechenden Kontrollbesuchen stattfinden“ und klargestellt würde „dass dieses Abgeben des Patienten in das […] Familienmilieu, den letzten Versuch darstellt, eine entsprechende, eingliedernde Entwicklung zu erzielen. Sollte dieser letzte Versuch fehlschlagen, müsste eine dauernde Fürsorgeerziehung bzw. Internierung in einem geschlossenen Heim angeraten werden. In diesem Falle käme nur mehr Kaiser-Ebersdorf in Frage.“ Wichtig für die Kliniker war wohl auch, dass sich der Patient an der Klinik sehr positiv verhalten hatte.[521] Der Patient wurde nach seinem 68tägigen Aufenthalt, bei dem er eine Malariafiebertherapie bekommen hatte, nach Hause entlassen, obwohl die Fürsorgebehörde dagegen war.[522]

Die Klinik dürften sich bei Zöglingen aus Eggenburg leichter durchgesetzt haben, da hier nur die Wiener Jugendfürsorge und nicht wie bei den Zöglingen aus Kaiserebersdorf zusätzlich auch die Justizbehörde zu überzeugen und die gerichtliche Heimeinweisung zu revidieren war. Dass in Egenburg im Unterschied zu Kaiserebersdorf eine institutionalisierte psychiatrisch-psychologische Betreuung fehlte, für eine psychiatrische Beobachtung und Weiterbetreuung also keine Möglichkeit bestand, dürfte auch in Einzelfällen zur Entscheidung

[521] In einem *Decursus*-Eintrag hieß es am 17. Tag: „Während seines Aufenthaltes hier wird d. Versuch einer psychoth[erapeutischen] Führung unternommen, welcher d. Pat. weitgehend aufgeschlossen gegenübersteht.“ Am 28. Tag wurde dem Patienten malariainfizierte Blut geimpft und 2 Tage später hieß es: „Pat. macht einen ziemlich geordneten Eindruck und ist vollkommen zufrieden, dass er vorläufig hier an der Klinik bleiben kann. Es bestehen keine disziplinäre od. sonst. Schwierigkeiten bei ihm, er ist willig und bei den Mitpat[ienten] ziemlich beliebt.“

[522] Die Magistratsabteilung XI sah in einem Schreiben an die Klinik Hoff die Entlassung aus Eggenburg „wegen des kaum begonnenen Erziehungsversuches [als] verfrüht“ an.

gegen eine Rücküberweisung beigetragen habe.[523] In Eggenburg scheint durch diese Entscheidungen die Hoffnung verbreitet gewesen zu sein, nach einem Suizidversuch von der Klinik nicht mehr ins verhasste Heim zurückgebracht zu werden. So berichtete 1958 der 16jährige Patient P473, der mit der Diagnose „SMV, Psychopathie, Erregungszustände" eine Malariafiebertherapie bekam: er wisse von Kameraden, „dass er, wenn er hier an der Klinik war, wahrscheinlich nicht mehr nach Eggenburg zurückkehren" müsse.

d) Zwischenresümee zur Malariafiebertherapie bei PatientInnen aus Heimen

„Psychopathie", manchmal auch „Verwahrlosung" als Vorstufe bzw. „Grundlage" der Entwicklung eines psychopathischen Syndroms, wurde bei der Mehrheit der Zöglinge aus den Heimen in Eggenburg und Kaiserebersdorf diagnostiziert. Wie bei anderen jugendlichen Patienten mit der Diagnose Psychopathie bestand die besondere Schwierigkeit in der Beurteilung ihrer Behandlung mit der Malariafiebertherapie darin, dass in der eingesehenen zeitgenössischen Literatur kein Hinweis auf diese Indikation bei Psychopathie gefunden wurde. Da in Wien „Psychopathie" als Syndrom definiert wurde, war erwartet worden, dass das Motiv für die Therapie bei den Patienten aus dem Bundeserziehungsheim Kaiserebersdorf, die von Klinikern bereits im Heim beobachtet worden waren, hier besonders deutlich an Symptomen bzw. an Symptomkombinationen festzumachen und dadurch eine Begründung für die Anwendung zu erkennen wäre. Die oben zitierten Wiener Publikationen der Zeit zu den Zöglingen in Kaiserebersdorf lassen zwar die Bedeutung von Symptomen für jede Indikation erkennen, eine systematisch und regelmäßig auf bestimmte Symptome bezogene Indikation der Malariafiebertherapie als „Erschütterungstherapie", dürfte jedoch wie bei den anderen nicht-luetischen Diagnosen auch hier nicht möglich sein – sei es wegen der geringen Zahl der Fälle, sei es, da die Informationen in den Krankenakten und in den Publikationen zu knapp sind oder überhaupt fehlen, sei es wegen einer nicht eindeutigen Praxis. Von Patienten mit gleicher Diagnose und ähnlicher Lebens- und Krankengeschichte bekamen die einen eine Fiebertherapie, die anderen nicht.

---

[523] Das lässt 1961 der Fall des 15jährigen Patienten X802 vermuten, der mit der Diagnose „SMV, neurotische Verwahrlosung" „mit depressiv-neurotischen Zügen" („gut begabte, IQ. 119") eine Fiebertherapie bekam und zur Großmutter entlassen wurde. Im Befundbericht an das Jugendamt hieß es: der im Heim weiterhin suizidgefährdete Patient werde „angewiesen werden, sich regelmässig zur ambulanten Kontr[olle] an der Klinik einzufinden."

Die Malariafiebertherapie scheint bei einer Minderheit der PatientInnen mit dieser und anderen Diagnosen außerhalb der Neurolues mit heftigen Erregungszuständen, Suizidtendenzen, Aggressivität, Uneinsichtigkeit in die Krankheit und Unzugänglichkeit, zur Beruhigung, zur „Umstimmung", zur „Nachreifung" und zur Beförderung einer sozialen Anpassung[524] gegeben worden zu sein. So wie bei den anderen Diagnosen außerhalb der Neurolues dürfte einer der Gründe für ihre Anwendung aber auch die Aufrechterhaltung des Malariastamms gewesen sein, wobei die Frage vermutlich nicht zu beantworten ist, ob dies im bestimmten Fall zur Wahl dieser Therapie führte, die aus der Erfahrung seit der Zwischenkriegszeit als wirksam angesehenen wurde – vielleicht auch als besser wirksam und verträglich, als eine Therapie mit den neuen Medikamenten –, oder ob dies ohne therapeutische Erwartung erfolgte, nur um den Stamm zu erhalten.

Bei Jugendlichen, die eine Malariafiebertherapie bekamen, wurde im Krankenakt manchmal „(Nach-)Reifung" als therapeutisches Ziel erwähnt. Reifung wurde nach einem Zeitzeugen auch gegenüber AssistenzärztInnen als Motiv für die Anwendung der Malariakur bei den Patienten aus dem Erziehungsheim Kaiserebersdorf genannt.[525] Auch in Publikationen der Wiener KlinikerInnen findet sich Reifung als therapeutisches Ziel in der Behandlung der Psychopathie, als Therapie werden jedoch nur Gruppen- und Psychotherapie sowie Erziehung erwähnt.[526] Bei jungen PatientInnen mit der Diagnose „Pfropfschizophrenie" („Pfropfhebephrenie")[527] und bei „zerebralgeschädigten retardierten Individuen"[528] mit Reifestörung wird hingegen in Wiener Publikationen auch eine Anwendung der Malariakur mit dem Ziel einer Reifung erwähnt.

Bei den Jugendlichen mit der Diagnose Psychopathie legt der Begriff „Reifung" („Nachreifung") als Motiv für die Malariafiebertherapie die Hypothese nahe, dass mit der Kur die Verzögerung der Entwicklung behoben werden sollte, die als Ursache für das asoziale Verhalten, für die Verhinderung der Anpassung des Jugendlichen an die sozialen Normen gesehen wurde. Diese Meinung einer therapeutische Wirkung der körperlichen Therapie als

[524] Vgl. oben S. 139f zu den beiden Patienten P825 und P/I438.
[525] Interview Gernot Heiss mit Bernd Küfferle am 06.05.2013.
[526] Vgl. z. B. oben auf S. 115 das Zitat aus RINGEL/SOLMS/SPIEL, Die Therapie der Psychopathie (1960), S. 456, in dem sie von „therapeutisch orientierte Nacherziehung" der „unausgereiften Persönlichkeit" von PsychopathInnen sprechen.
[527] Siehe oben S. 83 das Zitat von ARNOLD, Die Therapie der Schizophrenie (1963), S. 59-61.
[528] Vgl. oben S. 93 Anm. 325 die Zitate aus SPIEL, Die Therapie in der Kinder- und Jugendpsychiatrie (1967), S. 140 und – in der Anmerkung – aus MÜLLER-KÜPPERS, Die Therapie im Kindes- und Jugendalter (1972), S. 998.

„Nachreifung“ wurde durch die Beobachtung bestärkt, dass Besonderheiten im EEG, die nur bei kleinen Kindern in der Norm vorkommen, aber auch bei „psychopathischen“ Jugendlichen noch häufig nachzuweisenden sind, manchmal nach der Kur und dann sukzessive auch das asoziale Verhalten verschwinden würden.[529] Nach den drei Orientierungen der Motive der psychiatrischen Therapien in der Untersuchung der Züricher Psychiatrie „Zwang zur Ordnung“ von 2007, ob die Therapie therapeutisch und / oder disziplinierend auf die Herstellung der „Ordnung des Selbst“, der „Anstalts-“ und / oder der „Gesellschaftsordnung“ gerichtet ist, wäre hier eine doppelte Orientierung zu sehen: therapeutisch auf den Reifungsrückstand und disziplinierend auf das asoziale Verhalten: Die Malariakur würde angewandt, um die intellektuelle Reifung zu aktivieren und so dem / der Patienten/in zu ermöglichen, unterstützt durch die nachfolgende Arbeitstherapie, Gruppentherapie und / oder Erziehung aus eigenem[530] die durch den Reifungsrückstand „gestörte Ordnung des Selbst“ herzustellen. Der körperlich heilenden Therapie folgen in dieser Hypothese die sozial disziplinierenden Therapien, von denen gemeinsam eine Besserung erwartet wurde. Die therapeutische Orientierung war jedenfalls mehrschichtig. Dem Krankheitsbild der Psychopathie als asoziales Verhalten entsprechend, hatte freilich die Therapie der Psychopathie das Ziel, eine Anpassung des sozialen Verhaltens an die Normvorstellung zu erreichen, woraus hier Sozialdisziplinierung ebenfalls zum Ziel der Therapie[531] wird.

Im Zusammenhang mit dem Erziehungsheim in Kaiserebersdorf und im Wissen um die dort in den 1950er und 1960er Jahren angewandten Strafen stellte sich die Frage, ob die dem Heim zugeordneten Psychiater die Malariafieberkur auch als Erziehungs- bzw. Sanktionsmaßnahme[532] quasi als erweiterter Arm des Erziehungsinstituts gegen die als ‚schwererziehbar‘ geltenden Jugendlichen einsetzten. Dafür fanden sich keine Belege, keine ausdrücklich als Bestrafung bezeichnete Anwendung - anders als in den Institutionen der

---

529 Vgl. auch die dritte Form der Psychopathie nach HOFF/SLUGA, Das psychopathische Syndrom (1962), S. 156f. und S. 169f., zitiert oben S. 111-114.

530 Demnach hat psychiatrische Therapie nicht das Ziel, den / die Patienten/in zu einem den Normen angepassten Verhalten zu zwingen, sondern das Ziel, dem / der Patienten/in die Kraft und Mittel zu geben, um ihm / ihr selbst eine Anpassung des eigenen Verhaltens zu ermöglichen.

531 Zur Arbeitstherapie mit dem Ziel der sozialen (Wieder-)Eingliederung vgl. GERMANN, Arbeit als Medizin: Die „aktivere Krankenbehandlung“ 1930 – 1960 (2007), S. 195-234.

532 Vgl. dazu die Kritik am Projekt der Psychoanalytikerin und Psychiaterin Dr. med. Elisabeth Brainin, die als Expertin in die Kommission eingeladen worden war: sie nannte in ihrer Stellungnahme zum Endberichtsentwurf vom 8.5.2015 bei ihrem Austritt als „wesentliche[n] Einwand“, dass es „wichtig gewesen wäre nachzuprüfen, ob die erwähnten Behandlungsmethoden (Insulinschocktherapie, EKT, Malariatherapie, Cardiazolschock etc.) in bestimmten Fällen als Sanktionsmittel eingesetzt wurden.“

Behindertenhilfe und Psychiatrie für Kinder und Jugendliche in der BRD und DDR 1949 bis 1990, zu denen im Sammelband „Leid und Unrecht“ von 2021 publiziert wurden.[533]

In der eingesehenen zeitgenössischen Literatur finden sich Einschätzungen zur Wirkung von Bestrafung bei PatientInnen mit den Diagnosen Psychopathie bzw. (neurotische) Verwahrlosung. Zum Teil betrifft das wohl nur gerichtliche Strafen,[534] in einigen Fällen aber auch physische Erziehungsmaßnahmen. So schrieb W. H. Heaton-Ward 1963: „Psychopaths certainly do not respond well to the usual methods of discipline or to physical methods of punishment”.[535] Auch in Wiener Publikationen wurde auf die negative Wirkung von Bestrafung bei diesen PatientInnen verwiesen: Im Buch über den Suizid von Erwin Ringel heißt es 1953 im Kapitel über „das präsuicidale Syndrom“ zu Patienten nach einem SMV mit der Diagnose Neurose und Psychopathie: „Die Entwicklung dieser Menschen erfolgt immer unter besonders ungünstigen inneren und äußeren Umständen und führt zu Aggressionstendenzen, die so mächtig sind, daß sie auch die Umgebung betreffen müssen. Andererseits ist – analytisch gesprochen – ihr Bestrafungswunsch so groß, daß sie nicht nur durch sich selbst, sondern auch durch andere bestraft werden wollen (worauf besonders Alexander hinwies).“[536] Ähnlich kamen Manfred Haider und Walter Spiel 1956 in ihrem oben zitierten Artikel zum Jugendstrafvollzug in Bezug auf die Gruppe der „neurotisch Verwahrlosten“ zum Urteil, dass durch deren Bestrafung ein durch Schuldgefühle bestehendes „‚Strafbedürfnis’“ „abgesättigt“ würde.[537]

Eine weitere Frage ist, ob die Vormundschaft der Fürsorgebehörden die Erteilung der Erlaubnis (des Reverses) für eine der schwer eingreifenden körperlichen Therapien im Vergleich zur elterlichen Erlaubnis erleichterte. Aufgrund der mehrfachen Zusammenarbeit der Klinik mit der Fürsorge ist das zu vermuten. Dass diese Erleichterung der

---

[533] Die Begriffe Disziplinierung und Bestrafung werden hier auseinandergehalten: als Bestrafung werden nur solche Maßnahmen bezeichnet, die explizit als solche argumentiert werden. Im Sammelband „Leid und Unrecht“ von 2021 werden Beispiele für die Anwendung der EKT als Bestrafung in dieser Definition genannt. Außerdem werden aber auch Fälle der Behandlung von Unruhe- und Aggressionszuständen mit EKT als Disziplinierung, gleichgesetzt mit ‚Bestrafung‘ erwähnt (vgl. Christof BEYER, Kinder- und Jugenpsychiatrische Abteilung der Pfälzischen Nervenklinik Landeck [2021], S. 663), die im vorliegenden Text als Indikation zur Beruhigung mit ambivalenter (therapeutischer und disziplinierender) Zielsetzung und nicht als ‚Bestrafung‘ im engeren Sinn gesehen würden.
[534] So vermutlich bei PALMER, Psychopatic personality (1959), S. 40: “Punishment may deter, certainly it will not cure.”
[535] HEATON-WARD, Psychopatic Disorder (1963), S. 122.
[536] RINGEL, Der Selbstmord (1953), S. 111.
[537] Vgl. oben S. 133.

Genehmigung[538] in Einzelfällen zur Verwendung als Stammträger ohne therapeutische Indikation führte, ist allerdings nicht zu belegen.

2.1.4.2.5 Malariafiebertherapie bei den Diagnosen Alkoholismus, Neurose, Verwahrlosung (insb. Alkoholismus)

In den 1960er Jahren mehren sich Malariakuren bei Patienten[539], welche mit Diagnosen aufgenommen wurden, die in den 1950er Jahren keine Indikation zur Malariafiebertherapie dargestellt haben. Sie haben in der Diagnosezeile am Deckblatt keine der fünf Diagnosen der Einschlusskriterien des Projekts eingetragen.[540] So finden sich nun Patienten, die an der Klinik mit Malariafiebertherapie behandelt wurden, mit den Diagnosen „Alkohol chr[onisch]“, „Neurose“, „neurotische Verwahrlosung“, „innere Verwahrlosung“ „Aggressionsneurose“, „Hysterie, funktionelle Anfälle“[541] – ohne oder mit Diagnosekombinationen und Zusätzen, wie SMV, Zwangsmechanismen, Erregungszustände.[542]

Im Folgenden geht es um Patienten mit Alkoholproblemen, die eine Malariafiebertherapie bekamen[543] und in deren Patientenakten Bemerkungen auf einen Zusammenhang der Malariafiebertherapie mit ihrer Alkoholkrankheit schließen lassen.

---

[538] Der Revers dürfte aufgrund der damals recht allgemeinen Akzeptanz der Entscheidungsautorität des Arztes / der Ärztin von Eltern ebenfalls nicht schwierig zu erhalten gewesen sein. In der Datenbank finden sich nur zwei Fälle einer Ablehnung: 1965 lehnte die Pflegemutter des Patienten S7381 eine neuerliche Elektrokrampftherapie erfolgreich ab und 1962 die Eltern des Patienten S8744 eine neuerliche Majeptilkur (vgl. unten S. 202 Anm. 786.

[539] Darunter waren fast keine Patientinnen. In den 1960er Jahren finden sich in den Akten nur je eine Patientin 1960 bzw. 1963 (I904 und P194) mit einer Malariafiebertherapie ohne Neurolues, beide hatten eine Diagnose, die den Einschlusskriterien entsprach; vgl. oben S. 60 Anm. 183 und S. 63.

[540] Den Einschlusskriterien entsprachen die Diagnosefelder: Neurolues, Intelligenzmängel, Schizophrenie, affektive Störungen, Psychopathie.

[541] Dem 18jährigen zeitverpflichteten Soldaten X849 mit der Diagnose „Hysterie, funktionelle Anfälle“ wurde 1968 die Malariakur zur Beruhigung gegeben, nachdem die hysterischen Anfälle „selbst mit hohen Dosen Valium i. v. nicht kupiert werden“ konnten. Nach kurzer Fiebertherapie und Psychotherapie bzw. Hypnose konnten „die Frequenz der Anfälle bis zum Verschwinden herabgesetzt werde. Er sollte wegen seiner psychischen Probleme nicht mehr zum Bundesheer zurückkehren.

[542] Im Text und in der Tab. 12 oben auf Seite 62 sind mit drei zusätzlichen Diagnose-Klassen auch diese 97 Fälle erfasst, die eine Malariafiebertherapie bekamen, deren Diagnosen jedoch nicht den Einschlusskriterien des Projekts entsprechen. Da die Fälle der PatientInnen mit diesen Diagnosen ohne Malariakur nicht aufgenommen wurden, können dazu freilich keine Vergleichszahlen gegeben werden.

[543] Es war eine kleine Zahl, während die meisten der mit Alkoholproblemen aufgenommenen PatientInnen (sie dürften häufig die größte Gruppe gewesen sein) in den ersten vier Tagen *auf dem Steinhof* überstellt oder entlassen wurden. Im April 1964, zu dem alle Patientenakten der Männerstation in die Datenbank aufgenommen wurden, zeigt sich, dass bei 90 von 190 im April aufgenommenen Patienten ein Alkoholproblem in der Diagnose erwähnt wurde (47,36%), und dass die Klinik für die überwiegende Mehrheit von ihnen eine Durchgangsstation war: 84 von diesen 90 Patienten blieben weniger als fünf Tage, das sind 93,3%; eine Malariafiebertherapie bekam keiner der sechs verbleibenden Alkoholkranken. Zu diesem Schwerpunkt der Klinik meint FORSTER,

Bei mehreren der durchwegs jungen Patienten wurde eine Malariafiebertherapie vor einer geplanten Entwöhnungskur gegeben – ab 1961 im *Genesungsheim Kalksburg*, das in enger Verbindung mit der psychiatrischen Klinik stand.[544] Im Folgenden werden dazu nur zwei Fälle von sechs als Beispiele beschrieben:

> Der Patient P884 kam 1965 als 20jähriger mit der Diagnose „Psychopathie, Alc. Missbrauch" „freiwillig zur Aufnahme an die Klinik. […] Sowohl seine Lebensgefährtin, […] als auch seine Mutter drängen ihn, das Trinken aufzugeben. Da er sich selbst dazu nicht imstande fühlt, möchte er sich einer Entziehungskur unterziehen." Laut Befundbericht an den Betriebsarzt der Arbeitsstelle „[lag] der Patient […] unter der Diagnose Psychopathie, Alkoholmissbrauch hier und machte eine Fieberkur durch. Von uns wurde er in das Genesungsheim Kalksburg zwecks Durchführung einer Alkoholentwöhnungskur entlassen."
>
> Der 1967 21-jährige Patient X822 mit der Diagnose „Alk. Chron." wurde von Kalksburg an die Klinik zur Malariafiebertherapie gesandt. In der Anamnese heißt es: „Der Pat. kommt vom Genesungsheim Kalksburg wegen einer Fieberkur zu uns zur Aufnahme." Er war in *Kalksburg* acht Tage vorher „wegen Alk. Chronicus zur Entwöhnungskur direkt aufgenommen worden." Nach Beendigung der Fiebertherapie wurde er wieder an das *Genesungsheim Kalksburg*, also zur Weiterführung der Entwöhnungskur, rücküberstellt.

Während in diesen beiden und in vier weiteren Fällen im Patientenakt deutlich wird, dass der Patient nach der Malariafiebertherapie eine Entwöhnungskur absolvierte, ist in anderen Fällen zwar eine Absicht dazu aus den Akten ersichtlich, sie wurden jedoch nach der Entlassung aus der Klinik nicht oder nicht direkt ins Genesungsheim überstellt. So im folgenden Beispiel, in dem die Initiative des stellvertretenden Leiters des *Genesungsheims Kalksburg* Kornelius Kryspin-Exner für die Behandlung vor dem Entzug an der Klinik, wo er parallel als Assistenzarzt und ab 1966 als Leiter der psychiatrischen Männerabteilung tätig war, besonders deutlich wird:

> Bei dem 1965 aufgenommenen Patienten P724 diagnostizierten die Ärzte der Klinik Hoff „Psychopathie, Grenzdebilität, chron. Alkoholmissbrauch". In der Anamnese wurde festgehalten, dass der Patient – ein maschinenkundiger Landarbeiter – seit seinem zehnten Lebensjahr trank und wiederholt in Raufhändel verwickelt und

---

Staat, Politik und Psychiatrie in Österreich – am Beispiel der rechtlichen Regulierung von Zwangsmaßnahmen von 1916 bis 1990 (1999), S. 176, es sei dem einflussreichen Hoff in der Diskussion der 1950er Jahre um eine „Erweiterung des Kreises der anzuhaltenden Personen, insbesondere auf Suchtkranke" gegangen. Denn bei den Alkoholikern würde „sich den um ihren Status ringenden Psychiatern die Chance [bieten], ihre therapeutische Kompetenz zu beweisen und ihren Ruf in der Öffentlichkeit zu beweisen."

[544] 1961 gegründet, seit 17.1.1961 stellvertretend geleitet und ab 1969 geleitet durch Kornelius Kryspin-Exner, der zugleich „Hilfsarzt", ab 1.7. 1964 „Assistenzarzt", ab 17.1.1967 Dozent und ab 1966 Leiter der Männerabteilung an der Klinik war (1972 tit. ao. Prof.). Zur Verbindung des *Genesungsheims Kalksburg* mit der Klinik vgl. KRYSPIN-EXNER, Die moderne Behandlung des Alkoholkranken (1963), S. 194: „eine offene Anstalt für Alkoholkranke […], die unter der Leitung der Wiener Universitäts-Nervenklinik steht".

straffällig geworden war, um an Geld für den Kauf von alkoholischen Getränken zu gelangen. Der nun 18-jährige kam in Begleitung eines Fürsorgebeamten zur „freiwilligen“ Aufnahme an die Klinik Hoff. Im Anamneseblatt aus *Kalksburg*, das dem Patientenakt beiliegt, heißt es zum Plan einer Entwöhnungskur, er sei dort „Herrn Prim. Dr. Kryspin zur Begutachtung vorgestellt“ worden und „Herr Prim. hat zugesagt, [ihn] in der Klinik Hoff vorzubehandeln und ihn anschliessend in das Genesungsheim Kalksburg zu überstellen.“ An der Klinik bekam er die – als ‚Vorbehandlung‘ vorgesehene – Malariafiebertherapie.

Durch den Hinweis auf eine ‚Vorbehandlung‘ für die Entzugskur wird hier der Bezug der Malariakur auf die Alkoholkrankheit deutlich – wie auch im folgenden Fall:

Zum 19-jährigen Patienten I733, der 1965 mit der Diagnose „Chron. Alkoholmissbrauch“ (lt. Anamnese „Intelligenzschätzung: Grenzdebilität[545]. Zeichen des psychopathischen Syndroms“) eine Malariafiebertherapie bekam, hieß es, er sei an der Klinik in „Vorbereitung zur Entwöhnungskur“ behandelt worden.

Mit der Erwähnung der Malariafiebertherapie als ‚Vorbehandlung‘ zu einer Entwöhnungskur ist zwar eine therapeutische Absicht angesprochen; es bleibt jedoch unklar, was mit dieser Vorbereitung erreicht werden sollte.

Zum Alkoholismus wurde in Wien die Meinung vertreten, dass hinter dem Alkoholismus eine psychische Krankheit stehe, die diagnostiziert und behandelt werden müsse.[546] Kornelius Kryspin-Exner schrieb, dass Alkoholismus auf einer Persönlichkeitsstörung beruhe und „ein Hauptteil der Therapie auf diese Persönlichkeitsstörung gerichtet sein“ müsse. Dabei sei im „Gesamtbehandlungsplan“ zu berücksichtigen, ob es sich um „einen neurotischen Trinker“ einen „psychopathischen Trinker […], , der keine echten sozialen Kontakte eingehen kann und nicht dem Realitäts-, sondern dem Lustprinzip lebt“, oder einen „Schwachsinnigen“, einen „Gehirngeschädigten“ oder einen Menschen handle, „der auf Grund seiner Erbanlagen zu abnormen Stimmungsschwankungen neigt“.[547] Hoff meinte 1956, dass eine „große Gruppe der chronischen Alkoholiker“ „von Psychopathen gestellt“ würde[548] – und von den 24

545 „Grenzdebilität“ wurde im Hoff-Skriptum, Allgemeine Psychiatrie [um 1961], S. 29, mit einem IQ von 80-85 definiert. Grenzdebilität fällt in der ICD-8 in den Bereich der „Grenzfälle von Intelligenzmängeln“, für die ein IQ von 68-85 angegeben wurde.

546 Vgl. auch ARNOLD/ ROTTER, Elektroschockbehandlung des symptomatischen Alkoholismus (1954), S. 391: Alkoholismus sei „ein Symptom dahinterstehender seelischer Störungen“. Bei 15% seien es „Verstimmungszustände aus dem manisch-depressiven Formenkreis“. Hier schlagen die Autoren die „bei der Depression übliche Elektroschockbehandlung“ vor.

547 KRYSPIN-EXNER, Die moderne Behandlung des Alkoholkranken (1963), S. 191: Die Krankheit entwickle sich bei „Alkoholkranken auf Grund einer gestörten Persönlichkeit“, weshalb „ein Hauptteil der Therapie auf diese Persönlichkeitsstörung gerichtet sein muß“. Vgl. KRYSPIN-EXNER. Probleme der Bekämpfung des Alkoholismus in Österreich (1965), S. 646.

548 Vgl. HOFF, Lehrbuch der Psychiatrie (1956), S. 328ff.

Patienten in der Datenbank mit einer Alkoholismus-Diagnose und Malariafiebertherapie, die alle sehr jung waren,[549] hatten 13 Patienten auch die Diagnose Psychopathie.

In den eingesehenen Publikationen der Wiener Kliniker der Zeit konnte kein Hinweis auf eine Indikation der Malariafiebertherapie bei Alkoholismus gefunden werden. Die Publikationen der beiden Wiener Alkoholismus-Experten der Klinik, Wilhelm Solms (für die 1950er Jahre[550]) und Kornelius Kryspin-Exner (für die 1960er Jahre[551]) konzentrieren sich vor allem auf psychotherapeutische Maßnahmen als „ursächliche Therapie",[552] besonders auf die Gruppentherapie. Bei Kryspin-Exner, der bereits auf die Möglichkeiten und Erfahrungen im 1961 eröffneten *Genesungsheim Kalksburg* zurückgreifen konnte, war es auch die Arbeitstherapie in der Gruppe.[553] Diese „zweckbezogene Gemeinschaftsarbeit" und das (u. a. damit) „durch das spezifische Anstaltsmilieu erzeugte, besonders intensive Gemeinschaftsgefühl"[554] wurden von Kryspin-Exner als besonders wichtig für den Erfolg bezeichnet.

Diese psycho- und arbeitstherapeutischen Maßnahmen sollten an der Klinik mit der Malariafiebertherapie – wie es in einzelnen Krankenakten heißt – ‚vorbehandelt' bzw. ‚vorbereitet' werden. Von den bisher erwähnten erwünschten Wirkungen der Fieberkur bei Patienten mit nichtluetischen Erkrankungen – Beruhigung, Umstimmung, Reifung, Krankheitseinsicht, Öffnung des / der Patienten/in für das Gespräch mit dem / der Psychotherapeuten/in, aber auch Öffnung zur Integration in das soziale Umfeld – dürften bei den jungen Alkoholikern alle als Motiv für die Fieberkur in Frage kommen, vor allem aber die Ermöglichung des psychotherapeutischen Gesprächs und der sozialen Anpassung (Einbindung): Durch die intensive Betreuung des / der in den schweren Fieberschüben der

---

[549] Die 24 Patienten waren im Alter von 14 bis 27 Jahren (Median 21 Jahre), der älteste der 13 Psychopathie- und Alkoholismus-Patienten 23jährig (die meisten unter 21 – Median 19 Jahre). Ältere Alkoholkranke bekamen vor ihrem Entzug in *Kalksburg* an der Klinik sedative Medikamente und Antidepressva: Der 39jährige Patient, der im April 1968 freiwillig mit der Diagnose „Alk chron." aufgenommen wurde, bekam, bevor er am 7. Tag nach *Kalksburg* entlassen wurde, am 1. Tag Distraneurin (Clomethiazol), ein „Sedativum, das im Rahmen des Alkoholentzugs eingesetzt wird" (http://flexikon.doccheck.com/de/Clomethiazol: 4.12.2017), dann tgl. 3x 10mg Valium.
[550] Vgl. SOLMS, Moderne Therapie des Alkoholismus (1960), passim.
[551] Kornelius KRYSPIN-EXNER habilitierte 1966 in Wien mit der Arbeit: Psychosen und Prozessverläufe des Alkoholismus.
[552] Vgl. SCHILLER/SOLMS, Neue Methoden in der Behandlung des chronischen Alkoholismus (1949), S. 539: „Wir sind mit […] vielen anderen der Ansicht, daß der Alkoholismus nur ein Symptom ist. Eine ursächliche Therapie […] kann in den meisten Fällen nur eine psychotherapeutische sein."
[553] KRYSPIN-EXNER. Probleme der Bekämpfung des Alkoholismus in Österreich (1965), S. 646,
[554] KRYSPIN-EXNER, Die moderne Behandlung des Alkoholkranken (1963), S. 193f. Vgl. KRYSPIN-EXNER, Probleme der Führung einer offenen Anstalt für Alkoholkranke (1966), passim.

‚Erschütterungstherapie' hilfsbedürftigen Patienten/in wurde nach dieser Hypothese Kommunikation und Vertrauen zum medizinischen Personal aufgebaut, die die Voraussetzung für die Psychotherapie und die soziale Öffnung bildeten.

Beides stellt sich nicht nur bei dieser Patientengruppe, sondern auch für andere jugendliche PatientInnen mit Malariafiebertherapie und einer Diagnose außerhalb der Neurolues. Wie bei allen diesen Patientengruppen widerspricht dieser Hypothese, dass bei jungen Patienten mit gleichen Diagnosen – Alkoholismus, oft auch zusätzlich mit der Diagnose „Psychopathie" und mit dem Vermerk „SMV" – keine Fiebertherapie gegeben wurde. Manchmal könnten zwar unterschiedliche Charakterisierungen der Patienten in der Anamnese (z.B. Erwähnung bzw. Nichterwähnung von Aggressivität, oder fehlende Krankheitseinsicht als Hindernis für die Entwöhnungstherapie) zu unterschiedlichen Therapien geführt haben. Die individuelle Analyse des Patienten wurde mehrfach als notwendige Voraussetzung für die Erstellung des „Gesamtbehandlungsplans" betont. Aus Mangel an eindeutigen Parallelbeispielen und an klärenden Aussagen der Kliniker lässt sich daraus keine Regelmäßigkeit in den Entscheidungsgrundlagen ableiten. Nach der geforderten individuellen Beurteilung des Patienten / der Patientin, der Symptome und des Verlaufs der Krankheit, und der aus dieser Beurteilung aufgrund der persönlichen Erfahrung des behandelnden Arztes getroffenen Entscheidung für das therapeutische Vorgehen – Manfred Bleuler rechtfertigt diese unterschiedlichen Entscheidungen[555] – scheinen diese Sonderfälle, aus denen sich keine Systematik der Therapie ergibt, aber auch wieder an Plausibilität zu gewinnen.

Außerdem ist auch in den Fällen der Anwendung der Malariafiebertherapie bei Patienten mit Alkoholproblemen eine Anwendung vordringlich, um den *Malaria tertiana* Stamm zu erhalten, nicht völlig auszuschließen. Ein Argument für die Anwendung in dieser Funktion sind Anmerkungen – vergleichbar mit den im folgenden Exkurs zur Stammträger-Frage genannten – in den Akten von zwei Patienten:

> Der 21-jährige Patient P820 mit der Diagnose "Alk. chron., Psychopathie"[556] hatte sich 1967 auf Anraten seines Hausarztes wegen seines Alkoholproblems freiwillig an der Klinik aufnehmen lassen und unterschrieb schon am Tag der Ankunft den Vordruck zur freiwilligen Aufnahme, auf dem es in der Zeile „Kurze Anamnese" heißt: „als

[555] Vgl. das Zitat oben S. 33 zum Wirrwarr der Meinungen.
[556] Laut Anamnese habe er „mit [der] Polizei wegen [eines] Mopeddiebstahls, Körperverletzung und Randalierens zu tun gehabt".

Stammträger vorgesehen“.[557] Sein infiziertes Blut wurde weitergegeben.[558] Für eine therapeutische Indikation in Vorbereitung eines Entzugs im Sinn der oben genannten Hypothese spricht, dass er in der Anamnese als „etwas distanzlos, keine echte Einsicht, wenig kooperativ“ und damit als noch ungeeignet für eine Psychotherapie beschrieben wurde, und dass er, nachdem er eine Malariakur bekommen hatte, nach *Kalksburg* entlassen wurde. Ob also auf eine nicht-therapeutische Anwendung[559] oder auf das Gegenteil geschlossen werden kann, bleibt – wie bei bereits genannten ähnlichen Fällen – offen. So auch im folgenden vergleichbaren Fall.

Der Patient P380 wurde als 25-jähriger 1953 mit der Diagnose „Psychopathie, Alc. chron. (Antabusrückfall)“ zum zweiten Mal an der Klinik aufgenommen. Er gab an, nach seiner Scheidung wieder mit dem Trinken begonnen[560] und einen Suizidversuch unternommen zu haben. Während der Befragung durch den Arzt zeigte sich der Patient laut Anamnese wenig einsichtig. Er „entrüstet sich mehrmals, es könne ihm doch niemand das Trinken verbieten, er habe niemanden damit geschädigt. Zeigt sich auf verschiedene Vorhalte, die Folgen seiner Trunksucht und seinen SMV betreffend, völlig uneinsichtig.“ *Decursus*-Eintrag am dritten Tag: „Pat wird als Plasmodienstammträger mit 4 ccm Malariablut i. v. geimpft.“ Diese Bemerkung könnte ein Hinweis auf eine Vordringlichkeit der Stammträger-Funktion sein, und sein malariainfiziertes Blut wurde auch an drei Patienten – in deren Fieberkurve wie üblich der Spender namentlich genannt wird – weitergegeben. Es ist hier aber nicht auszuschließen, dass am *Steinhof*, wohin der Patient nach der Fieberkur gebracht wurde, noch einmal eine Entwöhnungstherapie[561] versucht wurde und die ‚Erschütterungstherapie‘ der fehlenden Krankheitseinsicht galt, die als Voraussetzung für einen Erfolg des Entzugs angesehen wurde.

2.1.4.3 Die Weitergabe des mit *Malaria tertiana* Plasmodien infizierten luesfreien Blutes und der Begriff „Stammträger“ und seine Bedeutung in den PatientInnenakten

Der Begriff „StammträgerIn“ bezeichnet einen Pateinten / eine Patientin, der / die als „SpenderIn“ während eines Fieberschubes das malariainfizierte Blut durch direkte Übertragung an einen / eine weitere Malariatherapie-Patienten/in weitergab. Die Übertragung erfolgte an der „Klinik Hoff“ meistens bei der letzten Fieberzacke vor der ersten Chinin-

[557] Dieses Formular liegt den PatientInnenakten nur manchmal bei; immer jedoch wurde der Hinweis auf eine freiwillige Aufnahme – wie auch eine Änderung von polizeiärztlicher Einweisung auf freiwillige Aufnahme – am Deckblatt eingetragen (häufig mit Stempel).
[558] Nur bei einem der Patienten dieser Gruppe ist aus den Akten die Weitergabe des malariainfizierten Blutes nicht nachzuweisen.
[559] Gegen eine hohe Dringlichkeit, einen ‚Stammträger‘ zu finden, spricht in diesem Fall auch, dass es nachweislich über drei Monate, d.h. mit sechs Übertragungen parallel einen zweiten Stamm gab, also gleichzeitig andere Patienten an der Klinik waren, von denen malariainfiziertes Blut übertragen werden konnte.
[560] Bei seiner ersten Aufnahme im Jahr zuvor hatte er eine Entwöhnungskur mit Antabus-Unterstützung gemacht.
[561] Bei der dritten und letzten Aufnahme 1956 mit der Diagnose „Psychopathie, Alc. Chron., SM-Versuch im Rausch“ wurde er laut AK-interner Anfragebeantwortung am zweiten Tag „zur Durchführung einer Entziehungskur in die Lds. H. u. Pflg. Anstalt ‚Am Steinhof‘ überstellt.“ Zu dieser Zeit gab es das Therapiezentrum in *Kalksburg* noch nicht.

Gabe, im Regelfall sogleich und direkt nach der Abnahme.[562] Damit wurde nicht nur die Therapie mit starken Fieberschüben über 40°, in seltenen Fällen und unerwünscht auch deutlich über 41° begonnen, sondern auch im / in der neu infizierten Patienten/in der *Malaria tertiana* Stamm, der außerhalb des Körpers nur etwa drei Tage haltbar ist,[563] für eventuelle weitere Übertragungen erhalten. (Ausführlich wird der Ablauf der Malariafiebertherapie oben im Unterkapitel 2.1.2 beschrieben.)

Der Name des / der Spenders/in wurden am Tag der Impfung des mit *Malaria tertiana* Plasmodien infizierten-Blutes auf der Fieberkurve des / der Empfängers/in genannt. Es finden sich jedoch in den eingesehenen Akten 127 Namen von SpenderInnen, deren Akten im Untersuchungszeitraum nicht archiviert und deshalb nicht in der Datenbank aufgenommen sind.[564] Das heißt es gab an der „Klinik Hoff" deutlich mehr Malariafiebertherapien.[565]

Es gab 1959/60 drei größere Lücken in der Weitergabe des mit *Malaria tertiana plasmodium vivax* infizierten luesfreien Blutes (in der Kette der Übertragungen),[566] die nur durch die Anforderung von mit Malaria-Plasmodien infiziertem luesfreiem Blut von anderswo (Tropeninstituten) behoben werden konnten:[567]

---

[562] In Einzelfällen dürfte aber auch das mit *Malaria tertiana* Plasmodien infizierte Blut abgenommen und erst wenige Tage später geimpft worden sein: So wurde Anfang 1952 das mit *Malaria tertiana* infizierte Blut des 52-jährigen Patienten N32 („Alte p.P.") drei Tage nach der ersten Chinin-Gabe an den Patienten N37 („P.P.") weitergegeben. Erwähnenswert ist nicht nur diese Verzögerung und die Weitergabe von nicht-luesfreiem Blut (zur Seltenheit vgl. oben S. 66 Anm. 203), sondern auch, dass das malariainfizierte Blut des Patienten übertragen wurde, nachdem die Fiebertherapie nach vier Fieberschüben mit dreimal Typhus Vakzine- und einmal Pyrifer-Provokation abgebrochen worden war.
[563] Zur Haltbarkeit lt. Wagner-Jauregg vgl. oben S. 54 Anm. 159.
[564] 12,75% von 996 – der Zahl, die sich aus 869 PatientInnen mit Malariafiebertherapie, deren Akten 1951-1969 archiviert und in die Datenbank aufgenommen wurden, und 127 PatientInnen, die nur im Akt des / der Empfängers/in genannt werden ergibt. Vgl. zu den möglichen Gründen oben S. 57f.
[565] Grob geschätzt ca. 200 PatientInnen bzw. über 20%. Stichproben zeigten, dass die Akten mehrerer dieser SpenderInnen mit einer späteren Aufnahme (nach 1969) archiviert sind. Es ist möglich, dass einzelne ‚Spender' an anderen Krankenanstalten (in Wien) behandelt und das Malariablut von dort den PatientInnen an der Klinik gegeben wurde (vgl. oben S. 57 Anm. 177 und S. 79 Anm. 264). Auch in diesen Fällen ist jedoch wahrscheinlich, dass es sich um den Stamm der Klinik und nicht um einen eigenen Stamm des betreffenden Krankenhauses handelte. Hinweise zeigen, dass malariainfiziertes Blut von der Wiener Klinik bezogen wurde (vgl. oben S. 54 Anm. 160 zu Graz und München), wie bereits Mitte der 1920er Jahre: vgl. Kundratitz, zitiert in GRÖGER, Röntgen- und Malariatherapie. Zur Therapie des kindlichen Schwachsinns (2019), S. 167.
[566] Andeutungen von Peter Berner in Gesprächen mit Gernot Heiss (Paris, 14.5.1912) und mit Eberhard Gabriel lassen vermuten, dass Assistenten die Malariatherapie nun auslaufen lassen wollten. Hoff wollte jedoch die Therapie weiter anwenden – nicht nur bei den zunehmend seltenen Fällen von progressiver Paralyse, sondern auch bei nicht-luetischen Erkrankungen. Vgl. unten S. 171 zu Peter Berners Einschätzung von Hoffs „Verwurzelung in organische Krankheitskonzepte".
[567] Von den drei Lücken gibt jeweils das erste Datum an, wann der letzte Spender luesfreies malariainfiziertes Blut an einen Patienten / eine Patientin weitergegeben hat, das Enddatum der Lücke, wann das Blut aus dem Tropeninstitut dem neuen Patienten / der neuen Patientin geimpft wurde. Das Datum zu Beginn der Lücke ist

--- 25. November 1958 – 12. Februar 1959: die Wiener Klinik bekam für eine Impfung Malariablut vom Tropeninstitut Hamburg.

--- 6. Juni 1959 – 19. Oktober 1959: die Wiener Klinik bekam für eine Impfung Malariablut vom Tropeninstitut Hamburg.

---19. Oktober 1959[568] - 29. Jänner /5. Februar 1960: die Wiener Klinik bekam für drei Impfungen (1mal mit *Malaria quartana*,[569] 2mal mit *Malaria tertiana*) Malariablut vom Tropeninstitut Amsterdam.

Nur ein Teil der PatientInnen, die eine Malariafiebertherapie bekamen, waren „StammträgerInnen“ und gaben ihr malariainfiziertes Blut weiter:

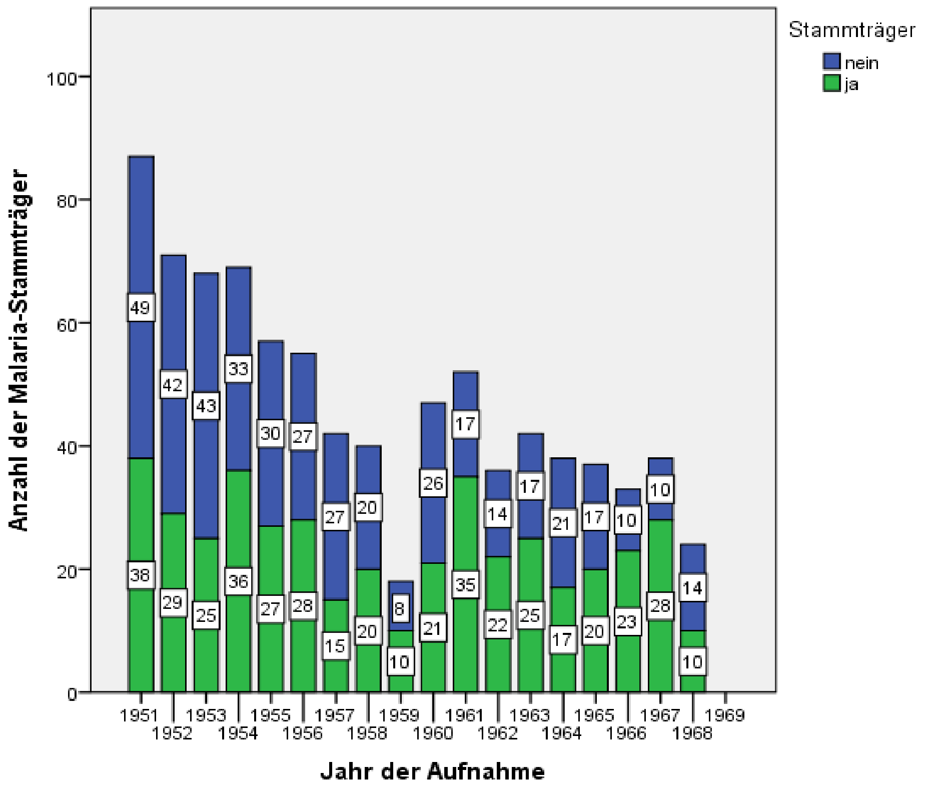

Abb. 7 Stammträger und Nichtstammträger, 1951-1969, n=869.[570] [*Auf der linken Seite der Tabelle müsste es heißen: Anzahl der PatientInnen mit Malariafiebertherapie*.]

---

insofern unsicher, als das Fehlen eines weiteren Empfängers durch die Unvollständigkeit der Akten bedingt sein könnte.

568 Der Patient, der am 19.10.1959 das (zweite) Blut vom Tropeninstitut Hamburg bekam, war nicht Spender, sodass hier sogleich wieder eine Lücke entstand.

569 Der Patient, der am 29.1.1960 mit einer *Malaria quartana* vom Tropeninstitut Amsterdam geimpft wurde, war nicht Spender.

570 Alle aufgenommenen Fälle mit Malariatherapie, nicht nur jene mit den fünf Diagnosen.

Der Anteil der StammträgerInnen (PST) an den PatientInnen mit Malariafiebertherapie ist in den 1960er Jahren höher, da viel weniger PatientInnen mit progressiver Paralyse aufgenommen wurden, die nur in Einzelfällen ihr malariainfiziertes Blut weitergaben, also fast immer als Nicht-Stammträger zu zählen waren.

Frauen bekamen ohne Neurolues sehr selten eine Malariafiebertherapie. In der Datenbank finden sich nur 13 Patientinnen ohne Neurolues mit einer Malariakur, davon waren sechs Spenderinnen, alle in den 1950er Jahren: vier 1951, eine 1953 und eine 1959. Die anderen sieben – die letzten zwei Anfang der 1960er Jahre – gaben ihr malariainfiziertes Blut nicht weiter.

Der Hypothese, die Malariafiebertherapie sei bei Diagnosen außerhalb der Neurolues insgesamt nur zum Erhalt des Stamms, also ohne therapeutische Absicht gegeben worden,[571] widersprechen die deutlichen Anteile von Nicht-StammträgerInnen bei Malariafiebertherapie-PatientInnen mit nichtluetischen Erkrankungen, d. h. von Anwendungen bei PatientInnen ohne Neurolues, von denen das malariainfizierte Blut nicht weitergegeben wurde. Vermerke im Krankenakt, wie sie im folgenden Exkurs zum Begriff „Stammträger“ erklärt werden, fehlten in ihrem Krankenakt oder waren durchgestrichen, sie war also für eine Übertragung auch nicht vorgesehen. Weiters spricht gegen diese Hypothese, dass diese Anwendung außerhalb der Neurolues nach der Einführung der Penicillinkur und den verschwindenden Zahlen der PatientInnen mit Neurolues[572] in den 1960er Jahren nur der einzige ersichtliche Grund für die Aufrechterhaltung, die „Pflege“ des Stammes sein konnte.

Im folgenden Balkendiagramm zur Anzahl der StammträgerInnen nach den Diagnosegruppen wurden drei Gruppen (Alkohol, Neurose, Verwahrlosung) und eine Kategorie für nicht klassifizierbare Diagnosen eingefügt, die nicht den Einschlusskriterien des Projekts und auch nicht diesen drei Gruppen entsprechen und von denen nur die Fälle mit Malariafiebertherapie in die Datenbank aufgenommen sind:

---

[571] Vgl. unten S. 159f.
[572] Vgl. Abb. 2 und Text auf S. 74.

	PST[573]	Keine PST	Gesamt
	Anzahl (Fälle)	Anzahl (Fälle)	Anzahl (Fälle)
Intelligenzmängel	163	89	252
Neurolues	3	173	176
Schizophrene Erkr.	121	78	199
Psychopathie	115	85	200
Affektive Erkr.	57	52	109
Alkoholismus*	0	3	3
Neurotische Erkr.*	41	13	54
"Verwahrlosung"*	2	3	5
Diagnose nicht klassifizierbar	26	24	44
Gesamt (Akten)	429	440	869

Tab. 13 Stammträger/Nicht-Stammträger und Diagnosen, 1961-1969, n = 869, **Mehrfachnennungen bei Diagnosen sind möglich**.

Es scheint keine PatientInnen mit einer bestimmten Diagnose zu geben, die sich speziell als StammträgerInnen eigneten bzw. nicht eigneten – mit Ausnahme der Neurolues: Nach der Datenbank wurden in Wien nur drei (männliche) Patienten mit progressiver Paralyse als Stammträger verwendet, die freilich auch nur an Patienten weitergegeben haben, die ebenfalls eine progressive Paralyse hatten. Zwei der Patienten waren im Jahr 1951, einer im Jahr 1954 an der Klinik.

Aufgrund der Hinweise in den Patientenakten von 28 Patienten[574], die lt. Datenbank von der *Heil- und Pflegeanstalt Steinhof* (*Baumgartner Höhe*) zur Malariatherapie an die Klinik (meistens rück-)überwiesen und danach wieder zurückgebracht wurden, soll im Folgenden versucht werden, Fragen zur Weitergabe, Erhaltung bzw. „Pflege" des *Malaria tertiana* Stamms (des malariainfizierten Blutes) durch Überimpfung zu klären. Die Akten dieser 28 Patienten wurden gewählt, da in einigen auffällige Bemerkungen zu finden sind, die in ihren Fällen die Hypothese von einer nicht-therapeutischen Anwendung der Malariafiebertherapie zu stützen scheinen.

[573] PST = Plasmodien-Stammträger.
[574] Die Malariapatienten in dieser im Folgenden besprochenen Gruppe sind ausschließlich männlich.

Organisatorisch war vorgesehen, dass die psychiatrische Universitätsklinik als ‚Clearingstelle' die meisten der psychiatrischen PatientInnen in den ersten Tagen nach der Aufnahme und Begutachtung weiter überweist an psychiatrische Krankenhäuser und hier vor allem an die *Heil- und Pflegeanstalt Steinhof*.[575] Auch viele der an der Klinik stationär länger als vier Tage behandelten PatientInnen wurden nach der Malariafiebertherapie auf den *Steinhof* überstellt; es waren jene Fälle, die stationär weiter betreut werden sollten. Vom *Steinhof* wurden PatientInnen an die Klinik als ‚in die Vorlesung gebetene Patientin / gebetener Patient' – so die damalige Formulierung – gebracht. Die in diesem Exkurs besprochenen Patienten kamen vom *Steinhof* „beurlaubt" nur für die Malariafiebertherapie und wurden (wohl aus Platzmangel) bereits zu Beginn der siebentägigen Chinin-Behandlung auf den *Steinhof* zurückgebracht.

Die im Folgenden besprochenen 28 Fälle betreffen Patienten, die vom *Steinhof* ohne Neurolues zu einer Malariafiebertherapie an die Klinik kamen und deren infiziertes Blut weitergegeben wurde, die also „Stammträger" waren. In einigen der Akten dieser Patientengruppe finden sich neben den üblichen Vermerken, die den Patienten als Stammträger kennzeichnen (wie PST für Plasmodium-Stammträger am Rand ihrer Fieberkurven), auch solche, die in Bezug auf ihre Positionierung und Wortwahl auffallen. Die Hinweise zu ihrer Funktion als Stammträger sind in drei Kategorien zu unterteilen:

A) Die Nennung der Stammträger-Funktion am Titelbogen in der Zeile des Vordrucks „12. Psychiatrische Diagnose", wenn auch neben einer oder mehreren Diagnosehinweisen, ist wohl nicht am Platz: hier sollte vor allem die Diagnose als Ergebnis der Befundung angegeben werden; häufig finden sich in der Diagnosezeile zusätzlich Einträge, die auf Symptome bzw. Handlungen des / der Patienten/in hinweisen (wie z. B. „SMV"), die für die Indikation relevant sein konnten. Bei den folgenden Patienten scheinen die Formulierungen jedoch auf eine nicht oder bestenfalls nachgereihte therapeutische Anwendung der Malariafiebertherapie und eine Vordringlichkeit der Stammträgerfunktion zu verweisen. Es sind Hinweise zur Organisation, um den Plasmodienstamm zu erhalten. So heißt es:

- 1951 beim 24jährigen Patienten S232 in der Diagnosezeile „Schizo (Malariastammhalter)",

[575] Vgl. zur Funktion als ‚Clearingstelle' oben S. 14.

- 1952 bei seiner 4. Aufnahme beim 29-jährigen Patienten *S235 „Pfropfschizo, Stammhalter“,[576]
- 1952 beim 18jährigen Patienten I239 „Imbezillität, PST“,
- 1952 bei seiner 5. Aufnahme beim 26-jährigen Patienten *S243 „Schizophrenie, PST“,
- 1952 bei seiner 2. Aufnahme beim 37-jährigen Patienten *S247, „Schizophrenie (Stammhalter)“,
- 1952 bei seiner einzigen Aufnahme beim 23-jährigen Patienten I248 „Idiotie, PST“.[577]

B) Bei 15 Patienten dieser Gruppe von 28, die vom *Steinhof* an die Klinik zur Malariafiebertherapie gebracht wurden, finden sich in der Anamnese[578] Formulierungen, die in ähnlicher Form den Schluss auf eine nicht oder nur nachrangig therapeutisch motivierte Anwendung nahelegen. Bei drei der bereits genannten sechs Patienten, die mit * gekennzeichnet sind, sind gleichbedeutende Formulierungen in der Diagnosezeile und in der Anamnese zu finden. Es sind Formulierungen wie „als Stammträger von Steinhof“, die auf die Funktion des Patienten als Malariablut-Erhalter und -Spender hinweisen und diesen Hinweis gleichsam als Begründung für die Aufnahme und Therapie anzugeben scheinen. So heißt es zu den drei bereits in Bezug auf den Stammträger-Hinweis in der Diagnosezeile erwähnten Patienten:

- 1952 beim oben erwähnten Patienten *S247 in der Anamnese: „Pat[ient] wurde vom Steinhof zu uns überstellt, um hier als Stammträger zu dienen“,
- 1952 beim Patienten *S235 „Pat[ient]wird als Stammträger von Steinhof rücktransferiert“,
- 1952 beim Patienten *S243 „Pat wurde als Malariastammträger von Steinhof rückverlegt“.

Formulierungen, die in ähnlicher Wortwahl eine Vordringlichkeit der Stammträger-Funktion für die Anwendung der Malariafiebertherapie suggerieren, finden sich in den Anamnesen von 12 weiteren Patienten der Steinhofgruppe,[579] von denen hier nur beispielhaft jene von vier Patienten erwähnt werden. So heißt es:

- 1952 zum bei seiner zweiten und letzten Aufnahme[580] 21-jährigen Patienten I249 mit der Diagnose „Imbezillität“ in der A[581]namnese: „Pat. wurde [gestern] vom Steinhof als PST an

[576] Lt. Eintrag in seinem Krankenakt am *Steinhof* (WStLA, M. Abt. 209, Otto-Wagner-Spital, A 12/4) vom 10.1.1952: „Pat. wird mit dem heutigen Tage als Plasmodienträger in die Klinik beurlaubt.“ Vgl. zu ihm auch unten S. 157.
[577] Sein malariainfiziertes Blut wurde lt. Datenbank nicht übertragen. Trotz Typhus-Vakzine-Provokation fieberte er nicht, was ein Grund dafür gewesen sein könnte, die Übertragung jedoch nicht ausschloss; außerdem bestand parallel eine andere Stammträgerkette.
[578] Die Anamnese wurde vor dem Eintrag auf dem Deckblatt in der Diagnosezeile und üblich von einem anderen Arzt / einer anderen Ärztin als dieser Eintrag aufgenommen, hatte freilich Einfluss auf die Diagnoseerstellung. Vgl. REITER/GABRIEL, Diagnose „Psychopathie“ und diagnostischer Prozeß bei Jugendlichen (1973), S. 137: „Der diagnostische Prozeß wird auch durch ‚institutionelle‘ Faktoren“ beeinflusst.
[579] Zu diesen gehört auch der Patient S636, dessen Fall oben S. 89 beschrieben wurde.
[580] Seine 1. Aufnahme war 1947.
[581] Lt. Eintrag in seinem Krankenakt am *Steinhof* (WStLA, M. Abt. 209, Otto-Wagner-Spital, A 12/4) vom 10.1.1952: „Pat. wird mit dem heutigen Tage als Plasmodienträger in die Klinik beurlaubt.“ Vgl. zu ihm auch unten S. 157.

unsere Klinik gebracht, er wurde bereits mit 4ccm Malariablut inoculiert“ – sonst steht nichts in der Anamnese,
- 1962 zum bei seiner dritten Aufnahme 17-jährigen Patienten I682 mit der Diagnose „Oligophrenie, Kurzschlussreaktion“: „Der Patient wurde vom Steinhof für PST an die Klinik transferiert“,
- 1964 zum bei seinem dritten Aufenthalt 22-jährigen Patienten I839 mit der Diagnose „Oligophrenie“ heißt es auf der Fieberkurve (wofür das gleiche gilt wie für ähnliche Hinweise in der Anamnese) – hier unüblich ausführlich: „Der Patient kommt gestern vom psychiatrischen Krankenhaus [am *Steinhof*] als Stammträger an unsere Klinik. Patient wurde gestern mit Malaria geimpft und wird als Stammhalter einige Zeit an unserer Klinik bleiben“;
- 1968 zum bei seinem einzigen Aufenthalt 18-jährigen Patienten I843 mit der Diagnose „(Oligophrenie / Notzuchtdelikt)“: „Der Patient wird uns vom psychiatrischen Krankenhaus der Stadt Wien als Stammträger überstellt.“

Im Fall des oben genannten 29jährigen Patienten *S235, der 1952 zum vierten Mal aufgenommen wurde (in der Diagnose-Zeile „Pfropfschizo, Stammhalter“) und dessen Fall in der beiliegenden Krankengeschichte als „Pfropfhebephrenie, Zustand nach Lobotomie. Endzustand“ beschrieben wurde, scheint der Bezug der Indikation auf die Diagnose noch zusätzlich erklärungsbedürftig:[582] Denn die Lobotomie war auch an der Wiener Klinik als *ultima ratio* vorgesehen, nachdem alle anderen Behandlungen erfolglos geblieben waren.[583] Dieses Argument ist jedoch zu relativieren, da Raoul Schindler in dem von Hoff 1960 herausgegebenen Sammelband dazu schreibt, es zeige sich oft nach einer Lobotomie, dass danach „eine erneute Ansprechbarkeit auf die geläufigen Schocktherapien eintritt.“[584] Auch der Patient S656 erhielt als 21-jähriger 1953 bei seinem vierten Aufenthalt an der Klinik mit der Diagnose „Hebephrenie, Zustand nach Lobotomie“ eine Malariafiebertherapie.[585] In Wien, wie auch anderswo, wurde auch die Malariakur selbst als *ultima ratio* in Erwägung gezogen.[586]

Auffallend ist, dass die meisten dieser insgesamt 18 Patienten der beiden Gruppen A und B aus 1951/2 (elf Fälle) und dann verteilt über die Jahre 1960 bis 1964 (sechs Fälle) stammen

---

[582] Er wurde 1951 von der „Heil. Anstalt Ybbs a. D. für Lobotomie-Behandlung“ an die Klinik überwiesen. In einer Krankengeschichte wurde zu den früheren Aufenthalten seit 1946 über heftige Erregungszustände, Aggressivität und Selbstbeschädigungstendenzen berichtet; nach der Lobotomie sei er „wesentlich ruhiger, stumpf, autistisch, gut zu führen“. Nach vier Fieberschüben und sieben Chinintagen kam er zurück auf den *Steinhof*.

[583] Vgl. den Wiener Kliniker Raoul SCHINDLER, Fortschritte der Psychochirurgie (1960), S. 474. Zur Lobotomie vgl. auch S. 221-224 im Kapitel zur Insulinkomatherapie.

[584] SCHINDLER, Fortschritte der Psychochirurgie (1960), S. 474; vgl. HOFF, Lehrbuch der Psychiatrie (1956), S. 512, wo er von einer meistens besseren „Ansprechbarkeit auf ES […] nach dem psychochirurgischen Eingriff“ schreibt.

[585] Zu ihm vgl. unten S. 215 Anm. 838 und S. 223.

[586] Zur Malariatherapie bei Schizophrenie als *ultima ratio* vgl. BLEULER, Lehrbuch der Psychiatrie ([10]1960), S. 405 zit. oben S. 31 Anm. 93. Vgl. auch die Erfahrungen des Zeitzeuge an der Salpêtrière Anfang der 1960er Jahre (oben S. 81f.).

und der 18. dieser Patienten erst 1968 eine Fiebertherapie bekam. Aus den vorliegenden Quellen bleibt jedoch offen, wie diese zeitliche Konzentrationen bzw. Unterbrechungen zu interpretieren sind.

C) An einigen der zehn Patienten, die keiner der beiden Gruppen zugezählt wurden, soll der Unterschied gezeigt werden, der in diesen Fällen durch die Begriffswahl und ihre Platzierung nicht auf eine Vordringlichkeit, den Malariastamm zu erhalten, schließen lässt: Sie hatten den PST-Hinweis nur als Vermerk auf der Fieberkurve, der – meistens mit rotem Stift – am Rand der Fieberkurven aller PatientInnen angebracht wurde, denen als SpenderIn während der Fieberschübe und vor der Gabe von Chinin malariainfiziertes Blut abgenommen werden sollte. D.h. letzteres wurde bei allen PatientInnen eingetragen, die eine Malariatherapie bekamen und die auch als SpenderInnen vorgesehen waren.[587]

Sechs Patienten hatten zwar bereits in der Anamnese nach ihrer Ankunft vom *Steinhof* einen Hinweis auf die Indikation einer Malariakur, aber nicht auf eine Stammträger-Funktion. So hieß es

- 1951 bei seiner 2. Aufnahme zum 20-jährigen Patienten P233 mit der Diagnose „Schwachsinniger Psychopath“: „vom Steinhof rücktransferiert, zur Durchführung einer Malariakur“. Er war nach einer Woche an der Klinik zwei Wochen am *Steinhof* gewesen und die Anamnese ist deshalb sehr kurz. In seinem Fall dürfte die Rücküberweisung zur Fieberkur bereits bei der Überweisung auf den *Steinhof* geplant gewesen sein. Die Formulierung „rücktransferiert“, in anderen Fällen „rückverlegt“, könnte auf eine Erstüberweisung auf den *Steinhof* aus Platzmangel schließen lassen.

- 1961 bei seiner zweiten und letzten Aufnahme zum nun 27-jährigen Patienten I553 mit der Diagnose „Oligophrenie“: „Patient kommt zwecks Durchführung einer Fieberkur von der Heil- und Pflegeanstalt Steinhof an die Klinik“. In der Krankengeschichte vom *Steinhof* steht zu seiner Überweisung: „Patient wird an die psychiatrische Klinik zwecks einer Malariatherapie […] beurlaubt.“[588]

Begründungen der Indikation fehlen, wie auch in den PatientInnenakten bei anderen Therapien. Zu einem Patienten dieser Gruppe – und diese Bemerkung kam von außen – gibt es einen Hinweis, dass die Malariatherapie bei „Psychopathie“ zur „Beruhigung“ zu Beginn einer (zuerst stationären) Therapie gegeben wurde:

> Dem Patientenakt P449 („Psychopathie“) zur dritten Aufnahme des nun 18jährigen Patienten im Jahr 1958 liegt die Begründung des Bezirksgerichts Wien Innere Stadt für

[587] Auch der Eintrag „PST“ allein auf der Anfrage an den Internisten ist nicht als Hinweis auf eine nichttherapeutische Anwendung zu interpretieren, denn die Freigabe zur Malariakur war nicht zugleich eine Freigabe als Spender, anders umgekehrt.
[588] WStLA, M. Abt. 209, Otto-Wagner-Spital, A12/9.

> eine weitere Anhaltung in einer geschlossenen Heilanstalt bei, in der es heißt: „Nach Vorgeschichte und erhobenen Befunden handelt es sich um einen jugendlichen Psychopathen mit verschiedenen Aggressionstendenzen gegen Angehörige und Umwelt. Im Verlaufe der Fieberkur ist bereits eine merkliche Beruhigung eingetreten, doch wird Fortsetzung der stationären Therapie an der Klinik noch für einige Zeit notwendig sein.“ In der Anamnese hieß es: „Pat[ient] wurde […] mit der D[ia]g[nose] Psychopathie auf den Steinhof transferiert, gestern zu einer Fieberkur (~~PST)~~ rücktransferiert.“ Aus der Streichung des PST-Vermerks dürfte zu schließen sein, dass er aufgrund einer Entscheidung im Verlauf seines Aufenthaltes auch gar nicht mehr als Stammträger vorgesehen war. In der Datenbank scheint er auch nicht als ‚Spender’ auf.

Resümierend ist zu bemerken, dass bei 18 von 28 Patienten, von denen einige beispielhaft unter A) und B) beschrieben wurden, die Vermerke in der Diagnosezeile am Deckblatt und / oder in der Anamnese die Anwendung der Malariafieberkur vordringlich mit der Funktion als „Stammträger“ zu begründen scheinen, also mit der Sicherung des mit *Malaria tertiana* Parasiten infizierten Blutes durch regelmäßige Überimpfung. Dass es sich dabei lt. Krankenakt der *Baumgartner Höhe* um befristete ‚Beurlaubungen‘ (nach Klinikakten um ‚Rücküberweisungen‘) nur zur Fiebertherapie handelte, könnte auf eine bewusste Verzögerung der Therapie bis zum Bedarf eines Stammträgers schließen lassen – auf eine Regelung, die zur Aufrechterhaltung der Kette der Blutspender notwendig war. So wäre auch verständlich, warum nun nur die Funktion als Stammträger genannt wurde. Gegen die Hypothese, die Stammträgerfunktion sei der einzige Grund für die Indikation gewesen, spricht, dass diese Patienten Diagnosen hatten, die mehrfach zur Malariafiebertherapie führten, und ein beachtlicher Teil dieser PatientInnen nicht als StammträgerInnen vorgesehen war.[589] Eine Anwendung der Malariafiebertherapie ohne therapeutisches Ziel im Einzelfall ist freilich nicht auszuschließen, wohl auch wahrscheinlich, allerdings im konkreten Fall kaum nachzuweisen.

Bei den Patienten, die vom *Steinhof* (*Baumgartner Höhe*) zur Malariatherapie an die Klinik kamen, kann die Kürze der meisten Anamnesen und der Hinweis in einigen Fällen „kommt vom Steinhof zur Malariatherapie“ auf Absprachen der Ärzte/innen der Klinik und des mit dieser eng verbundenen Wiener städtischen psychiatrischen Krankenhauses zurückzuführen sein. Um die Frage zu klären, warum gerade diese Patienten unter den vielen mit den gleichen Diagnosen die Fiebertherapie bekamen, sind freilich auch hier die Informationen aus den Patientenakten, zu denen in zehn Fällen auch die Akten der Baumgartner Höhe eingesehen werden konnten, unzureichend: weder wurde für die Therapie mit der klinischen

---

589 Vgl. Abb. 7 und Text auf S. 152f.

Einschätzung des besonderen Falls schriftlich argumentiert, noch waren die Diagnosen und Symptome genügend differenziert, um parallelisiert werden und daraus auf einen Standard, auf die Kriterien für die unterschiedlichen therapeutischen Entscheidungen schließen zu können.

Zu der in diesem Exkurs diskutierten Hypothese, dass in einigen Fällen dieser Gruppe eine therapeutische Indikation der Malariafiebertherapie nur nachrangig oder gar nicht gegeben war,[590] ist zu erwähnen, dass eine nicht therapeutisch motivierte Indikation der Malariafiebertherapie in der Zwischen- und Nachkriegszeit auch andernorts aufgrund von Formulierungen ähnlich wie in den genannten Wiener Akten zu vermuten ist. Marietta Meier ist für den Hinweis auf zwei Patientenakten aus dem Züricher Archiv zu danken,[591] in denen ähnliche Formulierungen stehen, wie sie hier aus den Klinikakten zur Stammträgerfrage zitiert wurden:1938 erhielt ein 54-jähriger Patient mit der Diagnose „Hebephrenie" „Malariablut" „um unseren Stamm zu erhalten";[592] 1950 wurde der 57-jährigen Patientin mit der Diagnose „Paranoid" „Malariablut" injiziert „zur Erhaltung des Stammes".[593]

Um die Wahrscheinlichkeit zu erhärten, dass in Einzelfällen die Malariafiebertherapie angewandt wurde, nur um den Stamm zu erhalten, ist hier ein Hinweis in der eingesehenen wissenschaftlichen Literatur aus dem Ende der 1920er Jahre zu nennen: In *Der Nervenarzt* wird 1930 von einer Studie aus dem Utaca state hospital (Utaca, N. Y.) berichtet,[594] dass „zwecks Erhaltung eines luesfreien Plasmodienstammes zur Behandlung von Fällen von postencephalitischen Zuständen, multipler Sklerose usw. […] zunächst ein Schizophrener geimpft [wurde], später, da sich gewisse Besserungen zeigten, noch andere, im ganzen zwischen Juli 1925 bis September 1926 36 Fälle von Dementia praecox." Die erste

[590] Eine Stelle im Hoff-Skriptum, Allgemeine Psychiatrie [um 1961], S. 16 im Abschnitt über die Malariafiebertherapie bei PP könnte als Argument für die Hypothese einer vordringlichen oder ausschließlichen Anwendung in diesen Fällen zum Erhalt des Stammes sprechen: hier ist notiert, dass „sich debile eretische Schwachsinnige und Hebephrene" (das – als Nebenbemerkung des Vortragenden? – in Klammern) als „luesfreie" Stammträger eignen, ohne die Malariafiebertherapie später unter den Therapien bei diesen Diagnosen zu nennen. Freilich ist zu beachten, dass das aufgrund einer Mitschrift verfasste Skriptum nicht vom Vortragenden autorisiert wurde.
[591] E-Mail an Gernot Heiss vom 12.11.2014.
[592] StAZH, Z100, KA-Nr. 31146.
[593] StAZH, Z100, KA-Nr. 338001. Diese beiden Beispiele fielen Marietta Meier bei ihrer Arbeit auf, in der es um andere Fragestellungen ging; wahrscheinlich handelt es sich also nicht um die zwei einzigen Fälle.
[594] PILZ, Malariaimpfung in Fällen von Dementia praecox (1930), S. 58. Zitiert wird WARNER, Malaria inoculation in cases of dementia praecox (1928), S. 494.

Anwendung erfolgte demnach ohne therapeutische Absicht, nur um den luesfreien (!) Stamm zu erhalten.

Die Beurteilung dieser Anwendungen, in denen es möglicherweise bzw. in der US-amerikanischen Studie eindeutig um die Aufrechterhaltung des Malariastamms ohne therapeutische Indikation ging, führt zur Frage nach den medizin-ethischen Prinzipien dieser Zeit – die in den folgenden Schlussbemerkungen noch zu stellen sein wird.[595]

2.1.5 Zusammenfassung, Schlussbemerkungen und offene Fragen zur Anwendung der Malariafiebertherapie

Die sehr breite und sehr lange Anwendung der Malariafiebertherapie an der Wiener Klinik (bis Anfang 1969) und ihr therapeutisches Umfeld sowie die Frage nach den medizinischen Erwartungen in diese Therapie standen im Zentrum dieses Kapitels. Nach Ausführungen zur Geschichte und zur Diskussion über die Wirkmechanismen, zur Technik und zur Vielfalt der Anwendung, wurde der Frage nachgegangen, warum und mit welchen Argumenten an der Klinik Hoff nach der Einführung von Penicillin weiterhin die Malariafiebertherapie in ihrem klassischen Anwendungsgebiet bei neuroluetischen Erkrankungen angewandt wurde. Während es dazu (meistens für eine Anwendung in Kombination mit Penicillin) aufschlussreiche zeitgenössische Publikationen gab, fehlten diese fast völlig zu ihrer Anwendung bei PatientInnen ohne Neurolues. Zu hypothetischen Schlussfolgerungen über die Ziele ihrer Anwendung führten Hinweise in den zeitgenössischen Publikationen zur Anwendung der ‚großen' körperlichen Therapien, die in der Zwischenkriegszeit in der Psychiatrie als „Schocktherapien", nach Manfred Bleuler „als moderne Methoden der uralten Erschütterungstherapien" eingeführt worden waren und aus Hinweisen in den PatientInnenakten. Demnach war das Ziel der „Erschütterungstherapien" nach Bleulers Lehrbuch außerhalb der Neurolues – wozu in dieser Anwendung auch die Fiebertherapie gezählt wurde – (1) „die kurmäßige Beruhigung, (2) die Unterbrechung eines […] *Circulus vitiosus*, (3) die Ermöglichung eines Kontakts mit dem / der Patienten/in „in einer packenden, elementaren Art", (4) körperliche und psychische unspezifischen Anpassungsreaktionen aufgrund der „Belastung des Organismus (‚stress')" durch die Kuren.[596] In den

---

[595] Vgl. unten S. 169f.
[596] BLEULER, Lehrbuch der Psychiatrie (^{9}1955), S. 159f; BLEULER, Lehrbuch der Psychiatrie (^{10}1960), S. 159; BLEULER, Lehrbuch der Psychiatrie (^{11}1969), S. 164. Vgl. das etwas ausführlichere Zitat oben S. 29. Zu diesen Therapiezielen gab es auch direkte oder indirekte Hinweise in zeitgenössischen wissenschaftlichen Publikationen.

PatientInnenakten wurden direkt oder indirekt als Therapieziele vor allem Beruhigung, Minderung der Ängste und Spannungen, Ansprechbarkeit der PatientInnen für die PsychotherapeutInnen, Reifung der Jugendlichen, Wiedereingliederung in das soziale Umfeld genannt.

Daten aus PatientInnenakten der Erwachsenenstationen der psychiatrischen Universitätsklinik in Wien mit den fünf Diagnosen Neurolues, Intelligenzmängel, Schizophrenie, affektive Störungen, Psychopathie, bei denen mehrmals (in 772 Fällen) eine Malariakur gegeben wurde, wurden in eine SPSS Datenbank aufgenommen, ob sie nun eine Malariafiebertherapie bekamen oder nicht. Ebenso Daten jener PatientInnen mit anderen Diagnosen, diese jedoch nur, wenn sie eine Malariakur bekamen (97 Fälle).[597] Zur Frage nach der Zielsetzung wurden Fallbeispiele und Wiener sowie internationale wissenschaftliche Publikationen der Zeit nach Hinweisen ausgewertet, die die Einschätzung der Wirkweise der Therapie durch die Ärzte/innen und ihre Erwartungen in die Indikation erschließen lassen. Diese Informationen waren nur zur Malariafiebertherapie bei Neurolues ausführlich, in den anderen Fällen so selten und kurz, dass daraus nur in Ansätzen und hypothetisch die Erwartungen der Ärzte/innen in die Therapie und keine standardmäßige Indikation der Malariafiebertherapie abzuleiten war.

Zur klassischen Indikation der Malariafiebertherapie bei Neurolues ist aus den obigen Ausführungen zusammenfassend zu rekapitulieren, dass diese klassische Indikation 163 PatientInnen betraf (ca. 21 % der Fälle mit Fiebertherapie – in 140 Fällen PatientInnen mit progressiver Paralyse, in 21 Fällen mit „Tabo-P.P." und in 2 Fällen mit „Tabes dorsalis"). Deutlich wurde auch, dass die Zahl der PatientInnen mit Neurolues wie auch ihre Behandlung mit der Malariafiebertherapie in den 1960er Jahren sehr stark zurückgingen.[598] Durch die Ausheilung der Syphilis in früheren Stadien mit Penicillin kam es zu einer radikalen Abnahme ihrer Endstadien. Dadurch und durch Penicillin als wirksame Therapie auch der Neurolues verlor die Malariatherapie in den 1950er und 1960er Jahren zunehmend ihr historisches Hauptanwendungsgebiet. Bei der Diagnose progressive Paralyse war die Malariafiebertherapie in diesen Jahren jedoch in Wien noch Standardtherapie; bereits vom

---

[597] Von 14.919 Fällen (es sind 11.720 PatientInnen mit 3.199 Mehrfachaufnahmen), die aufgrund der Einschlusskriterien aufgenommen wurden, wurden PatientInnen in 6.915 Fällen mit Krampf-, Koma- und / oder Fiebertherapie behandelt. Vgl. oben S. 19f. und Tab. 1 auf S. 34 und die folgenden Ausführungen.
[598] Dazu und zur Verteilung über die beiden Jahrzehnte s. oben S. 74 Abb. 2.

Anfang der Untersuchungsperiode an wurde sie aber zunehmend und schließlich standardmäßig auch hier mit Penicillin kombiniert. Klinische Symptome der Spätsyphilis waren nach Meinung nicht allein der Wiener PsychiaterInnen nur durch die Fiebertherapie zu bessern.[599] In der regen Diskussion in der wissenschaftlichen Literatur gab es bis Ende der 1960er Jahre nicht nur in Wien,[600] sondern auch international Fürsprecher einer kombinierten Malaria-Penicillin Therapie.[601] Während in Wien nur PatientInnen mit geringen klinischen Symptomen mit Penicillin allein behandelt wurden, setzte sich international die Behandlung der progressiven Paralyse nur mit Penicillin in den 1950er und 1960er Jahren durch. Unter den AutorInnen nahm die Überzeugung zu, dass die Behandlung mit Penicillin nicht nur einfacher und problemloser als in ihrer Kombination, sondern auch im Erfolg gleichwertig sei – der Kombination mit der Malariafiebertherapie also nicht bedürfe.

Es gab, abgesehen von der Zunahme der BefürworterInnen einer ausschließlichen Penicillin-Therapie auch andere Gründe dafür, dass die Malariafiebertherapie international nur mehr an wenigen Kliniken angewandt wurde: die Therapie galt zwar auch für ältere ParalytikerInnen ohne schwere Herz-, Leber- und Lungenerkrankungen als gut verträglich, konnte jedoch ohne die nötige Obsorge und Kenntnisse zu lebensgefährlichen Situationen führen;[602] sie erforderte viel Betreuung durch das Pflegepersonal und technisches Geschick. Vor allem aber war Malariablut nicht leicht zu bekommen,[603] was auch nach Hinweisen in den zeitgenössischen Publikationen zur einfacheren Behandlung nur mit Penicillin beitrug.

In Wien war die Anwendung der Malariafiebertherapie möglich, da der *Malaria tertiana* Stamm durch die Anwendung bei anderen psychiatrischen Diagnosen bzw. deren Symptomen

[599] Die Beobachtung, dass (nur) bei den hohen Temperaturen der Malariakur die Durchlässigkeit der Blut-Liquor Schranke für Penicillin gesteigert werde, könnte diese Meinung bestärkt haben; vgl. oben S. 53.
[600] So die Wiener Klinikerin Klara Weingarten in ihrem Artikel von 1960 mit in Bezug auf Symptomatik und Indikation sehr differenzierten Argumenten: vgl. oben S. 69f.
[601] Vgl. u.a. die Zitate aus einer Diskussion in *Der Nervenarzt* 1953 / 1955 oben S. 67 und die Zitate aus den Auflagen von 1960 und 1969 des Bleuler'schen Lehrbuchs der Psychiatrie, oben S. 68 Anm. 213.
[602] Bei den 869 Malariakuren, die in den untersuchten Akten beschrieben werden, gab es an der Klinik drei Todesfälle während einer Malariafiebertherapie, alle in den 1950er Jahren: vgl. oben S. 57 Anm. 177. Wie von C. S. NICOL, The treatment of neurosyphilis (1953), S. 32 bzw. S. 30, wird wiederholt darauf hingewiesen, dass sie nur in einem „special centre" angewandt werden soll, „where expert medical and nursing care will reduce the risk of a fatality to a minimum." Nicol nennt: „Such a centre exists in this country - The Mott Clinic, Epsom."
[603] Vgl. MÜLLER, Grundlagen und Methodik der somatischen Behandlungsverfahren der Psychiatrie: Einleitung (1963), S. 385, rechtfertigt, dass die Malariatherapie nicht in einem eigenen Abschnitt behandelt werde, mit dem Ersatz durch die Penicillinbehandlung und damit, dass es kaum mehr möglich sei, Malariastämme aufzutreiben, wenn die Behandlung doch einmal durchgeführt werden soll." Vgl. auch oben S. 32 Anm. 96_zu den Problemen der Züricher Klinik bereits 1948/49.

erhalten wurde, was anderswo nicht mehr der Fall war.[604] Von den 772 Malariakuren bei PatientInnen mit Diagnosen, die den Einschlusskriterien der Untersuchung entsprachen, wurden 609, d.h. fast 79 % nicht bei neuroluetischen sondern bei schizophrenen Erkrankungen und affektiven Störungen, bei Diagnosen aus dem Bereich der Intelligenzmängel oder bei ‚Psychopathie' gegeben – in weiteren 97 Fällen ohne Neurolues, bei Diagnosen, die nicht den Einschlusskriterien entsprachen.

Vergleicht man die Anwendung der Malariafiebertherapie bei Frauen und Männern, so zeigte sich, dass Patientinnen die Malariafiebertherapie in 51 Neuroluesfällen (drei Patientinnen mit progressiver Paralyse zweimal[605]) bekamen, jedoch nur in 13 Fällen bei Diagnosen außerhalb der Neurolues.[606] Während der Geschlechteranteil in der Anwendung der Malariafiebertherapie bei Neurolues in ihrer spezifischen Wirkung gegen das *Treponema pallidum* mit 112 Patienten : 51 Patientinnen relativ ausgeglichen war,[607] war er mit 13 Patientinnen : 693 Patienten bei anderen Diagnosen extrem ungleich.[608] Geimpft wurden vor allem männliche Patienten mit Diagnosen und mit Symptomen, die mit dem Ziel Beruhigung, Umstimmung, „Nachreifung", kommunikative Öffnung und soziale Reintegration zumindest in den 1960er Jahren anderswo nur mehr mit den neuen Psychopharmaka und / oder mit Psychotherapie oder auch mit anderen körperlichen Kuren, mit Elektrokrampf- oder Insulinkomatherapie, mit Schlaf- oder Dämmerkuren behandelt wurden. Zugleich wurde in Wien bei den Diagnosen außerhalb der Neurolues, die den Einschlusskriterien des Projekts entsprachen, in der deutlichen Mehrheit der Fälle (vgl. oben Tab. 1, die Kreuztabelle Diagnosen und Koma-, Krampf- bzw. Fiebertherapien)[609] – und es geht bei diesem Vergleich

[604] So die Schlussfolgerung aus der eingesehenen Literatur und einzelnen Informationen von Zeitzeugen. Der Vorschlag des rumänischen Malariologen Lupascu von 1972 (vgl. oben S. 82 und unten S. 169 Anm. 628 und 629) und seine Hinweise auf Zentren der Malariaimpfung dürften einer völligen Aufgabe der ‚Pflege' eines Malariastamms widersprechen.
[605] Vgl. KAINZ, Retrospektive Datenanalyse iatrogener P. vivax Infektionen (2019), S. 49. Mit anderen Diagnosen wurde kein/e Patient/in gefunden, der / die die Malariafiebertherapie zweimal bekam.
[606] Vgl. oben die S. 62f. Die Frage, warum Patientinnen ohne Neurolues im Vergleich zu den männlichen Patienten so selten eine Malariakur bekamen, ist aus den vorliegenden Quellen nicht zu beantworten. Von 6 dieser 13 Patientinnen wurde das malariainfizierte Blut weitergegeben, vgl. oben S. 153. An der Kinderstation war das Verhältnis ausgeglichener: nach den überlieferten Akten bekamen dort 10 Mädchen und 25 Knaben eine Malariafiebertherapie, vor allem Kinder mit Intelligenzmängeln bzw. Entwicklungsstörungen.
[607] Vgl. oben S. 62 bei Tab. 12.
[608] PatientInnen mit einer der vier nichtluetischen Diagnosen, die den Einschlusskriterien entsprechen, bekamen 596 Malariafiebertherapien; dazu sind noch die 97 PatientInnen zu rechnen, die mit einer der nicht den Einschlusskriterien des Projekts entsprechenden Diagnosen eine Malariafiebertherapie bekamen.
[609] Oben S. 34: Von 11.349 PatientInnen mit den vier den Einschlusskriterien entsprechenden nichtluetischen Diagnosen (11.720 – 371 mit Lues) bekamen 706 PatientInnen eine Malariafiebertherapie.

nur um jene PatientInnen, die länger als 4 Tage aufgenommen waren – keine Malariafiebertherapie gegeben.

Zum Verständnis dieser gegensätzlichen Entscheidungen in der Anwendung der Malariafiebertherapie bei PatientInnen mit gleichen Diagnose und vergleichbaren Symptomen in Wien kann Manfred Bleulers Rechtfertigung der unterschiedlichen Entscheidungen der Ärzte/innen bei der Behandlung mit den ‚großen' körperlichen Kuren allgemein dienen, zu denen die Malariakur gezählt wurde: Die unterschiedliche Entscheidung beruhe auf der Erfahrung der Ärzte/innen, dass die PatientInnen auf diese Therapien bei gleicher Krankheit ganz unterschiedlich reagieren würden. Deshalb sei das „Wirrwarr der Meinungen" zur Indikation der ‚großen' körperlichen Kuren „durchaus verständlich".[610] Er anerkannte damit die Entscheidung für bzw. gegen eine der körperlichen Therapien (auch jene mit den neuen Psychopharmaka) im Einzelfall aus der klinischen Erfahrung des Arztes / der Ärztin sowie aus seiner / ihrer Einschätzung und Beurteilung des / der einzelnen Patienten/in und seines / ihres Krankheitsfalls.

Zur Entwirrung der unterschiedlichen Indikationsentscheidungen im Einzelfall fehlt deren Verschriftlichung in den Krankengeschichten mit Argumenten für die Anwendung der Therapie und zu den Vorstellungen über ihre Wirkweise. Es finden sich jedoch kurze Bemerkungen, die auf eine Zielsetzung schließen lassen, vor allem wenn sie in Bezug zu den wenigen, ebenfalls meist sehr kurzen Hinweisen in Wiener und internationalen Publikationen gestellt werden. So brachten die Krankenakten keine Klarheit in der Frage, in welchen Fällen die Malariafiebertherapie bei PatientInnen mit Diagnosen außerhalb der progressiven Paralyse die Therapie der Wahl war.[611]

In den eingesehenen zeitgenössischen Publikationen wurden Anwendungen der Malariafiebertherapie bei Diagnosen außerhalb der Neurolues bei schizophrenen Erkrankungen, Intelligenzmängeln, affektiven Störungen bzw. bei ihren Symptomen genannt. Nur zur Anwendung der Malariafiebertherapie bei ‚zerebralgestörten' Kindern konnte eine

---

[610] Vgl. die Zitate von Bleuler: oben S. 33 zum Verständnis für das „Wirrwarr der Meinungen" über die Therapien, und oben S. 31, dass es bei der Auswahl des körperlichen Verfahrens auch auf die „individuell günstige oder ungünstige Erfahrung mit der einen oder anderen Behandlung" ankomme.

[611] Auch die Angaben von spezifizierten Diagnosen in den Diagnosefeldern und die Bemerkungen über die Symptome im Krankenakt trugen nicht zur Klärung, sondern nur zu hypothetischen Schlussfolgerungen bei.

ausführliche Wiener Publikation mit Hypothesen zur Wirkweise zitiert werden.[612] Für die Malariafiebertherapie als eine der „unspezifisch" wirkenden „Schock"-, respektive „Erschütterungstherapien" gelten auch die Wirkungen, die für diese ‚großen' körperlichen Kuren in den Publikationen angegeben[613] und manchmal aus Bemerkungen in den Krankenakten zu erschließen sind: die Therapie sollte beruhigen, umstimmen, dem Arzt / der Ärztin einen Zugang zu den PatientInnen öffnen und – vor allem die Fiebertherapie – einen Reifungsprozess anregen.[614] Diese Wirkungen sollten mit den nachfolgenden sozial integrierenden Therapien wie Psychotherapie und Arbeitstherapie eine Besserung der Einbindung in das soziale Leben des Alltags an der Klinik („Anpassung") als Voraussetzung für den Alltag außerhalb bringen.

Hingegen findet sich in der eingesehenen zeitgenössischen Literatur kein Hinweis auf die in der Wiener Praxis häufige Anwendung der Malariafiebertherapie bei „Psychopathie". Dazu müsste wohl in der Literatur der 1930er und 1940er Jahre recherchiert werden. So erwähnen Hans-Walter Schmuhl und Volker Roelcke 2013 rückblickend auf die Zeit vor 1945 ohne weitere Ausführungen, dass die Malariakur in verschiedenen Ländern bald nach ihrer Einführung auch bei Schizophrenen, bei Patienten mit bipolaren Störungen und „selbst" bei Patienten mit einer Psychopathie-Diagnose angewandt wurde.[615] An der Diagnose Psychopathie wurde schon damals heftig kritisiert, dass sie zu breit vergeben und zu unpräzise definiert würde. In Wien wurde sie als ‚Syndrom' charakterisiert. Es ist auch hier davon auszugehen, dass sich die therapeutische Anwendung der Malariakur auf Symptome und Symptomkombinationen bezog, die sich fast durchwegs mit den Symptomen bei PatientInnen überschneiden, die mit anderen der nicht-luetischen Erkrankungen eine Malariakur bekamen (heftige Erregungen, Reiferückstand, Persönlichkeitsstörung, Verschlossenheit, Suizidgefährdung u. ä.) und bei denen sie mit gleichen Erwartungen gegeben wurde (Beruhigung, Minderung der Ängste und Spannungen, Ansprechbarkeit der PatientInnen für

[612] KUNDRATITZ, Die therapeutische Beeinflußbarkeit zerebralgestörter Kinder (1957), S. 423-427 (vgl. oben S. 91f.) blieb jedoch ohne Diskussion in den eingesehenen Publikationen und ist deshalb in ihrem Stellenwert nicht zu bestimmen. MÜLLER-KÜPPERS, Die Therapie im Kindes- und Jugendalter (1972), S. 998, erwähnt die Malariakur jedoch noch 1972 in ihrer nur klinischen Anwendung „z.B. bei hirngeschädigten Kindern" (zitiert oben S. 93 Anm. 325).
[613] Vgl. die Zitate aus Publikationen des Züricher Psychiaters Manfred Bleuler, oben S. 28-33.
[614] Vgl. Kundratitz über die Indikation der Fiebertherapie zur ‚Umstimmung' bzw. (nach Spiel) „Verbesserung der Intelligenzfunktion" oben S. 93 und mehrere Hinweise in Publikationen und Krankenakten zur ‚(Nach)reifung' (vgl. z. B. in vielen Beispielen von S. 83f. und im Kapitel 2.1.4.2.4 Psychopathie S. 108-143).
[615] SCHMUHL/ROELCKE, Einleitung (2013), S. 23f. (dazu und zu ihrer Begründung dieser „Bereitschaft zum ‚off-label-use'", vgl. oben S. 78).

die TherapeutInnen, Reifung der Jugendlichen, Eingliederung in bzw. ‚Anpassung' an das soziale Umfeld).

Auch zu den Fällen, in denen Alkoholkranke eine Malariafiebertherapie bekamen, gibt es in den Wiener wissenschaftlichen Publikationen keinen Hinweis auf ihre Anwendung, wohl aber Hinweise in einzelnen PatientInnenakten, dass es sich um eine ‚Vorbehandlung' bzw. ‚Vorbereitung' eines Entzugs im *Genesungsheim Kalksburg* handle. Diese Hinweise stützen die Hypothese, dass mit dieser Therapie die PatientInnen ‚umgestimmt' und für soziale Kontakte und psychotherapeutische Gespräche ‚geöffnet' werden sollten.[616]

Die Öffnung einer kommunikativen Zugänglichkeit der PatientInnen, wie sie hier als Ziele der Malariakur zur Vorbereitung einer Entwöhnung angenommen wird, wurde in der medizinischen Literatur der Zeit mehrmals ‚großen' körperlichen Kuren zugeschrieben. So schrieb Manfred Bleuler allgemein den „Erschütterungsbehandlungen" zu, „einen Kontakt" mit dem Patienten „in einer packenden, elementaren Art" zu ermöglichen und nennt als erstes ihrer drei therapeutischen Ziele: „die Bildung eines engeren Verhältnisses mit Schwestern, Pflegern und Ärzten" – neben „der Überraschung und Erschütterung und der Beruhigung".[617] Daniel Helmchen zitiert 1999 in diesem Sinne auch die beiden Psychiater des frühen 20. Jahrhunderts Emil Kraepelin und Jacob Klaesi[618] und sieht rückblickend darin die einzige Ursache für Therapieerfolge: diese würden sich in der Behandlung „auch ‚reine[r]' Schizophrene[r], d.h. Geisteskranke[r] ohne syphilitische Vorgeschichte, nach dem Prinzip ‚Malariainfektion gegen Psychosen'" alleine aus der „‚Erschöpfung' des Patienten" und der damit „einhergehende[n], intensive[n] Interaktion der Patienten mit den Helfenden" ergeben.[619]

Mehrfach wurde die Indikation bei PatientInnen mit Persönlichkeitsstörungen mit den therapeutischen Zielen einer „Umstimmung" und „(Nach)Reifung" gegeben, sei es bei

---

[616] Vgl. oben S. 148.
[617] Vgl. oben S. 29-31.
[618] Vgl. oben S. 117.
[619] HELMCHEN, Malariaimpftherapie der Progressiven Paralyse in den Wittenauer Heilstätten (1999), S. 444-446 (ausführlicher zitiert oben S. 117f.). Vgl. dazu auch den Brief einer Mutter oben S. 118. und HOFF, Lehrbuch der Psychiatrie (1956), S. 519f. unten S. 200f. zum Ende der Insulinkomatherapie als günstigen Zeitpunkt, um mit einer Psychotherapie zu beginnen. Zur Verbesserung der Kommunikation aufgrund der Veränderungen in der Wahrnehmung der psychiatrischen PatientInnen als behandelbare Kranke durch die Ärzte/innen seit der Entdeckung der Malariafiebertherapie gegen progressive Paralyse, vgl. oben S. 76 Anm. 246 zu den textkritischen Publikationen von Joel T. Braslow 1995 und 1996.

Intelligenzmängeln[620] (u. a. mit einer Persönlichkeitsstörung aufgrund von Encephalitiden[621]), sei es bei „Psychopathie". Auch bei jungen PatientInnen, „bei denen auf der Basis einer Reifungsstörung […] in der Pubertät oder beginnenden Reifezeit eine primäre schizophrene Prozeßpsychose ausgeklinkt wird",[622] erwähnte der Klinik Ottokar H. Arnold in seinem Buch zur Schizophrenie 1963 eine Anwendung der Malariafiebertherapie. Zu diesem Problem sind auch die Ausführungen im Text zur Anwendung der Fiebertherapie bei „Reiferückständen" bei Patienten mit der Diagnose Psychopathie zu nennen, auf die bei ‚leicht abnormen' EEG-Befunden geschlossen wurde.[623] Da für eine wissenschaftliche Überprüfung der Wirkung der Malariafiebertherapie als „Erschütterungstherapie" bei einer nicht-luetischen Erkrankung in den 1950er und 1960er Jahren kein Beleg gefunden wurde, ist zu schließen, dass die therapeutische Anwendung der ‚großen körperlichen Kur' nur aufgrund älterer Erfahrungen aus der klinischen Praxis erfolgte.

Die Aufrechterhaltung eines ‚luesfreien Malariastamms' durch kontinuierliche Überimpfung (Infektion mit dem *Plasmodium vivax* durch Blutübertragung) zur breiten Anwendung der Fieberkur war – wie bereits vor der ‚Ära Hoff'[624] – Praxis. Für die Aufrechterhaltung dürfte auch von Bedeutung gewesen sein, dass die Malariafiebertherapie in der ‚Ära Hoff' (1951-1968) auch an anderen Kliniken bei progressiver Paralyse, in der physiotherapeutischen Nachbehandlung der Kinderlähmung und vermutlich auch noch in der Dermatologie[625] angewandt wurde, wozu das malariainfizierte (luesfreie) Blut – wie auch vor 1950[626] – von der Wiener Psychiatrie bezogen wurde.

Im Text wird die Hypothese vertreten, dass der Stamm durch „Überimpfung", durch die Setzung von Malaria-Infektionen bei Patienten erhalten wurde, deren Diagnosen und vor

---

[620] Zur Indikation der Malariafiebertherapie bei Kindern mit ‚Intelligenzmängeln' vgl. die oben S. 91f. ausführlich zitierte Publikation von Karl Kundratitz.
[621] Zur Anwendung bei Folgeschäden einer Enzephalitis an der Grazer Klinik, vgl das Zitat PAKESCH, Spezielle Therapie der Nervenkrankheiten (1951), S. 160-162, oben S. 127 Anm. 474.
[622] Siehe oben S. 83 das Zitat von ARNOLD, Die Therapie der Schizophrenie (1963), S. 59-61 zu den Diagnosen, „die man früher unter dem Begriff der ‚Pfropfschizophrenie' oder ‚Pfropfhebephrenie' zusammengefaßt hat".
[623] Siehe oben S. 122-126.
[624] An der Wiener Klinik war auch vor der ‚Ära Hoff' ein ‚luesfreier Malariastamm' vorhanden, d.h. die Parasiten wurden über PatientInnen übertragen, die keine Syphilis hatten. Vgl. dazu KAUDERS, Zur Klinik, Theorie und Geschichte der Malariabehandlung (1948), S. 68, wo er über die Nachbehandlung der Kinderlähmung mit der Malariafiebertherapie schreibt – also von PatientInnen ohne Syphilis, d.h. mit der Übertragung von luesfreiem malariainfizierten Blut – und sich auf eine eigene Publikation von 1937 zum Thema bezieht, zitiert oben S. 79 Anm. 263.
[625] Dazu im Text für die 1930er und 1940er Jahre die Hinweise in der Literatur, zitiert oben S. 78 Anm. 257.
[626] Vgl. zu Graz bzw. München vor 1951: oben S. 54 Anm. 160.

allem Symptome aufgrund der klinischen Erfahrung der älteren Psychiater von der Malariafiebertherapie eine therapeutische Wirkung erwarten ließen, wenn auch international (und in vielen Fällen auch in Wien) nun dazu eine körperliche Therapie mit den neuen Psychopharmaka oder eine psychotherapeutische Behandlung angewandt wurde.

Da an der Wiener Klinik viele PatientInnen mit den gleichen Diagnosen und Symptomen auch keine Malariafiebertherapie bekamen, lassen die, wenn auch nur kurzen Informationen zu den Zielen der Therapie darauf schließen, dass die Fiebertherapie in der Zeit der Psychopharmaka häufig statt einer medikamentösen Therapie gewählt wurde, da mit dieser (alten) therapeutischen Anwendung zugleich der *Malaria tertiana* Stamm erhalten wurde. Es ist aber auch nicht auszuschließen, dass von ihr im speziellen Fall bessere Ergebnisse erwartet wurden oder die manchmal schweren unerwünschten Nebenwirkungen der neuen Medikamente mit der stark invasiven Malariakur (obwohl sie mit ihren hohen Fieberschüben nicht ungefährlich war[627]) vermieden werden sollten. Dass diese therapeutische (!) Anwendung mit der zweiten Funktion, den Stamm zu erhalten, in diesen Jahren noch denkbar war, zeigt u. a. eine Publikation des rumänischen Tropenmediziners G. Lupascu von 1974: Im Interesse der Malariaforschung empfahl er, zur Aufrechterhaltung der Malariakette in Zentren „d'impaludation thérapeutique"[628] auf jene psychiatrischen PatientInnen zurückzugreifen,[629] die nun bei gleichen Symptomen ‚mit voller Berechtigung' – wie er vorher geschrieben hatte – mit den neuen Beruhigungsmitteln behandelt würden.[630]

Nicht auszuschließen ist, dass in mehreren Fällen die Funktion, den Malariastamm durch zeitgerechte Überimpfung zu erhalten, allein die Anwendung bestimmte, die Indikation also

---

[627] Vgl. zu den drei Todesfällen während einer Malariafiebertherapie oben S. 57 Anm. 177.
[628] Es gäbe seines Wissens nun nur noch zwei dieser Zentren, eines in Socola-Jassy in Rumänien und eines in Chamblee-Atlanta in den USA. Vgl. auch oben S. 82 Anm. 278.
[629] Zu den Indikationen, die Lupascu nennt, vgl. oben S. 82.
[630] LUPASCU, Applications actuelles de la malariathérapie (1974), S. 166, zitiert oben S. 82 Anm. 278 (aus dem Französischen übersetzt von GH). In Wien findet sich in der ‚Ära Hoff' kein Hinweis auf eine Kooperation mit der Malariaforschung durch eine Bereitstellung von Malariafiebertherapie-PatientInnen für die Testung von Medikamenten, wie sie G. Lupascu hier anspricht und wie sie in den 1950er Jahren im Zusammenhang mit dem Malaria-Eradication-Programm der WHO sehr aktuell war (vgl. HULVERSCHEIDT, Forschungslenkung international – Malariaforschung im Rahmen des Malaria Eradication Programme der WHO 1955-1972 (2010), S. 133-146; vgl. auch oben S. 65). Zur Malariaforschung ist damals im Bereich des Hygieneinstituts einzig Ludwig Popper zu nennen, der seine Erfahrungen bzw. Forschungen zur Malaria im Militär-Sanitätsdienst in der Emigration in Bolivien gemacht hatte; 1947 hielt er noch in La Paz einen Vortrag zu „Rationelle Malariabehandlung" (UA Wien, Med. PA 420). Aus seinem Personalakt im Wiener Universitätsarchiv mit Publikationslisten in den 1960er Jahren geht hervor, dass er nicht weiter zur Malaria forschte. 1948 habilitierte er sich in Wien für innere Medizin. Er war Primar in Lainz, dann am Wilhelminenspital und hielt (1963/64 und wiederholt) am Hygieneinstitut auch eine Vorlesung zu Tropenkrankheiten.

ohne therapeutische Erwartung gegeben wurde. Im konkreten Fall ist eine nicht-therapeutische Indikationen jedoch kaum eindeutig zu klären und noch weniger ihre Häufigkeit. Sie ist in der Zwischen- und Nachkriegszeit auch anderswo aufgrund von Formulierungen im Krankenakt zu vermuten bzw. in einer Erwähnung in einer der wissenschaftlichen Publikationen nachzuweisen.[631] Die Beurteilung dieser Anwendungen, in denen es eindeutig bzw. möglicherweise um die Aufrechterhaltung des Malariastamms ohne therapeutische Indikation ging, führt zur Frage nach der damaligen medizinischen Ethik. Abgesehen vom geltenden Grundsatz *primum non nocere* scheint das Verhalten in diesen Fällen stark von allgemeinen und nicht von individuellen, auf den Patienten / die Patientin gerichteten Behandlungsinteressen bestimmt gewesen zu sein. Die Frage, was bringt die Behandlung dem Patienten / der Patientin, schien in solchen Fällen nicht maßgeblich.

Allgemein wurde die ‚ärztliche Entscheidungsautorität' hinsichtlich der anzuwendenden Therapie[632] zwar in diesen Jahren von JuristInnen und BioethikerInnn zunehmend hinterfragt, es wurde auch die „informierte Zustimmung" des Patienten / der Patientin zur Behandlung rechtlich fixiert,[633] im Selbstverständnis der Ärzte/innen und in der Praxis führte das jedoch nur langsam zu Veränderungen.[634] Zu dieser Verzögerung trug bei, dass auch vonseiten der PatientInnen bzw. ihrer VertreterInnen verbreitet eine nicht zu hinterfragende ärztliche Autorität[635] anerkannt wurde. Das dürfte sich etwa in der äußerst seltenen Verweigerung des „Reverses", d. h. der Zustimmung des /der Patienten/in bzw. seines / ihres Vormunds zu einer der ‚großen' alten Therapien an der ‚Klinik Hoff' zeigen.[636] Außerdem haben eine

---

[631] Vgl. dazu die Beispiele oben S. 160.
[632] Vgl. MISSA, Naissance de la psychiatrie biologique. Histoire des traitements de maladies mentales aux XXe siècle (2006), S. 327, der rückblickend von der 'paternalistischen Epoche' der 1940er und 1950er Jahre spricht, in der der Arzt / die Ärztin alleine die Behandlung bestimmte, und es eigentlich niemand für notwendig erachtete, sich an die vorhandenen einschränkenden Gesetzte zu halten.
[633] Vgl. (wenn es hier auch um Forschung und Testungen geht) ECKART, Introduction (2006), S. 10, sowie ECKART/REULAND, Julius Moses (2006), S. 35-47. Zur Entwicklung in der Psychiatrie in Österreich vgl. FORSTER, Staat, Politik und Psychiatrie in Österreich – am Beispiel der rechtlichen Regulierung von Zwangsmaßnahmen von 1916 bis 1990 (1999), zitiert oben S. 86 Anm. 300**.**
[634] Zur Geschichte dieses Wandels in den 1950er und 1960er Jahren vgl. ROTHMAN, Strangers at the bedside (^{4}1998), passim.
[635] Zur Festigung dieser gesellschaftlichen Stellung des Arztes haben die großen Erfolge der Bakteriologie mit den ‚Helden' Pasteur und Koch Ende des 19. Jahrhunderts sowie die anderen großen Fortschritte der Medizin und die daraus resultierende enge Verbindung mit dem Staat (Seuchenbekämpfung, Volksgesundheit) beigetragen. (Vgl. dazu die Ausführungen zu den sozialen und wissenschaftlichen Umbrüchen in ECKART, Geschichte, Theorie und Ethik der Medizin (2013), S. 167-226, Kapitel 7 „Aufbruch in die Moderne – die Medizin des 19. Jahrhunderts"). Die staatliche Förderung der medizinischen Forschung aufgrund dieser Verbindung hatte im Fortschritt der Medizin durchaus positive, aber in der ideologischen Beeinflussung der Forschung auch extrem negative Folgen. Zu letzterem vgl. die Beiträge in ECKART (Hrsg.), Man, Medicine and the State (2006).
[636] Vgl. oben S. **145** Anm. 538.

uneinheitliche und nicht normierte Diagnostik in der Zeit vor einer breiten Anerkennung des ICD-8 (1965) und noch lange bevor in den 1980er und 1990er Jahren Therapie-Guidelines der Fachdisziplinen herausgegeben wurden, subjektive, vom Arzt / von der Ärztin geprägte Therapieentscheidungen erleichtert.

Hans Hoffs Mitarbeiter und Nachfolger in der Klinikleitung[637] Peter Berner beschrieb in seinem biographischen Artikel 1998 kritisch Hoffs Beharren auf den alten körperlichen Therapien und führt dies auf seine Ausbildung „in erster Linie als Neurologe“ zurück. Deshalb sei ihm „in der psychiatrischen Diagnostik vor allem die Erkennung organisch begründbarer Störungen mittels exakter neurologischer Untersuchungen am Herzen“ gelegen. „Hoffs Verwurzelung in organische Krankheitskonzepte war wohl dafür ausschlaggebend, daß er niemals an der somatischen Bedingtheit schizophrener und manisch-depressiver Psychosen zweifelte, für welche er eine rein psychogene Entstehung ausschloß, da er sie als erblich bedingt erachtete.“[638]

Es wurde oben im Text zur Diskussion um Malariatherapie und / oder Penicillin bei der progressiven Paralyse das Referat beim 1. Weltkongress der Psychiatrie in Paris zitiert, in dem das Zögern älterer französischer Mediziner ‚erprobte' Therapien zu Gunsten neuer Therapien völlig aufzugeben, als Generationenproblem angesprochen wurde. [639] In Bezug auf die in der Zwischenkriegszeit entwickelten „heroischen Therapien“, wie die in der Zwischenkriegszeit entwickelten, stark invasiven Therapien genannt wurden, war diese Tendenz an der Wiener Klinik in der Leitung von Hans Hoff stark ausgeprägt. Das zeigt sich deutlich in der langen Anwendung der Malariakur wie der Insulinkur.

Hans Hoff, wie sein Vorgänger in der Klinikleitung Otto Kauders,[640] standen durch ihre Ausbildung und Karriere in der Wiener Tradition der Zwischenkriegszeit, deren Fortsetzung

---

[637] Nach Hoffs Tod als supplierender Leiter der Klinik für Neurologie und Psychiatrie, nach der Trennung der beiden Fachbereiche als Leiter der Psychiatrie.

[638] Berner, Hans Hoff (1998), S. 59. Hoff stand damit freilich in der Wiener Tradition, vgl Gröger/Kasper, Zur Dominanz der organisch-biologischen Auffassung in der Psychiatrie der Wiener medizinischen Schule und den Anfängen der Psychopharmakotherapie (1997), passim.

[639] Vgl. zur Diskussion Malariafiebertherapie und / oder Penicillin oben S. 64. den Beitrag zum 1. Weltkongress der Psychiatrie in Paris 1950 von Bessiere/Alizon, Premiers résultats de l'introduction de la pénicilline dans le traitement de la paralysie générale (1950), S. 1; zu dieser Tendenz in der britischen Diskussion, vgl. auch oben S. 65.

[640] In seinem Festvortrag zum 90. Geburtstag von Wagner-Jauregg schildert Kauders als Mitarbeiter begeistert die Atmosphäre an der Klinik in der Zeit der Entwicklung der Malariafiebertherapie und den „Eindruck der ersten Heilerfolge nach Malaria, der ersten, gleichsam aus tiefem Todesschlaf geistiger Umnachtung

für Hoff in seiner Antrittsvorlesung 1951 auch Programm wurde: „An dieser Klinik hat Wagner-Jauregg [im Text gesperrt] den Negativismus seiner Periode überwunden und hat mit großer Energie und Entschlossenheit die Malariafiebertherapie der progressiven Paralyse eingeführt. In dieser Stadt hat Freud [im Text gesperrt] den Widerstand der Gesellschaft seiner Zeit überwunden, hat die Triebregungen klar aufgezeigt und eine Therapie der Neurosen versucht.[641] War die progressive Paralyse durch einen bestimmten Erreger charakterisiert und dadurch angreifbar geworden, so schienen die funktionellen Psychosen lange Zeit dem therapeutischen Nihilismus ausgeliefert. Es war das Verdienst Sakels [im Text gesperrt] und die Erkenntnis Pötzls [im Text gesperrt] von der Wichtigkeit der Sakelschen [im Text gesperrt] Therapie, den Versuch zu wagen, auch da eine Bresche für die aktive Behandlung zu schlagen. Ich sehe es als meine Aufgabe an, alle Bemühungen und Forschungen dieser Klinik in dem einen Sinne zu koordinieren: den Unglücklichsten aller Kranken zu helfen, den Kranken des Geistes."[642] Ähnlich wie auch bei der Insulinkomatherapie, der zweiten ‚Wiener' Therapie mit Weltgeltung, dürfte dieser Rückbezug auf die großen Erfolge der „Wiener" Psychiatrie zur Aufrechterhaltung der Malariafiebertherapie und des *Malaria tertiana* Stamms in der ‚Ära Hoff' beigetragen haben.

2.2 Die Insulinkoma- und die Insulinsubkomatherapie[643]

Die Insulinkomatherapie, in der durch die Gabe von Insulin eine Unterzuckerung (Hypoglykämie) künstlich verursacht wurde, wodurch der Patient / die Patientin in ein Koma fiel, wurde in den 1940er und 1950er Jahren weit verbreitet in Europa und im angelsächsischen Raum angewandt.[644] Sie weckte die Hoffnung, ein schizophrenes Geschehen spezifisch behandeln zu können.[645]

---

erwachenden Geisteskranken," die „auf jedermann, der es miterleben durfte, tief erschütternd" gewirkt hätten: KAUDERS, Zur Klinik, Theorie und Geschichte der Malariabehandlung (1948), S. 50.

[641] Zum Ziel, beide Schulen zusammenzuführen, vgl. HOFF/ARNOLD, Die Therapie der Schizophrenie (1954), S. 345: „In Österreich versuchen wir, die Schwerpunkte der verschiedenen Auffassungen in der Psychiatrie zur Übereinstimmung zu bringen. Die Tradition der Klinik eines Meynerth, eines Economo, Wagner-Jauregg und Pölzl mit den Schulen von Freud und Adler. Es scheint, dass die Zeiten vorbei sind, in denen diese beiden Schulen differierende Wege einschlugen. Wenn wir sie zur Übereinstimmung bringen, werden die Verschiedenheiten ihre Bedeutung verlieren. Pötzl war es ja schließlich, der die Grundlage aufklären konnte und auf dem Boden pathologischer Hirnprozesse einen teilweisen Beweis für die Theorie der Psychoanalyse erbrachte."

[642] HOFF, Die organischen Grundlagen der Psychosen (1951), S. 5.

[643] Im Folgenden wird vor allem die Insulinkomatherapie behandelt, aber auch die Insulinsubkomatherapie (‚Subschocktherapie') mit geringeren Dosen von Insulin und ohne Koma.

[644] Zur raschen Verbreitung vgl. unten S. 173 Anm. 650.

[645] Vgl. Hans Hoff an das Nobelkomitee, 19.10.1957, Kopie im Josephinum Wien, Nachlass Hans Hoff, Signatur [im Folgenden: NL-Hoff] MUW-AS-006005-0011-068 Zl. 68/5: Es sei das „Verdienst Sakels gezeigt zu haben, dass eine Möglichkeit besteht, die Schizophrenie zu heilen". Vgl. hingegen BLEULER, Lehrbuch der Psychiatrie

2.2.1 Geschichte und Diskussion der Insulinkomatherapie

Die Insulinkomatherapie war in ihrer Entwicklung eng mit der Wiener psychiatrischen Universitätsklinik verbunden. Der Begründer dieser Therapie, Manfred Sakel, stellte diese neue Behandlungsmethode erstmals 1933 in Wien vor. Otto Pötzl, der damalige Leiter der psychiatrisch-neurologischen Klinik,[646] bestätigte die Erfolge der Kur und erachtete die Intensität des Eingriffs aufgrund der Schwere der Krankheit Schizophrenie als gerechtfertigt. 1936 richtete Sakel mit der Unterstützung Pötzls eine Insulinstation ein. Bereits von Anfang an gab es in Wien jedoch auch Psychiater, die dieser Methode skeptisch gegenüberstanden, so Josef Berze und auch Julius Wagner-Jauregg.[647] Hans Hoff hingegen war ein überzeugter Verfechter der Insulinkomatherapie.[648] Otto Pötzl schrieb 1958 in der Festschrift für Hoff zum 60. Geburtstag, Hoff sei in den 1930er Jahren an der Entwicklung der Insulinkur beteiligt gewesen.[649]

Mitte der 1930er Jahre, nach dem Erscheinen von Sackel's Monographie „Neue Behandlungsmethode der Schizophrenie" 1935, bestand bereits weltweit großes Interesse an der Insulinkomatherapie.[650] Der internationale Ruf der Wiener Psychiatrie dürfte dazu

---

([10]1960), S. 159: „Früher wurde gehofft, die Insulinkur sei ein spezifisches Heilmittel gegen die Schizophrenie, meines Erachtens sind aber die Gegenargumente überzeugend. Von den Kuren mit Krämpfen und mit Medikamenten haben wir noch weniger Anlaß, eine spezifische Wirkung auf bestimmte Krankheiten zu vermuten."

[646] Otto Pötzl folgte 1928 seinem Lehrer und Förderer Wagner-Jauregg als Leiter der Klinik und wurde als Mitglied der NSDAP (vor 1933 und wieder 1938/1941) 1945 enthoben und pensioniert (vorübergehend mit gekürzten Bezügen): vgl. GABRIEL, Zum Wiederaufbau akademischer Lehrkörper in der Psychiatrie in Wien nach 1945 (2016), S. 37-39.

[647] Vgl. den kurzen Rückblick in MÜLLER, Insulinbehandlung (1952), S. 2. Zu den Kritikern in der britischen Kontroverse über die Insulinkomatherapie seit ihrer Einführung in den späten 1930er Jahre, vgl. FREUDENTHAL/MONCRIEFF, ‚A landmark on psychiatric progress'? The role of evidence in the rise and fall of insulin coma therapy (2022), S. 67-69.

[648] GRÖGER, Insulin-Schocktherapie (2005), S. 210f.

[649] PÖTZL, Widmung an Hans Hoff (1958), S. 6: „Als (1934) Manfred Sakel an die Wiener Klinik herantrat, um die Prüfung seiner Insulinschockmethode zu erreichen, war es Hoff, der ihn mit mir bekanntmachte und dann gemeinsam mit mir und ihm die entsprechenden Einrichtungen auf der Klinik organisierte." Vgl. auch GABRIEL, Hans Hoff (2019), S. 344.

[650] Zur Verbreitung bereits in der 2. Hälfte der 1930er Jahre u. a. mehrere der Beiträge im Sammelband von SCHMUHL/ROELCKE (Hrsg.), „Heroische Therapien" (2013) – so BORCK, Die Internationale der invasiven Therapien (2013), S. 131-148, in dem es zentral um die rasche Verbreitung der Insulinkomatherapie im Rahmen des Erfolgs auch der anderen in den 1930er Jahren entwickelten ‚großen' körperlichen Therapien („Schocktherapien") Cardiazol- und Elektrokrampftherapie geht, sowie von GERMANN, Ein Insulinzentrum auf dem Land (2013), S. 149-167. Vgl. auch KAPLAN. A history of insulin coma therapy in Australia (2013), S. 587.

beigetragen haben[651] sowie die positive Einstellung der Psychiater gegenüber körperlichen Behandlungsmethoden, die sich seit der Einführung der Malariafiebertherapie entwickelt hatte. „Die Erfindung Dr. Sakel's" habe, so Hans Hoff 1952 an das Nobelkomitee, „in einer gewissen Periode der Psychiatrie eine Bresche geschlagen in den therapeutischen Nihilismus gegenüber von Psychosen."[652] Neben den neuen Hoffnungen und dem daraus entstandenen „neuen professionellen Aktivismus" in der Psychiatrie nennt Cornelius Borck 2013 im Rückblick als „wichtige[n] Faktor bei der Durchsetzung der ‚Schockverfahren' […] deren intensive Evaluation allein am Maßstab klinischer Wirksamkeit"[653] – also an der empirischen Erfahrung am Krankenbett.

Bereits im Mai 1937 wurde beim Meeting der American Psychiatric Association in Pittsburgh mit großer Begeisterung über die Erfolge der Therapie berichtet. Borck schildert die Rolle des Leiters der Klinik in Münsingen (Kanton Bern, Schweiz) Max Müller, der sich „als neutraler und kritischer Beobachter" positionieren konnte und sowohl durch seine positive Beurteilung der Therapie in Studien[654], als auch durch seine in „betont sachlich[er]" Atmosphäre abgehaltene Tagung zum Thema 1937 und durch die vielen Besucher seiner Klinik wesentlich zur Verbreitung der Therapie beitrug.[655]

Die Bedeutung der Insulinkomatherapie in der internationalen psychiatrischen Diskussion des ersten Nachkriegsjahrzehnts war enorm.[656] Die 1952 erschienene Monographie zur Insulinbehandlung in der Psychiatrie von Max Müller nennt im Literaturverzeichnis ca. 1.000

---

[651] Joseph Wortis vom New Yorker Bellevue Hospital, der in Wien die Insulinkomatherapie kennen lernte, wird eine wichtige Rolle in den USA zugeschrieben: BORCK, Die Internationale der invasiven Therapien (2013), S. 141. SHORTER/HEALY, Shock Therapy (2007), S. 53 zitieren einen Brief von Sakel an Wortis von 1937, wonach bereits 17 psychiatrische Kliniken von Tokyo bis Polen die Komatherapie anwenden würden.

[652] Hans Hoff an das Nobelkomitee für Physiologie und Medizin, 11.10.[1952], Kopie im Josephinum Wien, NL-Hoff MUW-AS-006005-0015. Als Begründung für den therapeutischen Nihilismus bei Psychosen führte Hoff an, dass sie allein auf (nicht behandelbare) Erbanlagen zurückgeführt worden seien.

[653] BORCK, Die Internationale der invasiven Therapien (2013), S. 136. Borck stellt hier klar, dass er damit nicht behaupten würde, „die zeitgenössischen Daten würden heutigen Ansprüchen klinischer Metaanalysen entsprechen".

[654] HIFT, Zur weiteren Entwicklung der Insulintherapie (1952), S. 975: „Die Sammelstatistiken von Max Müller und von [Anton von] Braunmühl zeigten, daß die Remissionsquote der insulinbehandelten Patienten das Doppelte der Spontanremissionen überstieg. Bei ausgewähltem Patientenmaterial waren die Erfolge noch verblüffender."

[655] BORCK, Die Internationale der invasiven Therapien (2013), S. 139-141.

[656] Vgl. die Grafik von Joseph Wortis zu den Schwankungen der jährlichen Publikationszahlen zwischen 1935 und 1955 in MÜLLER, Grundlagen und Methodik der somatischen Behandlunggsverfahren in der Psychiatrie: Einleitung (1963), S. 385 (Einleitung zum Kapitel über die Somatotherapien), in der sich ein rapider Anstieg der Publikationen bis zum Beginn des Zweiten Weltkriegs (auf über 300 Publikationen), dann ein starker Abfall mit einer flachen Kurve ab ca. 1944 bis in die 1950er Jahre mit jährlich um 25 – 30 Publikationen zeigt.

Titel nur zu dieser Therapie und ihrem Umfeld.[657] Insgesamt bestand unter Psychiatern weitestgehend Konsens darüber, dass die Methode einen höheren Anteil auch an dauerhaften Remissionen bei Fällen von Schizophrenie, vor allem wenn PatientInnen erst seit kurzer Zeit erkrankt waren, erbringen würde und so bei diesen trotz der Gefährlichkeit[658] das Mittel der Wahl darstelle.[659] Ähnlich wie die Malariafiebertherapie und die Cardiazolkrampftherapie,[660] wurde auch die Insulinkomatherapie bei anderen psychischen Erkrankungen angewandt.[661]

Mit der Bedeutung der Einführung der „Schocktherapien"[662] – 1933 der Insulinkomatherapie durch Sakel, 1938 der Elektrokrampftherapie (EKT) durch Ugo Cerletti –, begründet Hans Hoff 1952 und nochmals 1957 seinen Vorschlag (zu dem er von der Nobelpreiskommission eingeladen worden war), Manfred Sakel und Ugo Cerletti gemeinsam 1953 bzw. 1958 den Nobelpreis für Medizin zu verleihen.[663] Denn „durch die Kenntnis der Schocktherapie" sei „die gänzliche Umstellung in der Psychiatrie von einer Wissenschaft, die sich mit rein deskriptiven Beschreibungen von Krankheitsbildern begnügte zu einem hochaktiven therapeutischen Teil der Medizin […] eingeleitet" worden. Es sei, schrieb Hoff, das „Verdienst Sakels gezeigt zu haben, dass eine Möglichkeit besteht, die Schizophrenie zu heilen" und „Verdienst von Cerletti, durch die Einführung der Elektroschockbehandlung das Los von Melancholikern […] verbessert zu haben".[664] 1957 bemerkte Hoff einleitend zu seinem Vorschlag: „Obwohl gerade jetzt Bestrebungen im Gange sind, die Schocktherapie bei der Behandlung der Schizophrenie und des manisch-deperessiven Irreseins zurückzudrängen",

---

[657] MÜLLER, Insulinbehandlung (1952), S. 251-281.
[658] Vgl. zu den Komplikationen, wie den Gefahren des protrahierten Komas, von epileptischen Manifestationen und bei Nichtbeachtung der Kontraindikationen: HOFF, Lehrbuch der Psychiatrie (1956), S. 507-509. Hier auch zum „heroischen Versuch [von Werner (Verners) Kraulis] des absichtlichen protrahierten Comas […] bei verzweifelten Fällen". Vgl. auch die Publikation der Wiener Kliniker ZEITLHOFER/TSCHABITSCHER/WANKO, Zur Pathologie des protrahierten Insulinschocks (1954), S. 445-458.Vgl. zu den Komplikationen auch die Bleuler- und Arnold-Zitate unten S. 194-196.
[659] Vgl. HIRSCHMÜLLER, Die Insulinkomabehandlung der Schizophrenie (2001), S. 222.
[660] Vgl. dazu oben S. 39 Anm. 123 das Zitat aus MICHAUX, Psychiatrie (1965), S. 1002.
[661] Vgl. COFFIN, French Biological Therapeutics (2013), S. 193f. zur Situation um 1940: "Most French psychiatrists chose to administer insulin therapy and cardiazol therapy not only to schizophrenic patients. For example, manic and depressive disorders as well as mentally confused states were considered eligible." Vgl. CLAUDE/RUBINOVITCH, Thérapeutiques biologiques des affections mentales (1940), S. 124f.: die Indikation sei ‹nicht nur auf das schizophrene Syndrom› beschränkt; Henri Claude vertrat die Meinung, dass bei allen diesen Diagnosen je nach der Reaktion des Patienten, sehr unterschiedliche Insulindosen, mit oder ohne Koma anzuwenden seien.
[662] Zur Verwendung des Begriffs „Schocktherapie" im Text vgl. oben S. **27** Anm. 76.
[663] Hans Hoff an das Nobelkomitee, 11.10.[1952], Kopie im Josephinum Wien, NL-Hoff MUW-AS-006005-0015; Hans Hoff an das Nobelkomitee, 19.10.1957, Kopie im Josephinum Wien, NL-Hoff MUW-AS-006005-0011-068 Zl. 68/5.
[664] Zitat aus Hans Hoff an das Nobelkomitee, 19.10.1957, Kopie im NL-Hoff MUW-AS-006005-0011-068 Zl. 68/5; vgl. unten S. 240 Anm. 921 und – aus dem Schreiben vom 11.10.[1952] zur EKT – S. 302 Anm. 1145.

mache er seinen Vorschlag. Er spielt damit auf deren Ersatz durch medikamentöse Therapien an.

In den Jahren um diese zweite Nominierung Manfred Sakels durch Hans Hoff regte sich aber bereits zunehmend Skepsis gegenüber der Insulinkomatherapie. Vorerst waren kritische Stimmen wie jene von Harold Bourne, der 1953 im *Lancet* von einem „insulin myth" sprach,[665] überwiegend auf Ablehnung gestoßen. In den folgenden Jahren wurden die Skeptiker jedoch zahlreicher. Als 1957 anlässlich des 30-Jahres-Jubiläums der Insulinkomatherapie (Sakel selbst gab an, 1927 erstmals einen an Schizophrenie leidenden Patienten mit Insulinkoma behandelt zu haben) an der Wiener psychiatrisch-neurologischen Klinik unter der Leitung Hoffs ein Symposium mit einem Vortrag von Sakel veranstaltet wurde, dürfte hier die Kritik noch sehr zurückhaltend gewesen sein.[666]

Zwei in *The Lancet* publizierte Studien sind als Beispiele für die beiden Tendenzen zu nennen, die international zur sukzessiven Aufgabe der Insulinkomatherapie beitrugen: Beide verglichen randomisierte Gruppen von Schizophrenen miteinander. Die eine von 1956 verglich die Erfolge der Behandlung durch Insulinkomatherapie mit jenen durch Chlorpromazin, kam zwar nur zu statistisch nicht signifikant besseren Ergebnissen beim Neuroleptikum, empfahl aber dennoch dieses als weniger gefährlich und aufwendig vorzuziehen.[667]

Anders die Studie in *The Lancet* im folgenden Jahrgang 1957 von B. Ackner, A. Harris und A. J. Oldham. Auch sie verwiesen auf Studien der letzten Jahre, die sich kritisch gegenüber dem Wert der Insulinbehandlung bzw. (wie Boardman et al.) für einen Ersatz durch die

---

665 BOURNE, The Insulin Myth (1953), S. 964-968; er kritisierte scharf die Erstellung der Vergleichsdaten und Vergleichsgruppen aus unterschiedlichen Perioden und Spitälern in den bisherigen Publikationen, die unterschiedliche und unklare Diagnostik und die Vernachlässigung von Faktoren, wie des Einflusses des persönlichen Umfelds und des Umfelds während der Behandlung; „there is no sound basis for the general opinion (as stated, for example, in a refresher course by Sargant and Slater in 1951) that insulin-coma therapy counteracts the schizophrenic process in some specific manner ". Vgl. zur Kontroverse über Bournes Artikel, zu seinen Kritikern und seinen Unterstützern, FREUDENTHAL/MONCRIEFF, ‚A landmark on psychiatric progress'? The role of evidence in the rise and fall of insulin coma therapy (2022), S. 69-71.

666 GRÖGER, Insulin-Schocktherapie (2005), S. 219 zitiert Professor Manfred Sakel of New York Celebrated in Vienna (1957), S. 10-11. Es gab keinen Sammelband der Vorträge und Diskussionen dieser Tagung. Vgl. SHORTER, Geschichte der Psychiatrie (1999), S. 315 (Foto von Manfred Sakel 1957 in Wien mit Hans Hoff und Otto Pötzl).

667 BOARDMAN/LOMAS/MARKOWE, Insulin and Chlorpromazine in Schizophrenia (1956), S. 487-491, hier S. 490; freilich wird hier auch betont, dass noch keine Ergebnisse aus mittelfristigen Nachuntersuchungen vorhanden seien.

weniger gefährliche und aufwendige Chlorpromazin-Behandlung aussprachen. Dann nannten Ackner et al. „Trugschlüsse“ bzw. Fehlerquellen der bisherigen Untersuchungen: in der Auswahl der Patienten (häufig mit unbeachtet unterschiedlichen Schizophrenie-Diagnosen), in der Definition der Ergebnisse der Therapie, der Erstellung der Kontrollgruppe etc. Daraufhin beschrieben sie die Vorgangsweise ihrer Studie, um diese Fehler zu vermeiden; die Studie kann wohl auch heute noch als vorbildlich in der Auswahl und randomisierten Zuteilung der Patienten zu den beiden Gruppen gelten, der Gestaltung des therapeutischen Umfelds und der Beurteilung des Erfolgs in den Jahren nach der Behandlung durch einen Arzt, der nicht wusste, welche Therapie angewandt worden war:[668] 25 Paare (50 PatienInnen) zeigten, dass die Ergebnisse der Kontrollgruppe, die mit Barbituraten statt mit Insulin in einen Koma-ähnlichen Zustand gebracht wurden, ‚so deutlich übereinstimmten, dass keine Auswertung statistischer Signifikanz notwendig war’. Falls es zu einem therapeutischen Erfolg der Kur käme, so nicht aufgrund des Insulins, sondern aufgrund der Verbesserung der Beziehung des / der Patienten/in zu Arzt / Ärztin und Pflegepersonal durch die aufwendige Pflege während der intensiven Kur.[669]

Das Ergebnis der überzeugend durchgeführten klinischen Studie zur Insulinkomatherapie von Ackner, Harris und Oldham im international bekannten Publikationsorgan, dass es keine Insulin-korrelierte Überlegenheit gegenüber der Anwendung von Barbituratnarkosen gäbe, führte jedoch allgemein nicht rasch zum Ende der Therapie. Dafür sind mehrere Gründe anzunehmen: es gab Studien, die weiterhin von Erfolgen der Insulinkomatherapie berichteten,[670] und PsychiaterInnen vertrauten aufgrund ihrer positiven Erfahrungen weiterhin der eingeführten Therapie. So schrieb der britische Psychiater William Sargant – ein entschiedener Befürworter der Insulin- und anderer somatischer Therapien – 1957 in einem ‘Brief an den Herausgeber’ von *The Lancet*, nachdem hier mehrere, teils kritische Artikel über den Wert der Insulintherapie erschienen waren: Die Strafe für eine Fehlentscheidung „in the treatment of a case of early recoverable schizophrenia, and the omission of any treatment

[668] Bei der in Wien angewandte Methode des ‚Simultanvergleichs‘ war hingegen die Vorgangsweise auf einen Arzt und dessen Einschätzung aufgrund seiner klinischen Erfahrung konzentriert: s. unten S. 188 Anm. 722 und 723.

[669] Die Bedeutung dieses Erfolgs wird von ACKNER/HARRIS/OLDHAM, Insulin Treatment of Schizophrenia (1957), S. 611 nicht ausgeführt, aber mehrmals von anderen Psychiatern als Öffnung des / der Patienten/in für die Kommunikation, für eine psychotherapeutische Behandlung beschrieben: Vgl. die Zitate von Bleuler allgemein zu den „Erschütterungstherapien“ oben S. 29-31, von Hoff unten S. 200 (wonach „die Insulinkur uns eine Art Eingangspforte zur Psyche des Patienten eröffnet“), und von Juillet unten S. 184f.

[670] SCHIPOKOWENSKY, Die Behandlung der Schizophrenie und der Zyklophrenie mit Heilkrampf, Insulinschock und Chlorpromazin (1960), S. 745.

helping to bring about recovery, may mean thirty years of intense suffering in the back wards of a mental hospital for the individual patient concerned. Few decisions on treatment in medicine, therefore, cary such disastrous sequelæ if they happen to be the wrong ones.“[671]

Der Medizinhistoriker Albrecht Hirschmüller kommt in seiner 2001 veröffentlichten Studie zur Einschätzung, dass die Ablösung der Insulinbehandlung „an den meisten Orten innerhalb weniger Jahre von der neuroleptischen Therapie [Ende der 1950er und Anfang der 1960er Jahre, GH] […] mehr mit Zeitströmungen und mit den inzwischen vorliegenden Alternativen [den Neuroleptika, GH] als mit der angeblich oder tatsächlich erwiesenen Unwirksamkeit der Methode zusammenhing“.[672] Denn, so Hirschmüller, „die inneren Kräfte, die in Therapeuten wirksam sind, sie zu heroischen oder verzweifelten Therapieversuchen antrieben oder sie ihre Hilflosigkeit erleben und erleiden lassen, sind von anderer Art als die Kraft so genannter objektiver kritischer Studien.“[673]

Im September 1957 fand in Zürich der 2. Internationale Kongress für Psychiatrie statt. Nach William Sargant in *The Lancet* wurden die Veränderungen bei mehreren der somatischen Behandlungsformen in der Psychiatrie besprochen. Die Insulinkomatherapie sei nach Meinung aller Vortragenden, die aus vielen verschiedenen Ländern kamen, bei bestimmten schizophrenen PatientInnen[674] zu empfehlen und selbstverständlich jetzt noch nicht aufzugeben. Bei manchen PatientInnen, die vorher mit der Insulinkomatherapie behandelt worden wären, hätten nach Erfolgen mit sedierenden Medikamenten, diese nun die Komakur ersetzt. Die Elektrokonvulsionstherapie habe jedoch niemand für geeignet gehalten, die Komatherapie zu ersetzten.[675] Ein Jahr später folgte in New York unter internationaler Beteiligung eine Konferenz, organisiert von der Sakel-Stiftung (Manfred Sakel war Ende

---

671 SARGANT, Insulin in early schizophrenia (Letters to the editor) (1957), S. 644.
672 HIRSCHMÜLLER, Die Insulinkomabehandlung der Schizophrenie (2001), S. 217.
673 Ebd., S. 225. FREUDENTHAL/MONCRIEFF, ‚A landmark on psychiatric progress‘? The role of evidence in the rise and fall of insulin coma therapy (2022), S. 65f., fassen die Meinungen zusammen, die in neueren britischen Publikationen über die Ursachen, die zur Beendigung der Insulinkomatherapie geführt haben, vertreten werden – jene, die der methodischen Weiterentwicklung der evidenzbasierten Medizin mit dem Meilenstein der oben zitierten randomisierten kontrollierten Studie von Ackner/Harris/Oldham 1957 die Hauptrolle beimessen und jene, die sie der Einführung der neuen Psychopharmakatherapien als den leichter anzuwendenden und billigeren ‚natürlichen Nachfolgerinnen‘ der Insulinkomatherapie zuschreiben.
674 Damit waren – wie allgemein in der Literatur – wohl die PatientInnen kurz nach dem Ausbruch der Krankheit gemeint.
675 SARGANT, Insulin in early schizophrenia [letter to the editor] (1957), S. 644 f. Der prominente britische Psychiater war ein Befürworter der Insulinkomatherapie.

1957 gestorben), bei der die meisten Autoren der Meinung waren, dass ihre Anwendung zugunsten der Neuroleptika weiter abnehmen würde.[676]

Eine Diskussion in *The Lancet* 1961 lässt den Schluss zu, dass die Insulinkomatherapie auch in England Anfang der 1960er Jahre noch angewandt wurde. A. D. Collins, eine Ärztin im Mental Hospital Ilkley (Yorkshire), berichtet 1961 darüber, dass sie im letzten Jahr für zwei Patienten, die mit den neuen Medikamenten nicht zu therapieren gewesen seien, vergeblich ein Spital mit einer Insulinstation gesucht habe.[677] Rolf Ström-Olsen vom Runwell Hospital in Wickford (Essex) antwortete daraufhin, dass in seinem Krankenhaus die Insulintherapie seit 1937 durchgehend angewandt werde. Da der Personalaufwand nicht besonders hoch sei (2 – 3 Krankenschwestern für 6 – 8 Patienten) ermunterte er Collins, in ihrem Krankenhaus eine eigene Insulinstation zu etablieren, andernfalls würde er aber auch gerne Patienten von ihr zur Insulinkur aufnehmen.[678] Ström-Olsens Replik liest sich, als ob er ‚die letzte Bastion' in England halten würde.

Dass die Entwicklung der Psychopharmaka, vor allem der Neuroleptika, auf die Zurückdrängung der Insulinkomatherapie hinauslaufen würde, kam also im internationalen Diskurs früh zur Sprache, wenn auch die beiden therapeutischen Konzepte zur Behandlung der Schizophrenie an mehreren psychiatrischen Krankenhäusern – wie in Wien bis um 1970 – parallel und in Kombination zur Anwendung kamen.[679] Nach dem Wiener Kliniker Stefan Hift war die Insulinkur auch 1961 „noch eine der erfolgreichsten Therapien der beginnenden schizophrenen Psychose"; sie sei jedoch „vorwiegend" wegen der „relativen Kompliziertheit der Technik", der „Häufigkeit von Komplikationen"[680] und der „langen Dauer der Kur" (mit hohen Kosten) „an vielen Stellen zugunsten der Elektroschockbehandlung oder

---

[676] EY/BERNARD/BRISSET, Manuel de Psychiatrie ([1]1960), S. 936.
[677] COLLINS, Insulin Therapy (Letters to the editor) (1961/II), S. 1457.
[678] STRÖM-OLSEN, Insulin Therapy (Letters to the editor) (1962), S. 47.
[679] Die am Ende des Untersuchungszeitraums aufgenommene 15jährige Patientin S/A20888 bekam 1969/70 bei ihrem zweiten Aufenthalt von 4 Monaten mit der Diagnose „M[orbus] Bleuler" in den ersten 12 Tagen eine EKT mit 6 Anwendungen, an den ersten 4 Tagen 3x 50mg. Truxal i. m. Vom 5. – 18. Tag bekam sie 3x 10gtt Triperidol (sehr potentes Neuroleptikum Trifluperidol) und – gegen die unerwünschten Nebenwirkungen – 3x 1/2 Kemadrin (Anticholinergikum Procyclidin); diese Dosierung wurde bis zum 29. Tag langsam verringert und beendet. Am 19. Tag wurde mit einer Insulinkomatherapie mit 70 Komata und insgesamt 6 E-Schocks im Koma begonnen; geweckt wurde immer mit 1,5mg Glucagon i. m. Parallel zur Insulintherapie wurden 2x 5gtt des hochpotenten Neuroleptikums Haloperidol (aus der Gruppe der Butyrophenone) gegeben; zum Haloperidol vgl. S. 184, S. 220 Anm. 860 und S. 288 Anm. 1088. Nach der Insulintherapie sind auf der Fieberkurve mehrmals Arbeitstherapie und Ausgang eingetragen. Sie wurde ins Rehabilitationszentrum Maria Lanzendorf entlassen, wo ev. die in der Wiener Literatur angeführte psychotherapeutische Behandlung (Gruppentherapie) stattfand, die in der zweiten Hälfte der 1960er Jahre lt. Zeitzeugen an der Klinik nicht praktiziert wurde (vgl. S. 104 Anm. 365).
[680] Zu schweren Zwischenfällen bei der Insulinkomatherapie und ihrer Beurteilung, s. unten S. 194-196.

medikamentöser Kuren aufgegeben“ worden.[681] Ein Faktor, der nach Albrecht Hirschmüller neben der Einführung der Neuroleptika und der Kritik dazu beitrug, die Insulinkomatherapie aufzugeben, waren die „parallel laufenden Entwicklungen sozialpsychiatrischer Sichtweisen“, durch die die Heilwirkung des Milieus in den Vordergrund der Schizophreniebehandlung gestellt wurde; für „martialische Behandlungsweisen“ wie die Insulinkomatherapie sei dabei „wenig Raum“ geblieben.[682]

Nichtsdestoweniger wurde die Insulinkur nicht nur 1963 im Beitrag von Max Müller im mehrbändigen Sammelband „Psychiatrie der Gegenwart: Forschung und Praxis“[683], sondern auch im Bleuler'schen Lehrbuch der Psychiatrie, noch in der 11. Auflage von 1969 als eine mögliche Therapie und in mehreren Anwendungen beschrieben. (Im ‚Burghölzli“, der von Manfred Bleuler geleiteten psychiatrischen Klinik der Universität Zürich, wurde zu dieser Zeit die Insulinkur jedoch kaum oder gar nicht mehr angewandt[684]). Nach einer ausführlichen Beschreibung der Anwendung der Insulinkomatherapie im Kreis anderer „'großer' körperlicher Kuren'“ (wozu nun auch Kuren „mit neuroleptischen Mitteln“ gezählt wurden) heißt es hier, zwar seien „Schlafkuren mit Barbituraten, Insulinkuren und Elektroschockkuren […] seit der Einführung der neuroleptischen Mittel seltener angezeigt als früher“, die Insulinkur werde aber noch „von vielen als Behandlung der Wahl bei Schizophrenen angesprochen, während sie von anderen ganz aufgegeben worden“ sei. Manfred Bleuler betonte hier auch die von ihm wiederholt genannte Wirkung der ‚großen körperlichen Kuren', indem er – ähnlich wie im obigen Zitat Ackner et al. 1957 – schrieb: „In anderen Fällen kann die lange Anlaufzeit der Insulinkur gerade günstig sein, u. a. deshalb, weil die Zeit für die Anknüpfung einer psychischen Bindung zwischen dem Kranken und Ärzten oder Schwestern wertvoll ist. Das gilt bei Schizophrenen besonders oft.“[685] Bleuler empfahl 1969 in

---

[681] HIFT, Die Ganglienblockade bei der Insulinkur in der Psychiatrie (1961), 430.
[682] HIRSCHMÜLLER, Die Insulinkomabehandlung der Schizophrenie (2001), S. 224 nennt Rudolf Karl Freudenberg, der in Wien Mitarbeiters von Sakel gewesen war, als „Kronzeuge[n] für diese Entwicklung“, und zitiert BECKER/BENNET, Rudolf Karl Freudenberg – from pioneer of insulin treatment to pioneering social psychiatry (2000), passim. Becker und Bennet argumentierten in ihrem *abstract* (S. 189), Freudenbergs „shift in professional interest from physical to social intervention“ spiegle wichtige allgemeine Trends, „i.e. profound change due to new forms of physical treatment on the one hand and similarly convincing evidence of the impact of social factors on clinical course and outcome on the other hand.“
[683] MÜLLER, Die Insulinbehandlung (1963), S. 389-414. Zu ihm als Propagator der Insulinkur vgl. oben S. 174f.
[684] TANNER/MEIER/HÜRLIMANN/BERNET, Zwangsmassnahmen in der Züricher Psychiatrie (2002), S. 93-95 (Grafik zur Anwendung der Kuren) und die Information von Eberhard Gabriel, der 1966 am 'Burghölzli' gearbeitet hat.
[685] BLEULER, Lehrbuch der Psychiatrie ([11]1969), S. 165. Ähnlich BLEULER, Lehrbuch der Psychiatrie ([10]1960), S. 405.

bestimmten Fällen schizophrener Erkrankungen auch die Insulinkur: „Eine der bedeutsamsten Anzeigen für die Insulinkur sind subakute paranoide Schizophrenien, die in der therapeutischen Klinikgemeinschaft nicht bessern wollen."[686] Zu den „'endogenen' Geistesstörungen" wurde zuerst die Anwendung der Neuroleptika beschrieben, dann aber auch die Anwendung der älteren Kuren als aktuell genannt; so heißt es zur Insulinkur: „In schweren Fällen können auch Elektroschock-, Insulin- und Schlafkuren manchmal bessern oder wenigstens vorübergehend Beruhigung und Erleichterung schaffen. Selten einmal schließt sich die Heilung an solche Kuren an."[687] Es ging – wie in der Praxis der Wiener Klinik – fast ausschließlich um schizophrene Erkrankungen: „Insulinkuren bessern Depressionen nur ausnahmsweise."[688] Und im Kapitel „'Psychoreaktive' oder ‚psychogene' Störungen" erwähnt Manfred Bleuler: „Bei schwersten Angstzuständen [hier aufgrund von Zwangsstörungen, GH] kommen Dämmerkuren mit Neuroleptica oder Schlafmitteln oder Insulinkuren in Frage."[689] In der Auflage von 1960 waren die Hinweise auf die Insulinkur ähnlich und auch in dieser Auflage ging es bereits mit einem gewissen Vorrang um die Anwendung der ‚neuen' Kuren mit Neuroleptika.[690] Nach dem Grundsatz, dass immer „die ungefährlichste Kur gewählt werden [soll], die wirksam ist", waren dies 1960 „diejenige mit Phenothiazinen und ähnlich wirkenden Körpern. […] Hat sie sich als undurchführbar oder unwirksam erwiesen, so konkurrieren nach ihr an Gefährlichkeit die Krampfverfahren und die Insulinkur. Bei Kranken, bei denen Hoffnung auf Wiedererlangen der vollen geistigen Kräfte besteht, besonders bei Intellektuellen, scheint mir die Gefahr des amnestischen Psychosyndroms nach Krampfbehandlung so gewichtig, daß die Insulinkur vorzuziehen ist."[691]

1960 brachten Henri Ey, P. Bernard und Ch. Brisset in ihrem renommierten Handbuch der Psychiatrie eine Zusammenfassung der kontroversen Diskussion der letzten 25 Jahre und

---

[686] BLEULER, Lehrbuch der Psychiatrie ([11]1969), S. 160-166. Ähnlich ebd., S. 422.
[687] Ebd., S. 447; ebenso BLEULER, Lehrbuch der Psychiatrie ([10]1960), S. 426. Zur Indikation vgl. auch die Zitate aus dem Bleuler-Lehrbuch in den Ausgaben von 1955, 1960 und 1969 oben S. 28f.
[688] BLEULER, Lehrbuch der Psychiatrie ([11]1969), S. 450.
[689] BLEULER, Lehrbuch der Psychiatrie ([11]1969), S. 495. Im Text ist nicht klar ausgedrückt, ob er in diesen Fällen Komata oder Subkomata meint, die auch in Wien zur Beruhigung von PatientInnen mit affektiven Störungen angewandt wurden.
[690] So hieß es etwa in BLEULER, Lehrbuch der Psychiatrie ([10]1960), S. 470, im Kapitel „'Psychoreaktive' oder ‚psychogene' Störungen": „In Zuständen von starker, angsterfüllter Spannung und Erregung können körperliche Kuren mildern und bessern. Bisher versuchte man vor allem Insulin- und Schlafkuren, heute sind Kuren mit Chlorpromazin oder ähnlichen Präparaten zuerst anzuwenden."
[691] BLEULER, Lehrbuch der Psychiatrie ([10]1960), S. 160; und weiter heißt es hier: Hingegen sei etwa bei schizophren dementen Patienten ein leichtes amnestisches Psychosyndrom nach Krampfbehandlung bedeutungslos (vgl. dazu auch ebd., S. 155f.) Am gefährlichsten schätzt Bleuler die Schlafkur ein.

insbesondere der letzten Jahre zur Insulinkomatherapie mit dem Schluss, dass der Insulinschock wenigstens bei 20-25% der Schizophrenen, die nach einer Kur das Spital verlassen, die Chance bringe, auf mehrere Jahre ohne Rückfall zu bleiben.[692] Auf die Anwendung der Insulinkomatherapie war nach ihrer Meinung vorläufig noch nicht zu verzichten. 1965, im französischen Handbuch der Psychiatrie von Léon Michaux hieß es, dass Sakels großes Verdienst darin bestand, in einer Zeit, als eine psychiatrische Therapeutik quasi inexistent gewesen sei, eine der ersten biologischen Techniken eingeführt zu haben, dass man aber nun, 30 Jahre nach der ersten Anwendung immer noch im Unklaren über den exakten Wert der Therapie sei.[693] Nach dem Hinweis auf die Studien, die von sehr geringen Erfolgen bzw. über die Erfolglosigkeit der Insulinkomatherapie berichten und nach Ausführungen über die Schwierigkeiten einer Erfolgsbeurteilung, spricht aber auch Michaux der Therapie nicht ihre Berechtigung ab – er erwähnt „eindrucksvolle Resultate bei gewissen schizophrenen Erkrankungen und bei schleppend verlaufenden melancholischen Depressionen, die dem Imipramin [Dibenzazepine, trizyklisches Antidepressivum] und der EKT widerstanden“. Eine realistische Einschätzung der Wirkung der Insulintherapie sei auch insofern praktisch unmöglich geworden, als man sie in den letzten Jahren systematisch mit starken Dosen von Chlorpromazin oder anderen Neuroleptika kombiniere, wodurch die Ergebnisse offensichtlich viel besser seien. So öffne sich ein neues Feld, in dem das Insulin mit Psychotropen kombiniert durch viel niedrigere Dosen seine Gefährlichkeit verliere und einen genau definierten Platz gewinne, indem es Veränderungen der zellulären Permeabilität begünstigt[694] und so vielleicht eine andere und wirksamere chemotherapeutische Behandlung ermöglicht.[695]

Pierre Pichot, Professor im Centre Psychiatrique St. Anne (der Universitätsklinik) in Paris und häufiger Gast an der Klinik Hoff, publizierte 1960 einen Artikel in der *Wiener Medizinischen Wochenschrift*, in dem er Probleme aufzeigt, die zu den konträren Bewertungen „der wichtigsten biologischen Behandlungsmethoden in der Psychiatrie“ – „E-Schock, Insulin-Schock, Behandlung mit verschiedenen Neuroleptika allein“ – führten:

---

[692] EY/BERNARD/BRISSET, Manuel de Psychiatrie ([1]1960), S. 936f. Sie zitieren in ihrer Zusammenfassung der Diskussion (ohne Literaturhinweis!) u.a.: « H. Hoff et O.H. Arnold (1958) assurent que 81% de leurs cas de schizophrénies confirmées ont pu être considérés comme guéris après 5 ans grâce à cette thérapeutique.» Die hier zitierte Publikation von 1958 konnte nicht identifiziert werden. Sehr positive Ergebnisse (mittelfristige Erfolge) nennen HOFF/ARNOLD, Die Therapie der Schizophrenie (1954), S. 348, und 1955 in französischer Übersetzung diese Publikation: HOFF/ARNOLD, Au sujet de la thérapie de la schizophénie (1955), S. 12.
[693] MICHAUX, Psychiatrie (1965), S. 998: immer noch «‘entre un enthousiasme démesuré et un scepticisme conservateur‘ comme le rappelle Ey.»
[694] « á la faveur des modifications de la perméabilité cellulaire ».
[695] MICHAUX, Psychiatrie (1965), S. 1001.

- Die Wirkmechanismen dieser Therapien seien noch immer zu unklar, um daraus die Indikation abzuleiten.[696]
- Auch die angewandten „Kriterien der Besserung" und selbst die Behandlungsmethoden seien zu unterschiedlich für eine Vergleichbarkeit.
- Die Zahlen aus den Studien zur Wirksamkeit der einzelnen Behandlungsmethoden seien kaum vergleichbar, da „genaue diagnostische Kriterien in der Psychiatrie" fehlten. Die Schizophrenie weise etwa so unterschiedliche Verlaufsformen auf, dass der Zustand eines Patienten / einer Patientin nicht eindeutig mit einer vorangegangenen Therapie zu korrelieren sei, besonders wenn ein größeres Zeitfenster dazwischen lag.

So würden „in den drei letzten bedeutendsten Studien über die Prognose der Schizophrenie […] die prozentualen Angaben über die Spontanheilungen und die sozialen Remissionen[697] […] um das Doppelte" variieren. Ein Vergleich der Besserungen in den Krankheitsbildern von PatientInnen nach verschiedenen Behandlungen war nach Pichot nur schwer zu eruieren, denn „objektive Kriterien der Wirksamkeit" ebenso wie darauf aufbauende vergleichende Studien stünden noch aus.[698]

Zu den vorliegenden Studien zur Insulinkur nennt Pichot die oben zitierte radikale Kritik von Bourne 1953 und eine weitere Publikation von Bourne von 1958[699] mit der Aussage, dass „die Erfolge (soweit vorhanden) der biologischen Therapieformen [im Allgemeinen, GH] der Tatsache zu verdanken" sei, „daß die Ärzte dadurch zu einer positiven Haltung den Kranken gegenüber kamen und, ohne es zu wissen, eine Psychotherapie ausüben, teils direkt, teils indirekt durch die Veränderung des Milieus."[700] „Eine solche Auffassung erscheint paradox", schrieb dazu Pichot, denn zweifelsohne sei das Milieu wichtig, „aber auch wenn wir diese Umstände berücksichtigen, können wir vernünftigerweise doch nicht den biologischen Wirkmechanismus dieser Behandlungen verneinen." Als bisher einzigen kontrollierten Blindversuch über die Wirkung des Insulinkomas nennt er den Vergleich mit einem Barbituratkoma von Ackner, Harris, Oldham (1957), der oben ausführlich zitiert wird. Er nennt auch das Ergebnis, dass die beiden Methoden eine identische Wirkung bei der

---

696 PICHOT, Vergleich der verschiedenen Behandlungsmethoden in der Psychiatrie (1960), S. 734. Als Gründe nannte er die „Unkenntnis der genauen Pathogenese psychischer Erkrankungen" und dass die „wesentlichsten Erkenntnisse über die Behandlungsmethoden […] rein empirischer Natur" seien.
697 Also soziale Anpassung, kurz oder dauerhaft, aber nicht Heilung, nicht völlige Genesung.
698 PICHOT, Vergleich der verschiedenen Behandlungsmethoden in der Psychiatrie (1960), S. 736.
699 BOURNE, Insulin coma in decline (1958), S. 1015-1017.
700 Diese Perspektive unterscheidet sich in der Betonung der ärztlichen Haltung von jener von ACKNER/HARRIS/OLDHAM.: vgl. oben S.177.

Schizophrenie zeigen, weist freilich im Text darauf hin, dass auch „kontrollierte Untersuchungen nicht immer die Sicherheit [bieten], die sie versprechen“. Seine Hoffnung setzt er in weitere sorgfältig vergleichende Studien.

Die zunehmende Bevorzugung der neuen Psychopharmaka vor allem gegenüber der Insulinkomatherapie, aber auch gegenüber der EKT[701] schrieb Pichot der „Einfachheit der Anwendung“ von Medikamenten zu und merkte an, dass Faktoren wie leichtere Verabreichung, höhere Akzeptanz bei den Angehörigen und geringerer Personalaufwand wohl eher den Ausschlag gegeben hätten als eine „objektive Überlegenheit“ der medikamentösen Therapie.

Im Vergleich zu den Wiener Klinikern Arnold und Hift sowie zum Klinikleiter Hoff[702] hatte für Pichot in seiner persönlichen Einschätzung und Praxis die Insulinkomatherapie bei Schizophrenie einen deutlich eingeschränkteren Stellenwert, wenn er auch die Abnahme ihrer Anwendung hauptsächlich auf die leichtere Anwendung der Medikamente zurückführte. Für unbedingt notwendig scheint er ihre Indikation nur bei den hebephrenen Formen gehalten zu haben und auch hier setzte er Hoffnungen auf die positiven Ergebnisse der Entwicklung der neuen Psychopharmaka wie Haloperidol, um die massiv intervenierende Therapie ersetzen zu können. Auch in Wien kam es durchgehend zu einer deutlichen Abnahme der Anwendungen der Insulinkomatherapie[703] durch eine Spezifizierung der Indikation auf bestimmte schizophrene Erkrankungen und – besonders in den 1960er Jahren – auch durch die neuen Neuroleptika. Diese wurden alleine nur in Fällen gegeben, in denen die erste Episode länger als 1 ½ Jahre zurücklag, in frühen Fällen aber auch in Kombination mit der Insulinkomatherapie.[704]

Pierre Juillet (vom Militärspital Val-de-Grâce in Paris) publizierte mehrfach zur Insulintherapie. In einem Enzyklopädie-Artikel zur Insulintherapie 1964 gemeinsam mit Roger Dorey werden Anwendungen der Insulinkomatherapie außerhalb ‹schizophrener Zustände› genannt: zu diesen nicht schizophrenen Erkrankungen zählen sie (nach der französischen diagnostischen Tradition, der diese enger als die deutschsprachige Psychiatrie

---

[701] Vgl. dazu auch unten im Kapitel zur EKT S. 256f. Die hier genannten Anwendungen der EKT zeigen Übereinstimmungen mit der Meinung der Wiener Kliniker.
[702] Pichots Freund Peter Berner dürfte bereits nun weitgehend auf dessen Seite gestanden haben.
[703] Vgl. unten Abb. 8 S. 211.
[704] Vgl. unten S. 283.

fasst) die Gruppe der ‹chronischen Wahnerkrankungen›; bei diesen würde meistens die Insulinkomakur – manchmal mit Elektro- oder Cardiazolkrampftherapie kombiniert – angewandt, obwohl hier auch Neuroleptika gut wirken.[705] Bei akut wahnhaften Psychosen, die eine sehr gute Indikation für Neuroleptika und eventuell auch für die Elektrokrampftherapie sind, könne es aussichtsreich sein, entweder geringer Dosen von Insulin, oder einige (wenige) Insulinkomatherapien anzuwenden. Sie nennen auch die Anwendung in bestimmten Fällen von affektiven Psychosen, bei ‹chronischen oder remittierenden Manien, widerspenstig gegenüber anderen Behandlungen›, bei ‹atypischen Melancholien bei Jugendlichen, die mit Thymoleptika oder Elektrokramptherapie nicht bessern›. Es folgen weitere, von einzelnen Autoren empfohlene Anwendungen etwa bei einer Verschlechterung der geistigen Fähigkeiten durch Alkoholismus, bei Hellerscher Demenz, sowie bei bestimmten schweren neurotischen Zuständen; letztere seien den beiden Autoren zufolge manchmal auch ein Anwendungsgebiet der Insulinsubkomatherapie, mit Gaben von Insulin in Dosen, die nicht zum Koma führen.[706] Die Anwendungen der (verkürzten) Koma- und der Subkomatherapie bei affektiven Störungen findet sich auch in den Krankenakten der Wiener Klinik und wird unten in Unterkapiteln zu ihrer Auswertung besprochen.

Vier Jahre später, 1968, schrieb Juillet in einem Artikel zu Koma- und Krampftherapien bei Schizophrenie, die Sakel-Kur sei im Vergleich mit den anderen ‚Schock'-Therapien die am meisten angewandte,[707] aber auch die am meisten diskutierte Therapie.[708] Die Indikationsgebiete hätten sich zunehmend verengt und verschoben und die Komatherapie sei nun Indikation bei den ‹einfachen und hebephrenen Formen und allgemein bei den Formen, die mit den medikamentösen Therapien nicht bessern. Auf diese Weise ist diese Therapie eine ergänzende Methode, wenn sich akute oder subakute Schübe nicht abschwächen und beruhigen.[709] Vor allem Autismus und Dissoziation («la discordance») seien mit Neuroleptika kaum behandelbar. Im Resümee betonte Juillet (wie bereits mehrmals im Artikel) die Bedeutung der «expérience vécue originale», der "original life experience":[710] "The insulin coma treatment, which provides an original life experience, chiefly in the anaclitic situation of

---

[705] Insulinkomata würden im Schockverlauf günstig den fundamentalen Wahn[zustand] oder das Syndrom des ‹‹automatisme mental›› verändern.
[706] JUILLET/DOREY, Insulinothérapie (1964), S. 14.
[707] Der Berliner Psychiater Prof. Tilo Held schrieb in einer e-mail am 4.3.2013 an Gernot Heiss, dass die 'Sakel-Kur' „in der Pariser Privatklinik, in der ich 1966/67 arbeitete, noch regelmäßig angewendet wurde."
[708] JUILLET, Traitements insuliniques et méthodes de choc dans la schizophrénie (1968), S. 108.
[709] Ebd., S. 117.
[710] Ebd., S. 120 (englisches Resümee).

waking[711], must be reserved for those cases in which the psychotherapeutic relation is impossible, either at an early stage (hebephrenic forms and simple schizophrenia resisting to disinhibiting neuroleptics), or after a more extended attempt (paranoid states in which the failure of drugtherapy is related to the fact that the patient is gratified by his delusion)."
Vergleichbar mit der Wiener Forderung, die Therapie in den individuell zu gestaltenden „Gesamtbehandlungsplan" einzuordnen, betonte Juillet, dass nun die Insulinkomatherapie nur mehr in Kombination mit (nachfolgender) Psychotherapie, mit Neuroleptika und / oder mit Elektrokrampftherapie angewandt würde; mit Hinweis auf das Handbuch von Léon Michaux zur Psychiatrie in der Ausgabe von 1965[712] schrieb er: ‹Es ist schließlich daran zu erinnern, dass nach Erfolglosigkeit der Insulinschocktherapie die Kombination von Fiebertherapie [allgemein, GH] und Elektroschocks manchmal gute Resultate bringt›.[713]

Die Kombination der Insulinkomatherapie auch mit Psychotherapie dürfte sich seit den 1940er Jahren durchgesetzt haben: Hans-Walter Schmuhl und Volker Roelcke weisen auf die Bedeutung hin, die – entgegen der öffentlichen Polemik – bereits in dieser Zeit von mehreren Psychiatern der Kombination der somatischen mit psychotherapeutischen Behandlungsmethoden gegeben wurde. Sie erwähnen dazu das Eindringen selbst der Psychoanalyse bereits um 1930 in die Psychiatrie an der Charité,[714] und allgemein den Umstand, dass etwa in der Schweiz und in den Niederlanden „die Psychotherapie als notwendige Ergänzung der Insulinkoma- und Cardiazolkrampftherapie" galt und dass man „in Frankreich […] die Psychiatrie zu dieser Zeit als ‚Psychobiologie' auf[fasste] und […] biologische, soziale und psychologische Ansätze [kombinierte]."[715] 1964 schließlich beschrieben Juillet / Dorey in ihrem Enzyklopädieartikel als Mehrheitsmeinung die

---

711 Vgl. den Abschnitt „Die Psychopathologie des Erwachens" in: MÜLLER, Die Insulinbehandlung (1963), S. 407-410.
712 Vgl. dazu das Zitat oben S. 78 von Henri Claude etc.
713 JUILLET, Traitements insuliniques et méthodes de choc dans la schizophrénie (1968), S. 115. Juillet verwendete 1968 in beiden Fällen den Begriff Schock.
714 Vgl. GABRIEL, Zur Beziehung zwischen Psychiatrie und Psychotherapie in Wien im 20. Jahrhundert (1999), S. 24 zu Wien, wo es trotz der heftigen Kontroversen in den 1920er Jahren „Doppelzugehörigkeiten von einzelnen Persönlichkeiten sowohl zum Stab der Psychiatrisch-Neurologischen Universitätsklinik unter Wagner-Jauregg als auch zu psychoanalytischen Institutionen" gab: z. B. Paul Schilder und auch Otto Pötzl.
715 SCHMUHL/ROELCKE, Einleitung (2013), S. 20, verweisen auf Beiträge im Sammelband: zu Berlin auf HERRN, Zum Umgang mit der Psychoanalyse (1913), S. 69-99; zu Frankreich auf COFFIN, French Biological Therapeutics (2013), S. 185-199; zu den Niederlanden auf VIJSELAAR, „A Hole in the Armour of Dementia Praecox". Somatic Cures within a Context of Psychiatry in Multiplicity: the Netherlands 1920-1950 (2013), S. 168-184.

Notwendigkeit der Kombination der Insulintherapie mit Psychotherapie und letzterer mit der Schaffung eines förderlichen sozialen Umfelds.[716]

1980 publizierte Juillet das Ergebnis einer Umfrage, in deren Rahmen Ende der 1970er Jahre Fragebögen zur Insulinkoma- und Insulinsubkomatherapie an 100 französische und 50 nicht französische Psychiater ausgesandt wurden.[717] Gefragt wurde zu den beiden Insulinkuren nach der Anwendung und ihrer Indikation bzw. nach der Ablehnung und ihren Gründen. Im Folgenden wird nur Juillets Auswertung der Umfrage zur Insulinkomatherapie zitiert. Auf die aufschlussreichen Ergebnisse der französischen Umfrage zur Therapie mit geringen Dosen Insulin, die nicht zum Koma führen, wird unten im Zusammenhang mit deren Anwendung in Wien vor allem bei PatientInnen ohne schizophrene Symptome hingewiesen.

106 der 150 Befragten (78 französische und 28 nicht-französische Psychiater) beantworteten die Umfrage zur Insulinkomatherapie. Zehn französische und ein nicht-französischer Psychiater wandten die Insulinkomatherapie noch an – mit einer recht uneinheitliche Palette von Indikationen: ‹schwere Anfangsformen, hebephren oder hebephren-kataton, gewisse Schizophrenien, die als besonders ernst eingeschätzt werden (hauptsächlich wenn Anorexie dominiert), insbesondere wenn sie nicht oder ungenügend auf eine korrekt gemachte neuroleptische Therapie oder auf die Elektrokrampftherapie reagieren.› Sechs dieser elf Antworten warnen vor einer zu raschen Anwendung, um Fehler in der Diagnose zu vermeiden, und bezweifeln den Sinn einer zweiten Anwendung bei Misserfolg. ‹Abweichend zu den klassischen Indikationen werden paranoiden Störungen nur einmal genannt. Außerhalb der Gruppe der Schizophrenien werden Indikationen nur ausnahmsweise erwähnt (in 2 von 11 Fällen): schwere Magersucht und gewisse depressive Zustände›. In der Zusammenfassung schrieb der Autor der Studie: ‹Die genannten Indikationen sind selten bis außergewöhnlich und wenig einheitlich. Sie gruppiere sich häufig um Formen von Autismus und Entwicklungen bei Jugendlichen, sowie um jene, vor allem autistischen Formen, die auf ein korrekt geführte neuroleptische Kur nicht besserten.› Insgesamt sprachen diese 11 Psychiater von einer sehr seltenen Indikation der Insulinkomatherapie.

---

[716] JUILLET/DOREY, Insulinothérapie (1964), S. 11f. In ihren Ausführungen zur Gruppentherapie zitieren sie auch Arnold. Zur ähnlichen Wiener Position über die Bedeutung des sozialen Umfelds in der Klinik vgl. unten S. 203.
[717] JUILLET, La cure de Sakel est-elle dépassée? (1980), passim. Juillet bezog sich auf eine Anregung von Henri Ey (1900-1977), der die völlige Aufgabe der Insulinkomatherapie bedauert habe ; vgl. EY, La Thérapeutique psychiatrique (Generalités) (1976), S. 15: « Il n'est pas sûr qu'on ait eu raison d'abandonner cette thérapeutique qui a pourtant fait ses preuves notamment dans les psychoses (dites aussi schizophrénies) délirantes aiguës. »

Die Mehrzahl der Psychiater, die auf die Umfrage antworteten, praktizierten die Insulinkomatherapie nicht mehr:

- 62 bezweifelten die Wirksamkeit, wozu Peter Berner (Wien) mit seiner Meinung namentlich genannt und zitiert wird.[718]
- 24 betonten die Risiken der Therapie, wovon der Psychiater Engelmeier (Essen) ‹sehr richtig bemerkt, dass die 'Sicherheitsanforderung' stark angewachsen sind›. Von einem Psychiater wurde hervorgehoben, ‹kein Kranker, keine Familie würde das [die Therapie] akzeptieren: ‚man habe schon so viele Schwierigkeiten mit einigen Elektroschocks!'› Die beiden Zitate verweisen auf die Veränderungen durch die Psychiatrie-Kritik der 1970er Jahre.
- 30 nannten Probleme der Organisation und des Personals, so Lothar B. Kalinowski,[719] der bedauerte, dass er seit zehn Jahren kein Spital im Raum New York finde, das sich für diese Behandlung eigne.
- Unter den nicht-Anwendern der Insulinkomatherapie waren 21 Psychiater, die sie nie angewandt hatten. Auf die Frage nach der Zukunft der Therapie waren in der Gruppe der nicht-Anwender 37 für ihr definitives Ende, 33 jedoch wollten sie nicht aus der therapeutischen Praxis ausschließen.

Den Recherchen zufolge, die Albrecht Hirschmüller für seinen Artikel von 2001 zur Geschichte der Insulinkomatherapie durchführte, kam man im Westen mit Beginn der 1960er Jahre mehrheitlich von der Insulinkomatherapie ab, während in der DDR, in Japan und ganz besonders in Russland ihre Anwendung noch bis in die 1980er Jahre häufig war.[720]

2.2.2 Die Position der Wiener Kliniker in ihren Publikationen und ihre Praxis

Ottokar H. Arnold[721] publizierte viel und vor allem zur Schizophrenie. Er war neben Hans Hoff der maßgebende Befürworter der Insulinkomatherapie an der Wiener Klinik. 1960 schrieb er: „Auch heute noch stellt die richtig indizierte und geführte Insulinvollschockkur die Basisbehandlung der Schizophrenie dar".[722] Gegen die statistischen Untersuchungen, die keinen messbaren Erfolg der Insulinkur ergaben, beriefen sich Stefan Hift und Hans Hoff in einem Artikel von 1958 auf die jahrelange Erfahrung, auf ihre Untersuchungen mit der Methode des „Simultanvergleichs"[723] und darauf, dass eine Normierung von psychiatrischen

---

[718] Eine ähnliche Bemerkung findet sich auch in BERNER, Hans Hoff (1998), S. 60 (s. Zitat unten S. 238).
[719] Er war aus NS-Deutschland vertrieben worden und einer der wichtigsten Befürworter der EKT.
[720] HIRSCHMÜLLER, Die Insulinkomabehandlung der Schizophrenie (2001), S. 224f. Zur DDR vgl. RZESNITZEK, „Schocktherapien" und Leukotomie in der DDR-Psychiatrie (2018), S. 292-296 (unten S. 238 Anm. 914).
[721] Lt. Personalstand der Universität Wien seit 1955 habilitiert, seit 1962 tit. a. o. Prof. und erster Oberarzt.
[722] ARNOLD, Die körperlichen Behandlungsmethoden der Schizophrenie (1960), S. 262.
[723] HIFT/HOFF, Die organische Therapie der Psychose (1958), S. 1047: „Wir wenden an unserer Klinik seit Jahren die Methode des Simultanvergleichs an. Alle therapeutischen Methoden werden in gleichem Maße in parallellaufenden Serien erprobt. Es wird dafür gesorgt, daß die Begleitumstände der Therapie in allen Fällen die gleichen sind. Dieselben Ärzte stellen die Diagnosen und beurteilen die Resultate." Es wurde dabei eine Gruppe

Störungen, wie sie bei einer statistischen Untersuchung vorgenommen werden muss, nur „eine Pseudoexaktheit“ einführen würde.[724]

2.2.2.1 Die Wirkweise der Insulinkomatherapie nach ihren Wiener Befürwortern

Wiederholt wurde in der Fachliteratur bedauert, dass eine allgemein anerkannte Erklärung für den Wirkmechanismus der Therapie fehle. Juillet / Dorey schrieben 1964 in einem Enzyklopädie-Artikel zur «Insulinothérapie», die Sakel-Kur bleibe eine im Wesentlichen empirische Methode; es gäbe zu ihr, wie zu den anderen ‚Schock‘-Therapien, keine zufriedenstellende Erklärung der Wirkweise. Dann führen sie Erklärungsversuche aus der Vorstellung der humoralen und vegetativen Wirkung des Schocks als Provokation einer „Alarmreaktion“ an, sowie Erklärungsversuche aus biologischen sowie aus psychologischen und psychopathologischen Konzepten.[725] Diese Ungewissheit hielt aber die Psychiater aufgrund positiver klinischer Erfahrungen nicht von der Anwendung der Therapie ab.[726]

An der Wiener Klinik hatte man jedoch eine Theorie zur Wirkweise der Komatherapie: „Die Wirkung der Insulinkur“, so schreiben etwa Hift und Hoff in dem bereits erwähnten Artikel von 1958, „beruht nach unserer Meinung auf der Änderung des Zuckerstoffwechsels der Gehirnzellen. Diejenigen Zellen des Gehirns, deren Zuckerstoffwechsel bereits gestört ist, sind gegen die Wirkung des Insulins weniger widerstandsfähig als die gesunden Zellen“. Sie „werden […] abgetötet oder fallen wenigstens funktionell aus. Dieser Vorgang wurde von Pötzl ‚Mauserung des Gehirns‘ genannt.[727] Wir glauben also nicht, daß die Insulinkur eine unspezifische Beruhigungstherapie ist, sondern halten sie für eine spezifische Therapie der

---

der PatientInnen, die mit einer Therapie behandelt wurde, mit einer unbehandelten Gruppe verglichen; „stets wurden die Fälle ohne Auswahl nach der Reihe der Aufnahme der einen und der anderen Reihe zugeteilt.“ Vgl. Wagner-Jauregg 1931 zu dieser Methode oben S. 78. Lt. KAUDERS, Zur Klinik, Theorie und Geschichte der Malariabehandlung (1948), S. 50, hatte Wagner-Jauregg den Begriff Simultanvergleich geprägt.

[724] HIFT/HOFF, Die organische Therapie der Psychose (1958), S. 1047: „Wir glauben [aufgrund der Unvergleichbarkeit der multifaktoriell bestimmten einzelnen Krankheiten und Krankheitsverläufe], dass die rein statistische Ausarbeitung eines möglichst großen Materials in der Psychiatrie zu Fehlresultaten führen muss. Es sind von Anfang an so viele Fehlerquellen vorhanden, dass die mathematische Bearbeitung nur eine Pseudoexaktheit einführt, die über die vorhandenen Fehler hinwegtäuscht.“ So würden nicht nur die Kriterien, die zu einer bestimmten Diagnose führten von Klinik zu Klinik variieren, es würde auch nicht zwischen Stadien und Unterformen psychiatrischer Erkrankungen unterschieden sowie auf den Ausbildungsgrad des behandelnden Arztes oder die „Atmosphäre“ an der Klinik geachtet.

[725] JUILLET/DOREY, Insulinothérapie (1964), S. 4. Auch JUILLET, Traitements insuliniques et méthodes de choc dans la schizophrénie (1968), S. 107 und S. 110f.

[726] Zu den verschiedenen „Theorien der Heilwirkung“ vgl. MÜLLER, Insulinbehandlung (1952), S. 239-250.

[727] Auf Pötzls „Mauserungstheorie“ bezogen sich bereits ARNOLD, Zur Theorie der Insulinschocktherapie der Schizophrenie (1952), S. 976 und HIFT, Zur weiteren Entwicklung der Insulintherapie (1952), S. 976, sowie auch noch ARNOLD, Die Therapie der Schizophrenie (1963), S. 23f.

Schizophrenie. Die Anwendung bei anderen Erkrankungsformen erscheint uns nicht gerechtfertigt.“ [728]

Auf dieser Annahme einer somatischen Grundlage der Schizophrenie und ebenfalls mit Bezug auf Otto Pötzls Mauserungstheorie[729] argumentierte Arnold ausführlich die Methode und Wirkung zur sukzessiven Ausschaltung der „in Läsion begriffenen“ Zellen, die schon ohne Unterversorgung mit Glukose nicht richtig arbeiten und die „dem schizophrenen Prozeß […] zugrunde“ liegen würden.[730] Durch das täglich (außer Sonntag) und mindestens 50 Mal wiederholte Insulinkoma werde die Glukoseaufnahme und damit der Erhaltungsstoffwechsel der Nervenzellen blockiert. „Entsprechend der […] angenommenen 36-Stunden-Grenze der Reversibilität wird nun bei sich innerhalb von 24 Stunden folgenden Komata an einer gewissen Anzahl Nervenzellen [vor allem an den gestörten Zellen, GH] die Erholungsphase unterschritten und im Sinne eines Kumulierungseffektes deren Vernichtung eingeleitet.“ [731] Der Sinn der Insulinkomatherapie liege also darin, „Hirnzellen zu vernichten, also eine Hirnschädigung zu setzen“. Draus ergäbe sich für den / die Therapeuten/in das Problem, jene Zellen „zu treffen, deren Fehlfunktion den Störfaktor der Gemeinschaftsleistung beinhaltet, die übrigen aber zu schonen. Behandelt man zu wenig intensiv oder konsequent, so wird der Effekt praktisch bedeutungslos bleiben, behandelt man mit rücksichtsloser Energie, so gibt es keinen Fall, bei dem der Prozeß nicht beseitigt werden würde, aber viele Fälle mit erheblichen hirnorganischen Läsionen, wie etwa unsere Erfahrung mit dem unabsichtlichen oder absichtlichen protrahierten Koma zeigen können. […] Die richtige Balance ist aber nur dann zu halten, wenn neben erstklassiger Organisation und richtiger Indikation eine riesige persönliche Erfahrung angesammelt worden ist. Der Autor glaubt leider, dass diesen Gedankengängen nur ganz wenige Insulinbehandlungsstationen auf der Welt gerecht zu werden vermögen.“[732]

---

[728] HIFT/HOFF, Die organische Therapie der Psychose (1958), S. 1045. Vorsichtiger formulierte Walter Schulte in einem Beitrag 1959, dass „sich die große Insulinkur noch am ehesten der Wurzel schizophrenen Geschehens nähert und auch dann noch Umstimmungen hervorzurufen vermag, wenn mit anderen Methoden nichts mehr erreicht werden kann.“ SCHULTE, Fortschritte der Somatotherapie bei Psychosen (1959), S. 356, zitiert in RZESNITZEK, „Schocktherapien“ und Leukotomie in der DDR-Psychiatrie (2018), S. 299. Auch die beiden Wiener Kliniker GASTAGER/SCHINDLER, Rehabilitationstherapie bei Schizophrenen (1961), S. 373, gehen in ihrer Planung der Therapie nicht mehr von einer „Methode der ‚Heilung'“ aus: „In Anbetracht der Tatsache, daß bis heute keine anerkannte Methode der ‚Heilung' der Schizophrenie existiert, wird angeregt, die Planung der Therapie bewußt auf den Gesichtspunkt der bestmöglichen Rehabilitation anzulegen.“

[729] Arnold bezieht sich auf PÖTZL, Die Wirkweise der Schockbehandlungen (1947), S. 11f.

[730] ARNOLD, Die körperlichen Behandlungsmethoden der Schizophrenie (1960), S. 267f. bzw. S. 263.

[731] ARNOLD, Die körperlichen Behandlungsmethoden der Schizophrenie (1960), S. 267f. bzw. S. 263.

[732] Ebd., S. 265.

Anders als Arnold, Hift, Hoff und Arnold hatte Schindler seine Thesen zur Schizophrenie nicht aus den somatischen Ursachen, sondern aus den psychischen Reaktionen des Betroffenen auf die „drohende […] Daseinszerreißung“ entwickelt: Was als schizophrene Defektzustände beschrieben wird, sind für Schindler das Ergebnis der „Umbauformen der Persönlichkeit“, eine „Persönlichkeitsabwandlung“, die der Patient in „Eigenleistung“ in der „akute[n] Abwehr“ des „Persönlichkeits-Zerfall[s]“ erreiche. Es sei möglichst früh mit einer Therapie einzusetzen, um die PatientInnen gegen die Gefahr des Persönlichkeitszerfalls zu stabilisieren. Auf der Basis des stabilisierten Zustandsbildes sei eine „Rehabilitationstherapie“ anzuschließen. In ihrer Studie von 1961 an Krankheits- und Rehabilitationsverläufen von 100 PatientInnen [733] empfahlen Gastager und Schindler zur Stabilisierung in der akuten Abwehrphase auch somatische Therapien (Elektrokrampf-, Insulinkoma-, Neuroleptika- und „bewußtseinsdämpfende“ sedierende Therapien), in der ‚Rehabilitierung‘ vor allem langfristige Gruppenpsychotherapie. Letztere erweise sich „als optimale Steuerungsmöglichkeit für den Ablauf der somatisch angeregten Persönlichkeitsabwandlung, gleichzeitig aber auch als gute Ausgangsbasis für die Erfordernisse der sozialen Rehabilitation in Beruf und Familie.“ Nach Zeitzeugen wurde die Gruppentherapie nach der Mitte der 1960er Jahre nicht mehr an der Klinik praktiziert.[734] Da die Gruppentherapie – wie auch das Anfang der 1950er Jahre von Schindler gemeinsam mit Arnold entwickelte therapeutische Verfahren der bifokalen Gruppentherapie[735] – bleibend in die Lehre eingegangen sind und vor allem in der ‚Rehabilitationsphase‘ vorgesehen waren, liegt die Vermutung nahe (wenn auch aus den Klinikakten nicht zu belegen), dass der Gruppentherapie eine zentrale Rolle im Rahmen des mit der Klinik verbundenen Rehabilitationszentrums für Schizophrene in Maria Lanzendorf zukam.

Der Wiener Kinder- und Jugendpsychiater Walter Spiel erwähnte 1967 kurz die theoretischen Vorstellungen zur Wirkungsweise der Insulinkomatherapie mit Verweis auf Pötzl und Arnold und führte weiters an, dass es auch eine „psychoanalytisch, psychogenetische Vorstellung über die Wirkung des Insulinschocks“ gäbe, „nämlich daß es dabei zu einer tiefen Regression

---

[733] GASTAGER/SCHINDLER, Rehabilitationstherapie bei Schizophrenen (1961), passim. Schindler und Gastager machten ihre Untersuchungen in den 1950er Jahren an der Klinik und verfassten die Studie auch noch an der Klinik, aus der sie 1960 bzw. 1962 ausschieden.
[734] Weder Bernd Küfferle (ab 1965 an der Männerstation) noch Eberhard Gabriel (ab 1966 an der Frauenstation) können sich an Gruppentherapien an der Klinik erinnern.
[735] Vgl. ARNOLD/SCHINDLER, Bifokale Gruppentherapie bei Schizophrenen, passim.

der Gesamtperson kommt, auf ein primitiv-menschliches Stadium und daß es das Erlebnis der absoluten Abhängigkeit vom Therapeuten, die Todesnähe, der psychische Streß sei, der die Reintegration fördere." Für die Stoffwechseltheorie von Arnold spreche, so Spiel, „daß, zumindestens im Kindesalter die schizophrenen Psychosen immer auch eine Veränderung der allgemeinen Hirnleistung zeigen; [...]. Diese Abbauzeichen sind leistungstestmäßig nachweisbar und sprechen für die ‚Organizität'. Aber auch die psychodynamischen Mechanismen sind bedeutungsvoll und beobachtbar. Man findet tatsächlich Zeichen von Regressionen und tiefsteckenden Abwehrmechanismen."[736] In Bezug auf die Anwendung der Insulinkomatherapie in der Kinder- und Jugendpsychiatrie schränkt Spiel 1967 diese auf Kinder ab 12 Jahren ein und zitiert auch Erfahrungen an der Wiener Klinik, die eine Unwirksamkeit bzw. negative Auswirkungen auf jüngere Kinder gezeigt hätten.[737] In seiner Monographie von 1961, in der er auf Ergebnisse seiner Studie mit 21 Insulinkomakuren verweist, wonach die Erfolge in der Nachbeobachtung weitgehend verloren gingen, schrieb er, dass auch „im allgemeinen […] der Optimismus bezüglich der Therapieerfolge in der Literatur nicht sehr überschwenglich [!]" sei.[738] Damit ließe sich begründen, warum in den überlieferten Akten der Kinderstation nach 1960 keine Insulinkomatherapie zu finden ist. Unter den sechs Kindern, die zwischen 1952 und 1960 mit einer Insulinkomatherapie behandelt wurden, waren laut Datenbank ein 9-jähriger, der 1952 behandelt wurde (XK66, Angstneurose); die anderen fünf Kinder waren 12- bis 15-jährig und hatten eine schizophrene Symptomatik.[739]

[736] SPIEL, Die Therapie in der Kinder- und Jugendpsychiatrie (1967), S. 135.
[737] Ebd., 137.
[738] SPIEL, Die endogenen Psychosen des Kindes- und Jugendalters (1961), S. 109. ASPERGER, Heilpädagogik (^{3}1961), S. 235 ist in der Behandlung der kindlichen Schizophrenie sowohl mit den „moderne[n] Schocktherapien" als auch mit den „‘Psychopharmaka'" skeptisch: „Bei beiden Behandlungsmethoden haben wir nicht den Eindruck, als ob sich das Auftreten neuer Schübe und dauernde Persönlichkeitsveränderungen wirklich verhindern ließen (bei Kindern wenigstens), so sehr es dadurch in manchen Fällen sicherlich möglich ist, den Kranken aus einem im Lauf befindlichen Schub herauszureißen."
[739] 1952 der 15jährige (S/IK64) mit dem Eintrag „hebephrenes Bild, Schwachsinn"; 1954 der 15jährige (SK319) mit „Hebephrenie"; 1957 die 14jährige (SK716) mit „Psychose"; 1957 der 13jährige (SK754) mit „Hebephrenie"; 1960 die 14jährige (SK1280) mit Schizophreniediagnose im Akt. Die PatientInnen von 1957 und 1960 bekamen auch EKT und / oder Cardiazolkrampftherapie im Koma ‚aufgesetzt'. GEIGER, Kinderstation (2015), S. 267, bringt Fallbeispiele.

2.2.2.2 Die Durchführung der Insulinkomatherapie an der Wiener Klinik und die (allgemeine) Einschätzung der Risiken

In der Durchführung der Insulinkomatherapie an der Wiener Klinik, die sich nach der oben zitierten Wiener Theorie zu ihrer Wirkweise richtete, hatte sich in den 1950er Jahren ein Standard entwickelt, der in den Publikationen beschrieben[740] und auch aus den PatientInnenakten ersichtlich wird: die Insulinkomabehandlung wurde sechs Mal die Woche jeweils vormittags durchgeführt, der Sonntag galt als Ruhetag. Die PatientInnen mussten laut internationalem Standard für mindestens eine halbe Stunde ins Koma versetzt werden; die Wiener Klinik bevorzugte eine Dauer von einer Stunde.[741] Einleitend wurde die Dosis schrittweise erhöht. Dabei begann man an der Wiener Klinik bei 20-30 Insulin-Einheiten und steigerte die Dosis täglich um weitere 10-30 Einheiten bis zum 1. Koma, das bei sehr unterschiedlich hoher Dosis eintrat;[742] mittlere Dosierungen lagen zwischen 80 und 250 Einheiten. Die Komadauer wurde von anfangs 10 bis 15 Minuten nach dem 10. Koma langsam auf eine Stunde gesteigert; manchmal wurde die Dosis im Verlauf der Therapie halbiert und wieder gesteigert (Zick-Zackverfahren nach Braunmühl[743]), bis das Koma bei weniger Einheiten wieder eintrat.[744]

Mit der Einführung der „kombinierte[n] Behandlung mit Insulin und dem Ganglienblocker Hexamethonium“[745] (als Standard ab 1956) konnte „im allgemeinen […] bei Frauen mit 80 und bei Männern mit 100-120 Einheiten „Altinsulin“[746] in etwa fünf Behandlungstagen mit Sicherheit das erste Koma“ erreicht werden.[747] Durchgeführt wurde die Therapie auf der dafür eingerichteten Insulinstation und von Ärzten, die eine Anästhesie-Ausbildung absolviert hatten und demnach in der Intubation und der Behandlung bei eventuellen Notfällen geübt waren. Auch das Pflegepersonal war speziell geschult. Die PatientInnen wurden früh am Morgen in die Insulinstation gebracht, und – da sie häufig im Koma mit starker Unruhe

---

740 Vgl. ARNOLD, Schockbehandlungen (1954), Teil II, S. 67-71; HOFF, Lehrbuch der Psychiatrie (1956), S. 505-507.

741 So ebd., S. 505 und auch noch ARNOLD, Die körperlichen Behandlungsmethoden der Schizophrenie (1960), S. 268.

742 Zur Dosierung ausführlich JOST, Zur Insulinempfindlichkeit der Schizophrenen (1958), S. 657-661.

743 Vgl. HOFF, Lehrbuch der Psychiatrie (1956), S. 481 und S. 510.

744 SPIEL, Die Therapie in der Kinder- und Jugendpsychiatrie (1967), S. 135f. Vgl. unten S. 194f. Anm. 755.

745 Zu dieser in Wien entwickelten Methode, um die Insulinresistenz und damit die Komplikationsrate zu senken, unten S. 195.

746 Veraltete Bezeichnung für Normalinsulin, d.h. für ein kurz wirkendes Insulin, das keinen Zusatz von wirkungsverlängernden Substanzen enthält.

747 ARNOLD, Die Therapie der Schizophrenie (1963), S. 32f.

reagierten – in Betten gelegt, die – in der Art von Kindergitterbetten – „mit etwa 70cm hohen Netzen umgeben" und nach oben offen waren.[748] Um das Koma abzubrechen, wurde den PatientInnen eine Zuckerlösung über eine Nasensonde eingeflößt; in den letzten Jahren des Untersuchungszeitraums wurde als schonendere Methode 1 mg Glucagon i. m. injiziert, wodurch die PatientInnen aus dem Koma so weit erwachten, dass sie selbst eine Zuckerlösung trinken konnten.[749] Die Anzahl der Schocks sollte nicht unter 40-50 Komata liegen, woraus sich bei dieser Therapie ein langer Aufenthalt der PatientInnen ergab.[750] Die Komatherapie wurde in der ‚Ära Hoff' standardmäßig nur einmal durchgeführt.[751]

Zur „Unterstützung" der Therapie wurden in Wien verschiedene Pharmaka gegeben. So erziele man, schrieb Hoff in seinem Lehrbuch 1956, „durch Beigabe von Adenosintriphosphorsäure[752] und [nach den Krankenakten sehr häufig von] Vitamin-B-Komplex eine günstige Beeinflussung des Zuckerstoffwechsels, [und] durch Anwendung von Beruhigungsmitteln, wie Luminal oder Belladenal einen ruhigeren und regelmäßigeren Schockablauf".[753] Zur üblichen Ampulle B-Komplex wurde lt. PatientInnenakten manchmal auch 1A Agrypnal (Phenobarbital) oder Pentavitol, (Calciumpantothenat) gegeben.

1960 verwies Arnold auf die Erfahrungen der letzten vier Jahre, in denen an der Wiener Klinik sowohl die Indikation und die Differenzierung in der Diagnose zur Steigerung der Effizienz als auch die Behandlungstechniken zur Ausschaltung der Risiken weiterentwickelt worden seien.[754] Zu letzterem, zur Verringerung der Risiken wurden vor allem nach

---

[748] Vgl. ARNOLD, Die körperlichen Behandlungsmethoden der Schizophrenie (1960), S. 269. Ausführlicher zur Organisation der Insulinstation siehe ARNOLD, Die Therapie der Schizophrenie (1963), S. 27-31: jede/r fünfte PatientIn sei „potentiell unruhig", „etwa jeder sechste oder siebente Patient [wird] ein oder mehrere Male während einer korrekt geführten Insulinbehandlung epileptische Manifestationen produzieren". Üblich blieben die PatientInnen von ca. 7 Uhr „bis spätestens 11 Uhr" in der Insulinabteilung.

[749] Vgl. GABRIEL/KÜFFERLE, Vergleich des Verlaufes der Insulinvollkoma-Kur bei Zucker- bzw. Glucagonweckung (1969), S. 338-343.

[750] ARNOLD, Schockbehandlungen (1954), Teil II, S. 67-71, hier S. 70: „Die Anzahl der Schocks soll nicht unter 40, bei uns unter 50, gelegen sein, Sakel selbst plädiert derzeit für Kuren mit bis zu mehreren Hundert Schocks. Nach eigenen Erfahrungen kann eine solche Behandlung (150 Schocks in unserem Fall) noch gelegentlich in prognostisch aussichtslosen Fällen wirksam sein." Lt. ARNOLD, Die körperlichen Behandlungsmethoden der Schizophrenie (1960), S. 268 „niemals unter 50 Vollschocks zu je einer Stunde, in manchen Fällen […] weit über 100." HOFF, Lehrbuch der Psychiatrie (1956), S. 504-520 (Vorlesung: Insulinbehandlung) erwähnt mehrmals, dass der ökonomische Aufwand für die Insulinkomatherapie durch die Länge des für die Therapie notwendigen Aufenthalts in Bezug auf die Erfolgsaussichten in Erwägung zu ziehen sei.

[751] In der Datenbank finden sich zwei Patienten, die 1950 eine Komatherapie zum zweiten Mal bekamen: vgl. unten zum Patienten S2536 S. **223f**. und zum Patienten S4636 S. 224.

[752] „Adenosintriphosphat ist der universelle und unmittelbar verfügbare Energieträger in Zellen und wichtiger Regulator energieliefernder Prozesse" (https://de.wikipedia.org/wiki/Adenosintriphosphat, 13.03.2023).

[753] HOFF, Lehrbuch der Psychiatrie (1956), S. 505ff.

[754] ARNOLD, Die körperlichen Behandlungsmethoden der Schizophrenie (1960), S. 263.

Möglichkeiten gesucht, um die Insulinempfindlichkeit des / der Patienten/in zu erhöhen und so eine Verringerung der zum Koma führenden Dosis zu ermöglichen.[755] An der Wiener Klinik wurde dazu in den 1950er Jahren eine Methode entwickelt,[756] deren Anwendung Stefan Hift 1961 beschrieb:[757] Demnach wurden an der Wiener Klinik seit Oktober 1956 Ganglienblocker gegeben, um „die initiale Insulinkomadosis zu verringern und damit die Einleitungsphase der Insulinkur [die langsame Steigerung der Insulindosis bis zum ersten Koma, GH] zu verringern." Verwendet wurde das im Handel befindliche Hexamethonium-Derivat *Depressin*, ein Präparat der Stickstoffwerke Linz. Hift beschrieb die Technik der Anwendung, die erwünschten und unerwünschten Wirkungen aus der Behandlung von 145 PatientInnen (123 Männern und 22 Frauen), bei denen durch diese Methode „die Insulindosis etwa auf ein Drittel gesenkt, die Dauer bis zum ersten Koma auf weniger als die Hälfte reduziert" worden sei. 1962, in dem oben zitierten Informationsaustausch zur Insulinkur in *The Lancet*, führte Stephen Kraus die ‚starke' Anwendung der Insulinkomatherapie an der Klinik seiner Geburtsstadt Wien – im Gegensatz zu vielen psychiatrischen Kliniken in Großbritannien, in denen die Insulintherapie abgeschafft worden war – auf die Etablierung dieser neuen Methode von Hift zur Senkung der Insulinresistenz und damit der Risiken der Therapie zurück. [758]

Als Komplikationen nannte Manfred Bleuler 1960: Die häufigsten seien „Lungenödeme und – wie bei der Schlafkur[759] – Pneumonie und verlängertes Koma."[760] Bei letzterem wache der Patient nicht mehr auf, obwohl die Hypoglykämie behoben ist, bekomme Fieber und sei „früher oft in wenigen Tagen" gestorben; beim „Überleben hinterließ das verlängerte Koma hin und wieder psychoorganische und neurologische Komplikationen (u.a. Aphasien)." Die Mortalität gab Bleuler mit „früher 1 – 2 %, heute darunter" an. Auch Arnold nannte 1960 das protrahierte Koma als gefährlichste Komplikation, zu dessen Lasten die Mortalität von 0,4 %" an der Wiener Klinik „ausschließlich" ginge.[761] Max Müller – er war ein wichtiger

[755] Vgl. dazu die beiden Publikationen aus der Innsbrucker Klinik: JOST, Zur Modifikation der Insulinschockkuren nach Sakel mit Insulin Novo Amorph (1956), S. 1016, und JOST, Zur Insulinempfindlichkeit der Schizophrenen (1958), passim. 1958 ging es um die Erhöhung der Insulinempfindlichkeit durch die Kombination mit Hyaluronidase, wovon angenommen wurde, dass sie die Permeabilität der Blut-Hirn-Schranke steigern würde.
[756] ARNOLD, Schockbehandlungen (1954), Teil II, S. 70.
[757] HIFT, Die Ganglienblockade bei der Insulinkur in der Psychiatrie (1961), S. 430-433.
[758] KRAUS, Insulin Therapy (Letters to the editor) (1962), S. 382.
[759] Im Unterschied zum Wiener Sprachgebrauch ist hier der Dauerschlaf mit künstlicher Ernährung gemeint.
[760] BLEULER, Lehrbuch der Psychiatrie ([10]1960), S. 154.
[761] ARNOLD, Die körperlichen Behandlungsmethoden der Schizophrenie (1960), S. 271: In 19 Fällen eines protrahierten Komas starben 3 Patienten: davon sei bei einem „ein vorhandener Herzfehler nicht ernst genug

Befürworter der Therapie –, schrieb 1952 zu „Mortalität und Dauerschäden“, es könne „wohl gesagt werden, daß die Mortalität der Insulinbehandlung bei richtiger Führung der Technik 1 % nicht übersteigt und bei großer Erfahrung und zweckmäßiger Organisation der Insulinstation unter 0,5 % gesenkt werden kann“.[762] Schäden „auf somatischem Gebiet“, die nicht nach einiger Zeit verschwinden würden – psychoorganische Störungen, Störungen des Kohlehydratstoffwechsels, Herzschädigungen –, seien (beim normalen Verlauf der Kur) nicht nachzuweisen.[763] Epileptische Anfälle, die nicht selten bei der Komakur vor bzw. nach dem Koma auftraten, wurden in ihrer Bedeutung für die Therapie unterschiedlich gesehen. Für Arnold hatte die Hypoglykämie die therapeutische Bedeutung und nicht die spontanen epileptischen Anfälle, wovon sich andere Psychiater (in Parallele zur Vorstellung über die Wirkung der Krampftherapien) einen wichtigen therapeutischen Effekt erwarteten.[764] Arnold zählte den epileptischen Anfall aufgrund der Hypoglykämie zu den Komplikationen.[765]

Kontraindiziert war die Insulinkomatherapie bei kranken und körperlich schwachen PatientInnen. In Wien wurden – aufgrund der Möglichkeit, mit Ganglienblockern die Insulinresistenz zu senken – auch DiabetikerInnen in Einzelfällen mit Insulinschocks behandelt, obwohl Diabetes auch als Kontraindikation angeführt war. Arnold und Heimo Gastager berichten im Jahr 1957 von einer Schizophreniepatientin, die unter Diabetes mellitus litt und nach der ersten Behandlung mit Elektrokrampftherapie kurze Zeit später wieder zur

---

beurteilt worden“, bei einem zweiten sei eine fehlerhafte Indikation gestellt worden; es sei eine akute bedrohliche Katatonie gewesen (die ja nach Arnold rasch mit E-Schocks hätte behandelt werden müssen). Übereinstimmend schrieben HIFT/HOFF, Die organische Therapie der Psychose (1958), S. 1047 in Bezug auf das Risiko der Insulintherapie von 3 Todesfällen aus ca. 250 Insulinkuren in den letzten 5 Jahren: zwei im protrahierten Koma, dass sich jetzt fast sicher vermeiden ließe, einer durch akutes kardiales Versagen. Vgl. auch unten S. 195.

762 MÜLLER, Die Insulinbehandlung (1963), S. 399 – schrieb von 0,4% bei 4835 Behandelten nach eigener Erfahrung an der Klinik in Münsingen (Bern); vgl. ebd. 399-403 zu den Zwischenfällen und ihrer Vermeidung. Im historischen Rückblick sprechen SCHOTT/TÖLLE, Geschichte der Psychiatrie (2006), S. 474 hingegen von 3 % Mortalitäts-Rate.

763 So MÜLLER, Insulinbehandlung (1952), S. 119-122. Anders SCHIPOKOWENSKY, Die Behandlung der Schizophrenie und der Zyklophrenie mit Heilkrampf, Insulinschock und Chlorpromazin (1960), S. 476; er anerkannte zwar die Erfolge der Therapie, schrieb jedoch: „Abgesehen von etwaigen körperlichen Schäden und in seltenen Fällen auch vom Todeseintritt, stellen die hypoglykämischen Schocks und die künstlichen epileptischen Anfälle für viele Patienten auch schwere Psychotraumen dar.“

764 So etwa HIFT, Zur weiteren Entwicklung der Insulintherapie (1952), S. 976: „Auch hier haben Pötzl und Sakel durch die Würdigung des epileptischen Anfalles in der Hypoglykämie Pionierarbeit geleistet.“

765 ARNOLD, Schockbehandlungen (1954), Teil II, S. 70: Frühanfälle, die noch vor Beginn des Prä-Komas einsetzen, würden keine Sofortmaßnahmen erfordern, die PatientInnen seien aber antiepileptisch weiterzubehandeln; im Gegensatz dazu seien Spätanfällen, welche 1 bis 2 Stunden nach dem Koma auftreten können, zu unterbrechen. Die Häufigkeit die je nach „Schockführung“ bei zwischen 20 und 40 % der PatientInnen liege, könne „mittels Hydantoinen, die sich zur routinemäßigen Prophylaxe einzubürgern beginnen,“ auf unter 10 % reduziert werden. Widersprüchlich dazu scheint zu sein, dass auch er die Kombination mit den im Koma „aufgesetzten“ Cardiazol- oder E-‚Schocks‘ empfahl: vgl. unten S. 205 die Abbildung aus Arnolds Buch von 1963, sowie S. 216.

Aufnahme kam. Da die Grenze der Wirksamkeit der Insulinbehandlung mit „1 bis 1 ½ Jahren“ nach dem primären schizophrenen Prozess allgemein anerkannt war, die Zeit also drängte, entschlossen sich Arnold und Gastager nun, eine Insulinkur an der Patientin durchzuführen, die laut ihrem Bericht nach 50 Komata zur vollen Remission führte. „Die weitere Behandlung erfolgte in einer psychotherapeutischen Gruppe und es trat kein Rückfall ein.“[766] 1960 schrieb dazu Arnold: „die Tatsache eines Diabetes mellitus“ stelle heute „keine Kontraindikation gegen die I-Behandlung dar“.[767]

2.2.2.3 Indikation und Erfolg lt. Wiener Publikationen

Im Artikel von Hift und Hoff von 1958, in dem die Autoren betonten, dass sie die Insulinkomatherapie nicht für „eine unspezifische Beruhigungstherapie“, sondern „für eine spezifische Therapie der Schizophrenie“ halten,[768] schrieben sie zur Indikation: Bei den Fällen „der beginnenden Schizophrenie […], welche mit Denkstörungen, Beachtungs- und Beziehungsideen beginnen“, scheine nach ihren Untersuchungen mit der Methode des „Simultanvergleichs“[769] „Insulin […] weit besser zu wirken [als Neuroleptika], besonders wenn man es mit Krampfbehandlungen kombiniert“. „Bei hebephrenen Formen mit Neigung zu Erregungszuständen und zum Autismus sind die Neuroleptika öfters dem Insulin vorzuziehen. Es wird hier eine gute Resozialisation auf niedrigem Niveau erreicht, wobei natürlich Psychotherapie und Arbeitstherapie unerläßlich sind. Die primär katatonen Formen werden nach wie vor am besten mit Elektroschock kupiert.“ Danach sei „nach dem weiteren Verlauf [zu] entscheiden, ob eine Insulinkur oder eine Kur mit Neuroleptika zweckmäßig ist.“ In der Behandlung älterer Schizophrenien, wie auch von affektiven Störungen wird in diesem Artikel keine Therapie mit Insulin erwähnt, sondern nur mehr Elektrokrampftherapie und Psychopharmaka, meist in Kombination und ergänzt durch Psychotherapie und Arbeitstherapie.[770]

---

766 ARNOLD/GASTAGER, Insulinschockbehandlung trotz Diabetes (1957), S. 261f.
767 ARNOLD, Die körperlichen Behandlungsmethoden der Schizophrenie (1960), S. 270.
768 Vgl. oben S. 189f.
769 Zur Methode des Simultanvergleichs, oben S. 188f. Anm. 722 und Anm. 723.
770 HIFT/HOFF, Die organische Therapie der Psychose (1958), S. 1047f. Vgl. ARNOLD, Die körperlichen Behandlungsmethoden der Schizophrenie (1960), S. 263 und S. 270, er argumentiert die Indikation fachbezogen noch ausführlicher. Vgl. zur „reiche[n] Vielfalt therapeutischer Ansätze“ mit den ‚heroische‘ somatischen Therapien und u. a. Psychotherapie und Arbeitstherapie in der Zwischenkriegszeit SCHMUHL/ROELCKE, Einleitung (2013), S. 19f. mit Verweisen auf Beiträge im Sammelband.

Vom Erfolg der Insulinkomatherapie waren die Wiener Kliniker bei diesen Indikationen überzeugt. In ihrem Artikel von 1954, der 1955 auch in französischer Übersetzung erschien, berichteten Hoff und Arnold über die Steigerung der Erfolgsquoten in den letzten Jahren: In der Anwendung von Sakel zwischen 1933 und 1940 seien in 60% der 273 Fälle Remissionen und davon in 30% der Fälle Rezidive beobachtet worden, 1950 bis 1952 in 158 Fällen – mit der Sakelschen Methode, aber mit einer veränderten Technik, u. a. indem Elektro- bzw. Cradiazolkrämpfe im Koma ‚aufgesetzt' wurden – 85% Remissionen und davon 15% Rezidive. Bei der allgemeinen Annahme von 30% Spontanremissionen waren „die von Sakel bearbeiteten Fälle, die ohne Rezidive blieben, kaum höher". Aber die Patienten mit Rezidiven lebten einen Großteil der 20 bis 30 Jahre nach der Therapie, die sie sonst in einer Irrenanstalt zugebracht hätten, „sozial angepasst". Hoff und Arnold unterstrichen neben der Kombination der Insulinkomatherapie mit einer der beiden Krampftherapien vor allem die frühe Anwendung als ausschlaggebend für den Erfolg der Insulinkomatherapie.[771]

Hans Hoff schrieb in seinem Lehrbuch von 1956 zu den Erfolgen ebenfalls von einer Verdoppelung der Remissionschancen bei insulinbehandelten PatientInnen,[772] um dann auszuführen, dass die durchschnittliche Dauer des Schubes bei insulinbehandelten PatientInnen abgekürzt sei, der Verlauf der Psychose jedoch nur unwesentlich beeinflusst zu sein scheine. So würde die Rezidivhäufigkeit nach den vielen dazu gemachten Studien gleichbleiben. Möglich sei, dass es zu weniger schweren Endzuständen käme. Prognostisch seien die hebephrenen Formen besonders ungünstig.[773] Man könne nie mehr als eine soziale Wiederanpassung erwarten und eine völlige Remission ohne Defekt käme praktisch nie vor.[774]

---

[771] HOFF/ARNOLD, Die Therapie der Schizophrenie (1954), S. 348 sowie (in Übersetzung) HOFF/ARNOLD, Au sujet de la thérapie de la schizophénie (1955), S. 12f.

[772] HOFF, Lehrbuch der Psychiatrie (1956), S. 517: etwa 50 bis 70% im Vergleich zu 20 bis 30%, die in Fachpublikationen recht allgemein bei frisch erkrankten Schizophrenen (innerhalb des ersten Jahres) als selbst remittierend angenommen wurden. Diese Einschätzung war in Wien allgemein, aber auch international verbreitet: ARNOLD, Die körperlichen Behandlungsmethoden der Schizophrenie (1960), S. 272f. So auch bereits HIFT, Zur weiteren Entwicklung der Insulintherapie (1952), S. 975, und wieder ARNOLD, Die Therapie der Schizophrenie (1963), S. 207. Ähnlich: EY/BERNARD/BRISSET, Manuel de Psychiatrie ([1]1960), S. 936f.

[773] Vgl. HIFT/HOFF, Die organische Therapie der Psychose (1958), S. 1045: „Bei hebephrenen Form mit Neigung zu Erregungszuständen und zum Autismus sind Neuroleptika öfters dem Insulin vorzuziehen."

[774] HOFF, Lehrbuch der Psychiatrie (1956), S. 517.

2.2.2.4 Integration der Insulinkomatherapie in den „Gesamtbehandlungsplan“ lt. Wiener Publikationen

In einem Artikel 1960[775] fasste Arnold die Ergebnisse seiner Publikationen der 1950er Jahre zum Thema zusammen und ergänzt sie durch die „in den letzten vier Jahren erzielten Fortschritte“ in der Diagnostik, Indikation und Technik der Anwendung. Er nannte die „Insulinvollschockkur die Basisbehandlung der Schizophrenie“ und betonte, dass auch sie nach der Beurteilung des individuellen Krankheitsfalls „in den Rahmen eines Gesamtbehandlungsplanes zu stellen“ sei, „in dem die körperlichen Methoden nur einen Teil bilden können, wenngleich dieser die Basis jeder anderen Therapie sein wird“. Hier und wieder ausführlich in seiner Monographie von 1963 betont er die Notwendigkeit eines „Gesamtbehandlungsplans“, in dem – je nach Art der Schizophrenie und je nach Prozessverlauf – die Insulinkomatherapie, konvulsive Therapien (Cardiazol- und Elektrokrampftherapie), psychochirurgische Methoden (Lobotomie), Fiebertherapie (Malariatherapie), die Behandlung mit Psychopharmaka und mit psychotherapeutischen Methoden (Einzel- und Gruppentherapien), „komplettierende Methoden“ (Arbeits- und Sporttherapie, Gymnastik-, Tanz- und Musiktherapie) sowie „*Soziotherapie*“ zu integrieren seien.[776] Das entsprach der damals an der ‚Klinik Hoff' vertretenen Ansicht von der „multifaktoriellen Genese“ der Geisteskrankheiten, aus der oft eine Abfolge oder Kombination mehrerer Therapien als notwendig abgeleitet wurde.

Die Kombination der Insulinkomatherapie mit einer Elektro- oder Cardiazolkrampftherapie wurde in Wien in der ‚Ära Hoff' durchgehend angewandt.[777] Hans Hoff geht im Lehrbuch der Psychiatrie 1956 ausführlich auf die Anwendungen der Insulinkomatherapie in Kombination mit Cardiazol- oder E-‚Schocks' bei Erkrankungen aus dem „Schizophrene[n] Formenkreis“ ein:[778] Nachdem er die Behandlung der „akute[n] bedrohliche[n] Katatonie“ und des „katatone[n] Stupors und katatone[r] Erregungszustände nicht bedrohlicher Art“ mit der Elektrokrampftherapie[779] beschreibt, kommt er zur „Therapie der Wahl“ der Hebephrenie

---

775 ARNOLD, Die körperlichen Behandlungsmethoden der Schizophrenie (1960), S. 262 – und gleichbleibend von ARNOLD/TSCHABITSCHER, Die Wiener Neurologisch-Psychiatrische Schule unter H. Hoff (1967), S. 1130.

776 Zum „Gesamtbehandlungsplan“, nachdem vorher die verschiedenen Therapien beschrieben wurden, ARNOLD, Die Therapie der Schizophrenie (1963), S. 133-198; die Tabelle zum Gesamtbehandlungsplan wurde auf S. 204f. übernommen.

777 HIFT, Zur weiteren Entwicklung der Insulintherapie (1952), S. 976.

778 HOFF, Lehrbuch der Psychiatrie (1956), S. 511 und S. 516f.

779 Ebd., S. 514-516. Bei letzterer fügt er hinzu: „Je nach dem weiteren Verlauf begnügt man sich mit der ES-Behandlung oder schließt eine Insulinkur an.“

und der paranoiden Schizophrenie: zur Insulinkur. „Ihr Indikationsbereich ist theoretisch sehr weit“, sei aber aus ökonomischen Gründen nur dort anzuwenden, „wo sie in einem größeren Prozentsatz erfolgversprechend ist“.

> Bei paranoider Schizophrenie beginne man mit einfacher Insulinbehandlung; falls es nach 15 bis 20 Komata zu keiner wesentlichen Besserung komme, sei sie mit „ES [‚Elektroschock‘] oder noch besser mit Cardiazol-Schocks“ zu kombinieren, „die zweimal wöchentlich, am besten jeweils an zwei aufeinanderfolgenden Tagen, kurz vor Abbrechen des Insulinkomas“ zu applizieren seien („4 bis 8 Krampfbehandlungen“ insgesamt).
>
> Insulintherapie bei alten Schizophrenien, d.h. mit einer Erkrankungsdauer über 1 ½, maximal 2 Jahren, würden nicht gemacht, „ausgenommen sind Schizophrenieformen mit deutlicher Remissionsneigung (z.B. ein völlig remittierter Schub vor 5 Jahren und jetzt der zweite Schub).“
>
> „Gelangt die Paranoia noch im ersten Stadium des Bedeutungs- und Beziehungswahns zur Behandlung […], muss unbedingt der Versuch einer Insulinkur gemacht werden. Die Erfolgsaussichten sind umso geringer, je systematisierter die Wahnideen und je intakter die Persönlichkeit sind.“

Hans Hoff ging in seinem Lehrbuch der Psychiatrie von 1956 auf die „Nachbehandlungen“ nach einer Insulintherapie bei Schizophrenie ein und beschrieb hier die Wichtigkeit psychotherapeutischer Maßnahmen:

> „Wir haben nicht nur eine Gruppe von Symptomen vor uns, die zum Abklingen gebracht werden müssen, sondern eine gestörte Gesamtpersönlichkeit, die ihre Anpassungsmöglichkeiten an die Umwelt zum Großteil verloren hat. Außerdem ist diese Umwelt selbst meistens bei der Entstehung der Psychose mitverantwortlich. Der Gesamtbehandlungsplan umfaßt also immer drei wichtige Punkte: 1. die Behandlung der Symptome der Krankheit. 2. Die Umgestaltung der Persönlichkeit und Erhöhung der Anpassungsfähigkeit. 3. Die Umgestaltung des Milieus.
>
> Der zweite Punkt wird meistens durch eine länger dauernde gelenkte Arbeitstherapie angegangen. […]
>
> Natürlich erfolgt auch in jedem Fall eine individuelle Psychotherapie, die je nach der besonderen Lage des Falles verschieden ist.
>
> Ich möchte wegen ihrer besonderen Bedeutung eine spezielle Art der Psychotherapie herausgreifen […] Wir haben gesehen, daß die Insulinbehandlung in frischen Fällen [der Schizophrenie] sehr große Erfolgsaussichten hat und daß über die Hälfte der Fälle vom Schub geheilt werden. Wir sahen aber auch, daß die somatische Therapie den schizophrenen Prozeß als solchen nur momentan unterbricht und ihn nicht für sehr lange Zeit aufhalten kann. Wir haben also hier eine bestimmte Zeitspanne zur Verfügung, um in andere Richtung aktiv einzugreifen.
>
> Erst jetzt, nachdem die Insulinkur uns eine Art Eingangspforte zur Psyche des Patienten eröffnet hat, ist ein richtiger Kontakt zum Psychotherapeuten möglich. Daher wird es verständlich, daß am Ende einer Insulinkur der günstigste Augenblick zum psychotherapeutischen Eingreifen ist.[780] […]

[780] Vgl. dazu Arnolds Ausführung 1956 in der Beurteilung und Therapieempfehlung für den 44jährigen Privatpatienten A3222 mit der Diagnose „Depression + Zwangsneurose“ während einer Insulinsubkomatherapie: „Ich schlage vor, die im Insulin sich bildende Lockerung zur Konfrontation mit der Ekphorierung verdrängter Inhalte zur Psychotherapie zu benützen.“

> Wir haben deshalb versucht, im Anschluß an die Insulinbehandlung bestimmte ausgewählte Kranke einer die analytische Methode benützenden Psychotherapie zu unterziehen. Daß diese Psychotherapie nicht eine Einzelbehandlung, sondern als Gruppentherapie erfolgt, hat nicht nur ökonomische Gründe. Schließlich symbolisiert eine Gruppe das Prinzip des Familienkreises und gibt daher dem psychotherapeutischen Leiter weitgehende Möglichkeit, die Wiedereingliederung des gewesenen Kranken in eine Gemeinschaftsform zu studieren und schließlich zu beeinflussen.
>
> […] [parallel dazu sei eine Gruppe zu bilden] aus den effektiven oder potentiellen Eltern des Kranken".[781]

Einer psychotherapeutischen Betreuung nach der Komatherapie wurde demnach – wie in der oben zitierten internationalen Literatur[782] – eine große Bedeutung beigemessen. Eberhard Gabriel beschrieb in einer Publikation zu den Verbindungen biologischer und psychotherapeutischer Sichtweisen in der Wiener Psychiatrie im 20. Jahrhundert im Zusammenhang mit „einer neuerlichen Annäherung von Psychiatrie und damals psychodynamisch dominierten Psychotherapieschulen" nach 1945 die Position von Hoff: Er habe „Wert auf die Integration psychodynamischer Gesichtspunkte in die Interpretation psychischer Krankheit und psychotherapeutischer Verfahren bei ihrer Behandlung" gelegt und „die Entwicklung der bifokalen Gruppenpsychotherapie Schizophrener durch O. H. Arnold und Raoul Schindler (1952)" sei „ein Beispiel für eine Methodenentwicklung zur Integration eines analytisch orientierten Verfahrens in der praktischen Psychiatrie."[783]Arnolds Beschreibung der „Organisation der Behandlung" (1960) zufolge wurde die Psychotherapie (Gruppentherapie) während der Insulinkomatherapie vorbereitet. „Der in Aussicht genommene Psychotherapeut […] vertritt dem Kranken gegenüber die Rolle dessen, der für baldige Entlassung eintritt, der für die kleinen persönlichen Wünsche und Sonderwünsche ein geneigtes Ohr hat, und versucht gegenüber dem Abteilungsleiter schrittweise Begünstigungen im Hinblick auf Unterbringung, Gartenbesuch, Angehörigenkontakt usw. durchzusetzen." [784]

Der Anspruch, einerseits an „Bewährtem" festzuhalten und dieses weiterzuentwickeln, andererseits die neuen Ansätze zu integrieren, zeigt sich auch 1963 in der Monographie zur Therapie der Schizophrenie von Ottokar H. Arnold, in der er sich für eine kombinierte Therapie der Schizophrenie, bestehend aus ‚Schock'-, medikamentöser- und Psychotherapie,

---

[781] HOFF, Lehrbuch der Psychiatrie (1956), S. 519f. (im Rahmen der Vorlesung „Schocktherapien"). Vgl. dazu ebenda, S. 494 im Kapitel zur Therapie der Schizophrenie zu den Erfolgen mit den nach einer Insulinkur von Schindler und Hift psychotherapeutisch betreuten Gruppen und zu Schindlers „bifokale[r] Schizophrenietherapie".
[782] Vgl. oben S. 186f.
[783] GABRIEL, Zur Beziehung zwischen Psychiatrie und Psychotherapie in Wien im 20. Jahrhundert (1999), S. 25.
[784] ARNOLD, Die körperlichen Behandlungsmethoden der Schizophrenie (1960), S. 270.

aussprach.[785] Hoff lehrte in seiner am Anfang der 1960er Jahre gehaltenen Vorlesung „Allgemeine Psychiatrie“ eine Kombination von Neuroleptika (Truxal/Chlorprothixen, Largactil) und Insulinkomatherapie bei schubhaft verlaufenden Schizophrenien, wobei er einschränkend betonte, dass eine Insulinkur nur dann angezeigt sei, wenn der Ausbruch der Krankheit nicht länger als 1 ½ Jahre zurücklag. Bei bereits länger bestehenden Schizophrenien favorisierte er hingegen den Einsatz des Neuroleptikums Majeptil (Thioproperazin)[786] und von Arbeitstherapie.[787] Bei Legierungspsychosen – der Mischdiagnose einer schizophrenen und affektiven Erkrankung – sollte der Arzt nach Hoff (um 1961) mit Neuroleptika und ‚E-Schockbehandlungen‘ beginnen und mit einer Insulinkur fortfahren, wenn nach Abklingen einer Melancholie ein schizophrenes Zustandsbild erkennbar sei. In Fällen, in denen Insulin wegen des langen Zurückliegens des Krankheitsausbruchs kontraindiziert sei und in denen auch alle anderen konventionellen Methoden versagt hatten, war eine Lobotomie seiner Meinung nach in Erwägung zu ziehen. Nach Hoff kamen für diesen chirurgischen Eingriff PatientInnen in Frage, die bereits verschiedentlich behandelt worden waren und die fortdauernd an inneren Spannungen litten und bei denen die Entlassung in ein gut vorbereitetes familiäres Milieu garantiert war.[788]

Die Neuroleptika wurden seit ihrer Entdeckung Anfang der 1950er Jahre von den Wiener Klinikern in die Behandlung schizophrener Erkrankungen einbezogen. Freilich waren Hoff und Hift, wie auch Arnold, jedoch der Meinung, dass die Insulinkomatherapie durch sie nicht zu ersetzen sei, da die Neuroleptika bei schizophrenen Erkrankungen nur symptomatisch wirken, es sich also um ein „Anpassungsphänomen und nicht um ein Heilungsphänomen“ handeln würde und im Gegensatz dazu die Insulinkomatherapie „in einem Teil der Fälle“

---

785 Vgl. zusammenfassend dazu GRÖGER, Insulin-Schocktherapie (2005), S. 219.
786 Zum Majeptil vgl. unten S. 285-287. Fallbeispiele zu Patientinnen mit Majeptiltherapie vgl. zum Patienten S8744 die folgende Anm. 786, zum Patienten S4903 S. 214 Anm. 829, zum Patienten S655 S. 84 und zum Patienten S7509 S. 287.
787 Dem entsprach die Behandlung des 1959 bei seinem ersten 88tägigen Aufenthalt 17jährige Mittelschüler S8744 mit der Diagnose „Schizophrenie“. Er bekam als Medikament nur an den ersten 8 Tagen am Abend N714 (Studienname nicht aufzulösen; 1 Amp. i. m.) und eine Insulinkur mit 50 Komata (danach noch 9mal Arbeitstherapie bis zur Entlassung) und war nach seiner Entlassung in der 7. Klasse Vorzugsschüler. 1961 kam er zum zweiten Mal zur Aufnahme und wurde an diesen 66 Tagen medikamentös behandelt; er bekam regelmäßig das Medikament „7843“ (3-4x 1Tbl. à 10mg; Studienname für Majeptil oder verwandtes Medikament, s. oben S. 89) oder „Majeptil“ (3-4x 1Tbl. à 10mg); auf der Fieberkurve ist auch häufig Arbeitstherapie eingetragen. Bei der 3., 28tägigen Aufnahme 1962 als 20jähriger verweigerten die Eltern die Zustimmung zur Majeptilkur; der Patient bekam eine EKT mit 6 Anwendungen. 1968 kam er zu seiner 4. und letzten Aufnahme mit der Diagnose „Medikamentenüberdosierung, chron. Medikamentenmissbrauch, Schizophrenie?“; am 2. Tagen wurde er „i.A. der Visite ad ‚M‘“, d.h. ins „Psych. Krk. Haus der Stadt Wien“ (Steinhof) gebracht.
788 Hoff-Skriptum, Allgemeine Psychiatrie [um 1961], S. 69f.

„eine spezifische Therapie der Schizophrenie“ sei.[789] Der Stellenwert der Neuroleptika im ‚Gesamtbehandlungsplan‘ zeigt sich etwa 1961 in der Tabelle zu den Therapien bei schizophrenen Verläufen von Arnold und Hoff[790] und in der Tabelle, die Arnold 1963 in seinem Buch zur Schizophrenie brachte und die hier im Text auf den beiden nächsten Seiten abgebildet ist.

Im einem Artikel von Arnold und Hoff von 1961 verwiesen die beiden Autoren auch auf die Bedeutung, die sie der Atmosphäre an der Klinik für den Behandlungserfolg beimaßen: die Klinik stelle für den / die Patienten/in „das Tor zum Leben dar“; er / sie müsse „imstande sein, menschliche Kontakte zu erneuern, und damit das geschehen kann, müssen geschlossene Türen geöffnet werden und die Gitter von den Fenstern verschwinden.“[791] Eine Tabelle zeigt, dass von den jeweils 3 Stationen der Männer- und der Frauenabteilung, die 1950 alle gesperrt, mit vergitterten Fenstern und jeweils 2 davon mit (auch oben geschlossenen) Gitterbetten ausgestattet waren, 1960 nur mehr B 12 und A 14 gesperrt und mit gesicherten (nicht vergitterten) Fenstern und statt der „Zellen“ nun mit „Einzelzimmer[n]“ und mit Insulinbetten[792] ausgestattet waren; die beiden anderen waren „offen, Fenster frei“. Diese Veränderungen würden zeigen, „welche organisatorischen und baumäßigen Veränderungen uns diese Fortschritte im therapeutischen Handeln allmählich gestattet haben“.[793]

1963 publizierte Arnold, der sich seit den 1940er Jahren zentral mit diesem Thema beschäftigt hatte, seine Monographie zur „Therapie der Schizophrenie“ mit dem Untertitel „Studien und Ergebnisse einer planmäßigen Ganzheitsbehandlung“. Hier brachte er als seinen letzten Stand zum Thema eine zweiseitige Tabelle zu den Verlaufstypen und zu ihrer Behandlung, die wohl

---

[789] Vgl. oben S. 189f. und S. 197. die Zitate aus HIFT/HOFF, Die organische Therapie der Psychose (1958), S. 1047-1048. Anderer Meinung war in seinem in Wien publizierten Artikel Juan LÓPEZ-IBOR, Therapie der Schizophrenie (1956), S. 31, der für die Behandlung der Schizophrenie mit Insulin- wie auch mit anderen ‚Schocktherapien‘ anführte, dass man dadurch zwar „den Fortgang der Schizose […] nicht aufzuhalten“ vermöge, dass jedoch „die Behandlungsmethoden […] die phasischen Formen der Krankheit [beeinflussen], indem sie deren Dauer verkürzen und es [dadurch] der Persönlichkeit erlauben, aus der Krankheit unversehrter hervorzugehen als ohne Behandlung.“

[790] ARNOLD/HOFF, Fortschritte in der Behandlung der endogenen Psychosen (1961), S. 507.

[791] ARNOLD/HOFF, Fortschritte in der Behandlung der endogenen Psychosen (1961), S. 509.

[792] D.h. oben offene, in der Art der Kindergitterbetten.

[793] ARNOLD/HOFF, Fortschritte in der Behandlung der endogenen Psychosen (1961), S. 507f. Vgl. zu Letzterem auch Hans Hoff an das Nobelkomitee (von dem er um einen Vorschlag gebeten wurde), 11.10.[1952], Kopie im Josephinum Wien, NL-Hoff MUW-AS-006005-0015: „Mit Hilfe der Elektroschockbehandlung gelingt es, unruhige Patienten zu beruhigen und wenn heute die Pflege an einer psychiatrischen Klinik oder in einer psychiatrischen Heilanstalt vereinfacht ist, so ist dies Ugo Cerletti zu danken.“ 1961 trugen zur Beruhigung des Klimas freilich nicht mehr nur die Elektrokrampftherapie, sondern vor allem die Neuroleptika bei.

– trotz einiger Skeptiker unter den Klinikern[794] – als Hauptrichtlinie an der Wiener Klinik für die letzten Jahre der ‚Ära Hoff‘ gelten kann. So blieben für Arnold noch in einem kurzen Vortrag beim 3. Donau-Symposium für Psychiatrie 1968,[795] in dem es um die Anwendung der Neuroleptika ging, die Insulinkomatherapie, ihre eventuelle Kombination mit Cardiazol-‚Schocks‘, wie auch die Elektrokrampftherapie wichtige Bestandteile des Gesamtbehandlungsplans der Schizophrenie.

Die folgenden beiden Abbildungen sind die beiden Teile eines Gesamtbehandlungsplans der Schizophrenie aus der Publikation von 1963,[796] den Arnold in seinem Vortrag von 1968 bzw. in der Publikation im folgenden Jahr wiederholt. Arnold geht von spezifizierenden Diagnosen aus und in den therapeutischen Maßnahmen bildet eindeutig die Insulinkomatherapie die Basis der Behandlung.[797] Die Neuroleptika zielen auch in der Argumentation sehr deutlich auf die Behandlung der Symptome ab:

Gesamtbehandlungsplan Tl. 1

O. H. Arnold, Die Therapie der Schizophrenie (1963) 168

168 — II. Der Gesamtbehandlungsplan

Tabelle 23. Indikationsschemata

Verlaufstyp	Phase der biologischen Basistherapie	Übergangsphase	Rehabilitierungsphase
Reine Phasenverläufe Erstmanifestation:	Elektroschockkur mit 6—15 ES, eventuell kombiniert mit Neuroleptika zum Akutgebrauch.	14tägige Kontrollphase, ab Ende der ES- bzw. Schlafkur gerechnet, unter Arbeits-, Gymnastik- und Tanztherapie.	Ambulante Gruppentherapie, Patientenklub, Berufsberatung, evtl. Umschulung. Hauptgewicht liegt auf der Rezidivprophylaxe, bei drohendem Rezidiv Aufnahme in der Tag- und Nachtklinik. evtl. Übergabe der Therapie an den Facharzt.
Bei schon bekanntem Verlauf:	Neuroleptika-Schlafkur oder ebenfalls Elektroschockkur.	Eventuelle Weitergabe eines Neuroleptikums zum Dauergebrauch.	
Akute bedrohliche Katatonie:	Schockblockbehandlung, s. Schema.		
Phasenverläufe mit Anzeichen für späteren Übergang in Schübe bzw. Prozesse, evtl. mit Exazerbationen:	Forcierte Elektroschockkur mit 3 ES im Abstand von je 8—12 Stunden, insgesamt 8—15 ES, evtl. Kombination mit Neuroleptika zum Akutgebrauch, baldmöglichster Übergang zur Insulinschockkur, evtl. kombiniert mit Cardiazolschocks.	Siehe bei Schubverlauf.	Siehe bei Schubverlauf.
Reine Schubverläufe: / Schubverläufe mit späterem Übergang in Prozeß, evtl. mit Exazerbationen:	Primäre Insulinschockkur, evtl. kombiniert mit Cardiazolschocks, Unterstützung mittels Neuroleptika z. Akutgebrauch, Kontaktaufnahme durch den Leiter der Rehabilitierungsabteilung in der zweiten Hälfte der Kur, Beginn mit Gymnastik- bzw. Sporttherapie, evtl. Tanztherapie.	Arbeitstherapie, Primäre Gruppenbehandlung in der bifokalen Gruppe, (gleichzeitige Elterngruppen), Gymnastik-, Sport-, Tanz-, evtl. Musiktherapie, Übergabe aus d. stationären Behandlung in die Tag- und Nachtklinik. Eventuell Neuroleptika zum Dauergebrauch.	Tag- und Nachtklinik, Gruppentherapie, Familientherapie, evtl. Übergang zur Einzeltherapie, Patientenklub, Arbeitstherapeutische Kurse, Umschulungskurse, Geschützte Werkstatt, evtl. Familienpflege.

[794] Vgl. zur Skepsis von Peter Berner gegenüber den Erfolgen der Insulinkomatherapie S. 188.
[795] ARNOLD, Zum Stellenwert der Neuroleptikatherapie in den Behandlungsplänen der Schizophrenie (1969), S. 11-14.
[796] ARNOLD, Die Therapie der Schizophrenie (1963), S. 168f.
[797] Vgl. Arnold oben S. 188 und S. 198, sowie S. 235.

2. Teil Gesamtbehandlungsplan

O. H. Arnold, Die Therapie der Schizophrenie (1963) 169

Verlaufstyp	Phase der biologischen Basistherapie	Übergangsphase	Rehabilitierungsphase
Primäre blande Prozesse:	Elektroschock- bzw. Neuroleptika (zum Akutgebrauch) nur dort, wo akute Überbaureaktionen, sonst Arbeitstherapie, Sporttherapie, unterstützt durch Neuroleptika z. Dauergebrauch.	Neuroleptika zum Dauergebrauch, unter deren Schutz Versuch der Einzeltherapie, Benützung der Tag- und Nachtklinik, Übergang von der Arbeitstherapie zur Wiederaufnahme des eigenen Berufes.	Weitergabe eines Neuroleptikums zum Dauergebrauch, Patientenklub, Umschulungskurse, evtl. langdauernder Einbau in eine Funktion im Rahmen eines Anstaltsbetriebes.
Primäre Prozesse mit rascher Progredienz, Exazerbationen: Bei Therapiebeginn bis zum Ende des 2. Jahres:	Langdauernde kombinierte Insulin-Cardiazolschockkur, Unterstützung mit Neuroleptika zum Akutgebrauch, Einbau der Gymnastik- und später Sporttherapie in der 2. Kurhälfte.	Langdauernde (Dressur-) Arbeitstherapie, Sport-, Gymnastik-, evtl. Tanztherapie, Leiter der Rehabilitierungsabteilung nimmt erst spät Kontakt auf, Einbau in eine lfde. Gruppe, gleichzeitige Elterngruppe, Entscheidung, ob Übergabe an eine Anstalt zum langdauernden Training oder Entlassung über eine lange Periode in der Tag- und Nachtklinik.	Gruppentherapie, Elterngruppe, Arbeitstherapeutische Kurse, Geschützte Werkstatt, Familienpflege, Patientenklub.
Bei Therapiebeginn nach dem Ende des 2. Jahres:	Abfangen einer evtl. Exazerbation mittels Elektroschockkur, 4—6 Majeptilkuren, dazwischen Arbeitstherapie, Gymnastik- und Sporttherapie, Gruppentherapie evtl. Lobotomie.	Eventuell Neuroleptika zum Dauergebrauch.	
Legierungspsychosen Reine Phasenverläufe:	Siehe Schema bei den Phasenverläufen.		Wie bei den Phasenverläufen, jedoch Rezidivprophylaxe mittels wochen- bis monatelanger kontrollierter Gabe von Imipramin oder Amitriptyline.
Mit Anzeichen für späteren Übergang in Prozesse:	Siehe Schema bei den entsprechenden Typen der Phasenverläufe.		

4. Indikationsschemata, Behandlungsphasen, Behandlungsintegrationen 169

Die Insulintherapie hatte für Arnold bei bestimmten schizophrenen Erkrankungen 1963 und auch 1968 ihren hohen Stellenwert beibehalten. Die Neuroleptika, die in Zürich und an vielen Orten die Komatherapie verdrängt hatten, waren im Gesamtbehandlungsplan bei den genannten Verlaufstypen „zum Akutgebrauch“ und bei den „reinen Phasenverläufen“ evtl. „zum Dauergebrauch“ aufgenommen, um im „Längsschnittverlauf schizophrener Psychosen und insbesondere die Rate der Resozialisierten“ zu beeinflussen und da die Neuroleptika in ihrer unspezifischen (!) Wirkung „gezielte Hilfen“ darstellten.[798]

Eine dem Umstand der Publikation geschuldete apologetische Ausführung zu Hoffs „Gesamtbehandlungsplan“, fasst die Position der Klinik zusammen und enthält zugleich eine für die Klinik charakteristische einschränkende Position gegenüber den neuen Medikamenten, findet sich in der Festschrift für Hans Hoff zum 70. Geburtstag 1967: Hier hieß es zum „Schizophrenieproblem“: „Die biologische Basistherapie (Elektroschock, Insulinschock) wurde unter der Leitung Hoffs in Teamarbeit systematisch weiterentwickelt. Schon 1952

[798] ARNOLD, Zum Stellenwert der Neuroleptikatherapie in den Behandlungsplänen der Schizophrenie (1969), S. 11-14.

konnten die ersten Neuroleptika in diese Therapie eingebaut werden. Sehr bald – wohl als erste Klinik der Welt – kamen wir unter der Leitung Hoffs zur Überzeugung, daß diese neuen Medikamente keine Revolution der Psychiatrie bedeuten, sondern vielmehr wesentliche Hilfen nur dann bieten, wenn sie in eine Gesamtbehandlung der Psychosen integriert werden. In einer Zeit, in der die Welt die bisher bewährten Behandlungen zugunsten einer rein medikamentösen Neuroleptikatherapie aufgab, war die Wiener Klinik einen Schritt weitergegangen: Gesamtbehandlungspläne mit Integration biologischer Basisbehandlung und Verwendung der Insulin- und Elektroschocktherapien bzw. Neuroleptikaanwendungen je nach Quer- und Längsschnitt der Erkrankung wurden ergänzt durch die Phase der Übergangstherapie, in der auf der Basis systematischer Arbeitstherapie und psychotherapeutischer Gruppenarbeit die Grundlage für die Resozialisierung gelegt wurde.“[799]

2.2.3 Die Insulinsubkomatherapie in Publikationen

Insulin, so der Wiener Kliniker Stefan Hift 1952, sei „um 1930 ein ziemlich viel verwendetes Mittel in psychiatrischen Anstalten“ zur „Bekämpfung der Sitophobie“[800] und „zur Dämpfung schizophrener Erregungszustände“ gewesen.[801] „Es handelte sich dabei um eine rein symptomatische Behandlung, da ja nicht die Psychose beeinflußt wurde, sondern das Symptom der Nahrungsverweigerung, beziehungsweise der Erregung.“[802] Zur Anwendung der „sog. ‚Subkoma-Behandlung‘“ findet sich im Bleuler‘schen Handbuch von 1960 der Vermerk: „Aus besonderen Indikationen heraus werden auch besonders leichte Behandlungen angewendet, z. B. unterbricht man stuporöse Fälle im Zeitpunkt, in dem der Stupor bei leichter Hypoglykämie einer Erregung weicht“.[803] Es ist 1960 in diesem Lehrbuch zu dieser Therapie wenig zu finden, was auf ihre geringe Bedeutung im deutschen Sprachraum in dieser Zeit schließen lässt,[804] wohl aber in Publikationen angloamerikanischer Psychiater aus den

[799] ARNOLD/TSCHABITSCHER, Die Wiener Neurologisch-Psychiatrische Schule unter H. Hoff (1967), S. 1130. Zu konkreteren Aussagen, die aber in die gleiche Richtung gingen, vgl. unten S. 253 die Zitate aus der Publikation von HIFT/HOFF, Die organische Therapie der Psychose (1958).
[800] Extreme Angst vor Essen, wodurch es zu einer Nahrungsverweigerung kommt.
[801] Nicht erwähnt werden die sehr frühen Versuche der Wiener Klinikerin Edith Klemperer mit kleinen Dosen von Insulin bei Delirium tremens: Vgl. KLEMPERER, Versuch einer Behandlung des Delirium tremens mit Insulin (1926), S. 549-551; KLEMPERER, Die Wirkung des Insulin beim Delirium tremens (1930), S. 163-190.
[802] HIFT, Zur weiteren Entwicklung der Insulintherapie (1952), S. 975.
[803] BLEULER, Lehrbuch der Psychiatrie ([10]1960), S. 153f.
[804] SARGANT/SLATER, Die modernen psychiatrischen Behandlungsmethoden (1951), S. 35: „Die Behandlungsmethode hat auf dem europäischen Kontinent noch keine allgemeine Anerkennung gefunden“. Vgl. auch MÜLLER, Die Insulinbehandlung (1963), S. 410; ebd., S. 404: Die Sukomabehandlung habe „namentlich in

1940/50er Jahren,[805] österreichischer Kliniker aus den 1950er Jahren und in französischen Publikationen auch noch in den beiden Jahrzehnten danach.

Erich F. Packesch, Assistent an der Grazer psychiatrisch-neurologischen Universitätsklinik, beschrieb 1951 in dem vom Leiter dieser Klinik Wolfgang Holzer[806] herausgegebenen Sammelband „Therapie der Nerven- und Geisteskrankheiten" die Insulinsubkomatherapie als „modifizierte Insulintherapie" mit folgenden Indikationen: „Insulinmastkuren" seien beim „Auftreten nervöser Zusammenbrüche bei körperlicher Erschöpfung, die manchmal mit schizophrenen Reaktionen einhergehen können," gemacht worden; das „langsame Ansteigen zu größeren Dosierungen, jedoch unter Vermeidung eines Komas, hat auf ängstliche und neurotisch verstimmte Patienten gute Einflüsse." Besonders zu empfehlen sei sie „bei Angstneurosen, nervösen Erschöpfungszuständen und Psychogenien, falls nicht zu schwere hysterieforme Charakterveränderungen vorhanden sind." Gute Erfolge verspreche die Behandlung mit Insulin ohne Koma bei PatientInnen die seit Anfang der Krankheit stark abgenommen haben. In einer Dosierung bis zum Subkoma sah Pakesch (1951) ein besseres Sedativum als die Barbituratsäurepräparate. Pakesch nennt nicht nur die Anwendung bei „manisch-erregten Kranken", sondern auch bei Delirium tremens und „zur Bekämpfung der Abstinenzerscheinungen" bei Morphiumsucht.[807]

Das war vor der Einführung des ersten Neuroleptikums Chlorpromazin (1952), die Subkomatherapie wurde aber auch in den Jahren danach an der Wiener Klinik noch in einigen Fällen von affektiven Störungen angewandt. „Therapeutisch ungünstige Verläufe aus dem MDK (Mischzustände, Dysphorien, rasch wechselnde Zykloidien, chronische Hypomanien)", schrieb Arnold 1954, würden „doch einigermaßen auf protrahierte Insulin-Subschockbehandlungen [reagieren] (vorwiegend eigene Erfahrungen)."[808] Im Lehrbuch von Hoff hieß es 1956: [809] Chronische Hypomanien seien „mit den heutigen Methoden absolut

England und Amerika große Verbreitung gewonnen, in erster Linie für die Behandlung von Neurosen und anderen psychogenen Leiden […], z. T. aber auch in der Schizophrenietherapie (Polatin)."

[805] Vgl. SARGANT/SLATER, Die modernen psychiatrischen Behandlungsmethoden (1951), S. 35-44.

[806] Wolfgang Holzer habilitierte sich in Wien Ende 1946 und war als Nachfolger von Kauders in Graz von 194**6** bis 1954 Leiter der psychiatrisch-neurologischen Universitätsklinik. Vgl. zu seiner Karriere GABRIEL, Zum Wiederaufbau akademischer Lehrkörper in der Psychiatrie in Wien nach 1945 (2016), S. 55f. und WATZKA, Die „Fälle" Wolfgang Holzer und Hans Bertha sowie andere „Personalia" (2016), S. 123-126.

[807] Erich F. PAKESCH, Therapie der Geisteskrankheiten (1951), S. 221, S. 235 und S. 239f. Sakel ist Ende der 1920er Jahre in Berlin bei der Behandlung von Süchtigen mit Insulin zu seiner Insulinkomatherapie bei Schizophrenen gekommen.

[808] ARNOLD, Schockbehandlungen (1954), Teil II, S. 71.

[809] HOFF, Lehrbuch der Psychiatrie (1956), S. 514 (Vorlesung: Schocktherapien).

unbeeinflussbar. Bei sozial untragbaren Patienten bleibt eine langdauernde Internierung die einzige Möglichkeit. Die besten Chancen einer vorübergehenden Beruhigung bietet noch eine langprotrahierte Insulin-Subschockkur." Bei zykloiden Verlaufsformen mit raschen Schwankungen zur Gegenseite empfahl Hoff „die lange prolongierte Insulinschockbehandlung (eventuell auch Insulin-Subschockbehandlung) zur Erreichung einer Stabilisation".

1967 schließlich war für Walter Spiel die „Subschockbehandlung" von Kindern und Jugendlichen als vier- bis sechswöchige Kur nur mehr bei der Diagnose Cachexia nervosa aktuell.[810] Denn wenn man, schrieb er, „aus therapeutischen Gründen einen leicht benommenen, gehemmten, passiven, dösigen Zustand wünscht, sei er heutzutage besser kontrollierbar durch die Neuroleptika herbeizuführen."[811]

In Frankreich wurden „chocs humides" in den 1950er und 1960er Jahren häufiger und breiter angewandt als in Wien. Léon Michaux schrieb 1965, dass viele Autoren, im Bewusstsein der großen Risiken der Komatherapie, die Anwendung geringer Dosen von Insulin ‹bis zum starken Schweiß› vorgeschlagen würden. So bleibe der / die PatientIn in einem Stadium leichter Benommenheit, halb bei Bewusstsein und könne durch selbständige Einnahme von Glukose geweckt werden. Diese ungefährliche Therapie sei auch günstiger für eine individuelle Psychotherapie oder Gruppentherapie.[812]

Im Folgenden soll zu Insulinsubkomatherapie die bereits oben zur Komatherapie zitierte französische Umfrage von Ende der 1970er Jahre erwähnt werden.[813] Von den ausgesandten Fragebögen (100 an französische und 50 an nicht-französische Psychiater) wurden zur Subschocktherapie 76 bzw. 27 beantwortet. 16 Franzosen und 6 Nichtfranzosen – also deutlich mehr als bei der Komatherapie – wandten noch Ende der 1970er Jahre die

[810] Es würden „nur so viel Einheiten Insulin gegeben […], daß ein ganz leicht benommener, apathisch-schläfriger, müder hypoglykämischer Zustand eintritt, der mit starkem Hungergefühl und leichten vegetativen Erscheinungen einhergeht". Lt. Datenbank zur Kinderstation wurden dort noch 1964 mit der Diagnose „Cachexia nervosa" eine 12-jährige (XK1819) und 1965/66 eine 13-jährige (XK2049) mit einer Subkomatherapie behandelt.
[811] SPIEL, Die Therapie in der Kinder- und Jugendpsychiatrie (1967), S. 138.
[812] MICHAUX, Psychiatrie (1965), S. 1001.
[813] JUILLET, La place actuelle de l'insulinothérapie à doses modérées en psychiatrie (1980), S. 170-176; gleichbedeutend wurden verwendet: « petite insuline », « chocs humides », « mild hypoglycemia » « subshocks, subcomas ».

Insulinsubkomatherapie an. Folgende Indikationen wurden angegeben (in 6 Fällen auch 2 oder mehrere):

- ‹ Anorexien und Beeinträchtigungen des Allgemeinzustands, wie hoch auch immer ihr pathologisches Niveau ist (10 [Psychiater]) › – wie auch in den folgenden Beispielen werden die Diagnosen für die Anwendung noch weiter präzisiert.
- ‹psychotische Zustände (10 [Psychiater]), mit Konkretisierung auf schizophrene Zustände (6 [Psychiater]) ›
- ‹ manche neurotischen Zustände (8 [Psychiater]) ›
- ‹ manche depressive oder ängstlich-depressive Zustände (6 [Psychiater]) ›
- ‹ ängstliche Zustände oder Ängste, ohne weitere Konkretisierung (4 [Psychiater]) ›
- ‹ Drogenabhängigkeiten (3 [Psychiater]) ›

Die Zahl der jährlichen Anwendungen war gering. Als Gründe für die Aufgabe der Insulinsubkomatherapie wurde von jenen, die sie nicht (mehr) anwandten, angegeben, sie hätte keine oder eine zu geringe Wirkung oder sei durch andere Therapien zu ersetzen; wieder wurde die fehlende Organisation und das fehlende Personal erwähnt, sowie die unerwünschten Wirkungen: Die Subschocktherapie sei im Vergleich zu den neuen Medikamenten zu stark eingreifend, wenige gut handhabbaren, für die PatientInnen anstrengender und hätte ‚psychologische Nachteile'.› Von den 60 französischen und 21 nicht-französischen Beantwortern der Umfrage, die die Subschocktherapie nicht anwandten, lehnten 30 bzw. 6 ihre Anwendung jedoch nicht ab, u.a. mit folgenden Bemerkungen: ‹falls einige daran glauben, muss man sie beibehalten›; es wurde empfohlen, Studien gemeinsam mit Endokrinologen, denn es sei durchaus möglich, dass sich dabei gewisse Vorteile der Therapie und eine Präzisierung ihrer Anwendung erschließen würde. Es wurde auch bedauert, die Subkomatherapie aufgegeben zu haben. 16 französische und 6 nicht-französische Psychiater der Umfrage wollten die Subschockkur endgültig ausschließen. Zusammenfassend schrieb der Autor der Studie: In den 103 auswertbaren Antworten habe sich gezeigt, dass von ein 1/5 der Antwortenden die Subkomatherapie noch angewandt wird, aber mit sehr seltenen Indikationen, und für die Hälfte ihre endgültige Aufgabe nicht erwünscht ist; ca. ¼ der Antwortenden lehnten die Weiterverwendung der Subkomatherapie vor allem als wenig wirksam und durch andere Therapien ersetzbar ab, ca. ¼ der Befragten gaben dazu keine Antwort.

Juillet / Dorey nannten 1964 in ihrem Enzyklopädie-Artikel zur Insulintherapie in Bezug auf die Anwendung der Insulinsubkomatherapie ihre Anwendung ‹ alleine oder in Kombination in einer großen Zahl von psychotischen und psychoneurotischen Zuständen ›. Die Autoren zitierten Eduardo Balduzzis Überblickswerk zu den Schocktherapien von 1962 mit der

Aussage, dass von der Insulinsubkomatherapie nichts anderes als ‹eine beruhigende, kräftigende und entgiftende Wirkung› erwartet werden dürfe.[814] Als Indikationen wurden von Juillet / Dorey (1964) vor allem Angstzustände genannt.[815] Weiters, wenn der Allgemeinzustand durch Anorexie gefährdet sei oder, um den circulus vitiosus bei neurotischen Erregungszuständen und Zuspitzungen zu durchbrechen.[816] Mit Subkomatherapie behandelt würden auch psychotische Zustände vor allem bei ‚Schizophrenen' sowie ‹Kriegs-Psychoneurosen›[817]; die letztgenannte Indikation stoße aber auch auf Kritik. Wie bei der Komatherapie wird besonders auf die Kombinationen der Therapie hingewiesen, so auf die in Frankreich von Henri Claude, dem Leiter der psychiatrischen Universitätsklinik St. Anne in Paris, und Pierre Rubenovitch schon um 1940 eingeführte Kombination mit Psychotherapie als Einzeltherapie oder vor allem Gruppentherapie.[818] Die beiden Autoren beschließen diese Ausführungen mit dem Hinweis: <Auch hier ist es die Erfahrung des Klinikers, die es ihm ermöglicht, für jeden Kranken die am besten geeignete Therapie zu wählen, sowohl auf der psychotherapeutischen wie der biologischen Ebene.>[819]

2.2.4 Die Anwendung der Insulintherapie an der Wiener Psychiatrie lt. PatientInnenakten

Die Insulintherapie (Insulinkoma- und Insulinsubkomatherapie[820]) ist unter den in der Datenbank aufgenommenen Fällen nach der EKT die zweithäufigste der ‚großen' körperlichen Therapien („Schockkuren") aus der Zwischenkriegszeit. In 965 Fällen[821] der in der Datenbank zu den Erwachsenenstationen im Untersuchungszeitraum (1951 – 1969)

---

[814] BALDUZZI, Le terapie di shock (1962), S. 79.

[815] Zitiert wird hier der Beitrag bei der internationalen Tagung über die Insulinbehandlung 1958 in New York von BERNATH, Modification of anxiety subsequent to insulin–induced mild hypoglycemia (1959): ‹ das Zielsymptom dieser Therapie ist die Angst, dieser Behandlung kommt jedoch nur eine unterstützende Rolle zu › (aus dem Französischen übersetzt von GH).

[816] Sie zitieren BALDUZZI, Le terapie di shock (1962), S. 78-80.

[817] EY/BERNARD/BRISSET, Manuel de Psychiatrie (^{1}1960), S. 936 zu den „chocs humides" mit starkem Schweißausbruch aber ohne Koma: « Cette insulinothérapie modifiée a été préconisée par Sargant et Craske (1941) dans le traitement des névroses de guerre. »

[818] JUILLET/DOREY, Insulinothérapie (1964), S. 14f.

[819] Ebd., S. 15 (aus dem Französischen übersetzt von GH).

[820] Insulinsubkomatherapien wurden in der Datenbank nicht extra ausgewiesen, ihr Anteil an der Gesamtzahl in Prozenten dürfte im einstelligen Bereich liegen.

[821] Es wird von Fällen gesprochen, da jede Aufnahme eines Patienten / einer Patientin, die den Einschlusskriterien (eine der fünf Diagnosen, ein Aufenthalt von mehr als vier Tagen) entsprach in die Datenbank aufgenommen und gezählt wurde.

aufgenommenen 14.919 Fälle, bzw. der 6.915 Fälle mit einer oder mehreren dieser Kuren, erhielten PatientInnen eine Insulinkur.[822]

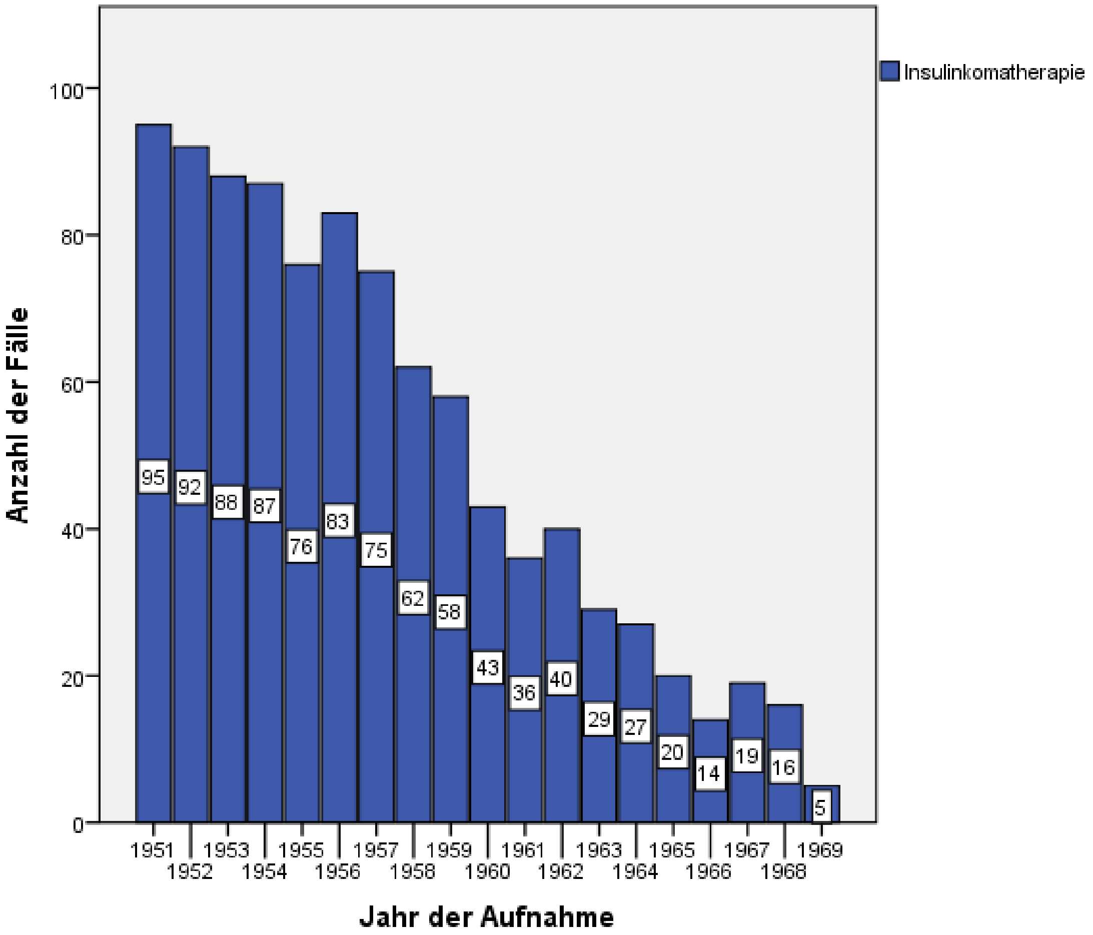

Abb. 8 Balkendiagramm, absolute Anzahl der **Insulinkoma- und Insulinsubkomatherapien** (1951-1969), Aufnahmen v. Pat. mit Einschlusskriterien (Diagnosen und Aufenthaltsdauer), n = 965 aus 14.919.

Die Häufigkeitsverteilung der Insulinkuren über die Jahre zeigt, dass die Insulintherapie über den Untersuchungszeitraum in absoluten Zahlen beinahe linear abnahm; um 1960 gab es einen spürbaren Einbruch. Ein ähnliches Bild eines kontinuierlichen Rückgangs wie bei den absoluten Zahlen zeigt sich in der Relation der Häufigkeit der Fälle mit einer Insulinbehandlung zu allen in der Datenbank nach den Einschlusskriterien aufgenommen Fällen bei einer Auswertung nach (Fünf-)Jahresblöcken: 1951-55 = 8,5%, 1956-60 = 6,9%, 1961-65 = 4,1%, 1966-69 = 2,4% der in der Datenbank in diesen Zeitabschnitten aufgenommenen Fälle bekamen demnach eine Insulinkur.

[822] Vgl. oben S. 34 Tab. 1 und S. 40 Tab. 2.

Im Folgenden geht es in diesem Abschnitt zur Praxis an der Wiener Klinik im ersten Teil um die Anwendung der Insulinkomatherapie bei PatientInnen mit der Diagnose einer schizophrenen Erkrankung. Nach den überlieferten und in die Datenbank aufgenommenen PatientInnenakten wurde die Insulinkomatherapie in Wien fast ausschließlich (ab 1962 ausschließlich) bei PatientInnen mit schizophrenen Erkrankungen angewandt.[823] Im zweiten Teil geht es um die Anwendung einer Insulintherapie bei PatientInnen mit anderen Diagnosen, vor allem mit einer affektiven Störung. 84 PatientInnen von 8.036 in der Datenbank aufgenommenen Fällen mit der Diagnose einer affektiven Störung[824] bekamen eine Insulinkoma- oder eine Insulinsubkomatherapie.[825] In diesen Fällen ist aufgrund von Hinweisen in mehreren der Akten zu zeigen, [826] dass die Komatherapie bei PatientInnen mit schizophrenen Symptomen gegeben wurde; ohne diese Symptome wurde in den 1950er und Anfang der 1960er Jahre bei affektiven Störungen manchmal eine „Insulinsubkomatherapie" ohne Koma angewandt.

Fallbeispiele sowohl zur Anwendung der Komatherapie, als auch zu jener der Subkomatherapie sollen vor allem dazu dienen, aufgrund von Bemerkungen, die sich manchmal zum Verlauf der Krankheit und / oder zum Erfolg der Therapie finden, auf Überlegungen des / der Therapeuten/in zur Indikation zu schließen – auf Absichten, die in diesen Quellen nicht deutlich artikuliert werden. Sie sollen auch die Kombination der Insulintherapie mit anderen „heroischen Therapien" im „Gesamtbehandlungsplan" verdeutlichen – in einigen Beispielen auch die Kombination mit medikamentösen Therapien und Psychotherapie.

---

[823] 1958 hielten HIFT/HOFF, Die organische Therapie der Psychose, S. 1045-1047 ihre Anwendung „bei anderen Erkrankungsformen" für „nicht gerechtfertigt": vgl. oben S. 190.; vgl. unten S. 225f. die Ausführungen zur Insulinkur bei affektiven Störungen.

[824] Zu beachten ist, dass die Legierungspsychose zur Schizophrenie und zu den affektiven Störungen gezählt wurde.

[825] Insgesamt wurden bei affektiven Störungen (vgl. oben S. 34 Tab. 1) 3.351 Schock- oder Fieberkuren angewandt: 3.155 Elektrokrampf-, 84 Insulin- (mit 5 Cardiazol-‚Schocks' im Koma) und 107 Malariafieberbehandlungen.

[826] Zur Frage, ob die Indikation nicht durchwegs den schizophrenen Symptomen gegolten hat, vgl. unten S. 225-227.

2.2.4.1 Insulintherapie bei PatientInnen mit der Diagnose einer schizophrenen Erkrankung

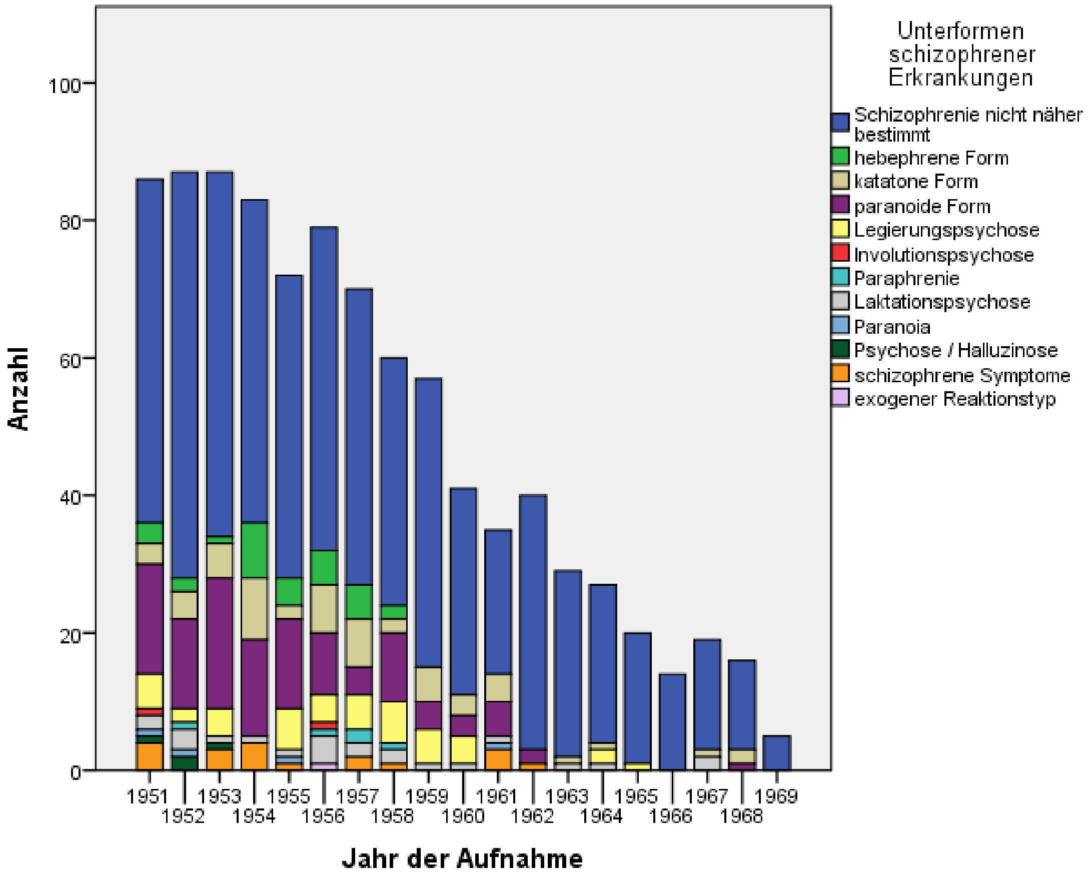

[redacted]

Abb. 9 Gestapeltes Balkendiagramm, Insulinkomatherapie + Unterformen der schizophrenen Erkrankungen (1951-1969), Pat. länger als 4 Tage stationär, n = 927, Mehrfachnennungen bei Diagnosen sind möglich.

Im gestapelten Balkendiagramm zu den schizophrenen Erkrankungen wird nicht nur die Abnahme der Anwendungen ersichtlich, sondern auch das Verschwinden der Diagnose Hebephrenie als Indikation für eine Insulinkur, die 1951 bis 1958 eine wichtige Indikation gewesen war (in grüner Farbe).[827] Nach der Statistik ging die Diagnose Hebephrenie völlig zurück – von 211 der in der Datenbank aufgenommenen Fälle 1951-1959 auf 14 Fälle 1960-

[827] HIFT/HOFF, Die organische Therapie der Psychose (1958), S. 1045: „Bei manchen Formen der Psychose, wie z. B. bei den hebephrenen Formen, ist meist das kurze Stadium der Therapierbarkeit mit Insulin bereits vorbei, wenn der Patient zu uns kommt.“ Vgl. oben S. 198.

1969.[828] Die wenigen Fälle in den 1960er Jahren wurden weder mit der Insulin- noch mit der Malariafiebertherapie behandelt.[829]

Zugleich sank aber auch der Anteil der Schizophrenen, die mit einer Insulinkomatherapie behandelt wurden, an der Gesamtzahl der in den Datenbank aufgenommenen schizophren Erkrankten deutlich (von 18% im Block 1951-1955 auf 16 % im Block 1956-1960, 10,4% im Block 1961-1965 und auf 7,0% im Block 1966-1969), was wahrscheinlich auf den zunehmenden Einsatz von Neuroleptika und die Einschränkung der Insulinkur auf bestimmte Verläufe von schizophrenen Erkrankungen, vielleicht aber auch auf eine zunehmende Skepsis einzelner Wiener Kliniker gegenüber der Insulinkomatherapie zurückzuführen ist.

In den Publikationen der Wiener Kliniker wurde häufig betont, dass die Insulinkomatherapie bei Schizophrenie nur bei einer möglichst frühen Behandlung nach dem ersten Ausbruch der Krankheit erfolgversprechend sei. Maximal ein bis eineinhalb Jahre danach werden etwa im Hoff-Skriptum von Anfang der 1960er Jahre genannt, sonst sei die Erfolgsaussicht gering.[830] Ebenso wurde hervorgehoben, dass die Insulinkur nur einen, wenn auch einen wichtigen Platz im Gesamtbehandlungsplan einnehme; erst ihre Kombination wurde für zielführend erachtet, häufig mit anderen somatischen Therapien und vor allem mit Psychotherapie und mit Maßnahmen zur Vorbereitung der sozialen Wiedereingliederung außerhalb der Anstalt.[831]

Auch die statistische Auswertung der Datenbank zeigt, dass die Insulinkomatherapie eine Indikation bei jungen Fällen von schizophrenen Erkrankungen war: lt. Boxplot Diagramm, das für den Endbericht des Projekts aus der SPSS Datenbank erstellt wurde, waren 75 % aller PatientInnen, die eine Insulintherapie erhielten, jünger als 30 Jahre.[832] Sie war auch eine

---

828 Vgl. ausführlicher dazu in HEISS, Projektendbericht (2015), S. 111f. die Tabelle zu den Krankheiten des schizophrenen Formenkreises (1951-1969) bei einem Aufenthalt an der Klinik länger als 4 Tage stationär.

829 Insgesamt wurden Koma-, Krampf- oder Fieberkuren in 156 von 225 der Fälle (69,3%) mit der Hebephrenie-Diagnose angewandt – in den 1960er Jahren noch bei 4 der 14 Fälle ‚E-Schocks'.

830 Vgl. das Zitat oben S. 200**.** Vgl. den Eintrag zur Therapieänderung im Krankenakt des Ende 1960 29jährigen Patienten S4903 mit der Diagnose „Schizophrenie": „nur Neuroleptica (Majeptil) und Arbeitstherapie"; „ursprüngliche Therapie (Insulinschockbeh[andlung]) wird geändert, da Psychose schon seit 1957 geht, Majeptil vorgesehen".

831 Die Meinung, die Komatherapie sei nur in Kombination mit anderen Therapien anzuwenden, wird auch von anderen PsychiaterInnen vertreten: vgl. JUILLET, Traitements insuliniques et méthodes de choc dans la schizophrénie (1968), S. 117. Nach der Lehre (vgl. oben die Zitate aus Publikationen von Hoff und Arnold) waren es in Wien vor allem Gruppentherapie und Arbeitstherapie, manchmal auch Sport- und Tanztherapie, welche die PatientInnen aktivieren und eine soziale Eingliederung vorbereiten sollten. Zur Psychotherapie an der Klinik bzw. im Rehabilitationszentrum für Schizophrene in Maria Lanzendorf vgl. oben S. 105.

832 Das Alter der PatientInnen, die eine Insulinkur bekamen, liegt bei einem Median von 25 Jahren deutlich unter dem Alter der in der Datenbank aufgenommenen PatientInnen von 35 Jahren. Vgl. auch BLEULER, Lehrbuch der

aufwendige Therapie mit langen Aufenthalten: 90% der PatientInnen, die eine Insulinkur erhalten haben, waren – so die Boxplot-Auswertung – über 68 Tage an der Klinik.[833]

Zur frühen Anwendung, die in den Publikationen häufig als einzig bzw. den besten Erfolg versprechende Anwendung genannt wurde, wurde im Projekt die Frage gestellt, ob eine höhere Wahrscheinlichkeit bestand, dass eine Insulinkomatherapie durchgeführt wurde, wenn PatientInnen erstmals mit der Diagnose Schizophrenie aufgenommen wurden. Die folgenden Aussagen beziehen sich auf die repräsentative Stichprobe, die Anfang 2014 im Rahmen der Pilotstudie aus den PatientInnenakten zu den Jahren 1955 bis 1960 gezogen wurde.[834] Eine Nicht-Erstaufnahme schloss demnach mit hoher Wahrscheinlichkeit eine Insulinkomatherapie aus. Lt. Stichprobe wurden auch mehrere ‚Schocktherapien' fast nur bei Erstaufnahmen vorgenommen[835] – was, wenn auch nicht in jedem Einzelfall, tendenziell auch für die Anwendungen in der ‚Ära Hoff' insgesamt zutreffend war; mit anderen Worten: Die Anwendung mehrerer der ‚großen alten Kuren' in einer Abfolge oder in Kombination ist üblicherweise bei Erstaufnahmen durchgeführt worden.

Die oben angeführten Richtlinien Hoffs und Arnolds zur Therapie schizophrener Erkrankungen zeigen sich in ihren Grundzügen auch in den PatientInnenakten. So der häufigen Kombination der Insulinkomatherapie mit einer Krampfbehandlung (E- oder Cardiazol-‚Schock').[836] Sie war nach Stefan Hift bereits 1952 „heute die Methode der Wahl bei den meisten schizophrenen Psychosen".[837] Mit der Ausnahme von drei Fällen 1951/1952/1957, in denen lt. Datenbank die Kombination „Cardiazol-Elektro-Schock" ohne Insulinkoma angewandt wurde,[838] und von zwei Fällen 1956, in denen die Patientinnen nach

Psychiatrie ([11]1969), S. 163: „Hohes Alter ist eine Gegenindikation für Insulin- und Schlafkur, weniger für Elektroschockkur."
[833] Die Aufenthaltsdauer der PatientInnen mit einer Insulinkur liegt bei einem Median von 99 Tagen weit über dem Median der in der Datenbank aufgenommenen PatientInnen von 25 Tagen.
[834] Die Stichprobe umfasste 525 PatientInnen (s. oben S. 21f.). Von diesen bekamen 265 PatientInnen eine oder mehrere Koma-, Krampf- und Fieberkuren (insgesamt 307 dieser Therapien); davon hatten 131 PatientInnen eine schizophrene Erkrankung, von denen 39 eine Insulinkur (2/3 davon PatientInnen der Privatstation) bekamen. Vgl. auch oben S. 43-47.
[835] In der Stichprobe aus den PatientInnenakten 1955-1960 ist die EKT mit Insulinkomatherapie die mit Abstand häufigste Abfolge bzw. Kombination: sie scheint in 28 Fällen auf, davon nur 1mal bei einer Nicht-Erstaufnahme.
[836] Vgl. oben S. 40 Tab. 2 sowie die Bemerkungen dazu.
[837] Vgl. oben S. 196 das Zitat aus HIFT, Zur weiteren Entwicklung der Insulintherapie (1952), S. 976.
[838] Vgl. dazu oben S. **90** zur Patientin S895. Vgl. den Patienten S656 mit der Diagnose Schizophrenie, der bei seiner 2. Aufnahme 1952 „6 Elektro-Cardiazol-Schocks" (nicht im Insulinkoma) zwischen einer Insulinkomatherapie und einer Lobotomie bekam und danach noch eine Malariatherapie (vgl. unten S. 222). Der 1957 20jährige Patient S3402 mit der Diagnose „Katatonie" erhielt an den ersten 11 Tagen seines 115tägigen Aufenthaltes EKT-Serien mit an 9 Tagen je 3, 2 oder einem „E-Schock mit Lysthenol", gegen Ende wurde er

einem Selbstmordversuch mit unbekannten Medikamenten Krampfdosen von Cardiazol bekamen, um sie aus dem Koma zurückzuholen,[839] sind in den ausgewerteten Akten des Untersuchungszeitraums die im Insulinkoma ‚aufgesetzten Cardiazolschocks' die einzige Form der Anwendung der Cardiazolkrampftherapie an der Wiener Klinik[840]; es ist die Anwendung, die William Sargant und Eliot Slater noch 1972 in der letzten Auflage ihres Lehrbuchs empfehlen, um die heftigen Angstzustände, die für die PatientInnen sehr unangenehmen Nebenerscheinungen der Cardiazolkrampftherapie, zu vermeiden.[841] Von den in der Datenbank nach den Aufnahmekriterien aufgenommenen 14.919 Fällen bekamen PatientInnen in 114 Fällen einen oder mehrere Cardiazol-‚Schocks'. Arnold schrieb 1960, die Kombination des Insulinkomas mit EKT trete immer mehr zu Gunsten des Cardiazolschocks zurück, der etwa bei jedem „primäre[n] Prozeß […] von vornherein ab dem 15. Insulinkoma […] unmittelbar am Ende der Komastunde […] dem Insulinkoma aufgesetzt" würde.[842]

Die im Lehrbuch von Sargant und Slater zitierte „symptomatische ES-Behandlung" bei „alte[n] Schizophrenien" ist in den PatientInnenakten schizophren Erkrankter häufig zu finden. So wurden einzelne oder mehrere Anwendungen („Schocks") nicht nur bei späteren Aufnahmen gegeben, sondern häufig in den Tagen nach der Erstaufnahme (manchmal kombiniert mit Sedativa oder Neuroleptika) und vor der langen Insulinkomatherapie, manchmal auch nach der Insulintherapie bei neuerlichem Auftreten von starker Erregung. Die Funktion der Beruhigung, Milderung oder Lösung von Angst- und Spannungszuständen wurde zunehmend häufig durch Neuroleptika übernommen – abgesehen im Fall der

---

5mal „combiniert Cardiazol-E-geschockt"; die Cardiazoldosis wurde mit 7-10ccm angegeben. Schließlich bekam er auch noch eine Insulintherapie mit 50 Komata. HOFF, Lehrbuch der Psychiatrie (1956), S. 515 erwähnt die Anwendung eines „*kombinierten Cradiazol-ES*" (ohne Insulinkoma) bei der akuten bedrohlichen Katatonie, um „einen [Krampf] Anfall auszulösen und dadurch das Leben des Patienten zu retten", der „in einem Erschöpfungsstadium […] nicht mehr auf den ES mit einem generalisierten Krampfanfall" reagiert. Diese Anwendung bei der akuten Katatonie, wenn der Patient auf die EKT nicht ausreichend (d. h. mit einem epileptischen Anfall) reagierte, nannte auch Arnold 1960: vgl. unten S. 252.

[839] Vgl. oben S. 39 Anm. 122.

[840] Hoff-Skriptum, Allgemeine Psychiatrie [um 1961], S. 69: „Cardiazolschock […] wegen des Vernichtungsgefühls nur im Zusammenhang mit einem Insulinschock".

[841] MCCRAE, 'A violent thunderstorm': Cardiazol treatment in British mental hospitals (2006), S. 84 zitiert SARGANT/SLATER, An Introduction to Physical Methods of Treatment in Psychiatry (1972), ohne Seitenangabe: "Cardiazol as an intervention for stuporous states, advising its injection during insulin sopor to avoid its notorious unpleasantness". Zum Verlauf der Cardiazolkrampftherapie: MCCRAE, 'A violent thunderstorm': Cardiazol treatment in British mental hospitals (2006), S. 71f. Zur Geschichte vgl. auch SHORTER, Geschichte der Psychiatrie (1999), S. 322-326.

[842] ARNOLD, Die körperlichen Behandlungsmethoden der Schizophrenie (1960), S. 268f.; nach Arnold wurde in Wien Cardiazol wegen „seiner hypoxämisch und hypämisch wirksamen Komponente" bevorzugt, „die über die des ES beträchtlich hinausreichen" könne. Auch HOFF, Lehrbuch der Psychiatrie (1956), S. 481, bevorzugte den dem Koma aufgesetzten ‚Cardiazolschock' gegenüber dem ‚E-Schock'.

Katatonie, bei der auch nach dem Lehrbuch die Elektrokrampftherapie die Therapie der Wahl war. Eine Malariatherapie wurde ebenfalls manchmal vor einer Insulinkur, manchmal bei einer späteren Aufnahme gegeben[843] und dürfte ebenfalls in dieser Funktion einer ‚symptomatischen Behandlung' gegeben worden sein. Nach den PatientInnenakten wurde kaum eine Insulinkomatherapie zweimal gegeben[844], in einzelnen Fällen aber bei einer ‚alten Schizophrenie' eine Insulinsubkomatherapie – in zwei in der Datenbank eingetragenen Fällen 1950 und 1948, also vor der ‚Ära Hoff', mit einem im Subkoma ‚aufgesetzten E-Schock'.[845]

> Der Fall des 18jährigen Patienten S7379,[846] der mit der Diagnose „Schizophrenie" 1965 (einzige Aufnahme, 126 Tage)[847] an der Klinik aufgenommen wurde, zeigt eine gängige Behandlung. Am Rückblatt der Fieberkurve war als Therapie eingetragen: „Th.: Insulin-Schock, 5h früh 2 Tbl. Mevasine, tgl. abds. 1 Tbl. [des Neuroleptikums] Melleril ret." Lt. Fieberkurve wurden um 12h und um 20h je 50mg. Melleril Drg. gegeben, die in den letzten 5 ½ Wochen durch 2x eine Tabl. Luvatren abgelöst wurde; er bekam in der 2. Woche je einen ‚E-Schock' an zwei aufeinanderfolgenden Tagen und am Tag darauf wurde eine Insulinkomatherapie begonnen mit insgesamt 70 Komata und 8 ‚Cardiazolschocks' (im Koma); aus dem Koma wurde er immer mit dem ab Mitte der 1960er Jahre in Wien üblichen Glucagon i.m. geweckt; in den letzten 3 ½ Wochen ist tgl. „A[rbeits]Th." eingetragen. Dann wurde er – wie bei SchizophreniepatientInnen in diesen Jahren üblich – zur Nachbehandlung in das Rehabilitations-Zentrum der Klinik in Maria Lanzendorf entlassen.
>
> Als Gegenbeispiel, d.h. ohne Komatherapie, kann im gleichen Jahr 1965 der 25jährige Student S7380 mit der Diagnose „Schizophrenie (Res[idual]zust[and]?)" angeführt werden. Bei seinem 66-tägigen einzigen stationären Aufenthalt an der Klinik bekam er nach zwei ‚E-Schocks' medikamentöse Therapien – Sedativa (Truxal und Valium) und schließlich Neuroleptika („P46" und Mellerill). Sein Fall dürfte ein Beispiel dafür sein, dass bei länger zurückliegendem ersten Auftreten der Krankheit keine Insulintherapie gegeben wurde. In den letzten sechs Wochen ist fast tgl. Arbeitstherapie eingetragen und im letzten Monat dazu auch meistens Gymnastik bis zur ‚Entlassung' ins Rehabilitationszentrum Maria Lanzendorf.

---

[843] Vgl. die Fallbeispiele auf Seite 219f.
[844] Zu 2 Ausnahmen vor 1950 vgl. unten zum Patienten S2536 S. 223f. und zum Patienten S4636 S. 224.
[845] 1950 zum Patienten S4636 vgl. unten S. 224. Der 1921 geborene Privatpatient S1579 mit der Diagnose „Schizophrenie": bekam 1948 bei seiner ersten Aufnahme eine EKT mit 13 Anwendungen und bei seiner zweiten Aufnahme im selben Jahr eine Insulinkur mit 14 Sub- und 5 Komata, wobei einmal im Subkoma ein ‚E-Schock' gegeben wurde. Er wurde 1952 lobotomiert. Bei seinem anschließenden 48tägigen Aufenthalt ist auf der Fieberkurve häufig Arbeitstherapie und einmal Psychotherapie eingetragen; es ist ein sehr seltener Eintrag, hier bei einem Privatpatienten hat das ev. mit der Verrechnung durch die Klinikverwaltung zu tun. Vgl. unten S. 223f. zum Patienten S2536.
[846] Derzeit beim Bundesheer 3 Jahre zeitverpflichtet (kein Hinweis auf Konflikte in der Anamnese), vorher nach Pflichtschule kaufmännischer Angestellter.
[847] Mit einem Schreiben eines niedergelassenen Psychiaters: „[…] Kopfschmerzen und plötzliche Gedankenflucht […]. Das EEG ist leicht diffus abnorm eher im Sinne eines durchgemachten cerebralen Leidens als eines Anfallsleidens."

Der genannte Fall des 18-jährigen Patienten S7379 (1965) zeigt die Kombination der Insulinkur mit E- oder Cardiazolkrampf an der Wiener Klinik, wie sie bereits in den beiden oben genannten Zitaten von Hift (1952) und von Hoff (1956) empfohlen und auch in den 1950er Jahren angewandt wurde; auch schlossen Arbeitstherapie an – vermutlich auch die in den Publikationen geforderte und nur selten in den Krankenakten erwähnte Psychotherapie (Gruppentherapie) nun im Rehabilitationszentrum der Klinik in Maria Lanzendorf.[848] Anders war es mit den Neuroleptika. Während in den beiden Fällen von 1965 Neuroleptika therapeutisch gegeben wurden, wurden bei PatientInnen, wenn sie bei schizophrenen Erkrankungen eine Insulinkomatherapie bekamen, Mitte der 1950er Jahre Neuroleptika nur kurzfristig zur Beruhigung oder gar nicht gegeben:[849]

> Ein Beispiel von 1954/55 ist die 26-jährige ausländische Privatpatientin[850] S11705 (Studentin) mit der Diagnose „schizophrene Reaktion", zu deren 109-tägigen Aufenthalt 1954/55 es im Arztbrief hieß: Die Patientin „litt an einer schizophrenen Reaktion und wurde einer kombinierten Insulin- und Elektroschocktherapie unterzogen.[851] Sie erhielt insgesamt 50 Insulin- und 7 Elektroschocks. […]. Unter der beschriebenen Therapie besserte sich der Zustand der Patientin weitgehend. In der darauffolgenden psychotherapeutischen Behandlung [Gruppentherapie, GH] erfolgte eine weitere Konsolidierung. Patientin kann jetzt aus unserer Beobachtung entlassen werden und in ihre Heimat zurückkehren. Eine medikamentöse Therapie erscheint uns derzeit nicht erforderlich, doch wäre Patientin nach Möglichkeit vor Aufregung sowie Überanstrengung zu schützen. Überdies erschiene eine psychotherapeutische Betreuung zweckmässig." Lt. Fieberkurve bekam sie auch an der Klinik keine Neuroleptika. Nach der Insulinkur blieb sie noch einen Monat, in dem auf der Fieberkurve mehrmals Arbeitstherapie und Ausgang eingetragen sind. Nach der Entlassung und einem dreiwöchigen Erholungsurlaub im Waldviertel sollte sie – so im Decursus-Eintrag am Tag der Entlassung – „wieder an der Gruppentherapie von Hr. Dr. Gastager teilnehmen".

> Ein Beispiel für die 1950er Jahre und für die Anwendung von drei der ‚großen alten Kuren' bei der Erstaufnahme ist der14jährige Patient S319 mit der Diagnose Hebephrenie: er blieb 1954/55 sechs Monate an der Klinik und erhielt während dieser Zeit zuerst eine Malariafiebertherapie und anschließend (14 Tage nach dem Ende der Chinin-Tage) eine Insulinkomatherapie mit insgesamt 70 Komata und eine Cardiazolkrampftherapie mit 15 Anwendungen im Koma. Zur Indikation heißt es, dass der Patient weiterhin depressiv, interesse-, antriebs- und kontaktlos sei und sich nicht in

[848] Vgl. oben S. 191.
[849] Dass jedoch die Neuroleptika auch vor 1955 bereits angewandt wurden, zeigt der unten 223f. angeführte Fall des Patienten S2536, der diese bei Aufenthalten nach 2 (!) Insulinkomatherapien und 2facher Lobotomie bekam.
[850] Zu den Kosten gibt in diesem Fall die Kopie eines Schreibens von Hoff Auskunft: „Frl. […] steht an der Psychiatrisch-neurologischen Klinik in Behandlung wegen eines Morbus Bleuler. Die Behandlung wird mindestens 3 Monate in Anspruch nehmen. Die Spitalskosten inklusive ärztlicher Behandlung und aller Spesen werden sich auf ungefähr öS 20.000, -- belaufen." Umgerechnet sind das ca. € 13.500,-- (vgl. https://www.eurologisch.at/docroot/waehrungsrechner/#/, 21.10.2024)
[851] Sowohl die Patientin, als auch ihre Mutter unterzeichneten den Revers zum „I Schock".

> die Gemeinschaft einfüge. Aber auch diese brachte lt. Decursus-Eintrag „keine Veränderung des psychischen Zustandsbildes."

Der Fall weist zwei Besonderheiten auf: erstens wurde der 14jährige auf der Erwachsenenstation behandelt, obwohl vergleichbare Altersgruppen in der Regel auf der Kinderstation therapiert wurden. Zweitens erhielt der Patient bei seiner Erstaufnahme drei verschiedene Koma-, Krampf- und Fiebertherapien. Schizophrene PatientInnen, die zum ersten Mal an der Klinik aufgenommen wurden, erhielten zwar relativ häufig mehrere somatische Therapien,[852] diese PatientInnen waren zum Zeitpunkt der Therapie üblicherweise aber mindestens 18 bis 20 Jahre alt. Eine weitere Ausnahme bildet der folgende Fall.

> Auch die 13-jährige Patientin S902 mit der Diagnose „Schizophrene Reaktion bei antriebsgestörter postencephalit[ischer] Persönl[ichkeit]" bekam bei ihren beiden ersten, nur durch eine Woche getrennten Aufenthalten 1953/54 drei der ‚großen alten Kuren'; bei ihr war im Unterschied zum letztgenannten Fall die Krampf- von der Komatherapie getrennt. Als „Grund der Aufnahme" wurde „Mutismus, Depression, Geräusche-Hören, SM-Gefahr, Fluchttendenzen" angegeben; lt. „Eigenanamnese" war sie ein unerwünschtes Kind. Sie wurde nach der Malariatherapie lt. *Decursus* „probeweise entlassen und zur Kontrolle in 2 Monaten bestellt". Da sie wenige Tage nach ihrer Entlassung wieder von zu Hause weglief, kam sie eine Woche später wieder zur Aufnahme. Nun erhielt sie eine EKT (sechs Anwendungen[853]) und danach eine Insulinkomatherapie mit 50 Komata. Nach der Entlassung lief sie noch einmal von zu Haus weg. Erst als ihre Mutter sich Urlaub nahm, um sich der Tochter intensiver zu widmen, besserte sich nach zwei Monaten ihr Zustand deutlich – so in einem Eintrag aufgrund eines Ambulanzbesuchs – und dürfte dann längere Zeit stabil geblieben sein[854]. Sie kam erst nach 5 Jahren wieder 1959 an die Klinik, nun mit der Diagnose „SMV [Sprung in die Donau] Schizophrenie", und wurde am 5. Tag *auf den Steinhof* überwiesen.

In diesen beiden Fällen lief die Folge der Anwendungen der ‚alten Kuren' von der Malariafiebertherapie zur mit ‚Heilkrampf'- (Cardiazol-‚Schocks') kombinierten Insulinkomatherapie bzw. von der Malariatherapie über eine (vorbereitende) Elektrokrampftherapie zur Insulinkomatherapie. Das Prinzip, dass die Insulinkomatherapie möglichst bald nach dem ersten Auftreten der Krankheit zu geben war, war bei beiden Patienten wegen der eng aufeinanderfolgenden Aufenthalte, die gemeinsam als Erstaufnahme gelten konnten, zu beobachten.

[852] Vgl. die oben S. 215 zitierte Auswertung der Pilotstudie des Projekts zur Therapie bei Erstaufnahmen.
[853] Eine Serie von 6 Anwendungen, meist eine Anwendung alle 2 Tage, war in Wien häufig. Vgl. unten im Kapitel zur EKT.
[854] Anamnese 1959 ohne einen Hinweis auf eine Behandlung in der Zwischenzeit.

Eine Malariatherapie nach einer Insulinkomatherapie bei einer nachfolgenden Aufnahme erhielt der Patient S348 mit der Diagnose „Schizophrenie“: Er war bis zur 7. Klasse Vorzugsschüler gewesen, nun aber bei seiner ersten Aufnahme 1953/54 als 19-jähriger seit einem Jahr „abgesondert, nervös“ und „kaum kontaktfähig“; er erhielt zuerst eine EKT mit acht Anwendungen, dann eine Insulinkur mit 80 Komata und ging danach, in den letzten vier Wochen zur Arbeitstherapie. Beim nächsten Aufenthalt 1 ½ Jahre später[855] (1955, 68 Tage Klinikaufenthalt) und mit der Anamnese „Pat ist sehr zerfahren, spricht deutlich faselig und hat während der ganzen Exploration überhaupt keinen Kontakt“ gab der Internisten auch eine Freigabe zur Lobotomie,[856] der Patient bekam jedoch eine Malariatherapie (PST); fünf Tage nach ihrem Ende wurde wieder eine EKT mit acht Anwendungen begonnen.[857]

Das im Untersuchungszeitraum sehr späte Beispiel der 29-jährigen Patientin S20014 (Sekretärin), die 1968 mit der Diagnose „Schizophrenie“ 125 Tage stationär aufgenommen war, zeigt, dass das therapeutische Vorgehen bei Erstaufnahmen von stuporösen Fällen mit der Abfolge EKT und Insulinkomatherapie mit Cardiazol-‚Schock‘ gleich blieb, aber nun mit Neuroleptika ergänzt wurde: im klinischen Befundbericht 16 Tage nach der Entlassung aus der Klinik und unter Einbeziehung des Rehabilitationsaufenthaltes hieß es: „Die Aufnahme erfolgte wegen eines ängstlich gespannten Stupors im Rahmen eines Morbus Bleuler. Es wurde an der Klinik eine Elektrokonvulsions-Serie durchgeführt [12 Anwendungen], anschließend eine komplette Insulinkur[858] mit insgesamt 60 Vollschocks[859] [die Patientin wurde immer mit 1,5 mgr. Glucagon i. m. geweckt]. Es konnte in den letzten Wochen die produktive Symptomatik völlig zum Abklingen gebracht werden und die Pat. war in der Rehabilitierungsstation [Maria Lanzendorf] sehr gut angepasst. Es bestand das Bild einer vollen Remission. Wir empfehlen die derzeitige Medikation weiter zu führen [lt. Fieberkurve: 3x5gtt Haloperidol[860]+ 2 Tbl Distraneurin[861]]. Ausserdem scheint ein Erholungsurlaub mit Domizilwechsel von 3 – 4 Wochen Dauer unbedingt vor Wiederaufnahme der Arbeit angezeigt. Gelegentliche Kontrollzuweisungen an die Klinik werden erbeten.“ In den 1960er Jahren dürfte sich die Kombination mit einer (mittelfristigen) Medikation von Neuroleptika durchgesetzt haben, nachdem sie in der zweiten Hälfte der 1950er Jahre zunehmend fallweise gegeben wurden.

---

855 D. h. 2 ½ Jahre nach der von der Mutter bei der Erstaufnahme angegebenen psychischen Veränderung.

856 Aus den Akten ist mehrmals zu ersehen, dass kurz nach der Aufnahme, im Indikations-Prozess vom Patienten / von der Patientin oder von seiner / ihrer Vormundschaft Reverse für mehrere Therapien und vom Internisten mehrfache Freigaben eingeholt, dann aber nur eine oder keine dieser Therapien gegeben wurden.

857 Nach der Malariatherapie hatte es im Decursus-Eintrag geheißen: „Bei einer Exploration ist Pat. ruhig, kaum kontaktfähig, sehr freundlich, Affektdissoziation, leicht ablenkbar, faselig.“

858 Insulin+1A Vit. B-Kompl. + 2Tbl. Mevasine (Mecamylamine).

859 Mit 8 Cardiazolschocks im 21., 23., 27., 28., 32., 34., 38. und 39. Koma ‚aufgesetzt‘.

860 „Haloperidol ist ein hochpotentes Neuroleptikum aus der Gruppe der Butyrophenone und wird unter anderem zur Behandlung akuter Erregungszustände und akuter und chronischer schizophrener Syndrome eingesetzt“. http://de.wikipedia.org/wiki/Haloperidol (22.1.15). Es handelte sich hier nicht um die „Haloperidolkur“, die in Wien selten angewandt worden sein dürfte (unerwünschte Nebenwirkungen waren etwas häufiger als bei der Majeptilkur). Sie wurde aber in ARNOLD, Die Therapie der Schzophrenie (1963), S. 81f. in ihrem ähnlichen Verlauf wie die Majeptilkur beschreibt. Begonnen wurde sie mit 1mg als Einzeldosis bzw. 2mg als Tagesdosis i. m. oder der doppelten Dosis p. o. und „je nach Reaktion, rasch bis maximal 5mg als Einzeldosis und 25mg als Tagesdosis“ gesteigert; wie bei der Majeptilkur sollte die „Krampfphase“ erreicht werden (Vgl. Arnold 1963 unten S. 286).

861 Clomethiazol, beruhigend; http://www.pharmawiki.ch/wiki/index.php?wiki=Clomethiazol (8.8.2016).

> Im Extremfall des 1929 geborenen Patienten S4570 mit der Diagnose „Schizophrenie“ wurden folgende Therapien und Therapiekombinationen in fünf, zwischen September 1957 und Jänner 1960 rasch aufeinanderfolgenden Aufenthalten an der Klinik gegeben: beim ersten Aufenthalt erhielt er eine Insulintherapie mit 66 Komata und gegen Ende 4 E-‚Schocks‘ und 2 Cardiazol-‚Schocks‘ im Koma ‚aufgesetzt‘; nach dem *Decursus* war durch die Insulintherapie eine deutliche Besserung eingetreten; es wurden hier auch die Teilnahme an der Gruppentherapie erwähnt und zwei psychotherapeutische Gespräche mit Raoul Schindler eingetragen. Dem Krankenakt liegt außerdem ein Protokoll der „Elterngruppe“[862] bei. Nach der Entlassung war der Patient weiter in Gruppentherapie. Bei diesem 1. Aufenthalt sind Medikamente (anfangs und unregelmäßig häufig 1gr Medinal und manchmal 2 Amp. Largactil), aber keine regelmäßigen medikamentösen Therapien eingetragen. Etwas anders beim zweiten Aufenthalt: nun erhielt er schon im Monat vor der Lobotomie als medikamentöse Therapie 3 Wochen lang täglich (meistens 3 Tbl.) Largactil und vor der Lobotomie 8 Tage lang täglich 4x 2 Tbl. „Homburg“ (Prothipendyl-Hydrochlorid)[863]; parallel zu den Medikamenten bekam er eine EKT mit 8 Anwendungen; 2mal ist auf der Fieberkurve „Psychotherapie“ vermerkt; am letzten Tag wurde er um 6:45 zum Transfer auf die I. Chrirurgie zur Lobotomie mit 1 Amp. Atropin, 2 Amp. Phenergan (Promethazin)[864] und 0,6g Pentothal i. v. vorbereitet. Nach der Lobotomie kam er zum zweiten Teil der zweiten Aufnahme zurück: als medikamentöse Therapie erhielt er ab der ersten Woche nach der Lobotomie täglich 3x 2 Tbl. à 80 mg oder 3x 1 Amp. „Homburg“, anschließend sehr lange täglich 2x 2 Amp. oder 2 Tbl., dann auch 3x 3 Tbl. Largactil; 8 Wochen nach der Lobotomie bekam er wieder 2 EKT-Serien mit 10 bzw. 4 Anwendungen; der Patient ging bei diesem langen Aufenthalt häufig zur Arbeitstherapie, manchmal zur Gymnastik, lt. sehr lückenhaftem *Decursus* auch in Gruppentherapie. Beim 3. bis 5. Aufenthalt (1959/60) bekam er EKT und durchgehend Truxal (2-3x 2 ½ Drg.s oder 50mg i. m.).

Zur Anwendung der Lobotomie, des schweren chirurgischen Eingriffs, der 1949 mit dem Nobelpreis für António Egas Moniz ausgezeichnet und im Untersuchungszeitraum weit verbreitet angewandt wurde, folgt ein kurzer Exkurs. Dieser soll die Stellung der Lobotomie im Rahmen des Wiener ‚Gesamtbehandlungsplans‘ als *ultima ratio* und die Anwendung der ‚großen‘ körperlichen Kuren und der neuen Psychopharmaka an der ‚Klinik Hoff‘ nach dem chirurgischen Eingriff verdeutlichen.

---

[862] Dieses Protokoll ist ein Hinweis auf die von Raoul Schindler (der gemeinsam mit Till Tesarek dieses Protokoll verfasste) entwickelte bifokalen Gruppentherapie für Schizophrene; vgl. ARNOLD/SCHINDLER, Bifokale Gruppentherapie bei Schizophrenen, passim.

[863] Im Versuchsstadium mit der Bezeichnung „Homburg D 206“ – HIFT/KRYSPIN-EXNER, Prothipendyl-hydrochlorid (1958), S. 664-668 – und heute noch als Schlaf- und Beruhigungsmittel unter dem Namen „Dominal forte“ auf dem Markt.

[864] Promethazin ist ein beruhigender, antipsychotischer und antiallergischer Wirkstoff aus der Gruppe der Antihistaminika der 1. Generation und der Phenothiazine (http://www.pharmawiki.ch/wiki/index.php?wiki=Promethazin, 21.1.2017). Zur Anwendung in Kombination mit Largactil bzw. Largactil als Alternative lt. HOFF, Lehrbuch der Psychiatrie (1956), S. 409, vgl. unten S. 275 Anm. 1033.

Exkurs: Fallbeispiele mit Lobotomie[865]

Blieb ein Erfolg der anderen körperlichen Therapien bei psychiatrischen PatientInnen aus, wurde auch in Wien in den 1950er Jahren und in Einzelfällen auch noch bis 1964[866] als oberste Stufe in der Hierarchie der körperlichen Behandlungsmethoden die Durchführung einer Lobotomie in Betracht gezogen.[867] Nach dem chirurgischen Eingriff wurden – direkt im Anschluss und in nachfolgenden Aufnahmen – weiterhin nicht nur medikamentöse, sondern auch ‚große' alte Kuren angewandt – wie im letztgenannten Beispiel des Patienten S4570 Elektrokrampfserien. 1960 schrieb der Wiener Kliniker Raoul Schindler im von Hans Hoff herausgegebenen Sammelband „Therapeutische Fortschritte in der Neurologie und Psychiatrie" zu diesen Weiterbehandlungen mit ‚alten Kuren' nach einer Lobotomie, man sollte „lieber zunächst zu wenig eingreifend operieren, da ja die Möglichkeit einer Relobotomie durchaus" offenstehe und sich auch oft zeige, „daß nach der Lobotomie eine erneute Ansprechbarkeit auf die geläufigen Schocktherapien eintritt".[868] Die im Folgenden zitierten Fälle aus den 1950er Jahren zeigen außerdem deutlich Kontinuitäten wie auch Veränderungen in der Therapie der Schizophrenie in Wien.

---

[865] Im Forschungsprojekt war nicht vorgesehen, die Lobotomie umfassend in die Untersuchung einzubeziehen. So ist die Datenbank mit Fokus auf die ‚großen Kuren' der Zwischenkriegszeit auf die Auswertung der Akten der PatientInnen mit den fünf Diagnosen eingerichtet. In ihr wurde die Lobotomie bei diesen, den Einschlusskriterien entsprechenden PatientInnen vermerkt, soweit sie während des Aufenthalts operiert wurden oder vor einer (neuerlichen) Aufnahme lobotomiert worden waren. Daraus wurden die folgenden Beispiele gewählt, die die Position der Lobotomie im ‚Gesamtbehandlungsplan' der Klinik verdeutlichen.

[866] Obwohl Raoul SCHINDLER, Fortschritte der Psychochirurgie (1960), positiv über die Lobotomie schrieb, die 1947 ins „Behandlungsrüstzeug" der Klinik aufgenommen worden war (ebd., S. 479), dürfte sie in 1960er Jahren in Wien kaum mehr angewandt worden sein. Von den in der Datenbank aufgenommenen Fällen war der letzte (1964) jener der 1926 geborenen Patientin S17448 mit der Diagnose „paranoide Schizophrenie" und ihre Lobotomie wurde nicht von der Klinik indiziert. Sie kam – mit Begleitschreiben – von der „Heilanstalt Ybbs mit der Bitte um Lobotomie" an die Klinik, wo sie kurz vor der Operation und wieder am selben Tag der Operation – nun mit der Diagnose „Schizophrenie, Zustand nach Lobotomie, vegetative Erschöpfung" – übernommen wurde; die Patientin starb 9 Tage nach der Operation; lt. Bericht des Chirurgen war die „Indikation: Langjährige Psychose bei der wegen therapieresistenter Aggressions- und Zerstörungshandlungen die Lobotomie psychiatrisch indiziert wurde."

[867] HOFF, Lehrbuch der Psychiatrie (1956), S. 484-488, vgl. S. 487: Entsprechend einer strengen Indikationsstellung sei „die Zahl der bei uns durchgeführten Operationen wesentlich geringer als in Amerika und England. Von unseren 121 Fällen stehen jedoch 67 draußen im Leben", d.h. sie konnten in ein soziales und berufliches Umfeld (re)integriert werde. Obwohl der Zeitraum nicht angegeben wird, ist anzunehmen, dass die 121 Fälle auch die Jahre vor der ‚Ära Hoff' umfassen: HUBER, Ueber Psychochirurgie (1950), S. 956-958. berichtet über 64 Operationen in den letzten 3 Jahren in Wien auf Indikation der Psychiater-Neurologen: „Schizophrenie, Melancholie, Zwangsneurosen, erethische Imbezillität, Paranoia"; „zur Bekämpfung des unstillbaren Schmerzes", einmal „wegen thalamischer Schmerzen […], zweimal wegen Phantomschmerzen", neunmal „wegen Schmerzen bei Metastasen". Vgl. auch Hoff-Skriptum, Allgemeine Psychiatrie [um 1961], S. 69f., zitiert oben S. 202.

[868] SCHINDLER, Fortschritte der Psychochirurgie (1960), S. 474 – weitere Zitate daraus oben S. 157 im Kapitel zur Malariafiebertherapie.

Elektrokrampftherapie, wie im oben zitierten Fall, ist in den PatientInnenakten auch nach einer Lobotomie – anschließend und / oder bei nachfolgenden Aufnahmen – in mehreren Fällen eingetragen.[869] In zwei Fällen erhielten Patienten, Anfang der 1950er Jahre, nach mehreren Klinikaufenthalte mit ‚großen alten Kuren' und nach einer Lobotomie bei einer neuerlichen Aufnahme auch eine Malariafiebertherapie: Der Patient S235 1952 als 29-jähriger bei seinem 4. Aufenthalt mit der Diagnose „Pfropfschizo, Stammhalter" (und der Beschreibung „Pfropfhebephrenie, Zustand nach Lobotomie. Endzustand");[870] der Patient S656 als 21jähriger bei seinem 4. Aufenthalt 1953 mit der Diagnose „Hebephrenie, Zustand nach Lobotomie".[871]

Eine Insulinkomatherapie dürfte für eine Behandlung nach einer Lobotomie aufgrund der Zeitspanne, innerhalb der sie nach dem ersten Auftreten schizophrener Symptome für sinnvoll gehalten wurde, nicht in Frage gekommen sein. Es dürfte sich also um eine Komatherapie vor der Lobotomie gehandelt haben, wenn Arnold 1954 „über die Kombination von Insulin und Heilkrampftherapie mit der Lobotomie" schrieb, es würden „noch sehr wenige Erfahrungen vor[liegen]", doch ließen sich in einigen Fällen „mit diesen Kombinationen manchmal doch Erfolg erreichen".[872] Beim folgendem Patienten handelte es sich nach der Lobotomie um eine Subkomatherapie. Er ist in der Länge seiner Aufenthalte, in der massiven Behandlung mit ‚großen' körperlichen Kuren und – nach den Akten – als Erfolgsgeschichte ein Extremfall. Seine Aufenthalte, die bis vor den Untersuchungszeitraum zurückreichen, zeigen deutlich sowohl Kontinuitäten der ‚Ära Kauders' in die ‚Ära Hoff', als auch die Veränderungen der 1950er Jahren:

> Der 1928 geborene Patient S2536 mit der Diagnose Schizophrenie, der ein Medizinstudium begonnen hatte, hatte von 1948 bis 1955 neun lange bzw. aneinandergereihte Klinikaufenthalte. Beim 625tägigen 4. Aufenthalt 1949/50 bekam er eine 2. Insulinkomatherapie mit 90 Komata und mehrmals ‚E-Schocks' im Koma ‚aufgesetzt' und danach wurde er 2mal lobotomiert. Nach der 2. Lobotomie bekam er – wie bereits 1948 – eine Insulinsubkomatherapie mit ‚E-Schocks' im Subkoma. Diese Anwendung dürfte in der ‚Ära Hoff' nicht oder kaum mehr vorgekommen sein. Ebenso wenig die beiden Acethylcholinschock-Behandlungen, von denen 1950/51 – also bereits

[869] Lt. HOFF, Psychochirurgie (1951), S. 439, habe „Freeman festgestellt […], daß auch die Schockbehandlung der Psychosen nach der Lobotomie mit besserer Aussicht auf Erfolg durchgeführt werden kann." Vgl. auch ebd., S. 486: „Manchmal wurde mit Erfolg versucht, eine Elektroschockbehandlung an die Lobotomie anzuschließen."
[870] Er wird oben S. 157 im Text zur Diskussion über die Plasmodienstammträger vom Steinhof erwähnt.
[871] 17 Monate nach der „b[ei]ds[eitigen] Lobotomie (seitlich)" erhielt er eine Malariatherapie mit acht Fieberschüben.
[872] ARNOLD, Schockbehandlungen (1954), Teil II, S. 72. Vgl. zur „Psychochirurgie" BLEULER, Lehrbuch der Psychiatrie ([10]1960), S. 169-171.

im Übergang zur ‚Ära Hoff‘ – berichtet wurde.[873] Zunehmend wurden aber Dämmerkuren[874] und die neuen Medikamente kurz zur Sedierung (Medinal und Paraldehyd) und als Therapie (die Neuroleptika Largactil und Serpasil[875]) angewandt. Der Kliniker Wilhelm Solms,[876] der sich sehr um die berufliche Eingliederung des Patienten bemühte, berichtete in zwei Gutachten 2 ½ und 3 Jahre nach der letzten Entlassung von 1955 von sehr guten Erfolgen des Patienten bei Prüfungen zur Berufsausbildung, auch dass er den Führerschein gemacht habe, und beurteilte ihn als „in persönlicher und arbeitsmässiger Beziehung angepasst und geordnet“ und „frei von Krankheitssymptomen, der Störung, die zu seiner Einweisung“ geführt habe. Er scheine „voll arbeitsfähig und ist nach ärztlichem Urteil seiner Arbeit durchaus gewachsen.“

Therapieänderungen von der ‚Ära Kauders‘ in die ‚Ära Hoff‘ zeigten sich auch beim 1926 geborenen Patienten S4636, der 1948 und 1950 (seinem dritten und vierten von 14 Aufenthalten zwischen 1947 und 1960) eine Insulinkomatherapie bekam. Eine zweite Anwendung (außerdem drei Jahre nach dem ersten Auftreten der Krankheit) war in der Ära Hoff nicht üblich, ebenso wenig die Anwendung der ‚(Dauer)schlafkur‘ mit Somnifen[877], die der Patient 1950 und noch einmal im Jänner 1951 – also zu Beginn der ‚Ära Hoff‘ – bekam. Bei seinem nächsten Aufenthalt 1956 bekam er die „Largactil-Schlafkur“ – d. h. die in der Kombination von Largactil mit Phenergan in Wien übliche ‚Dämmerkur‘, bei der er ansprechbar blieb und nicht, wie bei der ‚Schlafkur‘ mit Somnifen, künstlich ernährt werden musste. [878]

Auch bei den PatientInnen, bei denen eine Lobotomie durchgeführt wurde, dürften sich in der Weiterbehandlung an der Wiener Klinik in den 1950er Jahren sukzessive die medikamentösen Therapien gegen die ‚großen‘ körperlichen Kuren der Zwischenkriegszeit durchgesetzt haben.

Die in den vorliegenden Akten vorletzte Lobotomie wurde 1961 bei der 56jährige Patientin S19655 mit der Diagnose „Involutionspsychose“ durchgeführt.[879] Die Patientin, bei der aufgrund des Alters und wohl auch der dem Alter entsprechenden

---

[873] Der Patient bekomme anfangs 0,05 Acetylcholin i. v., dann „gesteiert bis auf 0,4g. Seit Beginn der Behandlung zeigt Pat[ient] keine Schlafstörung mehr, er schläft regelmäßig ohne Schlafmittel. […] war nach Angabe der Pfleger noch nie so gelockert und zugänglich wie jetzt.“ Vor der nächsten Aufnahme Ende 1951 war er „auf eigenen Wunsch wieder [in] ambulante[r] Behandlung mit Elektro- und Acethylcholinschock“. Vgl. MÜLLER, Prognose und Therapie der Geisteskrankheiten (1949), S. 58f.: Mario Fiamberti habe 1937 „sein Vorgehen damit [begründete], daß Acetylcholin als ein für die Nervenleitung notwendiger Stoff spezifisch auf den schizophrenen Grundprozeß des Denkzerfalles einwirke.“ Vgl. HOFF, Lehrbuch der Psychiatrie (1956), S. 484: „Die Erfolge dieser Methode stehen aber auch weit hinter der Insulin- und Elektroschocktherapie zurück.“ Ausführlich zum „shock acetilcolinico“ BALDUZZI, Le terapie di shock (1962), S. 81-110.

[874] Nach der ersten Lobotomie (1949/50) „wurde eine Dauerschlafkur versucht mit Chloralhydrat“, dem ersten synthetisch hergestellte Schlafmittel; es gehört zur Stoffgruppe der Aldehydhydrate (https://de.wikipedia.org/wiki/Chloralhydrat; 1.3.2017).

[875] Zur Kombination bzw. Abfolge von Serpasil und Largactil vgl. GHERARDUCCI, A propos de l’utilisation de la chlorpromazine associée a d’autres thérapeutiques psychiatriques (1956), S. 889. Vgl. auch unten S. 276 HOFF (Vortrag 1955, Publikation 1956) Serpasil zur Fortsetzung von Largactil wegen dessen „Gewöhnungseffekt“.

[876] Seit 1955 habilitiert.

[877] Diese Kur geht auf den Schweizer Psychiater Jakob Klaesi zurückgeht, der 1922 empfahl, mit dem Barbiturat Somnifen fünf- bis zehntägige Schlafkuren bei schizophrenen Patienten durchzuführen; vgl. https://de.wikipedia.org/wiki/Schlaftherapie (23.1.2017).

[878] Zur Kombination von Largactil und Phenergan (Promethazin, als Sedativum) vgl. das Zitat aus HOFF, Lehrbuch der Psychiatrie (1956), S. 409, unten S. 275 Anm. 1033.

[879] Zur in der Datenbank letzten Patientin mit einer Lobotomie (S17448 1964) vgl. oben S. 222 Anm. 865.

Diagnose keine Insulinkomatherapie vorzusehen war, wurde wegen Schlafstörungen und paranoiden, schweren Angstzuständen aufgenommen; bei ihrem 107-tägigen Aufenthalt, bei dem auf der Fieberkurve und in den Decursus-Eintragungen mehrmals über Tage extreme Unruhe verzeichnet wurde, bekam sie vor der Lobotomie eine Elektrokrampftherapie mit 6 Anwendungen und Medikamente zur Beruhigung (Librium, Pentothal, Largactil, Plexonal, Doca u.a.m.). 44 Tage nach der Aufnahme wurde sie lobotomiert; danach bekam sie ab dem 3. Tag regelmäßig Neuroleptika (2 – 3mal tgl. 2 – 3 Tabl. Largactil dann auch zusätzlich am Abend um 20h 2 A Largactil, manchmal + 1 A Phenergan). Auch bei ihren nächsten Aufenthalten 1963, 1966 und 1967 wurde sie medikamentös behandelt und nur einmal mit einem „Narko E-Schock“ (Anwendung unter Narkose).

2.2.4.2 Insulintherapie bei PatientInnen mit affektiven Störungen

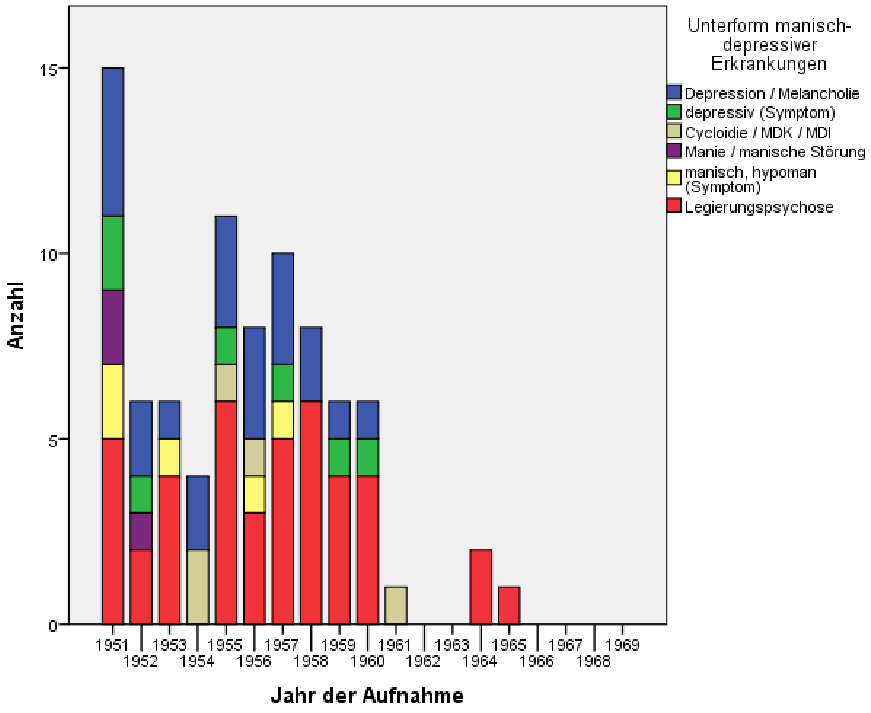

[redacted]

Abb. 10 Gestapeltes Balkendiagramm, Insulinkoma- bzw. -subkomatherapie + Unterformen der manisch-depressiven Erkrankungen (1951-1969), Pat. länger als 4 Tage stationär, n = 84, Mehrfachnennungen bei Diagnosen sind möglich.

Aus dem Balkendiagramm zur Insulintherapie bei den Unterformen der manisch-depressiven Erkrankungen[880] wird deutlich, dass PatientInnen mit affektiven Störungen insgesamt selten eine Insulinbehandlung bekamen und dass ab 1962 von den in der Datenbank aufgenommenen PatientInnen ausschließlich PatientInnen mit der Diagnose Schizophrenie mit einer Insulinkur behandelt wurden: die zwei Fälle im Jahr 1964 und der eine Fall 1965 sind Legierungspsychosen, die herausgelöst aus den affektiven Störungen zu den

[880] Zu den Vergleichszahlen vgl. oben Tab. 1 S. 34, sowie S. 100 Anm. 352.

schizophrenen Erkrankungen zuzuordnen sind. Sobald die weiteren Diagnosen bzw. Zusätze zur Diagnose auf der Diagnosezeile am Deckblatt einbezogen werden, zeigt sich, dass unter den 84 PatientInnen, die mit der Diagnose einer affektiven Störung eine Insulintherapie erhielten, 51 PatientInnen sind, die eine Legierungspsychose oder eine Schizophrenie mit manischem Bild (3mal) oder eine Schizophrenie mit depressivem Bild (1mal) als Diagnose hatten, also aufgrund schizophrener Erkrankung oder Symptome eine Insulinkur als Komatherapie bekamen. Hans Hoff beschrieb in seinem Lehrbuch von 1956 die Insulinkur bei manisch-depressivem Krankheitsgeschehen nur in einem Fall und als kombinierte Insulin-ES-Kur: Wenn „auftretende Wahnideen und paranoid-halluzinatorische Bilder im Rahmen dieses Syndroms [„jener Sonderformen, bei denen Schuld-Sühnetendenzen, Vernichtungsideen oder Agitation im Vordergrund stehen“] […] die Prognose um so mehr [verdüstern,] je weniger primäre traurige Verstimmung und Angst das Bild beherrschen“, würde es sich „öfters um Legierungspsychosen [handeln], die wie paranoide Schizophrenien zu behandeln sind. Bei Nichtansprechen auf ES-Behandlung“ könne man „den Versuch einer kombinierten Insulin-ES-Kur unternehmen.“[881]

Fallbeispiele lassen darauf schließen, dass auch die verbliebenen 33 PatientInnen, die als einzige Diagnose eine Depression (22mal), eine MDK (4mal), eine Manie (4mal) oder eine Kombination aus Neurose und Depression (3mal) vermerkt hatten, zumindest nach Mitte der 1950er Jahre[882] eine Insulinkomatherapie aufgrund von schizophrenen Symptomen bekamen, die nicht als Diagnose, aber manchmal im *Decursus morbi et therapiae* oder in den psychologischen Befunden erwähnt werden. Ohne schizophrene Symptome und/oder mit einer anderen therapeutischen Zielsetzung bekamen sie manchmal eine Insulinsubkomatherapie ohne Koma.

Bezogen auf die Insulinbehandlung von PatientInnen mit affektiven Störungen zeigte sich, dass von 23 PatientInnen (24 Fällen), bei denen in der Diagnose auf der ersten Seite des Umschlagblattes eine affektive Störung ohne Hinweis auf Schizophrenie (wie legiert oder paranoid) gegeben worden war, eine Patientin bei verschiedenen Aufenthalten eine Subschock- und eine Komatherapie bekam, fünf PatientInnen bekamen eine Komatherapie und 17 eine Subschocktherapie. Die im Folgenden zitierten Fallbeispiele zeigen, dass a)

---

[881] HOFF, Lehrbuch der Psychiatrie (1956), S. 511.
[882] Vgl. oben S. 190 die Ablehnung der Insulinkomatherapie „bei anderen Erkrankungsformen“ durch HIFT/HOFF, Die organische Therapie der Psychose (1958), S. 1045.

PatientInnen mit affektiven Störungen vor allem aufgrund von schizophrenen Symptomen eine vollständige Komakur bekamen, und dass b) PatientInnen mit affektiven Störungen aber ohne schizophrene Symptome meistens eine Subkomatherapie bekamen.

a) Hinweise auf schizophrene Symptome als Grund für die Insulinkomatherapie bei affektiven Störungen:

> Deutlich wird 1954 bei der 21-jährigen Patientin A11621 mit der Diagnose „Manische Phase, MDK (legiert?)[883]", dass die Komatherapie bei PatientInnen mit affektiven Störungen in Zusammenhang mit schizophrenen Symptomen stand: in den psychologischen Tests war bei ihr „das Bild einer manischen Persönlichkeit mit psychopathischen (schizoiden) Zügen" bzw. waren deutliche „Schizo Zeichen" festgestellt worden. Sie erhielt eine Insulinkomatherapie bis zum 44. Koma; zu diesem Datum ist in einer von Arnold unterfertigten Abschrift des *Decursus morbi et therapiae* eingetragen: „Pat[ientin] wird heute aus dem Insulin genommen**,** nachdem die Diagnose einer Schizophrenie nicht mehr aufrechterhalten werden kann."

> Bei der Patientin A20841, die als 22jährige bei ihrer 1. Aufnahme 1959/60 mit der Diagnose „hypochondrische Depression" eine Insulinkomatherapie mit 49 Komata erhielt, war vermutlich intern bereits die Diagnose auf Schizophrenie geändert worden, die bei den folgenden 3 Aufnahmen gestellt wurde.

> Bei der 22-jährigen Patientin A15910 mit der Diagnose „SMV, Cycloidie", die 1961 eine Insulinkomatherapie mit 49 Komata bekam, wurde im Entlassungsbrief erwähnt: „Psychologischer Test: Depressive Persönlichkeit mit leichter Abweichung zur Legierungspsychose möglich, stärkere Affektstörung, deutlich dysphorische Stimmungslage, bestehende suicidale Bereitschaft und paranoide Reaktionsbereitschaft angedeutet."

Diese Fälle waren nach der Mitte der 1950er Jahre. Vorher war der Bezug der Komatherapie zu schizophrenen Symptomen manchmal nicht oder nicht so deutlich gegeben.

> So wäre 1951 beim 59jährige Privatpatient A1463 mit der Diagnose „Depression mit paranoiden Zügen" zwar möglich, dass die Therapie mit 29 Komata aufgrund der Zusatzdiagnose „mit paranoiden Zügen" gegeben wurde, gegen eine Insulinkomatherapie bei einem schizophrenen Patienten sprechen jedoch sein hohes Alter und die Zahl der Komata, die nicht der ‚Mindestzahl' von 40 bis 50 entsprach.

In der Zeit vor der Übernahme der Leitung durch Hans Hoff (1950/51) wurde die Insulinkomatherapie mit deutlich weniger als 40 Komata auch in Wien PatientInnen mit affektiven Störungen gegeben. Häufig waren bei ihnen aber auch schon in diesen Jahren Subkomatherapien vorgesehen.

> Ein Beispiel für beides ist die 1899 geborene Patientin A13591 mit der Diagnose „Melancholie" bzw. „endogene Depression". Sie bekam bei der 5. Aufnahme 1948 –

[883] Die Klammerbemerkung dürfte ein Nachtrag sein**.**

also mit dem für die Komatherapie bei Schizophrenie (zu) hohen Alter von 49 Jahren – eine Insulinkomatherapie mit 21 Hypoglykämien (18 Komata mit „genährt“, d. h. dass sie mit der Gabe von Glukose über eine Sonde aus dem Koma geweckt werden musste, und drei Subkomata mit „*getrunken*“ – d. h. dass sie selbst Glukosewasser trinken konnte –; bei den drei Subkomata wird der Schweißausbruch vermerkt) bei Dosen von 80-60-40 Einheiten und anschließend noch 11mal 10-20 Einheiten (als Subschocktherapie ohne Schweiß, mit „getrunken“ bzw. „gegessen“, mit „Zittern“ und „Herzklopfen“, ohne Gewichtszunahme). Bald nach Beginn der Insulinkomabehandlung trat lt. Decursus-Aufzeichnungen eine Besserung ein: weniger ängstigende Selbstvorwürfe, besserer Schlaf. Bei der 6. Aufnahme 1949 mit der Diagnose „Melancholie, Suicidversuch“ bekam die Patientin lt. Fieberkurve 9 E-Schocks und eine Insulinsubkomakur mit 49 Insulingaben und diesmal einer deutlichen Gewichtzunahme von 49,6 kg auf 56,1 kg. Bei ihrem 7. und letzten, 84-tägigen Aufenthalt 1957/58 mit der Diagnose „rez. endogene Depression“ („nach einem Intervall von 8a neuerlich einen Depressionszustand“) bekam sie 2 Elektrokrampf-Serien mit insgesamt 12 Anwendungen; sie ging zur Arbeitstherapie und bekam als medikamentöse Therapie fast durchgehend zur Sedierung täglich 4mal 3 Tabletten Biobamat und 2 Tabletten Plexonal.

b) Die Insulinsubkomatherapie bei affektiven Störungen

Wie bei den anderen Therapien, wird auch die Zielsetzung für die ‚Insulinsubschocktherapie‘ – mit der Gabe von üblicherweise 10 bis 30 Einheiten täglich, manchmal auch deutlich mehr und bis zum starken Schweißausbruch jedenfalls aber ohne Koma – in den PatientInnenakten nicht ausführlich erklärt. Aus Hinweisen in PatientInnenakten ist jedoch zu erschließen, dass die appetitanregende und / oder die beruhigende Wirkung zur Indikation führte, in anderen Fällen die ‚Schockwirkung‘ an Stelle einer Elktrokrampftherapie, die man auch von der Unterzuckerung ohne Koma erwartete. Zu diesen drei Anwendungen werden im Folgenden Beispiele gebracht.

Noch 1971 haben Walter Schulte und Rainer Tölle in ihrem Handbuch der Psychiatrie unter „weitere[n] antidepressiv wirkenden Verfahren“, falls die Thymoleptika nicht wirken, „die kleine Insulinkur“ neben Schlafentzug, „zwischenzeitlich[er] Behandlung mit kleinen Dosen eines intensiven Neuroleptikums“ und „Pyrifer-Fieberkur-Behandlung“ genannt. „Gemeinsames Prinzip aller dieser Maßnahmen“ sei „offenbar eine nicht bekannte psychovegetative Umschaltung.“[884] Auch die Insulinsubkomatherapie wurde im „Gesamtbehandlungsplan“ nicht alleine, sondern mit anderen Therapien gegeben: Die

[884] SCHULTE/TÖLLE, Psychiatrie (1971), S. 226. In der 3. Auflage 1975 (S. 234) wird die Pyrifer-Fieberkur nicht mehr erwähnt.

Elektrokrampftherapie war bei den PatientInnen mit affektiven Störungen häufig in den 1950er Jahren mit der Subkomatherapie kombiniert.[885] Psychotherapie wurde – wie üblich – nur in Einzelfällen in den Krankenakten erwähnt.[886]

ba) Insulinsubkomatherapie zur Gewichtzunahme und Kräftigung

Bei „Nahrungsverweigerung" wurde eine Insulinbehandlung unter Vermeidung eines Komas bereits in den 1920er Jahren gegeben, „wobei gelegentlich auch die Absicht, eine Mästung herbeizuführen, das Körpergewicht zu heben und dadurch therapeutisch zu wirken, mitgespielt hat."[887]

> Die 27jährige Patientin X14844 mit der Diagnose „psychogene Gewichtsabnahme"[888] erhielt 1960 bei ihrem 72tägigen Aufenthalt eine Insulinsubkomatherapie mit 54 Gaben von Insulin; die Dosis wurde langsam auf 25 Einheiten Insulin gesteigert und dann beibehalten. Laut *Decursus morbi et therapiae* wurde „die Patientin [anfangs] mit Largactil ruhiggestellt"; „von einer aufdeckenden Psychotherapie wurde wegen starker Widerstände der Patientin und fehlender Bereitschaft zu Mitarbeit Abstand genommen" und stattdessen „nur einzelne führende therapeutische Gespräche" gemacht. Die Gewichtszunahme mit den „kleinen Insulindosen und Vit. B-Komplex" ergab 3 ½ kg auf 53,5 kg.

Wie die folgenden Beispiele und die oben genannten mit kurzen Komatherapien zeigen, spielte das Alter bei affektiven Störungen weder in der kurzen Anwendung der Komatherapie noch in jener der Subkomatherapie eine entscheidende Rolle.

> Der 54jährige Privatpatient A4814 aus dem Ausland mit der Diagnose „hypochondrische Depression" erhielt 1960 bei seinem einzigen, 187tägigen Aufenthalt drei Elektrokrampf-Serien (8+4+3 Anwendungen) und – lt. „klinischem Befundbericht", den Hoff bei der Entlassung unterschrieb – „einerseits eine Insulin-Subbehandlung[889] […], andererseits […] allgemein roborierende Massnahmen, Polyvitamingaben, faradische Rollmassage, Massage der Nacken-Schultermuskulatur. Es wurden ausserdem mehrere antidepressive Medikamente[890] angewandt sowie mehrmals 1-2tägige Schlafkuren."

---

[885] Vgl. die 51jährige Patientin A12528.
[886] Vgl. die 29-jährige Patientin A11853.
[887] MÜLLER, Insulinbehandlung (1952).
[888] Also mit einer Diagnose, die üblich nicht zur Aufnahme in die Datenbank führte, weshalb keine Aussage über die Häufigkeit der Diagnose und Therapie gemacht werden kann. Die Patientin hatte Probleme – Schlafstörungen und Gewichtsabnahme – seit 1 ¾ Jahren, seitdem sie die Scheidung gegen ihren Gatten eingereicht hatte.
[889] 12 bis 24 Einheiten Insulin wurden fast ohne Unterbrechung täglich länger als 4 Monate gegeben.
[890] Truxal, Niamid: vgl. GANN, Zur Differentialdiagnose und Therapie der Depressionen. Der parenterale Niamidstoß (1964), S. 588-592 und S. 609-612.

> Die 50jährige Patientin A11675 mit der Diagnose „Medikamentenmissbrauch; rezidivierende Depression“ erhielt 1954/55 während ihrer einzigen, 101tägigen stationären Behandlung an der Klinik eine EKT mit 4 Anwendungen und danach zwei Insulinsubkomatherapien mit 10 bzw. 31 Gaben von 10 bis 50 Einheiten Insulin;[891] parallel zur 2. Insulinsubkomabehandlung bekam sie einen Monat lang das Neuroleptikum Largactil.[892] Während einen Monat nach der Aufnahme „beträchtlicher“ Gewichtsverlust und fünf Tage später „eine wesentliche Verschlechterung der Pat[ientin]“ eingetragen wurde, hieß es zwei Wochen danach: „Die Pat[ientin] macht nun Insulinsubkoma durch, die nicht nur eine Besserung des körperlichen Befindens, eine wesentliche Gewichtszunahme und Besserung des A[llgemein] Z[ustands] sondern auch psychisch eine Besserung bringen.“

bb) Insulinsubschocktherapie zur Beruhigung

Wie die bisherigen Beispiele nahelegen, dürfte die Insulinsubkomatherapie in Wien häufig in niedrigen Dosen gegeben worden sein, die nur zur Schläfrigkeit und damit zur Beruhigung und zur Stabilisierung führten. Seltener waren die Subkomatherapien mit höheren Dosen, die zu Schweißausbrüchen (zum „choc humide“) und zu starker Dämpfung führen sollten – wie im folgenden Fall:

> Die 1900 geborene Privatpatientin A9838 aus dem Ausland mit der Diagnose „Manie“ hatte bereits bei ihrem Aufenthalt 1948/49 eine Insulinsubkomatherapie bekommen; 1951 bekam sie an die 50mal Insulin in höheren Dosen (70E-95E) wieder als Insulinsubkomatherapie, wie es ausdrücklich im *Decursus morbi et therapiae* hieß. Ab der Gabe von 60 Einheiten wurden – 1 ½ -2 Stunden nach der Insulingabe – immer Schweißausbrüche (manchmal mit Zittern) verzeichnet und diese Subkomata wurden (als jene, die die gewünschte Stärke hatten) gezählt. 1 Stunde später wurde „starkes Hungergefühl“ mit Benommenheit bzw. Schläfrigkeit und eine weitere ½ Stunde danach „*gegessen*“ eingetragen. Hier ging es also um eine „choc humide“-Insulintherapie zur Dämpfung:[893] Nach dem *Decursus morbi et therapiae* wurde die Patientin gegen Ende der Therapie ruhiger, hatte nun „keinen Affektausbruch mehr“ und „sich mit ihrer Situation ausgesöhnt“; so hatte sie ihre Versuche aufgegeben, telefonisch oder „brieflich [Kauf]Aufträge durchzugeben, was sie in den ersten Wochen täglich“ getan hatte.

bc) Insulinsubkomatherapie statt einer Elektrokrampftherapie

---

[891] Schweißausbrüchen werden nicht erwähnt, manchmal jedoch (bei 40 und 50 Einheiten) „genährt“ statt „getrunken“, d.h. dass sie nicht zum selbständigen Trinken aufgewacht oder fähig oder gewillt war; dennoch dürfte es sich um Subschocks gehandelt haben, da kein Koma eingetragen wurde.

[892] „4560“, Studienname für Largactil (Vgl. S. 273-275); tgl. 2 Ampulle i. m.

[893] Für die 51jährige wegen ihres hohen Alters möglicher Weise als Alternative zu einer Komatherapie, nachdem im PI-Test auf eine „ausgesprochen schizoide Reaktion“ hingewiesen worden war.

Wie die Insulinkomatherapie nicht nur häufig in Kombination mit einer Elektrokrampftherapie gegeben wurde, sondern bei manchen PatientInnen (in einer abgekürzten Form) als Ersatz einer Elektrokrampftherapie gegeben werden sollte,[894] so wurde auch die Subkomatherapie manchmal in Kombination[895] und bei vermuteter Kontraindikation der näherliegenden Elektrokrampftherapie auch an ihrer Stelle gegeben:

> So erhielt die Patientin A10115 mit der Diagnose „rezidivierende endogene Depression" als 60jährige bei ihrem 2. Aufenthalt 1952 „Insulin-Subschocks" mit 32 Gaben von anfangs 10, dann 30 Einheiten, nachdem „die bereits im Jahr 1951 durchgeführte Elektroschockbehandlung […] diesmal wegen eines Myokardschadens und einer Hypertonie nicht durchführbar" war.[896]

Die folgenden Fallbeispiele könnten aufgrund der Informationen im Krankenakt als Weiterführung der (erfolglosen) Elektrokrampftherapie durch eine Insulinsubkomatherapie zu interpretieren sein:

> Die 1956/57 bei ihrem ersten, 111tägigen Aufenthalt 31jährige Patientin A13582 erhielt mit der Diagnose „Depression (endogen + psychogen)"[897] eine EKT mit 12 Anwendungen. Nach dieser wurde eingetragen: „Psychisch unverändert, daher Beginn mit Insulinsubschock". Es wurden 40mal 30 Einheiten Insulin gegeben und parallel das Neuroleptikum Largactil, das auch nach dem Ende der Subschocks weitergeführt wurde. Gegen Ende des Aufenthalts bekam die Patientin zusätzlich zum Largactil – gegen die extrapyramidalen Syndrome als unerwünschte Nebenwirkung des Neuroleptikums – Parsidol.
>
> Zur Patientin A13089 hieß es 1956/57 nach ihrem dritten, nun 186tägigen Aufenthalt als 48jährige mit der Diagnose „Manisch-depressives Krankheitsgeschehen, depressive Phase"[898] im Arztbrief: „Wie bereits aus der Vorgeschichte bekannt,[899] zeigen die einzelnen Phasen bei der Pat. eine Tendenz zu ungeheurer Länge und die Neigung in einen chronischen Verlauf überzugehen. Auch diesmal trotzte die Phase einer 2-maligen

---

[894] Bleuler empfiehlt den Ersatz 1960 bei Intellektuellen, zitiert unten S. 181 und S. 241. Ein Beispiel für die Wahl einer kurzen Komatherapie anstatt einer EKT aufgrund der Hypertonie-Diagnose dürfte 1951/52 die Therapie mit nur 15 Komata im Fall der 52jährige Privatpatientin A10008 mit der Diagnose „Depression, Hypertonie" sein. Im Arztbrief Hoffs heißt es: „Der Grund des Depressionszustandes war unter anderem der Tod ihres Gatten. Zeichen einer endogenen Psychose waren bei ihr nicht vorhanden. Es wurden bei der Pat. 15 Insulinschocks durchgeführt, welche bei ihr einen ausgezeichneten Erfolg zeitigten. Sie wurde in geheiltem Zustand aus unserer Klinik entlassen. Da keine Zeichen einer Geisteskrankheit bei ihr vorhanden waren, ist die Prognose sicherlich eine sehr gute."

[895] Im Insulinsubkoma ein 'E-Schock' ‚aufgesetzt': Vgl. oben zum Patienten S1579 S. 217 Anm. 844, und zum Patienten S4636 S. 224.

[896] Im unten S. 254 zitierten Fall der Privatpatientin S14970 mit einer schizophrenen Erkrankung scheint die Insulinsubkur ebenfalls anstatt der vorher geplanten EKT gegeben worden zu sein.

[897] „Im Rorschachversuch Hinweise auf eine depressive Persönlichkeit mit neurotischen (hysteriformen) Zügen. Mögliche <u>paranoide Reaktionsbereitschaft</u>. Kein typisches Bild einer endogenen Depression. Hochgradig affektstörbar, labil. Möglicherweise auch aggressiv" Bei der 2. Aufnahme 1958 mit der Diagnose „rezidivierende Melancholie", wurde sie bereits am 2. Tag auf den *Steinhof* überstellt.

[898] Lt. bezirksgerichtlichem Beschluß über die Anhaltung in einer geschlossenen Anstalt: „mit Selbstvorwürfen, innerer Unruhe, Schlaflosigkeit und Grübelzwang".

[899] Mit Bezug auf die Aufenthalte 1933/34 („Melancholie") und 1951/52 („Melancholie, neurotische Persönlichkeit").

E-Schock-Serie von je 6 E-Schock[s], ebenso einer anschliessenden Insulin-Subschockbehandlung [mit 12mal 10-30 Einheiten, GH]. Erst unter einer weiteren Largactil-Parsidolkur konnte eine gewisse Aufhellung erzielt werden.“ In den Fieberkurven ist auch „Höhensonne“ (über 30mal) und häufig Arbeitstherapie eingetragen.

2.2.4.3 Insulintherapie bei Psychopathie

Wie bei den PatientInnen mit affektiven Störungen, so dürfte die Insulinkomakur bei PatientInnen mit der Diagnose „Psychopathie“ nur bei schizophrenen Symptomen gegeben worden sein. Ohne diese erhielten sie manchmal eine Insulinsubkomatherapie. Diesen Schluss lässt die Nachrecherche in zehn Fällen zu, wovon 7 mit dem Hinweis auf schizophrene Symptome Insulinkomatherapien bekamen und 3 – aus etwas unterschiedlichen Gründen – mit Insulinsubkomatherapie behandelt wurden.

Trotz der Diagnose „Schizophrene Reaktion bei Psychopathie“ bekam der 26jährige Student P3476 1951 bei seinem 41tägigen, ersten von drei Aufenthalten keine Koma-, sondern eine Insulinsubkomatherapie[900] mit 19 Gaben von meistens 70 Einheiten Insulin. Bei jedem der 7 letzten Insulingaben ist „Schweiß“ vermerkt, was der Stärke eines „choc humide“ nach französischer Diktion entsprach. Er wurde 1957 wieder aufgenommen, bekam eine EKT, an 6 Tagen tgl. 2 Amp. Largactil, und wurde schließlich nach Gugging überwiesen.

Der Angestellte P1385 bekam beide Insulinkuren: 1950 als 19jähriger, bei seinem ersten Aufenthalt an der Klinik mit der Diagnose „schizoide Psychopathie, Hebe?“ eine Insulinkomabehandlung mit 33 Komata und im folgenden Jahr 1951 mit der Diagnose „Psychopathie“ zwei Monate lang eine Insulinsubkomatherapie mit 50 Insulingaben; die Dosis wurde auf 60 und 70 Einheiten gesteigert, wobei auch bei diesen Dosen nicht immer „Schweiß“ als Reaktion eingetragen ist; der Eintrag „keine Erscheinungen“ lässt vermuten, dass hier der Schweißausbruch vermisst wurde. Es geht aus dem Patientenakt nicht hervor, ob die Insulintherapie als Subkomatherapie mit „chocs humides“ anstatt einer zweiten Komakur gegeben wurde (da eine Komatherapie kaum 2mal gegeben wurde), oder zur Beruhigung des aggressiven Patienten (er hatte den Vater tätlich angegriffen) oder zu seiner Kräftigung (er nahm im 1. Monat 7kg zu und war vorher am Rosenhügel „vorwiegend roborierend“ behandelt worden) – oder aus mehreren dieser Zielsetzungen.

Beim 1957 14jährigen Patient P4625 mit der Diagnose „Psychopathie“ scheinen Beruhigung und soziale Integration therapeutisches Ziel gewesen zu sein. So wurde Im *Decursus morbi et therapiae* eingetragen: „der zuerst sehr erregte und sehr widerwillig

[900] *Decursus*-Einträge zu seinem Verhalten und zur Subkomatherapie: „Pat. steht nun in Insulinbehandlung, die er sich wenn auch nur widerwillig gefallen lässt. […] Pat. hatte heute seinen 14. Insulin Subschock auf 70 Einheiten Insulin. Er ist reserviert, unterhält sich fast ausschliesslich mit Akademikern und lässt sich höchstens zu einem Schachspiel mit einem Anderen herab.“

auskunftgebende und gehorchende Patient wird unter der Therapie (Insulinsubschock[901] und Psychotherapie) ruhiger und angepaßter.“ Walter Spiel beschrieb denPatienten 1960 in einem Gutachten an die Wiener Jugendgerichtshilfe: diagnostisch handle „es sich um eine psychopathische Persönlichkeit mit stark hypomanischem Einschlag bei einer recht guten Intelligenz (IQ 116). Therapeutisch wurden sowohl entsprechende Tranquilizerkuren gemacht, als auch eine Insulinsubschockkur und eine entsprechend führende Psychotherapie“. Spiel hob hervor, dass sich der Patient an der Klinik sehr positiv verhalten und von hier ohne Probleme die Schule besucht habe; für die Probleme zu Hause, die der Grund für die Aufnahmen waren, trügen auch die Eltern Schuld, weshalb Spiel eine Trennung vom häuslichen Milieu möglichst bis zum Ende seiner Berufsausbildung empfahl.

2.2.5 Zusammenfassung zur Anwendung der beiden Insulintherapien

Die Auswertung der Krankengeschichten erfolgte hier nicht nur aufgrund der Einträge in der Datenbank des Projekts, sondern auch aufgrund einer ausführlichen, aber in der Auswahl unsystematischen Nachrecherche. Der Grund für dieses Vorgehen war, dass bei der Aufnahme in die Datenbank nicht zwischen Koma- und Subkomatherapie unterschieden wurde. Für die Anwendung beider Insulinkuren finden sich auch in anderen Ländern Belege in den beiden Jahrzehnten und darüber hinaus. In den folgenden Zusammenfassungen wird zuerst auf die Subkomatherapie und die Anwendung einer kurzen Komatherapie eingegangen, dann auf die Komatherapie.

2.2.5.1 Die Insulinsubkoma- und die verkürzte Komatherapie (Zusammenfassung)

Die Gabe von Insulin in Dosen, die nicht zum Koma führen, wurde als Therapie auch an anderen Kliniken seit den 1920er Jahren vor allem bei Essstörungen mit Gewichtsabnahme angewandt. Die Subkomatherapie wurde außerdem zur Sedierung der PatientInnen gegeben. Nach der oben zitierten französischen Umfrage zu den beiden Insulintherapien wurde sie auch noch nach 1969, nach dem Ende der Untersuchungsperiode, angewandt. In Wien wurde die Insulinsubkomatherapie nach den zitierten Fallbeispielen in den 1950er Jahren ebenfalls zur Appetitanregung und auch zur Beruhigung gegeben. Manchmal wurde sie hier – die Subkomatherapie wurde in französischen Publikationen ausdrücklich zu den ‚Schocktherapien‘ gezählt – auch bei PatientInnen mit affektiven Störungen nach einer erfolglosen oder anstatt einer kontraindizierten EKT angewandt.[902] Die Höhe der

[901] Lt. Fieberkurve bekam der Patient tgl. 40 Einheiten Insulin und 4x 2 dann 3x 2 Kps. Biobamat (dieses Medikament wurde als Tranquilizer gegeben). Er nahm in 3 Wochen 6 kg (von 45 auf 51kg) zu; die Gewichtzunahme wird jedoch nicht als ein Ziel der Therapie genannt.
[902] Vgl. oben S. 230f.

Dosierungen war unterschiedlich (20 bis 70 Einheiten, üblich mit „*Vitamin-B-Komplex*“) und ebenso die Zahl der Anwendungen (10 bis 126mal).

Mehrmals wurden PatientInnen mit affektiven Störungen in den 1950er Jahren mit einer Komatherapie mit deutlich weniger als den für die Therapie immer wieder als Mindestzahl genannten 40 bis 50 Komata behandelt. Die PatientInnen mit affektiven Störungen, die eine kurze Komatherapie erhielten, waren meistens deutlich älter als die PatientInnen, die aufgrund von schizophrenen Erkrankungen beim oder kurz nach dem ersten Auftreten der Krankheit mit der Standardanwendung der Komatherapie behandelt wurden. Auch die Subkomatherapie wurde nach den Fallbeispielen meistens älteren PatientInnen gegeben. Diese verkürzten Anwendungen der stärker als die Subkomatherapie intervenierenden Komakur bei älteren Personen, aber auch der Subkomakur mit starken Schweißausbrüchen dürften auf eine Anwendung als „Schocktherapie“ („Erschütterungstherapie“ nach Bleuler) bei PatientInnen mit starken inneren Spannungen zur Beruhigung bzw. ‚Umstimmung‘ schließen lassen; die Anwendung der Subkomatherapien mit niedrigen Dosen kombiniert mit Neuroleptika lässt auf eine weniger intervenierende Therapie ebenfalls zur Beruhigung schließen und die Subkomatherapie ohne Neuroleptika auf eine Therapie zur sanften Beruhigung (mit Schläfrigkeit) und zur körperlichen Kräftigung durch Appetitanregung.

Während es sich bei der Anwendung einer Insulinkur bei affektiven Störungen oder bei anderen Diagnosen (etwa Psychopathie) ohne weiteren Hinweis auf ein schizophrenes Geschehen üblich nur um eine Insulinsubkomatherapie mit täglich 10 bis 30 Einheiten (oder je nach Insulinverträglichkeit bzw. erwünschter Stärke der Somnolenz bzw. des erwünschten Schweißausbruchs auch mehr) gehandelt haben dürfte, blieb die Schizophrenie das typische Indikationsgebiet der Insulinkomatherapie über den gesamten Untersuchungszeitraum.

2.2.5.2 Die Insulinkomatherapie (Zusammenfassung)

In der Auswertung der Datenbank zur Praxis an der Wiener Psychiatrie wird deutlich, dass die Insulinkomatherapie fast ausschließlich und wahrscheinlich ab der Mitte der 1950er Jahre ausschließlich bei PatientInnen mit schizophrenen Erkrankungen bzw. ihren Symptomen angewandt wurde. Durch die Einschränkung der Komatherapie auf PatientInnen bis maximal 1 ½ Jahre nach dem ersten Auftreten der schizophrenen Erkrankung betraf die Therapie mit mindestens 40 bis 50 Komata fast ausschließlich junge PatientInnen (unter 25 Jahren). In den

Krankenakten finden sich zur Beschreibung der Symptomatik häufig Formulierungen, die bei den jungen PatientInnen mit frühen schizophrenen Erkrankungen auf ihre inneren Spannungen, auf Verschlossenheit (manchmal wechselnd mit Aggressivität gegen andere oder sich selbst), auf Kontaktlosigkeit und Gedankenflucht hinweisen. Weiters ist das deutliche Sinken des Anteils der Komatherapien bei den an der Klinik stationär länger als 4 Tage aufgenommenen schizophren Erkrankten hervorzuheben.[903]

1960 war Arnold einleitend zu einem Artikel über die Behandlungsmethoden der Schizophrenie der festen Überzeugung, dass „auch heute noch […] die richtig indizierte und geführte Insulinvollschockkur die Basisbehandlung der Schizophrenie dar[stellt]". Als Argument für die Weiterführung der Therapie in Wien ist anzuführen, dass hier in den 1950er Jahren Fortschritte in den Behandlungstechniken und ihrer Abstimmung auf differenzierte Diagnosen erzielt worden sind, die nach den Wiener Autoren zu einer wesentlichen Verringerung der Risiken geführt hatten: Jeder „Fall einer Schizophrenie" sei „nach genauester phänomenologischer Skizzierung seines Quer- und Längsschnittes, nach Erfassung seiner Erb- und Milieustruktur und nach Skelettierung seiner Persönlichkeit in den Rahmen eines Gesamtbehandlungsplanes zu stellen, in dem die körperlichen Methoden nur einen Teil bilden können, wenngleich dieser die Basis jeder anderen Therapie [der Schizophrenie, GH] sein wird."[904] Die Insulinkomatherapie blieb ein Bestandteil des aufgrund der multifaktoriellen Genese der psychischen Erkrankungen geforderten mehrschichtigen ‚Gesamtbehandlungsplans'. Von Hoff wurde – wie auch von französischen Psychiatern – außerdem betont, dass die Insulintherapie als gute Voraussetzung für eine Psychotherapie genützt werden müsse, d.h. in Wien vor allem der Gruppentherapie (auch der bifokalen Gruppentherapie). Während diese in den Akten nur selten verzeichnet, aber aus den Wiener Publikationen zu erschließen sind, wurde die Arbeitstherapie, die als Vorbereitung einer Reintegration der PatientInnen in ihr soziales Umfeld, in ihre bisherige berufliche Tätigkeit galt, in fast allen Fällen in der Fieberkurve vermerkt.

Resümierend zeigt sich nach einer anfänglichen Begeisterung für die Insulinkomatherapie und nach ihrer starken Verbreitung bereits Ende der 1930er Jahre, dass aufgrund der

[903] Zu Gründen für die Abnahme – wie Ausweitung der Anwendung der Psychopharmaka, Spezifizierung der Indikation nach den Arbeiten von Ottokar H. Arnold, Skepsis gegenüber der Therapie von einzelnen Klinikern wie Peter Berner – zu Letzterem vgl. oben S. 188.

[904] ARNOLD, Die körperlichen Behandlungsmethoden der Schizophrenie (1960), S. 262. So auch in anderen Wiener Publikationen, vgl. oben S. 188.

unerwünschten Nebenwirkungen (der manchmal vorkommende spontanen epileptischen Anfälle im Prä-Koma und – besonders unerwünscht – als „Spätanfälle“ danach[905]), aber auch aufgrund der hohen Kosten[906] dieser Behandlungsmethode und aufgrund der vergleichsweise geringen Remissionen schon ab Mitte der 1950er Jahre vermehrt Zweifel an der Sinnhaftigkeit der Komatherapie aufkamen. An der Wiener Klinik wurde sie bis Ende der 1960er Jahre als Therapie bei Schizophrenie, wenn auch mit deutlich abnehmender Häufigkeit angewandt. Wahrscheinlich ist diese Abnahme auf eine Ausweitung der Behandlung nur mit Psychopharmaka und auf eine Einschränkung der Anwendung der Insulinkur auf bestimmte Formen, Symptomatik bzw. Krankheitsverläufe der Schizophrenie zurückzuführen, zu deren Spezifizierung der Wiener Kliniker Ottokar H. Arnold mit seinen Publikationen beigetragen hatte.[907] Vielleicht trug dazu auch die Skepsis einiger Wiener Kliniker gegenüber der Insulinkomatherapie bei.[908] In den vorliegenden PatientInnenakten konnte letzteres aufgrund der fehlenden oder wenig ausführlichen und klärenden Hinweise zur Zielsetzung der Therapie im Einzelfall nicht geklärt werden.

International ging, so Pierre Juillet 1968, die Anwendung der Insulintherapie ebenso wie die der EKT bei schizophrenen Erkrankungen ‹seit dem Aufkommen psychotroper, vor allem neuroleptischer Substanzen› stark zurück; zugleich sei jedoch die Insulinkomatherapie im Vergleich zu den anderen ‹Schocktherapien› die am meisten angewandte, wie auch die am heftigsten diskutierte Therapie geblieben. Als weitere Ursachen für den Rückgang ihrer Anwendung wurde auch von ihm der Zweifel verschiedener Autoren an der Wirksamkeit der Therapie, ihre Gefährlichkeit, sowie der hohe technische und ökonomische Aufwand der Therapie genannt.[909] Auch die Einschränkung der Anwendung auf jene Fälle schizophrener Erkrankungen, ‹in denen der psychotherapeutische Kontakt [bisher] unmöglich› war und bei denen die Neuroleptika nicht besserten, dürfte zur Abnahme beigetragen haben.[910] In Handbuch-Publikationen zur Insulinkomatherapie, wie auch noch 1979/80 in den oben zitierten Antworten auf französischen Umfrage zur Insulintherapie, zeigt sich deutlich, dass

---

[905] Vgl. ARNOLD, Schockbehandlungen (1954), Teil II, S. 70. Vgl. ausführlicher oben S. 196 Anm. 765.
[906] Es geht hier vor allem um die Kosten des langen stationären Aufenthalts; JOST, Zur Insulinempfindlichkeit der Schizophrenen (1958), S. 657 nennt aber auch das Argument der Kosten des Insulins.
[907] Vgl. ARNOLD, Die Therapie der Schizophrenie (1963), passim.
[908] Vgl. unten S. 238 das Zitat aus Berners biographischem Artikel zu Hans Hoff von 1998. Vgl. auch die oben S. 188 erwähnte Stellungnahme Berners in JUILLET, La cure de Sakel est-elle dépassée? (1980), S. 167.
[909] JUILLET, Traitements insuliniques et méthodes de choc dans la schizophrénie (1968), S. 107f. (Übersetzunge aus dem Französischen in einfachen Guillemets von GH).
[910] Ebd., S. 120 – zitiert oben S. 185.

alle diese Unklarheiten, die Kritik und die Vorteile der einfacher anzuwendenden und weniger gefährlichen Neuroleptika zwar zu einer Abnahme ihrer Anwendung führten, nicht aber dazu, dass ihre Anwendung, die aufgrund der persönlichen positiven Erfahrungen erfolgte, allgemein als illegitim gesehen worden wäre.

Wenngleich die Methode auf der empirischen Ebene, d. h. nach den Beobachtungen eines guten Teils der damals damit befassten Ärzte, funktionierte, blieben die Erklärungsversuche ihrer Wirkungsweisen kontrovers. Die psychologische Erklärung, die auch für die Schlafkur und die Malariafieberkur vorgebracht wurde, dass die PatientInnen durch die Art der Behandlung in einen Zustand der Hilflosigkeit und Abhängigkeit vom Arzt und vom Pflegepersonal gerieten, wodurch sie aus ihrer psychischen Isolation befreit und für psychotherapeutische Interventionen empfänglich würden,[911] wurde seit den 1930er Jahren gegeben und scheint bei der langfristigen und sehr pflegeintensiven Insulinkomakur besonders einleuchtend zu sein.[912]

Die Insulinkomatherapie wurde im Rahmen eines „Gesamtbehandlungsplans“ in Kombination mit anderen Therapien gegeben: häufig gingen ihr zur Beruhigung des Patienten / der Patientin eine EKT oder die kurzfristige Gabe von Psychopharmaka voraus; oft wurden einigen der Komata ein E- oder Cardiazol-‚Schock‘ „aufgesetzt“; am Ende der Komatherapie dürfte standardmäßig eine Psychotherapie (meistens Gruppentherapie) begonnen worden sein; auf den Fieberkurven wurden nach der Komatherapie manchmal wieder EKT-Serien und Psychopharmaka als weiterführende Therapie sowie fast immer Arbeitstherapie eingetragen.

In Wien trugen risikomindernde Methoden in der Anwendung wie der Einsatz von Ganglienblockern (Hexamethonium-Derivate) in geringen Dosen, um die Insulinresistenz zu senken und so bei selben Effekten weniger Insulin verwenden zu müssen, sowie – nach Arnold – die „erstklassige Organisation und richtige Indikation“ und „eine riesige persönliche Erfahrung“ der Wiener Kliniker[913] ebenfalls zur langen Anwendung der massiv eingreifenden

[911] SCHMUHL/ROELCKE, Einleitung (2013), S. 22. Vgl. die Zitate aus dem Lehrbuch von Bleuler (Ausgabe von 1960 und 1969) zur unspezifischen Wirkung der „großen körperlichen Behandlungsverfahren“ oben S. 28f.
[912] Vgl. VIJSELAAR, „A Hole in the Armour of Dementia Praecox“. Somatic Cures within a Context of Psychiatry in Multiplicity: the Netherlands 1920-1950 (2013), S. 182f. zu den psychologischen Erklärungen der Erfolge der somatischen Therapien der holländischen Psychiater vor dem 2. Weltkrieg.
[913] ARNOLD, Die körperlichen Behandlungsmethoden der Schizophrenie (1960), S. 265, der die Gefahren aufzeigt und dazu die Risiko-mindernden Fortschritte in der Wiener Therapie nennt; vgl. das Zitat oben S. 190.

körperlichen Behandlungsmethode bei. Auch rechtfertigten die schweren unerwünschten Nebenwirkungen der frühen Neuroleptika (Dyskinesien und Parkinsonoid) eine Zurückhaltung. So kamen die Neuroleptika an der Wiener Psychiatrie zwar bereits in der ersten Hälfte der 1950er Jahre zum Einsatz, noch lange jedoch ohne die Insulinkomatherapie vollständig zu ersetzen.

Die Insulinkomakur wurde hier bis zum Ende der Ära Hoff angewandt.[914] Peter Berner, der Nachfolger von Hans Hoff an der Wiener Psychiatrie, wurde in der französischen Umfrage 1980 mit seiner Skepsis gegenüber der Therapie zitiert und bringt diese auch im Artikel über Hans Hoff 1998 zum Ausdruck: Da Hoff „aufgrund seines ungestümen Temperaments von seinem Standpunkt nicht abzubringen war, wurden die Malaria- und Insulinschockbehandlungen an der Wiener Klinik erst nach seinem Ausscheiden aufgegeben."[915] Mit Berner als Leiter der psychiatrischen Klinik wurde ihre Anwendung eingestellt.

Wie bei der Malariafiebertherapie ist auch bei der Insulinkomatherapie – bei der zweiten der in Wien in der Zwischenkriegszeit entwickelten Therapien – die „Wiener Tradition" als ein möglicher Faktor für das Zögern anzuführen, ‚erprobte' Therapien zu Gunsten neuer Therapien völlig aufzugeben. Hans Hoff wie auch sein Vorgänger in der Klinikleitung Otto Kauders wurden in dieser Tradition ausgebildet und standen in dieser Tradition.

2.3 Die Elektrokrampftherapie[916] und ihre Anwendung an der Klinik Hoff

Beispiele für eine therapeutische Anwendung von elektrischem Strom in der Medizin und in der physikalischen Therapie finden sich seit dem 18. Jahrhundert. Die Idee, mit einem starken „Elektroschock" einen künstlichen epileptischen Anfall auszulösen, um einen günstigen

---

[914] Freilich wurde sie nach RZESNITZEK, „Schocktherapien" und Leukotomie in der DDR-Psychiatrie (2018), S. 299 „mindestens bis Ende der 1960er Jahre" nicht nur in der DDR (Erfurt, Rodewisch, Leipzig, Hochweitzschen), sondern auch „in einigen Kliniken der Bundesrepublik, wie zum Beispiel in Bonn" angewandt. Vgl. auch Hirschmüller oben S. 188.

[915] BERNER, Hans Hoff (1998), S. 60.

[916] In den PatientInnenakten und in der zeitgenössischen Literatur wird von „Insulinschocktherapie" und von „Elektroschocktherapie" (selten von „Elektrokrampftherapie") gesprochen. Diese Bezeichnungen werden in wörtlichen Zitaten beibehalten, im Text wird Elektrokrampftherapie oder EKT verwendet (nicht der damals in deutschen Publikationen seltene und heute übliche Begriff „Elektrokonvulsionstherapie"), aber auch – um die einzelne Anwendung zu bezeichnen – ‚Elektroschock' oder ‚E-Schock'. Gemeinsam werden die in der Zwischenkriegszeit entwickelten Koma-, Krampf- und Fiebertherapien im Text auch ‚große alte Kuren' genannt, ein Begriff der für sie manchmal verwendet wird.

Effekt bei Geisteskrankheiten zu erzielen, setzte allerdings erst der italienische Psychiater Ugo Cerletti um. Theoretischer Ausgangspunkt war die empirische Beobachtung, dass Schizophrenie und Epilepsie nicht gemeinsam auftreten, weshalb ein Antagonismus der beiden Krankheiten angenommen wurde. In der Hoffnung auf eine therapeutische Wirkung epileptischer Anfälle bei PatientInnen mit schizophrenen Erkrankungen, gab ihnen 1934 der ungarische Arzt Ladislas J. Meduna ein Kampferpräparat und später Cardiazol,[917] um einen Krampfanfall hervorzurufen. In Kenntnis dieser Methode und der Berichte über Erfolge entwickelte Cerletti mit seinen Mitarbeitern an der Neuropsychiatrischen Universitätsklinik in Rom die Elektrokrampftherapie, die er zunächst im Tierversuch und 1938 erstmals an einem schizophrenen Patienten anwandte.[918] Anfangs waren schizophrene Erkrankungen das Indikationsgebiet für die Krampftherapien, schließlich erwies sich die Elektrokrampftherapie (EKT) jedoch als besonders wirkungsvoll bei „depressiven Zuständen“.[919]

In der ‚Ära Hoff‘ wurde die Elektrokrampftherapie deutlich häufiger angewandt als die Malariafieber- und die Insulinkomatherapie. Sie wird jedoch hier nicht so ausführlich behandelt wie diese, da im Projekt und im vorliegenden Text der Schwerpunkt auf die beiden ‚Wiener Therapien‘ gelegt wurde, deren intensive und lange Anwendung in Wien auf die Bedeutung der Wiener Tradition im Konzept des Klinikleiters Hans Hoff zurückgeführt wird.

2.3.1 Die Diskussion um die Elektrokrampfbehandlung in den 1950er Jahren

Die EKT hielt rasch weltweit Einzug in die psychiatrischen Kliniken. Über die genaue Wirksamkeit der Elektrokrampfbehandlung bei verschiedenen Diagnosen bestand aber noch zu Beginn der 1950er Jahre keine einheitliche Meinung. In der zentralen Frage der Diskussion, ob durch eine Elektrokrampfbehandlung schizophrene Psychosen und Psychosen aus dem manisch-depressiven Formenkreis geheilt oder nur ihr Verlauf abgemildert werden

---

917 Zur Geschichte der Cardiazolkrampftherapie vgl. MCCRAE, ‘A violent thunderstorm’: Cardiazol treatment in British mental hospitals (2006), passim.

918 ESCHWEILER/WILD/BARTELS, Elektromagnetische Therapien in der Psychiatrie (2003), S. 3f. Zur Geschichte der EKT Cerlettis und ihrer Einordnung in die „somatischen Behandlungen der 1930er Jahre“, vgl. GAWLICH, Eine Maschine, die wirkt (2018), S. 51-79.

919 SARGANT/SLATER, Die modernen psychiatrischen Behandlungsmethoden (1951), S. 45 und ebenso bereits in der englischen Auflage von 1946, S. 52. Vgl. SHORTER/HEALY, Shock Therapy (2007) zur wechselvollen Geschichte der EKT bis zur Jahrtausendwende: als umfassende Information, wenn es auch den Autoren „weniger um eine Historisierung und Erklärung der Elektrokrampftherapie […], als um eine geschichtliche Unterfütterung ihres [gegenüber der EKT sehr positiven, GH] therapeutischen Standpunktes“ geht – so GAWLICH, Eine Maschine, die wirkt (2018), S. 21. Gawlich gibt (ebd., S. 20-30) eine kritische Beschreibung des Forschungsstands nicht nur zur EKT, sondern auch allgemein zu den körperlichen Therapien in der Psychiatrie.

könnten, dürfte sich die Meinung durchgesetzt haben, dass die EKT nur symptombezogen wirke und so den klinisch manifesten Verlauf – nicht in gleichem Maß den Verlauf der Episode, d.h. die Verlaufsdynamik der Erkrankung insgesamt – verbessere.[920] So schrieb Hans Hoff bereits 1952 in seinem Vorschlag an das Nobelkomitee, Manfred Sakel und Ugo Cerletti den Nobelpreis für Medizin zu verleihen: Die EKT sei „zwar nicht imstande, das manisch-depressive Irresein als Krankheit zu heilen, wohl aber gelingt es dank dieser Therapie, einzelne Phasen wesentlich abzukürzen, mindestens aber das aktuelle Syndrom zu beseitigen."[921]

Das wurde nicht von allen ÄrztInnen so gesehen. Herbert Lewrenz (Hamburg) widersprach 1951 in „Der Nervenarzt" der von mehreren Autoren wie auch im Zitat von Hoff 1952 genannten „wesentlichen" Abkürzung der Erkrankungsphase durch die EKT. Seine Untersuchung an einer statistisch repräsentativen PatientInnenzahl habe gezeigt, dass es durch die EKT zu keiner Verkürzung der Krankheitsphasen im Vergleich zu früheren manischen oder depressiven Episoden kam; auch nach intensiven Schockserien habe sich eine Phasenverkürzung fast nie erreichen und statistisch nicht nachweisen lassen. Lewrenz kritisierte die hohen Erwartungen in die EKT „bei endogen phasischen Psychosen aus dem Formenkreis der manisch-depressiven Erkrankungen", anerkannte jedoch ihre Bedeutung „in der Beeinflussung der Symptomatik einer Psychose, also in der Möglichkeit, bestimmte Zustandsbilder zu wandeln" und damit „unter Umständen die einzig mögliche und sogar lebensrettende Behandlungsmaßnahme zu sein." Er dürfte damit insbesondere die EKT bei der ‚akuten tödlichen Katatonie' gemeint haben.[922]

---

[920] Deshalb wurde die Kombination von EKT und Antidepressiva zum Standard, die Medikamente zur Aufrechterhaltung des Effekts der EKT; das setzt eine Einschätzung der Episodendauer voraus – aus der Erfahrung beim / bei der Patienten/in, sonst interindividuell bei 6 Monaten mit breiter Streuung (Information von Eberhard Gabriel).

[921] Hans Hoff an das Nobelkomitee, 11.10.[1952], Kopie im Josephinum Wien, NL-Hoff MUW-AS-006005-0015 (ausführlicheres Zitat unten S. 302 Anm. 1145). Vgl. auch Hoff an das Nobelkomitee, 19.10.1957, Kopie ebenda, NL-Hoff MUW-AS-006005-0011-068 Zl. 68/5: Es sei der „Verdienst von Cerletti, durch die Einführung der Elektroschockbehandlung das Los von Melancholikern […] verbessert zu haben." Anders spricht Hoff noch im Schreiben von 1957 in Bezug auf die Insulinkomatherapie, von einer heilenden Wirkung (vgl. oben S. **175**). In einer Fragebeantwortung zur Wirksamkeit der Schocktherapie bei Schizophrenien(!) schrieb der Münchner Klinikchef KOLLE, Frage 21: Wie gestalten sich unter der Wirkung der Schocktherapie die Aussichten der Schizophrenen (1954), S. 263: „Elektroschock [und] Kardiazolschock […] eignen sich vor allem zur Bekämpfung akut aufgetretener schizophrener, vor allem katatoner Symptomkomplexe. Die Behandlung ist wohl als reinen Symptomtherapie aufzufassen"; etwas anders bei der Insulintherapie, wo Kolle von „praktische[n] Heilungen" bei frühen Fällen spricht, die zwar „mit und ohne Behandlung vor[kommen], anscheinend etwas häufiger nach der großen Insulinkur."

[922] LEWRENZ, Untersuchungen über den Wert der Schocktherapie bei endogenen Psychosen aus dem Formenkreis der manisch-depressiven Erkrankungen (1951), S. 207-209. Zu Arnolds frühen Arbeiten zur EKT bei der ‚akuten tödlichen Katatonie' vgl. S. 248 und S. 251f.

Bedenken gegen die EKT wurden vor allem wegen der Unfälle im Krampf und wegen des – kontrovers beurteilten – amnestischen Syndroms und seiner psychischen Folgen diskutiert. Die Verfechter der Elektrokrampftherapie, wie etwa der in den USA tätige Psychiater Lothar Kalinowsky[923], waren in der Fachliteratur sehr darum bemüht, die Unbedenklichkeit der Elektrokrampftherapie plausibel zu machen. Zu diesem Zweck wurden Ergebnisse mehrerer Studien, die sich neuropathologischer, elektroencephalographischer oder psychologisch-psychiatrischer Untersuchungsmethoden bedienten, veröffentlicht.[924] Kalinowsky argumentierte, dass die durch Schockbehandlung entstehenden Störungen im Hirn reversibel seien und gab an, mit dieser Position die einhellige Meinung seiner amerikanischen Fachkollegen zu teilen. Denn diverse Untersuchungen hätten ergeben, dass keine dauerhaften Funktionsausfälle entstehen würden, und dass trotz des Gedächtnisverlustes unmittelbar nach der Behandlung kein „Erinnerungsmaterial“ dauerhaft verloren ginge. „Sämtliche intellektuellen Funktionen werden auch bei Patienten, die Hunderte von Elektrokrämpfen durchgemacht haben, in keiner Weise beeinträchtigt.“[925] Manfred Bleuler war anderer Meinung und verwies 1960 auf die Gefahr nicht reversibler „leichter Störungen des Gedächtnisses und des Gedankenganges“ aufgrund des amnestischen Psychosyndroms, weshalb er bei Intellektuellen die Insulinkur empfahl.[926]

---

[923] Lothar B. Kalinowsky flüchtete aus dem nationalsozialistischen Deutschland und hielt sich zunächst in Italien auf, wo er die Elektrokrampftherapie in ihrer Anfangsphase kennen und schätzen lernte. Als er seine Emigration über Frankreich und Großbritannien bis in die USA fortsetzte, sprach er sich an den verschiedenen Stationen seiner Emigration für die neue Technik aus. Sein Bemühen, den Einfluss der „italienischen Schule“ international zu sichern, spiegelt sich auch in seinen Publikationen, die den E-Schock einerseits als ungefährlich und andererseits als dem Insulinschock auch bei der Behandlung der Schizophrenie überlegen erklären. Vgl. SCHMUHL/ROELCKE, Einleitung (2013), S. 25 und RZESNITZEK, Lothar B. Kalinowsky und die Einführung der Elektrokrampftherapie in Europa (2013), S. 200-215.

[924] KALINOWSKY, Das Problem der Hirnschädigung bei Schockbehandlungen (1950), S. 201.

[925] Ebd., S. 202. Kalinowsky sprach sich noch 1952 für die EKT bei der Schizophrenie (allgemein) aus, nachdem die histologisch nachweisbaren „hirnpathologischen Veränderungen“ durch die Insulinkomatherapie nicht, wie viele Kliniker meinten, zum therapeutischen Prozess beitrügen. „Nach unseren eigenen Erfahrungen kann mit intensiver Elektrokrampfbehandlung, die keine histologischen Veränderungen, wohl aber […] elektroencephalographische und psychologisch-psychiatrische hervorruft, der gleiche therapeutische Erfolg erzielt werden.“

[926] BLEULER, Lehrbuch der Psychiatrie ([10]1960), S. 155f. und S. 160. BLEULER, Lehrbuch der Psychiatrie ([11]1969), S. 452, betont ebenfalls die Gefahr, empfiehlt jedoch nicht mehr die Insulintherapie als Alternative. Weniger deutlich wird diese Gefahr in den eingesehenen Publikationen der Wiener Kliniker betont: vgl. HIFT, Die Behandlung der endogenen Depression an der Wiener Klinik (1960), S. 290, empfiehlt, um bei „Patienten, deren ganzer Beruf auf Gedächtnis aufgebaut ist (z.B. Anwälten)“ das amnestische Syndrom zu vermeiden, „einen Tofranilversuch zu machen, auch wenn die anderen Umstände dafür ungünstig sind.“ Dauerhafte Gedächtnisverluste blieben ein wesentlicher Punkt in der Kritik der EKT bis ins 21. Jahrhundert. Vgl. mehrfach dazu in SHORTER/HEALY, Shock Therapy (2007), bes. im 10. Kapitel

Nicht alle Psychiater hielten zu Beginn der 1950er Jahre die Elektrokrampfbehandlung für ebenso unbedenklich wie Kalinowsky. Verzichten wollte allerdings kaum ein Psychiater auf die Methode, obwohl Zwischen- und auch Todesfälle durchaus bekannt gemacht wurden. Die häufigste Komplikation bestand im Auftreten von Wirbelbrüchen, die sich infolge der massiven Überspannung des Muskelgewebes am Beginn des Krampfanfalls (in der tonischen ‚Ansprungsphase‘) ergaben. Viele dieser Brüche waren – anders als die viel seltener auftretenden Röhrenknochenbrüche und Kompressionsfrakturen der Wirbelsäule – klinisch unauffällig oder äußerten sich ‚nur' in Rückenschmerzen, die von der Brustwirbelsäule ausgingen. Die Angaben über die Häufigkeit der Wirbelbrüche variierten stark und korrelierten ganz offensichtlich mit der Einstellung der Autoren zur EKT. So meinte Cerletti, der die Methode entwickelt hatte und propagierte, bei 15.000 Schocks „eine einzige Fraktur, keine Läsion der Wirbelsäule“ gesehen zu haben – was jedoch damit zusammenhängen dürfte, „daß die Wirbelsäulenfraktur relativ spät entdeckt wurde.“ Hingegen wollten amerikanische Autoren aufgrund von Röntgenuntersuchungen vor und nach der Behandlung 50% Wirbelsäulen- bzw. Knochenschäden festgestellt haben.[927] Zu Beginn der 1950er Jahre waren Psychiater deshalb auf der Suche nach Möglichkeiten, die Frakturhäufigkeit bei Elektroschocks zu verringern.[928] Versuche, Frakturen zu vermeiden, indem man die PatientInnen in seitlicher Embryonalstellung lagerte, mittels Hilfspersonal aktiv streckte oder die Gliedmaßen in Rückenlage beugte, brachten nicht die erhofften Erfolge.[929] Mit Muskelrelaxanzien wie dem Curare bestand zwar die Möglichkeit, den Schockfrakturen vorzubeugen, allerdings wurde von diesen nur in Ausnahmefällen Gebrauch gemacht, da die Nebenerscheinungen oft gefährlicher waren als die Muskelkontraktionen selbst.[930]

2.3.2 Die Entwicklung von Methoden zur Verringerung der Risiken an der Wiener Klinik

Eine entscheidende Weiterentwicklung der Elektrokrampfbehandlung, durch die die Frakturhäufigkeit erstmals maßgebend eingedämmt werden konnte, gelang dem Wiener Psychiater Ottokar H. Arnold mit Kollegen, die 1951 publiziert wurde. In einer Untersuchungsreihe an der Wiener Universitätsklinik für Neurologie und Psychiatrie testeten

---

[927] HOFFMANN, Zur Frage der ambulanten Schockbehandlung (1950), S. 411.
[928] Vgl. WEINLAND, Zur Frage der Frakturhäufigkeit bei der Elektroschockbehandlung (1951), S. 298-302.
[929] HOFFMANN, Zur Frage der ambulanten Schockbehandlung (1950), S. 411.
[930] HIFT/HOFMANN, Der vollmitigierte Elektroschock (1954), S. 455. Zu den Weiterentwicklungen in der Technik der Anwendungen (elektrotechnische Weiterentwicklung, Einführung des Narkoseschutzes und der Muskelrelaxantien) und den Diskussionen dazu, vgl. Hugo SOLMS, Die Krampfbehandlung (1963), S. 439-462.

sie die muskellähmende Wirkung des Bernsteinsäureesters M115 (Lysthenon)[931] während der Elektrokrampfbehandlung. Es gelang ihnen, eine Anwendungsformel auszuarbeiten, deren genaue Einhaltung eine stark herabgesetzte Kontraktion des Muskelgewebes für die kurze Zeit des durch den Elektroschock ausgelösten Krampfanfalls gewährleistete. Da Bedacht darauf gelegt wurde, Lysthenon in Dosen einzusetzen, wodurch „am Ende des Schocks […] auch die Lähmung durch M 115 praktisch vorüber" und die Atmung „spontan und meist in normalem Umfang ein[setzt], nur in einzelnen Fällen waren die ersten, 2 bis 5 Atemzüge etwas eingeschränkt. Künstliche Beatmung und Sauerstoffinsufflation waren niemals erforderlich."[932]

Neben den Knochenbrüchen wurden um 1950 ernsthafte Komplikationen unter anderem bei bestehenden Herzschädigungen beobachtet. Vorsicht war laut Mitteilung zweier Psychiater aus Göttingen bei PatientInnen mit Coronarinsuffizenzkurven oder bei Vorliegen einer Mitralstenose angezeigt. Obwohl die Autoren auch einen Fall aus ihrer Klinik referierten, in dem es durch den Elektroschock zu Vorhofflimmern mit letalem Ausgang kam, fassten sie ihre Befunde über Elektrokrampfbehandlungen und kardiologische Erkrankungen wie folgt zusammen: „Überblickt man die Befunde, so ergibt sich, daß von bestimmten Fällen abgesehen der schädigende Einfluß des Elektrokrampfes auf das kranke Herz ein erstaunlich geringer ist angesichts seines gewaltsamen motorischen Ablaufs und der damit verbundenen nicht unerheblichen akuten Kreislaufbeanspruchung."[933]

Die Anwendung der Neuroleptika ab 1952 und schließlich auch der Antidepressiva ab 1958 führten allgemein bereits in den 1950er Jahren zu einer Einschränkung der EKT. Gegen Ende der 1950er Jahre erwarteten mehrere PsychiaterInnen einen baldigen Ersatz der EKT durch die Psychopharmaka. Der dänische Psychiater Villars Lunn etwa meinte in seiner Funktion als Präsident des XII. Nordischen Psychiater-Kongresses in Kopenhagen 1958, dass der Elektroschock in absehbarer Zeit entbehrlich sein würde; und sein Kollege Jörn Ravn führte in einem Tagungsbeitrag von 1960 aus, dass das jüngst auf den Markt gekommene Neuroleptikum Truxal (Chlorprothixen), das nach Testungen von Ravn u.a. „sowohl

---

[931] ARNOLD/BÖCK-GREISSAU/GINZEL, Über die Einführung eines neuen muskellähmenden Mittels in die Elektrotherapie (1951), S. 492.
[932] Ebd.
[933] HADDENBROCK/KEMMANN, Über die Wirkung der Elektrokrampfbehandlung auf das pathologische Elektrokardiogramm (1950), S. 445. Vgl. SCHULTE/DREYER, Eine cerebrale tödliche Komplikation nach Elektroschock (1950), S. 178: Psychosen stellten aus medizinischer Sicht eine so schwerwiegende pathologische Erscheinung dar, dass selbst eine mitunter risikoreiche Behandlung der Nicht-Behandlung vorzuziehen sei.

sedierend als auch ausgeprägt antidepressiv wirkt", mit anderen neuen Psychopharmaka dazu beitragen könnte, den Elektroschock sowohl bei schizophrenen als auch bei depressiven Erkrankungen zu ersetzen.[934]

In Wien wurden einige Syndrome schizophrener und manisch-depressiver Erkrankungen nun mit den neuen Psychopharmaka, andere mit der EKT, mehrere auch mit einer Kombination der beiden Therapien behandelt.[935] Aufgrund der Verbesserung der Methode und Reduzierung der Gefahren – wozu die Wiener Kliniker beigetragen hatten –, als auch durch die Spezifizierung der Indikation war man an der Wiener Klinik überzeugt, mit der EKT über eine risikoarme und in einigen Indikationen höchst wirksame Methode zu verfügen. Nicht nur gab es durch Lysthenon keine Knochenbrüche und -läsionen mehr und Abnormitäten im Knochenbau waren nun kein Hindernis mehr für die Indikation, die Wiener Kliniker entwickelten auch eine Methode, um die verbleibenden Kontraindikationen zu minimieren. Schwerwiegende Herzprobleme galten als Grund, auf Elektroschocks zu verzichten, und so konnten PatientInnen, die gleichzeitig unter schweren Myokardschäden und massiven Depressionen litten, nicht behandelt werden. Nach Ansicht der Wiener ÄrztInnen war es aber unabdingbar, Psychosen auch bei kontraindizierten PatientInnen zu therapieren, wenn durch dauernde Unruhe, Schlaflosigkeit und Nahrungsverweigerung bereits Lebensgefahr bestand. Die Lösung, die an der ‚Klinik Hoff' erdacht und im Sommer und Herbst 1953 an 27 PatientInnen getestet wurde, bildete eine Kurznarkose und eine starke Erhöhung der Dosis Lysthenon, auch wenn dadurch eine künstliche Beatmung erforderlich wurde. Dazu wurde zunächst das schnellwirkende Barbiturat (Hypnotikum) Pentothal und danach eine hohe Dosis Lysthenon verabreicht und so eine vollständige Muskel- und zuletzt auch Atemlähmung bewirkt.[936] Die PatientInnen wurden während der Krampfbehandlung und „bis nach etwa 3-5 Minuten [nach dem ES] die spontanen Atembewegungen einsetzten" künstlich beatmet. Bei 129 Anwendungen bei 27 PatientInnen kam es zu keinem Zwischenfall.[937]

---

[934] RAVN, Truxal, ein neuartiges Psychopharmacon (1960), S. 196.
[935] Vgl. unten S. 252f. die Ausführungen von Stefan Hift und Hans Hoff 1958 zu den Indikationen.
[936] Bei der höheren Dosis Lysthenon führte die Lähmung auch der Atemmuskulatur zum Atemstillstand (der durch Beatmung überbrückt werden mußte), während es bei der teilmitigierten Anwendung durch die niedrige Dosis von Lysthenon nur zu einer Behinderung der Atembewegung kommt, deren Erlebnis durch die Setzung der EKT und die dadurch eintretende Bewusstlosigkeit im Anfall gleichsam annulliert wird, ebenso wie das Erlebnis des Atemstillstandes im Krampfanfall (Information von Eberhard Gabriel).
[937] HIFT/HOFMANN, Der vollmitigierte Elektroschock (1954), S. 455.

Zur Gleichwertigkeit dieser „vollmitigierten“ Anwendung (Lysthenon und Narkose) mit der Anwendung mit dem Muskelrelaxans, aber ohne Narkose scheint es in Wien jedoch vorerst unterschiedliche Meinungen gegeben zu haben: Hift und Hofmann resümierten in ihrer Studie, dass sich „der vollmitigierte Schock“ in ihrer Untersuchung an einem freilich sehr kleinen Sample von zehn sorgfältig ausgewählten älteren PatientInnen mit schweren endogenen Depressionen dem ‚E-Schock‘ ohne Narkose als gleichwertig erwiesen habe.[938] Ihr Klinikkollege Arnold scheint bereits im gleichen Jahr 1954[939] anderer Meinung gewesen zu sein. So schrieb er zur Forderung angelsächsischer Autoren, die unter anderem „aus tiefenpsychologischen Gründen (ES = Todeserlebnis)“[940] bei der EKT allgemein eine Narkose befürworteten, Blutbefunde würden dafür sprechen, dass durch die Barbiturate die Wirksamkeit des ‚E-Schocks‘ abgeschwächt würde.[941] Auch aus einem psychiatrischen Zwischenbefund von Arnold (1957) während einer langen Behandlung der 33jährigen Privatpatientin A13416 mit der Diagnose „Depression u. Neurose“ (Behandlungsbogen: „endogene Depression“) geht hervor, dass er die EKT in Narkose therapeutisch für weniger wirksam hielt: „Von der weiteren Fortsetzung der E-S in voller Narkose verspreche ich mir nichts, zumal sie jeweils 1g Narkotion benötigt und trotzdem kaum ein amnestisches Syndrom nachher bietet. Daher wäre später, aber nur wenn sich sichere Symptome zur Wiederherstellung der depressiven Phase zeigen, der Versuch zu machen einige E-S in rascher Folge ohne Narkotion zu geben [mit Tinte unterstrichen].“[942] Eine Kurznarkose bei der EKT dürft jedoch bei PrivatpatientInnen bereits in diesen Jahren auch ohne Kontraindikation gegeben worden sein, erst in den letzten Jahren der Ära Hoff wurde sie aufgrund einer ‚Gleichbehandlungsinitiative‘ der AssistentInnen (nicht aus einer therapeutischen Bevorzugung) bei allen Anwendungen eingeführt.[943]

---

938 Ebd., S. 458.
939 ARNOLD, Schockbehandlungen (1954), S. 55.
940 Die Atemlähmung wurde auch ohne Kurznarkose nicht als solche erinnert, manchmal jedoch als etwas Unbestimmtes, Ängstigendes. Bernd Küfferle – er war ab 1965 an der Klinik – beschreibt im Gespräch mit dem Autor, dass diese unangenehme Erinnerung durch eine Aufklärung des / der Patienten/in über den kurzen Atemstillstand zu verhindern war.
941 Vgl. Hoff, Lehrbuch der Psychiatrie (1956), S. 498: eine Pentothal-Narkose würde sich ungünstig auf das Ergebnis der Schockbehandlung auswirken „und bei entsprechender Organisation absolut entbehrlich sein.“
942 In der Spitalsrechnung der Patientin wird deutlich, dass sie 2 nicht in voller Narkose erhielt: zu den „22 E-Schocks“ wurden nur „19 Narkosen f. Schock“ verrechnet. Zur Patientin und zur Therapie hieß es vor der zitierten Stelle: „Bei der jetzigen Kontrolle (einen Tag nach der letzten E-S) ist Patientin ausgeglichener Stimmung, im Gehaben infantil, regrediert, bietet keine körperlichen Aspekte einer noch bestehenden Melancholie, dagegen in der Ausdrucksmotorik ein organisches Psychosyndrom. Sie steht allerdings unter einer erheblichen Dosis von Largactil und Miltaun. Vielleicht wäre jetzt nach Entfernung des Bruders und Gatten der Zeitpunkt gekommen, den Versuch zu machen, sie an einen Psychotherapeuten zu fixieren. Dies könnte trotz der sprachlichen Schwierigkeit lohnenswert sein.“
943 Information von Bernd Küfferle und Eberhard Gabriel.

Einen Bericht über die Erfahrungen mit der ‚Elektroschockbehandlung' unter Kurznarkose und mit Muskelrelaxans lieferte F. Jost 1957 aus der Psychiatrisch-Neurologischen Klinik der Universität Innsbruck.[944] Durch die Verabreichung des Lysthenons in Kurznarkose (mit Thiopental oder Narkothion) habe sich die „therapeutische Breite" der EKT vergrößert. In einer Auflistung der 19 PatientInnen „höheren Alters", die mit dieser Methode im Jahr 1956 an der Innsbrucker Klinik behandelt wurden, waren die Indikationen: „In 5 Fällen lagen Osteoporose des Skeletes und schwere Spondylopathien der Wirbelsäule vor, in 4 Fällen Adipositas, in 3 Fällen Zustände nach Frakturen, in 1 Fall Hypertonie, in 1 Fall Morbus Bechterew, in 5 Fällen intensive Angst vor der Schockbehandlung." [945] Die Anzahl der Anwendungen schwankte bei diesen PatientInnen zwischen 4 und 18.

1958 waren Hift und Hoff der Meinung, die Methode sei „bei richtiger Technik die derzeit ungefährlichste Therapie, die wir in der Psychiatrie haben." Durch pharmazeutische (Lysthenon) und technische Fortschritte, die im Lauf der 1950er Jahre in die Behandlung eingebracht worden waren, gäbe es kaum noch Kontraindikationen und bei den rund 15.000 ‚normalen' und 2.000 ‚vollmitigierten E-Schocks'[946], die von 1953 – 1958 an der Wiener Klinik angewandt worden waren, habe es „keinerlei Zwischenfälle" gegeben.[947] Als Neuerungen, die neben der Verwendung von Muskelrelaxanzien die EKT um 1960 zu einer weitgehend sicheren Behandlungsmethode gemacht hätten, nannte Arnold auch die Einführung des Siemens-Konvulsators III, durch den die so genannte Ansprungszuckung deutlich vermindert würde, wodurch die Gefahr der Wirbelfrakturen „weitgehend" herabgesetzt worden sei.[948]

---

[944] JOST, Zur Verwendung der Allgemeinnarkose in der Psychiatrie. Mit besonderer Berücksichtigung der Elektroschockbehandlung unter Mitigierung durch Lysthenon (1957), S. 337-339.
[945] JOST, Zur Verwendung der Allgemeinnarkose in der Psychiatrie. Mit besonderer Berücksichtigung der Elektroschockbehandlung unter Mitigierung durch Lysthenon (1957), S. 338. Zur Angst der PatientInnen vor dem Elektroschock und deren Ursachen: PEMSL, Über die „Schockangst" (1957), passim; CERMAK/RINGEL, Zur Frage der Angst vor dem Elektroschock (1959), passim. Cermak und Ringel thematisieren auch die Angst vor der Psychiatrie, die häufig die notwendige Frühbehandlung verhindere.
[946] Den Krankenakten zufolge haben fast ausschließlich ältere und PatientInnen der Privatstation ‚vollmitigierte E-Schocks' (d.h. mit Muskelrelaxans und Kurznarkose) bekommen. Bei ihnen ist im Stempel „E-Schock" Lystenon bzw. außen auf der Akte oft „Narko" angegeben.
[947] HIFT/HOFF, Die organische Therapie der Psychose (1958), S. 1047.
[948] ARNOLD, Die körperlichen Behandlungsmethoden der Schizophrenie (1960), S. 274. Vgl. ausführlich zur Entwicklung der Konvulsatoren GAWLICH, Eine Maschine, die wirkt (2018), S. 153-226 (Kapitel 4, Morphologie(n) des Apparats) und GAWLICH, Buttons and Stimuli: The Material Basis of Electroconvulsive Therapy as a Place of Historical Change (2020), S. 202-222. Für die Wiener Klinik konstruierte Anfang der 1940er Jahre der Kliniker und studierten Elektrotechniker Wolfgang Holzer (vgl. oben S. 207 Anm. 806) den Apparat.

Als einzige „absolute" Kontraindikation gegen die EKT definierte Arnold 1960 die exzessive Blutungsneigung des zerebralen Gefäßsystems, wie sie oft durch Alkoholmissbrauch entsteht, sowie den akuten Myokardinfarkt. Alle anderen früher angenommenen Kontraindikationen würden durch die Möglichkeit entfallen, in diesen Fällen die ‚vollmitigierten E-Schocks' anwenden zu können. Bezüglich der Zwischenfälle gab Arnold 1960 – in Widerspruch zu „keinerlei Zwischenfall" im oben zitierten Artikel von Hift und Hoff 1958 – die sehr geringe Zahl von 0,17% („sechs Fälle von Wirbelfrakturen und eine Fraktur beider Oberarme") für die in den letzten vier Jahren ca. 3.500 PatientInnen an der Wiener Klinik an.[949]

Die Zahl und die Abfolge der Anwendungen waren unterschiedlich: In den 1950er Jahren war eine Serie von 6 Anwendungen, eine alle zwei Tage, in Wien üblich,[950] und manchmal wurde sie nach einigen Tagen wiederholt. Bei der endogenen Depression, bei der die EKT Standard war, verringerte sich die Zahl der Anwendungen nach der Einführung der neuen Antidepressiva. So lehrte Hoff Anfang der 1960er Jahre lt. nichtautorisiertem Vorlesungsskriptum, dass bei der Melancholie, bei der der „E-Schock [die] Therapie der Wahl!" sei, „früher 6 – 8 Schocks" gegeben wurden, womit „man die Leute aus der Phase heraus[bekam]. Heute gibt man 2 – 3 Schocks [so viele wie notwendig sind, „um die Phase zu unterbrechen"], dann Thymoleptika". Der „E-Schock" wirke zur „Aufhebung des Beharrungsphänomens", zur „Auflockerung des Vegetativums", wonach „die antidepressiven Drogen besser wirken" würden.[951] Zur Beruhigung stark erregter PatientInnen erfolgten eine, aber auch mehrere Anwendungen an einem Tag; zur Behandlung der „akuten bedrohlichen Katatonie" zwei „Elektroschockblocks" mit je „drei Elektroschocks im Abstand von je 15 Minuten" am ersten Tag, 2 „Schocks" am zweiten und 1 – 2 am dritten Tag; je nach Verlauf sollten weitere Anwendungen folgen.[952] Häufig wurden ‚E-Schocks' während der Insulinkomatherapie im Koma ‚aufgesetzten'.[953]

---

[949] ARNOLD, Die körperlichen Behandlungsmethoden der Schizophrenie (1960), S. 276.
[950] Vgl. im Abschnitt zur IKT auf S. 219 das Fallbeispiel 1954 der 13jährigen Patientin S902 mit der Diagnose „Schizophrene Reaktion bei antriebsgestörter postencephalit[ischer] Persönl[ichkeit]" („Grund der Aufnahme: Mutismus, Depression, Geräusche-Hören, SM-Gefahr, Fluchttendenzen").
[951] Hoff-Skriptum, Allgemeine Psychiatrie [um 1961], S. 102. 85% würden auf die EKT ansprechen, 15% würden (nur) „medikamentös behandelt."
[952] ARNOLD, Die Therapie der Schizophrenie (1963), S. 171f. Vgl. dazu die Anwendung nach Arnold 1953, zitiert unten S. 251f.
[953] Ähnlich vgl. MICHAUX, Psychiatrie (1965), S. 1003: « Une ‚série' d'électrochocs comporte en général 6 à 8 chocs à raison d'un par jour dans les cas aigus, ou un tous les deux jour habituellement. Les 'chocs en sommation' (4 chocs le premier jour, trois le second, puis un ou deux les jours suivants selon besoin) et les techniques dites d''anéantissement' (8 à 12 chocs par jour, jusqu'à obtention d'un grand état confusionnel) ont

2.3.3 Erklärung des Wirkmechanismus in den Publikationen der Zeit

Zur Wirkungsweise der Elektrokrampftherapie wie auch der Insulinkomatherapie meinte Arnold 1954, dass es sich um „mehr oder weniger unspezifische Eingriffe in den aktuellen Gesamtzustand des Kranken“ handle. Der somatische Effekt sei „schon sehr genau“ erfasst und bestünde in Veränderungen der neuro-vegetativ-hormonalen Regulationsabläufe. Die Wirkungen im seelischen Bereich seien allerdings noch weitestgehend unbekannt. Insgesamt bestünde laut Arnold die Wirkung der ‚Schockbehandlungen‘ in der „Tendenz zur Wiedererlangung gestörter Gleichgewichte und damit Wiedererlangung der Fähigkeit, sich gegebenen Situationen anzupassen“.[954] In seine theoretischen Erwägungen über die Wirkmechanismen der EKT sah Arnold bereits in seiner wegweisenden Studie zur akuten tödlichen Katatonien auch Bezüge zum vegetativen Nervensystem,[955] welches damals in Wien ein aktuelles Thema der Forschung im Schnittpunkt von Physiologie, Innerer Medizin und Neurologie-Psychiatrie war.[956] Nach Arnold (1960) hatte „die ES-Wirkung“ bei der akuten bedrohlichen Katatonie, bei der „die schizophrene Grundstörung Anlaß zum Aufbau besonderer Reaktionslagen[957] wird, die mit entsprechender vegetativ-humoraler Symptomatik verbunden sind,“ einen „teilkausale[n] Effekt“, während sie „in den meisten Indikationsgruppen [der Schizophrenie, GH] nur als symptomatischer Eingriff gewertet werden“ könne.[958]

Klarheit darüber, warum sich psychiatrische Krankheitsbilder nach einer Elektrokrampftherapie besserten, gab es nicht – trotz vieler Studien, die im folgenden Jahrzehnt erschienen. Dazu schrieb Stefan Hift 1954: „Die Wirkungsweise des ES ist

été abandonnés depuis l’association possible aux chimiothérapies antidépressives ou neuroleptiques (réserpine exceptée). » Zu den Anfängen der „Summation“ der Therapien im Insulinkoma vor 1950 vgl. GAWLICH, Eine Maschine, die wirkt (2018), S. 271f.

[954] ARNOLD, Schockbehandlungen (1954), Teil II, S. 53.

[955] Vgl. dazu ARNOLD, Untersuchungen zur Frage der akuten tödlichen Katatonien (1949), S. 394.

[956] Vgl. KAUDERS, Vegetatives Nervensystem und Seele (1946), die kleine selbständige Publikation nach einem Vortrag, den – lt. GABRIEL, Otto Kauders (2018), S. 218 – Kauders am 23. 7. 1945 an der Wiener Klinik hielt; PÖTZL, Leukotomie und Vegetativum (1950), S. 317-341 (nach einem Vortrag in der *Gesellschaft zur Erforschung des vegetativen Systems* 1950); BIRKMAYER/WINKLER, Klinik und Therapie der vegetativen Funktionsstörungen (1951). Richtunggebend für die Beschäftigung der Wiener Neurologen mit diesem Themenbereich dürft Otto Pötzl gewesen sein.

[957] Vgl. https://de.wikipedia.org/wiki/Katatonie#Symptombild (2.4.2023): Katatonie „geht mit Störungen der Motorik einher, die zwischen extremer Erregung und Passivität hin- und herwechseln können“ – zwischen „zielloser, ungerichteter Hypermotorik und gesteigerte Impulsivität“ und „unnatürlichen und stark verkrampften Haltungen bzw. Verhaltensweisen des ganzen Körpers bzw. der Person“ (wie Stupor).

[958] ARNOLD, Die körperlichen Behandlungsmethoden der Schizophrenie (1960), S. 274.

eigentlich bis heute im wesentlichen ungeklärt. Die große Anzahl von Erklärungsversuchen und -theorien kann kaum darüber hinwegtäuschen, daß die gesicherten experimentellen Ergebnisse spärlich sind."[959]

Vier Jahre später deutete Hift in einem Artikel, den er gemeinsam mit Hans Hoff verfasst hatte, die Elektroschockwirkung bei affektiven Störungen als eine Art „reset"-Funktion, die durch den Krampfanfall aktiviert würde. „Die Wirkung des Elektroschocks liegt in einem momentanen Zerschlagen der [krankhaft lang dauernden] Reaktionslage des Organismus.[960] Im organischen Bereich ist diese schlagartige Änderung am deutlichsten am vegetativen Nervensystem zu beobachten, wo eine gewaltige ‚Stress'-Reaktion mit langsamem Zurückpendeln in die Ausgangslage zu beobachten ist. Psychisch werden die aktuellen Erlebnisse durch die plötzliche Bewußtlosigkeit unterbrochen und durch die nachfolgende Amnesie abgeschwächt."[961]

Durch die Auslösung des künstlichen epileptischen Anfalls sollten demnach funktionelle cerebrale Fehlleistungen kurzfristig ausgeschaltet und anschließend dazu gezwungen werden, sich neu, das heißt in einer „normalen" Ordnung zu formieren. Zum „schrittweise und sukzessive erfolgenden Wiederaufbau der Leistung der Einzelzellen" und „zur Neubildung von Funktionsgemeinschaften" meinte Arnold 1960, dass „ontogenetisch altes Funktionszusammenspiel sich entsprechend frühzeitig wiederherstellt, während jüngste Funktionszusammenhänge gar nicht oder nur langsam wieder eingespielt werden und schließlich jene Funktionszusammenhänge, in der wir die Basis pathologischer Hirnleistung sehen müssen, relativ am stärksten an der Wiedereinspielung behindert werden."[962]

Noch 1967 bezog sich Walter Spiel auf Pötzels Mauserungstherapie.[963] Die Wiener Psychiater hatten zwar eine konsistente Hypothese, einer „klaren kausalen wissenschaftlichen Erklärung" – so der französischen Psychiater Pierre Pichot in der Wiener Medizinische Wochenschrift

---

[959] HIFT/HOFMANN, Der vollmitigierte Elektroschock (1954), S. 458.
[960] Die „organische Störung" die hier bei endogenen Depressionen, auch bei exogen ausgelösten Verstimmungen und ähnlich bei der Manie ‚angenommen' wird, wird folgendermaßen beschrieben: „Der Organismus gerät in eine Reaktionslage, die an sich noch nicht abnormal ist, die aber in abnormer Weise fixiert wird. […]. Das Krankhafte wird erst durch das lange Beharren in der gleichen Reaktionslage bedingt, das sich besonders klar bei den vegetativen Funktionen nachweisen läßt und das sicherlich hereditär und konstitutionell präformiert ist."
[961] HIFT/HOFF, Die organische Therapie der Psychose (1958), S. 1045. Anders wird hier die ‚Störung' bei der Schizophrenie gesehen: vgl. dazu das Zitat oben S. 189f.
[962] ARNOLD, Die körperlichen Behandlungsmethoden der Schizophrenie (1960), S. 273.
[963] SPIEL, Die Therapie in der Kinder- und Jugendpsychiatrie (1967), S. 130f.

1960 – bliebe der Wirkmechanismus der Elektrokrampftherapie ebenso wie die Wirkmechanismen der ‚Schockbehandlungen' und der meisten Psychopharmaka allgemein aufgrund der „Unkenntnis der genauen Pathogenese psychischer Erkrankungen" jedoch entzogen.[964]

2.3.4 Einsatzgebiete der Elektrokrampfbehandlung 1951-1969 nach den Publikationen der Zeit

Anfang der 1950er Jahre bestanden unterschiedliche Auffassungen, welche der ‚Erschütterungstherapien' (‚Schockbehandlungen') – Elektro-, Insulin- oder Cardiazol-‚Schock'[965] – vorzuziehen sei. Dass bei der Schizophrenie und bei einer schweren endogenen Depression eine dieser Behandlungen in jedem Fall angezeigt war, dürfte weitgehend unbestritten gewesen sein.

1949, zehn Jahre nach der Einführung der EKT und im Stichjahr der Züricher Studie, in dem im ‚Burghölzli' ein Höchststand der Anwendungen der Elektrokrampftherapie, der Insulinkur und der Schlafkur nach Klaesi zu verzeichnen war,[966] vertrat der Berner Psychiater Max Müller, der bereits als wichtig für die Durchsetzung der Insulinkur genannt wurde, folgende Position in der Behandlung von PatientInnen mit schizophrenen bzw. affektiven Störungen mit körperlichen Therapien: „Jede beginnende Schizophrenie sei ohne Rücksicht auf Erscheinungsbild und auf die übrigen die Prognose beeinflussenden Faktoren körperlich zu behandeln. […] Für die Wahl der Behandlungsmethode (Insulin- oder Krampfbehandlung) erweist sich dagegen das Zustandsbild als von größter Bedeutung": „Insulinbehandlung für die paranoiden Formen[967] […] Krampfbehandlung für die negativistischen, stuporösen, stark gesperrten Katatonien, […] ferner […] bei manisch-depressiv gefärbten Zustandsbildern [der

---

[964] PICHOT, Vergleich der verschiedenen Behandlungsmethoden in der Psychiatrie (1960), S. 735. Vgl. Hugo SOLMS, Die Krampfbehandlung (1963), S. 470-476, zur noch offenen Diskussion über die Wirkungsmechanismen der Krampfbehandlungen.
[965] Die Behandlung mit der Malariafiebertherapie als ‚Schock-' bzw. ‚Erschütterungstherapie' in der Psychiatrie bei Erkrankungen außerhalb der progressiven Paralyse wird in diesen Jahren in der eingesehenen Literatur nur vereinzelt genannt**:** vgl. die Zitate aus BLEULER, Lehrbuch der Psychiatrie ([10]1960), S. 403 und S. 405 oben auf 31 Anm. 93, sowie aus ARNOLD, Die Therapie der Schizophrenie (1963) S. 59-61 zur Fiebertherapie bei Schizophrenie, vor allem bei der Pfropfhebephrenie/-schizophrenie, zitiert oben S. 83. Zur häufigen Anwendung in Wien vgl. das Kapitel zur Malariafiebertherapie.
[966] TANNER/MEIER/HÜRLIMANN/BERNET, Zwangsmassnahmen in der Züricher Psychiatrie (2002), S. 93; vgl. oben S. 32.
[967] Vgl. oben S. 241 Anm. 925 zu Kalinowsky, der noch 1952 bei Schizophrenie (allgemein) die EKT empfahl und nicht die Insulinkomatherapie.

schizophrenen Erkrankung, GH] und bei Spätschizophrenien".[968] Bei Müller zeigt sich bereits 1949 die zunehmende Einschätzung der Insulinkur als Therapie bei schizophrenen Erkrankungen in frühen Phasen und der EKT bei affektiven Störungen, bei denen er mit der Insulinkur „im ganzen wenig befriedigend[e]" Erfolge sah. Vom „Cardiazolschock" riet Müller wegen der vorangehenden „quälenden Sensationen besonders bei depressiven und ängstlichen Patienten" ab.[969]

Müller schätzte die EKT 1949 noch als gefährlicher ein als die Insulinkomatherapie.[970] Das änderte sich freilich in diesen Jahren: in Wien durch die Einführung des Muskelrelaxans Lysthenon, das die Unfallsgefahr im Krampf reduzierte, und durch die ‚vollmitigierte' Anwendung etwa bei schwerwiegenden Herzproblemen. Wohl auch durch diese Neuerung konnten in bestimmten Fällen nun ‚E-Schockblocks' angewandt werden. Ein Krankheitsbild aus dem schizophrenen Formenkreis, das nach dem Wiener Kliniker Arnold auf jeden Fall sofort einer EKT zu unterziehen war, war die so genannte „akute tödliche Katatonie", die in Wien im Untersuchungszeitraum auch als „akute bedrohliche Katatonie" bezeichnet wurde.[971] Dazu hatte er bereits 1949 und in der Folge mehrmals das Krankheitsbild beschrieben und die in der Folge auch anderswo gebräuchliche Behandlung mit einem ‚E-Schockblock' entwickelt.[972] Er teilte den Verlauf der akuten tödlichen Katatonie in Phasen ein, die zusammengezählt eine durchschnittliche Krankheitsdauer von 17 Tagen ergaben: nach einem etwa 12-tägigen Vorstadium mit unspezifischen Symptomen folgte nach Arnold der vier bis fünf Tage dauernde Krankheitsbeginn mit Hyperkinese, danach das sieben bis neun Tage anhaltende hyperkinetische Stadium und schließlich das etwa vier Tage dauernde, unbehandelt tödlich endende Stadium, in dem die PatientInnen nicht mehr reagierten,

---

[968] Hier sind nicht jene PatientInnen gemeint, deren erste Erkrankung länger als 1 ½ Jahre zurücklag und denen deshalb keine Insulinkomatherapie mehr gegeben wurde (vgl. im Insulin-Kapitel). Zu ihnen vgl. HOFF, Lehrbuch der Psychiatrie (1956), S. 517f.: „Bei alten Schizophrenien, die zum ersten Mal in Anstaltsbehandlung kommen, wird man sich begnügen, die jeweiligen Exazerbationen durch eine symptomatische ES-Behandlung wieder auf das Defektniveau zu stabilisieren. Ansonsten können arbeitstherapeutische und psychohygienische Maßnahmen die Anpassungsfähigkeit des Patienten erhöhen. Man trachtet Träger von Gleichgewichtszuständen, die nur wenig auffällig sind, solange als nur möglich in ihrem gewohnten Milieu zu halten, […]."

[969] MÜLLER, Prognose und Therapie der Geisteskrankheiten (1949), S. 123-125. Zu diesem Argument, das in der ‚Ära Hoff' zur fast ausschließlichen Anwendung des Cardiazolkrampfes im Insulinkoma führte, vgl. oben S. 39 Anm. 118 und die Zitate von Hoff und aus SARGANT/SLATER (1972) oben S. 216 Anm. 840 und 841.

[970] MÜLLER, Prognose und Therapie der Geisteskrankheiten (1949), S.96, S. 125 und S. 62.

[971] ARNOLD/STEPAN, Untersuchungen zur Frage der akuten tödlichen Katatonie (1952), S. 256. Vgl. auch die Ausführungen zur ES-Behandlung in HOFF, Lehrbuch der Psychiatrie (1956), S. 514-516 bei „akute[r] bedrohliche[r] Katatonie", zitiert oben S. 199.

[972] Vgl. ARNOLD, Untersuchungen zur Frage der akuten tödlichen Katatonien (1949), passim; vgl. ARNOLD, Zur Indikation des Elektroschocks (1951), S. 108: „Delirium acutum […] nach Cholecystektomie". Vgl. u. a. ARNOLD/STEPAN, Untersuchungen zur Frage der akuten tödlichen Katatonie (1952), passim.

austrockneten und starr wurden. Arnold hat in seinen diversen Studien über das Syndrom herausgearbeitet, dass der tödliche Ausgang der Krankheit abgewendet werden konnte, wenn spätestens während des vorletzten, des hyperkinetischen Stadiums, eine Elektrokrampftherapie mit einem „E-Schockblock" eingeleitet würde.[973] Nach Hoffs Lehrbuch von 1956 war ein ‚E-Schockblock' auch bei manischen Exazerbationen einzusetzen.[974] 1960 nannte Arnold als eine Verstärkung des ‚E-Schocks' die Elektrocardiazol-Kombination, durch die ein epileptischer Anfall auch bei PatientInnen sichergestellt werden könne, die aufgrund ihres ernsten Zustandes nicht ausreichend auf den ‚Elektroschock' reagierten.[975]

Eine deutliche Änderung in der Indikation ergaben sich mit der Entwicklung der neuen Psychopharmaka, die an der Wiener Klinik ab 1952 eingesetzt wurden.[976] Bereits 1958 haben Stefan Hift und Hans Hoff in einem Artikel Therapievorschläge gemacht, die die Insulinkomatherapie und die EKT ebenso wie die neuen Psychopharmaka, in den ‚Gesamtbehandlungsplan' integrierten. Zu den verschiedenen Formen schizophrener und affektiver Erkrankungen werden Anwendungen vorgeschlagen, wobei nicht nur die beiden alten großen Kuren alleine und kombiniert genannt werden, sondern auch Behandlungen alleine mit den neuen Neuroleptika und Tranquilizern.

Zusammenfassend hieß es schließlich, dass „aus den angeführten Indikationen" ersichtlich sei, dass „wir die Schockbehandlung nicht für überholt ansehen.[977] Wir sind im Gegenteil der Meinung, daß ihre Leistungsfähigkeit durch genauere Indikationsstellung und verbesserte Technik in den letzten Jahren bedeutend zugenommen hat. Die Neuroleptika und die Tranquilizer erscheinen als sehr wertvolle Bereicherung unserer Therapie, ohne den Anspruch

---

[973] ARNOLD, Behandlungsergebnisse bei der akuten tödlichen Katatonie (1953), S. 93f. Zur (gleichbleibenden) Methode des Schockblocks vgl. oben S. 247 das Zitat aus ARNOLD, Therapie der Schizophrenie (1963), S. 171f.

[974] HOFF, Lehrbuch der Psychiatrie (1956), S. 513: „Akute manische Phasen im Rahmen des manisch-depressiven Krankheitsgeschehens […] an einem Tag, in Abständen von etwa einer Stunde, 5 ES nacheinander […]. Diese ES-Serie bewirkt immer eine wesentliche Beruhigung".

[975] ARNOLD, Die körperlichen Behandlungsmethoden der Schizophrenie (1960), S. 274f. Vgl. ARNOLD, Die Therapie der Schizophrenie (1963), S. 45. Vgl. zu dieser Anwendung auch bereits 1956 in HOFF, Lehrbuch der Psychiatrie (1956), S. 515, zitiert oben S. 215f. Anm. 838.

[976] Vgl. Hans Hoffs Vortrag bei der Tagung in Paris im Oktober 1955 zu den Neuroleptika: HOF, Advantages and Disadvantages of Treatment with Chlorpromazine and Serpasil (1956), S. 52-58.

[977] Entschiedener formulierte 1959 der Psychiater an der Landes-Heil und Plegeanstalt Hall i.T. H. Pemsel (Über die „Schockangst", S. 748): „Hatte man vielleicht einmal gemeint, die Phenothiazine und Rauwolfia-Alkaloide würden den Elektroschock entthronen, so konnte man sich doch bald davon überzeugen, daß der Elektrokrampf (wie auch das Insulin) den ersten Platz in der Therapie der Psychosen behauptet und sich nicht auf die Fälle der tödlichen Katatonie beschränken lässt."

auf eine dramatische Revolutionierung der Behandlung erheben zu können."[978] Mit dem „Elektroschock" könne man „jeden Erregungszustand unterbrechen und manchmal auch akut aufgetretene psychotische Erlebnisse kupieren. Diese Soforttherapie hat aber, wie die klinische Beobachtung ergeben hat, nur in seltenen Fällen, z.B. bei der akuten Katatonie, bei manchen schizophrenen Exazerbationen und bei manchen manischen Erregungszuständen, einen Sinn. Unbedacht angewandt, verschleiert der Elektroschock das Bild der Psychose und erschwert [aufgrund des amnestischen Syndroms, GH] den psychotherapeutischen Kontakt [aufgrund des amnestischen Syndroms, GH]. Die Hauptdomäne des Elektroschocks ist nach wie vor die Behandlung der endogenen Depression".[979]

Demnach waren die neuen Psychopharmaka in den ‚Gesamtbehandlungsplan' aufgenommen worden, ohne die ‚großen' körperlichen Kuren völlig zu verdrängen. So hieß es im Hoff-Lehrbuch von 1956 zur „Therapie des manisch-depressiven Irreseins", dass „bei Patienten mit manischen Phasen […] die Largactilkur[980] die Schockbehandlung" verdrängt, wohingegen „4 bis 8 Elektroschocks [genügen], um eine Unterbrechung der melancholischen Phase zu erzielen. Länger dauernde Schockbehandlungen scheinen uns [in melancholischen Phasen, GH] sinnlos zu sein."[981] Vor allem wurde auch bereits 1958 das soeben erst eingeführte erste moderne Antidepressivum Imipramin (Handelsname: Tofranil) eingesetzt,[982] zu dem Hift zwei Jahre später klarstellte: Entsprechend der „multifaktoriellen Genese" der endogenen Psychosen müsse eine multifaktorielle Therapie zum Einsatz kommen. Dies bedeute, dass man bei der Behandlung der Depression nicht zwischen Elektroschock und Tofranil entscheiden, sondern die Möglichkeiten beider Methoden nützen müsse – die er nach den Verläufen der endogenen Depression differenzierte: EKT zur schnellen Unterbrechung der „seltenen schweren Phasen" der rezidivierenden Depression; ohne EKT, wenn die „neurotische Abwehrmechanismen in den Vordergrund getreten sind […], um die psychotherapeutische Ansprechbarkeit nicht zu verschlechtern"; bei rasch schwankenden zyklischen Formen sei auf EKT und Tofranil zu verzichten und „zur Unterstützung der Psychotherapie gewöhnlich auf die Neuroleptika" zurückzugreifen.[983]

---

978 HIFT/HOFF, Die organische Therapie der Psychose (1958), S. 1048
979 Ebd., S. 1045.
980 Zur Anwendung vgl. unten S. 275-277 im Kapitel zu den Psychopharmaka.
981 HOFF, Lehrbuch der Psychiatrie (1956), S. 408f.
982 HIFT/HOFF, Die organische Therapie der Psychose (1958), S. 1045: Bisher sei „kein Neuroleptikum wirksam. Ob sich das Tofranil [als soeben eingeführtes erstes Antidepressivum, GH] bewähren wird, ist noch unsicher."
983 HIFT, Die Behandlung der endogenen Depression an der Wiener Klinik (1960), S. 289f. Diese Kombination (parallel) wurde in Wien zur Standardtherapie der Melancholie: ARNOLD, Kombinierte Elektroschock-Tofranil-

Der Fall des Buchdruckers A5147, der 1958 als 58-jähriger mit der Diagnose „endogene Depression“ und wieder 1959 aufgenommen wurde, zeigt die Kombination mit EKT und die Anwendung von Antidepressiva ohne EKT: Bei der 1. Aufnahme (32 Tage) – lt. Arztbrief wegen „rezidivierender endogener Depression mit Schlafstörungen, Angstzuständen, Selbstvorwürfen und SM-Gedanken“ und mit „deutliche Zeichen von allg. Sklerose“ – bekam er vier „vollmitigierte Elektroschocks“ und anschließend eine „Tofranilkur“ mit „3mal 2 Dragees Tofranil tgl. (die letzte Gabe nicht nach 14 Uhr)“; diese Medikamente sollten nach der Entlassung weitergenommen werden. Lt. Arztbrief am Ende der 2. Aufnahme 1959 wegen „einer rezidivierenden endogenen Depression bei beginnenden psychischen Abbauerscheinungen (Involutionsmelancholie)“ bekam der Patient „Amitriptyline (trizyklisches Antidepressivum), worauf sich sein depressiver Zustand aufhellte“. In der Fieberkurve sind tgl. „3x2 Tabl. C2103 [Bezeichnung des Amitriptylin im Teststadium] und 3x20gtt Hydergin [Co-Dergocrinmesila]“, am Abend „1 Miltaun+1 Doriden“ eingetragen und häufig „ATh“ für Arbeitstherapie – aber bei diesem zweiten Aufenthalt keine der ‚alten Kuren‘.

Die Elektrokrampftherapie schien im Wiener ‚Gesamtbehandlungsplan‘ bei schizophrenen und bei manisch-depressiven Erkrankungen auf, aber – wie die Insulinkur – kaum alleine. Sie wurde sehr oft mit den neuen Psychopharmaka, mit Arbeitstherapie und mit Psychotherapie kombiniert.

So auch in der Behandlung der Privatpatientin S14970 mit elf Aufnahmen zwischen 1954 und 1960 mit der Diagnose „rezidivierende paranoide Psychose“, die bei allen ihren Aufenthalten Elektrokrampfbehandlungen und Neuroleptika bekam.[984] Zusammenfassend hieß es sechs Monate nach der letzten Entlassung der Patientin 1960 im Arztbrief an die Innsbrucker Klinik: „Die erste Aufnahme erfolgte 1954 unter der Diagnose einer akuten schizophrenen Psychose, die einer Elektroschockbehandlung unterzogen wurde. Dann war sie […] immer wieder unter dem Bild von katatonen Exacerbationen bei uns in Behandlung. Es traten immer diffuse Wahnstimmungen mit optischen und akustischen Halluzinationen, sowie illusionären Verkennungen und zeitweisen akuten Erregungszuständen auf. Zu diesen Zeiten sperrte sich die Patientin meist in ihre Wohnung ein und war völlig in sich gekehrt, misstrauisch und verschlossen, wobei gelegentlich es zum Auftreten eines gespannten Stupors kam. Die Ausbildung eines zentrierten Wahnsystems konnten wir nie beobachten. In den Intervallen war die Patientin situativ angepasst, von einer beschränkten Kontaktfähigkeit und gesellschaftlich teilweise gut adaptiert. Allerdings war eine deutliche Dissimulationstendenz zu verzeichnen. Die letzte Aufnahme erfolgte im Juni 1960, wobei ebenfalls 4 Elektroschocks gegeben wurden. Daraufhin kam es zur raschen Rückbildung der damaligen Symptomatik. Aus der immer wieder vorgeschlagenen medikamentösen und psychotherapeutischen Behandlung“ in der Zeit zwischen den stationären Behandlungen „sprang die Patientin jedesmal sehr bald aus.“

Behandlung der Melancholie (1960), S. 255; ARNOLD/HOFF, Neuroleptika, Tranquilizer und Antidepressiva (1962), S. 43, zitiert unten S. 293f.

[984] 1958 als 45jährige bei ihrem 9. Aufenthalten bekam sie bei der Aufnahme 2 Amp. Largactil und am 2. und am 3. Tag je einen ‚E-Schock‘; obwohl auch nun „eine E-Behandlungsserie vorgesehen“ war, bekam sie schließlich eine Insulinsubschocktherapie mit 20 Gaben von 30-40E Insulin + 1A B-Kompl. und abends 3 Tabl. [à 25 mg] Largactil.

Besonders häufig wurde eine der beiden Krampftherapien (Elektro- oder Cardiazolkrampf) dem Insulinkoma „aufgesetzt".[985] Arnold, der für die Therapie der Schizophrenie an der Wiener Klinik maßgeblich war, nannte diese Kombination bei den meisten Insulinindikationen.[986] Zur Frage der medikamentösen Behandlung der Schizophrenie hielt Arnold 1960 noch strikt daran fest, dass die ‚Schocktherapien' keinesfalls durch die Psychopharmaka zu ersetzen, aber symptombezogen zu ergänzen seien. Neuroleptika seien in den Gesamtbehandlungsplan dann aufzunehmen, wenn eine akute oder langfristige Ruhigstellung nötig sei.-[987]

Die Wiener Indikationen der EKT stimmten weitgehend mit jenen französischer Autoren überein: In einem ausführlichen Artikel zur Insulin- und Krampfbehandlung der Schizophrenie von 1968[988] nannte der französische Psychiater Pierre Juillet, neben den Anwendungen der Krampfbehandlung (Elektro- und Cardiazolkrampf) im Insulinkoma, die Indikation der EKT bei folgenden schizophrenen Erkrankungen: vor allem bei der tödlichen, stuporösen Katatonie, allgemeiner bei akuten, besonders erregten und depressiven Zuständen. Die Kombination von EKT und medikamentöser Therapie scheinen für ihn jedoch, mehr als für die Wiener Autoren, die Regel gewesen zu sein. Auch für Juillet war nicht nur das Ende der Insulinkur,[989] sondern auch der Zeitpunkt nach der EKT besonders günstig für einen ‹psychotherapeutischen Ansatz›.[990]

Handelte es sich um eine endogene Depression, war nach Arnold, Hift und Hofmann (1960) die Elektrokrampfbehandlung mit einer Erfolgsquote von etwa 80% „weitaus wirksamer und vor allem ökonomischer als alle anderen Methoden."[991] Tofranil würde in Kombination den positiven Effekt des Elektroschocks unterstützen. Es könne bei PatientInnen, bei denen

---

[985] Vgl. oben im Kapitel zur Komatherapie, S. 215f.
[986] Vgl. ebd. die Tabelle zum „Gesamtbehandlungsplan" und ARNOLD, Die körperlichen Behandlungsmethoden der Schizophrenie (1960), S. 266f.
[987] ARNOLD, Die körperlichen Behandlungsmethoden der Schizophrenie (1960), S. 276f.
[988] JUILLET, Traitements insuliniques et méthodes de choc dans la schizophrénie (1968), S. 118**f**. Ähnlich auch MICHAUX, Psychiatrie (1965), S. 1004f.
[989] Vgl. Juillet zur Insulinkur als günstig für eine Psychotherapie, oben im Kapitel zur Insulinkur S. 185.
[990] Vgl. dazu HOFF, Lehrbuch der Psychiatrie (1956), S. 512, der zur „Frage der Psychotherapie im Rahmen des manisch-depressiven Krankheitsgeschehens" im Anschluss an eine EKT schreibt: „An Hand entsprechenden Erfahrungsmaterials ist die sofortige ES-Behandlung und dann folgende Psychotherapie als Methode der Wahl zu betrachten, da der durch Psychotherapie allein erzielbare Effekt in Bezug auf den Zeitaufwand in gar keinem Verhältnis zu den gleichen Faktoren bei der ES-Behandlung steht."
[991] ARNOLD/HIFT/HOFMANN, Die Therapie des manisch-depressiven Krankheitsgeschehens (1960), S. 278f.

Antriebslosigkeit und Hemmungszustände während einer depressiven Phase im Vordergrund stünden, alleine probiert werden. Allerdings sei eine Elektrokrampfbehandlung sofort einzuleiten, wenn der gewünschte Effekt nach acht bis 14 Tagen ausbleibt.[992]

Da auf der Wiener Klinik vor allem schwere endogene Depressionen stationär aufgenommen wurden, während man leichte und neurotische Depressionen oft auch ambulant behandelte,[993] ergibt die aus den Krankenakten erstellte Statistik eine sehr hohe Zahl an PatientInnen, die mit Elektroschocks behandelt wurden. Zur „Einstellung unserer Klinik zur Behandlung der Melancholie" schrieb Hift 1960 in der *Wiener Klinischen Wochenschrift*: „Wir haben keine prinzipielle Abneigung gegen den Elektroschock. Die Technik des Elektroschocks ist heute so weit fortgeschritten, daß er weniger Nebenerscheinungen und Komplikationen verursacht als die meisten medikamentösen Therapien. Der Elektroschock kann noch in Fällen appliziert werden, bei denen wegen hohen Alters bzw. wegen Herz-, Nieren- oder Leberschäden die Neuroleptika oder Thymoleptika kontraindiziert sind."[994] Die oft von PsychiaterInnen als Problem wahrgenommene Abneigung der PatientInnen gegen den Elektroschock sei durch „psychologische Führung" leicht auszugleichen und würde die Abneigung gegen Neuroleptika nicht übersteigen. Als Nachteil der Elektrokrampftherapie benannte er das amnestische Syndrom, welches die PatientInnen als „unangenehm" empfinden würden und durch das ein „psychotherapeutischer Kontakt" erschwert würde. Andererseits lobte er den ‚Elektroschock' dafür, dass er anders als Medikamente einen schnellen Erfolg bringen würde, den der Arzt brauche, um das Vertrauen der PatientInnen als Voraussetzung für eine „*tiefergehende Psychotherapie*" zu gewinnen. Und „der Notwendigkeit einer tiefergehenden Psychotherapie" galt nach Hift „die Hauptüberlegung bei der Aufstellung des Therapieplans".[995]

Im selben Jahr 1960 publizierten in dem von Hoff herausgegebenen Sammelband die drei Wiener Kliniker Arnold, Hift und Hofmann zur „Therapie des manisch-depressiven Krankheitsgeschehens". Bei den nur schwer in den Griff zu bekommenden rezidivierenden Depressionen bzw. „chronischen Melancholien" sprachen sie sich ebenfalls an erster Stelle für die Elektrokrampfbehandlung aus, die in besonders schweren Verlaufsformen durch eine

[992] Ebd., S. 293f.
[993] HIFT, Die Behandlung der endogenen Depression an der Wiener Klinik (1960), S. 289.
[994] Ebd.
[995] Ebd. Vgl. Juillet 1968 oben S. 255 zum Ende der EKT als idealen Zeitpunkt für den Beginn einer Psychotherapie.

Cardiazolkrampfserie zu ergänzen sei.[996] Eine medikamentöse oder psychotherapeutische Behandlung könne bei den chronisch wiederkehrenden Depressionen ebenfalls versucht werden, allerdings nicht als eigenständige Therapie, sondern nur als Erweiterung der EKT. Chronische oder rezidivierende Depressionen, die von ständigen Suizidgedanken begleitet waren und sich als resistent gegen alle anderen Behandlungsformen erwiesen hätten, wären in letzter Konsequenz einer Lobotomie zu unterziehen.[997]

Auch bei den sogenannten Laktationspsychosen (postpartalen Psychosen) war auf der Wiener Klinik die Elektrokrampfbehandlung die erste Wahl, wenn nicht ein schizophrener Prozess hinter der Psychose stand. In letzterem Fall sei nach dem Abstillen, das auch vor einer Elektrokrampfbehandlung zu erfolgen hatte, eine Insulinkomabehandlung durchzuführen.[998] Bei manischen PatientInnen sei die EKT nur wegen der beruhigenden Wirkung bei bestimmten Symptomen zu wählen. So wurde um 1960 der Elektroschock bei Legierungspsychosen oder katatonen Exazerbationen mit manischen Bildern nach der Literatur nur noch dann eingesetzt, wenn die PatientInnen eine starke psychomotorische Unruhe aufwiesen.[999]

Die Elektrokrampfbehandlung wurde 1961 und 1967 auch von Walter Spiel in seinen beiden Monographien zur Kinder- und Jugendpsychiatrie besprochen, wobei die Indikationen sich in der Altersbegrenzung (bei affektiven Störungen mit zehn Jahren) und sonst nur wenig unterscheiden. So heißt es in der Zusammenfassung der detailreichen Ausführungen, die Walter Spiel auch bereits 1961 als Indikation beschrieben hatte,[1000] es ergäbe „sich eine gewisse Einengung der Indikation auf die agitiert katatonischen Bilder und die stuporöse[n] gehemmten Zustandsbilder.[1001]

---

996 Der kombinierte „Cardiazol-E-Schock“ wird in einzelnen Fällen in den PatientInnenakten genannt: vgl. oben S. 90 und im Kapitel zur Insulinkur S. 215 Anm. 838.

997 ARNOLD/HIFT/HOFMANN, Die Therapie des manisch-depressiven Krankheitsgeschehens (1960), S. 285.

998 Vgl. ebd., S. 284f.

999 Vgl. ebd., S. 281.

1000 SPIEL, Die endogenen Psychosen des Kindes- und Jugendalters (1961), S. 106f. Hier berichtet er auch aus eigener Erfahrung von einer „eklatante[n] eindeutige[n] und schlagartige[n] Besserung“ „bei den in der Pubertät auftretenden […] langdauernden, tiefen depressiven Verstimmungszuständen.“ Lt. Datenbank wurde etwa 1956 die 13-jährige Patientin AK682 mit der Diagnose „endogene Depression i. d. Pubertät“ mit einer EKT behandelt.

1001 SPIEL, Die Therapie in der Kinder- und Jugendpsychiatrie (1967), S. 132f. Außerdem nannte er einige Besonderheiten in der Durchführung der EKT bei Kindern ab dem sechsten Lebensjahr (diese Altersgrenze ist auch aus den überlieferten Akten zur Kinderstation ersichtlich): sie würde genauso wie beim Erwachsenen erfolgen, jedoch häufiger als „vollmitigierter Narkose-Elektroschock“ („zuerst ½ mg Atropinsulfat iv., dann […] 0,6 bis1 g Kemital oder ein anderes kurzwirkendes Barbiturat“, schläft der Patient „0,1 mg pro kg/Körpergewicht Lysthenon“) und üblich in einer höheren Anzahl der Anwendungen (durchschnittlich 10 bis

In Wien kam es auch – wie bei der Insulinkomatherapie – in den 1960er Jahren zu einem Rückgang der EKT.[1002] Auch hier wurden in einigen Indikationen medikamentöse Therapien bevorzugt, wenn auch nicht in so hohem Ausmaß wie etwa im Burghölzli, wo Mitte der 1960er Jahre die EKT kaum[1003] mehr angewandt worden sein dürfte. Wesentlich für die breite Durchsetzung der neuen Antidepressive gegenüber dem ‚Elektroschock' (wie der Neuroleptika gegenüber der Insulinkur) wurde in der Zeit die „Einfachheit der Anwendung" von Medikamenten und ihre höhere Akzeptanz bei PatientInnen und ihren Angerhörigen – weniger die Diskussion um die therapeutische Wirksamkeit – gesehen.[1004]

2.3.5 Statistische Auswertung zur Elektrokrampftherapie (EKT)

Die Anwendung der EKT blieb an der Wiener Klinik in der ‚Ära Hoff' häufig. Durch den Einsatz der Neuroleptika (1952) und vor allem der Antidepressiva (1958) sowie durch die Spezifizierung der Anwendungen ging sie jedoch sukzessive in diesen Jahren auch in Wien deutlich zurück.

---

12 „Schocks"); diese Intensivierung sei bei Kindern notwendig, „um psychotische Störungen zum Verschwinden zu bringen". Beim Aufwachen des Kindes sollte sein/e Psychotherapeut/in anwesend sein.

[1002] Vgl. unten im Abschnitt zu den Antidepressiva S. 292f.

[1003] Die Grafik in TANNER/MEIER/HÜRLIMANN/BERNET, Zwangsmassnahmen in der Züricher Psychiatrie (2002), S. 93, zeigt im Stichjahr 1969 noch ca. 5 Fälle von EKT. Vgl. dazu die Erinnerungen des Wiener Psychiaters Eberhard Gabriel, der 1966 am Burghölzli gearbeitet hat: oben S. 32 Anm. 97 und (zur IKT) oben S. 180 Anm. 684.

[1004] Vgl. oben S. 184 die Zitate aus PICHOT, Vergleich der verschiedenen Behandlungsmethoden in der Psychiatrie (1960), S. 737.

2.3.5.1 Häufigkeitsverteilung über die Jahre

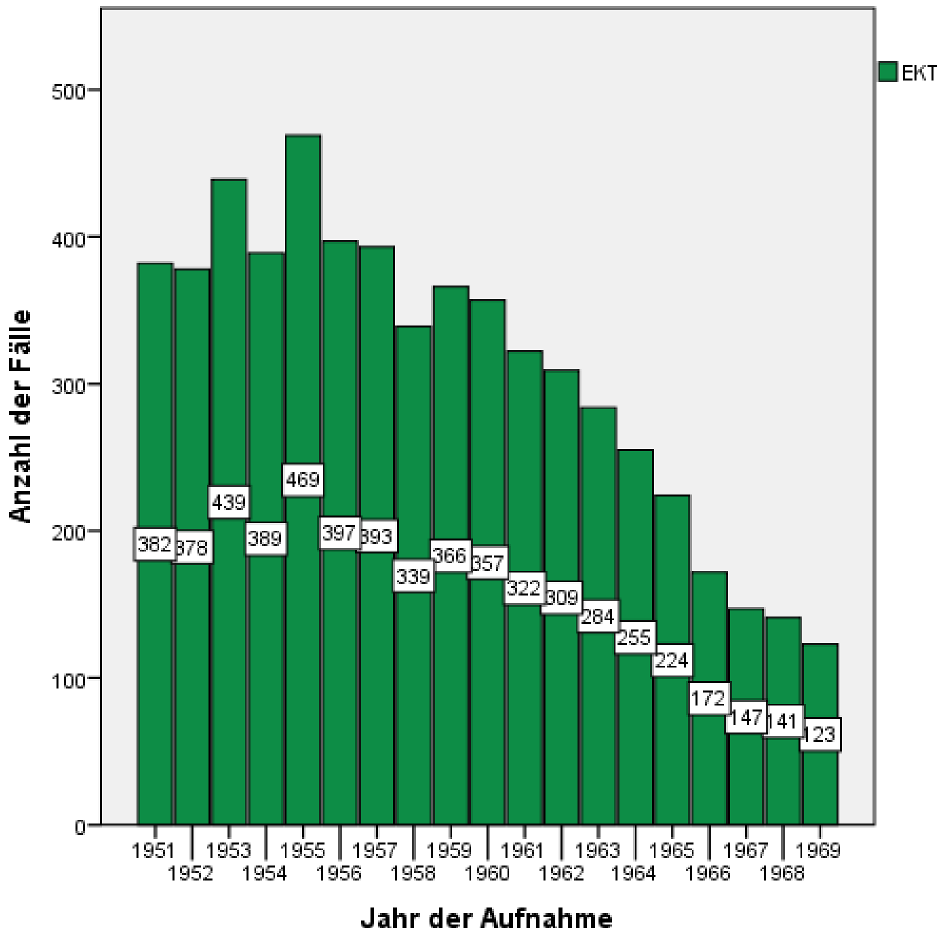

Abb. 11 Balkendiagramm, absolute Anzahl der EKT (1951-1969), Pat. mit Einschlusskriterien (Diagnosen und Aufenthaltsdauer), n = 5.886 aus 14.919.

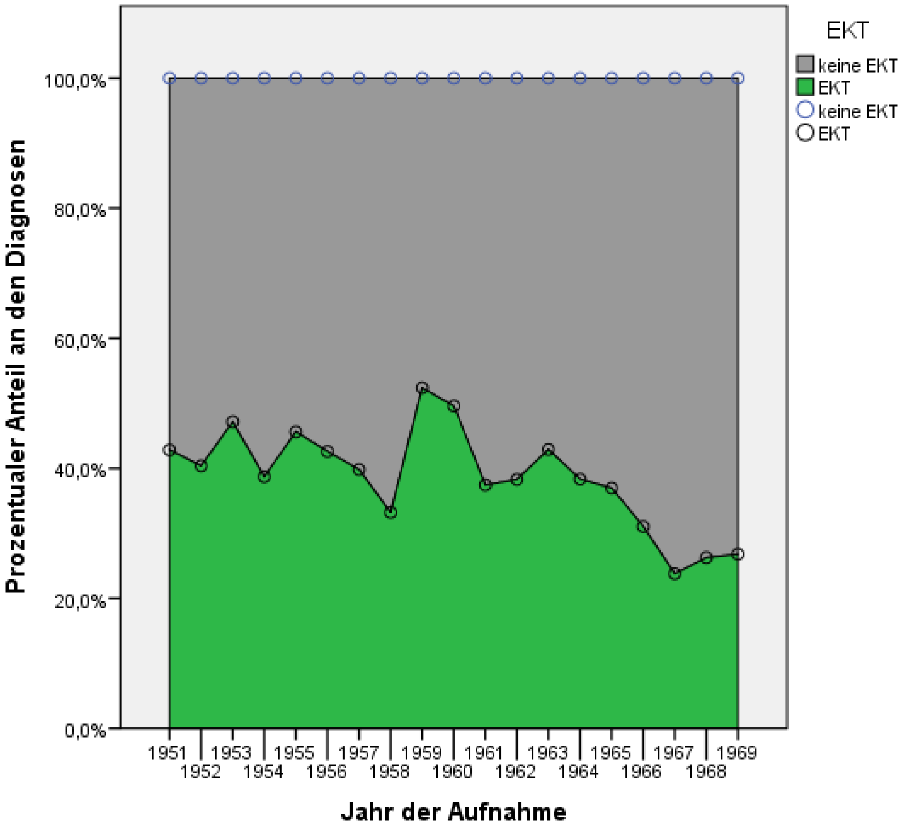

Abb. 12 Flächendiagramm (auf 100%), relative Häufigkeit der EKT bei den aufgrund der entsprechenden Einschlusskriterien (Diagnose, Aufenthaltsdauer) aufgenommen Akten, n = 5.886 zu 14.919.

Die Elektrokrampftherapie war an der Wiener Klinik die häufigste der in der Zwischenkriegszeit entwickelten ‚alten Kuren' (der ‚Schock'- und Fieberkuren). Bis 1965 schwankt der Anteil der mit einer EKT behandelten Fälle zu den insgesamt nach den Einschlusskriterien in der Datenbank aufgenommen Fällen zwischen 35 und 45% (mit einem Maximum im Jahr 1959 von über 50%), um nach 1965 auf unter 30% abzufallen. Es wurden laut Datenbank fast ausschließlich schizophrene und affektive Erkrankungen (in einer annähernd gleichen Anzahl) mit EKT behandelt. Andere Diagnosen waren Zweitdiagnosen einer schizophrenen oder affektiven Störung und waren nicht die Indikation zur EKT. Bei beiden Erkrankungen führte die Einführung der Neuroleptika ab 1952/53 und bei manisch-depressiven Erkrankungen besonders die Anwendung der neuen Antidepressive seit 1958 zu einer Abnahme der EKT.

2.3.5.2 Alter und Aufenthaltsdauer

Das Alter der PatientInnen, die eine EKT erhalten haben, liegt etwa auf dem Altersmedian aller aufgenommenen Fälle. Anders war es bei der Insulinkomatherapie, die für gewöhnlich nur einmal und zwar im frühen Erkrankungsalter und demnach im frühen Alter der PatientInnen durchgeführt werden sollte; anders auch bei der Malariakur, die in dieser Zeit bei einem / einer Patienten/in (fast) nur einmal gegeben wurde und bei den Diagnosen abgesehen von der Neurolues[1005] meistens in jungem Alter. Das dürfte bei diesen beiden Therapien zu einem jungen Altersdurchschnitt geführt haben, wohingegen die EKT immer wieder und unabhängig vom Alter bei affektiven und schizophrenen Störungen angewandt wurde. Der Altersdurchschnitt lag bei 38 Jahren.[1006]

Die Aufenthaltsdauer lag mit dem Median bei 35 Tagen, d.h. wie bei allen ‚Schock'- und Fieberkuren über der Dauer eines durchschnittlichen Aufenthalts (Median bei 25 Tagen), ist jedoch im Vergleich zur Malaria-, Insulin- und (kombinierten) Cardiazoltherapie wesentlich kürzer.

An der Kinderstation wurde nach den überlieferten Akten die EKT ab dem Alter von sechs Jahren gegeben, wobei sich die 21 Fälle mit nur je einem Fall im Alter von sechs, sieben und 17 Jahren auf zehn- bis 15-Jährige verteilen.[1007] Drei PatientInnen hatten die an der Klinik übliche Kombination mit einer Insulinkomatherapie; bei zwei ausländischen PrivatpatientInnen im Alter von sechs und elf Jahren wurde die seltene Kombination mit Malariafieberkur angewandt.[1008]

[1005] Auch bei der Schizophrenie wurde sie manchmal als Therapie gegeben, nachdem alle anderen keinen Erfolg brachten: vgl. BLEULER, Lehrbuch der Psychiatrie ([10]1960), S. 405 (oben S. 31 Anm 93) und vgl. auch die Erfahrungen des Zeitzeuge an der Salpêtrière Anfang der 1960er Jahre (oben S. 81f.).
[1006] Vgl. der Median nach der Quartillen-Berechnung im Projektbericht (2015), S. 197.
[1007] GEIGER, Kinderstation (2015), S. 265. Zur Indikation vgl. oben S. 257 Anm. 1000f.
[1008] Der 6jährige ausländische Privatpatient KI1861 hatte die Diagnose „Heller Psychose" und bekam bei seinem Aufenthalt 1964/65 (2 ½ Monate) zu EKT und Malariakur auch Majeptil; der 11jährige ausländische Privatpatient KI773 hatte die Diagnoseeinträge „Encephalopathie, Schwachsinn, Zust. nach ungeklärter Erkrankung im 3. Lj. (Heller Psychose, [...]?)" und bekam bei seinem 1jährigen Aufenthalt 1956/57 zur EKT und Malariakur zusätzlich Largactil, Serpasil und Psychotherapie.

2.3.5.3 Diagnosen zur Elektrokrampftherapie

Die folgende Tabelle zeigt die Zahl der Anwendungen der EKT in den Erwachsenenstationen bei PatientInnen mit einer der fünf Diagnosen (Neurolues, Intelligenzmängel, Schizophrenie, affektive Störungen, Psychopathie), wobei zu beachten ist, dass PatientInnen mehrfach gezählt wurden, bei denen in der Diagnosezeile mehrere der fünf Diagnosen bzw. Mischdiagnosen (wie Legierungspsychose oder Pfropfschizophrenie) genannt sind; in der Auswertung zu dieser Tabelle sind keine Überlegungen zu einer Zuordnung der EKT eingeflossen. Rot markiert sind jeweils die im Jahr genannte höchste Zahl, die erwartungsgemäß immer in eine der beiden Hauptindikationsfelder fiel.[1009]

	**Intelligenz-mängel**	**Neurolues**	**Schizophrene Erkrankungen**	**Psychopathie**	**Manisch-depr. Erkrankungen**	**Gesamt (alle Patienten)**
	Anzahl Fälle	Anzahl Fälle	Anzahl Fälle	Anzahl Fälle	Anzahl Fälle	Anzahl Fälle
**1951**	4	3	235	9	169	382
**1952**	2	0	187	5	205	378
**1953**	2	4	221	3	253	439
**1954**	5	5	200	0	211	389
**1955**	5	1	221	1	289	469
**1956**	8	0	198	5	223	397
**1957**	7	1	217	3	209	393
**1958**	1	0	182	1	194	339
**1959**	2	0	206	1	205	366
**1960**	6	3	188	4	197	357
**1961**	5	0	190	0	171	322
**1962**	6	0	181	2	170	309
**1963**	3	1	169	2	150	284
**1964**	4	0	181	0	127	255
**1965**	5	0	145	0	113	224
**1966**	1	0	115	0	79	172
**1967**	2	0	96	0	70	147
**1968**	1	0	90	0	71	141
**1969**	1	0	91	0	49	123
**Gesamt**	70	18	3313	36	3155	5886

Tab. 14 Diagnosen und EKT (1951-1969), bei den aufgrund der entsprechenden Einschlusskriterien (Diagnose, Aufenthaltsdauer) aufgenommen Akten, n = 5.886, Mehrfachnennungen bei Diagnosen sind möglich.

1009 Zu beachten ist außerdem, dass die EKT auch bei Diagnosen gegeben wurden, die nicht in die Datenbank aufscheinen. So lt. Hoff-Skriptum bei PatientInnen mit der Diagnose „Epilepsie“, bei der in der Phase, in der sich ein Anfall ankündigt, eine Anwendung sinnvoll zur künstlichen Auslösung des Anfalls sein könne, um den Anfall kontrollieren zu können: Hoff-Skriptum, Allgemeine Psychiatrie [um 1961], S. 37.

a) Schizophrene Erkrankungen

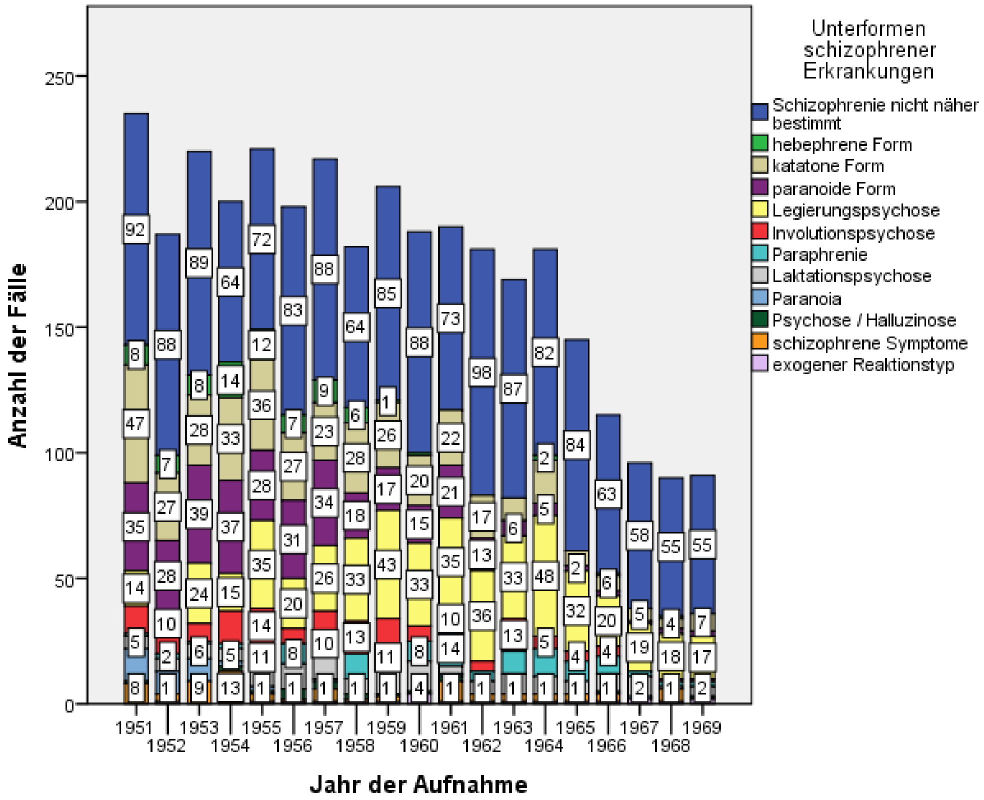

Abb. 13 Gestapeltes Balkendiagramm, EKT + Unterformen der schizophrenen Erkrankungen (1951-1969), Pat. länger als 4 Tage stationär, n = 3.313.

Häufig unter den Schizophrenien, die mit EKT behandelt wurden, sind Schizophrenien, bei denen es zu Halluzinationen und Wahnvorstellungen gekommen ist (paranoide Schizophrenie und Legierungspsychosen) sowie die katatone Schizophrenie, bei der die EKT die Therapie erster Wahl war. Bei PatientInnen im ersten schizophrenen Schub, aber auch wenn dieser nicht länger als eineinhalb Jahre zurücklag, blieb in Wien die Insulinkomatherapie Standard; auch in diesen Fällen wurde die Behandlung in der Regel mit einer Serie von EKT zur Linderung der akuten Symptome und zur Beruhigung eingeleitet.[1010]

[1010] Zum 1965 16-jährigen Zögling aus dem Heim in der Seuttergasse 29 im 13. Bezirk, S7504, hieß es in der Anamnese: „Der Patient äußert, daß ihn alle umbringen wollen, er weiss es genau, man gebe ihm eine Injektion, um ihn zu töten, und wird wegen seiner großen Ängstlichkeit sofort nach Einholung einer internistischen Kontraindikation einer Elektroschockbehandlung zugeführt." Er bekam während seines 110-tägigen (einzigen) Aufenthalts sechs ‚E-Schocks' und drei Wochen später wurde mit einer Insulintherapie begonnen mit insgesamt 60 Komata; vier Cardiazolschocks wurden dem Koma „aufgesetzt"; er wurde in das Rehabilitationszentrum der Klinik in Maria Lanzendorf entlassen. Die EKT war also zur Behandlung des Symptoms, zur Beruhigung des Patienten, die IKT zur Behandlung der Grundkrankheit.

Bereits in der Publikation von Hoff und Arnold 1954/55 (deutsch und französisch) wird der Erfolg der EKT mit ca. 20 Anwendungen bei schizophrenen Erkrankungen zwar als häufig hervorragend aber nur vorübergehend eingeschätzt; sie hielten sie deshalb im Bereich der Schizophrenie nur bei den katatonen Formen angezeigt.[1011] Die in der Abb. 13 und in den folgenden Ausführungen genannte Häufigkeit ergibt sich aus den bei vielen Aufenthalten nur einzelnen Anwendungen (‚Schocks') und aus den – auch in der Publikation von 1954/55 besprochenen – Anwendungen in Kombination mit der Insulinkomatherapie (‚ES' dem Koma aufgesetzt).

57,5% der PatientInnen mit paranoider Schizophrenie wurden mit einer EKT behandelt (336 von 584 Fällen). Dieser Anteil wurde im Verlauf des Untersuchungszeitraumes größer. So lag er in den 1950er Jahren bei 54,4% (267 von 491 Fällen) und in den 1960er Jahren bei 74,1% (69 von 93 Fällen). Für die Ausweitung der Indikation findet sich jedoch keine Erklärung in den Krankengeschichten oder in den Publikationen.[1012]

88,3% der katatonen Schizophrenien wurden mit einer EKT behandelt (388 von 439 Fällen). Der Anteil der mit EKT-Behandelten blieb bei der Katatonie über den Untersuchungszeitraum in etwa gleich, so sind in den 1950er Jahren 89,6% (275 von 307 Fällen) und in den 1960er Jahren 85,6% (113 von 132 Fällen) mit einer EKT behandelt worden.

Darüber hinaus lassen sich in der statistischen Auswertung der repräsentativen Stichprobe, die im Projekt im Rahmen der Pilotstudie zu den Jahren 1955 bis 1960 gemacht wurde, Aussagen zu Unterschieden bei der Behandlung von Frauen und Männern treffen, was in den Fachpublikationen kaum thematisiert wird.

[1011] HOFF/ARNOLD, Die Therapie der Schizophrenie (1954), S. 348: Die EKT habe jedoch „das Feld des manisch-depressiven Irreseins erobert".

[1012] Die Indikation – so Eberhard Gabriel - dürfte von Anfang an in Hinblick auf die produktive Symptomatik (und vielleicht auch auf affektive Symptome in deren Hintergrund) gegeben worden sein.

			Therapie 1: E-Schock		
			ja	nein	Gesamt
			Anzahl	Anzahl	Anzahl
männlich	Unterform der Schizophrenie	Keine schizophrene Störung	49	86	135
		Schizophrenie nicht näher bestimmt	22	26	48
		Hebephrene Form	1	5	6
		Katatone Form	3	0	3
		Paranoide Form	6	6	12
		Sonst. schizophrene Erkrankungen*	7	5	12
		Gesamt	88	128	216
weiblich	Unterform der Schizophrenie	Keine schizophrene Störung	69	140	209
		Schizophrenie nicht näher bestimmt	20	14	34
		Hebephrene Form	2	1	3
		Katatone Form	16	1	17
		Paranoide Form	7	8	15
		Sonst. schizophrene Erkrankungen*	27	4	31
		Gesamt	141	168	309
Gesamt	Unterform der Schizophrenie	Keine schizophrene Störung	118	226	344
		Schizophrenie nicht näher bestimmt	42	40	82
		Hebephrene Form	3	6	9
		Katatone Form	19	1	20
		Paranoide Form	13	14	27
		Sonst. schizophrene Erkrankungen*	34	9	43
		Gesamt	229	296	525

Tab. 15 Verhältnis vom Elektroschock zum Geschlecht und der Unterform der Schizophrenie 1955-1960 (Stichprobe, n=525); *mit anderen spezifizierten Diagnosen aus dem Diagnosefeld der schizophrenen Erkrankungen.

Mithilfe der Tabelle lässt sich errechnen, dass etwa 1,16mal mehr Männer mit einer Schizophrenie diagnostiziert worden sind als Frauen. Während diese minimalen Abweichungen im Normalbereich liegen, gibt es einen klaren Unterschied in der Applikation der EKT. So wurden 72% der schizophrenen Frauen, aber nur 48% der schizophrenen Männer mit EKT behandelt. Daraus ergibt sich, dass mit Schizophrenie Frauen im Vergleich zu Männern mit 1,5-facher Wahrscheinlichkeit (Odds Ratio) mit einer EKT behandelt wurden. Hingegen liegt in unserer Stichprobe bei einer Auswertung aller Diagnosen (nicht nur der Schizophrenie) der Faktor für die EKT nur bei 1,12 (Frauen : Männer). Für die auffallend häufigere Anwendung der EKT bei schizophren erkrankten Frauen wurden in der zeitgenössischen Literatur keine Erklärung gefunden; auch die Frage, ob die indikationsstellenden Leiter der Stationen dabei eine Rolle spielten, konnte nicht geklärt werden.

Zudem wird aus dieser Stichprobe ersichtlich, dass eine katatone Schizophrenie auch in diesem Zeitabschnitt (1955 – 1960) fast immer mit einer EKT behandelt wurde (19 von 20 katatonen PatientInnen). Praxis und Publikationen sind hier deckungsgleich. Auch paranoide Formen der Schizophrenie werden häufig, genauer gesagt in der Hälfte der Fälle (13 von 27) mit EKT behandelt.

b) Manisch-depressiver Formenkreis

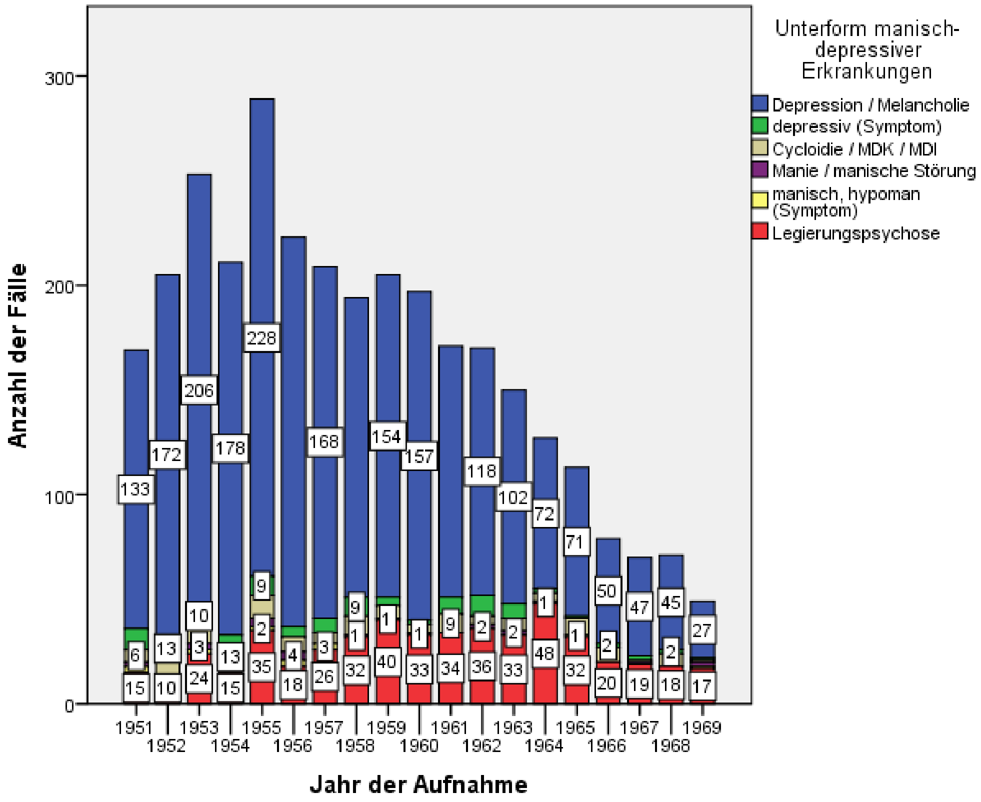

Abb. 14 Gestapeltes Balkendiagramm, EKT + Unterformen der manisch-depressiven Erkrankungen (1951-1969), Pat. länger als 4 Tage stationär, n = 3.155.

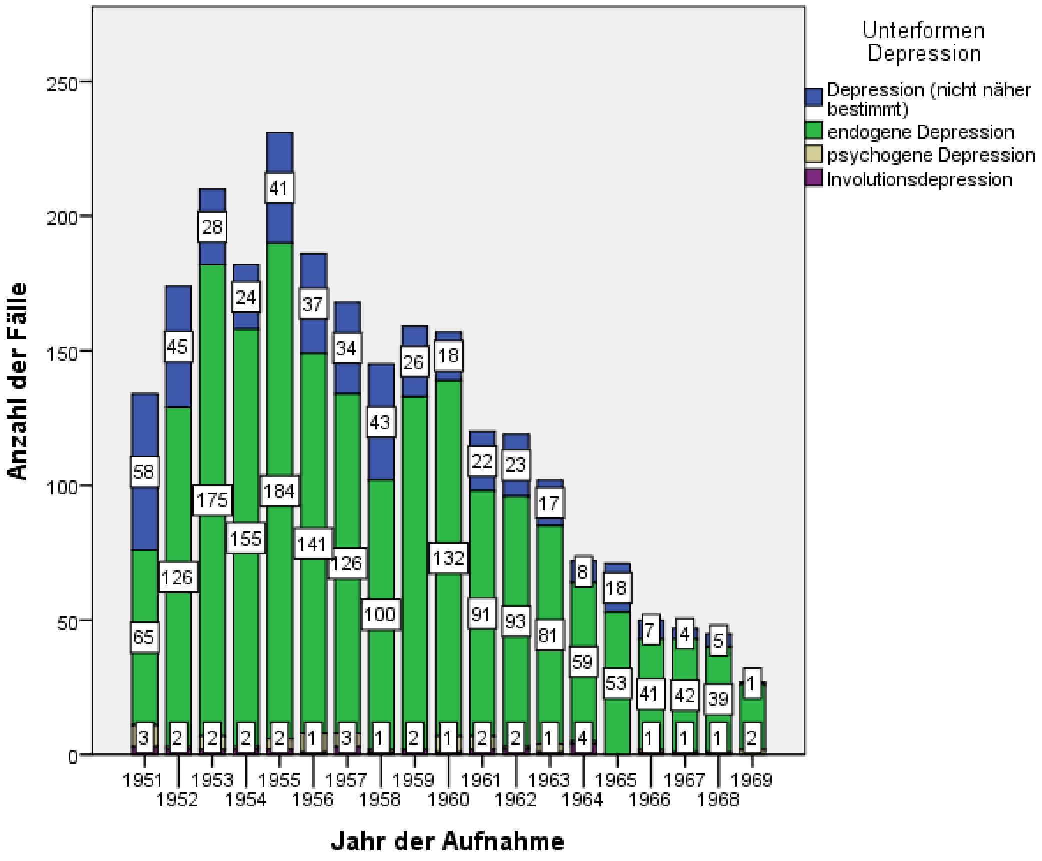

Abb. 15 Gestapeltes Balkendiagramm, EKT + Unterformen der Depression (1951-1969), Pat. länger als 4 Tage stationär, n = 2.399.

Die Häufigkeit der Anwendung bei Legierungspsychosen wurde bereits bei der Schizophrenie erwähnt. Das Hauptanwendungsgebiet aus dem Diagnosefeld manisch-depressive Erkrankungen sind die Depressionen.

Unter den Depressionen spielen die psychogene Depression und die Involutionsdepression eigentlich keine Rolle. EKT war eine typische Behandlung für die endogene Depression: So wurden 63,2% aller endogenen Depressionen mit einer EKT behandelt (1.858 von 2.941 Fällen). In den 1950er Jahren lag der Anteil der EKT bei endogenen Depressionen noch bei 80,5% (1.203 von 1.494 Fällen), in den 1960er Jahren lag er nur noch bei 45,3% (655 von 1.447 Fällen), vermutlich da nun mehr PatientInnen nur mit Antidepressiva behandelt wurden.

Auch zur Verteilung von Diagnosen und Therapien zwischen Frauen und Männern bei verschiedenen depressiven Erkrankungen ergibt die statistische Auswertung der repräsentativen Stichprobe für die Jahre 1955 – 1960 Unterschiede in der Verteilung von Diagnosen und Therapien zwischen Frauen und Männern. Die Unterschiede in der Häufigkeit

der Diagnose Depression zwischen den Geschlechtern war auch in diesen Jahren Thema der wissenschaftlichen Diskussion. Um die unterschiedlichen Gewichtungen hinreichend erklären zu können, müsste sich hier freilich weiterführend eine eingehende geschlechter- und medizinhistorische Untersuchung anschließen, in der insbesondere nach dem Einfluss der Kategorie Geschlecht auf die Konstruktion von Diagnosen und die Praxis ihrer Vergabe zu fragen wäre.

			Therapie 1: E-Schock		
			ja	nein	Gesamt
			Anzahl	Anzahl	Anzahl
männlich	Unterform der affektiven Störungen	Keine affektive Störung	38	83	121
		Depression (nicht endo./ nicht psych.)	16	33	49
		Endogene Depression	31	5	36
		Psychogene Depression	0	0	0
		Manie / manische Störung	1	1	2
		Cycloidie/MDK (bipolare Störung)	2	6	8
		Gesamt	88	128	216
weiblich	Unterform der affektiven Störungen	Keine affektive Störung	72	58	130
		Depression (nicht endo./ nicht psych.)	17	24	41
		Endogene Depression	45	13	58
		Psychogene Depression	0	63	63
		Manie / manische Störung	4	1	5
		Cycloidie/MDK (bipolare Störung)	3	9	12
		Gesamt	141	168	309
Gesamt	Unterform der affektiven Störungen	Keine affektive Störung	110	141	251
		Depression (nicht endo./ nicht psych.)	33	57	90
		Endogene Depression	76	18	94
		Psychogene Depression	0	63	63
		Manie / manische Störung	5	2	7
		Cycloidie/MDK (bipolare Störung)	5	15	20
		Gesamt	229	296	525

Tab. 16 Verhältnis von Elektrokrampftherapie zum Geschlecht und der Unterform der affektiven Störungen (damals „affektive Psychosen“) 1955-1960 (Stichprobe, n=525).

Anhand der Tabelle wird ersichtlich, dass etwa 1,32mal mehr Frauen als Männer mit einer Depression diagnostiziert wurden. In der Auswertung der Gesamtdatenbank (1951 – 1969) sind Frauen zu 65% in dieser größten Diagnosegruppe vertreten (s. oben S. 41, Tab. 3).

Etwa 55,3% aller Männer mit einer Depression (ohne bipolare Störung) haben eine EKT erhalten (47/85), aber nur ≈ 38,3% der Frauen mit denselben Diagnosen (62/162). Dies ergibt eine höhere Wahrscheinlichkeit von 1,44 (Odds Ratio), dass Männer mit diesen Diagnosen eine EKT erhielten. Ganz allgemein sind also depressive Männer häufiger mit EKT behandelt worden. Dieses Ergebnis überrascht aber nur bei einem oberflächlichen Blick, da bei Männern nie eine psychogene Depression diagnostiziert wurde, welche wiederum nie mit EKT behandelt wurde. Dieses Ergebnis relativiert sich, sobald die Geschlechter-Verteilung der EKT zum Beispiel bei der endogenen Depression verglichen wird; hier liegt die Odds Ratio, die Wahrscheinlichkeit, dass Männer häufiger als Frauen mit EKT behandelt wurden, nur noch bei 1,1.

Lt. Datenbank zu den überlieferten Akten der Kinderstation dürften dort deutlich mehr Mädchen (15) als Knaben (6) mit EKT behandelt worden sein. Die EKT wurde mit den neuen Medikamenten (Largactil, Tofranil, Valium, Nozinan und Melleretten) sowie meistens mit einer Psychotherapie kombiniert, aber nur fünfmal mit einer anderen der ‚großen' alten Kuren.[1013]

Die endogene Depression wurde in über 80% der Fälle mit EKT behandelt, dabei spielt das Geschlecht keine Rolle. Es zeigt sich also, dass eine endogene Depression üblicherweise mit EKT, die psychogene Depression hingegen mit anderen Therapien behandelt wurde. Interessant ist zudem, dass die psychogene Depression nicht ein einziges Mal bei einem Mann diagnostiziert wurde, aber bei 63 Frauen. Bei Männern ist in diesem Zusammenhang auf die häufige Diagnose „Alc. chronisch" hinzuweisen, die das psychopathologische Erscheinungsbild in vielen Fällen eingeschlossen haben dürfte.

[1013] Dreimal mit einer Insulinkur (dem Koma aufgesetzt) und zweimal mit einer Malariafiebertherapie.

2.4 Psychopharmaka an der Klinik Hoff[1014]

In den 1950er Jahren wurden mit den ersten Neuroleptika und Antidepressiva auch mögliche Alternativen zu den „alten Kuren“ entwickelt.[1015] Eine statistische Analyse wie sie bei den Schock- und Fiebertherapien erfolgte, war im Rahmen des Projekts für die Psychopharmaka aufgrund zweier Faktoren nicht vorgesehen:

(1) Viele der Psychopharmaka weisen (auch heute noch) ein sehr weites Indikationsspektrum auf, so wurden zum Beispiel niedrigpotente Neuroleptika bei Einschlafstörungen, Angststörungen, Neurosen, Psychosen und zur allgemeinen Beruhigung eingesetzt.

(2) Vor allem in den 1960er Jahren ist ein regelrechter Boom in der Entwicklung der Psychopharmaka ausgelöst worden, so dass innerhalb kurzer Zeit eine Vielzahl unterschiedlicher Medikamente getestet wurden und auf den Markt kamen, die teilweise aber ebenso schnell auch wieder aus dem klinischen Alltag verschwanden.

Dadurch würde sich eine statistische Auswertung, wie sie bei den Schock- und Fiebertherapien durchgeführt wurde, bei den Medikamenten als weitaus schwieriger erweisen. Vor allem das breite Anwendungsgebiet lässt keine eindeutige Medikamentenklasse-Diagnose-Korrelation zu. So wird mithilfe einer groben Einteilung der Medikamente in klinischen Klassen (Analeptika, Antidepressiva, Neuroleptika, Tranquilizer) kein Unterschied sichtbar: In der repräsentativen Stichprobe zu den Jahren 1955–1960 (in ihr wurden zusätzliche Daten aus den Krankenakten aufgenommen, so auch die Psychopharmaka), sind von den 525 Fällen, die aus 5.140 Fällen gezogen wurden, etwa 44,6 % der schizophrenen PatientInnen, aber auch 31,3 % der depressiven PatientInnen mit einem Neuroleptikum behandelt worden. Auch haben 67,7 % der schizophrenen und 69,9 % der depressiven PatientInnen Tranquilizer erhalten. Was jedoch sehr wohl aus diesen Zahlen hervorgeht, ist, dass die Medikamente nicht krankheitsspezifisch, sondern symptomenspezifisch eingesetzt wurden, was auch mit den Aussagen in den Publikationen der Wiener Klinik

[1014] In einem Verzeichnis der Medikamente am Ende der Publikation sind die Handelsnamen und die entsprechenden Wirkstoffe angegeben. Die folgenden Ausführungen beziehen sich auf die repräsentative Stichprobe zu den Jahren 1955–1960, auf Fallbeispiele aus den PatientInnenakten und auf zeitgenössische Publikationen.

[1015] Dazu die Untersuchungen von HALL, Zur Geschichte der deutschen Pharmakopsychiatrie von 1844 Bis 1952 (1997), und von BALZ, Zwischen Wirkung und Erfahrung – eine Geschichte der Psychopharmaka (2010).

übereinstimmt.[1016] Walter Spiel scheint 1967 in der Konzentration der zahlreichen Publikationen auf „die erfolgreiche Behandlung bestimmter Symptome und Syndrome“ mit Psychopharmaka eine Rückkehr zur Symptombehandlung und Abwendung „von der ätiologischen Denkweise und Therapie“ befürchtet zu haben.[1017]

Die Hoffnungen und Erwartungen einiger PsychiaterInnen bereits in der ersten Zeit nach der Einführung der Neuroleptika, dass diese die Insulinkoma- und die Elektrokrampftherapie in der Behandlung der Psychosen völlig ersetzen würden, wurden an der Wiener Klinik unter Hans Hoff nicht geteilt.[1018] Das galt auch ab 1958 für die neuen Antidepressiva. Andererseits bestand in Wien traditionell ein starkes psychopharmakologisches Interesse[1019] und die neuen Psychopharmaka wurden hier auch sehr früh getestet. Wiener Studien kamen bereits in der Testphase zu positiven Ergebnissen[1020] und die neuen Medikamente wurden sehr rasch in die Wiener ‚Gesamtbehandlungspläne‘ aufgenommen und manchmal alleine, manchmal in Kombination mit den ‚großen alten Kuren‘ angewandt.[1021]

In den Wiener Publikationen wurde die Idee der multifaktoriellen Kausalität der Krankheiten und des dynamischen Krankheitsprozesses vertreten, ein Konzept, das einen organischen, einen psychischen und einen sozialen Faktor in der Entstehung psychiatrischer Krankheiten annimmt. Die Therapie müsse folglich ebenso vielschichtig sein wie die Entstehungsfaktoren. Damit wurde gefordert, auch eine medikamentöse Therapie so individuell wie möglich zu gestalten. Trotz vieler Studien – auch aus der Wiener Klinik – fehlten bis etwa 1960 jedoch einheitliche Studienergebnisse und Erfahrungen über die Wirkung der neuen Medikamente bei bestimmten Symptomen bzw. Symptomgruppen (Syndromen), sodass die Forderung nach einer individuellen Therapie für die PatientInnen im Rahmen des multifaktoriellen Ansatzes

---

[1016] HIFT/HOFF, Die organische Therapie der Psychose (1958), S. 1048. Auch Nijdam, Chef der Psychiatrischen Klinik in Utrecht, schreibt, dass die Indikation zur Neuroleptika-Therapie nach „target symptoms“ gestellt wurde: NIJDAM, Die therapeutische Wirkung der Neuroleptika allein und in Kombination mit anderen Therapien (1960), S. 723.

[1017] SPIEL, Die Therapie in der Kinder- und Jugendpsychiatrie (1967), S. 118: „eine gewisse Gefahr […], nämlich von der ätiologischen Denkweise und Therapie sich abzuwenden und wieder einer Symptombehandlung zu huldigen.“ Vgl. ebd., S. 118-129 die Ausführungen zu den Psychopharmaka.

[1018] Vgl. unten S. 277 zu Hoffs Pariser Vortrag von 1955, sowie S. 299 das Zitat aus ARNOLD/HOFF, Neuroleptika, Tranquilizer und Antidepressiva (1962).

[1019] Vgl. dazu SPRINGER, Pschopharmakologische Forschung und Behandlung an der Wiener Psychiatrischen Universitätsklinik und die Frühphase des Collegium Internationale Neuro-Psychopharmacologicum (CINP) (2016), passim

[1020] Vgl. unten S. 273f. zu den Studien von 1952/53 zum Largactil und S. 253f. 1958 zum Tofranil.

[1021] Vgl. ARNOLD/HOFF, Neuroleptika, Tranquilizer und Antidepressiva (1962), S. 42f. zur Kombination von EKT und Tofranil (vgl. oben S. 253f.), und zum Versuch kombinierter, paralleler Malaria-Neuroleptika-Kuren, von denen jedoch ebenso wie von Antabus-Neuroleptika-Kuren abgeraten wird.

in Bezug auf die medikamentöse Therapie nur beschränkt umzusetzen war. Wenn etwa damals nach möglichst guten „Breitband-Neuroleptika" gesucht wurde,[1022] so ist das wohl in Zusammenhang damit zu sehen, dass Neuroleptika, die gleich auf Plus- oder Minus-Symptome[1023] wirken, erst in den darauffolgenden Jahren und Jahrzehnten entwickelt wurden. Der Mangel an spezifisch auf psychopathologische Zielsymptome (target symptoms) wirksamen Medikamenten bzw. an praktischer Erfahrung mit ihnen änderte sich jedoch zusehends, was sich etwa 1962 in einer zusammenfassenden Publikation von Ottokar H. Arnold und Hans Hoff zu „Neuroleptika, Tranquilizer und Antidepressiva" zeigt, in der eine vielfältige Anwendung der Psychopharmaka beschrieben wird.[1024]

2.4.1 Neuroleptika

Als erstes Neuroleptikum ging Chlorpromazin in die Geschichte ein, dessen psychische, psychiatrisch nützliche Wirkung als Zufallsbefund vom französischen Marinechirurgen Henri Laborit erstbeschrieben und von den Psychiatern Jean Delay und Pierre Deniker vom Hôpital Sainte-Anne in Paris aufgegriffen und in ‚der klinische[n] Psychiatrie eingeführt' wurde.[1025] Schon 1952, und damit an einer der ersten Kliniken weltweit, konnte an der Wiener Klinik eine Studie zum Chlorpromazin durchgeführt werden.[1026] Das Präparat wurde vom Pharmaunternehmen *Specia* in Paris hergestellt[1027] und erhielt den Testnamen „4560 RP", weshalb sich auch in den späteren Patientenakten der Klinik häufig die Bezeichnung „4560" statt Largactil (späterer Handelsname) finden lässt. Wie ein Bericht der Wiener Kliniker Arnold, Hift und Solms aus dem Sommer 1953 zeigt,

---

[1022] ARNOLD, Klinische Erfahrungen mit dem Neuroleptikum Truxal (1959), S. 898: „Es zeigt sich, daß Truxal derzeit das beste ‚Breitbandneuroleptikum' darstellt, indem es sich ohne schwere Nebeneffekte bei nahezu allen psychiatrischen Erkrankungen und Altersstufen zur Bekämpfung der angeführten akuten Symptome rasch wirksam anwenden lässt." Verweist dann auf die Vorteile gegenüber Chlorpromazin. Vgl. unten S. 279f.

[1023] „Plussymptomatik, Symptome der Schizophrenie und anderer Psychosen, wie Wahn, Halluzinationen, Angst und Erregung. Sie werden auch produktive Symptome genannt. Das Gegenteil sind die Minussymptome, die sich durch sozialen Rückzug, Apathie und Verflachung im Affekt zeigen" (17.4.2023 https://www.google.com/search?client=firefox-b-d&q=plus+minus+symptome+schizophrenie).

[1024] ARNOLD/HOFF, Neuroleptika, Tranquilizer und Antidepressiva (1962), passim.

[1025] Vgl. unten S. 277 das Zitat aus HOFF, Advantages and Disadvantages of Treatment with Chlorpromazine and Serpasil (1956), S. 52f.

[1026] Vgl. zu dieser Studie die beiden Publikationen von ARNOLD/HIFT/SOLMS, Über die Anwendung eines zentralvegetativen Hemmungsstoffes in der Psychiatrie (1952), S. 48; ARNOLD/HIFT/SOLMS, Die Anwendung von Largactil in der Psychiatrie (1953), S. 563-566. GRÖGER/KASPER, Zur Dominanz der organisch-biologischen Auffassung in der Psychiatrie der Wiener medizinischen Schule und den Anfängen der Psychopharmakotherapie (1997), S. 17 schreiben in Bezug auf diese frühen Studien: „Die Bedeutung der Psychopharmaka wurde klar erkannt und die Auseinandersetzung mit ihnen setzte früh ein."

[1027] Vgl. zur Geschichte, Testung und Diskussion von Megaphen, dem Chlorpromazinpräparat von Bayer (1953), umfassend BALZ, Zwischen Wirkung und Erfahrung – eine Geschichte der Psychopharmaka (2010), S. 123-308.

galt das Interesse der Psychiater weniger der antipsychotischen Wirkung des Medikaments, als seiner „schlafmachende[n]“, „beruhigende[n] Wirkung“. Dieses Interesse argumentierten sie damit, dass längere Anwendungen von Scopolamin und Morphin zur Beruhigung oder von Barbituriaten als Schlafmittel den Organismus schwer schädigen würden und die Dauerschlafbehandlung sich wegen ihrer „allzu großen Gefährlichkeit und der komplizierten pflegerischen Maßnahmen […] nicht einbürgern“ habe können.[1028] Largactil hingegen könne ohne organische Schädigungen über Wochen verabreicht werden.[1029]

Eine spezielle Indikation, zum Beispiel für Psychosen oder Erkrankungen des schizophrenen Formenkreises[1030], wurde nicht explizit gemacht, vielmehr wurde auf die symptomatische Anwendung zur Beruhigung erregter PatientInnen und auf Erfolge im Bereich affektiver Erkrankungen hingewiesen. So zeigte sich die sehr positive Einstellung der Wiener Kliniker gegenüber dem Neuroleptikum auch in ihren Ausführungen in Bezug auf die Anwendung gemeinsam mit dem bzw. alternativ zum Elektroschock: Bei der Manie, schrieben sie 1953, seien ‚Elektroschock‘ und Largactil gleichwertig,[1031] bei Largactil seien jedoch weniger unerwünschte Wirkungen feststellbar (eben kein amnestisches Syndrom); bei depressiven Störungen, in denen der Elektroschock keine Wirkung mehr zeige, seien mit Largactil zumindest symptomatische Erfolge erzielt worden; bei der akuten Katatonie und dem Status epilepticus sei der Elektroschock jedoch unentbehrlich. (In späteren Publikationen wird

[1028] Die Neuroleptika wurden schließlich auch bei „Dämmerkuren“ und statt dem Barbiturat Somnifen bei „Dauerschlafkuren“ eingesetzt: Vgl. BLEULER, Lehrbuch der Psychiatrie ([11]1969), S. 163. Zum einzigen Fall mit Somnifen in den bearbeiteten Wiener Akten vgl. S. 254 den Patienten S4636.

[1029] ARNOLD/HIFT/SOLMS, Die Anwendung von Largactil in der Psychiatrie (1953), S. 563f. Hier wird ausführlich auf die akute sowie auf die mehrwöchige Anwendung von Largactil zur Beruhigung und zum Dämmerschlaf eingegangen, sowie auf die Nebenwirkungen und medikamentösen Kombinationen. Auch drei Jahre später geht es in der Beschreibung der Anwendung in HOFF, Lehrbuch der Psychiatrie (1956), S. 408-411 um Beruhigung und Dämmerschlaf, mit deutlich höherer Dosis und Vorschlägen zu medikamentösen Kombinationen bei der Gewöhnung an Largactil. Die Dauer der Largactilkur gab Hoff hier mit nicht weniger als 3 Wochen und manchmal 8 bis 10 Wochen an. 2 bis 3 Wochen nach Beginn der Kur soll die Arbeitstherapie einsetzen. Da Largactil im Gegensatz zur EKT kein amnestisches Syndrom hervorruft, habe die Kur den Vorteil, den psychotherapeutischen Kontakt mit dem Patienten nicht zu unterbrechen. Falls in Fällen von endogenen Depressionen die EKT nicht zum Ziel führe, „kann unter Largactil eine Sozialisierung der Patienten erzielt werden. Eine lang dauernde orale Medikation bewährt sich hier insbesondere als eine Basis für Psychotherapie.“

[1030] Auch hier geht es nur um „die schizophrenen Erregungszustände“: sie „können durch eine Largactilkur gedämpft und ein gestörter schizophrener Gleichgewichtszustand wiederhergestellt werden, die Grundkrankheit bleibt unbeeinflußt.“

[1031] „Die Erfolge bei der Manie sind nicht so durchschlagend wie bei den posttraumatischen Verwirrtheitszuständen. Sie scheinen diejenigen der Elektroschockbehandlung – in Serien von fünf Schocks an einem Tag – nicht zu übersteigen, aber auch nicht dahinter zurückzubleiben. Dabei hat die Largactilkur den wesentlichen Vorteil, ohne amnestisches Syndrom einherzugehen, so daß es zu keinem auch nur zeitweisen Abbau der geistigen Fähigkeiten des Patienten kommt und auch der psychotherapeutische Kontakt aufrechterhalten werden kann.“ Die Studie bezieht sich auf Erfahrungen mit 66, diagnostisch sehr heterogenen Fällen – was auch von den Autoren in Bezug auf die Vorläufigkeit der Ergebnisse betont wird.

betont, dass bei der Katatonie die Anwendung der Neuroleptika kontraindiziert sei[1032]). Largactil wird auch „als symptomatische Therapie […] zur Beruhigung von Verwirrtheits- und Erregungszuständen jeder Art“, bei Suchtmittelentziehungskuren, „als Unterstützung der Psychotherapie bei Neurosen, deren hauptsächliches Symptom, die Angst, die Gewinnung eines Kontaktes erschwert“, „bei einem hysterischen Ausnahmezustand mit Faxensyndrom“, bei „schizophrenen Erregungszuständen“ als sehr erfolgversprechend von Arnold, Hift und Solms beschrieben.[1033]

Im Oktober 1955 fand ein großes ‚internationales Kolloquium zu Chlorpromazin und den neuroleptischen Medikamenten in der psychiatrischen Therapie‘ unter der Leitung von Jean Delay in Paris statt.[1034] Hans Hoff hielt einen Vortrag zu „Advantages and Disadvantages of Treatment with Chlorpromazine and Serpasil“, in dem er darauf hinweist, dass Largactil (im Versuchsstadium 4560 RP) im August 1952 an der Wiener Klinik eingeführt und seither in 1.000 Fällen angewandt wurde, und dass die ersten Wiener Studien dazu bereits im November 1952 und Anfang 1953 publiziert wurden.[1035] Zum zweiten Medikament seines Vortrags betonte er, dass – ermutigt durch die Berichte über ausgezeichnete Erfolge mit Serpasil / Reserpin in fast allen psychiatrischen Gebieten seit 1953 – diese Substanzen seit ca. eineinhalb Jahren (also seit Anfang 1954) in mehr als 200 Fällen angewandt wurden.[1036] Nachdem er die beobachteten Wirkungen und unerwünschten Nebenwirkungen von Largactil

---

[1032] Vgl. ARNOLD/HOFF, Neuroleptika, Tranquilizer und Antidepressiva (1962), S. 40f.: „Die Gabe beliebiger Neroleptika macht die Elektroschockbehandlung in keinem Fall unmöglich. Die einzige Ausnahme stellt das Zustandsbild der akuten bedrohlichen Katatonie dar, bei dem der Koordinationszerfall zentraler vegetativer Regulationen als Krankheitssymptom durch die Gabe von Neuroleptika verstärkt wird.“ Vgl. HIFT/HOFF, Die organische Therapie der Psychose (1958), S. 1048: bei der akuten bedrohlichen Katatonie würden Neuroleptika negativ wirken.

[1033] ARNOLD/HIFT/SOLMS, Die Anwendung von Largactil in der Psychiatrie (1953), S. 565f. BLEULER, Lehrbuch der Psychiatrie ([10]1960), S. 470, betont in Zusammenhang mit der Behandlung von Zwangsneurosen, allgemein: „In Zuständen von starker, angsterfüllter Spannung und Erregung können körperliche Kuren mildern und bessern. Bisher versuchte man vor allem Insulin- und Schlafkuren [bei Bleuler heißt das Dauerschlafkuren,GH], heute sind Kuren mit Chlorpromazin oder ähnlichen Präparaten zuerst anzuwenden.“ Vgl. zur Suchtbehandlung mit Largactil den Fall der 1954 68jährige Patientin X000 (nicht in der Datenbank) mit der Diagnose „chronische Coxitis, Schmerzmittelmissbrauch [Heptadon]“; Eintrag in ihrer Fieberkurve: „6stdl [bzw. 8stdl] 45-60 i. m.“, im Decursus zum gleichen Datum: „sechsstündig Largactyl“ und „unter Largactyl ruhig, aber deprimiert“; später, zusammenfassend für Versicherung: „Zunächst Largactilbehandlung, erst 6stündig, dann 8stündig. Allmählich Übergang auf Phenergan [lt. Fieberkurve bis zur Entlassung 8stdl 1A, dann 1Tbl. „45-60“, also Largactil, und nur am Abend 1A, dann 1Tbl. Phenergan] und intensive physikalische Therapie gegen die Schmerzen (Hochfrequenz, Vierzellenbäder, Zwischenhirndiathermie).“

[1034] Vgl. den umfangreichen Sammelband des „Colloque international sur la chlorpromazine et les médicaments neuroleptiques en thérapeutique psychiatrique“, Paris 21.-23. Oktober 1955, hrsg. von Jean DELAY.

[1035] Vgl. die oben S. 274 in Anm. 1025 zitierten beiden Studien von ARNOLD/HIFT/SOLMS von 1952 und 1953.

[1036] HOF, Advantages and Disadvantages of Treatment with Chlorpromazine and Serpasil (1956), S. 52f.

und Serpasil beschrieben hatte, ging er auf die bisherigen Erfahrungen mit ihrer therapeutische Anwendung und teilweise auf seine Vorstellungen zum Wirkmechanismus ein:

Für die Indikation war ausschlaggeben, wie rasch und dauerhaft die beiden Medikamente beruhigend wirken sollten und welche unerwünschten Nebenwirkungen sie hatten. So war Largactil als schneller wirksam als Serpasil bei manischen Zuständen die Methode der Wahl. Es sei nicht klar, ob das Medikament den manischen Zustand beeinflusse, jedenfalls würden die PatientInnen ruhig und nach einem kurzen Schlaf würden sie zur Arbeitstherapie gehen. Sie hätten noch „manic ideas", die sie jedoch nur in der Analyse ausdrücken würden. Der Nachteil von Largactil sei der Gewöhnungseffekt,[1037] die Beruhigungstherapie könne jedoch durch das langsamer wirkende Serpasil fortgeführt werden.

Bei melancholischen Zuständen scheine Largactil in Einzelfällen zu Remissionen zu führen. Den PatientInnen würden durch beide Medikamente ihre Angst genommen und Hoff führte hier als Beispiel an, dass sie so auch ihre Angst vor dem ‚Elektroschock' verlieren würden und es nicht mehr notwendig sei, sie zu dieser Behandlung zu zwingen.

Zur Behandlung schizophrener Erkrankungen schrieb Hoff: Largactil habe bei der beginnenden Schizophrenie eine beruhigende Wirkung, könne jedoch nicht die ‚Schockbehandlung' und die Psychotherapie ersetzen; Serpasil sei wegen seiner langsamen Wirkung nicht anzuwenden.

Bei akuten katatonen Zuständen würde an der Wiener Klinik weder Largactil noch Serpasil, sondern der ‚Schockblock' angewandt.

Bei chronischen Schizophrenien sei Largactil wegen seiner nachlassenden Wirkung schlechter als Serpasil, welches möglicherweise wegen seiner dem Serotonin konträren Wirkung auch eine spezifische Wirkung auf schizophrene Prozesse habe.

Hoff beurteilte im Vortrag von 1955 auch die Anwendung von Largactil und Serpasil bei Epilepsie, bei Alkoholkranken mit Delirium tremens oder anderen Psychosen,[1038] bei senilen

[1037] Vgl. HOFF, Lehrbuch der Psychiatrie (1956), S. 412-414, hier S. 414: „Im Hinblick auf die erwähnte Gewöhnung an Largactil bietet der Übergang auf Serpasil eine besonders günstige Bereicherung unserer Behandlungsmöglichkeiten." Vgl. GHERARDUCCI, A propos de l'utilisation de la chlorpromazine associée a d'autres thérapeutiques psychiatriques (1956), S. 888f., mit mehreren Kombinationsvorschlägen zu Largactil, die deutlich die Versuchsphase des Neuroleptikums zeigen.

[1038] Bei der Anwendung von Largactil bei Delirium tremens und anderen alkoholischen Psychosen hatten die Wiener Kliniker zwei Todesfälle und bevorzugten deshalb Paraldehyd; andere Forscher berichten jedoch von Erfolgen. Serpasil habe keine Wirkung.

und arteriosklerotischen Zuständen, bei Verwirrungszuständen nach Hirnverletzungen, im Entzug, bei Neurosen,[1039] bei extrapyramidalen Hyperkinesien,[1040] sowie zu Schlafkuren.

Abschließend betonte Hoff, dass Largactil und Serpasil für ihn keine neue Ära in der Psychiatrie eröffnen würden, wie andere Vortragende meinten, die die ‚alten' Kuren durch die Neuroleptika ersetzen wollten. Für Hoff erforderten die verschiedenen Faktoren, die zu Psychosen und Neurosen führen, eine Behandlung mit einem oder mit einer Kombination der alten und der neuen Mittel.[1041] Beide Medikamente seien wichtig in der Therapie der Psychosen, da sie zur Linderung der Symptome beitragen, und durch diese symptomatische Behandlung, durch das Verschwinden des quälenden Symptoms den Patienten befähigen würden, „to mobilize forces in himself which will lead to his recovery."

Hoff endete seinen Vortrag mit einem Lob der französischen psychiatrischen Schule, wobei er die den klinischen Alltag und das Leben der PatientInnen außerhalb der Klinik entscheidend verändernde beruhigende Wirkung der Neuroleptika hervorhob: „we owe this to the French Psychiatric School […] [to] the introduction of Largactil into clinical psychiatry by Delay and Deniker."

Hoffs Ausführungen im Vortrag von Oktober 1955 zeigen Korrekturen der ersten Ergebnisse von 1952/53 (etwa bei der Manie) und Ergänzung vor allem durch die Aufnahme von Serpasil (Reserpin) als neues Neuroleptikum. 1958 wurde in der bereits in den vorigen Kapiteln zitierten Studie von Hift und Hoff, in der die „drei Hauptbehandlungsmethoden", die

[1039] Largactil reduziere Angstzustände und ermögliche so eine Psychotherapie, die sonst nicht möglich wäre, und vermindere dadurch den Widerstand gegen andere Behandlungen. Es gäbe auch neurotische Reaktionen, die alleine mit Largactil und ohne Psychotherapie unter Kontrolle gebracht werden könnten.
[1040] Largactil sei nicht anzuwenden, sondern Serpasil, welches in Wien bisher in zwei Fällen, bei Chorea Huntington und Athetose double (erfolgreich) angewandt wurde
[1041] Vgl. die Anwendung zur Beruhigung und in Kombination, aber auch alleine im Beispiel der Patientin P11360, die bereits oben S. 129 Anm. 486 wegen ihrer 1. Einweisung aus dem Erziehungsheim Wiener Neudorf 1953 erwähnt wurde: 1951 hatte sie in Graz, nachdem sie zu Hause nichts gegessen und alles zusammengeschlagen habe, 3 Monate lang eine Insulin[sub?]komatherapie und danach eine EKT bekommen. 1953 war vom „beratenden Psychiater der Bundesanstalt für Erziehungsbedürftige, Dr. Erwin Ringel" die Diagnose „eine[r] schwerste[n] Psychopathie mit übermächtigen Aggressionstendenzen, die sowohl gegen die Umgebung als auch gegen die eigene Person gerichtet sind" gestellt worden. Mit der Diagnose „Psychopathie, Selbstbeschädigung" bekam sie an der Klinik EKT und mehrmals danach 10gr. Paraldehyd per Sonde, sowie bei der rasch folgenden 3. Aufnahme 1954 („Pseudologia phantastica bei Psychopathie") das Neuroleptikum Largactil: am 1. Tag um 20h 2 A Largactil, vom 2.-8. Tag (Rücküberstelung nach Gugging) um 6, 12, 18 und 24h je „2 Amp. 45/60 [Largactil]". Die Symptome des psychopathischen Syndroms – auch Bleuler nennt die „Pseudologia phantastica" unter den Symptomen der Psychopathie (zitiert oben S. 97 Anm. 339) – werden hier wieder ähnlich behandelt wie jene bei den anderen der 4 nicht-luetischen Diagnosen.

„Insulinkur, die Krampfbehandlung, die Behandlung mit Neuroleptika und Tranquilizern […] miteinander verglichen“ werden, die Anwendung von Chlorpromazin bei erregten Patienten als „bis heute […] unerreicht“ hervorgehoben. Hier wurde nun das ganz neue, erste moderne Antidepressivum Tofranil einbezogen, das im nächsten Unterkapitel behandelt wird. Gleichbleibend wie in den früheren Studien messen die Wiener Kliniker den neuen Psychopharmaka einen wichtigen Platz im „Gesamtbehandlungsplan“ zu, in dem jedoch die beiden „alten Kuren“ einen prominenten Platz behalten. Vom „Standpunkt“ der Wiener Psychiatrisch-Neurologischen Universitätsklinik wird hier die Indikation bei den verschiedenen Diagnosen aus dem Bereich der schizophrenen und der affektiven Erkrankungen beschrieben, die Wahl im Einzelfall einer der drei Methoden alleine oder in ihren Kombinationen, mit dem Schluß, „daß wir die Schockbehandlung nicht für überholt ansehen. Wir sind im Gegenteil der Meinung, daß ihre Leistungsfähigkeit durch genaue Indikationsstellung und verbesserte Technik in den letzten Jahren bedeutend zugenommen hat. Die Neuroleptika und die Tranquilizer erscheinen als sehr wertvolle Bereicherung unserer Therapie, ohne den Anspruch auf eine dramatische Revolutionierung der Behandlung erheben zu können.“[1042]

Die Bewegungsstörungen (das Parkinsonoid und die Dyskinesien) als unerwünschte Nebenwirkungen der Neuroleptika wurden breit diskutiert. Hift und Hoff warnten 1958 vor der „Ausbildung extrapyramidaler Syndrome, die bis zu dem Bild eines schweren Parkinsonsyndroms, ja bis zu extrapyramidalen Lähmungen der Schluck- und Kaumuskulatur führen“ können und betonten, dass diese Symptome „für eine unangenehme und mitunter gefährliche Komplikation und nicht für ein erwünschtes therapeutisches Ziel“ gehalten werden müssten.[1043] Während die Wiener Kliniker jedes Auftreten extrapyramidaler Symptome als Nebenerscheinung sahen, die sie vermeiden wollten, schätzten einzelne Psychiater[1044] sie als Zeichen des Erfolgs ein: So beispielsweise S. J.

[1042] HIFT/HOFF, Die organische Therapie der Psychose (1958), S. 1046-1148. Die Autoren verweisen auch bereits auf die Gefahren eines „unkontrollierten Massenverbrauchs“ der Psychopharmaka.
[1043] HIFT/HOFF, Die organische Therapie der Psychose (1958), S. 1046.
[1044] Vgl. HAASE, Über Vorkommen und Deutung des psychomotorischen Parkinsonsyndroms bei Megaphen- bzw. Largactil-Dauerbehandlung (1954), passim, bezeichnet die extrapyramidalen Nebenwirkungen als Indikator der Wirkweise der Neuroleptika als „neuroleptische Schwelle“ und erkannte als erster – so Paul Janssen – "die unabdingbare Gebundenheit der neuroleptischen Wirkung an das extrapyramidale System beim Menschen [...]. Die Ergebnisse der Untersuchungen Haases haben für die Dosierung der Neuroleptika eine entscheidende Bedeutung erlangt": Paul Janssen, Geleitworte zur Erstauflage von 1966, zitiert aus der 2. Auflage: HAASE, Therapie mit Psychopharmaka und anderen psychotropen Medikamenten (1969), S. 6. Haase hat als Instrument zur minutiösen Feststellung selbst geringer extrapyramidaler Störungen – eben an der Schwelle ihrer Entstehung

Nijdam, Leiter der psychiatrischen Universitätsklinik Utrecht, der postulierte, dass ein mildes Parkinsonoid für die Wirksamkeit des Neuroleptikums spräche, weshalb die Dosierung bis zum Auftreten der ersten bewegungsgehemmten (akinetischen) Symptome erfolgen sollte. Eine höhere Dosierung werde zwar nicht angestrebt, bleibe aber dennoch harmlos, da selbst ein „ausgesprochenes Parkinsonoid" problemlos mit Anti-Parkinsonmittel zu therapieren sei.[1045] Häufig wurden diese Nebenerscheinungen als weniger gefährlich eingeschätzt als in Wien, so durch die Innsbrucker Kliniker F. Jost und K. Katzelberger, da die Symptome durch die Gabe von Atropin oder durch das Absetzen von Reserpin „immer gänzlich" verschwinden würden. Die beiden Autoren schrieben zugleich, dass sie Reserpin „vornehmlich zur akuten und aktuellen Symptomatik" einsetzen würden[1046] – ihre positive Erfahrung kann deshalb wohl auf die kurze Dauer der akuten Therapie zurückgeführt werden.

Die Einschätzung, dass alle extrapyramidalen Störungen, einschließlich der sogenannten „Spätdyskinesien"[1047], wieder bis zur *restitutio ad integrum* abklingen würden, war verbreitet und mag nicht zuletzt dazu geführt haben, dass Neuroleptika oft zu spät abgesetzt wurden und dass es daher in der Frühzeit der Neuroleptika-Ära häufiger zu den bekannten „Entstellungen der Patienten" (Spätdyskinesien) kam. Gleichzeitig gilt zu erwähnen, dass das Risiko stark abhängig von der Dosis, der Einnahmedauer und dem Neuroleptikum war und dass die ersten – der nicht zwingend auftretenden – Symptome (Frühdyskinesien) für gewöhnlich verschwanden. Dass die Klinik Hoff stärker vor den Nebenwirkungen warnte als andernorts, zeigt, dass hier eine andere Risikobewertung vorgenommen wurde.

1959 wurde auch in Wien von Ottokar H. Arnold eine Studie mit Truxal (Chlorprothixen) durchgeführt, das im selben Jahr auf den Markt gekommen war: Laut dieser Studie habe sich

– die von Kraepelin erfundene 'Schreibwaage' und insgesamt die Beurteilung der Handschrift eingesetzt (ebd., S. 114-133).

[1045] NIJDAM, Die therapeutische Wirkung der Neuroleptika allein und in Kombination mit anderen Therapien (1960), S. 723.

[1046] JOST/KATZELBERGER 1958: Zu den Reserpin-Kuren in der klinischen Praxis der Psychiatrie (1958), S. 155.

[1047] Spätdyskinesien sind unwillkürliche Bewegungen, die meist stereotyp und repetitiv vorwiegend im Gesicht auftreten. Die typischen „Grimassen" wie Kau- und Schmatzbewegungen können ein Leben lang bestehen bleiben und beeinträchtigen das Sozialleben der Patienten beträchtlich. Das heutige Pharmazie-Lehrbuch von AKTORIES/FÖRSTERMANN/HOFMANN/STARKE, Allgemeine und spezielle Pharmakologie und Toxikologie ([10]2009), S. 315 gibt an, dass bei einer dauerhaften Einnahme (über mehrere Monate) von klassischen Neuroleptika etwa 20 Prozent der PatientInnen unter Spätdyskinesien leiden. Die unter Umständen bereits nach der ersten Dosis auftretenden Früh- oder akuten Dyskinesien, das pharmakogene Parkionsonsyndrom (das eine gewisse Zeit der Einnahme des Medikaments voraussetzt) und die die Einnahme des Wirkstoffes überdauernden Spätdyskinesien (die allenfalls bei einer Langzeiteinnahme auftreten) sind keine zwingend auftretenden unerwünschten Wirkungen. -> kann evtl. gelöscht werden, weil Sie das ja in Beispielen schon im Fließtext verdeutlichen

Truxal als ideales Medikament zur Beruhigung herausgestellt, da es intramuskulär injiziert werden konnte und schon kurz nach der Applikation in der Lage sei, jeden Erregten zu beruhigen oder gar in den Schlaf zu versetzen. Bei Langzeitbehandlung – so die Wiener Ergebnisse – würde überdies kein Gewöhnungseffekt stattfinden, im Gegensatz zum Largactil (Chlorpromazin), bei dem der stark ausgeprägte Gewöhnungseffekt immer wieder Therapieänderungen notwendig mache. Bei 88 Prozent der PatientInnen habe man keine Nebenwirkungen festgestellt. Insgesamt wäre Truxal unter den damals verfügbaren „Breitbandneuroleptika" das Beste.[1048] Auch Heinrich Gross, der trotz seiner NS-Euthanasie-Vergangenheit[1049] 1957 Primarius am *Steinhof* geworden war und sich in den 1960er Jahren besonders intensiv mit der Testung von Psychopharmaka befasste, ist 1960 der Ansicht, dass sich das Chlorprothixen (Truxal) gegenüber den bereits in klinischer Verwendung stehenden Neuroleptika als überlegen erwiesen habe. Der antipsychotische Effekt hingegen sei zu gering, weshalb das Mittel am *Steinhof* vor allem als Basisneuroleptikum während der ersten drei Aufenthaltstage zur initialen Dämpfung eingesetzt würde, um dann auf ein effektiveres Neuroleptikum umzustellen.[1050] Auch an der Klinik Hoff war Truxal in den 1960er Jahren eines der am meisten verabreichten Medikamente.

Eberhard Gabriel, der 1965/66 an der Psychiatrie in Zürich und ab 1966 an der Wiener Klinik (Frauenabteilung) tätig war, nennt Truxal als stark sedierend wirkendes Neuroleptikum und Saroten (Amitryptilin) sowie Tryptizol als sedierend wirkende Antidepressiva als jene Medikamente, die in den ersten Jahren seiner Tätigkeit an der Wiener Klinik – abhängig von der Krankheit – für eine Dämmerkur als Behandlungseinleitung verwendet worden sind. In Zürich sei hingegen 1966 noch das Morphin-Scopolamin[1051] für die Dämmerkur und für die Einleitung einer [Dauer-]Schlafkur[1052] eingesetzt worden.[1053] Anhand der Wiener Akten lässt

---

[1048] ARNOLD, Klinische Erfahrungen mit dem Neuroleptikum Truxal (1959), S. 897f.
[1049] Vgl. den Wikipedia-Eintrag und die dort zitierte Literatur (22.4.2023: https://de.wikipedia.org/wiki/Heinrich_Gross).
[1050] GROSS, Erfahrungen mit neuroleptischen Behandlungsverfahren (1960), S. 718. Zu Erfahrungen in Graz mit ähnlichen Ergebnissen, EICHHORN, Die gegenwärtige Situation bei der klinischen Behandlung von Depressionen (1960), S. 224f., zitiert unten S. 292 Anm. 1109.
[1051] Vgl. BLEULER, Lehrbuch der Psychiatrie ([10]1960), S. 152 und S. 166 zur Anwendung von Scopolamin (Hyoscin) als „Mittel der Wahl zur raschen (vorübergehenden) Ruhigstellung bei tobsüchtigen Zuständen", in der Wirkung zu verbessern mit Morphin.
[1052] Gabriel hat diese in Wien nicht erlebte. Lt. Gabriel wurde in Wien in der 2. Hälfte der 1960er Jahre „Antidepressiva- bzw. Neuroleptika-Kuren mit dämpfend wirkenden Substanzen unter dem Jargontitel ‚Schlafkur' (ungleich dem Klaesi-Begriff von Schlafkur) fast stereotyp eingeleitet." Es waren (nach Züricher Diktion) ‚Dämmerkuren', bei denen der / die Patient/in jederzeit aufgeweckt werden konnte.
[1053] E-mail Eberhard Gabriel an Gernot Heiss, 17.2.2015. Zur Schlafkur oder „Dauernarkose" von Klaesi vgl. oben S. 224 Anm. 877.

sich nachvollziehen, dass Truxal ab 1960 immer häufiger verwendet und Largactil langsam verdrängt wurde. Niedrigpotente Neuroleptika (ab 1960 vermehrt Truxal, zuvor eher Largactil) wurden zudem häufig in Kombination mit der Elektrokrampftherapie verabreicht. Vor Beginn einer Elektrokrampfbehandlung wurden Neuroleptika oft Tage bis einige Wochen lang gegeben.[1054]

Ein ebenfalls häufig appliziertes Neuroleptikum war Reserpin, das in natura in der Rauwolfia (indische Schlangenwurzel) enthalten ist und an der Klinik Hoff hauptsächlich in Form des Präparats Serpasil[1055] verabreicht wurde. Eine bekannte unerwünschte Wirkung des Reserpin liegt in seiner depressiogenen Wirkung, weshalb es niemals bei Psychosen mit einer depressiven Komponente eingesetzt werden sollte, sich aber umgekehrt als Indikation bei Psychosen im Rahmen einer Manie eignete.[1056] Laut den Innsbrucker Klinikern F. Jost und K. Katzelberger würden zwar die psychiatrischen Symptome nicht verschwinden, da jedoch Reserpin die emotionalen Spannungen löse, hätten die PatientInnen die Möglichkeit, sich von ihrer Krankheit zu distanzieren, d.h., dass zwar immer noch Ängste und Depressionen feststellbar seien, die PatientInnen sich jedoch ihres eigenen Zustands besser bewusst würden und auf diese Weise ein Stück weit Kontrolle über ihr Affektleben zurückgewinnen könnten.[1057] Für die Indikation einer hochdosierten Reserpinkur als Ersatz der Insulinkomatherapie auch bei Schizophrenen setzte sich Heinz Häfner ab 1958 an der Heidelberger Klinik ein.[1058]

Auf den Erwachsenenabteilungen und sehr häufig auf der kinderpsychiatrischen Station der Wiener Klinik wurde Thioridazin verwendet. Dieses konnte nur oral appliziert werden, wobei es bei Erwachsenen in Form von Tabletten (Handelsname: Melleril) und bei Kinder auch als

---

[1054] Vgl. dazu HOF, Advantages and Disadvantages of Treatment with Chlorpromazine and Serpasil (1956), S. 56, dass mit der Beruhigung durch die beiden Medikamente (Largactil und Serpasil) auch die Angst der PatientInnen vor der Behandlung verringert werde.

[1055] Vgl. oben S. 275-277 die Zitate aus Hoffs Vortrag in Paris 1955 (publiziert 1956).

[1056] Hoff-Skriptum, Allgemeine Psychiatrie [um 1961], S. 66.

[1057] JOST/KATZELBERGER 1958: Zu den Reserpin-Kuren in der klinischen Praxis der Psychiatrie (1958), S. 155. Reserpin verschwand jedoch bald wieder aus den Akten.

[1058] E-mail an Gernot Heiss, 19.4.2014; vgl. u. a. HÄFNER/SCHLIACK/ZENZ, Die hochdosierte Reserpin-Kur in der Behandlung chronischer Schizophrener (1968), S. 226: Indikation eingeschränkt auf Fälle, „die wegen der Chronizität des Leidens oder der bestehenden Defektsymptomatik bei der Behandlung mit weniger eingreifenden Methoden keine ausreichenden Erfolge versprechen. Im Vergleich mit der früher unter gleicher Indikation üblichen Insulin-Kur, an deren Stelle die hochdosierte Reserpin-Kur praktisch getreten ist, scheint das Risiko lebensgefährlicher oder zu Dauerschäden führender Komplikationen wesentlich niedriger zu liegen.“

niedrig dosierte Dragees und als Tropflösung (Handelsname: Melleretten)[1059] mit einer mittleren antipsychotischen und schlafsichernden Wirkung ohne bedeutendes Risiko eines Parkinsonsyndroms (aufgrund seines pharmakologischen Wirkprofils) verabreicht wurde.[1060] Laut Gross eignete es sich besonders gut für eine ambulante Dauerbehandlung,[1061] und auch nach Hoff (laut einer Vorlesungsmitschrift) habe man es zur Dauerbehandlung chronisch Schizophrener „gerne genommen".[1062]

Der große Vorteil der neuen Neuroleptika, so Hift und Hoff 1958, zeige sich vor allem in ihrer „dauerhaften Anwendung". Dadurch würde den PatientInnen die Möglichkeit gegeben, sich von ihren psychotischen Symptomen zu distanzieren. Mit „dauerhaft" dürfte hier eine Anwendung über die akute Verwendung zur Unterdrückung von Symptomen hinaus zur Verhinderung von deren Wiederauftreten zu verstehen sein und nicht eine Fortsetzung nach dem Klinikaufenthalt.[1063] Hift und Hoff betonten, dass die Neuroleptika rein symptomatisch und nicht kausal wirken, weshalb beobachtete Verbesserungen ein „Anpassungsphänomen", nicht jedoch ein „Heilungsphänomen" darstellen würden. Der Insulinschock sei hingegen – so die Meinung der beiden Wiener Psychiater 1958 – in der Lage, die Schizophrenie auch kausal zu heilen.[1064]

Die weiterhin positive Einschätzung der ‚alten Kuren' und die höhere Bewertung der Gefahren der Nebenwirkungen der Psychopharmaka dürften dazu beigetragen haben, dass an der ‚Klinik Hoff' im Vergleich mehr ‚Schockkuren' und weniger Neuroleptika appliziert

---

[1059] Vgl. zur Anwendung in der Kinderpsychiatrie MÜLLER-KÜPPERS, Die Therapie im Kindes- und Jugendalter (1972), S. 991.
[1060] Für Ergänzungen ist Eberhard Gabriel zu danken.
[1061] GROSS, Erfahrungen mit neuroleptischen Behandlungsverfahren (1960), S. 719.
[1062] Hoff-Skriptum, Allgemeine Psychiatrie [um 1961], S. 66. Vgl. CZERWENKA-WENKSTETTEN/HOFMANN, Klinische Erfolge mit Melleril-retard (1966), S. 846: „1. Eine überzeugende Wirkung zeigt Melleril retard bei der Stabilisierung schizophrener Psychosen, besonders wenn akustische Halluzinationen, Angst oder Wahnphänomene bzw. Denkstörungen oder instabile Affektivität im Vordergrund stehen. 2. […] Amplitudenminderung zykloider Phasen […]. 3. […] bei der Nachbehandlung von Legierungspsychosen manischer wie depressiver Prägung. 4. In Fällen von Involutionsdepressionen haben wir mit Melleril-retard vielfach bessere Resultate erzielt als mit Antidepressiva allein (Kombinationstherapie). 5. […] jene inveterierten Neurosen […], die für eine Psychotherapie nicht in Frage kommen. […]. 6. Bei ambulanten Patienten […] günstige Beeinflussung anakastischer Syndrome und von Phantomschmerzen."
[1063] Klärender Hinweis von Eberhard Gabriel.
[1064] Vgl. ausführlicher aus HIFT/HOFF, Die organische Therapie der Psychose (1958), S. 1047-1048 oben S. 189f. und Hoff an das Nobelkomitee 1957, zit. oben S. 175. Nach BLEULER, Lehrbuch der Psychiatrie ([10]1960), S. 403-405, war hingegen den „‚großen', kurmäßig durchgeführten Behandlungsverfahren in der Psychiatrie" mit der „Ausnahme: Fieberkur bei progressiver Paralyse" „eine spezifische oder kausale Wirksamkeit für die eine oder andere psychisch Störung" nach heutigem Wissensstand „abzusprechen", sie würden jedoch „symptomatisch" wirken. Vgl. die Zitate oben S. 28-30.

wurden als an anderen, auch Wiener Spitälern. So schrieb Gross 1959, dass nun 80 Prozent der schizophrenen PatientInnen am *Steinhof* einer neuroleptischen Kur unterzogen würden.[1065] An der Klinik hingegen befanden sich unter den 525 Fällen der Stichprobe der Pilotstudie (1955-1960)[1066] im Zeitraum 1958-1960 insgesamt 29 schizophren erkrankte PatientInnen, von denen 17 PatientInnen Neuroleptika (≈ 58,6 %) erhielten.[1067] Aber auch an der Klinik nahm die Neuroleptika-Behandlung gegenüber den ‚alten Kuren' zu – das legen die Publikationen von Hoff, Arnold und Hofmann 1962/63 und der Rückgang der Insulinkoma- und der Elektrokrampftherapie in der Praxis der Klinik laut PatientInnenakten nahe.

Hans Hoff und Gustav Hofmann schrieben 1963, dass die „Elektrobehandlung, [der] Insulinschock und sonstige biologische Behandlungsmethoden, sowie die Psychotherapie durch die Einführung der Neuroleptika [nicht] obsolet geworden" seien, und dass auch andernorts nun diese älteren, dort bereits vergessenen Behandlungsverfahren wieder „in das therapeutische Schema" einbezogen" würden.[1068] Als Argument für die Anwendung der Neuroleptika wurden hier vor allem die Verbesserungen im „Milieu" angeführt: Die „Ära der N[euro]l[eptika]" hätte „das Milieu der psychiatrischen Spitäler gewandelt".[1069] Durch die dämpfende Wirkung der Neuroleptika bei akuten Psychosen seien die PatientInnen viel früher als ohne Neuroleptika für einen „therapeutischen Eingriff im Sinne einer Psychotherapie teilweise zugänglich" und es komme auch früher „zu einer beginnenden Gruppenbildung im sozialen Milieu des Krankensaales".[1070] Es ging also wieder vor allem um die beruhigende Wirkung der Neuroleptika, die eine soziale Anpassung und weitere Behandlung der PatientInnen erleichtern würde.

Wie sah nun ein solches Vorgehen an der Klinik Hoff aus? Dazu wieder das Beispiel der Behandlung schizophrener Erkrankungen: Wurde in den 1960er Jahren ein Patient am Beginn einer schizophrenen Episode eingeliefert, wurde zuallererst ein ‚Breitbandneuroleptikum'

[1065] GROSS, Erfahrungen mit neuroleptischen Behandlungsverfahren (1960), S. 718.
[1066] Dazu oben S. 21: in den 525 Fällen der Stichprobe wurden zusätzliche Daten aufgenommen, so auch die Psychopharmaka.
[1067] Für das Jahr 1959: 5 von 11 Patienten (≈ 45,5 %).
[1068] HOFF/HOFMANN, Die Anwendung der Neuroleptika in der psychiatrischen und allgemeinen Praxis (1963), S. 269.
[1069] Ebd. Letzteres hatte Hoff bereits in seinem Vortrag in Paris 1955 betont: zitiert oben S. 277.
[1070] HOFF/HOFMANN, Die Anwendung der Neuroleptika in der psychiatrischen und allgemeinen Praxis (1963), S. 271f.

gegeben.[1071] Ein solches besitze, so Hans Hoff und Gustav Hofmann, einen starken Dämpfungseffekt, der jedoch bei längerfristiger Applikation des Medikaments, meist nach einigen Wochen, verschwinden würde. Da Breitbandneuroleptika jedoch bei nahezu jeder Unterform der Schizophrenie dämpfend wirken würden, seien sie vor allem für die akute Therapie geeignet. In der Phase der Akuttherapie müsse zudem abgewogen werden, ob der Patient eine ‚Schockbehandlung' benötigt: So sei zum Beispiel die Elektrobehandlung bei einer Katatonie immer die Standardtherapie und bei einem jungen Patienten, bei dem erstmals eine schizophrene Phase ausgebrochen war, sei eine Insulinkomatherapie in Erwägung zu ziehen. Die Beruhigung des Patienten durch Breitbandneuroleptika führe zusätzlich zu einem wichtigen Zeitgewinn für die Differentialdiagnostik. Sobald die Akutphase überstanden war, wurden sogenannte ‚Langzeitneuroleptika' eingesetzt, die einen geringeren, aber dafür längerfristigen Dämpfungseffekt ausübten und zugleich weniger unerwünschte Nebenwirkungen als die Breitbandneuroleptika hatten. Während eine Dauertherapie mit einem Langzeitneuroleptikum versucht wurde, sollten die PatientInnen auch in eine Arbeits-, Gruppen- oder Psychotherapie eingeführt werden.[1072]

Oben im Kapitel zur Insulinkomatherapie wurde der „Gesamtbehandlungsplan" zu schizophrenen Erkrankungen wiedergegeben, den Arnold 1963 in seiner Monographie zur Schizophrenie und 1969 in einem Sammelband zu einer Tagung publizierte.[1073] Dieser dürfte für die Praxis der Klinik maßgeblich gewesen sein, auch wenn nicht alle KlinikerInnen der Meinung Arnolds gewesen sind. Hier wird der häufige Einsatz der Neuroleptika deutlich, auch wenn die Insulinkomatherapie für Arnold eindeutig die Basis der Behandlung bildete.[1074] Neuroleptika scheinen auf „zum Akutgebrauch", d. h. zur Behandlung von Symptomen wie starker Erregung (wobei sie bei primär blanden Prozessen auch den ‚Elektroschock' ersetzen konnten), aber auch in der „Übergangsphase" zur „Rehabilitierungsphase" ev. „zum Dauergebrauch". In den 1960er Jahren waren „bei Therapiebeginn nach dem Ende des 2.

[1071] Ebd., S. 271, schreiben sie, dass sie (also die Wiener Kliniker) den Begriff Breitbandneuroleptikum selbst geprägt haben. Zum Begriff vgl. oben S. 273 Anm 1022 und S 283f. Im Untersuchungszeitraum der Pilotstudie von 1955 – 1960 war es noch üblich, dass die Patienten erst mit Doriden/ Miltaun, also ohne Neuroleptika beruhigt wurden.

[1072] HOFF/HOFMANN, Die Anwendung der Neuroleptika in der psychiatrischen und allgemeinen Praxis (1963), S. 271f. Vgl. HOFMANN, Über Kriterien einer differenzierten Anwendungsweise von Neuroleptica und Antidepressiva in der klinischen Psychiatrie (1964), S. 144: „Breitbandneuroleptica mit starkem Dämpfungseffekt."; „Langzeitneuroleptica für den langen klinischen und ambulanten Kurgebrauch."

[1073] Vgl. die beiden Abbildungen oben S. 204f.

[1074] Vgl. Arnold oben S. 199 und 235.

Jahres“ der Krankheit EKT und „4-6 Majeptilkuren,[1075] dazwischen Arbeitstherapie, Gymnastik- und Sporttherapie, Gruppentherapie, evtl. Lobotomie“ vorgesehen. Die Neuroleptika, die in den 1960er Jahren an vielen Kliniken die Komatherapie verdrängt hatten, waren im „Gesamtbehandlungsplan“ aufgenommen, um den „Längsschnittverlauf schizophrener Psychosen und insbesondere die Rate der Resozialisierten“ positiv zu beeinflussen und da die Neuroleptika in ihrer unspezifischen(!) Wirkung „gezielte Hilfen“ darstellten.[1076]

Bedeutsam für den multifaktoriellen Ansatz der Klinik Hoff war, dass herausgearbeitet wurde, für welche Abwehrreaktionen welche Präparate besonders gut geeignet seien. Dazu eine Tabelle von Gustav Hofmann 1964, die eine Möglichkeit der Differentialdiagnose einzelner Substanzen suggeriert (und vermutlich weniger die klinische Routine als die Vorstellungen des Wiener Klinikers zeigt):

Anwendungsweise von Neuroleptica und Antidepressiva 147

Tabelle 2

Abwehrreaktion	Therapeutische Maßnahmen	Psychoseverlauf
Ausgliederung	Biologische Therapie	Phase, Schub
Isolierung	Majeptil	Prozeß („Hebephrenie“)
Primitivreaktion	Truxal	Exacerbation aus
Erregung		Prozeß oder aus
Hypochondrisch	Melleril	Defektniveau
Regression	Nozinan	
Zwangs-S.	Decentan	„Milieu“-Exacerbation
Wahnbildung	Randolectil	
Psychosomatisch	Haloperidol	
Konversion		
Phobie	Biobamat	Neurotische Abwehr
	Librium	(m. E.)
Fix. Persönlichkeits-	Psychotherapie	(Persönlichkeitsabwand-
abwandlung	Arbeitstherapie	lung)
		Labiler —
		stabiler Defekt

An dieser Tabelle[1077] von Hofmann aus dem Jahr 1964 sieht man zum Beispiel, dass PatientInnen, die sich im Rahmen einer Hebephrenie zunehmend isoliert hatten, am besten auf Majeptil (Thioproperazine) ansprechen würden; auf hypochondrische Störungen würde hingegen Melleril besser wirken, bei Regressionsbildungen Nozinan und so fort. An dieser

[1075] Vgl. Hoff-Skriptum, Allgemeine Psychiatrie [um 1961], S. 69, zu „prozeßhafter Verlauf (Hebephrenie!): Majeptil in gesteigerten Dosen, bis zum Krampfstadium […], dann absetzen und nach wenigen Wochen die Kur wiederholen. Insgesamt 5-6 Kuren.“

[1076] ARNOLD, Zum Stellenwert der Neuroleptikatherapie in den Behandlungsplänen der Schizophrenie (1969), S. 11-14.

[1077] HOFMANN, Über Kriterien einer differenzierten Anwendungsweise von Neuroleptica und Antidepressiva in der klinischen Psychiatrie (1964), S. 147.

Tabelle erkennt man auch, dass die „biologische Therapie“ im Behandlungsschema integriert war. Im Artikel spricht Hofmann aber nur von der Insulin- und von der Elektrotherapie, das heißt, die Malariakur, die etwa in den 1950er Jahren bei einer hebephrenen Schizophrenie häufig angewandt wurde, wird von Hofmann (1964) nicht mehr erwähnt.[1078]

Majeptil war trotz der für die PatientInnen sehr unangenehmen Krämpfe in Wien Anfang der 1960er Jahre in der Männerabteilung bei schwer beeinflussbaren Psychosen ein geschätztes Neuroleptikum:[1079] Für Arnold war 1963 – wie oben ausführlich zitiert – bei Jugendlichen mit Diagnosen, „die man früher unter dem Begriff der ‚Pfropfschizophrenie’ oder ‚Pfropfhebephrenie’ zusammengefaßt hat“, die Kombination der Malariafiebertherapie „mit anschließenden Majeptil-[Thioproperazin]kuren“ die „Methodik der Wahl“.[1080] Als Beispiel wird im Kapitel zur Malariafiebertherapie die Behandlung des 1963 19-jährigen Mittelschülers S655 mit der Diagnose „Schizophrenie (pri[märer] Prozess)“ beschrieben.[1081] Zur Anwendung von Neuroleptika bei spontanen Verläufen schrieb Arnold: „Unter zahlreichen Versuchen mit Neuroleptikaanwendung einen positiven Einfluß auf die spontanen Verläufe zu nehmen, hat sich bisher nur die Anwendung von Majeptil bzw. Haloperidol[1082] in Form einer speziellen Kur bewährt.“ Daraufhin schildert er ihre Indikation. Die Majeptiltherapie dauerte nach Arnolds Ausführungen im Durchschnitt vier bis fünf Wochen, während der über eine „Pseudoparkinsonphase“ die „Krampfphase“ zu erreichen sei; „der Effekt scheint bis zu einem gewissen Maß von der Krampfphase selbst abhängig. Sie bleibt daher immer das Ziel während der Kur. Die Krampfattacken selbst sollen so lange zu erhalten versucht werden, als es der Patient aushält“, der „versuchen muß, diese [möglichst lange] zu ertragen.“ Die mehrfache Wiederholung dieser Therapie nach Intervallen von zwei Wochen wurde „von vornherein für notwendig erachtet.“[1083] Ähnlich war für Hoff nach dem unautorisierten Skriptum von Anfang der 1960er Jahre Majeptil „das Mittel der Wahl bei

---

[1078] Vgl. oben S. 285.
[1079] Zur Anwendung von Majeptil publizierte Kryspin-Exner Anfang 1963 eine Studie, die auf eine 2 ½jährige „Erprobungszeit“ zurückgreift. Zusammenfassend schrieb er von einer „günstige[n] Wirkung der Verbindung bei prognostisch ungünstigen, sonst therapieresistenten schizophrenen Prozeßverläufen. In zirka einem Drittel dieser Fälle konnte durch mehrere Thioproperazinkuren eine völlige oder soziale Remission erreicht werden“: KRYSPIN-EXNER, Zur Behandlung schizophrener Prozeßpsychosen mit Thioproperazin (1963), S. 159f.
[1080] ARNOLD, Die Therapie der Schizophrenie (1963), S. 59-61, ausführlich vgl. oben im Kapitel zur Malariafiebertherapie S. 83.
[1081] Vgl. oben S. 84.
[1082] Vgl. oben S. 220 Anm. 860.
[1083] ARNOLD, Die Therapie der Schizophrenie (1963), S. 79-81. Die Krampfperiode wurde freilich nicht nur aufrechterhalten, solange der Patient die Schmerzen ertragen konnte, sondern auch nur „solange sich nicht sekundäre, bedrohliche Komplikationen anbahnen, wie z. B. schwere Krämpfe der Schlundmuskulatur oder gar Störungen der Atmung“.

Prozeßpsychosen“. Es unterbreche – so Hoffs Erklärung des Wirkmechanismus – „die Bahn Thallamus – extrapyramidales System und zurück. Die Impulse gelangen zum Stirnhirn, man zwingt den Pat. zu neuem corticalen Antrieb. Nachteil: Hyperkinesien […], Krampfattacken […], Torsionsattacken […]!! Die psychischen Erscheinungen werden besser, aber die Pat. leiden stark unter den körperlichen Nebenwirkungen!“[1084] Hoff empfahl demnach „insgesamt 5-6 Kuren“. Da das Ziel der Majeptilkur in der Behandlung „der Prozeßpsychose (Hebephrenie)“ „soziale Anpassung“ sei, müsse „auch Psycho- und Beschäftigungstherapie“ folgen. Falls die Schizophrenie schon älter als 1 ½ Jahre war empfahl Hoff nach dieser Mitschrift „nur Neuroleptica (Majeptil/Thioproperazine) und Arbeitstherapie.“

> Anfang 1964 bekam bei seinem ersten, 17tägigen Aufenthalt der 22jährige Patient S7509 mit der Diagnose „Pfropfschizophrenie“[1085] an den ersten 3 Tagen eine EKT mit 4 Anwendungen (2+1+1) und vom 2. bis zum 15. Tag Majeptil gesteigert von 1mal bis 11mal tgl.; bei den hohen Dosen wurden die Tabletten zu 10 mg ab 7h stündlich gegeben; dazu am Abend meistens 100 mg Sordinol (Neuroleptikum Clopenthixol). Am 6. Tag (bei „5x10 mg Majeptil per os“) hatte der Patient ab 13:30 leichte Krämpfe „die sich tagsüber steigerten“; sonst sind auf der Fieberkurve keine Krämpfe eingetragen, die bei diesen Dosen jedoch wahrscheinlich waren. Mehrmals ist Arbeitstherapie eingetragen. 1964/65 wurde er noch weitere 5mal aufgenommen und erhielt, wie beim ersten Aufenthalt und mit einer Ausnahme nach einem SMV, 4mal eine EKT und Medikamente zur Beruhigung, aber nicht mehr Majeptil.

> Auch der 21jährige Patient S6833 mit der Diagnose „Schizophrenie“ bekam 1964 anschließend an eine EKT mit 6 Anwendungen eine 6tägige Majeptilkur mit 1 x bis 6 x 10 mg; starke Krämpfe sind am 3. und 4. Tag verzeichnet. 3 Wochen später wurde die Majeptilkur wiederholt: mit an den ersten beiden Tagen 10 mg, in den folgenden 4 Tagen auf 50 mg pro Tag gesteigert; vom 4. bis zum 6. Tag sind Krämpfe verzeichnet. Während der Majeptilkur wurde Akineton (2 x 1 Tbl.)[1086] gegeben; täglich während seines 54tägigen Aufenthalts am Abend 30mg Valium. Auch er ging häufig zur Arbeitstherapie.

Weitere wesentliche Punkte bei der Auswahl eines Neuroleptikums waren nach Hofmann zum einen die spezifischen Effekte des Medikaments und zum anderen die „kranke Persönlichkeit“:

1084 Hoff-Skriptum, Allgemeine Psychiatrie [um 1961], S. 66.

1085 In der Anamnese wurden Wutanfälle, „Kotschmierattacken“, Jugend in Heimen und Jugendgefängnissen erwähnt. Bei allen seiner Aufnahmen kam er von der Sonderjustizanstalt Mittersteig für zurechnungsfähige, geistig abnorme Rechtsbrecher.

1086 „Biperiden ist ein Arzneistoff aus der Gruppe der Anticholinergika und wird als Antiparkinsonmittel verwendet. Biperiden wird auch zur Behandlung der oft starken extrapyramidalen Nebenwirkungen wie beispielsweise Körpersteifheit, Blickstarre und Akathisie eingesetzt, die viele Antipsychotika ab einer bestimmten Dosis verursachen können. Der Wirkstoff wird unter dem Handelsnamen *Akineton* seit 1953 vertrieben“ (https://de.wikipedia.org/wiki/Biperiden, 19.8.2020)

G. HOFMANN: Anwendungsweise von Neuroleptica und Antidepressiva 145

Tabelle 1. *Wesentliche Punkte bei der Auswahl eines Neurolepticums*

Medikament	Kranke Persönlichkeit
Dämpfungseffekt	Psychoseverlauf
Psychomotorik	Persönlichkeitsspezifische Abwehr
Gedankenablauf	Soziale Position
Bewußtseinslage	Grad der Differenzierung der Persönlichkeit
Wirksamkeitsdauer	
Nebenerscheinungen	Grad der Entdifferenzierung durch die Psychose
Pseudoparkinson	
Spezifischer Effekt (?)	

Beim Medikament galt es zum Beispiel den unterschiedlichen Dämpfungseffekt zu beachten, sowie die unterschiedlichen Wirkungen auf die Psychomotorik oder den Gedankenablauf und so fort. In Bezug auf die PatientInnen musste der Verlauf der Psychose, die (psychischen) Abwehrreaktionen, die soziale Situation oder der Grad der Differenzierung der Persönlichkeit für die Auswahl des richtigen Neuroleptikums berücksichtigt werden.[1087]

Während die klassischen, vor allem die hochpotenten Neuroleptika,[1088] die Plus-Symptomatik[1089] der Schizophrenie (Wahnvorstellungen, Ich-Störungen, Spastik, Anstieg des Sympathikotonus etc.) positiv beeinflussen konnten, blieben Erfolge zur Therapie der Minus-Symptomatik (Depression, Apathie, Akinese, kognitive Defizite etc.) aus. Als Erklärung wurde angenommen, dass das thalamo-retikuläre System für die Beeinflussung der Positiv-Symptome gehemmt, zur Aufhebung der Minus-Symptome jedoch aktiviert werden musste. Dazu sind Studien mit Amphetaminen und LSD durchgeführt worden.[1090] An der Universität Innsbruck wurde 1958 LSD „therapeutisch als Provokationsmittel häufig vor Schockkuren bei bestimmten Schizophrenien"[1091] gegeben. Ähnliches wurde auch an der Klinik Hoff getestet. Hier hat man entweder mit LSD oder mit Meskalin versucht, den chronischen Verlauf einer

[1087] HOFMANN, Über Kriterien einer differenzierten Anwendungsweise von Neuroleptica und Antidepressiva in der klinischen Psychiatrie (1964), S. 145.
[1088] So wurde etwa das hochpotente Neuroleptikum Haloperidol/Haldol (wenn auch in geringen Dosen) 1969 bei der 19jährige Patientin S20579 mit der Diagnose „Schizophrenie" – Anamnese: „verbale Halluzinationen" gegeben, die auch ein Beispiel für die Kombination von ‚alten Kuren' und neuen Medikamenten ist. Sie bekam in den ersten 12 Tagen nach der Aufnahme eine EKT mit 7 Anwendungen, am 20. Tag nach der Aufnahme wurde langsam mit einer Insulinkomatherapie begonnen (mit insgesamt 50 Komata), ab dem 30. Koma wurde alle zwei Tage sechs Mal im Koma ein Cardiazolschock „aufgesetzt"; ab dem 21. Koma und über das Ende der Insulinkur hinaus bis zur Entlassung (nach insgesamt 97 Tagen) wurden täglich 3x10gt Haloperidol gegeben
[1089] Vgl. oben S. 273 Anm. 1023.
[1090] AMBROZI/BIRKMAYER/DANIELCZYK, Die pharmako-dynamische Beeinflussung des thalamo-retikulären Systems als therapeutisches Prinzip in der Psychiatrie (1960), S. 727 (die drei Autoren waren Mitarbeiter der Neurologischen Abteilung des Altersheims der Stadt Wien, Lainz).
[1091] JOST/KATZELBERGER 1958: Zu den Reserpin-Kuren in der klinischen Praxis der Psychiatrie (1958), S. 155.

Hebephrenie in einen akuten zu überführen, da man der Meinung war, ein solcher sei besser durch eine gezielte Therapie zu beeinflussen.[1092] So wurde versucht, mit LSD 25 das aktuelle Zustandsbild der Hebephrenie in ein katatones oder paranoides zu überführen und dann mit EKT zu behandeln;[1093] während Mitte der 1950er Jahre Erfolge beobachtet wurden, hieß es 1962: „unsere therapeutischen Versuche, torpide Prozessverläufe mit LSD 25-Gaben aufzulockern und sodann einer Somatotherapie zu unterziehen, habe keine positiven Ergebnisse gebracht, wohl aber Versuche, nach medikamentöser Auflockerung Patienten einer Psychotherapie zugänglich zu machen.“[1094]

> Weiterhin die Verbindung der ‚alten Kuren‘ mit den neuen Psychopharmaka zeigt 1964 der Fall der 21jährigen Privatpatientin S18002 aus dem Ausland mit der Diagnose „Hebephrenie“:[1095] am Tag nach der Aufnahme bekam die Patientin eine „E-Schockbehandlung“ mit 4 vollmitigierten Anwendungen; dem folgten eine „Truxal-Valium und Melleriltherapie“, sowie einer Fieberkur (keine Malariakur, sondern eine milder Fiebertherapie mit acht Injektionen von Typhus-Vakzinen in Abständen von 2 bis 3 Tagen[1096]). „Unter dieser Behandlung kam es zu einer recht guten Besserung. Die Patientin wurde nach Hause entlassen und angewiesen weiterhin täglich 3 x 5 mg Valium und 3 x 1 Tbl. Encephabol weiterzunehmen.“ Bei ihrem Aufenthalt zur „Kontrolluntersuchung“ 1965 wurde sie für die folgenden 6 Monate „therapeutisch“ auf täglich 3 x 1 Tablette des Neuroleptikums Luvatren[1097] und gegen dessen Nebenwirkung auf 2 x 1 Tablette Kemadrin (Procyclidin), sowie abends 30 mg Valium eingestellt.

[1092] Hoff-Skriptum, Allgemeine Psychiatrie [um 1961], S. 65. Vgl. ARNOLD/HOFMANN, Untersuchungen über Bernsteinsäureeffekte bei LSD-25-Vergiftungen und Schizophrenien (1955). Hier gehen die Autoren auch auf Erfahrungen ein, die aus Versuchsreihen mit LSD an der Wiener Klinik für Neurologie und Psychiatrie in den Jahren 1947 und 1948 gewonnen werden konnten. Vgl. zu diesen Versuchsreihen SPRINGER, Pschopharmakologische Forschung und Behandlung an der Wiener Psychiatrischen Universitätsklinik und die Frühphase des Collegium Internationale Neuro-Psychopharmacologicum (CINP) (2016), S. 224-227. Vgl. HOFF, Lehrbuch der Psychiatrie (1956), S. 484: dass sie versuchten, das „hebephrene Zustandsbild durch die Gabe von Lysergsäurediäthylamid [LSD, GH] in ein mehr kataton oder paranoid gefärbtes Krankheitsbild zu verwandeln“ und dann einer EKT zu unterziehen.

[1093] HOFF/ARNOLD, Die Therapie der Schizophrenie (1954), S. 348 bzw. in der französischen Übersetzung HOFF/ARNOLD, Au sujet de la thérapie de la schizophénie (1955), S. 15.

[1094] ARNOLD/HOFF, Neuroleptika, Tranquilizer und Antidepressiva (1962), S. 36; auch zitiert von SPRINGER, Pschopharmakologische Forschung und Behandlung an der Wiener Psychiatrischen Universitätsklinik und die Frühphase des Collegium Internationale Neuro-Psychopharmacologicum (CINP) (2016), S. 227.

[1095] Lt. Befundbericht „handelt [es] sich um eine Retardation unbekannter Genese im Verein mit einem M[orbus] Bleuler“ „mit Exacerbationen!“

[1096] Sie bekam „anabole Substanzen“, was auf eine schwache körperliche Konstitution der Patientin schließen lässt.

[1097] Zu den Erfahrungen mit Luvatren: WEISER, Erfahrungen mit dem Butyrophenonpräparat Luvatren in der Behandlung der Schizophrenie (1968), S. 444-446.

2.4.2 Antidepressiva

Im heutigen Sprachgebrauch hat sich die Bezeichnung „Antidepressivum“ durchgesetzt, während in der psychiatrischen Fachliteratur der 1950er und 1960er Jahre auch Begriffe wie Thymoleptikum oder Thymoplegikum (von θυμός = thymos = Gemüt) oder die Unterteilung in aktivierende, euthymisierende und euphorisierende[1098] Medikamente vorgeschlagen wurden. Als erste Substanz dieser Wirkstoffklasse gilt das Imipramin (Handelsname: Tofranil), ein trizyklisches Antidepressivum, dessen Wirkung vom Schweizer Psychiater Roland Kuhn[1099] 1957 bei dessen klinischer Prüfung als Neuroleptikum entdeckt wurde. Ebenfalls im Jahr 1957 konnten auch die amerikanischen Psychiater Nathan S. Kline, Harry Loomer und John Saunders mittels klinischer Beobachtung feststellen, dass das gegen Tuberkulose eingesetzte Iproniazid (Handelsname: Marsilid*),* ein Monoamino-Oxidase-Hemmer (MOI), eine antidepressive Wirkung aufweist. Zu nennen ist auch das in den Wiener PatientInnenakten genannte psychomotorisch dämpfende Antidepressiva Amitriptylin (Handelsname: Saroten), das seit 1962 auch als Tryptizol auf dem Markt ist.[1100]

Im Untersuchungszeitraum kamen an der Klinik Hoff vor allem Tofranil und Marsilid als Antidepressiva zum Einsatz.[1101] Bereits 1958, also im ersten Jahr der Einführung des Tofranil, verweisen Stefan Hift und Hans Hoff auf die positiven Erfolge und Erwartungen und bauen sie Tofranil in ihren Therapieplan ein.[1102] In einem Vergleich zwischen Tofranil und Iproniazid kommen sie für beide Medikamente zu einem positiven Ergebnis („vielversprechende Anfangserfolge“), jedoch stünden dem vor allem bei Iproniazid „unangenehme Nebenerscheinungen gegenüber“.[1103]

[1098] Die Meinung, es gäbe euphorisierende Antidepressiva, hat sich nicht gehalten: Information Eberhard Gabriel.

[1099] Roland Kuhn war in den 1950er und 1960er Jahren Oberarzt, von 1970 bis 1980 Direktor der Psychiatrischen Klinik Münsterlingen in der Schweiz. 2012 meldeten sich ehemalige PatientInnen bei der Schweizer Presse, da sie in den 1960er und frühen 1970er Jahren an Testreihen von trizyklischen Antidepressiva, die in Münsterlingen unter Beteiligung von Kuhn entwickelt wurden, teilgenommen hätten und zwar ohne dass sie vonseiten der Ärzte davon in Kenntnis gesetzt wurden. Die Ergebnisse dieser Testreihen seien zudem nie publiziert worden. Um den Vorwürfen nachzugehen, hat der zuständige Kanton Thurgau im Dezember 2013 ein Forschungsprojekt bewilligt (vgl. http://de.wikipedia.org/wiki/Roland_Kuhn, 30.3.2015). Vgl. MEIER/KÖNIG/TORNAY, Testfall Münsterlingen (2019), passim.

[1100] Vgl. ARNOLD/FOIDL, Die Behandlung der endogenen Depression mit Amitriptyline (1961), S. 272-274.

[1101] In der Diskussion zu zwei Vorträgen – von H. Walter-Büel (Münsingen) und von C. G. Tauber (Bern) – über Marsilid äußerte sich SOLMS 1959 (Diskussionsbeitrag, S. 108) zurückhaltend.

[1102] Vgl. oben S. 253f.

[1103] HIFT/HOFF, Die organische Therapie der Psychose (1958), S. 1046, nennen keine dieser „unangenehmen Nebenerscheinungen“. Lt. https://de.wikipedia.org/wiki/Iproniazid (21.4.2023) wurde Iproniazid „1958 als

Aus den PatientInnenakten wird deutlich, dass dem Tofranil eine größere Bedeutung zukam als den Monoaminooxydase-Hemmern (MOI). Ein großer Vorteil bei Tofranil liege darin – so eine Untersuchung an der Neurologischen Abteilung des Altersheims der Stadt Wien, Lainz von 1960 –, dass es seine Wirkung nicht bei einer gesunden Person entfalte. „Gibt man es hingegen einer gehemmten endogenen Depression, dann sieht man im Breitbanddiagramm eine Verschiebung zur Normallage.“[1104]

1960, im zweiten Jahr nach der Entwicklung von Tofranil und im ersten Jahr nachdem es auf den Markt gekommen war, zeigt die Aufnahme mehrerer Artikel zum Thema auch namhafter nichtösterreichischer Autoren in der *Wiener Medizinische Wochenschrift*, wie intensiv die Diskussion und das Interesse am neuen Antidepressivum in Wien war. Im Vergleich von Tofranil und den MOI wurde durch seine raschere antidepressive und stark beruhigende Wirkung ersterem der Vorzug gegeben, während von der aktivitätssteigernden Wirkung der MOI bei den depressiven PatientInnen ein höheres Suizidrisiko befürchtet wurde.[1105] Vor allem bei älteren PatientInnen und bei organischen Störungen des zentralen Nervensystems wurde sowohl dem Tofranil als auch den MOI der Vorzug gegenüber der Elektrokrampftherapie gegeben, da sie weniger Nebenwirkungen hätten; auch würden sie eine sozial „mehr angepasste Besserung“ erlauben. Der ‚E-Schock‘ sollte jedoch seine Funktion als Notfalltherapie bei einer schweren Melancholie behalten.[1106]

Mehrfach wurde Anfang der 1960er Jahre gegen eine schematisierte Indikation und für eine individuell angepasste Behandlung (auch in Kombination mit Elektrokrampftherapie) argumentiert, [1107] sowie vor einer zu leichtfertigen Verschreibung von Tofranil gewarnt.[1108] Die Gefahr einer „ubiquitäre Verschreibung durch den Praktiker“ ergab sich aus dem Vorteil, dass mit der medikamentösen Behandlung der Depression ihre ambulante Behandlung „in

---

Antidepressivum zugelassen, doch schon 1961 wieder vom Markt genommen, nachdem Fälle von Hepatitis auftraten, und durch weniger lebertoxische Substanzen […] ersetzt.“

[1104] AMBROZI/BIRKMAYER/DANIELCZYK, Die pharmako-dynamische Beeinflussung des thalamo-retikulären Systems als therapeutisches Prinzip in der Psychiatrie (1960), S. 727.

[1105] HIPPIUS, Klinische und theoretische Aspekte der Pharmakotherapie des depressiven Syndroms (1960), S. 261f.

[1106] CAZZULLO, Wirkung der Monoaminooxydasehemmer (MOI) in der Depression (1960), S. 732.

[1107] Vgl. HOFMANN, Über Kriterien einer differenzierten Anwendungsweise von Neuroleptica und Antidepressiva in der klinischen Psychiatrie (1964), S. 153; GABRIEL [sen.], Erfahrungen mit Tofranil in der psychiatrischen Sprechstunde (1962), S. 222. Vgl.auch KUHN, Probleme der praktischen Durchführung der Tofranil-Behandlung (1960), S. 248; zu Kuhn vgl. oben S. 290 Anm. 1099.

[1108] So HIFT, Die Behandlung der endogenen Depression an der Wiener Klinik (1960), S. 290.

breitem Maße möglich“ wurde.[1109] In Graz, wo an der Klinik ab Oktober 1958 kein/e einzige/r depressive/r Patient/in mit einer ‚Schockbehandlung‘ allein, sondern immer in Kombination mit den neuen Medikamenten behandelt wurde,[1110] sah Ernst Pichler 1960 in Anbetracht der Häufigkeit endogener Depressionen und Melancholien die Notwendigkeit, vermehrt ambulant Tofranil-Behandlungen (ohne EKT[1111]) vorzunehmen, um die Spitäler zu entlasten und die PatientInnen adäquat behandeln zu können.[1112]

Erfahrungen mit Tofranil wurden 1960 auch aus der Nervenheilanstalt Rosenhügel berichtet, wo man Produktproben der Firma Geigy in einer Versuchsreihe testete. Die größten Erfolge des hemmungslösenden und anregenden Medikaments beobachteten die Ärzte bei der endogenen Depression. Gröbere Nebenwirkungen, von denen andere Autoren mitunter berichteten, konnten am Rosenhügel nicht festgestellt werden. Die Erfahrung, dass sich die Elektrokrampfbehandlung mit Tofranil reduzieren oder sogar ersetzen ließ, führte am Rosenhügel bereits 1959 zu einer Reduktion der Elektroschock-Serien um 20%. Die Autoren der Mitteilung bezeichneten diese Entwicklung ausdrücklich als „therapeutischen Fortschritt“[1113] und betonten in der Zusammenfassung ihres Artikels nochmals: *„Bemerkenswert war die Einsparung der Elektrokrampfbehandlung*.“[1114]

Die in der *Wiener Medizinischen Wochenschrift* um 1960 von österreichischen und einzelnen ausländischen Psychiatern publizierten Artikel lassen darauf schließen, dass nicht nur in Wien, sondern auch anderswo der Anteil der verabreichten Antidepressiva im Laufe des Untersuchungszeitraums sukzessive zunahmen, während gleichzeitig der Anteil der

---

[1109] EICHHORN, Die gegenwärtige Situation bei der klinischen Behandlung von Depressionen (1960), S. 224f.: Den „Vorteil […] gegenüber der Schockbehandlung“ sah der Grazer Kliniker darin, dass „eine individuelle, dem jeweiligen Zustand angepasste Behandlung möglich ist und sie lange Zeit hindurch bis zum Abklingen der Phase [auch ambulant, GH] durchgeführt werden kann.“ Er betonte, „daß man besonders bei der ambulanten Behandlung der Depressionen auf eine gleichzeitige Psychotherapie in keinem Fall verzichten“ dürfe. Der Begriff Psychotherapie taucht immer wieder in den Publikationen als Forderung auf, ohne dass geklärt wird, was damit gemeint ist; es waren wohl keine spezifischen Formen der Psychotherapie, sondern klärende und mutmachende Gespräche mit dem/der Patienten/in, um zugängliche individuelle und soziale Bewältigungsmöglichkeiten zu suchen und zu fördern u. ä.).
[1110] EICHHORN, Die gegenwärtige Situation bei der klinischen Behandlung von Depressionen (1960), S. 224, nennt Melleril, Largactil, Phenergan, Decentan, Marsilid und das hier im Frühjahr 1959 neu eingeführte Tofranil.
[1111] Vgl. RISTIC, Kurze Übersicht über einige mit Tofranil ambulant behandelte Fälle leichter Depressionen (1960), S. 752.
[1112] PICHLER, Ambulante Behandlung von Depressionszuständen mit Tofranil (1960), S. 754. E. Pichler war zu dieser Zeit als Arzt im Nervenambulatorium der Gebietskrankenkassa in Graz tätig. Er wandte sich aber ebenfalls gegen Verschreibungen durch Ärzte, die nicht vom Fach waren.
[1113] ROTH/MÜLLER 1960: Zur Tofranilbehandlung der Depressionszustände (1960), S. 226.
[1114] Ebd., S. 227.

Elektrokrampftherapien bei affektiven Störungen abnahmen. Letzteres ist auch für die ‚Klinik Hoff' aus der statistischen Auswertung der Datenbank zu erkennen: In den 1950er Jahren lag der Anteil der EKT bei endogenen Depressionen (dem Anwendungsgebiet innerhalb der Depressionen) noch bei 80,5% (1.203 von 1.494 Fällen), in den 1960er Jahren lag er nur noch bei 45,3% (655 von 1.447 Fällen).[1115] Mit Bezug auf die von ihm seit 1957 geleitete Abteilung am *Steinhof* berichtete Gross, dass 1955 (vor seiner Übernahme des Primariats) noch 132 Kranke mit insgesamt 1.787 „Elektroschocks" behandelt wurden, während 1959 nur 36 seiner PatientInnen insgesamt 138 „Elektroschocks" erhalten hätten.[1116] Sowohl die Anzahl der PatientInnen, die eine EKT erhielten, als auch die durchschnittliche Anzahl der Anwendungen pro PatientIn hätten abgenommen (1955: 13,5 „Elektroschocks" pro PatientIn; 1959: 3,8 Anwendungen pro PatientIn).

An der Wiener Klinik blieb die EKT bei endogenen Depressionen in Kombination mit Antidepressiva die Therapie der Wahl. So bezeichneten Arnold und Hoff in der kleinen Broschüre von 1962 zu „Neuroleptika, Tranquilizer und Antidepressiva" die „kombinierte Elektroschock-Imipramin-Kur" als die „derzeit die Standardtherapie der Melancholie bildende Kombinationskur". Eine „den Tofranilgaben um einige Tage vorausgehende Therapie mit 1 – 2 Elektroschocks" wurde empfohlen, wodurch „sich nicht nur eine wesentlich bessere subjektive Verträglichkeit der Schockbehandlung ergab, sondern darüber hinaus auch noch die vegetativen Nebenerscheinungen [„erhebliche Regulationsstörungen des Vegetativapparates"] der Tofranilmedikation als solche erheblich verminderten".[1117] Sie empfahlen deshalb folgende Therapie, wie sie „an der Klinik routinemäßig geübt" wurde: Beginnend mit einer EKT mit 4, 6 oder 8 Anwendungen (eine Anwendung jeden zweiten Tag) wurden ab dem Tag nach der zweiten bzw. dritten Anwendung (dem 4. bzw. 6. Tag der Behandlung) bis zum 60. bzw. 70. Behandlungstag[1118] (also bis weit über das Ende der EKT hinaus) täglich um 6, 9, 12 und 15h je 50 mg Tofranil gegeben; danach ließ man über 20 Tage die Medikation langsam auslaufen.[1119]

[1115] Vgl. oben S. 268.
[1116] GROSS, Erfahrungen mit neuroleptischen Behandlungsverfahren (1960), S. 718.
[1117] ARNOLD/HOFF, Neuroleptika, Tranquilizer und Antidepressiva (1962), S. 42.
[1118] Je nach der Zahl der geplanten Anwendungen verzögerte sich die Medikation etwas.
[1119] Vgl. die Tabelle in ARNOLD/HOFF, Neuroleptika, Tranquilizer und Antidepressiva (1962), S. 43. Drei Jahre später scheint sich die Reihenfolge bei „einer depressiven Manifestation im Rahmen des manisch-depressiven Krankheitsgeschehens (MDK)" in der Kombination von EKT und Antidepressiva geändert zu haben (was zur Abnahme der EKT-Anwendungen beigetragen haben dürfte): vgl. ARNOLD/KRYSPIN-EXNER, Zur Frage der Beeinflussung des Verlaufs des manisch-depressiven Krankheitsgeschehens durch Antidepressiva (1965), S- 929 und S. 934: „Die einfache Empirie hat gezeigt, daß es besser ist, ein stark wirksames Antidepressivum zu geben

Im bereits zu den Neuroleptika zitierten Aufsatz von Hofmann 1964 wurde wieder „versucht, isoliert die persönlichkeitsspezifische Abwehrreaktion sowie die Form der Depression als Kriterien“ für die Wahl der Therapie heranzuziehen und die Differenzierung des Vorgehens in eine Tabelle zu fassen:[1120]

Abwehrreaktion	Therapeutische Maßnahme	Form der Depression
	Biologische Therapie	Stupor<br>Legierte Depression<br>oder depressiv<br>legierte Schizophrenie
	Tofranil	Gehemmte Depression
	Amitriptiline	Ängstlich<br>agitierte Depression
Neurotisch	MAO-Hemmer<br>Insidon	Depression in senio
		Neurotisch durchmischte<br>Depression
Erregung	BB. NL.	Legierungspsychose akut
Unruhe	LZ. NL.	Dysphorie
Hypochondrisch	LZ. NL.	Legierungspsychose
Psychosomatisch	LZ. NL.	MDK
Spannung	Biobamat	
Phobie	Librium	
Neurasthenisch	Stimulantien	
Depressiver „Charakter“		
„Existentielle“<br>Depression	Psychotherapie	

Aus dieser Tabelle sind die Therapievorschläge über dem Strich in Bezug auf die Form der Depression und als alleinige medikamentöse Therapie ersichtlich, unter dem Strich die Kombination von Psychopharmaka (einmal von Psychotherapie) im Rahmen einer Elektrokrampfbehandlung; einschränkend schreibt Hofmann: „sicherlich ist jedoch bei den zuletzt genannten Formen (neurasthenisch, depressiver Charakter usw.), die schon zu den

und bei mangelnder Wirkung mit einer kurzen Elektroschockserie zu kombinieren, als von vornherein Elektroschocks anzuwenden und dann einen Imipraminkörper einzusetzen.“

[1120] BB = Barbiturat, NL = Neuroleptikum, LZ.NL. = Langzeitneuroleptikum.

Formen der Neurosen mit möglicherweise endogener Komponente überleiten, auch die alleinige Therapie mit Psychopharmaka möglich.“[1121]

In den Publikationen der Wiener Kliniker zu den neuen Antidepressiva (ab 1958) wird deutlich, dass sie diesen gegenüber keinesfalls negativ eingestellt waren, dass sie jedoch nicht bereit waren, die alten Therapien (in diesem Fall die EKT) in ihren positiven und von ihnen bei einigen Diagnosen und Krankheitsverläufen als besser eingeschätzten Wirkungen völlig aufzugeben. Die von Bernd Küfferle und Helmut Schultes zu den an der Klinik 1967 verwendeten sedierenden Medikamenten angeführten geringen Mengen von meist schwach wirksamen Neuroleptika könnte ein Hinweis darauf sein, dass an der Klinik mehr auf die alten körperlichen Kuren als auf Therapien mit Psychopharmaka gesetzt wurde.[1122]

2.4.3 Tranquilizer, Sedativa, Anxiolytika

Tranquilizer wurden auch als Ataraktika oder Anxiolytika bezeichnet[1123] und sollten vorwiegend angstlösend wirken. Zuweilen wurde auch synonym der Begriff „Sedativa“ verwendet, der genau genommen Medikamente bezeichnet, die Erregungszustände abmildern und die PatientInnen beruhigen, während Hypnotika ihre Bezeichnung durch ihre schlaffördernde Wirkung erhielten. Eine strenge Einteilung ist jedoch praktisch nicht möglich, da die meisten Medikamente dieser Gruppe dosisabhängig anxiolytisch, sedativ, hypnotisch und teilweise narkotisch wirken.

1912 kam mit Phenobarbital (Luminal) das erste Barbiturat auf den Markt.[1124] In den 1950er Jahren wurde an der Wiener Psychiatrie eine Reihe von Barbituraten regelmäßig verwendet, so zum Beispiel Medinal, Plexonal, Agrypnal, Cyclobarbital, Nembutal, Prominal und Secomal. Ihre Hauptindikation in der Psychiatrie lag in der Behandlung der Epilepsie und der Beruhigung und Sedierung der PatientInnen, jedoch wurden Barbiturate auch zur Vorbereitung einer Psychotherapie verabreicht. Ziel der so genannten Narkoanalyse, für

---

[1121] HOFMANN, Über Kriterien einer differenzierten Anwendungsweise von Neuroleptica und Antidepressiva in der klinischen Psychiatrie (1964), S. 149f.
[1122] KÜFFERLE/SCHULTES, Übersicht über die an der Wiener Psychiatrischen Klinik im Jahr 1967 zur Sedierung verwendeten Medikamente (1969), S. 71-75.
[1123] Anxiolytika (lateinisch anxius = Angst; griechisch lytikos = fähig zu lösen) und Ataraktika (griechish taraktor = ausgeglichen): https://de.wikipedia.org/wiki/Tranquilizer (25.4.2023).
[1124] HALL, Zur Geschichte der deutschen Pharmakopsychiatrie von 1844 Bis 1952 (1997), S. 207-210

welche Thiopental oder Narkothion verwendet wurden, war eine Kurznarkose, aus welcher die PatientInnen etwa 10-15 Minuten später wieder belebter und vor allem gesprächiger aufwachen sollten.[1125]

Barbiturate haben erhebliche unerwünschte Wirkungen. Da Barbiturate jedoch weitaus stärkere sedativ-hypnotische Wirkungen besitzen als pflanzliche oder andere Tranquilizer, wie Paraldehyd oder Meprobamat, sind sie bis zur Entdeckung und Einführung der Benzodiazepine (Librium 1960) regelmäßig verwendet worden. In den 1960er Jahren wurden sie jedoch langsam aus dem klinischen Alltag verdrängt, eine Tendenz, die sich bereits in der Stichprobe der Pilotstudie für die Jahre 1955–1960 abzeichnet: Während 1955 noch etwa 30% der Patienten mit Barbituraten behandelt wurden, sind es im Jahr 1960 gerade noch 5%.

1960 brachte Hoffmann-La Roche mit Librium (Chlordiazepoxid) das erste Benzodiazepin auf den Markt, das auch im selben Jahr an der Klinik Hoff eingesetzt wurde. Im Untersuchungszeitraum waren jedoch Paraldehyd (Trimer des Acetaldehyd), Doriden (Glutethimid) und Miltaun (Meprobamat) die am häufigsten verabreichten Tranquilizer. Nun wurde üblicherweise den Benzodiazepinen die Gruppe der Barbiturate und der Non-Benzodiazepin-Tranquilizer gegenübergestellt. Paraldehyd, das schon seit 1883 als Hypnotikum Verwendung fand, weist einen raschen Gewöhnungseffekt auf, sodass eine dauerhafte Therapie trotz Dosissteigung oftmals ineffizient war.[1126] Sein großer Vorteil, weshalb es selbst in den 1950er Jahren noch zum Standardrepertoire der Sedativa zählte, lag im relativ günstigen Nebenwirkungsprofil (im Vergleich zu den Barbituraten).[1127] Doriden kam 1954 auf den Markt und wurde laut Bleulers Lehrbuch der Psychiatrie als leichtes Beruhigungsmittel klassifiziert.[1128] Die anxiolytische Wirkung des Meprobamats wurde 1954 von Frank Berger entdeckt.[1129] Zwischen 1955 und 1960 kamen an der Klinik Hoff

---

[1125] JOST, Zur Verwendung der Allgemeinnarkose in der Psychiatrie. Mit besonderer Berücksichtigung der Elektroschockbehandlung unter Mitigierung durch Lysthenon (1957), S. 337.

[1126] Nach Eberhard Gabriel wurde Paraldehyd wohl vor allem als Schlafmittel und in der Behandlung des Delirium tremens (um das Delir ‚auszuschlafen‘) angewandt. Nach den PatientInnenakten war die Anwendung meistens kurz (2 – 4 Tage), manchmal aber (in Kombination) auch länger: So bekam der 63-jährige Patient A1727 mit „Dissimulierende Melancholie“ bei seinem 20tägigen Aufenthalt an der Klinik 1952 eine EKT mit 4 Anwendungen und vom 6. Bis 9. Tag tgl. 0,5 Medinal+6gParaldehyd, dann noch weitere 3 Tage nur Paraldehyd (6g bzw. 4g). Der 23jährige Patient S1718 mit der Diagnose „Katatoner Stupor“ bekam bei seinem 2. Aufenthalt 1952 eine EKT mit 9 Anwendungen und an den ersten beiden Tagen 10mg. Paraldehyd; am 4. Tag wurde er künstlich Ernährt und bekam 3x2 Amp. Sympathol und 2x 1 Amp. Coramin, am 5. Tg. 3x1 Amp. Coramin; vom 8. Bis 14. sowie am 17. Tag bekam er 10g Paraldehyd, vom 11. Bis 14. und am 16. Tag 0,5g Medinal.

[1127] HALL, Zur Geschichte der deutschen Pharmakopsychiatrie von 1844 Bis 1952 (1997), S. 167-170.

[1128] BLEULER, Lehrbuch der Psychiatrie ([10]1960), S. 163-165.

[1129] MÖLLER/LAUX/DEISTER, Psychiatrie und Psychotherapie (2009), S. 14.

unterschiedliche Generika zum Einsatz (Miltaun, Miltown, Biobamat). Vor allem die Kombination von Doriden und Miltaun wurde regelmäßig zu Aufnahmebeginn oder auch während der Elektrokrampftherapie verabreicht. Üblicherweise bekamen PatientInnen an den ersten beiden Tagen leichte Tranquilizer, meist Paraldehyd oder die eben angesprochene Kombination aus Miltaun und Doriden, etwas seltener auch ein Barbiturat. Die meisten Neuankömmlinge sollten zuerst einmal beruhigt werden.[1130]

2.4.4 Schlussbemerkungen zu den Psychopharmaka

In diesem Abschnitt wurde der Einsatz von Medikamenten nach den zeitgenössischen Erfahrungsberichten vor allem der Wiener Kliniker behandelt, ergänzt durch Vergleiche mit der Wiener Praxis, d. h. mit Ergebnissen aus der repräsentativen Stichprobe der Pilotstudie für die Jahre 1955–1960, sowie aus den PatientInnenakten des gesamten Untersuchungszeitraums des Projekts. Es ging bei den drei hauptsächlich verwendeten Medikamentengruppen einerseits um die kurzfristigen Anwendungen und andererseits um die längerfristigen Therapien gemeinsam mit den ‚Schock- und Fieberkuren' bzw. als Ersatz für diese.

Bereits 1952 wurden in Wien Studien zum eben entdeckten ersten Neuroleptikum Chlorpromazin (Largactil) gemacht, ebenso ab 1957 Studien zu den ganz neuen Antidepressiva Imipramin (Tofranil) und Iproniazid (Marsilid). Die neuen Psychopharmaka wurden in ihrer symptombezogenen Wirkung rasch in den „Gesamtbehandlungsplan" aufgenommen und häufig – je nach Diagnose und Krankheitsverlauf – auch mit den ‚Schock- und Fiebertherapien' kombiniert. Bei schweren Depressionen blieb (wie häufig auch international[1131]) die Elektrokrampftherapie die Methode der Wahl. Die Psychopharmaka haben die ‚großen alten Kuren' in der ‚Ära Hoff' aber auch mehrfach und zunehmend – je nach Diagnose (etwa bei der Manie schon in den 1950er Jahren) und Krankheitsverlauf – abgelöst. So ist an der Wiener Klinik eine deutliche Abnahme der Insulinkoma- und der Elektrokrampftherapien in den 1960er Jahren festzustellen, die auch hier auf den Ersatz durch Psychopharmaka in mehreren Fällen zurückzuführen ist.

[1130] ARNOLD, Klinische Erfahrungen mit dem Neuroleptikum Truxal (1959), S. 892: vor allem die Sedierung durch Neuroleptika hätte, so Arnold, erst die Möglichkeit geschaffen, „in den letzten Jahren systematisch gesperrte Stationen zu de facto offenen umzugestalten, Fenstergitter und Netzbetten restlos zu entfernen und die sogenannten unruhigen Abteilungen in ruhige zu verwandeln".
[1131] Vgl. (vor den neuen Antidepressiva) DELAY/DENIKER/PAUWELS, Cure de sommeil et cures neuroleptiques en psychiatrie (1956), S. 137f. (die beiden ersteren waren die Entwickler des ersten Neuroleptikums Largactil).

Zur medikamentösen Behandlung der Psychosen bei Kindern schrieb Walter Spiel bereits 1961 in seiner Habilitationsschrift: „Im allgemeinen hat man den Eindruck, daß im Kindesalter die Therapie mit Neuroleptika und Tranquillizer sich besser durchsetzen wird als die Insulinschockbehandlung und in gewissem Sinn auch die Elektroschockbehandlung, weil diese Methoden vom Publikum nicht so perhorresziert werden und weil sich das Schwergewicht der Beeinflussung bei kindlichen Psychosen scheinbar doch auf den Sektor der Psychotherapie verlegt."[1132]

Darin, dass die ‚alten' Kuren nicht vollständig zugunsten der Psychopharmaka aufgegeben wurden, war die Wiener Klinik in der ‚Ära Hoff' keinesfalls ein Einzelfall. Das zeigen die Beispiele zur Insulinkomatherapie und zur Elektrokrampftherapie aus der französischen Literatur,[1133] aber auch aus der DDR und BRD.[1134]

Der ‚pharmakologische Paradigmenwechsel' verlief in Wien langsamer als etwa in Zürich, wo bereits Mitte der 1960er die Insulinkur nicht mehr und die Elektrokrampftherapie, laut Eberhard Gabriel, der 1966 dort gearbeitet hat, kaum noch angewandt worden sein dürften.[1135] Die Studie von 2002 zu Zürich spricht zu den Stichjahren 1959 und 1969 vom Abschluss der ‚Kurperiode', der in den 1950er Jahren mit dem „fulminanten" „Einsatz von Neuroleptika" bzw. in den 1960er Jahren mit dem ansteigenden Einsatz der Antidepressiva zusammenfiel.[1136] Die Wiener Position zum steigenden Einsatz der Psychopharmaka fassten

---

1132 SPIEL, Die endogenen Psychosen des Kindes- und Jugendalters (1961), S. 112.
1133 Vgl. Juillet 1968, zitiert oben S. 185f. und S. 236f.
1134 So kam etwa in Bezug auf die EKT eine Studie aus der psychiatrischen Abteilung der Universitäts-Nervenklinik Rostock von 1963 zum Ergebnis, „daß trotz Kombination verschiedener Medikamente auf die Konvulsionstherapie nicht verzichtet werden kann", dass jedoch „die Zahl der Konvulsionen erheblich abgenommen hat und die Heilungserfolge bedeutend besser geworden sind": GIERCKE/KLEINPETER, Vergleichende Untersuchungen über Behandlungserfolge bei psychiatrischen Kranken zur Zeit der Konvulsivtherapie und der Psychopharmaka (1963), S. 336. Zur Insulinkomatherapie vgl. oben S. 238 Anm. 914, das Zitat aus RZESNITZEK, „Schocktherapien" und Leukotomie in der DDR-Psychiatrie (2018), S. 299.
1135 Im zitierten Forschungsbericht von 2002 ist die Kurve zur Insulinkur zum Stichjahr 1969 kaum noch vorhanden.
1136 Vgl. TANNER/MEIER/HÜRLIMANN/BERNET, Zwangsmassnahmen in der Züricher Psychiatrie (2002), S. 93-95: „In den Jahresberichten wird bereits seit 1953 eine ‚im Zusammenhang mit den Fortschritten der Wissenschaft erfolgte Verschiebung' festgehalten, welche die körperlichen Behandlungsformen betraf: ‚Unsere Hauptaufgabe, die Behandlung, erfuhr im Berichtsjahr mit den Fortschritten der Wissenschaft eine neue Belebung. Die Einführung neuer Heilmittel ermöglichte es, einen Teil der früheren Kuren mit Insulin, mit Schlafmittel und mit Elektroschock […] zu ersetzen.' So scheint die Behandlung mit den Neuroleptika Largactil, Melleril und dem Antidepressivum Tofranil bereits in den 50er Jahren zugenommen zu haben. […] in den 60er Jahren kamen die Medikamente Nozinan, Vesitan, Lithium und Haldol auf" und „gingen ‚körperlich eingreifende Kuren', wie Elektroschock- und Insulinkur […] zu Gunsten der ‚milderen medikamentösen Kuren'

Hans Hoff und sein nun erster Oberarzt Ottokar H. Arnold 1962 am Ende einer Broschüre zu den neuen Medikamenten, die deren vielfältige Anwendung auch in Wien zeigt, folgendermaßen zusammen: „Ob somit die modernen Psychopharmaka eine neue Ära der Psychiatrie eingeleitet haben, kann derzeit noch nicht entschieden werden. Sicher haben sie nicht die bisher bewährten Behandlungsmethoden z. B. der modernen modifizierten Insulin- und Elektroschocktherapie außer Kraft gesetzt, so sehr und so gerne einige ‚Erneuerer der psychiatrischen Therapie' dies auch gesehen hätten."[1137]

3 Zusammenfassung zum Projekt und seinen Ergebnissen

Das auf eine Laufzeit von zwei Jahren konzipierte Forschungsprojekt konzentrierte sich auf die diagnostische und therapeutische Praxis an der Psychiatrie der Wiener Psychiatrisch-Neurologischen Universitätsklinik in der Leitung durch Hans Hoff in Bezug auf die „'großen' körperlichen Behandlungsverfahren", die „Schock- und Fieberkuren" aus der Zwischenkriegszeit (1918 Malariafiebertherapie, 1933 Insulinkomatherapie, 1934 Cardiazolkrampftherapie, 1938 Elektrokrampftherapie), sowie auf ihren sukzessiven Ersatz durch die neuen Psychopharmaka (1952 Neuroleptika, 1958 Antidepressiva). Der Zeitraum wird als „pharmakologische Wende" beschrieben, als sukzessiver Übergang von den in der Zwischenkriegszeit entwickelten ‚großen' körperlichen Kuren zu den neuen medikamentösen Therapien – wobei im Forschungsprojekt auch der Übergang von der Malariafiebertherapie in ihrer ‚klassischen' Anwendung bei Neurolues auf Penicillin einbezogen wurde. Im Projekt wurde die statistische Auswertung der PatientInnenakten ins Zentrum gestellt, um die Häufigkeit sowie die Breite der Anwendung der ‚großen alten Kuren' und die Veränderungen im Untersuchungszeitraum zu zeigen. Um diese Ergebnisse in den Kontext der wissenschaftlichen Diskussion der Zeit zu stellen, waren aus der zeitgenössischen Literatur unterschiedliche Positionen der Ärzte / Ärztinnen zu erheben. Fallbeispiel sollten zur Klärung einiger Fragen zur Praxis beitragen.

Der Übergang vollzog sich vor allem in den zentralen Anwendungsbereichen der ‚großen alten Kuren', in den fünf Diagnosefeldern Neurolues, schizophrene und affektive Erkrankungen, Intelligenzmängel und Psychopathie. Die Akten der PatientInnen mit diesen

weiter an Zahl zurück. Zudem erhöhte sich in den letzten beiden Stichjahren [1959 und 1969] auch der Einsatz von Schlaf- und Beruhigungsmitteln stark."

[1137] Vgl. ARNOLD/HOFF, Neuroleptika, Tranquilizer und Antidepressiva (1962), S. 44f.

Diagnosen, die im Archiv der Universitätsklinik für Psychiatrie und Psychotherapie des Allgemeinen Krankenhauses Wien mit den Jahren 1951 bis 1969 archiviert sind, sind sehr umfangreich, wenn auch nicht lückenlos erhalten.[1138] So sind für diesen Zeitraum für die Kinderstation ca. 2.400 Akten überliefert, die verlorenen Akten werden auf etwa ein Drittel geschätzt. Für die beiden psychiatrischen Erwachsenenabteilungen sind in diesen Jahren ca. 90.000 Akten archiviert und hier dürften etwas mehr als 20 Prozent der Akten fehlen. In dieser nicht personenbezogenen, historischen Untersuchung ist jedoch davon auszugehen, dass die aus diesem umfangreichen Quellenmaterial quantitativ und qualitativ gezogenen Annahmen und Schlussfolgerungen trotz dieser Fehlstellen valide sind.

Aus den ca. 90.000 in den Jahrgängen 1951 bis 1969 archivierten PatientInnenakten der Erwachsenenabteilungen der Psychiatrie[1139] wurden die Informationen aus jenen Fällen, die eine der Diagnosen aus den genannten fünf Diagnosefeldern und einen Aufenthalt von mindestens fünf Tagen hatten, in eine Datenbank zur statistischen Auswertung aufgenommen. Es sind 14.919 Akten der Erwachsenenabteilungen (etwa die Hälfte aller Aufnahmen mit einem Aufenthalt von mehr als vier Tagen und wegen der Mehrfachaufnahmen sind es Akten von insgesamt 11.720 PatientInnen), in denen in 6.915 Fällen eine oder mehrere der Koma-, Krampf- und / oder Fieberkuren gegeben wurden: 5.886mal eine Elektrokrampftherapie (EKT), 965mal eine Insulinkomatherapie (davon ein auf unter 10% geschätzter Teil eine Insulinsubkomatherapie[1140]), 772mal eine Malariafiebertherapie, 114mal eine Cardiazolkrampftherapie (fast immer im Insulinkoma, selten als „Cardiazol-Elektroschock“).

Die Akten der bis 14jährigen PatientInnen der Kinderstation wurden wegen der diagnostischen und therapeutischen Unterschiede zur Erwachsenenstation in eine eigene Datenbank eingegeben und gesondert in einem Kapitel des Projektendberichts von 2015 ausgewertet.[1141] Diese Auswertung wurde nicht in diese Publikation übernommen, die Daten jedoch ergänzend bzw. im Vergleich zu jenen der Erwachsenenstationen im vorliegenden Text eingearbeitet. Hier finden sich 35 Anwendungen der Malariafiebertherapie, 21

[1138] Vgl. zur Archivierung und zu den Verlusten, oben S. 15.
[1139] Zur großen Zahl der Aufnahmen und ihrer starken Abnahme in den ersten 4 Tagen um ca. 2/3, vgl. oben S. 20.
[1140] In die Datenbank wurden beide Insulinkuren aufgenommen, ohne zu unterscheiden. Nach einer ausführlichen, aber nicht alle Fälle umfassenden, unsystematischen Nachrecherche in den PatientInenakten wurde im Text die Insulinsubkomatherapie in ihren Anwendungen und Erwartungen behandelt.
[1141] Vgl. GEIGER, Kinderstation (2015), S. 253-255 (Malariafiebertherapie), S. 265 (EKT) und S. 266f (Insulinkomatherapie).

Elektrokrampftherapien bei endogenen Psychosen und sechs Fälle, in denen die Insulinkomatherapie angewandt wurde – drei davon in Kombination mit EKT.

Der Malariafiebertherapie und der Insulinkomatherapie, die an der Wiener Klinik in der ganzen ‚Ära Hoff' angewandt wurden, während sie an anderen Kliniken durch Penicillin bzw. Neuroleptika bereits in den 1950er Jahren ersetzt worden waren,[1142] galt aufgrund der besonders langen Anwendung (auch der Anwendung der Malariafiebertherapie bei nichtluetischen Erkrankungen) und da beide Therapien in Wien entwickelt worden waren, ein besonderes Augenmerk. Das Zögern, die beiden ‚Wiener Therapien' aufzugeben, war wohl auch auf den Klinikchef Hans Hoff zurückzuführen: Er stand in der Tradition der Wiener Psychiatrie der Zwischenkriegszeit, wollte deren internationalen Ruf erhalten bzw. wiederherstellen – ein Renommee, das durch diese beiden Therapien, an deren Entwicklung Hoff mitgewirkt hatte, wesentlich mitbegründet worden war.

Die neuen Psychopharmaka wurden neben den ‚alten' somatischen Therapien bereits sehr früh[1143] angewandt – ebenso Penicillin bei der progressiven Paralyse neben und gemeinsam mit der Malariafiebertherapie. Die Anwendungen der „‚großen' alten Kuren" oder „Heroischen Therapien", wie die Koma-, Krampf- und Fieberkuren als stark intervenierende Therapien genannt wurden, finden sich – wie auch anderswo[1144] – neben den Psychopharmaka im Wiener „Gesamtbehandlungsplan". Ihre Anwendung ging in diesen beiden Jahrzehnten der „pharmakologischen Wende" aber auch hier sukzessive zurück.

3.1 Die Elektrokrampftherapie

Die letzte der vier ‚großen alten Kuren' der Zwischenkriegszeit, die seit ihrer Einführung durch Ugo Cerletti 1938 zwar – nicht nur in Fachkreisen, sondern immer wieder sehr heftig in der medialen Öffentlichkeit – kontrovers diskutiert wurde, die sich aber dennoch rasch weltweit verbreitet hatte, war in Wien im Untersuchungszeitraum jene, die deutlich am

---

[1142] Für die Universitätsklinik „Burghölzli" in Zürich gibt es eine Studie mit Daten, die diesen Unterschied verdeutlichen: TANNER/MEIER/HÜRLIMANN/BERNET, Zwangsmassnahmen in der Züricher Psychiatrie (2002), sowie MEIER/BERNET/ DUBACH/GERMANN (Hrsg.), Zwang zur Ordnung. Psychiatrie im Kanton Zürich (2007).
[1143] Zum 1952 in Frankreich entwickelte erste Neuroleptikum Largactil wurde von Wiener Klinikern bereits im selben Jahr eine Forschungsmitteilung und 1953 ein Forschungsbericht publiziert (vgl. oben S. **273** Anm. 1026).
[1144] RZESNITZEK, Schocktherapien und Psychochirurgie in der frühen DDR (2015), S. 1417, etwa schreibt zur DDR: „Die Einführung der Psychopharmaka ab Mitte der 1950er führte weder zur Aufgabe der Elektrokrampf- noch der Insulinkoma-Therapie."

häufigsten angewandt wurde. An der Wiener Psychiatrie war man bereits Anfang der 1950er Jahren davon überzeugt, mit der Elektrokrampftherapie in der Anwendung mit dem Muskelrelaxans Lysthenon über eine risikoarme und höchst wirksame Methode zur Behandlung sowohl von bestimmten schizophrenen, als auch von manisch-depressiven Erkrankungen bzw. ihren Symptomen zu verfügen. Hans Hoff schrieb 1952 in seiner Begründung des Vorschlags, Ugo Cerletti (neben Manfred Sakel) den Nobelpreis für Physiologie und Medizin des Jahres 1953 zu verleihen, von den Erfolgen in der Abkürzung der Phasen der Melancholie, in der Behandlung der akuten tödlichen Katatonie und in der Beruhigung unruhiger PatientInnen, wodurch sich die Pflege in den psychiatrischen Kliniken und Heilanstalten wesentlich vereinfacht habe.[1145]Die Zahl ihrer Anwendungen, die zu annähernd gleichen Teilen schizophren und affektiv Erkrankte betrafen, ging im Untersuchungszeitraum nicht sehr stark, aber doch deutlich zurück: von 382 Elektrokrampftherapien 1951, über einen Höhepunkt von 469 1955 und 366 1959, ist ab 1960 – mit 357 EKT ein kontinuierlicher Abfall bis auf 123 1969 zu erkennen.[1146] Sie blieb jedoch in Wien auch in den 1960er Jahren im Vergleich etwa zur Züricher Universitätsklinik sehr hoch. Einer der Gründe dafür dürfte die Einschätzung maßgeblicher Wiener Kliniker gewesen sein, dass die EKT „weniger Nebenerscheinungen und Komplikationen“ verursache „als die meisten medikamentösen Therapien“.[1147]

Um unerwünschte Nebenwirkungen der EKT deutlich zu verringern, hatte der Wiener Kliniker Ottokar H. Arnold bereits Anfang der 1950er Jahre entscheidend zur Verbesserung der Anwendung der EKT beigetragen, indem er Lysthenon als Muskelrelaxans einsetzte. Dies

---

[1145] Hans Hoff an das Nobelkomitee (von dem er um einen Vorschlag gebeten wurde), 11.10.[1952], Kopie im Josephinum Wien, NL-Hoff MUW-AS-006005-0015: „Die Elektroschocktherapie ist leicht durchzuführen und ihre Gefahren sind, wie tausendfache Erprobung zeigt, ausserordentlich gering. Die Therapie Cerletti's war zunächst auf die Schizophreniebehandlung gerichtet. Sie machte aber ihren eigenen Weg und heute ist sie die Therapie der Wahl des manisch-depressiven Irreseins. Sie ist zwar nicht imstande, das manisch-depressive Irresein als Krankheit zu heilen, wohl aber gelingt es dank dieser Therapie, einzelne Phasen wesentlich abzukürzen, mindestens aber das aktuelle Syndrom zu beseitigen. Abgesehen davon erscheint es immer wieder wie ein Wunder, wenn manche melancholische Phase, die früher 9 Monate bis 2 Jahre gedauert hätte, nun in wenigen Wochen völlig beendet werden kann. Die Qual einer Melancholie ist so gross, dass die Abkürzung der melancholischen Phase oder die Beseitigung des Syndroms für den Patienten von ungeheurer Bedeutung ist. Die Elektroschocktherapie hat schließlich die akute tödliche Katatonie, eine Erkrankung, die früher eine Mortalität von 100% aufwies, zu einer Krankheit gemacht, bei der die Mortalität zwischen 10-15 Prozent schwankt. Gerade die akute tödliche Katatonie war früher eine der häufigsten Todesursachen an einer Psychiatrischen Klinik. Mit Hilfe der Elektroschockbehandlung gelingt es, unruhige Patienten zu beruhigen und wenn heute die Pflege an einer psychiatrischen Klinik oder in einer psychiatrischen Heilanstalt vereinfacht ist, so ist dies Ugo Cerletti zu danken.“

[1146] Vgl. das Balkendiagramm Abb. 11, oben S. 259.

[1147] Vgl. HIFT, Die Behandlung der endogenen Depression an der Wiener Klinik (1960), S. 289: EKT verursache „weniger Nebenerscheinungen und Komplikationen […] als die meisten medikamentösen Therapien“, zitiert oben im Text S. 257.

half, durch die Krampfanfälle verursachte Knochenbrüche während des Eingriffs zu verhindern. Wiener Kliniker klärten zudem die Indikationen für die EKT in Fachveröffentlichungen. Auch wurden die Psychopharmaka schon in den 1950er Jahren in die Behandlung einbezogen, alleine oder gemeinsam mit der Elektrokrampftherapie.

3.2 Die Insulinkomatherapie

Die Insulinkomatherapie wurde seit ihrer Einführung 1933 an der Wiener Klinik durch Manfred Sakel äußerst kontrovers diskutiert, setzte sich aber dennoch rasch und weltweit durch. Sie war an der Wiener Klinik im Untersuchungszeitraum die zweithäufigste der „‚großen' alten Kuren". Hans Hoff schrieb zu seiner Einschätzung der Therapie, als er dem Nobelkomitee vorschlug, Manfred Sakel gemeinsam mit Ugo Cerletti 1953 bzw. 1958 den Nobelpreis für Medizin zu verleihen: „durch die Kenntnis der Schocktherapie" sei „die gänzliche Umstellung in der Psychiatrie von einer Wissenschaft, die sich mit rein deskriptiven Beschreibungen von Krankheitsbildern begnügte zu einem hochaktiven therapeutischen Teil der Medizin […] eingeleitet" worden; es sei das „Verdienst Sakels gezeigt zu haben, dass eine Möglichkeit besteht, die Schizophrenie zu heilen" und der „Verdienst von Cerletti, durch die Einführung der Elektroschockbehandlung das Los von Melancholikern […] verbessert zu haben".[1148]

Die Insulinkomatherapie wurde fast ausschließlich bei schizophrenen Erkrankungen angewandt. Ähnlich wie bei der EKT wurde bei der Insulinkur an der Wiener Klinik die Indikation präzisiert, auf wenige Diagnosen der Schizophrenie eingeschränkt,[1149] und auch hier trugen Wiener Kliniker Wesentliches zur Verbesserung der Anwendung und Verminderung der Zwischenfälle bei: So gelang ihnen mit dem Hexamethonium-Derivat *Depressin* als ‚Ganglienblocker' die Insulintoleranz deutlich zu senken.[1150] Ottokar H.

[1148] Zitat aus Hans Hoff an das Nobelkomitee, 19.10.1957, Kopie im Josephinum Wien, NL-Hoff MUW-AS-006005-0011-068 Zl. 68/5. 1957 bemerkte Hoff einleitend zu seinem Vorschlag: „Obwohl gerade jetzt Bestrebungen im Gange sind, die Schocktherapie bei der Behandlung der Schizophrenie und des manisch-deperessiven Irreseins zurückzudrängen" mache er seinen Vorschlag – und spielte damit auf deren Ersatz durch medikamentöse Therapien und durch Psychotherapien an.

[1149] Vgl. die Tabelle in ARNOLD/HOFF, Fortschritte in der Behandlung der endogenen Psychosen (1961), S. 507 bzw. jene in ARNOLD, Die Therapie der Schizophrenie (1963), S. 168f., abgebildet oben S. 204f.

[1150] Vgl. oben S. 193 Als Erleichterung für die PatientInnen wurde in den letzten Jahren der ‚Ära Hoff', um das Koma abzubrechen, nicht mehr eine Zuckerlösung über die Nasensonde eingeflößt, sondern Glucagon i. m. injizierten, worauf sie erwachten und trinken konnten (vgl. oben S. 194).

Arnold, der viel zu den „in den letzten vier Jahren erzielten Fortschritten“[1151] in der Diagnostik, Indikation sowie zur Technik der Anwendung beigetragen hatte und vor allem zur Schizophrenie publizierte, betonte zum „Gesamtbehandlungsplan“: je nach Art der Schizophrenie und je nach Prozessverlauf müssten andere konvulsive Therapien (Cardiazol-, Elektrokrampftherapie), psychochirurgische Methoden (Lobotomie), Fiebertherapie (Malariatherapie), die Behandlung mit Psychopharmaka und mit psychotherapeutischen Methoden (Einzel- und Gruppentherapien), „komplettierende Methoden“ (Arbeits- und Sporttherapie, Gymnastik-, Tanz- und Musiktherapie) sowie „Soziotherapie“ integriert werden.[1152] Klar blieb jedoch für ihn, wie er 1960 schrieb, dass „auch heute noch […] die richtig indizierte und geführte Insulinvollschockkur die Basisbehandlung der Schizophrenie darstellt.“[1153]

Die Neuroleptika, die an der Wiener Klinik von Anfang an (ab 1952/53) verwendet wurden, ohne die Insulinkur völlig zu ersetzen,[1154] führten aber doch zu einer kontinuierlich abfallenden, deutlich selteneren Anwendung der Insulinkomatherapie im Untersuchungszeitraum: von 95 Insulinkuren 1951, über 58 1959 und 43 1960, zu 16 1968.[1155]

Die Wirkung der Insulinkur wurde mehrmals bezweifelt bzw. als sehr gering angenommen, von anderen jedoch auch in den 1960er Jahren noch für unverzichtbar gehalten.[1156] Im Vergleichsbeispiel Zürich ging ihre Anwendung – ebenso wie jene der Malariatherapie – Ende der 1950er Jahre zu Ende, in Wien erst um 1970, nach dem Ende der „Ära Hoff“, und in anderen Ländern noch deutlich später.[1157] Zur langen Anwendung der Insulinkomatherapie in

[1151] ARNOLD, Die körperlichen Behandlungsmethoden der Schizophrenie (1960), S. 262.
[1152] Zum „Gesamtbehandlungsplan“, nachdem vorher die verschiedenen Therapien beschrieben wurde, ARNOLD, Die Therapie der Schizophrenie (1963), S. 133-198.
[1153] ARNOLD, Die körperlichen Behandlungsmethoden der Schizophrenie (1960), S. 262.
[1154] Vgl. dazu Hoffs Ausführungen im Oktober 1955 bei der von Jean Delay geleiteten Pariser Tagung zu Chlorpromazin (Largactil) oben S. 274-276.
[1155] Vgl. das Balkendiagramm Abb. 8 oben S. 211.
[1156] So auch das französische Standardhandbuch von EY/BERNARD/BRISSET, Manuel de Psychiatrie (^{1}1960), S. 936f.
[1157] HIRSCHMÜLLER, Die Insulinkomabehandlung der Schizophrenie (2001), S. 224f.: Nach Hirschmüller kam man im Westen mit Beginn der 1960er Jahre mehrheitlich von der Insulinkomatherapie ab, während in der DDR [möglicherweise aus Mangel an Neuroleptika, GH], in Japan und ganz besonders in Russland ihre Anwendung noch bis in die 1980er Jahre häufig war. Nach der oben zitierten französischen Umfrage von 1980 wandten „unter 78 französischen Psychiatern 10 und unter 28 nichtfranzösischen Psychiatern einer nach wie vor in gewissen Fällen von Hebephrenie und von Schizophrenie mit Anorexie die Insulinbehandlung an“. Zur sehr positiven Aufnahme der Therapie in den USA vgl. DOROSHOW, Performing a Cure for Schizophrenia: Insulin Coma Therapy on the Wards (2006), S. 213.

Wien dürfte – wie bei der Malariatherapie – die Erfahrung des Klinikchefs Hans Hoff beigetragen haben, der an der Entwicklung der Insulinkomatherapie in den 1930er Jahren beteiligt gewesen war.[1158]

3.3 Die Malariafiebertherapie

Die Malariafiebertherapie war an den Erwachsenenstationen[1159] der Wiener Klinik die dritthäufigste der ‚großen alten Kuren' der Zwischenkriegszeit. In der Datenbank sind 772 (bzw. 869[1160]) Fälle ihrer Anwendung aufgenommen. Da sie nur in Einzelfällen PatientInnen zweimal gegeben wurde, entspricht die Fallzahl weitgehend der Zahl der PatientInnen (wie bei der Komatherapie, anders als bei den Krampftherapien). Sie erreichte 1959 einen Tiefststand, stieg jedoch in 1960er Jahren wieder auf eine Zahl zwischen 35 und 45 Anwendungen. Die letzte Überimpfung war Mitte Dezember 1968,[1161] also kurz vor dem Ende der „Ära Hoff" – Hans Hoff starb noch aktiv im Dienst am 23. August 1969.

Die Malariafiebertherapie wurde in dieser Zeit bei Neurolues (vor allem bei progressiver Paralyse) meistens gemeinsam mit einer Penicillintherapie angewandt. In der internationalen zeitgenössischen Diskussion von den 1940er Jahren bis in die 1960er Jahre ging es darum, ob diese Kombination gleiche oder bessere Erfolge bringen würde als Penicillin alleine. Wiener KlinikerInnen, aber weltweit auch andere PsychiaterInnen waren der Meinung, dass die Kombination zu besseren klinischen Ergebnissen führt. Schließlich setzte sich die Behandlung der Neurolues mit Penicillin durch: Bei vielen ÄrztInnen hatte sich die Meinung durchgesetzt, dass die Ergebnisse bei der einfacheren und schonenderen Therapie nur mit Penicillin gleich gut seien wie bei der Kombination. Entscheidend war aber auch, dass durch die Ausheilung

[1158] Vgl. PÖTZL, Widmung an Hans Hoff (1958), S. 5-14. Pötzl schreibt in diesem Festschriftartikel von einer Mitarbeit Hoffs an der Insulinkomatherapie. Vgl. dazu die Publikation HOFF, Hypoglykämie-Schockbehandlung von Psychosen (1936), S. 917f.

[1159] Die 35 überlieferten Fälle, in denen eine Malariafiebertherapie an der Kinderstation angewandt wurde (letzter Fall 1965), hatten Gehirnschädigungen aufgrund von Geburtstraumata, Encephalitis, postnatalem Icterus und Diagnosen aus dem Diagnosefeld der ‚Intelligenzmängel'; nur zwei Patientinnen hatten die klassische Anwendung bei juveniler progressiver Paralyse: vgl. die Tabelle in GEIGER, Kinderstation (2015), S. 253f., zitiert oben S. 92 Anm. 322. Abgesehen von den letztgenannten beiden Fällen fielen die Anwendung in das diagnostische Feld, in dem deutlich mehr als der Hälfte PrivatpatientInnen waren, wovon die meisten aus dem Ausland kamen und zu dem die einzige umfangreichere zeitgenössische Wiener Publikation gefunden wurde: KUNDRATITZ, Die therapeutische Beeinflußbarkeit zerebralgestörter Kinder (1957), S. 423-427 (vgl. oben S. 91f.). Karl Kundratitz war damals Leiter der Wiener Universitätskinderklinik.

[1160] Vgl. dazu oben S. 37.

[1161] Der 21jährige Patient X872 mit der (außerhalb der Einschlusskriterien stehenden) Diagnose „Medikamentenüberdosierung, neurotische Reaktion".

der Syphilis mit Penicillin in früheren Stadien die Neurolues kaum noch vorkam und deshalb die Aufrechterhaltung eines *Malaria tertiana* Stamms durch Überimpfung von einem / einer ParalytikerIn auf den / die nächste/n – wie an vielen Kliniken üblich – nicht mehr möglich war.[1162] Ein Bedauern über den Mangel an geeignetem mit Malariaparasiten infiziertem Blut zur Behandlung der progressiven Paralyse findet sich mehrmals in den Fachpublikationen.[1163] In Wien hingegen konnte der *Malaria tertiana* Stamm durch die häufige Symptom- bzw. Zustand-bezogene Anwendung unter Vernachlässigung von diagnostischen Kategoriengrenzen, d. h. auch bei den Diagnosen aus dem Feld der Intelligenzmängel, schizophrenen und affektiven Erkrankungen sowie Psychopathie, bis 1968 mit einer kurzen Unterbrechung 1959 aufrechterhalten werden.

Zur Indikation der Malariafieberkur bei den vier Diagnosen außerhalb der Neurolues (Intelligenzmängel, schizophrene und affektive Erkrankungen, Psychopathie) gab es keine ausführliche Diskussion in der zeitgenössischen Literatur: Hinweise auf ihre Anwendung bei diesen Diagnosen waren in den eingesehenen Publikationen selten bzw. – im Fall der „Psychopathie" – gar nicht zu finden. Diese breite Anwendung, für die es in der Literatur nur in einer Wiener Publikation eine ausführliche Ableitung gab, dürfte in den beiden Jahrzehnten im deutschen Sprachraum abgesehen von Wien kaum praktiziert worden sein:[1164] weder in einer der eingesehenen historischen Untersuchungen zu psychiatrischen Kliniken und Krankenhäusern in der Nachkriegszeit, noch in der Erinnerung von Zeitzeugen, die an anderen Kliniken arbeiteten, fanden sich Hinweise darauf. Einem Hinweis eines Zeitzeugen auf das Vorhandensein des Stamms und auf die Anwendung der Malariafiebertherapie bei Diagnosen außerhalb der progressiven Paralyse als *ultiama ratio* an der Pariser Salpêtrière Anfang der 1960er Jahre[1165] konnte im Rahmen des Projekts in der französischen Literatur nicht weiter nachgegangen werden, auch nicht dem Hinweis des rumänischen Malariologen G. Lupascu (1974) auf die Zentren «d'impaludation thérapeutique» in Socola-Jassy in Rumänien und in Chamblee-Atlanta in den USA.[1166] Der renommierte Züricher Psychiater Manfred Bleuler definierte jedoch die „Schock- und Fiebertherapien", zu denen die

---

[1162] Vgl. oben S. 66 Anm. 204.
[1163] Z.B. in *The Lancet*, COLLINS, Insulin Therapy (Letters to the editor) (1961/II), S. 1457, zitiert oben S. 179.
[1164] Vgl. die ausführliche Ableitung durch KUNDRATITZ, Die therapeutische Beeinflußbarkeit zerebralgestörter Kinder (1957), S. 423-427. Vgl. auch die Nennung als eine der „letzte[n] therapeutische[n] Reserve[n]" in der klinischen(!) Anwendung in der Kinder- und Jugendpsychiatrie: MÜLLER-KÜPPERS, Die Therapie im Kindes- und Jugendalter (1972), S. 997f., zitiert oben S. 93 Anm. 325.
[1165] Vgl. oben S. 81f.
[1166] Vgl. oben S. 169 Anm. 628.

Malariafiebertherapie gezählt wurde, als in der Praxis zur symptomatischen Behandlung nützliche „Erschütterungstherapien“, brauchbar für „alle psychopatischen Zustände, die noch nicht kausal angegangen werden können“.[1167] Das war vor der Einführung der neuen Psychopharmaka. Aber auch danach finden sich im deutschsprachigen Standardlehrbuch zur Psychiatrie von Eugen Bleuler, in den von Manfred Bleuler überarbeiteten Auflagen von 1960 und 1969, Hinweise zu den – nach der damaligen Diktion – ‚Schock- und Fieberkuren‘, die in diese Richtung gehen und die dafür sprechen, dass die Malariafiebertherapie als eine der „’großen’ körperlichen Behandlungsverfahren“ bei den nichtluetischen Diagnosen, bei denen sie in Wien in der „Ära Hoff“ angewandt wurde, nach Bleuler auch noch in den 1960er Jahren als möglich und zulässig angesehen wurde.[1168]

Die Indikationsentscheidung für die ‚unspezifisch wirkenden‘ körperlichen Therapien erfolgte im Allgemeinen – und ganz offensichtlich[1169] bei Erkrankungen aus den vier ausgewählten Diagnosefeldern außerhalb der Neurolues, die in dieser Studie behandelt werden – nach der Beurteilung der Symptome und Syndrome, der syndromatologischen Gestalt der Krankheit aus der Beobachtung des Zustands der Kranken und ihrer Entwicklung und nicht nach der – im Krankenakt oft erst kurz vor der Ablage eingetragenen – „Diagnose“ (dem nosologischen Kategorie-Begriff).[1170] Die hypothetischen Schlussfolgerungen zu den Zielen der Anwendungen der Malariafiebertherapie konnten deshalb nicht alleine auf die Diagnosen in der Diagnosezeile des Krankenakts bezogen werden, zu denen sie in der eingesehenen Literatur selten und fast immer nur in kurzen Erwähnungen (bzw. zur „Psychopathie“ gar nicht) genannt wurde, sondern vor allem auf die Symptome und Symptomgruppen, die wiederum Diagnosefelder übergreifend genannt wurden.

Dass die Wiener Klinik in den deutschsprachigen Ländern in den 1960er Jahren in der Anwendung der Malariafiebertherapie außerhalb der progressiven Paralyse vermutlich alleine

---

[1167] BLEULER, Forschungen zur Schizophreniefrage (1948), S. 145-147.
[1168] Vgl. oben S. 28-33 die Zitate aus den überarbeiteten Auflagen des deutschsprachigen Standard-Lehrbuchs der Psychiatrie von 1960 und 1969. Zulässig freilich nur in einer therapeutischen Anwendung: vgl. oben S. 159f. und S. 168f.
[1169] So brachte die Frage nach der Indikationsentscheidungen für oder gegen eine dieser Therapien nur auf der Basis der Krankheitseinheiten (nosologischen Kategorien, der „Diagnosen“ in der Diagnosezeile am Krankenakt) keine Lösung.
[1170] Vgl. etwa die Ausführung von Walter SPIEL, Die Therapie in der Kinder- und Jugendpsychiatrie (1967), S. 132f. zur Indikation der Elektrokrampftherapie (EKT) eingeschränkt „im Rahmen dieser beiden Formenkreise [des schizophrenen wie des manisch-depressiven] nur für spezifische Ausformungen der Psychose“, die oben S. 258 zitiert wird.

stand, wirft die Frage auf, ob die Therapie in diesen Fällen damals dem *state of the art* entsprach. Diese Frage ist für diese Zeit nicht eindeutig zu beantworten, denn: Die Anwendung erfolgte aufgrund von positiven Erfahrungen seit den 1920er Jahren. Auch wenn die Malariafiebertherapie kaum an einer anderen Klinik bei diesen Diagnosen angewandt wurde, so war im Untersuchungszeitraum vermutlich wenig gegen die schon in der Zwischenkriegszeit große „Bereitschaft zum ‚off-label-use'"[1171] einzuwenden. Das kann aufgrund der damaligen Akzeptanz einer therapeutischen Vielfalt in der klinischen Praxis angenommen werden, wofür zur Insulinkur Beispiele aus Bleulers Lehrbuch und aus einer französischen Umfrage zitiert wurden.[1172] Es war eine Zeit, in der gegen eine Standardisierung der Therapien in der Psychiatrie geäußert wurde, dass in jedem Einzelfall nach den Besonderheiten der Symptome und des Krankheitsverlaufs die Therapie zu entscheiden sei: es sei nicht der Fall „bestimmten Methoden, sondern umgekehrt diese dem Fall anzupassen".[1173] Es war eine Zeit, in der man sich zwar intensiv um eine Standardisierung des diagnostischen Prozesses, der Diagnosen und ihrer Behandlung bemühte, darüber aber noch zu keiner allgemeine Übereinkunft gekommen war.[1174] Daraus ergab sich eine sehr unterschiedliche Praxis auf der Basis der verschiedenen Schulen und Traditionen sowie der unterschiedlichen individuellen Erfahrungen und Einschätzungen. Nicht eine international geprüfte, diskutierte und anerkannte Therapie bei einer ebenso in der internationalen Fachgemeinschaft fixierten Diagnose gab den Ausschlag, sondern die persönliche Einschätzung, was ‚das Beste für den Patienten/die Patientin' sei: In Wien war das Ergebnis, wie immer wieder betont wurde, aufgrund der angenommenen multifaktoriellen Genese der psychischen Krankheiten ein differenzierter, auf den individuellen Fall abgestimmter „Gesamtbehandlungsplan".

Die positiven Erfahrungen, die in der Zwischenkriegszeit in der Anwendung der Malariafiebertherapie bei Psychosen gemacht worden waren,[1175] wirkten noch nach und ebenso jene mit ihrer Anwendung bei Persönlichkeitsstörungen, sei es bei ‚Intelligenzmängeln' (aufgrund von Encephalitiden), sei es ‚zur Nachreifung' bei den Diagnosen aus dem Bereich ‚Psychopathie'. Die Erfahrungen der Generation von Hans Hoff

[1171] Vgl. dazu SCHMUHL/ROELCKE, Einleitung (2013), S. 23f., oben S. 78 und S. 166.
[1172] Vgl. zu Bleuler oben S. 30-33, sowie zur französischen Umfrage oben S. 187f.
[1173] SPIEL, Die Therapie in der Kinder- und Jugendpsychiatrie (1967), S. VIII f.
[1174] Der ICD-8, die breit akzeptiert Version der internationalen statistischen Klassifikation der Krankheiten, erschien 1965, die Therapie-Guidelines in den 1980er und 1990er Jahre.
[1175] HELMCHEN, Malariaimpftherapie der Progressiven Paralyse in den Wittenauer Heilstätten (1999), S. 444f.

und seinem Vorgänger Otto Kauders mit der erfolgreichen und mit dem Nobelpreis für Julius Wagner-Jauregg 1927 ausgezeichneten ‚Wiener‘ Therapie, an deren Entwicklung sie teilgenommen hatten, kann als mitentscheidend angenommen werden für die ausnehmend breite[1176] und – wie bei der Insulinkomatherapie - lange Anwendung der Malariafiebertherapie an der Wiener Psychiatrie, die aus dieser Tradition international auch in den 1950er Jahren noch einen guten Ruf hatte.[1177]

Durch die breite Anwendung der Malariefiebertherapie außerhalb der Neurolues konnte der *Malaria tertiana* Stamm an der Wiener Klinik aufrechterhalten werden. Die Voraussetzung für die Anwendung der Malariatherapie bei diesen Diagnosen, das Vorhandensein eines („luesfreien“) *Malaria tertiana* Stamms durch regelmäßige Überimpfung, dürfte in dieser Zeit nirgends sonst in Österreich, der Schweiz und der Bundesrepublik Deutschland gegeben gewesen sein.[1178] Der Grund für die ‚Pflege’ des Stamms konnte – nach der radikalen Verminderung der Fälle von Neurolues[1179] und der Möglichkeit, diese gut mit Penicillin alleine zu behandeln – nur gewesen sein, dass von diesen Anwendungen außerhalb der Neurolues aus den Erfahrungen seit den 1920er Jahren aufgrund der individuellen Beurteilung des Krankheitsfalls ein therapeutischer Erfolg erwartet wurde. Die spärlichen Informationen zur Beurteilung der Indikation im Einzelfall in den PatientInnenakten und auch die Ergänzungen in der Literatur reichen jedoch nicht aus, um die spezifische Beurteilung nachvollziehen zu können, die für bzw. gegen die Indikation der Malariatherapie im Einzelfall entschied.

Warum aber wurde die Malariafiebertherapie noch in einer Zeit angewandt, in der etwa ‚Beruhigung‘ und ‚Umstimmung‘ mit den neuen Medikamenten für möglich gehalten wurden?[1180] Die Malariafiebertherapie wurde bei den Diagnosen außerhalb der Neurolues als

[1176] Die Malariafiebertherapie wurde auch in anderen Fachdisziplinen angewandt, so in der Dermatologie und in den 1950er Jahren noch in der physikalischen Medizin in der Nachbehandlung der Kinderlähmung nach Kauders; vgl. oben S. 78f.
[1177] Zeichen für die internationale Anerkennung waren etwa die Platzierung internationaler Tagungen in Wien und die Einladung des Nobel-Komitees an Hans Hoff, Vorschläge für den Nobelpreis für Physiologie und Medizin 1953 bzw. 1958 zu präsentieren.
[1178] Ob es in anderen Ländern diese Voraussetzung gab, dazu waren genauere Hinweise aus Pezinok bei Bratislava oder aus Budapest nicht zu bekommen und zu den Zentren « d’impaludation thérapeutique. » in Socola-Jassy (Rumänien) und in Chamblee-Atlanta (USA), die LUPASCU, Applications actuelles de la malariathérapie (1974), S. 167 als noch aktiv in der Anwendung der Malariafiebertherapie nannte, wurde im Rahmen des Projekts kein Kontakt hergestellt.
[1179] Vgl. die Zahlen oben bei Abb. 2 S. 74.
[1180] Der rumänische Tropenmediziner Lupascu hielt zwar 1974 die Anwendung der Malariafiebertherapie bei nichtluetischen PatientInnen seit der Einführung der neuen Psychopharmaka für überholt, schlug sie im Interesse

alte ‚erprobte' Indikationen weitergeführt;[1181] sie wurde zu den „Schock-" bzw. „Erschütterungstherapien" gezählt, deren Wirkungen noch in der zeitgenössischen Literatur positiv beschrieben wurden;[1182] mehrere Ziele wie Beruhigung, Umstimmung oder Reifung wurden in den Krankenakten übergreifend bei allen vier Diagnosen genannt. Damit liegt die Hypothese nahe, dass die Fiebertherapie häufig statt der neuen Medikamente gegeben wurde, da von der Malariafiebertherapie gleiche, vielleicht auch bessere Ergebnisse als von diesen (mit ihren starken Nebenwirkungen) erwartet wurden und da damit zugleich ein luesfreier *Malaria tertiana* Stamm für Behandlungen in diesen und anderen Krankheitsfällen[1183] aufrechtzuerhalten war.

Der Frage, ob in einigen Fällen nur die Notwendigkeit, den Malariastamm durch Überimpfung zu erhalten, zu dieser Entscheidung führte, wurde in einem Exkurs zu Patienten zu klären versucht, die „als Stammträger von Steinhof rücktransferiert" wurden und bei denen diese oder eine ähnliche Wortwahl im Krankenakt eine Interpretation als nichttherapeutische Anwendung nahelegt.[1184] Eine Anwendung der Malariatherapie nur zur Aufrechterhaltung des Malaria-Tertiana-Stamms konnte zwar auch in diesen Fällen nicht völlig geklärt und bewiesen werden (und das dürfte auch in der Konkretisierung auf Einzelfälle so bleiben), der Eindruck festigte sich jedoch – auch mit einzelnen Beispielen vor 1950 aus anderen Kliniken –, dass die Anwendung manchmal nicht therapeutisch war, sondern ausschließlich zur Erhaltung des Stamms der *Malaria tertiana* diente.[1185]

3.4 Ergebnisse, offene Fragen und Forschungsdesiderata

Publikationen der beiden leitenden Wiener Kliniker Hoff und Arnold zeigen, wie stark sie die verschiedenen therapeutischen Methoden in den „Gesamtbehandlungsplan" einbezogen. Nicht nur die somatischen Therapien der Zwischenkriegszeit und der neuen Psychopharmaka waren vorgesehen, sondern auch Psychotherapie und Therapien, die wie die Arbeitstherapie die

---

der Malariaforschung aber dennoch vor; vgl. oben S. 82. Malariamedikamente wurden nicht in Wien, aber anderswo an Malariafiebertherapie-PatientInnen erprobt: vgl. oben S. 169 Anm. 628 und 630.

[1181] Auch vor 1950 wurde in Wien ein luesfreier Malariastamm verwendet, die Therapie also regelmäßig bei PatientInnen ohne Neurolues angewandt.

[1182] Etwa im Bleuler'schen Lehrbuch, vgl. oben S. 28-30.

[1183] Vgl. etwa oben S. 78f. zur Nachbehandlung der Kinderlähmung nach Kauders.

[1184] Oben S. 150-161 der Exkurs zu „Stammträger" und die weiteren Ausführungen dazu S. 167-170.

[1185] Vgl. oben S. 7 zur Entschuldigung für eine nichttherapeutische Verwendung von Patienten als „Stammträger", die Univ. Prof. Dr. Johannes Wancata im Namen der Klinik und in Absprache mit dem Projektleiter und dem Rektorat bei der Pressekonferenz des Rektorats der MedUni zum Projektendbericht 2015 vorbrachte.

soziale Reintegration der PatientInnen erleichtern sollten.[1186] Psychotherapie (Gruppentherapie) findet sich nur selten in den Krankenakten vermerkt, was vermutlich weniger auf eine Diskrepanz von Theorie und Praxis, sondern auf eine ungenaue Dokumentation schließen lässt. Die Mangelhaftigkeit der Krankenakten resultiert wohl vor allem aus der Überlastung der Klinik als Clearingstelle, als Verteiler- und „Durchgangsstation" für ca. zwei Drittel der aufgenommenen PatientInnen.[1187]

In der zeitgenössischen Literatur wurde mehrmals beklagt, dass die Wirkmechanismen der in der Zwischenkriegszeit entwickelten ‚großen' körperlichen Kuren noch immer zu unklar seien, um daraus die Indikation abzuleiten.[1188] Obwohl die Wirkzusammenhänge strittig blieben und die stark invasiven Therapien auch gefährlich waren, kam es rasch weltweit zu ihrer Anwendung.[1189] Die klinische Erfahrung bestimmte die Erwartungen in die Therapie und ihre (breiten) Anwendungen.[1190] Als Erfolge der ‚großen' alten Kuren (bei mehreren der Diagnosen) werden in den Krankengeschichten bzw. in den zeitgenössischen Publikationen genannt:

- „Unterbrechung des circulus vitiosus von Stimulus und Erregung";[1191]
- Beruhigung einer aufgrund ihrer Krankheit immer wieder heftig erregten und aggressiven Person;[1192]
- Anstoß zu einer positiven Persönlichkeitsentwicklung (Reifung);
- Öffnung der verschlossenen PatientenInnen für eine Psychotherapie durch die erhöhte Zuwendung des Pflegepersonals während der Therapie und / oder durch die „original life experience", die beim Erwachen aus dem Koma erlebt würde.[1193]

[1186] Vgl. die Tabelle in ARNOLD/HOFF, Fortschritte in der Behandlung der endogenen Psychosen (1961), S. 507 bzw. jene in ARNOLD, Die Therapie der Schizophrenie (1963), S. 168f., abgebildet oben S. **204f**. Vgl. auch die Bedeutung, die der Verbesserung der Atmosphäre durch eine bauliche Gestaltung beigemessen wurde, oben S. 203.
[1187] Vgl. oben S. 14 und S. 20.
[1188] Z.B. Pierre Pichot 1960, vgl. oben S. 182f.
[1189] SCHMUHL/ROELCKE, Einleitung (2013), S. 22; vgl. hier die Beispiele zur Unklarheit über die Wirkweise, die eben dadurch nach den beiden Autoren „mit den unterschiedlichsten Konzepten der Ätiologie psychischer Erkrankungen kompatibel" waren und so zum Erfolg beitrugen.
[1190] Ebd., S. 20f., indem die beiden Herausgeber die Argumentation im Sammelband-Beitrag von BORCK, Die Internationale der invasiven Therapien (2013), S. 136f. aufgreifen; vgl. oben S. 173f.
[1191] SCHMUHL/ROELCKE, Einleitung (2013), S. 22; vgl. dazu und zu anderen Wirkungen der „Erschütterungstherapien" die Zitate aus den Bleuler'schen Lehrbüchern von 1960 und 1969, oben S. 28f.
[1192] Aber auch „die ‘sedative Therapie‘ mit unterschwelligen Schlafmitteln, die Insulin-Subschocktherapie und wohl auch die Insulin-Therapie mit Komadosen für die [verkürzte] Dauer […] bis etwa zum 15. Insulinschock" als “bewußtseinsdämpfende Therapien" um die „Wahnfixierung", die dritte der vier „Persönlichkeitsabwandlungen" nach Raoul Schindler, zu stabilisieren: vgl. oben S. 190f.
[1193] Vgl. oben S. 185f.

Es sind Symptome bzw. Syndrome, die mit dem Ziel der sozialen (Re-)Integration mit den ‚großen alten Kuren' behandelt wurden: um die Ansprechbarkeit, die soziale Eingliederung in den Klinikalltag und schließlich in den familiären und beruflichen Alltag zu ermöglichen. Die Fieber-, Krampf- und Komatherapien waren dabei Teil eines ‚Gesamtbehandlungsplans', gemeinsam mit medikamentösen Therapien, mit Psycho- und Arbeitstherapie. Sie waren ‚unspezifische', nicht ‚kausal' wirkende Therapien, anders als die Malariafiebertherapie bei progressiver Paralyse und die Insulinkomatherapie, die an der Wiener Klinik als ‚spezifische Therapie' bei früher Schizophrenie galt.[1194]

Der Untersuchungszeitraum als Periode des Übergangs, wie er im Projekt mit den abnehmenden Anwendungen der in der Zwischenkriegszeit entwickelten „Schock- und Fieberkuren" zugunsten von Penicillin bzw. Psychopharmaka deutlich wird, zeigt sich auch in einigen Veränderungen bei den Diagnosen: Die Abnahme des Anteils der Schizophrenien an der Gesamtheit der Aufnahmen im Diagnosebereich der Projektstudie – von etwas über 50% auf deutlich unter 40% – ist in Zusammenhang mit der Zunahme der Diagnosen der affektiven Störungen (vor allem der Depressionen) zu sehen. Eine Erklärung für die unterschiedliche Diagnosehäufigkeiten im Untersuchungszeitraum ist, dass in diesen beiden Jahrzehnten die traditionellen Diagnostiziergepflogenheiten kritisiert wurden und diese Kritik einerseits zu Veränderungen diagnostischer Systeme und ihrer breiten Akzeptanz (z.B. ICD-8 von 1965), andererseits zu einer zunehmend anderen Gewichtung von Symptomgruppen[1195] im diagnostischen Prozess führte: Nach einer Dominanz ‚schizophrener' (produktiver) Symptome, sodass zum Teil andere, d.h. affektive Symptome gar nicht mehr erhoben wurden, führte diese Entwicklung zu einer Dominanz affektiver über ‚schizophrenen' Symptomen, sodass solche Erkrankungen nicht mehr als ‚Schizophrenie' sondern z.B. als Legierungspsychose – der Mischdiagnose einer schizophrenen und affektiven Erkrankung – oder überhaupt nun als affektive Psychose mit produktiver Symptomatik diagnostiziert wurden und werden.[1196]

[1194] Vgl. oben S. 189, S. 197 und S. 202; kausal heilend S. 282.
[1195] Der ICD-8 und seine Nachfolger hatten zu einem guten Teil durch die Standardisierung auf der syndromatologischen Ebene Erfolg. Ich danke Eberhard Gabriel für diesen Hinweis.
[1196] Vgl. oben S. 213f. zur Diagnose „Hebephrenie", die in den 1960er Jahren deutlich abnimmt. Zum Begriff „produktive" bzw. „plus" Symptomatik vgl. oben S. 273 Anm. 1023.

Nicht nur für die Änderungen in der Diagnoseerstellung, sondern auch für den Einfluss von Geschlecht auf die Diagnoseerstellung und von sozialer Zugehörigkeit auf die Therapie ergaben sich einzelne Hinweise, die informativ für weiterführenden Untersuchungen sein könnten. So wurden Frauen ohne Neurolues kaum mit einer Malariafiebertherapie[1197] behandelt und häufiger als Männer mit einer EKT.[1198] Der Grund für diesen Unterschied ist aus den PatientInnenakten nicht ersichtlich. Zur sozialen Indikation wurden Auswertungen der Akten von 1955 – 1960 in der Pilotstudie zu Hinweisen auf die Indikationen bei HilfsarbeiterInnen und Angestellten bzw. bei PatientInnen der Privatstation und der allgemeinen Station herangezogen.[1199]

Die Frage, die man sich retrospektiv in Bezug auf die ‚alten Kuren' stellen muss, ist die nach der Angemessenheit der Indikation in ihrer Zeit. Damals dürfte die Meinung weit verbreitet gewesen sein, dass der chronische Verlauf vieler Krankheitszustände in der Psychiatrie und die damit verbundenen schweren Beeinträchtigungen sowohl individueller als auch sozialer Lebensvollzüge auch die Anwendung stark invasiver Verfahren rechtfertigte.[1200] Die ärztliche Entscheidung für eine Therapie, für die die persönlichen Erfahrungen bzw. die Schultraditionen des Standorts den Ausschlag gaben,[1201] wurden in der Fachgemeinschaft zwar kritisiert, aber doch grundsätzlich akzeptiert, wie die Zitate aus dem Bleuler'schen Lehrbuch (1960 und 1969) oder die Antworten auf die französische Umfrage zu den beiden Insulinkuren von Ende der 1970er Jahre zeigen.[1202]

[1197] Vgl. oben S. 62f. und S. 164 Anm. 605.
[1198] Vgl. oben S. 268-270.
[1199] Vgl. oben S. 43-47.
[1200] So der damalige Leiter der Klinik Otto Pötzl, der die zentrale Beteiligung Hoffs bei der Entwicklung der Komakur hervorhebt: vgl. oben S. 173 Anm. 649. KRAGH, Malaria, Sulfosin and Metallosal in the Treatment of Mental Disorders in Denmark (2013), S. 108 zitiert den dänischen Psychiater Paul Reiter, der trotz der starken und gefährlichen Nebenwirkungen bei Impfungen von schizophren Erkrankten mit Coli-Bakterien meinte, es habe doch auch sehr gute Resultate gegeben und es sollten zusätzliche Versuche gemacht werden, denn Schizophrenie sei „an illnes of such great sozial significance that any treatment giving even the smalest hope could not be ignored." SCHMUHL/ROELCKE, Einleitung (2013), S. 23 bringen mehrere Beispiele ähnlicher Aussagen, die als „Hintergrund […] eine Auffassung erkennbar [werden lassen], die schwere psychische Erkrankungen, etwa die Progressive Paralyse oder eine fortgeschrittene Schizophrenie, mit lebensbedrohlichen körperlichen Krankheiten wie einem Krebsleiden mit infauster Prognose gleichsetzte."
[1201] Vgl. RZESNITZEK, „Schocktherapien" und Leukotomie in der DDR-Psychiatrie (2018), S. 297: „Das Therapierepertoire in der DDR unterschied sich von Klinik zu Klinik beträchtlich. […] Die Unterschiede waren dabei maßgeblich durch die individuelle Einstellung des Klinikdirektors bedingt, genau wie es auch für die Kliniken in der Bundesrepublik Deutschland nachweisbar ist."
[1202] Vgl. die Zitate der französischen Umfrage (oben S. 187f.).

Es war auch die Zeit einer uneinheitlichen, nicht normierten Diagnostik vor einer breiten Anerkennung des ICD-8 (1965) und eine Zeit noch lange bevor Therapie-Guidelines der Fachdisziplinen herausgegeben wurden. Auch wurde international über medizinische Ethik diskutiert,[1203] das führte jedoch in Wien erst 1978 zur Gründung der Vorgängerin der Ethikkommission. Das Fehlen von einheitlichen und breit anerkannten Regeln in diesen beiden Bereichen dürfte subjektive, vom Arzt / von der Ärztin geprägte Therapieentscheidungen erleichtert haben, und – besonders in autoritär geführten Kliniken wie in Wien unter Hans Hoff – Entscheidungen im Sinne einer Schultradition, die in Wien durch Hoffs Lehrer Julius Wagner-Jauregg (1857-1940) und Otto Pötzl (1877-1962) geprägt war.

Die Krankengeschichten als Hauptquelle erwiesen sich nur zum Teil als informativ: Mit ihrer Aufnahme in eine Datenbank konnte die Häufigkeit der Anwendung, ihrer Kombinationen und als Vergleichsdaten auch die Nichtanwendungen bei den fünf Diagnosen und ihren Unterdiagnosen festgestellt werden und das auch im Jahres- und Altersraster, sowie im Zahlenverhältnis der Patientinnen und der Patienten. Die Informationen aus den Fachpublikationen zur Technik der Anwendung konnten aufgrund der Krankenakten konkretisiert werden. Die rasche Aufnahme der neuen medikamentösen Therapien war zu dokumentieren und auch, dass sie in diesen beiden Jahrzehnten an der Wiener Klinik die ‚großen' alten Kuren nur zum Teil und nicht völlig ersetzten.

Aus den PatientInnenakten und auch in Kombination mit den Wiener Publikationen der Zeit konnte jedoch nur bruchstückhaft abgeleitet werden, warum jeweils im Einzelfall auf die beschreibbare Weise therapiert wurde, d. h. die Indikationsstellung war aufgrund der PatientInnenakten nur sehr mangelhaft zu rekonstruieren. So insbesondere bei der Malariafiebertherapie, zu der es, abgesehen zu ihrer ‚klassischen' Anwendung bei der progressiven Paralyse, in Publikationen der Wiener KlinikerInnen der Zeit nur wenige kurze Hinweise gibt. Bei der „Psychopathie" steht sie sogar im Widerspruch zu ihrer Anwendung, indem in den Publikationen die Malariafiebertherapie nie genannt, in der Praxis aber häufig gegeben wurde. Hier mussten die Hypothesen aus den erwähnten Zielen ‚Beruhigung',

[1203] Vgl. 1964 die Deklaration von Helsinki des Weltärztebundes zu „Ethischen Grundsätzen für die medizinische Forschung am Menschen".

‚Umstimmung' und ‚Reifung' abgeleitet werden – aus Zielen, die auch bei den anderen Diagnosen zur Anwendung der Malariafiebertherapie (symptombezogen) genannt wurden.

Die Einschränkung des Forschungsprojekts auf die von MedizinerInnen produzierten Quellen (Diagnosen, Anamnesen, Entlassungsschreiben etc. in den PatientInnenakten, Fachpublikationen, mündliche und schriftliche Informationen von Ärzten als Zeitzeugen) war für die Fragestellung in der Planung und dann in der Ausführung maßgebend. Es ging um die Sicht der der ÄrztInnen in den beiden Jahrzehnten.[1204]

Aufgrund des Zeitrahmens waren Erweiterungen nicht möglich: so wurde eine Recherche nach Malariafiebertherapiefällen an der von Karl Kundratitz in den 1950er Jahren geleiteten Kinderklinik[1205] nach Maßgabe der Zeit noch im Projektantrag als wünschenswert angeführt; aus PatientInnenakten der *Heil- und Pflegeanstalt Steinhof* wären vielleicht noch weitere, klärende Informationen zu einzelnen PatientInnen zu bekommen gewesen (etwa zu den 28 Patienten die im Exkurs zur Stammträgerproblematik genannt werden und vom Steinhof zur Malariafiebertherapie an die Klinik rücküberwiesen wurden); durch eine intensivere Recherche in französischen Fachzeitschriften der Zeit hätten vermutlich die Hinweise in den Publikationen und Zeitzeugenberichten, aus denen zitiert wurde, ergänzt werden können.

Viele Desiderata mussten für künftige Forschungen offenbleiben, so:

- Untersuchungen zur Anwendung der Malariafiebertherapie an der Universitätskinderklinik in den 1950er und 1960er Jahren in Erweiterung der Untersuchung zur Kinderstation der Psychiatrie im Projektendbericht von 2015.
- Untersuchungen – zumindest aufgrund der Fachpublikationen – zur Anwendung der somatischen Therapien an der Wiener Psychiatrie vor 1950, so zur Malariafiebertherapie bei nicht-luetischen Erkrankungen[1206] und zur Frage der Erhaltung des luesfreien Malariastamms.

[1204] Der Einbezug von PatientInnen als Zeitzeugen ins Projekt hätte eine Recherche nach Betroffenen (wie beim Projekt zum ‚Spiegelgrund') und Interviews durch PsychotherapeutInnen erfordert. Das vorliegende Projekt war ohne personenbezogene Recherche konzipiert.

[1205] Karl Kundratitz, von dem der einzige ausführliche Artikel über die Anwendung der Malariafiebertherapie bei ‚Intelligenzmängeln' stammt (1956), leitete von 1952 - 1961 die Wiener Universitätskinderklinik: vgl. ausführlich oben. S. 91f.

[1206] So berichten z. B. bereits mehrere Literaturbesprechungen in den ersten Jahrgängen der ab 1928 in Berlin erscheinenden Zeitschrift *Der Nervenarzt* über internationale Publikationen zur Anwendung der Malariafiebertherapie mit wechselnden Erfolgen auch bei nicht-luetischen Erkrankungen – bei Schizophrenie, bei manisch-depressiven Psychosen, bei chronischer Encephalitis epidemica. Vgl. auch HOFF, Uebersicht der

- Eine Textanalyse der ärztlichen Quellen (PatientInnenakten und wissenschaftliche Literatur) zum Bild, dass die KlinikärztInnen – ev. im Vergleich zu den ÄrztInnen in den psychiatrischen Krankenhäusern (PatientInnenakten) – von ihren PatientInnen hatten und wie dieses ihre Behandlung beeinflusste.[1207]
- Eine die statistischen Ergebnisse[1208] weiterführende Untersuchung der geschlechtsspezifischen und sozialen Aspekte in der Diagnoseerstellung und Behandlungspraxis der Zeit.
- Anschluss könnte auch die Malariaforschung finden, die bereits für zwei Diplomarbeiten die Datenbank und die gleichen PatientInnenakten ausgewertet hat,[1209] deren Ergebnisse 2025 erweitert in einem englischen Aufsatz im AJTMH erscheinen soll.[1210]

therapeutischen Versuche bei der Encephalitis lethargica auf der Klinik vom 1. Jänner 1916 bis 30. Mai 1923 (1923), passim.

[1207] Vgl. oben S. 76 Anm. 246 zu den textkritischen Publikationen von Joel T. Braslow 1995 und 1996.

[1208] Vgl. oben S. 41-50 und 264f.

[1209] KAINZ, Retrospektive Datenanalyse iatrogener P. vivax Infektionen (2019); REIMOLD, Retrospektive Datenanalyse iatrogener P. vivax Infektionen an der Wiener Psychiatrie (2020),

[1210] WOLF, KAINZ, REIMOLD, LAGLER, MISCHLINGER, RAMHARTER, Clinical and parasitological characteristics of *Plasmodium vivax* malaria in malaria-naïve patients: A review of malaria fever therapy in patients with schizophrenia and neurosyphilis during the 1950s and 1960s in Vienna, Austria, in: American Journal of Tropical Medicine and Hygiene (AJTMH), submitted 2025.

Quellenverzeichnis

a) Quellen in Archiven:

Nachlass Hoff im Josephinum, Wien

Krankenakten der beiden psychiatrischen Erwachsenenstationen und der Kinderstation der Wiener Psychiatrisch-Neurologischen Universitätsklinik 1951 bis 1969 im Archiv der Universitätsklinik für Psychiatrie und Psychotherapie des Allgemeinen Krankenhauses, Wien

Krankenakten der Psychiatrie Baumgartner Höhe (*Steinhof*) von 10 Patienten im Wiener Stadt- und Landesarchiv [WStLA] Bestand M. Abt, 209.2 – Otto-Wagner-Spital, Krankengeschichten

Personalakten [PA] der Medizinischen Fakultät im Universitätsarchiv Wien [UA] bzw. (und ergänzend durch den) Personalstand der Universität Wien von Ottokar Heinrich Arnold, Kornelius Kryspin-Exner, Ludwig Popper

Staatsarchiv des Kantons Zürich
StAZH, Z100, KA-Nr. 31146 (1938) und StAZH, Z100, KA-Nr. 338001 (1950) – Hinweis von Marietta Meier
Persönlichen Korrespondenz Manfred Bleuler, Staatsarchiv des Kantons Zürich, Signatur Z 99.1047 und Z99.1048 – Hinweis von Eberhard Gabriel

Im Projekt aus den genannten Krankenakten der beiden Erwachsenenstationen und der Kinderstation erstellte Datenbanken, Interviews und Antworten von Zeitzeugen auf Anfragen, die nach Abschluss des Projekts im Josephinum, Wien deponiert wurden.

Literatur:

B. ABEL, Katamnestische Untersuchungen zur modernen Therapie der Neurolues. In: Psychiatrie, Neurologie und medizinische Psychologie. Zeitschrift für Forschung und Praxis 13 (Leipzig 1961), S. 421-428.

B. ACKNER/A. HARRIS/A. J. OLDHAM, Insulin treatment of schizophrenia. A controlled study. In: The Lancet 272 (1957), S. 607-611.

Klaus AKTORIES/Ullrich FÖRSTERMANN/Franz Bernhard HOFMANN/Klaus STARKE, Allgemeine und spezielle Pharmakologie und Toxikologie (München. ^{10}2009).

L. AMBROZI/W[alter] BIRKMAYER/W[alter] DANIELCZYK, Die pharmako-dynamische Beeinflussung des thalamo-retikulären Systems als therapeutisches Prinzip in der Psychiatrie. In: Wiener Medizinische Wochenschrift 110/36 (1960), S. 726-728.

Kaj ARENTSEN/Joseph WELNER, Hypertherm Treatment of Neurosyphilis. In: Acta Psychiatrica et Neurologica Scandinavica 30/4 (1955), S. 529-552.

Ingrid ARIAS, Die medizinische Fakultät von 1945-1955. Provinzialisierung oder Anschluss an die westliche Wissenshaft. In: Margarete GRANDNER/Gernot HEISS/Oliver RATHKOLB (Hrsg.), Zukunft mit Altlasten. Die Universität Wien 1945 bis 1955 (= Querschnitte. Einführungstexte zur Sozial-, Wirtschafts- und Kulturgeschichte 19, Innsbruck/Wien/München/Bozen 2005), S. 68-88.

Ingrid ARIAS, Die Wiener Medizinische Fakultät von 1945 bis 1955. Entnazifizierung, Personalpolitik und Wissenschaftsentwicklung (ungedr. phil. Dissertation Wien 2013).

Ingrid ARIAS, Hans Hoff 1897-1969) – Remigrant und Reformer? Neue Impulse oder Kontinuität in der Psychiatrie nach 1945. In: Virus. Beiträge zur Sozialgeschichte der Medizin 14 (Leipzig 2016) S. 177-190.

O[ttokar] [Heinrich] ARNOLD, Untersuchungen zur Frage der akuten tödlichen Katatonien. In: Wiener Zeitschrift für Nervenheilkunde und deren Grenzgebiete 2 (1949), S. 386-401.

O[ttokar] [Heinrich] ARNOLD, Zur Indikation des Elektroschocks. In: Wiener klinische Wochenschrift 63 (1951), S. 108.

Ottokar [Heinrich] ARNOLD/W. BÖCK-GREISSAU/K. H. GINZEL, Über die Einführung eines neuen muskellähmenden Mittels in die Elektrotherapie. In: Wiener Medizinische Wochenschrift 101/26 (1951), S. 492.

O[ttokar] H[einrich] ARNOLD/H. STEPAN, Untersuchungen zur Frage der akuten tödlichen Katatonie. In: Wiener Zeitschrift für Nervenheilkunde und deren Grenzgebiete 5/2-3 (1952), S. 235-258.

O[ttokar] H[einrich] ARNOLD/R[aoul] SCHINDLER, Bifokale Gruppentherapie bei Schizophrenen. In: Wiener Zeitschrift für Nervenheilkunde und deren Grenzgebiete 5/2-3 (1952), S. 155-174.

O[ttokar] [Heinrich] ARNOLD, Zur Theorie der Insulinschocktherapie der Schizophrenie. In: Wiener Medizinische Wochenschrift 102/49 (1952), S. 976-978.

O[ttokar] H[einrich] ARNOLD/St[efan] HIFT/W[ilhelm] SOLMS, Über die Anwendung eines zentralvegetativen Hemmungsstoffes in der Psychiatrie. In: Wiener Medizinische Wochenschrift 102 (1953), S. 48.

O[ttokar] H[einrich] ARNOLD/St[efan] HIFT/W[ilhelm] SOLMS, Die Anwendung von Largactil in der Psychiatrie. In: Wiener Medizinische Wochenschrift 103/31 (1953), S. 563-566.

O[ttokar] H[einrich] ARNOLD, Behandlungsergebnisse bei der akuten tödlichen Katatonie. In: Wiener Medizinische Wochenschrift 103/5 (1953), S. 91-94.

O[ttokar] H[einrich] ARNOLD/H. ROTTER, Zur Elektroschockbehandlung des symptomatischen Alkoholismus (Tagesgeschichtliche Notizen: Gesellschaft der Ärzte in Wien –Wissenschaftliche Sitzung am 5. Juni 1953). In: Münchner medizinische Wochenschrift 14 (2.4.1954), S. 391.

O[ttokar] H[einrich] ARNOLD, Schockbehandlungen in der Psychiatrie, Teil I, in: Wiener Medizinische Wochenschrift 104/3 (1954), S. 53-56 und Teil II, ebenda 104/4 (1954), S. 69-72.

O[ttokar] H[einrich] ARNOLD/G[ustav] HOFMANN, Untersuchungen über Bernsteinsäureeffekte bei LSD-25-Vergiftungen und Schizophrenien. In: Wiener Zeitscchrift für Nervenheilkunde 26/11 (1955), S. 92-104.

O[ttokar] H[einrich] ARNOLD, Schizophrener Prozess und schizophrene Symptomgesetze. Eine prognostisch-statististische Grundlagenstudie (Wien/Bonn 1955).

O[ttokar] H[einrich] ARNOLD/H[eimo] GASTAGER, Insulinschockbehandlung trotz Diabetes. n: Wiener Zeitschrift für Nervenheilkunde und ihre Grenzgebiete 13/3 (1957), S. 260-266.

O[ttokar] H[einrich] ARNOLD, Klinische Erfahrungen mit dem Neuroleptikum Truxal. In: Wiener Medizinische Wochenschrift 109/46 (1959), S. 892-898.

O[ttokar] H[einrich] ARNOLD, Kombinierte Elektroschock-Tofranil-Behandlung der Melancholie. In: Wiener Medizinische Wochenschrift 110/111 (1960), S. 250-255.

O[ttokar] H[einrich] ARNOLD, Die körperlichen Behandlungsmethoden der Schizophrenie, in: Hans HOFF (Hrsg.), Therapeutische Fortschritte in der Neurologie und Psychiatrie (Wien/Innsbruck 1960), S. 262-278.

O[ttokar] H[einrich] ARNOLD/St[efan] HIFT/G[ustav] HOFMANN, Die Therapie des manisch-depressiven Krankheitsgeschehens. In: Hans Hoff (Hrsg.), Therapeutische Fortschritte in der Neurologie und Psychiatrie (Wien/Innsbruck 1960), S. 278-289.

O[ttokar] H[einrich] ARNOLD/H[ans] HOFF, Fortschritte in der Behandlung der endogenen Psychosen. In: Wiener Klinische Wochenschrit 73 (1961), S. 502-510.

O[ttokar] H[einrich] ARNOLD/G. FOIDL, Die Behandlung der endogenen Depression mit Amitriptyline. In: Wiener Medizinische Wochenschrift 111 (1961), S. 272-274.

O[ttokar] H[einrich] ARNOLD/H[ans] HOFF, Neuroleptika, Tranquilizer und Antidepressiva. Zusammenfassende kritische Stellungnahme (Wien 1962).

O[ttokar] H[einrich] ARNOLD, Die Therapie der Schizophrenie. Studien und Ergebnisse einer planmäßigen Ganzheitsbehandlung. Mit einem Vorwort von Professor Dr. med. Hans HOFF (Stuttgart 1963).

O[ttokar] H[einrich] ARNOLD/ K[ornelius] KRYSPIN-EXNER, Zur Frage der Beeinflussung des Verlaufs des manisch-depressiven Krankheitsgeschehens durch Antidepressiva. In: Wiener Medizinische Wochenschrift 115 (1965), S. 929-934.

O[ttokar] H[einrich] ARNOLD/H[elmut] TSCHABITSCHER, Die Wiener Neurologisch-Psychiatrische Schule unter H. Hoff. In: Wiener Medizinische Wochenschrift 117 (1967) S. 1128-1131.

O[ttokar] H[einrich] ARNOLD, Zum Stellenwert der Neuroleptikatherapie in den Behandlungsplänen der Schizophrenie. In: G[ustav] HOFMANN (Hrsg.), III. Donau-Symposium für Psychiatrie, Wien, 15.-18. Mai 1968. Tagungsbericht (Wien 1969), S. 11-14.

Hans ASPERGER, Heilpädagogik. Einführung in die Psychopathologie des Kindes für Ärzte, Lehrer, Psychologen, Richter und Fürsorgerinnen (Wien [1]1952, [2]1956, [3]1961, [4]1965, [5]1968).

Hans ASPERGER, Die medizinische, heilpädagogische und soziale Problematik zerebraler Störungen im Kindesalter, in: Wiener Medizinische Wochenschrift 49 (1958), S. 1081-1086.

M. ATSCHKOVA, Die Pfropfschizophrenie im Kindes- und Jugendalter, in: Psychiatrie, Neurologie und medizinische Psychologie 18 (1966), S. 292-295.

Edoardo BALDUZZI, Le terapie di shock (Milano 1962).

Viola BALZ, Zwischen Wirkung und Erfahrung – eine Geschichte der Psychopharmaka. Neuroleptika in der Bundesrepublik Deutschland, 1950 – 1980 (Bielefeld 2010).

Alexandre BARATTA/Alexandre MORALI, Les traitements biologiques en psychiatrie entre la seconde moitié du XIXe siècle et la première moitié du XXe siècle, in: L'information psychiatrique 86 (2010), S. 539-547 (20.1.2020: https://www.cairn.info/revue-l-information-psychiatrique-2010-6-page-539.htm)

Piroska BAUMANN/György KARDOS, Über die Schrankenprobleme bei der Penicillintherapie der Neurolues. In: Psychiatrie, Neurologie und medizinische Psychologie. Zeitschrift für Forschung und Praxis 9 (Leipzig 1957), S. 22-28.

T. BECKER/D. BENNET, Rudolf Karl Freudenberg – from pioneer of insulin treatment to pioneering social psychiatry. In: History of Psychiatry 11 (2000), S. 189-211.

Otto BENKERT/Hanns HIPPIUS, Psychiatrische Pharmakotherapie. Ein Grundriß für Ärzte und Studenten (Berlin/Heidelberg/New York 1974).

Otto BENKERT/Hanns HIPPIUS, Psychiatrische Pharmakotherapie, unter Mitarb. von Hermann Wentzel (Berlin/Heidelberg/New York/Tokio, 4. vollst. überarb. Aufl. 1986).

A.K. BERNATH, Modification of anxiety subsequent to insulin–induced mild hypoglycemia. In: Max RINKEL/Harold E. HIMWICH (Hrsg.), Insulin treatment in psychiatry. Proceedings of the international conference on the insulin treatment in psychiatry held at the New York Academy of Medicine October 24 to 25, 1958 (New York 1959).

Peter BERNER/Wilhelm SOLMS, Die Entwicklung einer Neurose. In: Wiener Zeitschrift für Nervenheilkunde und deren Grenzgebiete 8 (1954), S. 242-252.

Peter BERNER, Hans Hoff (1897-1969). In: Hans SCHLIACK/Hanns HIPPIUS (Hrsg.), Nervenärzte. Biographien (Stuttgart/New York 1998) S. 55-64.

Birgitta BERNET, „Der bürgerliche Tod": Entmündigungsangst, Psychiatriekritik und die Krise des liberalen Subjektentwurfs um 1900, in: Marietta MEIER/Brigitta BERNET/Roswitha DUBACH/Urs GERMANN (Hrsg.), unter Mitarbeit von Gisela HÜRLIMANN, mit einem

Schlusswort von Jakob TANNER, Zwang zur Ordnung. Psychiatrie im Kanton Zürich, 1870-1970 (Zürich 2007).

German E. BERRIOS/Roy PORTER (Hrsg), A History of clinical psychiatry. The origin and history of psychiatric disorders (London 1995).

R. BESSIERE/J. ALIZON (Paris), Premiers résultats de l'introduction de la pénicilline dans le traitement de la paralysie générale. In : Journal du 1er Congrès Mondial de Psychiatrie [Paris 1950], No. 4, 22 septembre 1950, S. 1.

Christof BEYER, Kinder- und Jugenpsychiatrische Abteilung der Pfälzischen Nervenklinik Landeck, in: Heiner FANGERAU/Anke DREIER-HORNING/Volker HESS/Karsten LAUDIEN/Maike ROTZOLL (Hrsg.), Leid und Unrecht. Kinder und Jugendliche in Behindertenhilfe und Psychiatrie der BRD und DDR 1949 bis 1990 (Köln 2021), S. 649-668.

Christoph BEYER/Cornelius BORCK/Jonatan HOLST/Gabriele LINGELBAC (mit einem Beitrag von Sebastian Graf von KIELMANSEGG), Wissenschaftliche Untersuchung der Praxis der Medikamentenversuche in schleswig-holsteinischen Einrichtungen der Behindertenhilfe sowie in den Erwachsenen-, Kinder- und Jugendpsychiatrien in den Jahren 1949 bis 1975 (Endbericht 2021): https://www.bmas.de/SharedDocs/Downloads/DE/Soziales/SAH/Materialien-und-Unterlagen/abschlussbericht_medikamentenversuche_1949-1975_schleswig-holstein.pdf?__blob=publicationFile&v=1 (23.09.2023)

Walther BIRKMAYER/Wilhelm WINKLER, Klinik und Therapie der vegetativen Funktionsstörungen (Wien 1951).

Eugen BLEULER, Lehrbuch der Psychiatrie (Berlin [6]1937).

Eugen BLEULER, Lehrbuch der Psychiatrie. Umgearbeitet von Manfred Bleuler (Berlin [7]1943).

Manfred BLEULER, Forschungen zur Schizophreniefrage. In: Wiener Zeitschrift für Nervenheilkunde 1/2-3 (1948), S. 129-148.

Eugen BLEULER, Lehrbuch der Psychiatrie. Umgearbeitet von Manfred BLEULER (Berlin/Göttingen/Heidelberg [9]1955).

Eugen BLEULER, Lehrbuch der Psychiatrie. Umgearbeitet von Manfred BLEULER (Berlin/Göttingen/Heidelberg [10]1960).

Eugen BLEULER, Lehrbuch der Psychiatrie. Umgearbeitet von Manfred BLEULER (Berlin/Heidelberg/New York [11]1969).

R. H. BOARDMAN/J. LOMAS/M[orris] MARKOWE, Insulin and Chlorpromazine in Schizophrenia. A comparative study in previously untreated cases. In: The Lancet 268 (1956), S. 487-491.

Jørgen BOAS, On the Treatment of Neurosyphilis with Artificial Fever and Penicillin. In: Acta Psychiatrica et Neurologica Scandinavica 25 (60) (1950), S. 24-34.

Cornelius BORCK, Hirnströme. Eine Kulturgeschichte der Elektroenzephalographie (Göttingen 2005).

Cornelius BORCK, Die Internationale der invasiven Therapien und die Diskussion in Deutschland. In: Hans-Walter SCHMUHL/Volker ROELCKE (Hrsg.), „Heroische Therapien.“ Die deutsche Psychiatrie im internationalen Vergleich, 1918-1945 (Göttingen 2013), S. 131-148.

Harold BOURNE, The Insulin Myth. In: The Lancet 265 (7. November 1953), S. 964-968.

Harold BOURNE, Insulin coma in decline. In: American Journal of Psychiatry 114 (1958), S. 1015-1017.

Joel T. BRASLOW, Effect of Therapeutic Innovation on Preception of Disease and the Doctor-Patient Relationship: A History of General-Paralysis of the Insane and Malaria Fever Therapy, 1910-1950. In: American Journal of Psychiatry 152/5 (1995), S. 660-665.

Joel T. BRASLOW, The Influence of a Biological Therapy on Physicians' Narratives and Interrogations: The Case of General Paralysis of the Insane and Malaria Fever Therapy, 1910-1950. In: Bulletin of History of Medicine 70 (1996), S. 577-608 [ausführlichere Fassung des Artikels von 1995].

J[oel] T. BRASLOW, History and Evidence-Based Medicine: Lessons from the History of Somatic Treatments from the 1900s to the 1950s. In: Mental Health Services Research 1/4 (1999), S. 231-240.

Burkhart Brückner/Thomas Röske/Maike Rotzoll/Thomas Müller, Geschichte der Psychiatrie „von unten“. Entwicklung und Stand der deutschsprachigen Forschung, in: Medizinhistorisches Journal 54 (2019), S. 347-376.

E. A. J. BYRNE, Malarial Therapy in Lipoid Nephrosis. In: The Lancet 259/6713 (1952), S. 844-845.

Carlo Lorenzo CAZZULLO, Wirkung der Monoaminooxydasehemmer (MOI) in der Depression. Psychodynamik und biologische Reaktivität. In: Wiener Medizinische Wochenschrift 110/36 (1960), S. 731-733.

I. CERMAK/E[rwin] RINGEL, Zur Frage der Angst vor dem Elektroschock. In: Wiener klinische Wochenschrift 71 (1959), S. 407-411.

Henri CLAUDE/Jean DUBLINEAU, Résultats de la pyrétothérapie dans 34 états dit de démence précoce. In: Annales médico-psychologiques 93/1 (1935), S. 553-583.

Henri CLAUDE/Pierre RUBINOVITCH, Thérapeutiques biologiques des affections mentales (Paris 1940).

Jean-Christophe COFFIN, French Biological Therapeutics in the European Context. In: Hans-Walter SCHMUHL/Volker ROELCKE (Hrsg.), „Heroische Therapien.“ Die deutsche Psychiatrie im internationalen Vergleich, 1918-1945 (Göttingen 2013) S. 185-199.

A. D. COLLINS, Insulin Therapy (Letters to the editor). In: The Lancet (December 30, 1961 / Bd. 2), S. 1457.

H. CZERWENKA-WENKSTETTEN/G. HOFMANN, Klinische Erfolge mit Melleril-retard. In: Wiener klinische Wochenschrift 78 (1966), S. 845-847.

Rudolf DEGKWITZ, Reicht bei der Neurolues in jedem Falle alleinige Penicillinbehandlung aus? In: Der Nervenarzt 26 (1955), S. 120f.

Jean DELAY, Méthodes biologiques en clinique psychiatrique (Paris 1950).

Jean DELAY/P[ierre] DENIKER/R. PAUWELS, Cure de sommeil et cures neuroleptiques en psychiatrie. In: Colloque international sur la chlorpromazine et les médicaments neuroleptiques en thérapeutique psychiatrique. Paris 20, 21, 22 octobre 1955, hrsg. Von Jean DELAY (Paris 1956) 136-139.

Jean DELAY (Hrsg.), Colloque international sur la chlorpromazine et les médicaments neuroleptiques en thérapeutique psychiatrique. Paris 20, 21, 22 octobre 1955 (Paris 1956).

Andrea DÖRRIES, Der „Würzburger Schlüssel“ von 1933 – Diskussionen um die Entwicklung einer Klassifikation psychischer Störungen. In: Thomas BEDDIES/Andrea DÖRRIES (Hrsg.), Die Patienten der Wittenauer Heilstätten in Berlin (= Abhandlungen zur Geschichte der Medizin und der Naturwissenschaften 91, Husum 1999), S. 188-205.

Deborah Blythe DOROSHOW, Performing a Cure for Schizophrenia. Insulin Coma Therapy on the Wards. In: Journal of the History of Medicine and Allied Sciences 62/2 (2006), S. 213-243

Wolfgang U. ECKART (Hrsg.), Man, Medicine and the State. The Human Body as an Object of Government Sponsored Medical Research in the 20th Century (Stuttgart 2006).

Wolfgang U. ECKART, Introduction. In: Wolfgang U. ECKART (Hrsg.), Man, Medicine and the State. The Human Body as an Object of Government Sponsored Medical Research in the 20th Century (Stuttgart 2006), S. 9-13.

Wolfgang U. ECKART, Geschichte, Theorie und Ethik der Medizin (Berlin ^{7}2013).

Wolfgang U. ECKART/Andreas REULAND, First principles: Julius Moses and medical experimentation in the late Weimar Republic. In: Wolfgang U. ECKART (Hrsg.), Man, Medicine and the State. The Human Body as an Object of Government Sponsored Medical Research in the 20th Century (Stuttgart 2006), S. 35-47.

O[tto] EICHHORN, Die gegenwärtige Situation bei der klinischen Behandlung von Depressionen. In: Wiener Klinische Wochenschrift 72/13 (1960), S. 224-225.

Gerhard W. ESCHWEILER/Barbara WILD/Mathias BARTELS, Elektromagnetische Therapien in der Psychiatrie. Elektrokrampftherapie (EKT), Transkranielle Magnetstimulation (TMS) und verwandte Verfahren (Darmstadt 2003).

Henri EY/Paul BERNARD/Charles BRISSET, Manuel de Psychiatrie (Paris 1960).

Henri EY/Paul BERNARD/Charles BRISSET, Manuel de Psychiatrie (Paris ^{2}1963).

Henri Ey, La thérapeutique psychiatrique (Généralités). In : Encyclopédie Médico-Chirurgicale, Psychiatrie 5-37800 A^{10} (Paris 1976), S. 1-20.

Heiner FANGERAU/Anke DREIER-HORNING/Volker HESS/Karsten LAUDIEN/Maike ROTZOLL (Hrsg.), Leid und Unrecht. Kinder und Jugendliche in Behindertenhilfe und Psychiatrie der BRD und DDR 1949 bis 1990 (Köln 202).

Rudolf FORSTER, Staat, Politik und Psychiatrie in Österreich – am Beispiel der rechtlichen Regulierung von Zwangsmaßnahmen von 1916 bis 1990. In: Brigitta KEINTZEL/Eberhard GABRIEL (Hrsg.), Gründe der Seele. Die Wiener Psychiatrie im 20. Jahrhundert (Wien 1999), S. 166-189.

Robert FREUDENTHAL/Joanna MONCRIEFF, ‚A landmark on psychiatric progress‘? The role of evidence in the rise and fall of insulin coma therapy. In: History of Psychiatry 33 (2022), S. 65-78.

E[berhard] GABRIEL [sen.], Erfahrungen mit Tofranil in der psychiatrischen Sprechstunde. In: Wiener Medizinische Wochenschrift 112 (1962), S. 222-227.

E[berhard] GABRIEL/B[ernd] KÜFFERLE, Vergleich des Verlaufes der Insulinvollkoma-Kur bei Zucker- bzw. Glucagonweckung. In: Wiener Zeitschrift für Nervenheilkund 26 (1969), S. 338-343.

Eberhard GABRIEL, Zur Beziehung zwischen Psychiatrie und Psychotherapie in Wien im 20. Jahrhundert – Eine psychiatriegeschichtliche Einführung zu ihrer Entwicklung um die Jahrhundertwende. In: Brigitta KEINTZEL/Eberhard GABRIEL (Hrsg.), Gründe der Seele. Die Wiener Psychiatrie im 20. Jahrhundert (Wien 1999), S. 15-28.

Eberhard GABRIEL/Wolfgang NEUGEBAUER (Hrsg.), Vorreiter der Vernichtung? Von der Zwangssterilisierung zur Ermordung. Zur Geschichte der NS-Euthanasie in Wien Teil II (Wien 2002).

Eberhard GABRIEL, 100 Jahre Gesundheitsstandort Baumgartner Höhe. Von der Heil- und Pflegeanstalt Am Steinhof zum Otto Wagner-Spital. Mit einem Beitrag von Sophie Ledebur (Wien 2007).

Eberhard GABRIEL, Die Orientierung(en) der österreichischen Psychiatrie 1945 bis Mitte der 50er Jahre. In: Schriftenreihe der Deutschen Gesellschaft für Geschichte der Nervenheilkunde 21 (2015) S. 586-611.

Eberhard GABRIEL, Zum Wiederaufbau akademischer Lehrkörper in der Psychiatrie in Wien nach 1945. In: Virus. Beiträge zur Sozialgeschichte der Medizin 14 (Leipzig 2016), S. 35-77.

Eberhard GABRIEL, „Wie dann der Hoff gekommen ist, hat man den Kauders geschwind vergessen." Otto Kauders (1893-1949), Professor für Psychiatrie und Neurologie und Klinikvorstand in Wien 1945 bis 1949. In: Schriftenreihe der Deutschen Gesellschaft für Geschichte der Nervenheilkunde 24 (2018) 209-228.

Eberhard GABRIEL, „… trotzdem ich … vielleicht die Rolle eines Filmstars in anderen Ländern habe …" Hans Hoff (1897-1969), Professor für Psychiatrie und Neurologie in Wien 1950-1969. In: Schriftenreihe der Deutschen Gesellschaft für Geschichte der Nervenheilkunde 25 (2019) S. 337-364.

G[erhard] GANN, Zur Differentialdiagnose und Therapie der Depressionen. Der parenterale Niamidstoß. In: Wiener Medizinisch Wochenschrift 114 (1964), S. 588-592 und S. 609-612.

Douglas GAIRDNER, Nephrosis treated by Malaria. In: The Lancet 259/6713 (1952), S. 842-844.

H[aimo] GASTAGER/R[aoul] SCHINDLER, Rehabilitationstherapie bei Schizophrenen. In: Der Nervenarzt 32 (1961) 368-374.

H[aimo] GASTAGER/I. HAAS/E.WEINKAMER, Erfahrungsbericht über die Anwendung von Distraneurin in der Psychiatrie. In: Wiener Klinische Wochenschrift (1964), S. 639-644.

Max GAWLICH, Eine Maschine, die wirkt: die Elektrokrampftherapie und ihr Apparat, 1938-1950 (Paderborn 2018).

Max Gawlich, Buttons and Stimuli: The Material Basis of Electroconvulsive Therapy as a Place of Historical Change. In: Monika Ankele/Benoît Majerus, Material Cultures of Psychiatry (Bielefeld 2020), S. 202-222.

Vgl. Katja GEIGER, Die Kinderstation. In: Die Malariatherapie und weitere diagnosekorrelierte Therapien. Ihre Anwendung an der Wiener Universitätsklinik für Psychiatrie und Neurologie in den 1950er und 1960er Jahren und ihre Diskussion in der zeitgenössischen Forschung. Projektendbericht des Projektleiters Gernot HEISS für den Jubiläumsfonds der Österreichischen Nationalbank (Typoskript Wien 2015), S. 249-264.

Urs GERMANN, Arbeit als Medizin: Die „aktive Krankenbehandlung" 1930-1960 (2007), in: Marietta MEIER/Brigitta BERNET/Roswitha DUBACH/Urs GERMANN (Hrsg.), unter Mitarbeit von Gisela HÜRLIMANN, mit einem Schlusswort von Jakob TANNER, Zwang zur Ordnung. Psychiatrie im Kanton Zürich, 1870-1970 (Zürich 2007), S. 195-233.

Urs GERMANN, Ein Insulinzentrum auf dem Land. Die Einführung der Insulinbehandlung und der therapeutische Aufbruch in der Schweizer Psychiatrie der Zwischenkriegszeit. In: Hans-Walter SCHMUHL/Volker ROELCKE (Hrsg.), „Heroische Therapien." Die deutsche Psychiatrie im internationalen Vergleich, 1918-1945 (Göttingen 2013), S. 149-167.

D. GHERARDUCCI, A propos de l'utilisation de la chlorpromazine associée á d'autres thérapeutiques psychiatriques. In: L'encéphale. Journal de neurologie, de psychiatrie et de médecine psycho-somatique 45 (Paris 1956), S. 887-889.

K. GIERCKE/U. KLEINPETER, Vergleichende Untersuchungen über Behandlungserfolge bei psychiatrischen Kranken zur Zeit der Konvulsivtherapie und der Psychopharmaka. In: Psychiatrie, Neurologie und medizinische Psychologie. Zeitschrift für Forschung und Praxis 15 (Leipzig 1963), S. 331-337.

Eberhard GLAUBITZ, Penicillin allein oder in Kombination mit Fieber bei der Behandlung luischer Erkrankungen des Zentralnervensystems. In: Der Nervenarzt 24 (1953), S. 505-507.

Helmuth GRAGE, Penicillin allein oder in Kombination mit Fieber bei der Behandlung luischer Erkrankungen. Bemerkungen zu der gleichnamigen Arbeit von Eberhard Glaubit. In: Der Nervenarzt 25 (1954), S. 301.

Margarete GRANDNER/Gernot HEISS/Oliver RATHKOLB (Hrsg.), Zukunft mit Altlasten. Die Universität Wien 1945 bis 1955 (= Querschnitte. Einführungstexte zur Sozial-, Wirtschafts- und Kulturgeschichte 19, Innsbruck/Wien/München/Bozen 2005).

Helmut GRÖGER/Siegfried KASPER, Zur Dominanz der organische-biologischen Auffassung in der Psychiatrie der Wiener medizinischen Schule und den Anfängen der Psychopharmakotherapie, in: Helmut GRÖGER/Eberhard GABRIEL/Siegfried KASPER (Hrsg.), Zur Geschichte der Psychiatrie in Wien (Wien 1997), S. 14 – 18.

Helmut GRÖGER, Zur Entwicklung der Psychiatrie in der Wiener Medizinischen Schule. In: Brigitta KEINTZEL/Eberhard GABRIEL (Hrsg.), Gründe der Seele. Die Wiener Psychiatrie im 20. Jahrhundert (Wien 1999), S. 30-48

Helmut GRÖGER, Die Insulin-Schocktherapie – ihre Problematik und grundsätzliche Bedeutung. In: Schriftenreihe der deutschen Gesellschaft für die Geschichte der Nervenheilkunde 11 (2005), S. 209-224.

Helmut GRÖGER, Die Entwicklung der psychischen Hygiene von der Bewegung zur psychiatrischen Disziplin – der österreichische Weg. In: Schriftenreihe der Deutschen Gesellschaft für Geschichte der Nervenheilkunde 18 (2012) S. 221-232.

Helmut GRÖGER, Röntgen- und Malariatherapie. Zur Therapie des kindlichen Schwachsinns. In: Schriftenreihe der Deutschen Gesellschaft für Geschichte der Nervenheilkunde 25 (2019), S. 155-180.

Heinrich GROSS, Erfahrungen mit neuroleptischen Behandlungsverfahren in der Anstaltspsychiatrie. In: Wiener Medizinische Wochenschrift 110/36 (1960), S. 718-719.

E. Guth/G[ustav] Hoffmann, Erfahrungen bei der Anwendung von SD 709 beim depressiven Syndrom, in: Wiener Klinische Wochenschrift 78 (1966), S. 14-16.

Hans-J HAASE, Über Vorkommen und Deutung des psychomotorischen Parkinsonsyndroms bei Megaphen- bzw. Largactil-Dauerbehandlung. In: Der Nervenarzt 25 (1954), S. 486-492.

Hans-J. HAASE, Therapie mit Psychopharmaka und anderen psychotropen Medikamenten (Oldenburg ^{2}1969).

S. HADDENBROCK/W. KEMMANN, Über die Wirkung der Elektrokrampfbehandlung auf das pathologische Elektrokardiogramm. In: Der Nervenarzt 10 (1950), S. 442-446.

H. HÄFNER/E. SCHLIACK/H. ZENZ, Die hochdosierte Reserpin-Kur in der Behandlung chronischer Schizophrener. In: Fortschritte der Neurologie, Psychiatrie und ihrer Grenzgebiete 36 (1968), S. 197-227.

M[anfred] HAIDER/W[alter] SPIEL, Einige psychologische und psychiatrische Betrachtungen zum Jugendstrafrecht und Jugendstrafvollzug. In: Wiener Archiv für Psychologie, Psychiatrie und Neurologie 6 (1956), S. 142-153.

M[anfred] HAIDER/W[illibald] SLUGA, Probleme der psychotherapeutischen Betreuung in der Erziehungsanstalt. In: Mitteilungen. Verein der Ärzte in der Justizverwaltung 1 (1956/57), Heft 2, S. 17-24.

Frank HALL, Psychopharmaka - Ihre Entwicklung und klinische Erprobung. Zur Geschichte der deutschen Pharmakopsychiatrie von 1844 Bis 1952 (Hamburg 1997).

Brigitta HASELBACHER, Die „Revolte“ in der Bundesanstalt für Erziehungsbedürftige Kaiser-Ebersdorf im Jahre 1952 (ungedr. Dipl.-Arbeit Wien 1991).

W. H. HEATON-WARD, Psychopatic Disorder. In: The Lancet, 19.1.1963, S. 121-123.

Die Malariatherapie und weitere diagnosekorrelierte Therapien. Ihre Anwendung an der Wiener Universitätsklinik für Psychiatrie und Neurologie in den 1950er und 1960er Jahren und ihre Diskussion in der zeitgenössischen Forschung. Projektendbericht des Projektleiters Gernot HEISS für den Jubiläumsfonds der Österreichischen Nationalbank (Typoskript Wien 2015).

Barbara HELIGE/Michael JOHN/Helge SCHMUCKER/Gabriele WÖRGÖTTER/Marion WISINGER, Endbericht der Kommission Wilhelminenberg (hrsg. Institut für Rechts- und Kriminalsoziologie der Universität Wien & Kommission Wilhelminenberg, Wien 2013).

Daniel HELMCHEN, Malariaimpftherapie der Progressiven Paralyse in den Wittenauer Heilstätten. In: Thomas Beddies/Andrea Dörries (Hrsg.), Die Patienten der Wittenauer Heilstätten in Berlin (= Abhandlungen zur Geschichte der Medizin und der Naturwissenschaften 91, Husum 1999), S. 435-461.

HERRMANN (Prag), Rezension von M. L. Bianchini (Teramo), Malariatherapia delle psicosi maniacodepressiva (Arch. gen. di Neur. 10/5, 1929). In: Der Nervenarzt 3/1 (1930), S. 58.

Rainer HERRN, Wie die Traumdeutung durch die Türritze einer geschlossenen Anstaltsickert. Zum Umgang mit der Psychoanalyse an der Psychiatrischen und Nervenklinik der Charité. In: Hans-Walter SCHMUHL/Volker ROELCKE (Hrsg.), „Heroische Therapien.“ Die deutsche Psychiatrie im internationalen Vergleich, 1918-1945 (Göttingen 2013), S. 69-99.

Georges HEUYER, Introduction à la psychiatrie infantile (Paris 1952).

G[eorges] HEUYER, Evolution de la Pédiatrie et de la Neuropsychiatrie infantile. In: Wiener Zeitschrift für Nervenheilkunde und deren Grenzgebiete 19 (1962), S. 135-145.

St[efan] HIFT, Zur weiteren Entwicklung der Insulintherapie. In: Wiener Medizinische Wochenschrift 102/49 (1952), S. 975-976.

St[efan] HIFT/G[ustav] HOFMANN, Der vollmitigierte Elektroschock. Theoretische Erwägungen an Hand einer neuen Schockmethode, in: Wiener Medizinische Wochenschrift 104/23 (1954), S. 455-458.

St[efan] HIFT, Die klinische Lenkung der psychiatrischen Therapie. In: Wiener Zeitschrift für Nervenheilkunde und deren Grenzgebiete 15 (1958), S. 127-134.

St[efan] HIFT/H[ans] HOFF, Die organische Therapie der Psychosen. In: Wiener Medizinische Wochenschrift 108/47 (1958), S. 1043-1048.

St[efan] HIFT/Kornelius KRYSPIN-EXNER, Prothipendyl-hydrochlorid, ein neues Neuroleptikum, in: Wiener Medizinische Wochenschrift 108/32 (1958), S. 664-668.

St[efan] HIFT, Die Behandlung der endogenen Depression an der Wiener Klinik. In: Wiener Klinische Wochenschrift 72/16 (1960), S. 289-290.

St[efan] HIFT, Die Ganglienblockade bei der Insulinkur in der Psychiatrie. In: Wiener klinische Wochenschrift 73 (1961), S. 430-433.

Hanns HIPPIUS, Klinische und theoretische Aspekte der Pharmakotherapie des depressiven Syndroms. In: Wiener Medizinische Wochenschrift 110/11 (1960), S. 260-263.

A[lbrecht] HIRSCHMÜLLER, Die Insulinkomabehandlung der Schizophrenie oder: Wie erweist sich die Unwirksamkeit einer Therapiemethode? In: Nervenheilkunde 4 (2001), S. 217-226.

Ferdinand HOFF, Fieber – unspezifische Abwehrvorgänge – unspezifische Therapie (Stuttgart 1957).

Hans HOFF, Uebersicht der therapeutischen Versuche bei der Encephalitis lethargica auf der Klinik vom 1. Jänner 1916 bis 30. Mai 1923. In: Wiener Klinische Wochenschrift 1923, S. 899-901.

Hans HOFF/Otto KAUDERS, Über die Malariabehandlung der Tabes dorsalis. In: Zeitschrift für die gesamte Neurologie und Psychiatrie 104 (1926), S. 306-322.

Hans HOFF, Hypoglykämie-Schockbehandlung von Psychosen. In: Wiener klinische Wochenschrift 49 (1936), S. 917-918.

Hans HOFF, Die organischen Grundlagen der Psychosen [Antrittsvorlesung, 12. 10. 1950]. In: Wiener klinische Wochenschrift 63 (1951), S. 2-5.

Hans HOFF, Psychochirurgie. In: Wiener Zeitschrift für Nervenheilkunde und deren Grenzgebiete 3 (1951), S. 425-439.

Hans HOFF, Die Therapie der jugendlichen Psychopathie. In: Wiener Archiv für Psychologie, Psychiatrie und Neurologie 1 (1951), S. 201-211.

Hans HOFF, Tätigkeitsbericht über die Psychische Hygiene in Österreich für das Jahr 1950/51. In: Wiener Archiv für Psychologie, Psychiatrie und Neurologie 2 (1952) 47-55.

Hans HOFF/Erwin RINGEL, Die sogenannte Soldatenbraut. Über eine besondere Form weiblicher Gefährdung in unserer Zeit. In: Wiener Archiv für Psychologie, Psychiatrie und Neurologie 2 (1952), S. 140-154.

Hans Hoff, Professor Dr. Otto Pötzl – 75 Jahre, in: Wiener Medizinische Wochenschrift 102 (1952), S. 971f.

Hans HOFF/Walter SPIEL, Die Dynamik der kriminellen Psychopathie Jugendlicher. In: Der Jugendliche im Lichte der Kriminalbiologie. Forschungsergebnisse vorgetragen auf der 6. Arbeitstagung der Kriminalbiologischen Gesellschaft in München (Oktober 1951) (= Schriftenreihe der Vereinigung für Jugendgerichte und Jugendgerichtshilfen, NF 3 / Mitteilungen der Kriminalbiologischen Gesellschaft 6, München/Düsseldorf [1952]), S. 21-34.

H[ans] HOFF/O[ttokar] H. ARNOLD, Die Therapie der Schizophrenie. In: Wiener Klinische Wochenschrift 66 (1954), S. 345-352.

Hans HOFF/O[ttokar] H. ARNOLD, Au sujet de la thérapie de la schizophénie. In : L'encéphale. Journal de Neurologie et de Psychiatrie 44 (1955), S. 1-25 [französische Version von HOFF/ARNOLD 1954: Die Therapie der Schizophrenie (1954)].

Hans HOFF, Advantages and Disadvantages of Treatment with Chlorpromazine and Serpasil. In: Colloque international sur la chlorpromazine et les médicaments neuroleptiques en thérapeutique psychiatrique, Paris octobre 1955, hrsg. Jean Delay (Paris 1956), S. 52-58. (Auch publiziert in: L'encéphale. Journal de neurologie, de psychiatrie et de médecine psycho-somatique 45, Paris 1956, S. 352-358.)

Hans HOFF, Lehrbuch der Psychiatrie. Verhütung, Prognostik und Behandlung der geistigen und seelischen Erkrankungen, 2 Bde [durchpaginiert, aus Vorlesungen hervorgegangen und nach Vorlesungen gegliedert] (Wien/Basel 1956).

Hans HOFF/Erwin RINGEL, Anfänge der Psychopathie. In: Medizinische Klinik 51 (1956), S. 423-425.

Hans HOFF, Zum 100. Geburtstag Wagner-Jaureggs. In: Wiener Zeitung für Nervenheilkunde und deren Grenzgebiete 14 (1958), S. 1-15.

Hans HOFF/Hans STROTZKA (Hrsg.), Die psychohygienische Betreuung ungarischer Neuflüchtlinge in Österreich 1956-1958. In Verbindung mit einer Anleitung zum Verständnis und zur Betreuung von Menschengruppen in Extremsituationen (Wien 1958).

Hans HOFF (Hrsg.), Therapeutische Fortschritte in der Neurologie und Psychiatrie (Wien/Innsbruck 1960).

[Hans HOFF], Allgemeine Psychiatrie (unautorisiertes Skriptum zur Vorlesung der Hochschülerschaft der Universität Wien [um 1961]) – zitiert als Hoff-Skriptum, Allgemeine Psychiatrie [um 1961].

H[ans] HOFF/W[illibald] SLUGA, Das psychopathische Syndrom. In: Wiener Zeitschrift für Nervenheilkunde und deren Grenzgebiete 19 (1962), S. 241-270.

Hans HOFF, War die Errichtung einer kinderpsychiatrisch-neurologischen Abteilung nötig? In: Wiener Zeitschrift für Nervenheilkunde und deren Grenzgebiete 19 (1962), S. 101-104.

H[ans] HOFF/G[ustav] HOFMANN, Die Anwendung der Neuroleptika in der psychiatrischen und allgemeinen Praxis. In: Wiener Medizinische Wochenschrift 113 (1963), S. 269-275.

Bernt HOFFMANN, Zur Frage der ambulanten Schockbehandlung. In: Der Nervenarzt 9 (1950), S. 409-412.

Gustav HOFMANN, Über Kriterien einer differenzierten Anwendungsweise von Neuroleptica und Antidepressiva in der klinischen Psychiatrie. In: Wiener Zeitschrift für Nervenheilkunde und deren Grenzgebiete 21 (1964), S. 144-154.

Karl HUBER, Ueber Psychochirurgie. In: Wiener klinische Wochenschrift 62 (1950), S. 956-958.

Marion A. HULVERSCHEIDT, Germann Malariology experiments with humans, supported by the DFG. In: W. U. ECKART (Hrsg.), Man, Medicine and the State (Stuttgart 2006), S. 221-236.

Marion HULVERSCHEIDT, Die Beteiligung von Mitarbeitern des Robert Koch-Instituts an Verbrechen gegen die Menschlichkeit – tropenmedizinische Menschenversuche im Nationalsozialismus. In: Marion HULVERSCHEIDT/Anja LAUKÖTTER (Hrsg.), Infektion und Institution: zur Wissenschaftsgeschichte des Robert Koch-Instituts im Nationalsozialismus (Göttingen 2009), S. 147-168.

Marion A. HULVERSCHEIDT, Forschungslenkung international – Malariaforschung im Rahmen des Malaria Eradication Programme der WHO 1955-1972. In: Axel C. HÜNTELMANN/Michael C. SCHNEIDER (Hrsg.), Jenseits von Humboldt – Wissenschaft im Staat 1850-1990 (Frankfurt a. M. 2010), S. 133-146.

Maxwell JONES/Fergus STALLARD/Isobel H. HUNTER/Ronald A. BROOKS, The Psychopath and the Mental Health Bill, in: The Lancet (March 14, 1959), S. 566-568.

F[ranz] JOST, Zur Modifikation der Insulinschockkuren nach Sakel mit Insulin Novo Amorph. In: Wiener klinische Wochenschrift 68 (1956), S. 1013-1017.

F[ranz] JOST, Zur Verwendung der Allgemeinnarkose in der Psychiatrie. Mit besonderer Berücksichtigung der Elektroschockbehandlung unter Mitigierung durch Lysthenon. In: Wiener Medizinische Wochenschrift 107/17 (1957), S. 337-339.

F[ranz] JOST, Zur Insulinempfindlichkeit der Schizophrenen. In: Wiener klinische Wochenschrift 70 (1958), S. 657-661.

F[ranz] JOST/K. KATZELBERGER 1958: Zu den Reserpin-Kuren in der klinischen Praxis der Psychiatrie. In: Wiener Medizinische Wochenschrift 108/7 (1958), S. 154-156.

Pierre JUILLET/Roger DOREY, Insulinothérapie. In : Encyclopédie Médico-Chirurgicale, Psychiatrie 37820 C^{10}: Méthodes physiotherapique (Paris 1964), S. 1-15.

P[ierre] JUILLET, Traitements insuliniques et méthodes de choc dans la Schizophrénie. In: Confrontations psychiatriques 2 (1968), S. 107-123.

P[ierre] JUILLET, La cure de Sakel est-elle dépassée? In : Annales medico-psychologiques 138 (1980), S. 164-170.

P[ierre] JUILLET, La place actuelle de l'insulinothérapie à doses modérées en psychiatrie. In : Annales medico-psychologiques 138 (1980), S. 170-176 (mit Diskussion zu beiden Teilen der Untersuchung ebd., S. 176-179).

Rosa Maria KAINZ, Retrospektive Datenanalyse iatrogener P. vivax Infektionen von an Syphilis erkrankten PatientInnen in Wien (ungedr. med. Diplomarbeit Wien 2019).

Lothar B. KALINOWSKY, Das Problem der Hirnschädigung bei Schockbehandlungen. In: Der Nervenarzt 22/6 (1951), S. 201-203.

Robert M. KAPLAN, A history of insulin coma therapy in Australia. In: Australasian Psychiatry 21/6 (2013), S. 587-591.

Otto KAUDERS, Zur Technik der Wagner-Jaureggschen Malariatherapie in der Praxis. Über Malariablutkonservierung, in: Psychiatrisch-Neurologische Wochenschrift 28 (Halle a. S. 1926), S. 372.

Otto KAUDERS, Erfahrungen mit Pyrifer bei der Behandlung der progressiven Paralyse, in: Medizinische Kritik 25 (1929), S. 1262-1264.

Otto KAUDERS, Psychopathie und Neurose als Grenzgebiete der Nervenheilkunde. In: Wiener klinische Wochenschrift 49 (1936), S. 1-6.

Otto KAUDERS, Über die Anwendung der Malariabehandlung im Anschluß an das akute Lähmungsstadium bei Poliomyelitis epidemica. In: Medizinische Klinik. Wochenschrift für praktische Ärzte 32 (Wien 1936), S. 1729-1732 und S. 1766-1769.

Otto KAUDERS, Weitere Mitteilung über die Malariabehandlung bei Poliomyelitis. In: Medizinische Klinik. Wochenschrift für praktische Ärzte 33 (Wien 1937), S. 1464-1468 und S. 1502-1506.

Otto Kauders, Vegetatives Nervensystem und Seele (Wien 1946, [3]1947).

Otto KAUDERS, Zur Klinik, Theorie und Geschichte der Malariabehandlung. In: Wiener Zeitschrift für Nervenheilkunde und deren Grenzgebiete 1 (1948), S. 47-71.

Brigitta KEINTZEL/Eberhard GABRIEL (Hrsg.), Gründe der Seele. Die Wiener Psychiatrie im 20. Jahrhundert (Wien 1999).

Lucy Jane KING, The Best Possible Means of Benefiting the Incurable: Walter Bruetsch and the Malaria Treatment of Paresis. In: Annals of Clinical Psychiatry 12/ 4 (2000), S. 197-203.

Viktor KLARE/Walter FURTENBACH, Erfahrungen und Erfolge in der Behandlung der Poliomyelitis. In: Wiener Zeitschift für Nervenheilkunde und deren Grenzgebiete 1/ 4 (1948), S. 369-381.

V[iktor] KLARE, Rehabilitation und Poliomyelitis. Eigene Erfahrungen an einem Material von 2140 Fällen in einem Zeitraum von 10 Jahren. In: Hans HOFF (Hrsg.), Therapeutische Fortschritte in der Neurologie und Psychiatrie (Wien/Innsbruck 1960), S. 166-199.

Edith KLEMPERER, Versuch einer Behandlung des Delirium tremens mit Insulin. In: Psychiatrisch-Neurologische Wochenschrift 28 (Halle a. S. 1926), S. 549-551.

Edith KLEMPERER, Die Wirkung des Insulin beim Delirium tremens. In: Monatsschrift für Psychiatrie und Neurologie 74 (1930), S. 163-190.

F. KOHL, Wagner von Jauregg und die Entwicklung der Malariatherapie. In: Psychiatrische Praxis 20/4 (1993), S. 157-159.

Kurt KOLLE, Frage 21: Wie gestalten sich unter der Wirkung der Schocktherapie die Aussichten der Schizophrenen bezüglich Vollständigkeit und Dauer von Remissionen und bezügl. Heilung? In: Münchner Medizinische Wochenschrift (5.3.1954), S. 263.

von Kurt KOLLE, Psychiatrie. Ein Lehrbuch für Studierende und Ärzte (München/Berlin ^{4}1955).

Jesper Vaczy KRAGH, „Fumbling in the Dark“. Malaria, Sulfosin and Metallosal in the Treatment of Mental Disorders in Denmark, 1917-1937. In: Hans-Walter SCHMUHL/Volker ROELCKE (Hrsg.), „Heroische Therapien“. Die deutsche Psychiatrie im internationalen Vergleich, 1918-1945 (Göttingen 2013) 100-113.

H[einrich] Kranz, Psychopathie in ihrer Problematik. Medizinische Gesellschaft in Mainz, Sitzung am 24. Oktober 1953. In Münchner medizinische Wochenschrift 95 (1953), S. 763.

Stephen KRAUS, Insulin Therapy [in der Rubrik: Letters to the editor]. In: The Lancet (February 17, 1962), S. 382.

K[ornelius] KRYSPIN-EXNER, Zur Behandlung schizophrener Prozeßpsychosen mit Thioproperazin. In: Wiener Klinische Wochenschrift 75 (1963), S. 157-160.

Kornelius KRYSPIN-EXNER, Die moderne Behandlung des Alkoholkranken. In: Mitteilungen der österreichischen Sanitätsverwaltung 64 (1963), S. 7-8.

Kornelius KRYSPIN-EXNER. Probleme der Bekämpfung des Alkoholismus in Österreich. In: Wiener medizinische Wochenschrift (1965), S. 643-647.

Kornelius KRYSPIN-EXNER, Probleme der Führung einer offenen Anstalt für Alkoholkranke. In: Wiener Zeitschrift für Nervenheilkunde 23 (1966), S. 244-248.

K[ornelius] KRYSPIN-EXNER, Psychosen und Prozessverläufe des Alkoholismus (Wien 1966).

B[ernd] KÜFFERLE/H[elmut] SCHULTES, Übersicht über die an der Wiener Psychiatrischen Klinik im Jahr 1967 zur Sedierung verwendeten Medikamente. In: Gustav HOFMANN (Hrsg.), III. Donau-Symposium für Psychiatrie. Wien, 15.-18.5.1968. Tagungsbericht (Wien 1969), S. 71-75.

Roland KUHN, Probleme der praktischen Durchführung der Tofranil-Behandlung. In: Wiener Medizinische Wochenschrift 110/11 (1960), S. 245-250.

K[arl] KUNDRATITZ, Die Bedeutung der Encephalographie bei Krampfkrankheiten und geistigen Defekten im Kindesalter und deren therapeutische Beeinflussbarkeit, in: Wiener klinische Wochenschrift 48 (1946), S. 35-36.

K[arl] KUNDRATITZ, Die therapeutische Beeinflußbarkeit zerebralgestörter Kinder. In: Wiener klinische Wochenschrift 69 (1957), S. 423-427. Auch publiziert in. E[rwin] Domanig (Hrsg.), Zehnte Österreichische Ärztetagung Wien 28. Bis 30. September 1956. Tagungsbericht. Herausgegeben für die Van-Swieten Gesellschaft (Wien 1957), S. 232-244.

Ehrig LANGE, Kritische Darstellung der Behandlungserfolge bei syphilitischen Erkrankungen des Zentralnervensystems. In: Psychiatrie, Neurologie und medizinische Psychologie. Zeitschrift für Forschung und Praxis 6 (Leipzig 1954), S. 44-49.

Herbert LEWRENZ, Untersuchungen über den Wert der Schocktherapie bei endogenen Psychosen aus dem Formenkreis der manisch-depressiven Erkrankungen. In: Nervenarzt 22/6 (1951), S. 205-209.

Jaromir LHOTSKÝ, Kritische Betrachtung der modernen Neuroluesbehandlung. In: Der Nervenarzt 22/6 (1951), S. 228-230.

Juan LÓPEZ-IBOR, Die Therapie der Schizophrenie. In: Wiener Zeitschrift für Nervenheilkunde und deren Grenzgebiete 12/4 (1956), S. 25-34.

G. LUPASCU, Applications actuelles de la malariathérapie. In: Bull[etin de l‘] Org[anisation] Mond[ial de la] Santé / Bull[etin of the] W[or]ld H[ea]lth Org[anisation] 50 (1974), S. 165-167. https://www.ncbi.nlm.nih.gov/pmc/articles/PMC2481201/pdf/bullwho00165-0024.pdf (1.6.2024).

Jørgen MADSEN, Treatment of neurosyphilis. Introductory lecture: Malariotherapy. In: Acat Psychiatrica et Neurologica Scandinavica 25/60 (1950), S. 13-23.

A. MANDL/O. SPERLING, Ergebnisse und Indikationen der Fieberbehandlung mit Pyrifer bei Nervenkrankheiten. In: Wiener klinische Wochenschrift 42 (1929), S. 169-172.

E. MARLEY (Institue of Psychiatry, Maudslex Hospital, London), Insulin Therapy [in der Rubrik: Letters to the editor], in: The Lancet (March 3, 1962) 485.

James Purdon MARTIN, The treatment of neurosyphilis with penicillin. In: British Journal of Veneral Diseases 24/3 (1948), S. 89-100.

James Purdon MARTIN et al., Discussion on the treatment of neurosyphilis with penicillin. In: British Journal of Veneral Diseases 24/3 (1948), S. 100-103.

Karol MATULAY, Liečenie syfilisu nervstva penicilínom. Klinické a laboratórne výsledky. Zusammenfassung: Die Behandlung der Syphilis des Nervensystems mit Penicillin (Bratislava 1957), S. 175-182.

Heinz MAYRHOFER, Besuch in der Bundesanstalt für Erziehungsbedürftige in Wiener Neudorf. In: Verein der Ärzte in der Justizverwaltung, Mitteilungen, Jg. 2 (1958/59) Heft 3, S. 73f.

Niall MCCRAE, ‘A violent thunderstorm’: Cardiazol treatment in British mental hospitals. In: History of Psychiatry 17/1 (2006), S. 67-90.
http://hpy.sagepub.com/content/17/1/67.full.pdf+html (24.1.2015)

Marietta MEIER/Brigitta BERNET/Roswitha DUBACH/Urs GERMANN (Hrsg.), unter Mitarbeit von Gisela HÜRLIMANN, mit einem Schlusswort von Jakob TANNER, Zwang zur Ordnung. Psychiatrie im Kanton Zürich, 1870-1970 (Zürich 2007).

Marietta MEIER/Mario KÖNIG/Magaly TORNAY, Testfall Münsterlingen. Klinische Versuche in der Psychiatrie, 1940 – 1980. Unter Mitarbeit von Ursina Klauser (Zürich 2019).

Léon MICHAUX, Psychiatrie (= Collection médico-chirurgicale à révision périodique 43, Paris 1965).

Jean-Noël MISSA, Naissance de la psychiatrie biologique. Histoire des traitements de maladies mentales aux XXe siècle (Paris 2006).

Hans-Jürgen MÖLLER/Gerd LAUX/Arno DEISTER, Psychiatrie und Psychotherapie (= Duale Reihe, Stuttgart [4]2009).

Max MÜLLER, Prognose und Therapie der Geisteskrankheiten (Stuttgart 1949).

Max MÜLLER, Die körperlichen Behandlungsverfahren in der Psychiatrie: Ein Lehr- und Handbuch Bd. 1: Die Insulinbehandlung (Stuttgart 1952).

Max MÜLLER, Grundlagen und Methodik der somatischen Behandlunggsverfahren in der Psychiatrie: Einleitung (1963), S. 385 (Einleitung zum Kapitel über die Somatotherapien), in: Bally et al. (Hrsg.), Grundlagen und Methoden der klinischen Psychiatrie (= H. W. GRUHLE/R. JUNG/W. MAYER-GROSS/M. MÜLLER (Hrsg.), Psychiatrie der Gegenwart: Forschung und Praxis, Bd. I/2, Berlin/Göttingen/Heidelberg 1963), S. 384-387.

Max MÜLLER, Die Insulinbehandlung (1963), in: Bally et al. (Hrsg.), Grundlagen und Methoden der klinischen Psychiatrie (= H. W. GRUHLE/R. JUNG/W. MAYER-GROSS/M. MÜLLER (Hrsg.), Psychiatrie der Gegenwart: Forschung und Praxis, Bd. I/2, Berlin/Göttingen/Heidelberg 1963), S. S. 389-414.

Manfred MÜLLER-KÜPPERS, Die Therapie im Kindes- und Jugendalter. In: K. P. KISKER/J.-E. MEYER/M. MÜLLER/E. STRÖMGREN (Hrsg.), Psychiatrie der Gegenwart. Forschung und Praxis Bd. II / Teil 1 (Berlin/Heidelberg/New York 1972), S. 977-1006.

N. N., 10-Jahresbericht der Psychiatrisch-Neurologischen Kinderstation an der Wiener Universitätsklinik für Psychiatrie und Neurologie. In: Wiener Zeitschrift für Nervenheilkunde und deren Grenzgebiete 19 (1962), S. 215-220.

C. S. NICOL, The treatment of neurosyphilis. In: Postgraduate Medical Journal 29 (Jannuary 1953), S. 27-33.

W. D. NICOL, Treatment of neurosyphilis: a comparison between malaria plus tryparsamide and malaria therapy. In: British Journal of Venereal Diseases 22/3 (1946), S. 112-121.

W. D. NICOL, General paralysis of the insane. In: British Journal of Venereal Diseases 32/9 (1956), S. 9-16.

W. D. NICOL, Julius Wagner von Jauregg, M.D., 1857-1940. An appreciation. In: British Journal of Venereal Diseases 33/2 (1957), S. 125-126.

S. J. NIJDAM, Die therapeutische Wirkung der Neuroleptika allein und in Kombination mit anderen Therapien. In: Wiener Medizinische Wochenschrift 110/36 (1960), S. 722-725.

Thorsten NOACK, Über Kaninchen und Giftschlangen: Psychiatrie und Öffentlichkeit in der frühen Bundesrepublik Deutschland. In: Heiner FANGERAU / Karen NOLTE (Hrsg.), „Moderne“ Anstaltspsychiatrie im 19. und 20. Jahrhundert – Legitimation und Kritik (Stuttgart 2006), S. 311-340.

Carl Hans PAHNKE, Ueber den Einfluss der Fiebertherapie auf die Behandlung der Psoriasis vulgaris (ungedr. med. Dissertation Wien 1941).

Erich F. PAKESCH, Spezielle Therapie der Nervenkrankheiten. In: Wolfgang Holzer (Hrsg.), Therapie der Nerven- und Geisteskrankheiten (Wien 1951), S. 153-212.

Erich F. PAKESCH, Therapie der Geisteskrankheiten. In: Wolfgang HOLZER (Hrsg.), Therapie der Nerven- und Geisteskrankheiten (Wien 1951), S. 213-248

Harold PALMER, Psychopatic personality. Definition and use of the term. In: The Lancet, 3.1.1959, S. 40-41.

Emilio PAMPANA, A Textbook of Malaria Eradication (London/New York/Toronto 1963).

H. PEMSL, Über die „Schockangst“. Vorläufige Mitteilung. In: Wiener klinische Wochenschrift 69 (1957), S. 748f.

E[rnst] PICHLER, Ambulante Behandlung von Depressionszuständen mit Tofranil. In: Wiener Medizinische Wochenschrift 110/36 (1960), S. 753-754.

Pierre PICHOT, Vergleich der verschiedenen Behandlungsmethoden in der Psychiatrie (E-Schock, Insulin-Schock, Behandlung mit verschiedenen Neuroleptika allein). In: Wiener Medizinische Wochenschrift 110/36 (1960), S. 734-737.

Alexander PILZ, Malaria inoculation in cases of dementia praecox (1930), in: Der Nervenarzt 3 (1930), S. 58.

O[tto] PÖTZL, Die Wirkweise der Schockbehandlungen. In: Wiener Medizinische Wochenschrift 97 (1947), S. 11-12.

Otto PÖTZL, Leukotomie und Vegetativum. In: Acta Neurovegetativa 1 (1950), S. 317-341.

Otto PÖTZL, Widmung an Hans Hoff (1958). In: Wiener Zeitschrift für Nervenheilkunde und deren Grenzgebiete 15 (1958), S. 5-14. (Der Band ist Hans Hoff zum 60. Geburtstag gewidmet).

Antoine POROT, Manuel alphabétique de Psychiatrie clinique, thérapeutique et médico-légale (Paris 1952, ²1960, ³1965).

Joachim RADKAU, Zum historischen Quellenwert von Patientenakten. Erfahrungen aus Recherchen zur Geschichte der Nervosität, in: Dietrich Meyer/BerndHey, Akten betreuter Personen als archivische Aufgabe. Beratungs- und Patientenakten im Spannungsfeld von Persönlichkeitsschutz und historischer Forschung (Neustadt an der Aisch 1997), S. 73-101.

Herbert RAU, Behandlung der Gonorrhöe[!] im Kindesalter mit Impfmalaria. In: Zeitschrift für Kinder-Heilkunde 50 (1930), S. 121–124 (https://doi.org/10.1007/BF02248226, 19.5.2023).

Jörgen RAVN, Truxal, ein neuartiges Psychopharmacon. In: Wiener Klinische Wochenschrift 72 (1960), S. 192-196.

Dietrich REIMOLD, Retrospektive Datenanalyse iatrogener P. vivax Infektionen an der Wiener Psychiatrie (Dipl. A. 2020).

Ludwig REITER/Eberhard GABRIEL, Diagnose „Psychopathie“ und diagnostischer Prozeß bei Jugendlichen. In: Hans STROTZKA (Hrsg.), Neurose, Charakter, soziale Umwelt. Beiträge zu einer speziellen Neurosenlehre (München 1973), S. 119-138.

J. H. REY/D. A. POND/C. C. EVANS, Clinical and Electroencephalographic Studies of Temporal Lobe Function. In: Proceedings of the Royal Society of Medicine 42 (1949), S. 891-904.

Erwin RINGEL, Der Selbstmord. Abschluß einer krankhaften psychischen Entwicklung (Eine Untersuchung an 745 geretteten Selbstmördern) (Wien/Düsseldorf 1953).

Erwin Ringel/ Wilhelm Solms/ Walter Spiel, Die Therapie der Psychopathie. In: Hans Hoff (Hrsg.), Therapeutische Fortschritte in der Neurologie und Psychiatrie (Wien/Innsbruck 1960), S. 453-463.

J. RISTIC (Belgrad), Kurze Übersicht über einige mit Tofranil ambulant behandelte Fälle leichter Depressionen. In: Wiener Medizinische Wochenschrift 110/36 (1960), S. 751-752.

G[ottfried] ROTH/Th. MÜLLER, Zur Tofranilbehandlung der Depressionszustände. Ein Beitrag zur optimalen Anwendung pharakopsychiatrischer Medikamente. In: Wiener Klinische Wochenschrift 72/13 (1960), S. 225-227.

David J. ROTHMAN, Strangers at the bedside. A history of how law and bioethics transformed medical decision making (London [4]1998).

Lara RZESNITZEK, Lothar B. Kalinowsky und die Einführung der Elektrokrampftherapie in Europa. Die verflochtene Geschichte eines „zufälligen“ Sufbruchs „wider Willen“? . In: Hans-Walter SCHMUHL/Volker ROELCKE (Hrsg.), „Heroische Therapien.“ Die deutsche Psychiatrie im internationalen Vergleich, 1918-1945 (Göttingen 2013), S. 200-215.

Lara RZESNITZEK, Schocktherapien und Psychochirurgie in der frühen DDR. In: Nervenarzt 86 (2015), S. 1412-1419.

Lara RZESNITZEK, „Schocktherapien” und Leukotomie in der DDR-Psychiatrie. In: Ekkehardt KUMBIER/Holger STEINBERG (Hrsg.), Psychiatrie in der DDR. Beiträge zur Geschichte (Berlin-Brandenburg 2018), S. 289-299.

Professor Manfred SAKEL of New York Celebrated in Vienna. In: Medical Cercle Bulletin 4 (1957), S. 10-11.

William SARGANT/Eliot SLATER, An Introdurction to Physical Methods of Treatment in Psychiatry (Edinburgh 1946).

William SARGANT/Eliot SLATER, Die modernen psychiatrischen Behandlungsmethoden (Göttingen [1951]).

William SARGANT, Insulin in early schizophrenia. In: The Lancet, 28.9.1957 [letter to the editor], S. 644 f.

William SARGANT/Eliot SLATER, An Introduction to Physical Methods of Treatment in Psychiatry (New York [5]1972).

H. SASS/S. HERPETZ, Personality Disorders: Clinical Section. In: German E. BERRIOS/Roy PORTER (Hrsg.), A History of clinical psychiatry. The Origin and History of Psychiatric Disorder (paperback ed. London u.a.1999), S. 633-644.

H[elmut] SCHARFETTER, Die “freiwillige Aufnahme” in eine geschlossene Anstalt. In: Wiener klinische Wochenschrift 70 (1958) 661-662.

H[elmut] SCHARFETTER, Die Bewährung des österreichischen Anhaltungsverfahrens. In: Wiener klinische Wochenschrift 71 (1959), S. 21-29.

O[tto] SCHILLER/W[ilhelm] SOLMS, Neue Methoden in der Behandlung des chronischen Alkoholismus. In: Wiener klinische Wochenschrift 61 (1949), S. 536-539.

Anna SCHISCHITZA, Einige Betrachtungen zur Kooperation an der psychiatrisch-neurologischen Kinderabteilung. In: Wiener Zeitschrift für Nervenheilkunde und deren Grenzgebiete (1962), S. 185-190.

R[aoul] SCHINDLER, Fortschritte der Psychochirurgie. I. Klinischer Teil. In: Hans HOFF (Hrsg.), Therapeutische Fortschritte in der Neurologie und Psychiatrie (Wien/Innsbruck 1960), S. 470-481.

Sepp SCHINDLER, Jugendkriminalität. Struktur und Trend in Österreich 1945 – 1965 (Wien/München 1968).

H. SCHINKO/E. SLUGA-GASSER/H. TSCHABITSCHER, Therapie der Multiplen Sklerose. In: Hans Hoff (Hrsg.), Therapeutische Fortschritte in der Neurologie und Psychiatrie (Wien/Innsbruck 1960), S. 49-73.

N. SCHIPOKOWENSKY, Die Behandlung der Schizophrenie und der Zyklophrenie mit Heilkrampf, Insulinschock und Chlorpromazin. In: Wiener Medizinische Wochenschrift 110/36 (1960), S. 745-747.

Hans-Walter SCHMUHL/Volker ROELCKE (Hrsg.), „Heroische Therapien.“ Die deutsche Psychiatrie im internationalen Vergleich, 1918-1945 (Göttingen 2013).

Hans-Walter SCHMUHL/Volker ROELCKE, Einleitung. In: Hans-Walter SCHMUHL/Volker ROELCKE (Hrsg.), „Heroische Therapien.“ Die deutsche Psychiatrie im internationalen Vergleich, 1918-1945 (Göttingen 2013), S. 19-24.

Heinz SCHOTT/Rainer TÖLLE, Geschichte der Psychiatrie. Krankheitslehren, Irrwege, Behandlungsformen (München 2006).

Walter SCHULTE/Rudolf DREYER, Eine cerebrale tödliche Komplikation nach Elektroschock. In: Der Nervenarzt 4 (1950), S. 175-178.

Walter SCHULTE, Fortschritte der Somatotherapie bei Psychosen während der letzten 10 Jahre. In: Medizinische Klinik 9 (1959), S. 256-360.

Walter SCHULTE/Rainer TÖLLE, Psychiatrie (Berlin/Heidelberg/New York 1971).

Siegfried SCHWARTZ, Zur Frage der Malariatherapie der Gonorrhoe (ungedr. med. Dissertation Innsbruck [1941]).

Edward SHORTER, Geschichte der Psychiatrie. Aus dem Amerikanischen von Yvonne Badal (Berlin 1999).

Edward SHORTER/David HEALY, Shock Therapy. A History of Electroconvulsive Treatment in Mental Illness (New Brunswick/New Jersey/London 2007).

Reinhard SIEDER/Andrea SMIOSKI/Holger EICH/Sabine KIRSCHENHOFER, Der Kindheit beraubt. Gewalt in den Erziehungsheimen der Stadt Wien (1950er bis 1980er Jahre) (Innsbruck 2012).

Willibald SLUGA, Jugendpsychiatrische Tätigkeit bei Verwahrlosten. In: Wiener Zeitschrift für Nervenheilkunde und deren Grenzgebiete 19 (1962), S. 210-214.

Hugo SOLMS, Die Krampfbehandlung, in: in: Bally et al. (Hrsg.), Grundlagen und Methoden der klinischen Psychiatrie (= H. W. GRUHLE/R. JUNG/W. MAYER-GROSS/M. MÜLLER (Hrsg.), Psychiatrie der Gegenwart: Forschung und Praxis, Bd. I/2, Berlin/Göttingen/Heidelberg 1963), S. 415-494.

Wilhelm SOLMS, Zum Psychopathieproblem. In: Wiener Archiv für Psychologie, Psychiatrie und Neurologie 1 (1951), S. 24-35.

Wilhelm SOLMS in einer Diskussion zu Marsilid (Iproniazid) nach Vorträgen von H. Walter-Büel (Müsingen) und C. G. Tauber (Bern) in der Gesellschaft der Ärzte in Wien, in: Wiener klinische Wochenschrift 71 (1959), S. 108.

Wilhelm SOLMS, Moderne Therapie des Alkoholismus. In: Hans HOFF (Hrsg.), Therapeutische Fortschritte in der Neurologie und Psychiatrie (Wien/Innsbruck 1960), S. 402-413.

Walter SPIEL, Über den Beginn neurotischer und psychopathischer Entwicklungen. Vorläufige Mitteilung. In: Wiener Zeitschrift für Nervenheilkunde und deren Grenzgebiete 9 (1954), S. 21-28.

Walter SPIEL, Über jugendpsychiatrische Erfahrungen im Jugendstrafvollzug. In: Mitteilungen. Verein der Ärzte in der Justizverwaltung 1/4 (1956/57), S. 28-37.

Walter SPIEL, Beitrag zur Frage der psychopathischen und neurotischen Entwicklung im Kindesalter. In: Wiener Zeitschrift für Nervenheilkunde und deren Grenzgebiete 15 (1958), S. 291-297.

Walter SPIEL, unter Mitarbeit von Erika HIFT, Marta KOS, Anna SCHISCHITZA, Die Psychotherapie im Kindes- und Jugendalter. In: Hans HOFF (Hrsg.), Therapeutische Fortschritte in der Neurologie und Psychiatrie (Wien/Innsbruck 1960), S. 432-447.

Lona und Walter SPIEL, Zur Therapie des Schwachsinns. In: Hans HOFF (Hrsg.), Therapeutische Fortschritte in der Neurologie und Psychiatrie (Wien/Innsbruck 1960), S. 447-452.

Walter SPIEL, Die endogenen Psychosen des Kindes- und Jugendalters (= Bibliotheca psychiatrica et neurologica 113, Basel/Wien 1961).

Walter SPIEL, Die Therapie in der Kinder- und Jugendpsychiatrie (Stuttgart 1967).

W[alter] SPIEL, Nachuntersuchungsergebnisse psychopathischer und neurotischer Entwicklungen. In Wiener Medizinische Wochenschrift 117 (1967), S. 1166-1169.

Walter SPIEL, 25 Jahre Neuropsychiatrie des Kindes- und Jugendalters in Wien (1951-1976). Beilage zur Zeitschrift für Kinder- und Jugendpsychiatrie 1 (1977).

Walter SPIEL/R. MUTSCHLECHNER/V. SCHAUFLER/I. STÜTZ, Die Entstehung des Fachgebietes Kinder- und Jugendneuropsychiatrie und der Universitätsklinik für Neuropsychiatrie des Kindes- und Jugendalters an der Medizinischen Fakultät der Universität Wien. Versuch einer Dokumentation (Wien 1994).

Alfred SPRINGER, Pschopharmakologische Forschung und Behandlung an der Wiener Psychiatrischen Universitätsklinik und die Frühphase des Collegium Internationale Neuro-Psychopharmacologicum (CINP). In: Virus. Beiträge zur Sozialgeschichte der Medizin 14 (Leipzig 2016), S. 221-237.

Marianne SPRINGER-KREMSER, Die Neukonstituierung der Psychotherapeutischen Schulen und der Beginn der Akademisierung der Psychotherapie. In: Virus. Beiträge zur Sozialgeschichte der Medizin 14 (Leipzig 2016), S. 191-206.

Rolf STRÖM-OLSEN (Runwill Hospital in Wickford, Essex), Insulin Therapy [in der Rubrik: Letters to the editor]. In: The Lancet (January 6, 1962), S. 47.

Jakob TANNER/Marietta MEIER/Gisela HÜRLIMANN/Brigitta BERNET, Zwangsmassnahmen in der Züricher Psychiatrie 1870 – 1970. Bericht im Auftrag der Gesundheitsdirektion des Kantons Zürich (Typoskript Zürich 2002)

Jakob TANNER, Ordnungsstörungen: Konjunkturen und Zäsuren in der Geschichte der Psychiatrie. In: Marietta MEIER/Brigitta BERNET/Roswitha DUBACH/Urs GERMANN (Hrsg.), unter Mitarbeit von Gisela HÜRLIMANN, mit einem Schlusswort von Jakob TANNER, Zwang zur Ordnung. Psychiatrie im Kanton Zürich, 1870-1970 (Zürich 2007), S. 271-306.

Clement Price THOMAS/A. M. CLAY, Treatment of the Psychopath. In: The Lancet (2.8.1958), S. 249-252.

M[oritz] TRAMER, Zur Frage der Somatopsychik, In: Wiener Zeitschrift für Nervenheilkunde und deren Grenzgebiete 19 (1962), S. 166-171.

Hubert J. URBAN (Hrsg.), Festschrift zum 70. Geburtstag von Prof. Dr. Otto Pötzl (Innsbruck 1947).

Jost VIJSELAAR, „A Hole in the Armour of Dementia Praecox“. Somatic Cures within a Context of *Psychiatry in Multiplicity:* the Netherlands 1920-1950. In: Hans-Walter SCHMUHL/Volker ROELCKE (Hrsg.), „Heroische Therapien.“ Die deutsche Psychiatrie im internationalen Vergleich, 1918-1945 (Göttingen 2013), S. 168-184.

Julius WAGNER-JAUREGG, Fieber- und Infektionstherapie. Ausgewählte Beiträge 1887-1935. Mit verknüpfenden und abschließenden Bemerkungen (Wien/Leipzig/Bern 1936).

Julius WAGNER-JAUREGG, Über die Einwirkung der fieberhaften Erkrankungen auf Psychosen [aus: Jahrbücher für Psychiatrie7 (1887) 94-131]. In: Julius WAGNER-JAUREGG, Fieber- und Infektionstherapie. Ausgewählte Beiträge 1887-1935. Mit verknüpfenden und abschließenden Bemerkungen (Wien /Leipzig /Bern 1936) S. 1-57.

Julius WAGNER-JAUREGG, Über die Einwirkung der Malaria auf die progressive Paralyse [aus: Psychiatrisch-neurologische Wochenschrift, Jg. 1918/19, Nr. 21/22, S. 132-134 und Nr.

39/40, S. 251-255]. In: Julius WAGNER-JAUREGG, Fieber- und Infektionstherapie. Ausgewählte Beiträge 1887-1935. Mit verknüpfenden und abschließenden Bemerkungen (Wien/Leipzig/Bern 1936), S. 117-133, Bemerkungen [1936], S. 133-138.

Julius WAGNER-JAUREGG, Der Mechanismus der Wirkung der Infektions- und Fiebertherapie [aus: Wiener Klinische Wochenschrift 1935, Nr. 14]. In: Julius WAGNER-JAUREGG, Fieber- und Infektionstherapie. Ausgewählte Beiträge 1887-1935. Mit verknüpfenden und abschließenden Bemerkungen (Wien/Leipzig/Bern 1936), S. 271-287.

Julius WAGNER-JAUREGG, Über spezifische und unspezifische Behandlung von Geisteskrankheiten. [aus: Deutsche Zeitschrift für Nervenheilkunde 1931, S. 672-674], in: Julius WAGNER-JAUREGG,, Fieber- und Infektionstherapie. Ausgewählte Beiträge 1887-1935. Mit verknüpfenden und abschließenden Bemerkungen (Wien/Leipzig/Bern 1936), S. 288-293.

H. WALTHER-BÜEL, Zur klinischen Therapie der endogenen Psychosen. In: Der Nervenarzt 25 (1954), S. 191-194.

H. WALTHER-BÜEL, Marsilid in der Psychiatrie (Vortrag). In: Wiener klinische Wochenschrift 71 (1959), S. 107.

A. WAND, Die Fieberbehandlung bei der genuinen Epilepsie. Vorläufige Mitteilung. In: Psychiatrie, Neurologie und medizinische Psychologie. Zeitschrift für Forschung und Praxis (= Mitteilungsorgan der Gesellschaft für Psychiatrie und Neurologie der DDR) 17 (1965), S. 17-19.

G. L. WARNER, Malaria inoculation in cases of dementia praecox. In: Psychiatric Quarterly 2 (1928), S. 494.

Carlos WATZKA, Die „Fälle“ Wolfgang Holzer und Hand Bertha sowie andere „Personalia“. Kontinuitäten und Diskontinuitäten in der Grazer Psychiatrie 1945-1970. In: Virus. Beiträge zur Sozialgeschichte der Medizin 14 (Leipzig 2016), S. 103-138.

Klara WEINGARTEN, Die derzeitige Therapie der Neurolues an der Wiener Nervenklinik. In: Wiener Medizinische Wochenschrift 102/48 (1952), S. 953-955.

Klara WEINGARTEN, Zur Therapie der Neurolues. In: Wiener klinische Wochenschrift 69 (1957), S. 722-725.

K[lara] Weingarten, Therapie der Neurolues. In: Hans Hoff (Hrsg.), Therapeutische Fortschritte in der Neurologie und Psychiatrie (Wien/Innsbruck 1960), S. 233-250.

Wolf Ludwig WEINLAND, Zur Frage der Frakturhäufigkeit bei der Elektroschockbehandlung, in: Der Nervenarzt 8 (1951), S. 298-302.

G[erhard] WEISER (Linz), Erfahrungen mit dem Butyrophenonpräparat Luvatren in der Behandlung der Schizophrenie. In: Wiener Medizinische Wochenschrift 118 (1968), S 444-446.

Magda WHITROW, Wagner-Jauregg and the fever therapy, in: Medical History 34 (1990), S. 294-310.

Urban WIESING (Hrsg.), Ethik in der Medizin. Ein Studienbuch (4., erweiterte und vollständig durchgesehene Auflage, Stuttgart 2012).

E. WISSFELD/E. KAINDL, Über die Deutung und den Wert abnormer EEG-Befunde bei psychopathischen Persönlichkeiten. In: Der Nervenarzt 32 (1961), S. 57-66.

Simone WOLF, Rosa Maria KAINZ, Dietrich REIMOLD, Haimo LAGLER, Johannes MISCHLINGER, Michael RAMHARTER, Clinical and parasitological characteristics of *Plasmodium vivax* malaria in malaria-naïve patients: A review of malaria fever therapy in patients with schizophrenia and neurosyphilis during the 1950s and 1960s in Vienna, Austria, in: American Journal of Tropical Medicine and Hygiene (AJTMH), submitted 2025.

J[ohann] ZEITLHOFER/H[elmut] TSCHABITSCHER/Th. WANKO, Zur Pathologie des protrahierten Insulinschocks. In: Wiener Zeitschrift für Nervenheilkunde und deren Grenzgebiete 9 (1954), S. 445-458.

Verzeichnis der Medikamente – Handelsname bzw. Wirkstoff / chemische Kurzbeschreibung[1211]

C2103 (Amitriptylin im Versuchsstadium, Handelsname: Tryptizol, Saroten)

Fr33 (wie Haloperidol / Haldol und Luvatren ein Neuroleptikum aus der Stoffgruppe Butyrophenone, das im Versuchsstadium blieb)

NPL 82 (1965 Studienname für eine neues Neuroleptikum)

N714 (Studienname nicht aufzulösen)

4560 RP, 45-60, 45/60 (Studienname für Largactil)

SD 709 (Studienname für Istonil)

7843 (Studienname für Majeptil oder ein verwandtes Neuroleptikum)

P46 (Studienname nicht aufzulösen, Neuroleptikum)

T.L.8 (Chlorpromazin wie Largactil?)

T.L.10 (Chlorpromazin wie Largactil?)

Weitere, nicht aufgelöste Studiennamen: TP21, AT10, A66, D23, H61, N714, PH21, T57, P1133, S771

Acetarsol (Handelsname: Stovarsol)

Acetylcholin (Neurotransmitter)

Agrypnal (Wirkstoff: Phenobarbital)

[1211] Zu den Informationen zu den Medikamenten wurden vor allem die beiden Ausgaben von BENKERT/HIPPIUS, Psychiatrische Pharmakotherapie (1974 und 1986), sowie das Internet herangezogen.

Akineton (Wirkstoff: Anticholinergikum Biperiden)
Akrinor (Wirkstoffkombination: Cafedrin + Theodrenalin)
Amitriptylin (im Versuchsstadium: C2103; Handelsname: Tryptizol, Saroten)
Amobarbital (Wirkstoff: Barbiturat)
Antabus (Wirkstoff: Tetraäthylthiuramdisulfid Disulfiram)
Atebrin (Wirkstoff: Antiprotozoikum Mepacrin)

B-Komplex+Pentovitol (Wirkstoff: Calciumpantothenat)
Barbital (Handelsname: Medinal; Veronal)
Barbiturate (Barbitursäureabkömmlinge mit dämpfender Wirkung auf das Zentralnervensystem, mit verschiedenen Handelsnamen wie: Medinal, Plexonal, Agrypnal, Cyclobarbital, Nembutal, Prominal, Secomal, Pentothal)
Belladenal (Wirkstoff: Phenobarbital)
Benzodiazepin Diazepan (Handelsname: Valium)
Benzylpenicillin (Handelsname: Hypropen)
Bernsteinsäureester M115 (Handelsname: Lysthenon)
Biobamat (Wirkstoff: Meprobamat)
Biperiden (Handelsname: Akineton)
Bulbocapnin (Aporphin-Alkaloid, ähnelt strukturell dem Apomorphin)
Butyrophenone (Handelsname: Haloperidol, Haldol, Luvatren, Moperon)
Butyrylperazin (Handelsname: Randolectil)

Cafedrin kombiniert mit Theodrenalin (Handelsname: Akrinor)
Carbamazepin (Handelsname: Tergetol)
Cardenolid-Glykosid (Handelsname: Strophanthin)
Chloraldurat (Wirkstoff: Aldehydhydratderivat Chloralhydrat)
Chloralhydrat (Handelsname: Chloraldurat)
Chlordiazepoxid (Handelsname: Librium)

Chloroquin (Handelsname: Resochin)
Chlorpromazin (Handelsname: Largactil, Megaphen)
Chlorprothixen (Handelsname: Truxal)
Clomethiazol (Handelsname: Distraneurin)

Clopenthixol (Handelsname: Sordinol)

Co-Dergocrinmesila (Handelsname: Hydergin)
Coramin (Wirkstoff: Nikethamid)
Cyclobarbital (Wirkstoff: Barbiturat)

Decentan (Wirkstoff: Prophenazin)
Depressin (Wirkstoff: Ganglienblocker Hexamethonium)
Desoxycorticosteronacetat (Handelsname: Doca)
Destrydon (Wirkstoff: Depot Strychnin)
Diazepam (Handelsname: Valium)
Dibenzazepine (vgl. Imipramin bzw. Tofranil)
Dimetacrin (Handelsname: Istonil; Studienname SD 709)
Dipenzepin (Handelsname: Noveril)
Distraneurin (Wirkstoff: Clomethiazol)
Disulfiram (Handelsname: Antabus)
Doca (Wirkstoff: Desoxycorticosteronacetat)
Dominal forte (im Versuchsstadium: Homburg D 206; Wirkstoff: Azaphenothiazinderivat Prothipendyl)
Doriden (Wirkstoff: Piperidinderivat Glutethimid)

Effortil (Wirkstoff: Etilefrin hydrochlorid)
Encephabol (Wirkstoff: Pyritinol)
Epilan (Wirkstoff: Phenytoin)
Etilefrin hydrochlorid (Handelsname: Effortil)

Glucagon (Peptidhormon)
Glutethimid (Handelsname: Doriden)

Haldol (Wirkstoff: Butyrophenonderivat Haloperidol)
Haloperidol (Handelsname: Haldol, Haloperidol)
Harmin (Alkaloid aus der Gruppe der Harman-Alkaloide)
Heptadon (Wirkstoff: Methadon)

Hexamethonium, Ganglienblocker (Handelsname: Depressin)
Homburg D 206 (Bezeichnung im Versuchsstadium, Wirkstoff: Prothipendyl-Hydrochlorid; Handelsname: Dominal forte)
Hydergin (Wirkstoff: Co-Dergocrinmesila)
Hyporpen (Wirkstoff: Benzylpenicillin)

Imipramin (Handelsname: Tofranil)
Insidon (Wirkstoff: Opipramol)
Iproniazid (MOI, Handelsname: Marsilid)
Istonil (Wirkstoff: Dimetacrin)

Kemadrin (Wirkstoff: Procyclidin)

Largactil (Wirkstoff: Chlorpromazin; Studienname 4560 RP)
Leptocurare (Handelsname [fr.]: Brévatonal, Brévicurarine, Célocurine)
Levomepromazin (Handelsname: Nozinan, Neurocil)
Librium (Wirkstoff: Benzodiazepinderivat Clordiazepoxid)
LSD (Lysergsäurediethylamid)
Luminal (Wirkstoff: Phenobarbital)
Luvatren(a) (Wirkstoff: Butyrophenonderivat Methylperidol*)*
Lysthenon (Wirkstoff: Bernsteinsäureester M115, synthetisiertes Succinylcholin)

Majeptil (Wirkstoff: Phenothiazinderivat Thioproperazin)
Marsilid (Iproniazid)
Medinal (Wirkstoff: Barbital)
Megaphen (Wirkstoff: Chlorpromazin)
Melleretten (Wirkstoff: Phenothiazinderivat Thioridazin)
Melleril (Wirkstoff: Phenothiazinderivat Thioridazinhydrochlorid)

Mepacrin (Handelsname: Atebrin)
Meprobamat (Handelsname: Miltaun, Biobamat)
Meskalin (Alkaloid aus der Stoffgruppe der Phenethylamine)
Methadon (Handelsname: Methadon)

Methylphenidat (Handelsname: Ritalin)
Methylphenobarbital (Handelsname: Prominal)
Mevasine (Wirkstoff: Mecamylamine)
Miltaun (Wirkstoff: Meprobamat)

Narkothion (Wirkstoff: Thiobutabarbital sodium)
Nembutal (Wirkstoff: Pentobarbital-Natrium)
Neurocil (Wirkstoff: Phenothiazinderivat Levomepromazin)
Niamid (Wirkstoff: MOI Nialamid)
Nikethamid (Handelsname: Coramin)
Noveril (Wirkstoff: Dipenzepin)
Nozinan (Wirkstoff: Phenothiazinderivat Levomepromazin)

Opipramol (Handelsname: Insidon)

Paraldehyd (Sedativum, Hypnotikum; Trimer des Acetaldehyds)
Parsidol (Phenothiazin-Derivat Profenamin)
Penicillin (Handelsname: Hypropen)
Pentobarbital (Handelsname: Nembutal)
Pentothal (Wirkstoff: Thiopental aus der Reihe der Barbiturate)
Percorten (Wirkstoff: Desoxycorticosterone pivalate)
Phenergan (Wirkstoff: Phenothiazinderivat Promethazin)
Phenobarbital (Handelsname: Luminal, Belladenal, Agrypnal)
Phenothiazin (Handelsname: Nozinan)
Phenytoin (Handelsname: Epilan)
Plexonal (Wirkstoffe: Dihydroergotamin + Scopolamin + Barbital)
Prochlorperazin (Phenothiazin)
Procyclidin (Handelsname: Kemadrin)
Profenamin (Handelsname: Parsidol)

Promethazin (Handelsname: Phenergan)
Prominal (Wirkstoff: Methylphenobarbital)
Prophenazin (Handelsname: Decentan)

Prothipendyl (im Versuchsstadium: Homburg; Handelsname: Dominal forte)
Pyritinol (Handelsname: Encephabol)

Randolectil (Wirkstoff: Butyrylperazin)
Rauwolfia-Alkaloid Reserpin (indische Schlangenwurzel) (Handelsnamen: Serpasil)
Reserpin, Rauwolfia-Alkaloid (Handelsname: Serpasil, Reserpin, Phasein)
Resochin (Wirkstoff: Chloroquin)
Ritalin (Wirkstoff: Methylphenidat)

Saroten (Wirkstoff: Amitriptylin)
Scopolamin / Hyoscin (Tropan-Alkaloid)
Secomal (Wirkstoff: Barbiturat)
Serotonin (Gewebshormon und Neurotransmitter)
Serpasil (Wirkstoff: Rauwolfia-Alkaloid Reserpin)
Somnifen (Barbiturat)
Sordinol (Wirkstoff: Clopenthixol)
Strophanthin (Wirkstoff: Cardenolid-Glykosid)
Depot Strychnin (Handelsname: Destrydon)
Stovarsol (Wirkstoff: Acetarsol, aus der Gruppe der org. Arsenverbindungen)
Strychnin / Depot- (Handelsname: Destrydon)
Sympathol (Wirkstoff: Phenylethylaminderivat Synephrin)
Synephri (Handelsname: Sympatol)

Tergetol (Carbamazepin; zählt chemisch zur Klasse der Dibenzazepine)
Theodrenalin kombiniert mit Cafedrin (Handelsname: Akrinor)
Thiobutabarbital sodium (Barbiturat; Handelsname: Narkothion)
Thiopental (Barbiturat; Handelsname: Pentothal)
Thioproperazin (Handelsname: Majeptil; Studienname 7843)
Thioridazinhydrochlorid (Handelsname: Melleretten, Melleril)
Thymoleptikum (Synonym für Antidepressivum)
Tofranil (Wirkstoff: Dibenzazepinderivat Imipramin)
Trifluperidol (Handelsname: Triperidol)
Triperidol (Wirkstoff: Butyrophenonderivat Trifluperidol)

Truxal (Wirkstoff: Chlorprothixen)
Tryparsamid (Arsenverbindung)
Tryptizol (Wirkstoff: Amitriptylin)

Valium (Wirkstoff: Benzodiazepin Diazepam.)
Veronal (Wirkstoff: Barbital)
